# Parental micronutrient deficiency distorts liver DNA methylation and expression of lipid genes associated with a fatty-liver-like phenotype in offspring

**DOI:** 10.1038/s41598-018-21211-5

**Published:** 2018-02-14

**Authors:** Kaja H. Skjærven, Lars Martin Jakt, Jorge M. O. Fernandes, John Arne Dahl, Anne-Catrin Adam, Johanna Klughammer, Christoph Bock, Marit Espe

**Affiliations:** 1 Institute of Marine Research, IMR, Bergen, Norway; 2 Faculty of Biosciences and Aquaculture, Nord University, Bodø, Norway; 3 Department of Microbiology, Oslo University Hospital, Oslo, Norway; 4 CeMM Research Center for Molecular Medicine of the Austrian Academy of Sciences, 1090 Vienna, Austria

## Abstract

Micronutrient status of parents can affect long term health of their progeny. Around 2 billion humans are affected by chronic micronutrient deficiency. In this study we use zebrafish as a model system to examine morphological, molecular and epigenetic changes in mature offspring of parents that experienced a one-carbon (1-C) micronutrient deficiency. Zebrafish were fed a diet sufficient, or marginally deficient in 1-C nutrients (folate, vitamin B12, vitamin B6, methionine, choline), and then mated. Offspring livers underwent histological examination, RNA sequencing and genome-wide DNA methylation analysis. Parental 1-C micronutrient deficiency resulted in increased lipid inclusion and we identified 686 differentially expressed genes in offspring liver, the majority of which were downregulated. Downregulated genes were enriched for functional categories related to sterol, steroid and lipid biosynthesis, as well as mitochondrial protein synthesis. Differential DNA methylation was found at 2869 CpG sites, enriched in promoter regions and permutation analyses confirmed the association with parental feed. Our data indicate that parental 1-C nutrient status can persist as locus specific DNA methylation marks in descendants and suggest an effect on lipid utilization and mitochondrial protein translation in F_1_ livers. This points toward parental micronutrients status as an important factor for offspring health and welfare.

## Introduction

Around 2 billion humans globally are affected by chronic micronutrient deficiency, collectively known as hidden hunger^1^. The term hidden hunger refers to a chronic deficiency of essential minerals or vitamins (micronutrients) resulting typically from an unbalanced diet that cannot meet nutritional requirements. Young children and reproductive aged females are most vulnerable to micronutrient deficiency due to the increased requirements for micronutrients during pre- and postnatal development. An improved attention to micronutrient composition has the potential to affect both the mother’s health and the child’s development^2^. The World Health Organization (WHO) is especially concerned about the nutritional supply of folate and vitamin B12 due to low blood concentrations across population groups in both developing and industrialized countries^1,3^. Reasons for the deficiency in these B-vitamins are complex and range from changes in food availability, food costs and eating habits, but reflect a general world-wide dietary intake shift away from ingredients rich in micronutrients towards feeds lower in micronutrients^2^.

Folate and vitamin B12 are fundamental components of the one carbon (1-C) metabolism, and together with the nutritional availability of methionine, choline and vitamin B6 these 1-C nutrients determine the cellular capacity for 1-C metabolism. The 1-C metabolism transfers a methyl group to homocysteine converting it first to methionine and then to S-adenosylmethionine^4^, which is consumed in subsequent methylation reactions including DNA methylation. Together these micronutrients are essential for several metabolic processes including energy metabolism, methylation and transamination reactions^5–7^. Deficiency in 1-C nutrients has been linked to severe developmental defects like neural tube defects, anemia and reduced cognitive function^8–10^.

The nutrients involved in the 1-C metabolism are potentially important in epigenetic gene regulatory mechanisms, as the 1-C metabolism provides the methyl group for DNA methylation. The overall epigenetic state of chromatin, including DNA methylation, have the potential to alter tissue specific gene expression^11^, and can be influenced by environmental factors including nutrients, toxicants and other stress factors^7,10,12^. The parental environment can directly modify DNA methylation in succeeding generations through consequences to the germ cells as well as through direct exposure of F_1_ somatic cells during embryo development. For female offspring, the in utero exposure for F_1_ can also affect the foetal germline (future F_2_), and thus give rise to affects in the F_2_ generation^13^. It has also been reported that either mutation of genes encoding enzymes^14^ or chemical interference of enzymes^15^ of the 1-C metabolism can affect the phenotype of the next generation.

Using zebrafish as a model, we have previously observed changes in embryonic gene expression in genes associated with several health related biological processes including inflammation, blood coagulation, and lipid transport due to a parental dietary 1-C nutrient deficiency^16^. Here, we test if this parental nutritional 1-C micronutrient deficiency has an impact on mature male offspring and found that these descendants had increased liver lipid inclusion and had lower expression of genes associated with lipid biosynthetic processes and mitochondrial protein synthesis. We also identified changes in DNA methylation at around 2869 sites. Extensive permutation analyses supports the robustness of a specific parental dietary induced effect on both mRNA expression patterns and DNA methylation. Together these observations argue for a physiological impact of the parental feed throughout the lifespan of the F1 generation.

## Results

### Parental feed did not affect body weight, but changed liver histology

**Figure 1:**
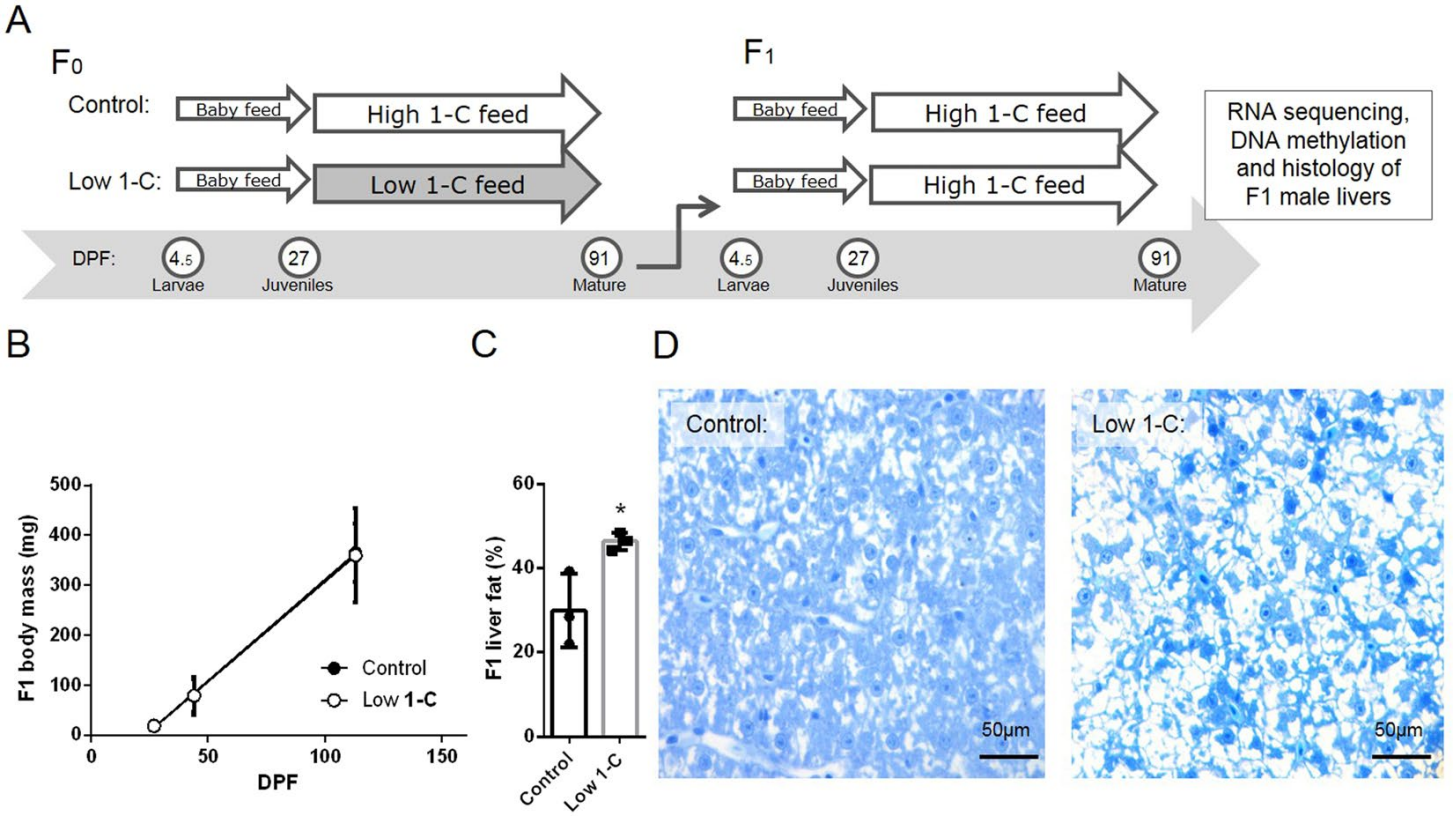
Experimental design, growth and F_1_ liver lipid accumulation. (**A**) After an initial start feed period (Gemma micro and *Artemia nauplii*) from larva (5 days post fertilization (DPF)) to juvenile (27 DPF) stage, F_0_ generation zebrafish were fed a control diet (high 1-C feed) or a low 1-C diet until mature stage. F_1_ progenies resulting from cross tank mating of F_0_ groups were raised on the control diet. Male F_1_ livers were sampled for transcriptome, DNA methylation and histology determination. (**B**) Offspring (F_1_) body mass fed only control diet. Data are presented as means ± SD of six independent feeding tanks from each feed group. (**C**) Liver fat (lipid and sterols) content of male offspring (F_1_) as assessed by image analysis of toluidine blue stained sections obtained from three males per feed group sampled from independent feeding tanks. The plot shows mean percentage ±SD, asterisk indicate significant difference between feed groups (p = 0.033). (**D**) Representative image showing toluidine blue stained sections of offspring (F_1_) livers from the control and the low 1-C group. White non-stained areas are lipids, sterols, solutes and the lumen of the blood vessels, bile canaliculi and sinusoids (see Methods).

Decreasing the level of 1-C nutrients in the parental feed (Experimental design: Fig. 1A, Feed composition: Table 1) did not affect the body weight in the F_1_ generation in either of the three stages measured (repeated measures ANOVA at 27, 44 and 113 days post fertilization (DPF), Tukey HSD test, p = 0.2, Fig. 1B). However, F_1_ livers from the low 1-C parental group had a noticeably paler red color than livers collected from the control group, and a histological analysis revealed a significant (t-test, p = 0.033) increase in liver lipid content in offspring from the low 1-C group (46.3% ± 1.2, n = 3) than in offspring from control fed F_0_ fish (29.9% ± 5.0, n = 3) (Fig. 1C,D).

**Table 1:** Diet formulation and chemical analysis.

	**Control feed**	**Low-1C feed**
***Diet formulation (g/kg)***		
Protein blend^1^	768,00	768,00
Oil blend^2^	80	80
Agar	1,00	1,00
Dextrin	46,44	50
Cellulose	19,25	19,25
Lecitin	20	20
Mineral mix^3^	50,00	50,00
Vitamin mix^4^	10	10
Astaxanthin	0.003	0.003
Sucrose	1	1
Tocopherol	0,75	0,75
Choline (50%)^5^	1	0
Vitamin B12 (0.1%)	1,00	0,00
Folate	0,011	0
Vitamin B6	0,02	0,00
Methionine	2,533	0
***Diet chemical analysis***		
Energy (MJ/kg)	21,10	21,10
Protein (g/kg)	490	500
Fat (g/kg)	12,7	12,2
Ash (g/kg)	68	68
Folate (mg/kg)	12,51	0,32
Vitamin B12 (mg/kg)	0,649	0,009
Vitamin B6 (mg/kg)	23,21	1,86
Methionine (g/kg)	9,41	5,79
Choline (g/kg)	1,903	1,254

^1^Protein blend: Fishmeal 5%, Krill meal 1%, Soya protein concentrate 6.2%, Corn 5%, Wheat 7.5%, Wheat gluten 13%, Pea protein 49.9%, Field peas 12.5%.

^2^Oil blend: Fish oil 1%, Rapeseed oil 60%, Leenseed oil 25%, Arachedonic acid oil 5%.

^3^Mineral mix: CaHPO_4_ × 2H_2_O 55.25%, CoCl_2_ × 6H_2_O 0.01%, CuSO_4_ × 5H_2_O 0.04%, K2SO_4_ 27.62%, KI 0.09%, MgSO_4_ × 7H_2_O 9.21%, MnSO_4_ × H_2_O 0.09%, NaCl 5.29%, Se-yeast 0.37%, ZnSO_4_ × 7H_2_O 0.92%, FeSO_4_ × 7H_2_O 1.10%.

^4^Vitamin mix: VitA 0.2%, VitD3 0.04%, VitE (50% stock (S)) 2%, VitK (50% S) 0.1%, VitC (35% S) 3.5%, Ascorbic acid 10%, Thiamin 0.15%, Riboflavin (80% S), 0.19%, Niacin 2,00%, Inositol 4%, CA-pantothenate 0.6%, Biotin (2% S) 0.5%. Choline 10,00% in control, 0% in low 1-C. Protein blend (carrier) 66.72% in control, 76.72% in low 1-C.

^5^Choline: Added as 10% of the vitamin mix of the control feed.

### Parental (F_0_) diet affects gene expression in F_1_ livers

**Figure 2:**
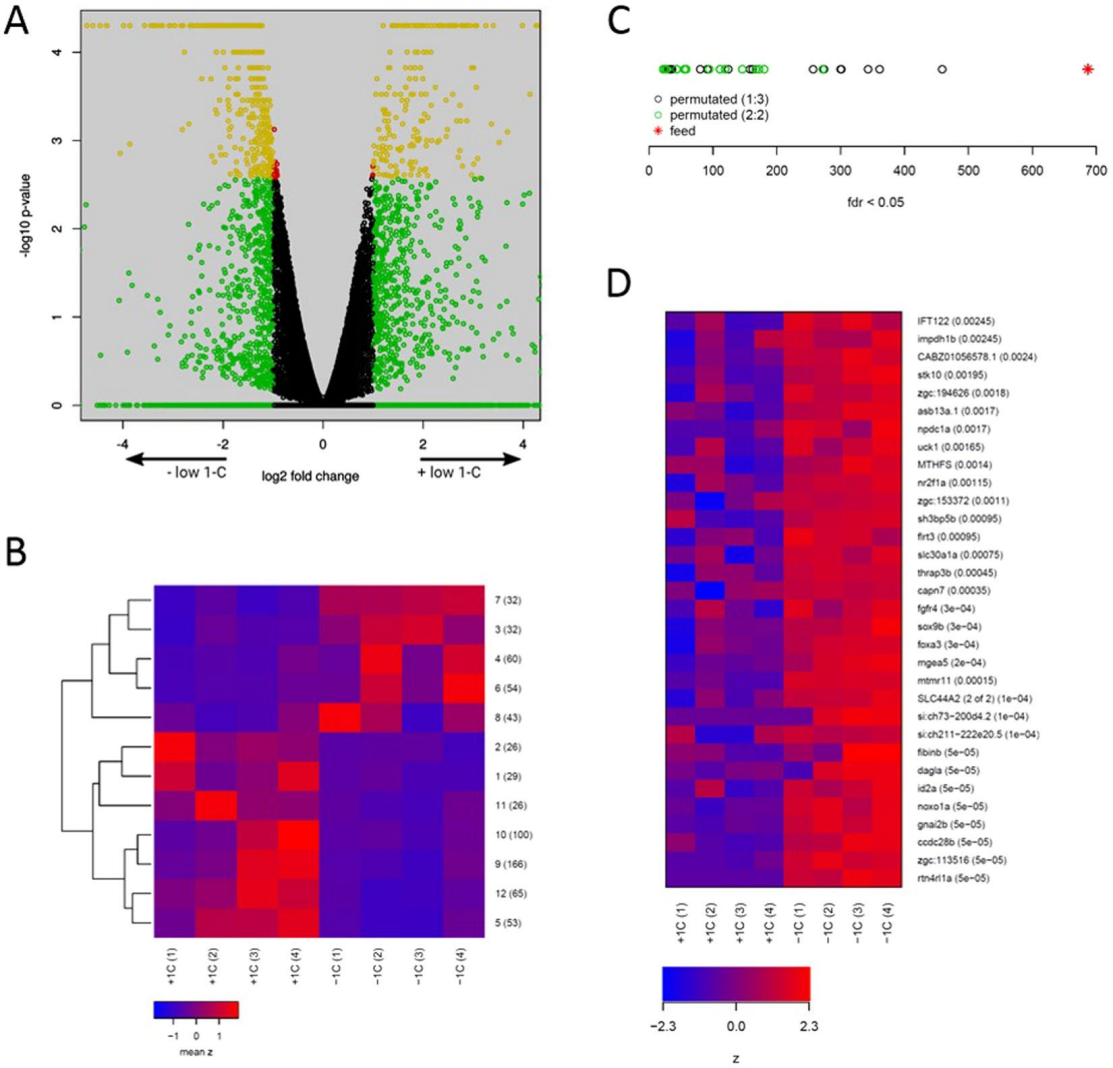
F_1_ male liver gene expression is affected by parental 1-C diet. (**A**) Differential expression in low 1-C compared to control F_1_ male livers as reported by Cuffdiff. x-axis: log2 fold change (low 1-C/control) of expression values, y-axis: −log10 p-values. Green indicates a fold change of more than 2, red a q-value of less than 0.05, yellow both and black neither of these. (**B**) Mean expression levels of clusters of differentially expressed genes. Genes identified as differentially expressed by Cuffdiff were divided into 12 clusters by k-means clustering of their gene expression levels across the replicate series. Each row of the heat map displays the cluster mean expression scaled by row from control (+1C) and low 1-C (−1C) F_1_ male livers. The cluster number (1–12) and number of differentially expressed genes are indicated at each row. (**C**) Permutation analysis. Numbers of genes identified as differentially expressed by Cuffdiff (fdr <0.05) for all possible sample permutations. Red asterisk indicates the number for the correct sample permutation that separates the two feed groups. Green points indicates sample permutations where each set of samples contained 2 samples from each feed group, black points where each set contained 3 samples from the same feed group. (**D**) Expression of individual genes in the cluster with the least within-group variance (cluster 7, 32 genes). Expression levels are indicated by color, with blue to red indicating min to max expression levels for each gene. P-values for differential expression (from Cuffdiff) are indicated next to the gene names.

We performed sequencing of mRNA molecules obtained from male F_1_ livers from offspring derived from the two feed groups (control and low 1-C) in order to determine whether parental diet can affect gene expression in adult offspring. Males were studied to avoid fluctuations in liver physiology caused by the female fluxes in vitellogenesis and oogenesis related to spawning in fish. We used the Cuffdiff program to identify differentially expressed genes (DEGs) and found 686 DEGs with a reported false discovery rate (FDR) of less than 0.05 (Table S1). Out of these, 663 genes had a fold change greater than 2 and the majority (467) had a lower expression in F_1_ male livers of the low 1-C group (Fig. 2A).

Cuffdiff uses sampling statistics based on the numbers of reads mapped to genes. The reported p-values reflect the probability of the measurements in the two feed groups being drawn from a single distribution of values, but do not address the probability of there being an unrelated difference between the groups caused by an uknown factor (eg. stress or immune response). To make sure that the differential expression observed was not caused by an unknown random factor we re-ran Cuffdiff on the full set of possible sample permutations (re-orderings). These permutations identified between 22 and 459 genes as differentially expressed (mean 143) within these permutations (Fig. 2C), with all permutations that gave more than 300 DEGs having 3 samples from the same feed group in each sample set. This argues strongly that the genes identified by Cuffdiff, although not uniformly expressed within each group, are nevertheless caused by the difference in the parental feed (Fig. 2C).

We used orthology mapping from Ensembl to map DEGs to human orthologues and submitted these to the DAVID bioinformatics resource^17^ to determine if the differential expression was likely to be due to a shift in liver physiology. The DEGs were enriched for genes involved in lipid biosynthesis, especially sterol and steroid related processes. We also observed enrichment for molecular functions related to protein folding (chaperone activity) and redox regulation. The protein localizations (cellular components) of DEGs were enriched for the endoplasmic reticulum (ER) and mitochondria consistent with lipid biosynthetic process, protein folding and redox regulation functions (Table 2 (complete list in Table S3)). To ensure that the observed enrichments were not due to the selection of genes expressed in the liver we also performed enrichment analyses using genes sampled to have similar distributions of expression levels as those of the differentially expressed genes (Fig. S5). These confirmed the specificity of the enrichments. RNA sequencing verification revealed equivalent expression pattern for selected genes using RT-qPCR (Fig. S4).

**Table 2:** Gene ontology. Gene ontology analyses for biological processes, molecular function and cellular components for differentially expressed genes in low 1-C F_1_ male livers compared to control F_1_ male livers. In each ontology, the two most enriched groups of significant GO terms, with the number of enriched genes, p-value and Benjamini are listed.

Biological process		Genes	P-value	Benjamini
**1**	**GO:0016126: sterol biosynthetic process**	18	5.19E-17	1.10E-13
	**Official gene symbols:** *ebp*, *sqlea*, *mvda, sigmar1, nsdhl, msmo1, c14orf1, dhcr7, mvk, fdps, hmgcra, cyb5r2, dhcr24, hmgcs1, cyp51, lss, sc5d, idi1*			
**2**	**GO:0006694: steroid biosynthetic process**	23	9.79E-15	1.03E-11
	**Official gene symbols:** *rdh8a, ebp, sqlea, hsd17b12b, mvda, nsdhl, sigmar1, msmo1, hsd11b2, c14orf1, dhcr7, mvk, fdps, hmgcra, cyb5r2, hsd17b7, hsd17b3, dhcr24, hmgcs1, cyp51, lss, sc5d, idi1*			
**Molecular function**				
**1**	**GO:0051082: unfolded protein binding**	16	2.85E-6	2.11E-3
	**Official gene symbols:** *pfdn2, hspa5, hsp90b1, dnajb11, hspd1, lrpap1, sil1, trap1, hspe1, ruvbl2, grpel1, canx, tubb4b, hspa9, uggt1, ptges3b*			
**2**	**GO:0016860: intramolecular oxidoreductase activity**	9	1.83E-5	6.77E-3
	**Official gene symbols:** *pdia4, pdia2, ddt, pdia6, ebpl, ebp, sigmar1, ptges3b, idi1*			
**Cellular component**				
**1**	**GO:0005783: endoplasmic reticulum**	79	2.26E-14	7.78E-12
	**Official gene symbols:** *scdb, sel1l, cav1, ebp, zmpste24, ebpl, sqlea, ostc, alg8, pld1a, tmed9, hyou1, lrpap1, sil1, prkcsh, hsd11b2, rrbp1b, dhcr7, c14orf1, nucb2a, hmgcra, mgst2, tnrc5, cyp51, dpm3, uggt1, man1a1, pdia2, lman2L, pdia6, hsp90b1, lrrc59, sdf2l1, cd74a, creld2, ncl1, nsdhl, srpr, ergic1, sec11a, rcn3, tmed5, cyp3a65, rsad2, sc5d, insb, hspa5, hsd17b12b, pdia4, cyp7a1a, canx, sec61al2, lmf2a, mgst1.1, sec31a, slc35b1, mpdu1a, stt3B, mogat3a, atp2a2a, dnajb11, mesdc2, fkbp14, mogs, nomo, mlec, elovl6, tram1, edem1, hm13, sigmar1, dnajc3a, msmo1, hsd17b10, hsd17b7, dhcr24, ubqln4, sgk1, alg12*			
**2**	**GO:0005739: mitochondrion**	76	2.43E-10	2.79E-8
	**Official gene symbols:** *cav1, agpat5, mrpl14, acsbg2, si:ch73-209e20.3, mcl1a, mrpl43, trap1, si:ch211-149b19.3, sh3bp5b, slc25a32a, tufm, dlat, chchd2, oxr1, sdhb, pdk2a, aass, lrrc59, abcb8, mrpl41, hspd1, mrpl35, slc25a48, hspe1, txn2, cox10, samm50l, uqcr10, slc25a43, mrpl18, timm10, mrps18c, grpel1, timm44, timm8b, endog, mrpl19, qars, mrps16, acp6, cyp24a1, idh3a, dnajc11, tomm40, ptcd3, hcls1, fam136a, timm13, mrpl1, mgst1.1, abce1, phb2a, timm22, aco2, chchd4, vars, agk, lap3, cyb5b, timm17a, elovl6, glula, tmem186, hccsa.1, cox17, stoml2, hsd17b10, slc25a39, dap3, mtif2, amt, mrps31, phb, hspa9, lonp1*			

### Parental F_0_ diet upregulated DEGs of diverse diseases and redox regulation

To determine if there are distinct expression patterns that are associated with specific biological functions we used K-means clustering to divide the DEGs into 12 clusters (Fig. 2B). Five of these clusters were predominantly upregulated in the low 1-C group. We submitted up- and downregulated gene clusters separately to DAVID to determine whether distinct biological processes were affected in opposite directions. For the upregulated gene set the most enriched functions were related to immune processes and chemokine signaling, but the numbers of genes annotated to these functions were low (Table S5). From the 12 DEG clusters, one cluster (no. 7, contains 32 genes) of genes were homogeneously upregulated across all replicate F_1_ low 1-C livers (Fig. 2D). This cluster contains genes of diverse functions that are also associated with different diseases (*id2a*, *fgfr4*, *thrap3b, stk10, rtn4rl1a, ccdc28b, sh3bp5b, sox9b, flrt3, mgea5, gnai2b* (ref: www.genecards.org)), redox regulation (*noxo1a*), 1-C metabolism (*mthfs*) and choline transport (*slc44a2*), but was not significantly enriched for any specific biological processes.

### Parental F_0_ diet downregulate sterol, steroid and lipid biosynthetic processes

**Figure 3:**
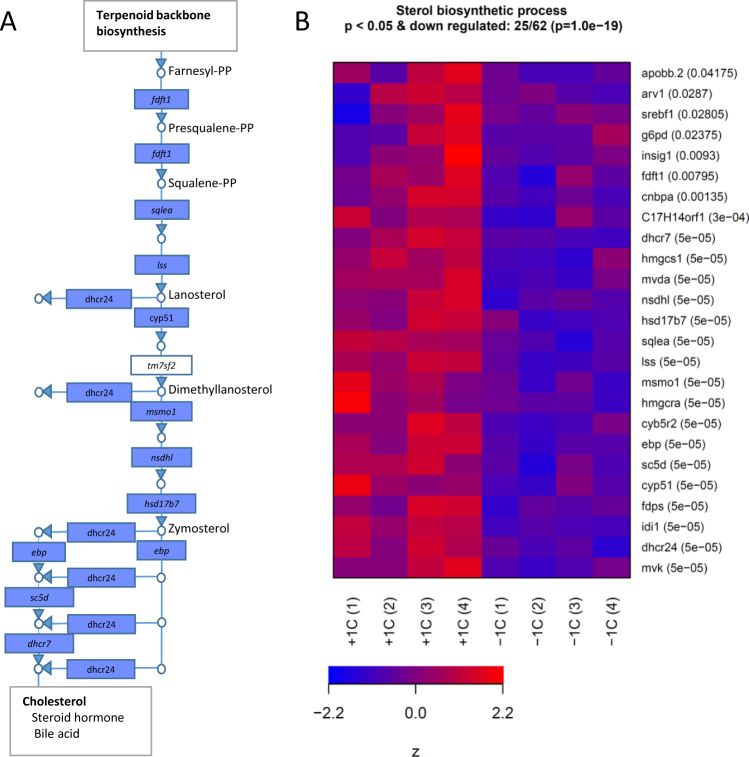
Parental diet downregulates genes related cholesterol synthesis in F_1_ male livers. (**A**) Differentially expressed genes in the cholesterol biosynthetic process. Blue boxes indicate significant downregulation in the low 1-C group. Only one gene in this pathway (tm7sf2, white box) was not downregulated. (**B**) Heat map of mRNA expression levels of significantly affected genes related to sterol biosynthetic processes from control (+1C) and low 1-C (−1C) F_1_ male livers. The low 1-C F_1_ male livers have lower expression levels than control F_1_ male livers. Expression levels are indicated by color, with blue to red indicating min to max expression. P-values are indicated next to the gene names. The probability of observing the given proportion of downregulated genes with p < 0.05 was determined using the hypergeometric distribution (p = 1e − 19).

The downregulated genes were significantly enriched for biological processes related to sterol, steroid and lipid biosynthesis (Table 2) and KEGG pathways like the steroid and terpenoid biosynthesis (cholesterol synthesis, Fig. 3A, and Table S5). To confirm whether there was an overall change in sterol biosynthetic process we inspected the expression patterns of all genes annotated as having lipid biosynthetic function. Close to half of these genes were identified as differentially expressed by Cuffdiff (p < 0.05, 25/62), and all of these were downregulated in the low 1-C group. This confirms that there was an overall directed change in metabolism associated with the parental feed (Fig. 3B).

### Parental F_0_ diet affects genes involved in mitochondrial protein synthesis

Our initial DAVID analysis found an enrichment (cellular components, Table 2) for genes encoding proteins that locate to the ER and mitochondria. Close to half of the genes downregulated in the low 1-C group were either annotated as being located to the endoplasmic reticulum (105/467) or the mitochondrion (107/467). Of the ER associated genes, more than half of the genes were annotated as having functions involved in protein folding, sterol biosynthesis or lipid metabolism, which is consistent with the functional enrichment reported by DAVID (Fig. S2, Table 2).

**Figure 4:**
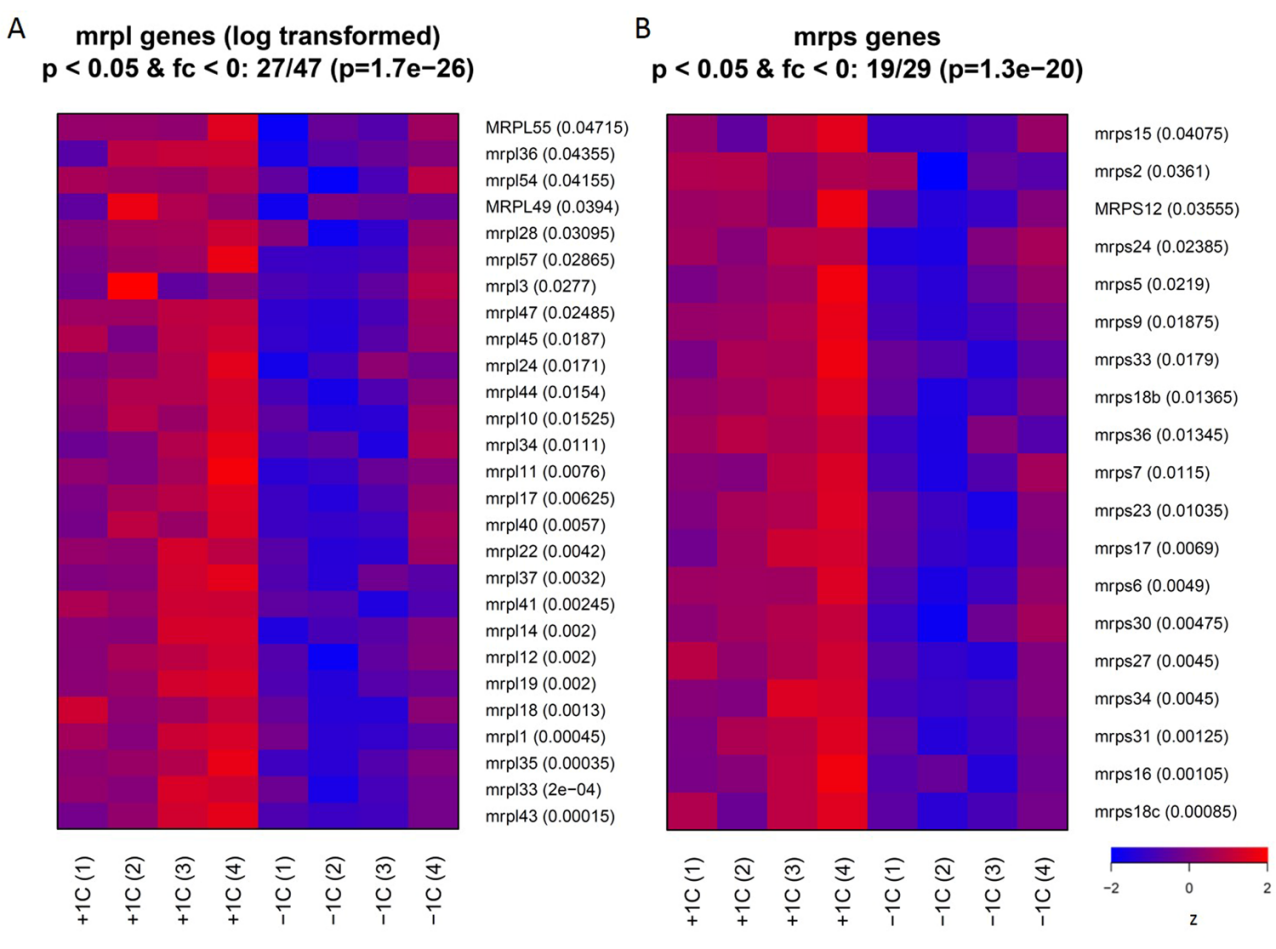
Parental diet affects the regulation of mitochondrial ribosomal proteins. Heat map of mRNA expression levels of significantly affected mitochondrial ribosomal proteins from small (**A** *mrps*) and large (**B** *mrpl*) mitoribosome units in control (+1C) and low 1-C (−1C) F_1_ male livers. Expression levels are indicated by color, with blue to red indicating min to max expression level for each gene, respectively. p-values reported by Cuffdiff are given next to the gene names. The probabilities (1.3e − 20 and 1.7e − 26) of observing the given proportion of downregulated genes with p < 0.05 was determined using the hypergeometric distribution.

**Figure 5:**
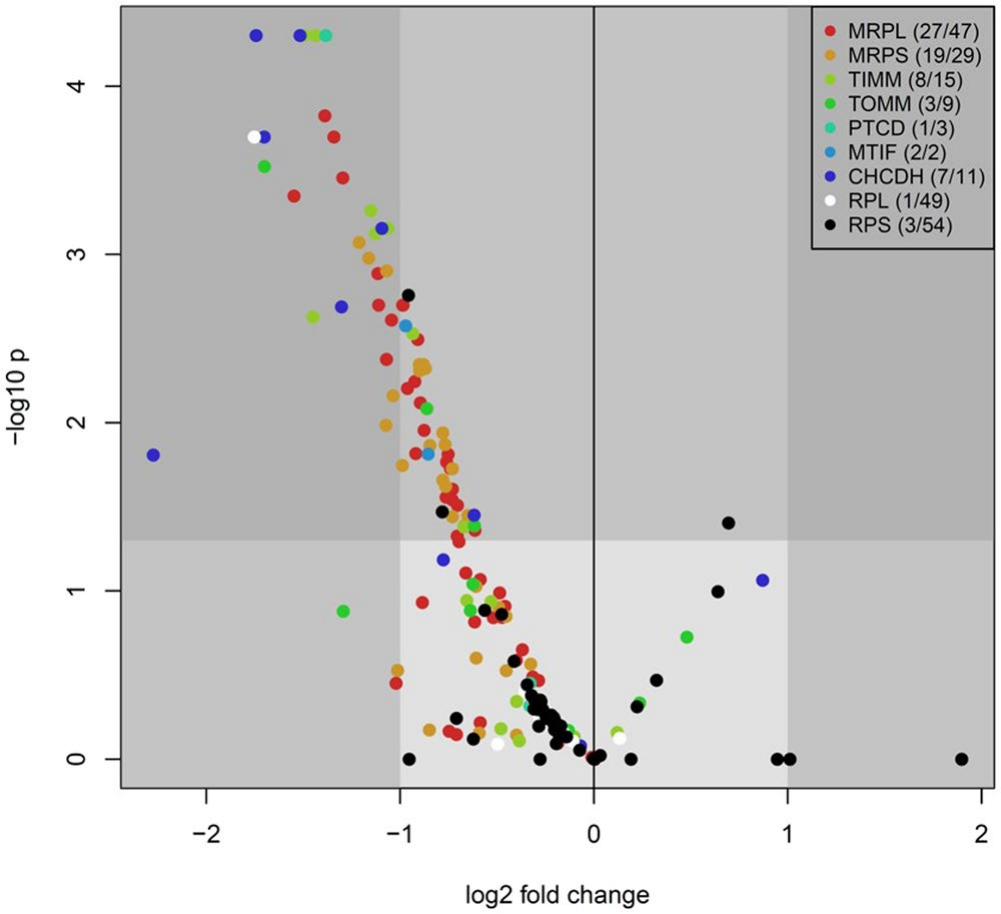
Parental diet affects the regulation of mitochondrial proteins. Differential gene expression pattern of mitochondrial proteins in low 1-C F_1_ male livers compared to control F_1_ male livers. Differential expression as reported by Cuffdiff, x-axis: log 2 fold change (low 1-C/control) of expression values, y-axis: −log10 p-values. Protein groups are indicated by color codes: *mrpl* (mitochondrial ribosomal proteins large subunit), *mrps* (mitochondrial ribosomal proteins small subunit), *timm* (translocase of inner mitochondrial membrane), tomm (translocase of outer mitochondrial membrane), *ptcd* (pentatricopeptide repeat domain), *mtif* (mitochondrial translation initiation factor), *chcdh* (coiled-coil-helix-coiled-coil-helix domain containing). White and black points indicate the non-mitochondrial *rpl* (cytoplasmic ribosomal proteins large subunit) and *rps* (cytoplasmic ribosomal proteins small subunit) proteins respectively included for contrast. Dark grey shading indicates p < 0.05 and a fold change greater than 2.

In contrast, genes associated with the mitochondrial compartment contained a number of mitochondrial ribosomal proteins (MRPL and MRPS, mitochondrial ribosomal proteins large and small subunit, respectively, Fig. 4A,B). We inspected the expression patterns of the full set of MRPL and MRPS genes and found that more than half were expressed at lower levels in the low 1-C group using a p-value cut-off of 0.05. None of the MRPL or MRPS were expressed at a higher level in the low 1-C group (Fig. 5) and the majority had a similar expression pattern across the samples (Fig. 4A,B). To determine whether this was indicative of a more general pattern, we identified other genes related to mitochondrial translation and inspected their expression patterns as above (Fig. 5). All of the genes which were differentially expressed (MRPL: 27/47, MRPS: 19/29, TIMM: 8/15, TOMM: 3/9, MTIF: 2/2, PTCD: 1/3 and CHCDH: 7/11) between the two groups (p < 0.05) were expressed at lower levels in the low 1-C samples (Fig. 5, Fig. S3). To confirm that this was not due to a general decrease in protein synthesis we also looked at cytoplasmic ribosomal proteins (RPL: 1/49 and RPS: 3/54) and these did not show any similar pattern confirming that the effect is specific to mitochondrial translation (Fig. 5).

### Parental feed affects locus specific DNA methylation changes in F_1_ livers

To investigate if the parental feed changed the offspring DNA methylation pattern we performed reduced representation bisulfite sequencing of six livers from each sample group. The bisulfite conversion rates (Fig. S6) and CpG motif counts (Fig. S7) were close to equal for all samples, estimated by the biseqMethCalling.py script as described^18^. Most CpG positions in vertebrate genomes are fully methylated^19^, with demethylation associated with CpG islands and active promoters^20^. Our data set follows this pattern, with the majority of CpG sites being either fully methylated (80% more than 80% methylated) or almost completely demethylated (6% less than 10% methylated) (Fig. S8).

We observed a decrease in methylation at transcriptional start sites (TSSs) (Fig. S9A). This decrease coincided with a peak in coverage, presumably due to the presence of CpG islands overlapping with TSSs, as expected due to the nature of RRBS libraries (Fig. S7B). DNA methylation of regions upstream of TSSs was generally associated with decreased expression levels (Fig. S9C and D).

**Figure 6:**
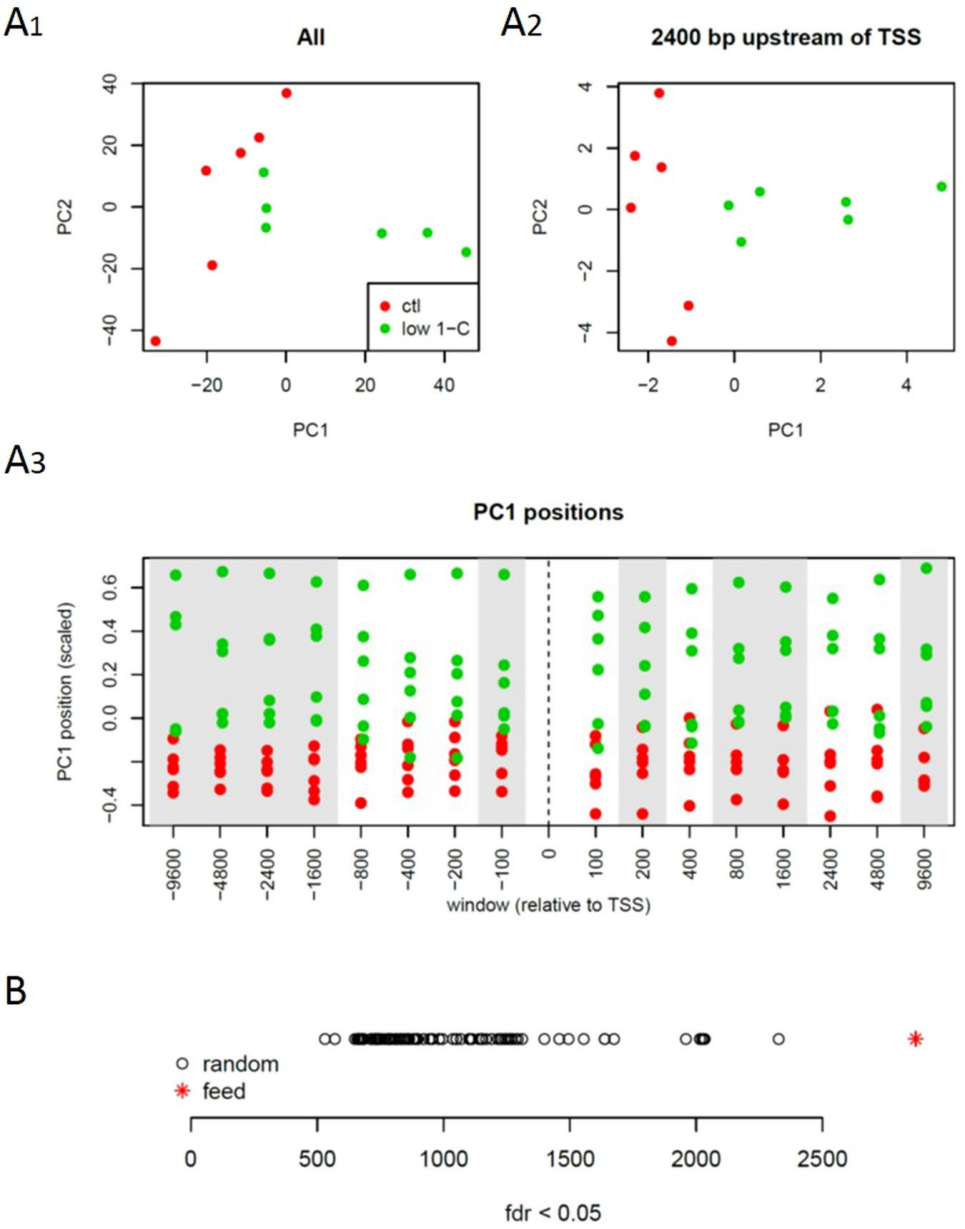
Differential methylation due to parental feed. (**A**) Principal components analyses (PCA) for methylation levels at (**A**_**1**_) all CpG positions with a minimum coverage of 10 reads, (**A**_**2**_) mean methylation levels for regions lying up to 2400 bases upstream of TSSs and (**A**_**3**_) the positions in the first components for PCAs carried out on mean methylation levels of nested regions upstream (negative numbers) and downstream of TSSs. (**B**) The numbers of significantly (false discovery rate < 0.05) differentially methylated sites identified by DSS for 100 random sample permutations (black circles) and classified by feed (red asterisk).

We found that parental feed had an overall effect on DNA methylation by performing a principal components analysis on all CpG sites where the minimal coverage was 10 reads or more. Using the full set of individual CpGs we did not see a clear segregation of two feed groups (Fig. 6A_1_). However, we found a clear pattern using PCAs based on the mean methylation levels of a series of nested regions upstream and downstream of 57,881 TSSs obtained from Ensembl (non-nested regions PCA plot confirms the site specificity of the parental feed, Fig. S10). In five of the eight upstream, and four of the downstream windows we observed a complete separation of the two sample classes in the first dimension (Fig. 6A_2–3_). This separation was obvious for windows from 1600 to 4800 bases upstream of TSSs. Although there was within-class variance for these positions, in all cases the primary component co-segregated with the feed-group as assessed by either t-tests or Mann-Whitney U tests (giving p-values ranging from 0.03 to 0.004) and which confirms that the locus specific DNA methylation pattern observed in the F_1_ livers does indeed reflect parental diet (Fig. 6B).

### Identification of differentially methylated loci

To identify differentially methylated sites we made use of the DSS Bioconductor package^21^. DSS models the sequence count data as Beta-binomial distributions where biological variations are captured by dispersion parameters estimated through a shrinkage estimator^21^. This identified a total of 2869 CpG sites with an estimated FDR less than 0.05 from a total of 2.7 million CpG sites considered (Table S6). The p-values obtained by DSS depend on a number of assumptions and to assess the biological FDR we performed the same test for 100 random sample permutations. Most of these analyses reported around a 1000 (mean 1028) sites with FDR values less than 0.05 (Fig. S11) suggesting that 1800 locus specific sites identified by DSS are due to the parental feed group. To assess these results, we quantified the FDR from the permutation data and calculated the ratio of the rate of discoveries for random sample permutations to the correct set. These FDRs were higher than those reported by DSS. However, the number of sites with a FDR less than 0.5 was 3222, suggesting again 1500 sites with feed related differential methylation (Table S7) which is of a similar order of magnitude reported directly by DSS.

### Genome locations of differential methylation

**Figure 7:**
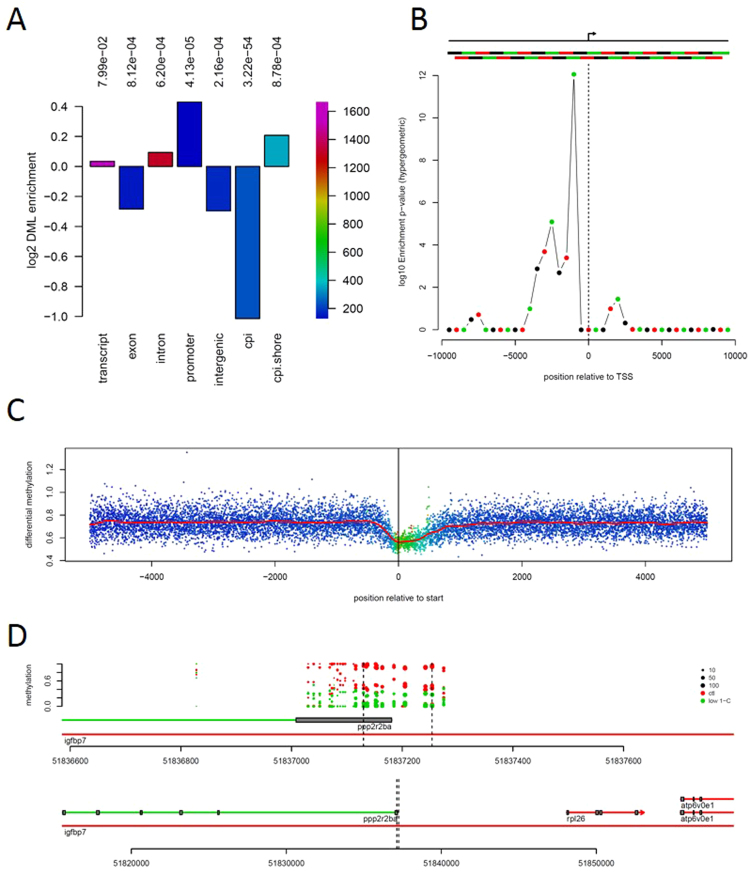
Non-random location of differential methylation. (**A**) Enrichment ratios (log2 transformed) for differential methylation loci (DML) within the indicated gene and genomic features. The associated p-values for enrichment or depletion indicated above the plot were determined by Fischer’s exact test (hypergeometric distribution). The bar colors map to the total number of DML within the respective regions (scale bar on right axis). (**B**) Enrichment probabilities (hypergeometric, adjusted for multiple testing using Holm’s method and −log10 transformed) of overlapping 1000 bp windows either side of TSSs. Upper section indicates the TSS (arrow) and the regions used in the calculation. p-values indicate the probability of observing the observed number or larger of DMLs (p < 0.05) within each window given the total number of measurements falling within the windows and the total number of DMLs. (**C**) Mean differential methylation around TSSs. The mean of absolute DSS test statistics observed around TSSs. Each point indicates the mean value at the specific distance. The red line is a kernel smoothed mean for the points. Colors of points indicates the sampling density (i.e. the number of measurements contributing to each mean value), with values mapping from low to high from blue to purple via cyan, green, yellow and red. (**D**) Differential methylation around the first exon of the gene *ppp2r2ba* (protein phosphatase 2, regulatory subunit B, beta a), overlapping an intron of *igfbp7* (insulin-like growth factor binding protein 7) gene located on chromosome 14. Each point indicates mean methylation measurement in each of the six samples per group, red and green points indicate DNA methylation for the control and low 1-C samples, respectively. The point size maps to the number of reads used to derive the methylation level estimate. The *ppp2r2ba* gene is transcribed from right to left in the plot. Lower panel gives an expanded view of the genomic locus containing *ppp2r2ba*.

We determined distances between the measured CpG positions and genome features (transcripts, exons, introns, promoter, CpG islands and CpG island shores) to determine whether the differential methylation was randomly distributed across genomic regions covered by RRBS or enriched in specific locations (Fig. 7A and Table S7). The strongest effect is seen for CpG islands which contain about half the expected number of differentially methylated loci (DML). Differential methylation was strongly enriched in promoter regions (5000 bp upstream of transcription start site (TSS)) and less in CpG island shores (here defined as outside of, but within 5000 bp of a CpG island) (Fig. 7A). Note that the enrichment observed for CpG island shores is not markedly different from that observed for all CpG sites outside of CpG islands. Conversely, differential methylation was depleted from CpG islands (strongly), exons and intergenic regions (>50000 bp from a gene) (Fig. 7A).

Tissue specific differential methylation has previously been reported to be enriched in CpG islands shores rather than in CpG islands themselves^22^; here we do not see a strong enrichment for such shores, but do see an enrichment around promoters. This difference may arise from the fact that we are looking at a different system (fish), or a different type of difference (within a single tissue type).

Enrichment for differential methylation was strongest within one kb upstream of TSSs (Fig. 7B). Differential methylation decreases immediately upstream, and stays low for approximately 200 bp downstream of TSSs (Fig. 7C). These observations confirm that the feed associated differential methylation is associated with gene features and is not caused by random variance.

### Differentially methylated regions and genes

We associated these sites with genes by locating the most proximal genes for each site and tried to determine if such genes were enriched for functional categories or for differential expression (Differentially methylated regions (DMR): Fig. S12 and differentially methylated loci (DML): Fig. S13). However, we were unable to find a strong enrichment for functional categories.

We also searched for regions of differential methylation (see methods), reasoning that such regions might be more informative than individual sites. However, we were also unable to find any robust enrichment for functional categories or differential expression for genes adjacent to these regions. Instead we manually inspected plots of genomic regions containing DMRs or DMLs to search for differential methylation with potential for affecting the expression of adjacent genes. This identified twelve genes lying within 40,000 base pairs (bp) of the DMRs or DMLs and which were also differentially expressed (Table S8). Further, we identified four genes where a decreased gene expression coincided with increased methylation; *sdhb*, *psmc6*, *thop1*, *mtmr4* (Table S8). Interestingly, both *sdhb* and *thop1* are localized as part of the mitochondrial respiratory chain complex II and in the mitochondrial intermembrane space, respectively. The weak correlation between gene expression and differential DNA methylation may be due to the difficulty of identifying which genes are regulated by specific differentially methylated sites or because most of the methylated sites are not regulating genes expressed in the liver. Nevertheless, several genes had a clear separation of DNA methylation levels between the sample groups at multiple CpGs in the promoter and first exon (*ppp2r2ba* (Fig. 7D), *tpm4b* and *fibina* (both Fig. S12) and in the second exon (*tpst2* and *abhd8a* (Fig. S12)). Our analysis confirm the presence of both hypo- and hypermethylated genomic loci (Fig. S13) and regions (Fig. S12) resulting from the parental feed.

## Discussion

Our observations indicate that a parental 1-C micronutrient deficiency, using zebrafish as a nutritional model, can lead to modifications in locus specific DNA methylation, gene expression and lipid accumulation in adult F_1_ male offspring without having a noticeable effect on growth. This is in contrast to the parental generation where the micronutrient deficiency resulted in both reduced growth and fecundity^16^. This shows that the parental micronutrient status can have long-lasting effects in vertebrate offspring that are not easily visible, and suggests that changes in DNA methylation should be considered as one of the effects of ‘hidden hunger’^1,2^.

Studies on the effect of 1-C nutrients have led to somewhat contradictory observations, probably due to differences in experimental details such as the duration of exposure, variation in feed raw materials and compensatory mechanisms where other nutritional components have been diverted to the 1-C cycle. For example, when vitamin B12 availability is limited, folate cannot re-methylate homocysteine to methionine, leading to a shift in the S-adenosylmethionine (SAM) to S-adenosylhomocysteine (SAH) ratio, however if there is an excess of choline, then this can be utilized to sustain the SAM/SAH ratio^23^. The SAM/SAH ratio determines the methylation potential (including DNA methylation) in cells, and nutrients involved in the 1-C cycle are vital for both the general energy metabolism and methylation and transamination reactions. A dysregulation of the 1-C cycle either through an inadequate nutrient supply, or the depletion or mutation of enzymes catalyzing the 1-C metabolism has been linked to developmental deformities and metabolic diseases^5–7,24–26^. In addition, a vitamin B12 deficiency has been shown to directly induce a decrease in intestinal DNA methylation in rats^27^, and a maternal vitamin B12 deficiency reduced the birth weight of offspring that eventually became heavier than control rats 12 months after birth^28^. For zebrafish, a 1-C micronutrient deficiency resulted in both reduced growth and fecundity for the parental generation but did not lead to deformities in F_1_ suggesting that the parental generation received adequate levels to avoid direct developmental defects^16^.

Early embryonic development is particularly sensitive to its environment because of the rapid growth and cellular differentiation (including replication of DNA where folate is needed for thymidine synthesis)^4,29,30^. These developmental processes are accompanied by major epigenetic events including the maternal-to-zygotic transition involving zygotic genome activation (ZGA) and an initial global demethylation followed by remethylation as the cells differentiate^31^. Compared to the mammalian ZGA which occurs during early cleavage stages^11^, zebrafish has a “delayed” major wave of ZGA after the pluripotent blastula stage^32^. These differences between mammals which have early segregation of extra and intra-embryonic linages, and zebrafish which maintains totipotent cells throughout the blastula stage provides opportunity for differences also at the DNA methylation level. Zebrafish lack Dnmt3L suggesting that they do not have parentally specific imprinting, and it has also been suggested that the paternal DNA methylation pattern serves as a template for embryonic DNA methylation in order to establish the totipotency needed for ZGA^33,34^. Embryo development relies on the availability of 1-C donors in the blastomeres and later the yolk (or placental circulation in mammals) for appropriate cellular growth and differentiation.

Embryo development relies on the availability of 1-C donors in the blastomeres and later the yolk (or placental circulation in mammals) for appropriate cellular growth. A methyl-deficient diet during the peri-conception period for rodents changes the homocysteine levels in the mothers, but is rapidly restored when adequate nutrition were offered, however, depending on time of conception, and time for malnutrition, these nutritional challenges affects the offspring metabolism^35^. In fish these early embryonic stages express and regulate almost all of the enzymes that participate in 1-C metabolism^16,36,37^, and using zebrafish, we have previously shown that a marginal parental 1-C micronutrient deficiency led to an upregulation of genes associated with immune, lipid and apolipoprotein functions already at the sensitive embryo stage of F_1_ generation^16^. Here we present results that indicate that the effects of the parental diet persist in mature F_1_, and observed a general shift with decreased expression of genes related to lipid and steroid biosynthesis in the fatty livers in offspring of the 1-C deficient parents.

Hepatic steatosis is commonly observed as a direct effect (within generation) when the methylation capacity is compromised^38,39^, and our observations suggest that effects of the parental micronutrient status can cross to the descendant generation leading to changes in histology, gene expression and DNA methylation pattern. Other studies using rodents have shown a dietary effect of parental feed on glucose metabolism^35,40^, gene expression^41,42^ and DNA methylation^42^. Similar to the effects we report here, these effects observed at mature stages may result from developmental effects of nutrient availability (ancestral environment) or from changes in DNA methylation in the parental germ cells^12^. Our observations cannot distinguish between these alternatives but suggest that zebrafish may be a suitable nutritional model to investigate how nutrient composition affect the vertebrate offspring epigenetic regulation.

The permutation analyses we carried out argue that the differences in DNA methylation we observed in mature F1 livers are caused by the parental dietary deficiency. However, only few genes revealed changes in both DNA methylation and gene expression in the liver. Previous studies on DNA methylation changes during cellular linage commitment have reported a similar low correlation between DNA methylation and gene expression, but those genes which correlated were strong candidates for biological function and cellular identity^43^. Possible explanations for this include a specific tissue and/or developmental stage dependent effect of DNA methylation that control gene expression. Interestingly, a few genes associated with mitochondria were hypermethylated and downregulated due to parental 1-C nutrient composition in the liver.

We identified DMRs within a number of genes which were either not expressed or not differentially expressed in the liver. These include genes as *ppp2r2ba*, which encodes a protein phosphatase, implicated in the negative control of cell growth and division. Defects in *ppp2r2ba* have been linked to degeneration of parts of the central nervous system including the cerebellum^44,45^. It is possible that the differences in DNA methylation were established early in development (including from the germ-line) and these may have regulatory effects in other tissues. This would be reasonable in the case of *ppp2r2ba*, which is known to have functions in the CNS rather than the liver.

We observed decreased expression levels of genes encoding proteins involved in cholesterol storage, synthesis, and lipid/sterol sensing. This may be due to direct effects of altered gene expression, or may alternatively result from feedback from internal lipid and sterol levels as this pathway is known to be regulated by tissue sterol levels^46^. The reduction, at the mRNA level, of key regulators of cholesterol synthesis is consistent with our observation of a general decrease in expression of genes associated with steroid biosynthesis. We also found lower levels of several upstream regulators of cholesterol synthesis, including the sterol regulatory element-binding protein transcription factor 1 (*srebf1*). Sterols inhibit the cleavage of *srebf1* that is necessary for the translocation to the nucleus and as such this is the main sensor and regulator of cellular cholesterol levels. Srebf1 acts as a transcription factor for HMG-CoA reductase (encoded by the *hmgcr* gene) which regulates an early rate limiting step of cholesterol synthesis. Both of these are located to the ER membrane. High levels of intermediate lipid species in cholesterol synthesis (e.g. lanosterol) leads to the binding of HMG-CoA reductase to insig (encoded by *insig1* gene) resulting in the degradation of the HMG-CoA reductase protein by ubiqutination^46^. Hence the downregulation of genes linked to lipid utilization appears to be consistent with the observed high lipid phenotype in the F_1_ livers and with recent work that has linked the 1-C metabolism with fatty acid regulation^38,39,47–49^.

We found an enrichment for genes encoding mitochondrial proteins in the set of downregulated genes. An inspection of these genes revealed that they were predominantly associated with mitochondrial translation and transport across the mitochondrial membranes. Indeed, more than half of all mitochondrial ribosomal protein genes and a number of genes coding for proteins belonging to the mitochondrial ribosomal machinery were downregulated (Figs 4 and 5), suggesting an overall decrease in mitochondrial transcription in livers of offspring of 1-C deficient parents. Mitochondria are the site of oxidative phosphorylation which is necessary to utilize the energy content of lipid molecules. An overall decrease in mitochondrial mRNAs may therefore reflect a decrease in the rate of lipid utilization and this may be the primary cause of the increased lipid content in offspring livers of 1-C deficient parents. Further studies related to lipid utilization and mitochondrial activity would elucidate how nutritional alteration of the 1C-metabolism can alter the mitochondrial functionality in livers through generations. This adds parental micronutrient status as a new level to mitochondrial dysfunction^50^, as the assembly of the mitochondrial ribosome (mitoribosome), oxidative phosphorylation and mutations in related genes have been associated with human disease^51^.

The lipid accumulation in F_1_ livers of the low 1-C group was surprising, and together with the changes in gene expression and DNA methylation profiles these results support the use of zebrafish as a nutritional model for further studies related to the epigenetic mechanisms underlying micronutrient-associated phenotypes and their inheritance across generations.

## Conclusion

Here we have demonstrated that the parental diet in zebrafish can have effects that persist in adult offspring. These effects can be observed in the DNA methylation profile of the genome, as changes in gene expression and in the liver histology. The changes observed in gene expression are consistent with the liver phenotype and are likely to underlie the lipid accumulation observed. In addition, we found locus specific DNA methylation changes as a result of parental 1-C nutrient status. Our results highlight the complex interplay between diet and physiology and how these interactions can affect gene regulation across generations. However, additional studies are required to determine the mechanisms of these interactions and how the DNA methylation memory of historical environmental stimuli is laid down and maintained.

## Methods

### Ethical considerations

The feeding experiments were carried out in accordance with the terms and guidelines of the Norwegian Regulation on Animal Experimentation and European Community Directive 86/609/EEC. The Norwegian Food Safety Authority approved the experimental protocol for zebrafish feeding trials performed in IMR’s zebrafish facility (division No. 54, reference 2012/145126).

### Feeding trial and diets

Zebrafish (AB strain) parental generation (F_0_) was fed either a diet slightly below (low 1-C diet) or above (control diet) the requirement levels given for carp^52^, whereas both groups of the descendant generation (F_1_) were fed the control diet (Fig. 1A). The 1-C nutrients folate, vitamin B12 (cyanocobalamin), vitamin B6, methionine, and choline were supplemented to the control feed, whereas the low 1-C contained only the amount present in the feed raw materials. Dietary crude composition of protein, lipids, ash and energy was analyzed as described^53^. The dietary composition is listed in Table 1. Complete dietary raw materials, ingredients and standard operating procedures for both F_0_ and F_1_ as described^16^. In short, at 15 days post fertilization (DPF) 60 F_0_ larvae were randomly assigned to six replicate tanks for each of the two dietary groups. The experimental feeds were given twice a day from 27 DPF until mating (F_0_ generation) to obtain the next generation (F_1_ generation). During the initial experimental feeding period (27–44 DPF) the fish were fed *ad libitum*, thereafter the tank biomass was reduced to 20 fish and they were fed 7% and 5% of total tank biomass until mating or sampling. The fish were maintained under standard conditions (temperature: 28 ± 1 °C, conductivity: 500 µS, pH 7.5, 14:10 h light/dark photoperiod) in an Aquatic habitats (Apopka, FL, USA) closed recirculation system.

### Crossing to obtain F_1_ generation and sampling

Mature individuals from the F_0_ generation (80 DPF) were crossed with unrelated (derived from independent crosses) individuals given the same diet to obtain the F_1_ generation. F_0_ fecundity, as well as F_1_ fertilization, hatching and survival rates have been reported^16^. Weighing of F1 fish from each of the six tanks per feed (totally 12 tanks) were performed at 27, 44, and 113DPF. Prior to weighing, the fish were anesthetized in MS222 (0.5 min in 50 mg/100 mL), briefly dried on tissue paper, and then weighed before being returned to their respective tanks. For RNA sequencing we dissected four single mature male F_1_ livers (n = 4). For reduced representation bisulfite sequencing (RRBS) we dissected six single mature male F_1_ livers (n = 6). A single fish was sampled from each of 12 tanks for RRBS (6 per feed-group) or from 8 tanks for RNA-seq (4 per feed-group). Prior to liver dissection the fish were not fed the morning meal, anesthetized as described above, weighed, and thereafter the cardinal vein was cut, as obliged. All dissections were performed between 10 and 12 am to avoid effects of the circadian rhythm. Livers were rinsed briefly in 1× PBS and prepared for histology or flash frozen in liquid nitrogen and stored in −80 °C until RNA and DNA extraction.

### RNA extraction and mRNA sequencing of F1 male livers

Four single F_1_ male livers from each diet group were defrosted in 1 mL Trizol reagent (Invitrogen, USA), homogenized using a Precellys 24 homogenizer at 3 × 15 s at 6000 rpm with 10 sec intervals and RNA extraction was performed according to Trizol manufacturer’s protocol as described^16^. RNA quantity and quality were assessed using a Nanodrop ND-1000 UV Spectrophotometer (NanoDrop Technologies) and an Agilent 2100 Bioanalyzer (Agilent Technologies), respectively.

mRNA-sequencing (mRNA-seq) and analyses were performed as described^16^. Briefly, mRNA sequencing was done by a strand specific Illumina Hi-seq run (Rapid run, 50 cycles, single end read, 5 nM concentration using the dUTP protocol to gain strand specificity), sequences were mapped to the GRCz10 version of the Ensembl zebrafish genome using STAR and the Cufflinks suite was used to estimate expression levels and differential expression. Each sample had, on average, 28 million reads, of which an average 20 million mapped uniquely (Fig. S1). The resulting data were analyzed and visualized using Perl scripts and R.

RNA sequencing verification were performed using reverse transcription followed by quantitative real-time PCR (qRT-PCR) as described^54^. Gene expression levels of *apoA4b* and *hif1al* were normalized using *ef1a* and *tuba1* as reference genes. GeNorm^55^ was used to calculate the mean normalized expression level of target genes (Table S2).

### Reduced representation bisulfite sequencing (RRBS)

Six single F_1_ livers form each feed group were resuspended in lysis buffer and added 50 ng/µL RNase A (provided by the Wizard SV Genomic DNA purification kit (Promega, WI, USA)) followed by a 10 min incubation (room temperature). DNA purification was performed according to Promega’s protocol, except that samples were treated with Proteinase K (New England Biolabs (NEB), #P8102S) and incubated for 1.5 h at 55 °C immediately following the RNase treatment. Quantification of double stranded genomic DNA was done using the Qubit High Sensitivity Assay (Life Technologies #Q32854). The RRBS library preparation, including DNA digestion with Msp1 (incubated for 16 h at 37 °C, concentration 20 U pr 100ng gDNA, NEB #R0106L), adapter ligation, quantification, pooling, bisulfite conversion (EZ DNA Methylation-Direct kit, Zymo Research # D5020) and enrichment PCR was performed on 100ng purified genomic DNA per sample, as described previously^18,56^. Library quantities were assessed by the Qubit dsDNA HS assay using Qubit 2.0 Flurometer (ThermoFisher Scientific, Q32851 and Q32866, respectively) and library fragment sizes were determined using the Experion DNA 1K Analysis kit on an Experion Automated Electrophoresis Station (Bio-Rad 700–7107 and 701–7000, respectively). Sequencing was performed on Illumina HiSeq. 2000/2500 machines.

### RRBS data processing

Sequencing reads originating from bisulfite converted libraries were trimmed of adapter sequences and low quality regions using Trimmomatic (version 0.32)^57^ and mapped to the genome (GRCz10, Ensembl zebrafish genome) using the BSMAP program^58^. The resulting bam files were then sorted and indexed using SAMtools (Ver. 1.2.)^59^ and the methylation levels extracted as bed files, bisulfite conversion rates and CpG motif counts using the biseqMethCalling.py script^18^. This pipeline estimated a mean bisulfite conversion rate of 99.2% for all samples based on cytosine methylation in non-CpG context (Fig. S6). Plots of total numbers of sequences obtained versus the numbers of unique motifs identified suggested a sufficient sequencing depth and saturated CpG coverage across all samples (Fig. S7). The methylation levels and read depths were extracted from the resulting bed files and merged using a Perl script.

### Liver histology

F_1_ livers from three fish from each parental groups were dissected at 142 DPF, rinsed in 1x PBS and fixed in 4% paraformaldehyde overnight at 4 °C, followed by dehydration, infiltration and embedding in Technovit 7100 following the manufacturers’ protocol (Kulzer Histo-Technik). Semi-thin sections (1 µm) were cut on a microtome (Leica, model #RM2155) and stained with toluidine blue. Sections were imaged on an Olympus BX51 microscope attached to a Nikon D5-Fi1 camera. Quantification of stained liver tissue was performed using ImageJ (Rasband, 1997–2015). Images were converted to RGB stacks (selected image/type/RGB stack, followed by Image/stacks/make montage) and the red channel used to segment the image into stained vs non-stained tissue. Toluidine blue is a metachromatic dye that stains proteins, nucleic acids and membranes. Non-stained tissues are lipids, solutes and the lumen of the blood vessels, bile canaliculi and sinusoids. The images were converted to binary images (image/adjust/threshold) and the percentage of stained vs non-stained tissue was measured from each fish and used as an approximate for liver lipid content.

### Statistical and data analyses

#### Weights & lipid proportions

Statistical calculations comparing weight of the two feed groups were performed in Statistica 12 (Statsoft, Inc., USA) using repeated measures ANOVA followed by Tukey’s HSD *post hoc* test. Leven’s test was applied to test for homogeneity in variance between the groups. To compare the liver lipid and solutes percentage from each groups GraphPad Prism 6 unpaired t-test (GraphPad Software, USA) was applied.

#### RNA-sequencing & differential expression

Sequences were mapped to the GRCz10 version of the Ensembl zebrafish genome using STAR (version 020201) and the Cufflinks suite (version 2.2.1) was used to estimate expression levels (Cufflinks) and differential expression (Cuffdiff). Cufflinks was used with -G option and gene locations obtained from Ensembl (GRCz10). Each sample had, on average, 28 million reads, of which an average 20 million mapped uniquely (Fig. S1). The resulting data were analyzed and visualized using Perl scripts and R. The specific commands and scripts used for RNA sequencing and RRBS are reported in Tables S9 and S10, respectively. Permutation analyses were carried out using a Perl script to generate appropriate commands to run Cuffdiff with the appropriate sample permutations. k-means clustering was done with the base R kmeans() function using log transformed FPKM values.

Analysis of enrichment of functional groups (gene ontology and KEGG) was performed using DAVID 6.7^20^. We used human orthologues (exported from Ensembl biomart) as input to DAVID, and the full set of identified orthologues was used as background. Genes belonging to specific GO annotations were identified using the org.Hs.eg.db Bioconductor (version 3.2.3) package^60^, and mapped back to zebrafish orthologues. Colors in all heatmaps indicate row-normalized log FPKM values. The probability of observing a given number or larger of downregulated genes in a given functional grouping was calculated using the hypergeometric function using the R phyper() function. Mitochondrial genes (eg. *mrps*, *mrpl*) were identified by searching the gene names using the R regular expression function (grep).

#### Differential DNA methylation analyses

Tables containing the total and methylated number of reads were read into an R-session and analyzed using the DSS package^61^ or base R to inspect overall properties and to identify differentially methylated sites and regions. To use DSS we created a BSseq object^62^ and used the DML test function with default parameters to identify differentially methylated sites. To estimate FDR we made a set of 100 random permutations (using the R sample() function) of the samples and ran the DML test as for the original permutations. We calculated simple f- statistics (variance between sample groups/variance within groups) for all sites that had a minimum coverage of 10 reads; these were used to identify differentially methylated regions (DMR) using a running-sum method inspired by the code used to identify CpG islands in the genome (G. Micklem, personal communication) and implemented as a C++ extension to R. The genome is traversed and the score (s_i_) at the i^th^ CpG position is calculated as: max(0, s_(i−1)_ + log_2_(f_i_/mean(f)) − p(d_i_ − d_(i−1)_)), where f_i_ and d_i_ are the f statistic and genomic position respectively at the i^th^ CpG, p is a penalty associated with the physical separation of neighbouring CpG loci and mean(f) is the mean of all f statistics. A DMR is defined each time the local score drops to 0 and the prior score was above 0; this DMR is defined from its first CpG to the CpG giving the maximal score. The score of a given DMR is the maximal score which will evaluate to the sum of its containing log-ratios minus its width minus one multiplied by the separation penalty. For the windows shown we used a separation penalty of 0.02. Gene locations were obtained from the Ensembl danio_rerio_core_88_10 database using the RMysql package. Primary components analyses were carried out using the prcomp() function with scale set to FALSE. Scripts used to run major commands, R code and associated functions (in C and C++) used for this analysis are available on request, or in Tables S9 and S10. Raw sequences reads are available at http://www.ncbi.nlm.nih.gov/bioproject/413770, and BioSample accessions number SAMN07765782-SAMN07765801 for RRBS and RNA sequence samples.

## Electronic supplementary material

Supplementary info, figures and tables

Figure S12

Figure S13

Figure S14

Dataset 1
