# Supplementary material for: Parental micronutrient deficiency distorts liver DNA methylation and expression of lipid genes associated with a fatty-liver-like phenotype in offspring
Source: Sci Rep. 2018 Feb 14;8:3055. doi: 10.1038/s41598-018-21211-5 (PMC5812986; doi:10.1038/s41598-018-21211-5)
Supplement: Supplementary file 1 — Supplementary info, figures and tables [file 41598_2018_21211_MOESM1_ESM.pdf]

Supplementary Info, figures and tables:

Parental micronutrient deficiency distorts liver DNA methylation and expression of lipid genes associated with a fatty-liver-like phenotype in offspring

Kaja H. Skjærven[1*], Lars Martin Jakt[2], Jorge M.O. Fernandes[2], John Arne Dahl[3], Anne-Catrin Adam[1], Johanna Klughammer[4], Christoph Bock[4] and Marit Espe[1]

[1]*Institute of Marine Research, IMR, Norway*

[2]*Faculty of Biosciences and Aquaculture, Nord University, Norway*

[3]*Department of Microbiology, Oslo University Hospital, Norway*

[4]*CeMM Research Center for Molecular Medicine of the Austrian Academy of Sciences, 1090 Vienna, Austria*

[*]Corresponding author:

Dr. Kaja Helvik Skjærven

Institute of Marine Research (IMR),

PO Box 1870 Nordnes,

5817 Bergen, Norway

Tel.:+4741458362; fax: +4755905299.

E-mail address: ksk@hi.no

Supplementary Info, figures:

Figure S1:

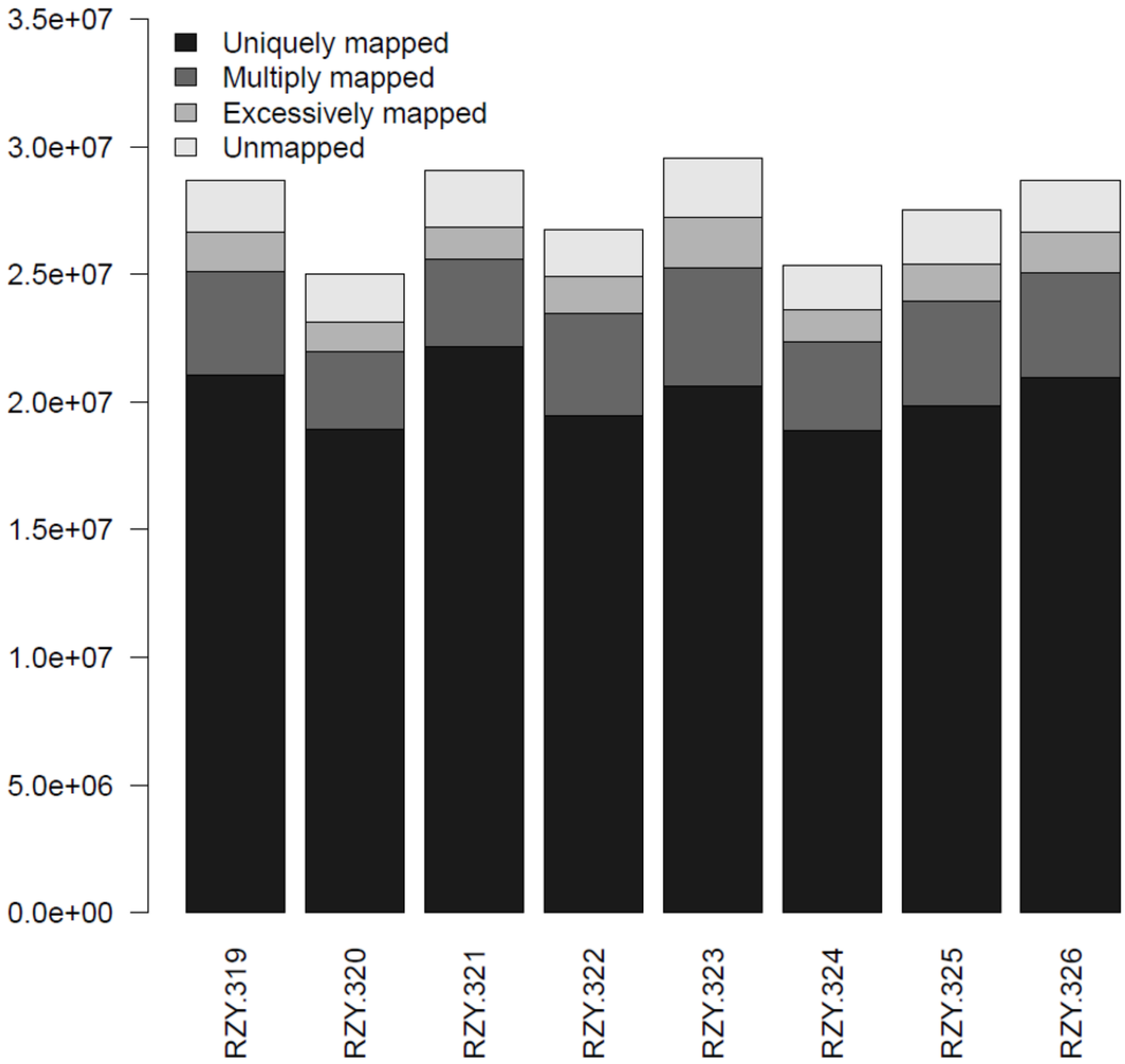

Figure S1: Mapping statistics

Mapping statistics from STAR for Illumina Hi-sequencing run indicating total reads: uniquely mapped, multiply mapped, excessively mapped and unmapped reads for each of the four independent samples from each feeding group: F1 generation Low 1-C samples: RZY.319-322 and control samples: RZY.323-326

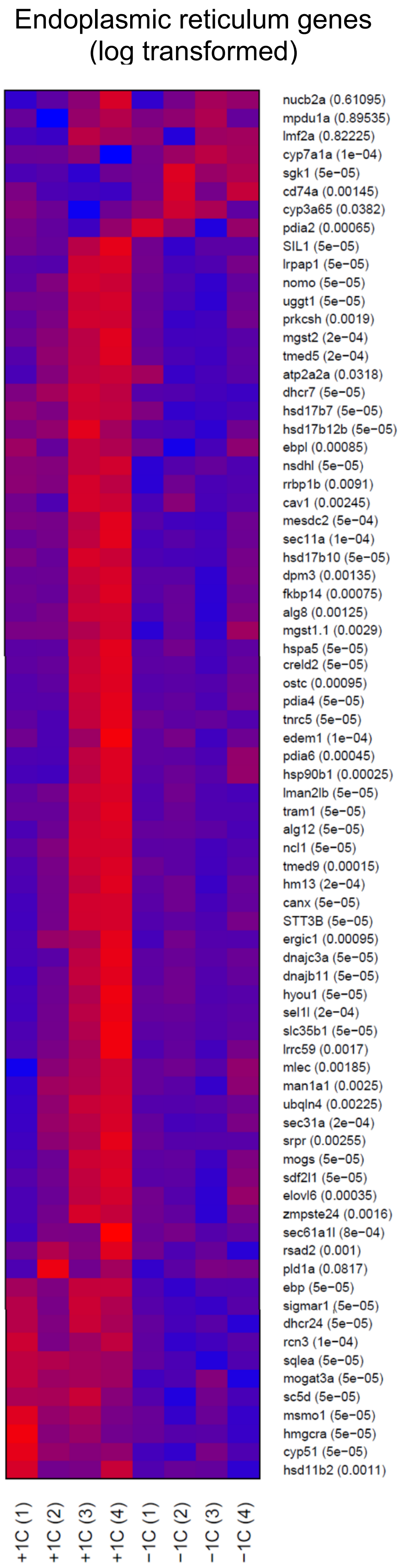

Figure S2: RNA differential expression of endoplasmic reticulum proteins

Heat map of mRNA expression levels of significantly affected sequences encoding proteins associated to endoplasmic reticulum from control (+1C) and low 1-C (-1C) F_1 male livers (genes in rows, samples in columns). The low 1-C F_1 male livers have significantly lower expression levels than control F_1 male livers. Expression levels are indicated by colour, with blue to red indicating min to max expression level for each gene, respectively. P-values from Cuffdiff are indicated next to the gene names.

Figure S3:

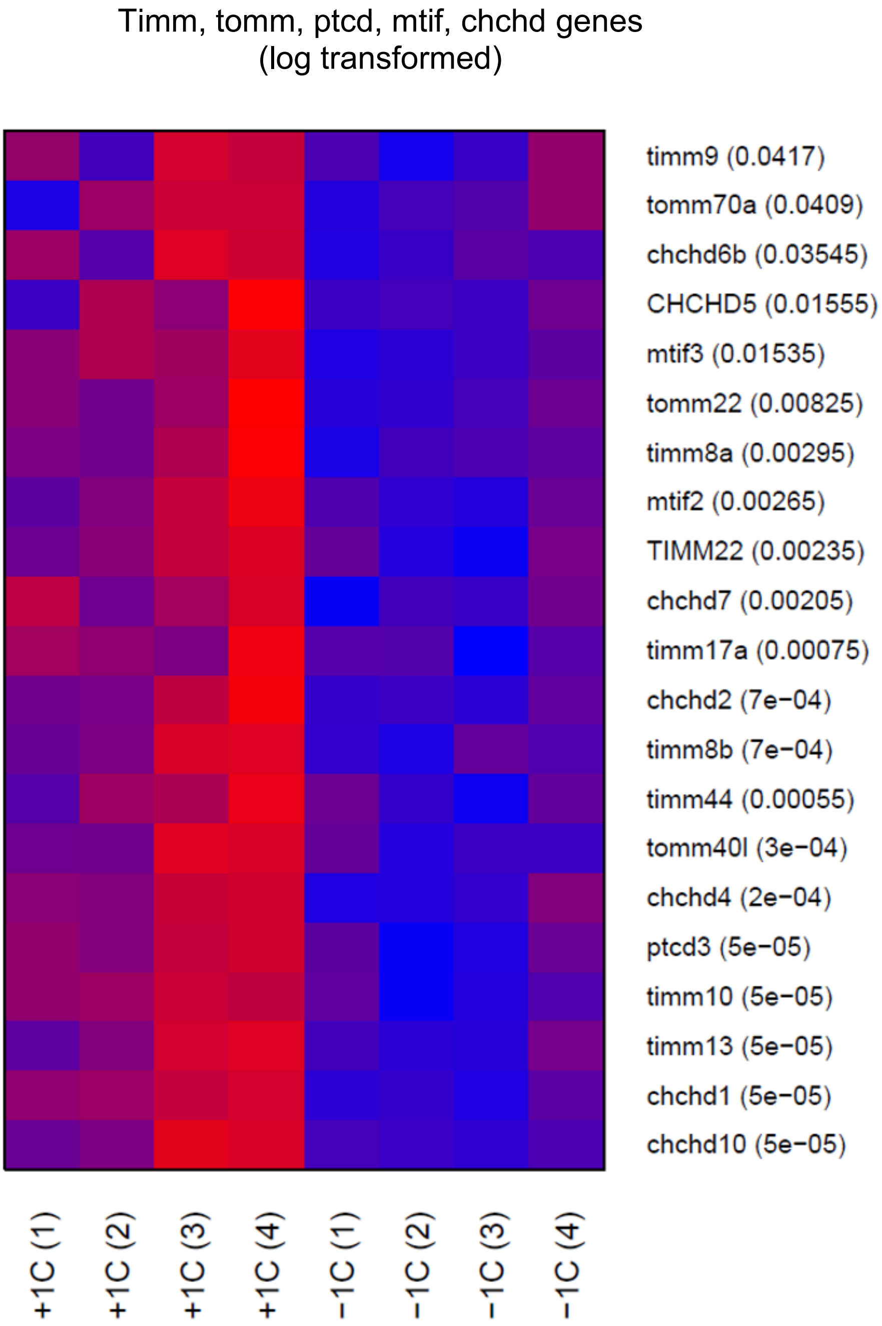

Figure S3: RNA differential expression of mitochondrial proteins
Heat map of mRNA expression levels of significantly affected sequences encoding mitochondrial proteins from control (+1C) and low 1-C (-1C) F_1 male livers. The low 1-C F_1 male livers have significantly lower expression levels than control F_1 male livers. Expression levels are indicated by colour, with blue to red indicating min to max expression level for each gene, respectively. P-values are indicated next to the gene names.

Figure S4:

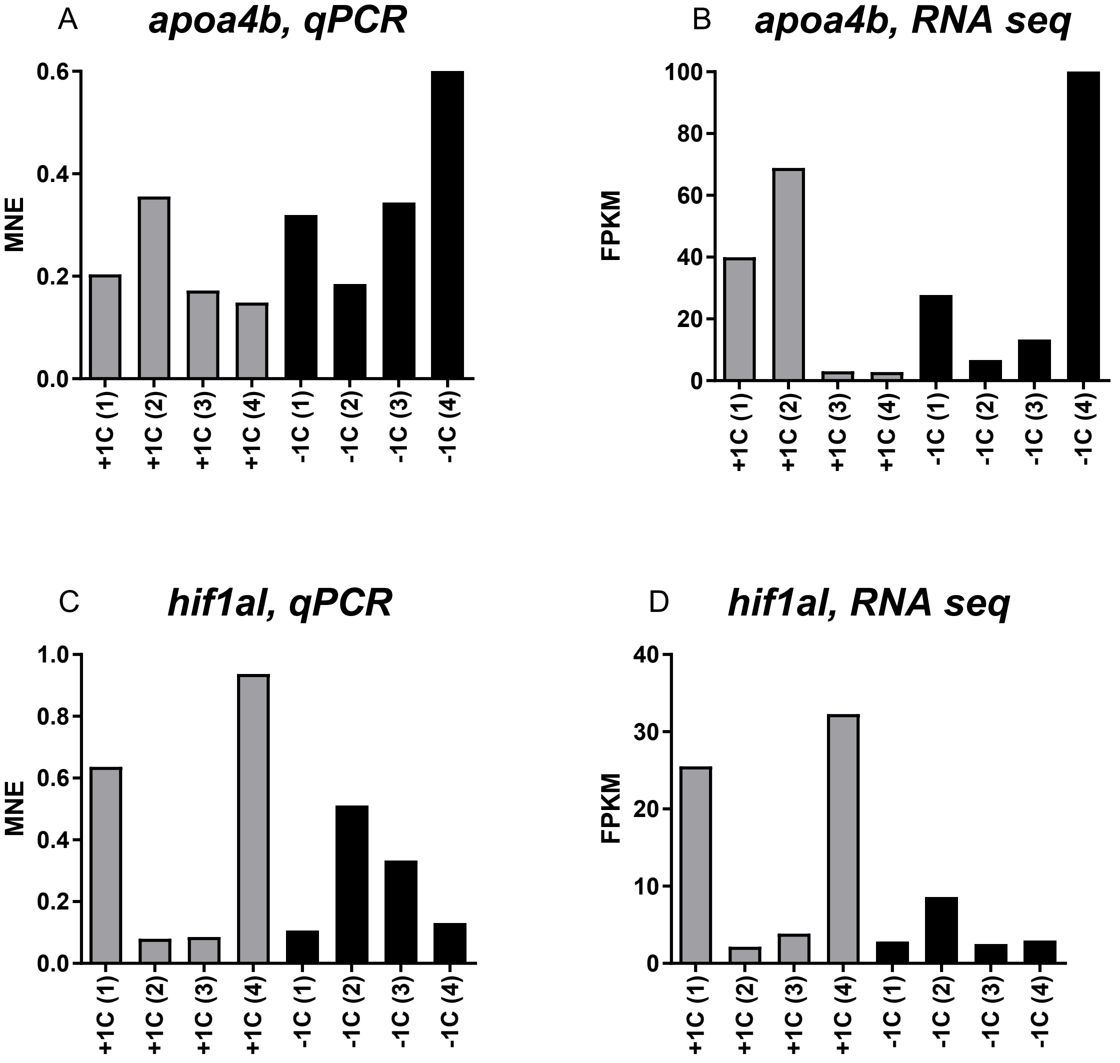

Figure S4: RNA sequencing verification by RT-qPCR.

mRNA levels from four independent groups (n=4, four feeding tanks per feed group) of mature male F1 control livers (+1C) versus low 1-C livers. The RNA used for both qRT-PCR and RNA sequencing were from the same fish and RNA extraction. Mean normalized expression (MNE of target genes are normalized against *ef1a* and *tuba1* as reference genes. Gene abbreviations, gene name and gene bank accession numbers are given in table S2.

Figure S5:

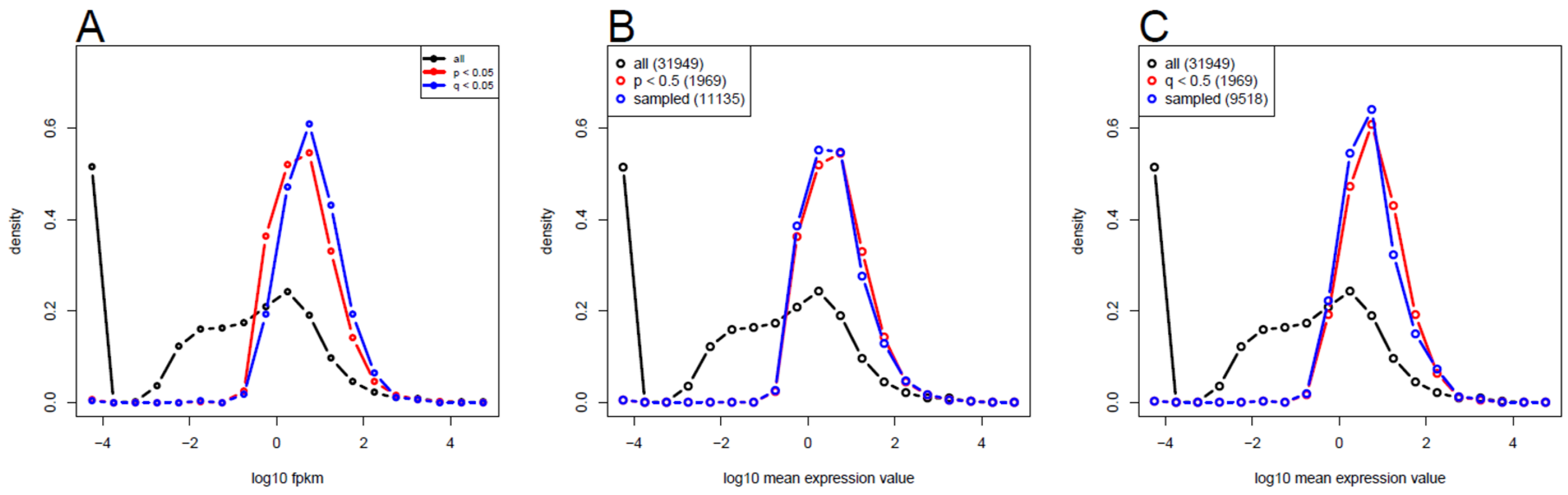

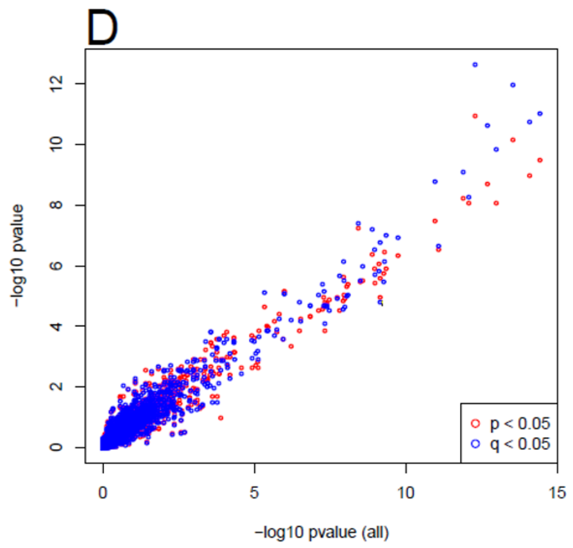

E

	All			p < 0.05 (6x)			q < 0.05 (16x)			
	p value	ratio	count	p value	ratio	count	p value	ratio	count	size
sterol biosynthetic process	3.6e−15	18.5	18	3.2e−10	11.9	15	9.2e−12	13.0	17	41
secondary alcohol biosynthetic process	8.0e−15	20.0	17	1.1e−09	12.1	14	1.7e−11	13.9	16	37
alcohol biosynthetic process	2.9e−14	10.7	22	7.0e−11	8.9	19	1.1e−12	10.2	21	59
cholesterol biosynthetic process	1.0e−13	18.7	16	8.4e−09	11.2	13	1.5e−10	13.0	15	36
protein folding	2.0e−13	6.2	30	2.0e−09	4.6	27	2.3e−11	5.3	30	137
organic hydroxy compound biosynthetic process	5.0e−13	7.1	26	1.1e−11	7.5	23	2.2e−13	8.5	25	80
single−organism metabolic process	8.3e−13	2.2	166	8.3e−09	1.9	159	5.5e−09	1.9	161	2040
small molecule biosynthetic process	1.3e−12	4.1	43	5.8e−09	3.2	40	7.9e−10	3.4	42	279
small molecule metabolic process	8.3e−12	2.4	98	2.8e−07	1.9	94	2.3e−07	1.9	95	1074
steroid biosynthetic process	1.1e−11	7.1	23	3.2e−08	5.3	20	1.6e−09	5.9	22	91
response to topologically incorrect protein	1.8e−10	5.7	24	4.6e−07	3.9	23	1.1e−07	4.1	24	132
sterol metabolic process	4.4e−10	6.8	20	1.2e−06	4.7	17	1.0e−07	5.2	19	86

Figure S6: Specificity of functional enrichment

A. Genes identified as differentially expressed are selected from the subset of genes expressed in the liver samples. Plot shows the distributions of gene expression level for all genes (black), genes with a $q < 0.05$ (red) and $p < 0.05$ (blue). B and C show the distributions of genes sampled to have a similar distribution of expression levels as for differentially expressed genes. The blue and red lines indicate the distributions of the sampled gene sets and the differentially expressed genes respectively. The sets were sampled to recreated distributions similar to those seen for genes with a p-value (B) and q-value (C) less than 0.05. Human orthologues of the resulting sampled gene sets were used as the universe sets for gene enrichment analyses of the genes down-regulated in the low 1-C group ($q < 0.05$) using the GOstats package and the Biological Process gene ontology. D. Enrichment p-values obtained using the full set of genes (X) plotted against p-values obtained using the two sampled populations (Y). E. Enrichment parameters for the top 12 categories identified in the full analysis. There is a small difference in the order of the enrichments, but similar enrichments are seen for all analyses. Columns are; p value: the probability of selecting at least the number of class members, ratio: the enrichment ratio, count: the number of class members down-regulated, size: the number of members of the group in the $q < 0.05$ universe.

Figure S6:

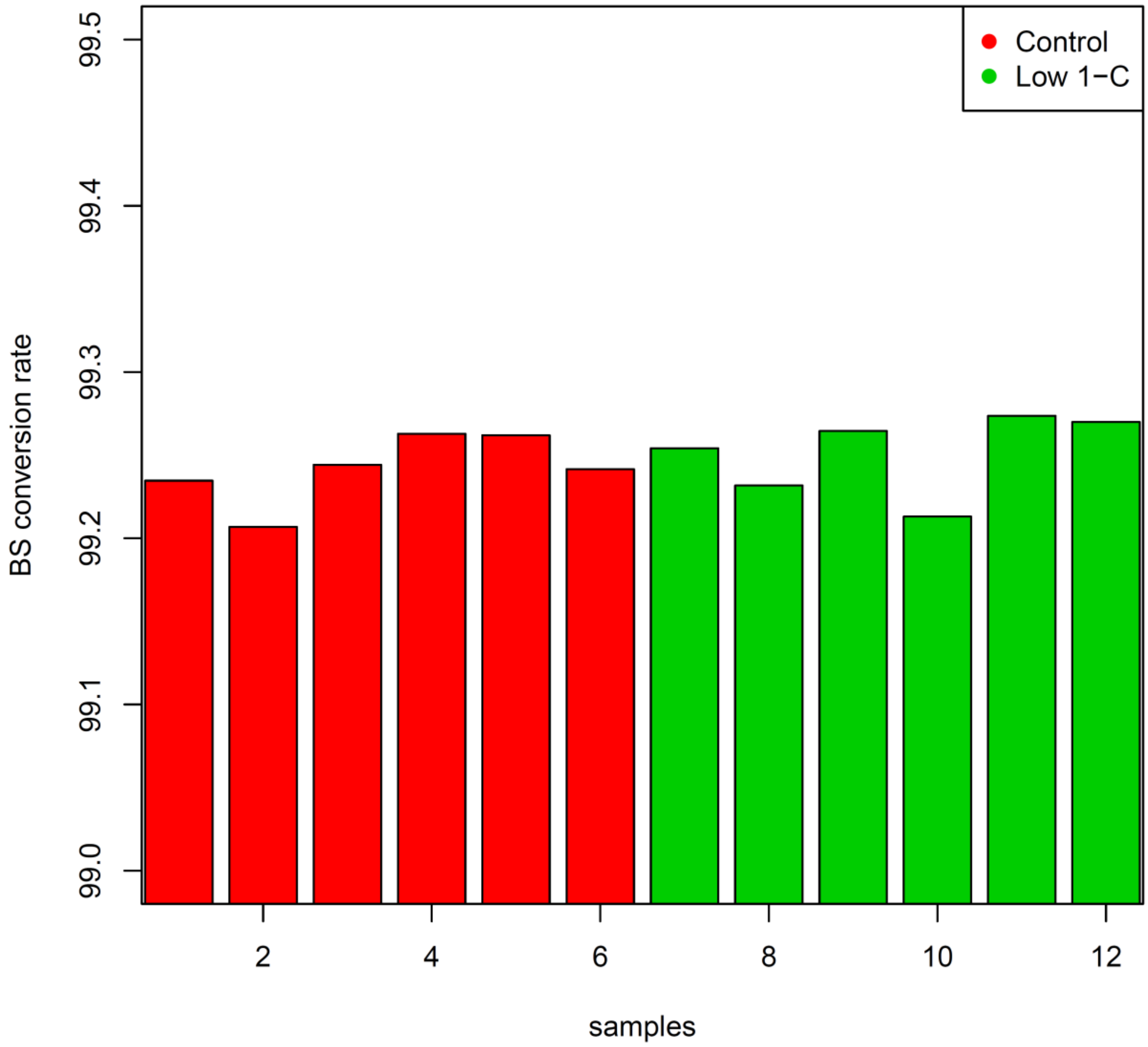

Figure S6: Bisulfite conversion rate is equally high in all samples
Bisulfite conversion rates were estimated by the biseqMethCalling.py script from Bam files created by the BSMAP program using control sequences.

Figure S7:

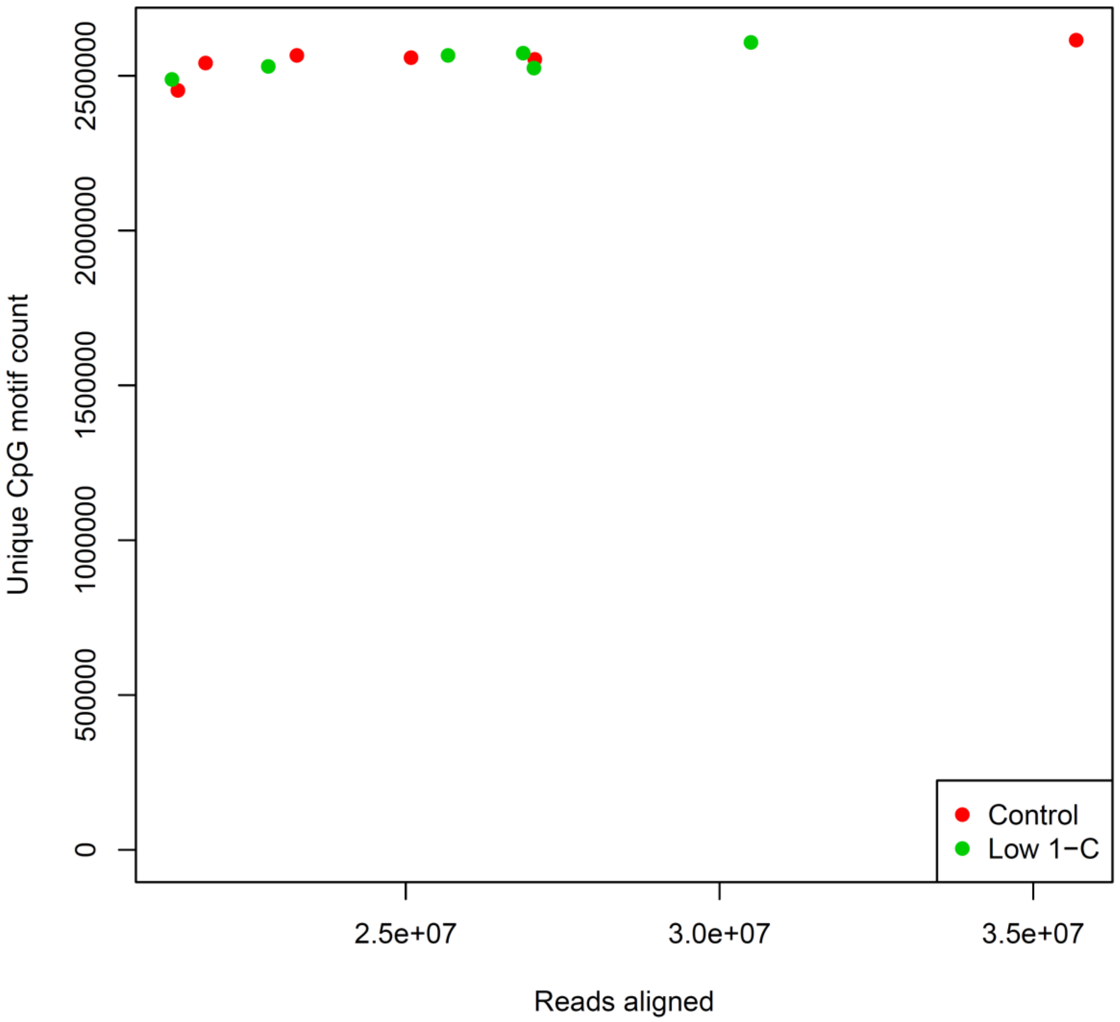

Figure S7: Unique CpG versus aligned reads shows no difference in sequencing between samples

Numbers of Unique CpG motifs identified from alignments plotted against total numbers of sequences aligned to the zebrafish genome. The numbers of identified sites does not increase with increasing sequencing depth indicating that a sufficient sequencing read depth.

Figure S8:

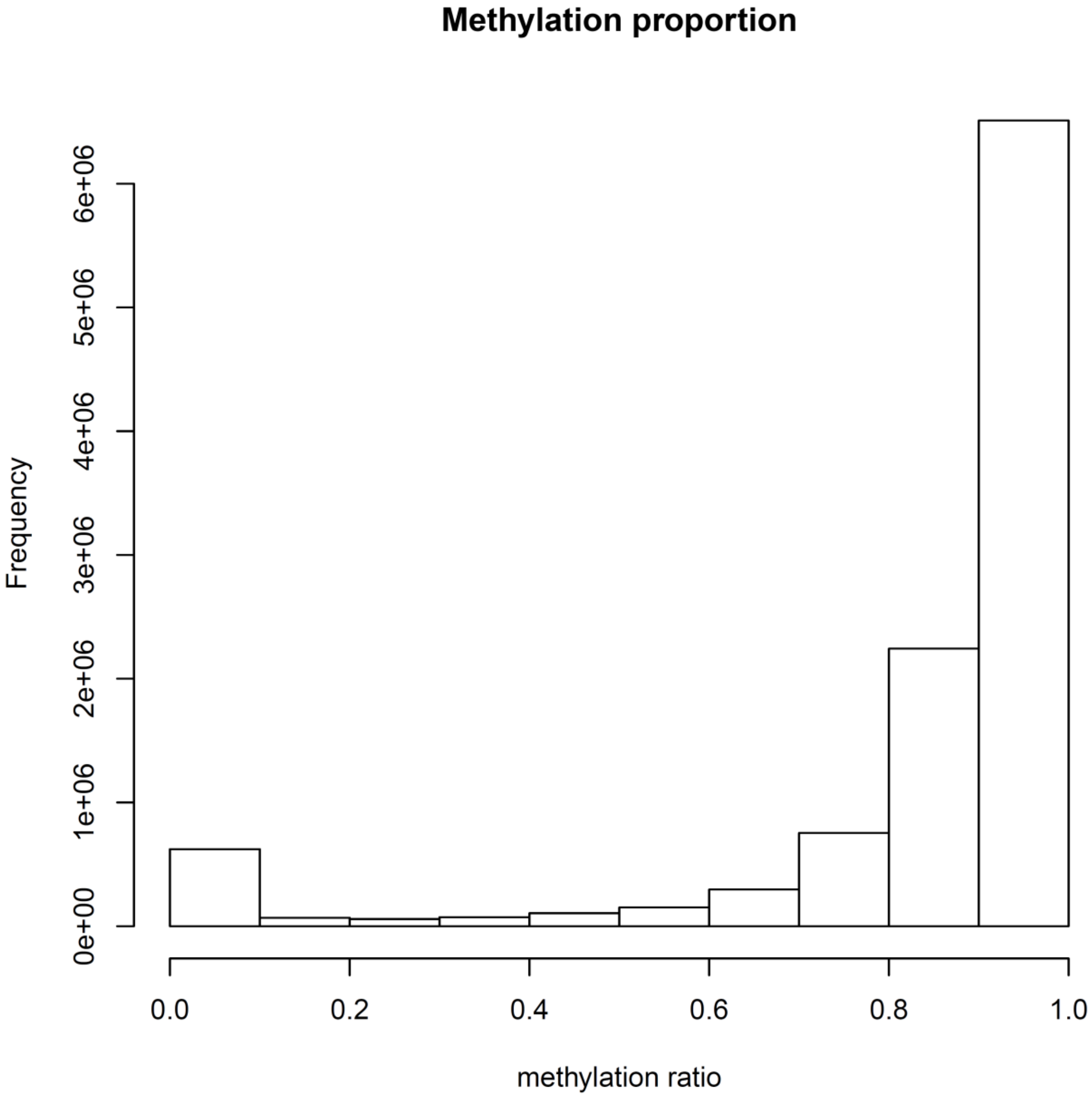

Figure S8: Methylation distribution at differentially methylated loci

Distribution of methylation levels, with the methylation level on the x-axis and the numbers of sites at each methylation level given on the y-axis. Most sites are either almost completely methylated or de-methylated.

Figure S9:

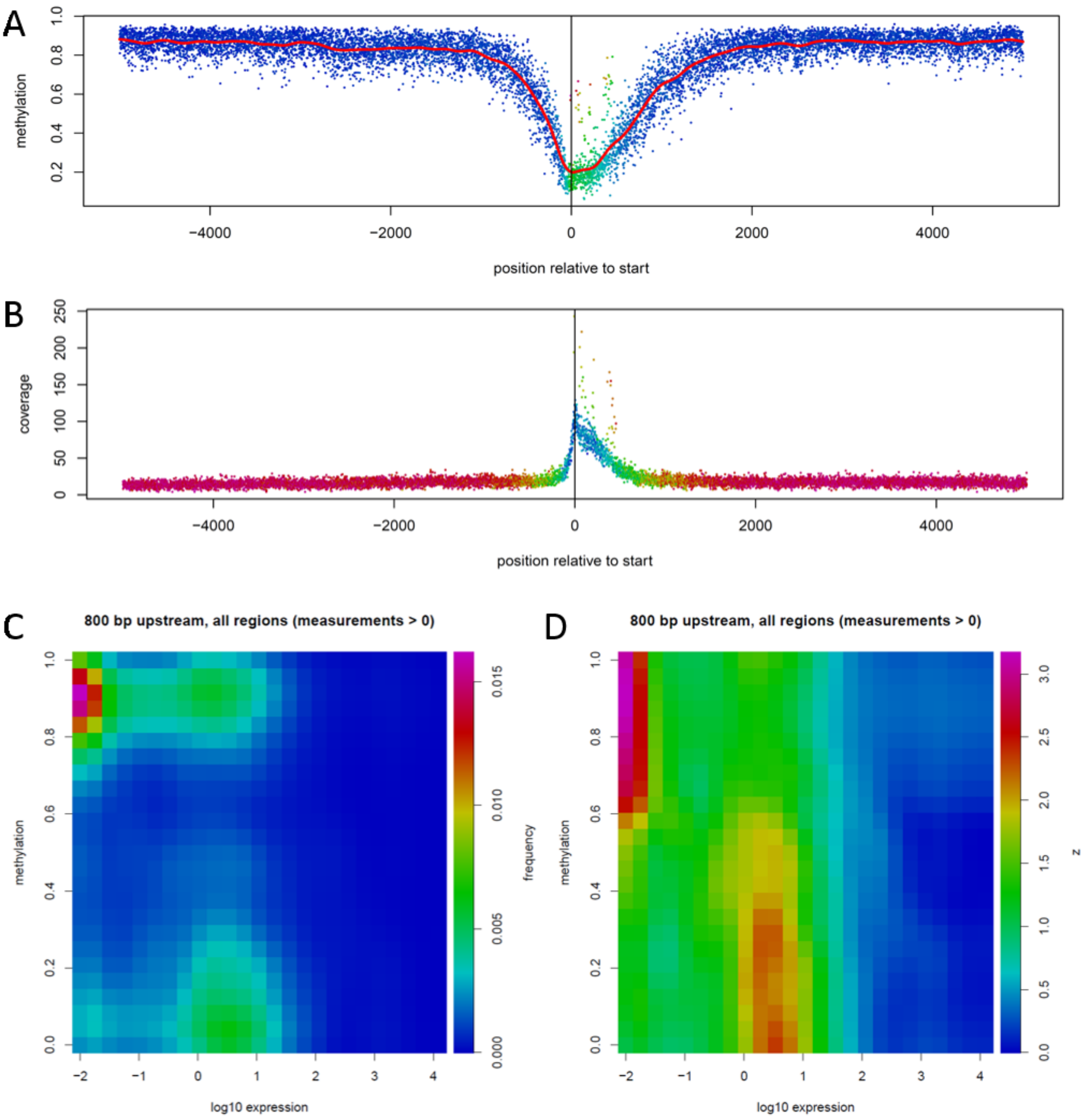

Figure S9: DNA methylation

Methylation levels decrease around transcriptional start sites (TSSs). DNA methylation and sampling depth around TSSs. **A)** Mean DNA methylation at positions relative to TSSs. Each point gives the mean methylation of measurements offset from the TSS by the indicated number of bases. Color indicates the number of measurements comprising the mean value. **B)** The number of measurements contributing to the mean values in A) with color indicating the methylation levels as plotted in A).

C and **D)** Two-dimensional kernel density estimates of the relationship between promoter methylation and gene expression. The density estimates were obtained from the mean methylation of 800 bp regions upstream of all TSSs reported by Ensembl and mean gene expression measurements using the kde2d function of the Mass package. Color indicates the frequency of measurement pairs. C) Raw frequencies, D) frequencies normalised by row indicating the distributions of expression levels for different levels of promoter methylation. DNA methylation levels are generally close to 0 or 1; genes associated with high DNA methylation levels are mostly not expressed though a sizeable proportion of these are expressed at normal levels. This may partly be explained by differential promoter utilization as we have not attempted to map 5' ends from the sequencing data.

Figure S10:

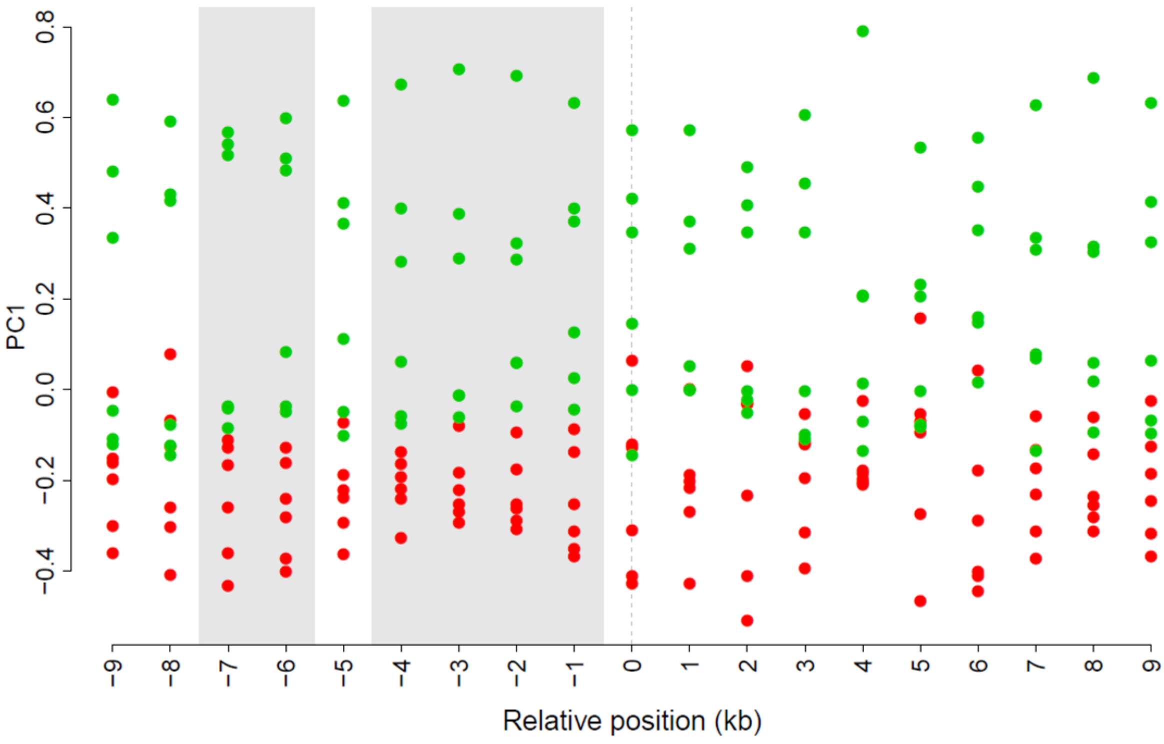

Figure S10: Principal component analyses

Principal components analyses (PCA) for methylation levels at all CpG positions in the first components for PCAs carried out on mean methylation levels of non-nested regions upstream (negative numbers) and downstream (positive numbers) of TSSs (0) .

Figure S11:

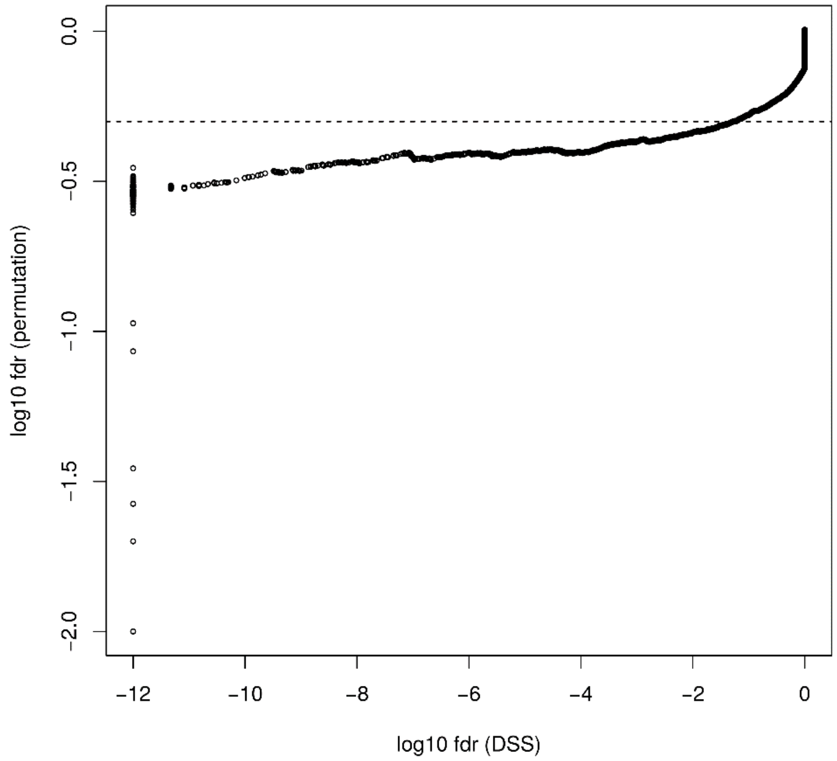

Figure S11: False discovery rate estimation
False discovery rates (fdrs) as reported by the DSS algorithm plotted against fdrs estimated by 100 random sample permutations. The minimal fdr that can be calculated by 100 permutations is 0.01, and the minimal fdr reported by DSS is approximately 3e-12. To allow log-scaling of 0 values we added either 0.01 to permutation estimates or 1e-12 to DSS estimates. The dashed line indicates an fdr of 0.

Figure S12: 100 differentially methylated regions, DMRs (attached as Supplementary Info Figure S12)
Chromosomal context of the 100 most differentially methylated regions (DMRs). Upper panel: Each point indicates the methylation level at a single CpG position in a single sample. The feed groups are indicated by color with purple and green representing the control and low 1-C samples, respectively. The point size maps to the number of reads used to derive the methylation level estimate. Solid circles indicate points identified by DSS (methods) as significantly differentially methylated (fdr <= 0.05). Middle panel: DMRs were identified as regions containing higher f-statistics using the log-ratio of f / mean(f) in a running sum method. The log-ratios for CpGs having at least 10 reads for each sample are shown in the middle panel. The lower panel gives an expanded view of the genomic locus containing the gene locations on the chromosomes, with arrows and colors (red: forward, green: reverse) indicating the direction of transcription.

Figure S13: 100 most differentially methylated loci, DML, identified by DSS (attached as Supplementary Info Figure S13)
Differentially methylated loci (DML) identified by DSS (methods) and their chromosome locations. Upper panel: Each point indicates the methylation level at a single CpG position in a single sample. The feed groups are indicated by color with purple and green representing the control and low 1-C samples, respectively. The point size maps to the number of reads used to derive the methylation level estimate. Solid circles indicate points identifed by DSS as significantly differentially methylated (fdr <= 0.05). Middle panel: log10 p-values reported by DSS for differential methylation. The lower panel gives an

expanded view of the genomic locus containing the gene locations on the chromosomes, with arrows and colors (red: forward, green: reverse) indicating the direction of transcription.

Figure S14: 100 DMLs with highest f-statistics (attached as Supplementary Info Figure S14)
Differentially methylated loci (DML) identified by a simple f-statistic (variance between means / variance within means) and their chromosome locations. Upper panel: Each point indicates the methylation level at a single CpG position in a single sample. The feed groups are indicated by color with purple and green representing the control and low 1-C samples, respectively. The point size maps to the number of reads used to derive the methylation level estimate. Solid circles indicate points identified by DSS as significantly differentially methylated (fdr <= 0.05). Middle panel: log2 transformed f-statistics. The lower panel gives an expanded view of the genomic locus containing the gene locations on the chromosomes, with arrows and colors (red: forward, green: reverse) indicating the direction of transcription.

Supplementary Info, tables:

Table S1: Complete list of 663 differentially expressed genes in F_1 low 1-C male livers compared to control (attached separately as supplementary data, Table S1)

Table S2: Gene abbreviation, gene name, gene bank accession number, forward and reverse primer sequence, primer amplicon size and qRT-PCR efficiency for selected reference and target genes analysed.

Gene abbr.	Gene name	Accession no.	Forward primer (5' - 3')	Reverse primer (5' - 3')	Amplicon size (bp)	PCR eff.
**Reference genes:**						
ef1α	*Elongation factor 1 α*	NM_131263.1	AGACAACCCCAAGGCTCTCA	CTCATGTCACGCACAGCAAA	126	1,81
tuba1	*tubulin, alpha 1c*	NM_001105126	GGTGCCCTCAATGTGGATCT	GCCACAGAGAGCTGCTCATG	131	1,99
**Target genes:**						
apoa4b.2	*apolipoprotein A-IV b, tandem duplicate 2*	NM_001128758.1	AAGCAACACAAACCGCAGAG	CGGCGTATTCACTGGCCATA	111	2,05
hif1al	*hypoxia-inducible factor 1, alpha subunit, like*	NM_200405.1	TCTACGTCGAAAAGGCCCAG	GCGCAGATAGCTGAGTGTCA	181	1,88

Table S3: Complete list of significantly altered Gene Ontologies for biological processes, molecular function and cellular components for differentially expressed genes in F_1 low 1-C male livers compared to control. The significant GO terms with altered number of genes, gene name, p-value and Benjamini values are listed.

**Biological process**			**Genes**	**Gene ID**	**P-value**	**Benjamini**
1	GOTERM_BP_FAT	GO:0016126~sterol biosynthetic process	18	*ebp, sqlea, mvda, sigmar1, nsdhl, msmo1, C14orf1, dhcr7, mvk, fdps, hmgcra, cyb5r2, dhcr24, hmgcs1, cyp51, lss, sc5d, idi1*	5.19E-17	1.10E-13
2	GOTERM_BP_FAT	GO:0006694~steroid biosynthetic process	23	*rdh8a, ebp, sqlea, hsd17b12b, mvda, nsdhl, sigmar1, msmo1, hsd11b2, C14orf1, dhcr7, mvk, fdps, hmgcra, cyb5r2, hsd17b7, hsd17b3, dhcr24, hmgcs1, cyp51, lss, sc5d, idi1*	9.79E-15	1.03E-11
3	GOTERM_BP_FAT	GO:0016125~sterol metabolic process	24	*cel.2, ebp, ebpl, sqlea, lipf, mvda, nsdhl, sigmar1, msmo1, C14orf1, ldlrb, dhcr7, mvk, fdps, cyp7a1a, hmgcra, sorl1, cyb5r2, dhcr24, hmgcs1, cyp51, lss, sc5d, idi1*	4.84E-14	3.40E-11
4	GOTERM_BP_FAT	GO:0008202~steroid metabolic process	31	*cel.2, ebpl, ebp, sqlea, lipf, cyp24a1, hsd17b12b, hsd11b2, C14orf1, dhcr7, fdps, cyp7a1a, hmgcra, cyb5r2, hsd17b3, hmgcs1, cyp51, rdh8a, mvda, nsdhl, sigmar1, msmo1, mvk, ldlrb, sorl1, cyp3a65, hsd17b7, dhcr24, lss, sc5d, idi1*	1.11E-12	5.88E-10
5	GOTERM_BP_FAT	GO:0008610~lipid biosynthetic process	38	*scdb, pdss2, agpat5, ebp, sqlea, pld1a, hsd17b12b, hsd11b2, C14orf1, mboat2a, dhcr7, fdps, hmgcra, mgst2, cyb5r2, hsd17b3, hmgcs1, cyp51, dpm3, ptges3b, mogat3a, rdh8a, cd74a, elovl6, prp19, mvda, nsdhl, sigmar1, msmo1, mvk, rnpep, hsd17b7, dhcr24, lss, fasn, sc5d, alg12, idi1*	8.00E-12	3.38E-9
6	GOTERM_BP_FAT	GO:0006695~cholesterol biosynthetic process	12	*mvk, dhcr7, fdps, ebp, hmgcra, dhcr24, mvda, hmgcs1, cyp51, nsdhl, lss, idi1*	1.28E-10	4.49E-8
7	GOTERM_BP_FAT	GO:0008203~cholesterol metabolic process	17	*cel.2, ebp, sqlea, mvda, nsdhl, dhcr7, mvk, ldlrb, fdps, cyp7a1a, hmgcra, sorl1, dhcr24, hmgcs1, cyp51, lss, idi1*	2.32E-8	7.01E-6
8	GOTERM_BP_FAT	GO:0055114~oxidation reduction	45	*scdb, dhrs13b, loxl2a, rdh12, tdo2b, sqlea, pgd, gpx1a, dao.2, idh3a, cyp24a1, hsd17b12b, impdh1a, si:ch73-209e20.3, hsd11b2, dhcr7, cyp7a1a, hmgcra, cyb5r2, ugdh, aldh16a1, HSD11B1L (1 of 2), hsd17b3, cyp51, pipox, rdh8a, sdhb, aass, cyb5b, FTL (2 of 2), nsdhl, hccsa.1, msmo1, rnls, hsd17b10, cox10, txn2, UQCR10, cyp3a65, mpx, hsd17b7, dhcr24, fasn, ldhbb, sc5d*	7.54E-7	1.99E-4

9	GOTERM_BP_FAT	GO:0046394~carboxylic acid biosynthetic process	19	*scdb, cbsb, pld1a, cd74a, glula, elovl6, bcat1, si:ch73-209e20.3, msmo1, asns, mgst2, rnpep, SRR (2 of 2), ugdh, MTR, cthl, fasn, ptges3b, sc5d*	1.72E-6	4.03E-4
10	GOTERM_BP_FAT	GO:0016053~organic acid biosynthetic process	19	*scdb, cbsb, pld1a, cd74a, glula, elovl6, bcat1, si:ch73-209e20.3, msmo1, asns, mgst2, rnpep, SRR (2 of 2), ugdh, MTR, cthl, fasn, ptges3b, sc5d*	1.72E-6	4.03E-4
11	GOTERM_BP_FAT	GO:0006457~protein folding	20	*pdia2, pfdn2, LMAN2L (2 of 2), pdia6, dnajb11, hsp90b1, fkbp14, cd74a, hspd1, lrpap1, SIL1, trap1, hspe1, ruvbl2, grpel1, canx, hspa9, mpdu1a, uggt1, alg12*	2.92E-6	6.17E-4
12	GOTERM_BP_FAT	GO:0008299~isoprenoid biosynthetic process	7	*mvk, fdps, pdss2, hmgcra, mvda, hmgcs1, idi1*	2.44E-5	4.68E-3
**Molecular function**			**Genes**	**Gene ID**	**P-value**	**Benjamini**
1	GOTERM_MF_FAT	GO:0051082~unfolded protein binding	16	*pfdn2, hspa5, hsp90b1, dnajb11, hspd1, lrpap1, SIL1, trap1, hspe1, ruvbl2, grpel1, canx, tubb4b, hspa9, uggt1, ptges3b*	2.85E-6	2.11E-3
2	GOTERM_MF_FAT	GO:0016860~intramolecular oxidoreductase activity	9	*pdia4, pdia2, ddt, pdia6, ebpl, ebp, sigmar1, ptges3b, idi1*	1.83E-5	6.77E-3
**Cellular component**			**Genes**	**Gene ID**	**P-value**	**Benjamini**
1	GOTERM_CC_FAT	GO:0005783~endoplasmic reticulum	79	*scdb, sel1l, cav1, ebp, zmpste24, ebpl, sqlea, ostc, alg8, pld1a, tmed9, hyou1, lrpap1, SIL1, prkcsh, hsd11b2, rrbp1b, dhcr7, C14orf1, nucb2a, hmgcra, mgst2, tnrc5, cyp51, dpm3, uggt1, man1a1, pdia2, LMAN2L (2 of 2), pdia6, hsp90b1, lrrc59, sdf2l1, cd74a, creld2, ncl1, nsdhl, srpr, ergic1, sec11a, rcn3, tmed5, cyp3a65, rsad2, sc5d, insb, hspa5, hsd17b12b, pdia4, cyp7a1a, canx, sec61al2, lmf2a, mgst1.1, sec31a, slc35b1, mpdu1a, STT3B, mogat3a, atp2a2a, dnajb11, mesdc2, fkbp14, mogs, nomo, mlec, elovl6, tram1, edem1, hm13, sigmar1, dnajc3a, msmo1, hsd17b10, hsd17b7, dhcr24, ubqln4, sgk1, alg12*	2.26E-14	7.78E-12
2	GOTERM_CC_FAT	GO:0044432~endoplasmic reticulum part	42	*hspa5, ostc, sqlea, alg8, hyou1, lrpap1, SIL1, dhcr7, pdia4, rrbp1b, cyp7a1a, hmgcra, sec61al2, cyp51, dpm3, mpdu1a, uggt1, STT3B, pdia2, LMAN2L (2 of 2), pdia6, dnajb11, atp2a2a, hsp90b1, fkbp14, sdf2l1, nomo, elovl6, tram1, edem1, srpr, sigmar1, nsdhl, msmo1, sec11a, rcn3, cyp3a65, hsd17b7, dhcr24, ubqln4, insb, alg12*	6.43E-13	1.11E-10

3	GOTERM_CC_FAT	GO:0005739~mitochondrion	76	*cav1, agpat5, mrpl14, acsbg2, si:ch73-209e20.3, mcl1a, mrpl43, trap1, si:ch211-149b19.3, sh3bp5b, slc25a32a, tufm, dlat, chchd2, OXR1, sdhb, pdk2a, aass, lrrc59, abcb8, mrpl41, hspd1, mrpl35, slc25a48, hspe1, txn2, cox10, samm50l, UQCR10, slc25a43, mrpl18, timm10, mrps18c, grpel1, timm44, timm8b, endog, mrpl19, qars, mrps16, acp6, cyp24a1, idh3a, DNAJC11 (2 of 2), tomm40, ptcd3, hcls1, fam136a, timm13, mrpl1, mgst1.1, abce1, phb2a, TIMM22, aco2, CHCHD4 (2 of 2), vars, agk, lap3, cyb5b, timm17a, elovl6, glula, tmem186, hccsa.1, cox17, stoml2, hsd17b10, slc25a39, dap3, mtif2, amt, mrps31, phb, hspa9, lonp1*	2.43E-10	2.79E-8
4	GOTERM_CC_FAT	GO:0044429~mitochondrial part	51	*mcl1a, si:ch73-209e20.3, mrpl43, si:ch211-149b19.3, slc25a32a, tufm, dlat, pdk2a, sdhb, lrrc59, abcb8, hspd1, mrpl41, mrpl35, slc25a48, cox10, hspe1, samm50l, mrpl18, slc25a43, timm10, UQCR10, mrps18c, grpel1, timm44, timm8b, qars, mrps16, tomm40, DNAJC11 (2 of 2), idh3a, cyp24a1, timm13, mrpl1, mgst1.1, phb2a, aco2, TIMM22, CHCHD4 (2 of 2), agk, cyb5b, timm17a, hccsa.1, cox17, stoml2, hsd17b10, slc25a39, dap3, hspa9, phb, lonp1*	5.90E-10	5.09E-8
5	GOTERM_CC_FAT	GO:0031090~organelle membrane	75	*scdb, cav1, waslb, sqlea, ostc, alg8, si:ch73-209e20.3, mcl1a, tmed2, rrbp1b, dhcr7, nucb2a, hmgcra, slc25a32a, cyp51, dpm3, man1a1, sdhb, LMAN2L (2 of 2), hsp90b1, abcb8, hspd1, cope, slc25a48, nsdhl, srpr, cox10, samm50l, sec11a, UQCR10, slc25a43, timm10, cyp3a65, SYCN (2 of 3), timm44, timm8b, insb, mrpl19, hspa5, cyp24a1, DNAJC11 (2 of 2), tomm40, WIPI1 (1 of 2), cyp7a1a, timm13, rer1, copb1, sec61al2, lmnb1, mgst1.1, sec31a, phb2a, mpdu1a, TIMM22, STT3B, agk, cyb5b, atp2a2a, nomo, timm17a, elovl6, tram1, edem1, hccsa.1, sigmar1, stoml2, msmo1, ldlrb, hsd17b10, slc25a39, hsd17b7, dhcr24, ubqln4, phb, alg12*	8.75E-10	6.04E-8
6	GOTERM_CC_FAT	GO:0042175~nuclear envelope-endoplasmic reticulum network	32	*hspa5, sqlea, ostc, alg8, dhcr7, rrbp1b, cyp7a1a, nucb2a, hmgcra, sec61al2, cyp51, dpm3, mpdu1a, STT3B, LMAN2L (2 of 2), hsp90b1, atp2a2a, nomo, elovl6, tram1, edem1, srpr, nsdhl, sigmar1, msmo1, sec11a, cyp3a65, hsd17b7, dhcr24, ubqln4, insb, alg12*	3.37E-9	1.94E-7

7	GOTERM_CC_FAT	GO:0005789~endoplasmic reticulum membrane	31	*hspa5, sqlea, ostc, alg8, rrbp1b, dhcr7, cyp7a1a, hmgcra, sec61al2, cyp51, dpm3, mpdu1a, STT3B, LMAN2L (2 of 2), hsp90b1, atp2a2a, nomo, elovl6, tram1, edem1, srpr, nsdhl, sigmar1, msmo1, sec11a, cyp3a65, hsd17b7, dhcr24, ubqln4, insb, alg12*	3.67E-9	1.81E-7
8	GOTERM_CC_FAT	GO:0012505~endomembrane system	54	*scdb, cav1, waslb, ostc, sqlea, alg8, icn, tmed2, dhcr7, rrbp1b, nucb2a, hmgcra, cyp51, dpm3, man1a1, ipo4, LMAN2L (2 of 2), hsp90b1, xpot, hspd1, nup50, cope, nsdhl, srpr, sec11a, cyp3a65, SYCN (2 of 3), sec13, insb, mrpl19, hspa5, cyp7a1a, rer1, copb1, sec61al2, lmnb1, sec31a, mpdu1a, anxa11a, STT3B, atp2a2a, ddx19, nomo, elovl6, tram1, edem1, sigmar1, msmo1, ldlrb, hsd17b7, dhcr24, ubqln4, dnase1, alg12*	2.68E-7	1.15E-5
9	GOTERM_CC_FAT	GO:0005793~ER-Golgi intermediate compartment	11	*man1a1, fn1b, pdia6, ergic1, hspa5, nucb2a, tmed5, tmed9, uggt1, tmed2, mydgf*	5.43E-7	2.08E-5
10	GOTERM_CC_FAT	GO:0031967~organelle envelope	45	*mrpl19, scdb, icn, tomm40, DNAJC11 (2 of 2), cyp24a1, mcl1a, si:ch73-209e20.3, dhcr7, timm13, nucb2a, slc25a32a, lmnb1, mgst1.1, phb2a, TIMM22, anxa11a, ipo4, sdhb, CHCHD4 (2 of 2), agk, cyb5b, ddx19, timm17a, abcb8, nup50, hspd1, xpot, slc25a48, sigmar1, hccsa.1, stoml2, cox17, hsd17b10, cox10, samm50l, slc25a39, UQCR10, slc25a43, timm10, timm44, dnase1, timm8b, phb, sec13*	8.93E-7	3.08E-5
11	GOTERM_CC_FAT	GO:0031975~envelope	45	*mrpl19, scdb, icn, tomm40, DNAJC11 (2 of 2), cyp24a1, mcl1a, si:ch73-209e20.3, dhcr7, timm13, nucb2a, slc25a32a, lmnb1, mgst1.1, phb2a, TIMM22, anxa11a, ipo4, sdhb, CHCHD4 (2 of 2), agk, cyb5b, ddx19, timm17a, abcb8, nup50, hspd1, xpot, slc25a48, sigmar1, hccsa.1, stoml2, cox17, hsd17b10, cox10, samm50l, slc25a39, UQCR10, slc25a43, timm10, timm44, dnase1, timm8b, phb, sec13*	9.92E-7	3.11E-5
12	GOTERM_CC_FAT	GO:0005788~endoplasmic reticulum lumen	14	*pdia2, pdia6, hspa5, hsp90b1, dnajb11, fkbp14, sdf2l1, hyou1, lrpap1, SIL1, pdia4, rcn3, uggt1, insb*	1.65E-6	4.76E-5
13	GOTERM_CC_FAT	GO:0005759~mitochondrial matrix	23	*pdk2a, qars, mrps16, lrrc59, hspd1, idh3a, mrpl41, mrpl35, si:ch73-209e20.3, mrpl43, hspe1, dap3, si:ch211-149b19.3, mrpl18, mrps18c, tufm, mrpl1, grpel1, dlat, timm44, hspa9, lonp1, aco2*	4.94E-6	1.31E-4

14	GOTERM_CC_FAT	GO:0031980~mitochondrial lumen	23	*pdk2a, qars, mrps16, lrrc59, hspd1, idh3a, mrpl41, mrpl35, si:ch73-209e20.3, mrpl43, hspe1, dap3, si:ch211-149b19.3, mrpl18, mrps18c, tufm, mrpl1, grpel1, dlat, timm44, hspa9, lonp1, aco2*	4.94E-6	1.31E-4
15	GOTERM_CC_FAT	GO:0005740~mitochondrial envelope	31	*tomm40, cyp24a1, DNAJC11 (2 of 2), si:ch73-209e20.3, mcl1a, timm13, slc25a32a, mgst1.1, phb2a, TIMM22, CHCHD4 (2 of 2), sdhb, agk, cyb5b, timm17a, abcb8, hspd1, slc25a48, hccsa.1, stoml2, cox17, hsd17b10, cox10, slc25a39, samm50l, UQCR10, timm10, slc25a43, timm44, phb, timm8b*	4.42E-5	1.09E-3
16	GOTERM_CC_FAT	GO:0031966~mitochondrial membrane	29	*tomm40, cyp24a1, DNAJC11 (2 of 2), si:ch73-209e20.3, mcl1a, timm13, slc25a32a, mgst1.1, phb2a, TIMM22, sdhb, agk, cyb5b, timm17a, abcb8, hspd1, slc25a48, hccsa.1, stoml2, cox10, hsd17b10, slc25a39, samm50l, timm10, slc25a43, UQCR10, timm44, phb, timm8b*	9.16E-5	2.10E-3
17	GOTERM_CC_FAT	GO:0005792~microsome	21	*ebp, sqlea, atp2a2a, cyb5b, hsp90b1, pld1a, lrrc59, acsbg2, tmed2, hsd11b2, cyp7a1a, sec11a, hmgcra, mgst2, cyp3a65, aqp8a.1, hsd17b3, mgst1.1, cyp51, slc35b1, lss*	9.63E-5	2.08E-3
18	GOTERM_CC_FAT	GO:0042598~vesicular fraction	21	*ebp, sqlea, atp2a2a, cyb5b, hsp90b1, pld1a, lrrc59, acsbg2, tmed2, hsd11b2, cyp7a1a, sec11a, hmgcra, mgst2, cyp3a65, aqp8a.1, hsd17b3, mgst1.1, cyp51, slc35b1, lss*	1.45E-4	2.93E-3
19	GOTERM_CC_FAT	GO:0019866~organelle inner membrane	25	*DNAJC11 (2 of 2), si:ch73-209e20.3, timm13, slc25a32a, lmnb1, mgst1.1, phb2a, TIMM22, sdhb, cyb5b, timm17a, abcb8, hspd1, slc25a48, sigmar1, hccsa.1, stoml2, hsd17b10, slc25a39, timm10, slc25a43, UQCR10, timm44, timm8b, phb*	1.97E-4	3.76E-3
20	GOTERM_CC_FAT	GO:0005743~mitochondrial inner membrane	23	*sdhb, cyb5b, timm17a, abcb8, hspd1, DNAJC11 (2 of 2), si:ch73-209e20.3, slc25a48, hccsa.1, stoml2, hsd17b10, slc25a39, timm13, UQCR10, slc25a43, timm10, slc25a32a, timm44, mgst1.1, phb, timm8b, phb2a, TIMM22*	4.35E-4	7.87E-3
21	GOTERM_CC_FAT	GO:0005744~mitochondrial inner membrane presequence translocase complex	5	*timm13, timm10, timm17a, timm44, timm8b*	4.45E-4	7.64E-3

Table S4: Complete list of altered Gene Ontologies for biological processes, molecular function and cellular components for up-regulated differentially expressed genes in F_1 low 1-C male livers compared to control. The significant GO terms with altered number of genes, gene name, p-value and Benjamini value are listed.

Biological process			Genes	Gene ID	P-value	Benjamini
1	GOTERM_BP_FAT	GO:0006952~defense response	14	*pld1b, ncf1, cd74a, hck, elf3, ccl19b, fn1a, itgb2, mxd, mst1ra, BX323596.2, ciita, cotl1, ins*	0.0020	0.8803
2	GOTERM_BP_FAT	GO:0006955~immune response	14	*zap70, ncf1, cd74a, si:busm1-48c11.3, ighv1-4, si:zfos-2070c2.4, endou, ccl19b, tcf7, CABZ01068208.1, si:dkeyp-9d4.2, BX323596.2, ciita, anxa11b*	0.0053	0.9414
3	GOTERM_BP_FAT	GO:0042127~regulation of cell proliferation	15	*zap70, gnai2b, tbx2b, fynrk, icn2, gucy2c, sox9b, hcls1, fgfr4, id2a, zbtb16a, mst1ra, wfdc1, ins, lamb1b*	0.0063	0.8950
4	GOTERM_BP_FAT	GO:0008284~positive regulation of cell proliferation	10	*zap70, gnai2b, fgfr4, tbx2b, icn2, id2a, mst1ra, hcls1, lamb1b, ins*	0.0083	0.8904
5	GOTERM_BP_FAT	GO:0006935~chemotaxis	6	*pld1b, cxcr3.1, itgb2, BX323596.2, lect2l, ccl19b*	0.0114	0.9124
6	GOTERM_BP_FAT	GO:0042330~taxis	6	*pld1b, cxcr3.1, itgb2, BX323596.2, lect2l, ccl19b*	0.0114	0.9124
7	GOTERM_BP_FAT	GO:0016042~lipid catabolic process	6	*cel.1, cyp7a1a, pld1b, neu3.3, lipia, dagla*	0.0155	0.9375

Molecular function			Genes	Gene ID	P-value	Benjamini
1	GOTERM_MF_FAT	GO:0004713~protein tyrosine kinase activity	8	*zap70, fgfr4, fynrk, ros1, gucy2c, epha4l, hck, mst1ra*	7.905E-4	0.2087
2	GOTERM_MF_FAT	GO:0004181~metallocarboxypeptidase activity	4	*cpa4, cpb1, cpa5, cpa2*	0.0015	0.1938

Cellular component			Genes	Gene ID	P-value	Benjamini
1	GOTERM_CC_FAT	GO:0015629~actin cytoskeleton	9	*micall2b, wasla, aif1l, actc1b, acta2, cgnl1, mst1ra, myl9a, wipf1a*	0.0021	0.3059
2	GOTERM_CC_FAT	GO:0005886~plasma membrane	45	*itgb2, amotl2a, ros1, MTHFS, neu3.3, flrt3, magi1a, lamb1b, dagla, apbb1ip, rdh8a, zap70, slc26a3.1, CABZ01079192.1, gnai2b, noxo1a, cd74a, cgnl1, ncf1, si:busm1-48c11.3, rtn4rl1a, si:zfos-2070c2.4, cdh17, jam3b, slc6a19b, fgfr4, gcgrb, sorl1, cxcr3.1, rims1a, mst1ra, zbtb16a, csf2rb, sgk1*	0.0100	0.4392

Table S5: Enriched KEGG pathways of down-regulated and up-regulated differentially expressed genes in F1 male livers of low 1-C offspring compared to control offspring. For each pathway term the number of genes, gene ID, p-value and Benjamini value are given. All pathways with significant p-values are listed.

Down-regulated KEGG pathways	Genes	Gene ID	P-value	Benjamini
1: hsa00100:Steroid biosynthesis	11	*dhcr7, ebp, sqlea, lipf, hsd17b7, dhcr24, cyp51, nsdhl, lss, msmo1, sc5d*	3.99E-12	4.35E-10
2: hsa00900:Terpenoid backbone biosynthesis	8	*mvk, fdps, pdss2, hmgcra, ubqln4, hmgcs1, acat2, idi1*	6.72E-8	3.66E-6
3: hsa03040:Spliceosome	13	*usp39, sf3b4, snrpd1, LSM3, snrpd2, sf3b5, prp19, magoh, snrpg, prpf40a, lsm6, snrpd2, snrpe*	2.05E-4	0.0074
4: hsa00260:Glycine, serine and threonine metabolism	6	*cbsb, SRR (1 of 2), dao.3, amt, cth, pipox*	0.00159	0.0425
5: hsa00970:Aminoacyl-tRNA biosynthesis	6	*yars, vars, larsb, quars, cars, WARS (1 of 2)*	0.00560	0.11525
6: hsa00520:Amino sugar and nucleotide sugar metabolism	6	*pmm2, pgm2, ugdh, galk3, gale, pgm3*	0.00759	0.12919
7: hsa00510:N-Glycan biosynthesis	6	*man1a1, alg8, mogs, dpm3, STT3B, alg12*	0.00915	0.13340
8: hsa03050:Proteasome	6	*psmd14, psmc6, psmd7, psma5, psma3, psmd11b*	0.01001	0.12814
9: hsa00270:Cysteine and methionine metabolism	5	*cbsb, srm, mtr, cth, idhba*	0.01479	0.16512
10: hsa00910:Nitrogen metabolism	4	*glula, amt, cth, asns*	0.02602	0.24973
11: hsa00030:Pentose phosphate pathway	4	*tktb, pgd, pgm2, dera*	0.03243	0.27870
12: hsa00290:Valine, leucine and isoleucine biosynthesis	3	*vars, larsb, bcat1*	0.03705	0.29029
13: hsa00140:Steroid hormone biosynthesis	5	*cyp3c4, hsd17b7, hsd17b12b, hsd11b2*	0.04016	0.29085

Up-regulated KEGG pathways	Genes	Gene ID	P-value	Benjamini
1: hsa04062:Chemokine signaling pathway	7	*wasl1, gnai2b, cxcr3.1, ncf1, BX323596.2, hck, ccl19b*	0.01038	0.55678
2: hsa04670:Leukocyte transendothelial migration	5	*gnai2b, itgb2, ncf1, myl9a, jam3b*	0.02980	0.69270
3: hsa04530:Tight junction	5	*gnai2b, myl9a, jam3b, hcls1, magi1a*	0.04440	0.58757
4: hsa04612:Antigen processing and presentation	4	*cd74a, si:busm1-48c11.3, si:zfos-2070c2.4, ciita*	0.04986	0.54971

Table S6: RRBS sequencing summary for all 12 samples.

RRBS summary	positions	%
Total number of CpG positions:	23946935	
Total number of data points:	2995170	12.5% of total CpG positions
Total number of data points, with min coverage 10:	906808	30% of total data points
Total number of differential methylation estimates:	2702485	90% of total data points
Total number of differentially methylated positions (fdr < 0.05):	2869	0,3% of total data points

Table S7: RRBS sequencing summary for all differentially methylated loci comparing Low1-C and control samples.

DML class	transcript	exon	intron	promoter	intergenic	cpi	cpi.shore
class size[1]	1479878,00	256914,00	1222964,00	129426,00	290423,00	591579,00	354116,00
dml[2]	1609,00	224,00	1385,00	185,00	251,00	311,00	434,00
dml e[3]	1571,06	272,74	1298,32	137,40	308,32	628,03	375,94
p.under[4]	0,93	0,00	1,00	1,00	0,00	0,00	1,00
p.over[4]	0,08	1,00	0,00	0,00	1,00	1,00	0,00
dml.r[5]	1,02	0,82	1,07	1,35	0,81	0,50	1,15

[1] *class size:* the number of positions where we have measurements

[2] *dml:* the number of loci that are differentially methylated (of the given class) with an fdr of less than 0.05

[3] *dml e:* the number of loci that would expected to have an fdr of less than 0.05 in a random sample of the same size as the respective class when drawn from the full set measurements. Calculated as the frequency of differential methylation multiplied by the class size.

[4] *p.under* and *p.over* refer to the probability of observing a smaller than or equal, or greater than or equal number of differentially methylated loci in a random sampling of measurements. *p.under and p.over* were calculated using the hypergeometric distribution.

[5] *dml.r:* The enrichment ratio (observed / expected) of differential methylation.

Table S8: Correlation between RRBS DMR/DML and RNA seq DEG

			Methylation	Gene regulation
**Zebrafish gene name**	**RRBS plot page**	**Position of DMR/DML**	**Low 1C/ control**	**Low 1C/ control**
sdhb	Figure S12, page 2	DMR 20000 bp downstream	hypermethylated	downregulated
prps1a	Figure S12, page 16	DMR 40000 bp downstream	hypomethylated	downregulated
U1	Figure S12, page 25	DMR repetetive sequence	hypomethylated	downregulated
yars	Figure S12, page 29	DMR 20000 bp upstream	hypomethylated	downregulated
slc5a1	Figure S12, page 36	DMR in intron and in fibina gene	hypermethylated	upregulated
gfra3	Figure S12, page 38	DMR in intron	hypomethylated	downregulated
abcd3b	Figure S12, page 43	DMR 30000 bp downstream	hypomethylated	downregulated
psmc6	Figure S12, page 71	DMR 40000 bp downstream	hypermethylated	downregulated
thop1	Figure S12, page 95	DMR in intron	hypermethylated	downregulated
gcgrb	Figure S13, page 7	DML 35000 bp downstream	hypomethylated	upregulated
flnbl	Figure S13, page 89	DML first exon	hypomethylated	downregulated
mtmr4	Figure S14, page 35	DML 1000 bp downstream	hypermethylated	downregulated

Table S9: Commands and scripts RNA sequencing data analysis

RNA sequencing:

Mapping of reads to the genome using STAR version 020201:

```
STAR  --outFilterMultimapNmax 5 \
      --outFilterMismatchNoverLmax 0.05 \
      --outFilterIntronMotifs RemoveNoncanonicalUnannotated \
      --genomeDir ~/apps/STAR/STAR/genomeDir/Zv10/unmasked/ \
      --readFilesIn <fastq_file> --runThreadN 6 \
      --outSAMtype BAM SortedByCoordinate
```

Where the index used was created from an unmasked version of the Zv10 assembly, indicates the fastq file containing the reads.

Expression values were estimated using cufflinks v2.2.1 using the command:

```
cufflinks -G Danio_rerio.GRCz10.82.gtf -M mask_file.gtf \
          --multi-read-correct --library-type fr-firststrand -p 12\
          Aligned.sortedByCoord.out.bam
```

Where the main gtf file was obtained from Ensembl, the mask_file.gtf contained entries for ribosomal and pseudo genes and the bam file was created by the STAR program.

Differential expression was estimated using the cuffdiff program using:

```
cuffdiff --labels low1C,ctl --library-type fr-firststrand -p 6 \
         --multi-read-correct -o liver_cuffdiff \
         Danio_rerio.GRCz10.82.gtf \
RZY-319/Aligned.sortedByCoord.out.bam,\
RZY-320/Aligned.sortedByCoord.out.bam,\
RZY-321/Aligned.sortedByCoord.out.bam,\
RZY-322/Aligned.sortedByCoord.out.bam \
RZY-323/Aligned.sortedByCoord.out.bam,\
RZY-324/Aligned.sortedByCoord.out.bam,\
RZY-325/Aligned.sortedByCoord.out.bam,\
RZY-326/Aligned.sortedByCoord.out.bam
```

Where the bam files were produced using STAR as above.

Table S10: Commands and scripts RRBS data analysis

RRBS:

The 'detect_quality_code.py', and 'biseqMethCalling.py' scripts are described in: Klughammer, J. *et al.* Differential DNA Methylation Analysis without a Reference Genome. *Cell Rep* 13, 2621-2633, doi:10.1016/j.celrep.2015.11.024 (2015).

Commands are given without the specific locations of the executable scripts and with shell variables used to indicate file names. Comments are preceded by '#'.

```
## Adapter trimming with custom python script and trimmomatic
python -u detect_quality_code.py -f $input.fastq

java -Xmx16000m -jar trimmomatic-0.32-epignome.jar SE -phred33 -threads 4 \

     $input.fastq $input_trimmed.fq \ ILLUMINACLIP:/data/groups/lab_bock/shared/resources/adapters/\

RRBS_adapters.fa:2:40:7 SLIDINGWINDOW:4:15 MAXINFO:20:0.50 MINLEN:18

## Alignment with BSMAP
bsmap -a $input_trimmed.fq -d danRer10.fa -o $input_mapped.bam \
-D C-CGG -w 100 -v 0.08 -r 1 -p 4 -n 0 -s 12 -S 0 -f 5 -q 0 -u -V 2

## sorting and indexing with samtools
samtools sort -f $input_mapped.bam $input_mapped.bam
samtools index $input_mapped.bam

## Extract methylation levels using biseqMethCalling.py
python -u biseqMethCalling.py --sampleName=$input \
--alignmentFile=$input_mapped.bam --methodPrefix=RRBS --rrbsMode \
--checkRestriction --minFragmentLength=20 --maxFragmentLength=1000 \
--pfStatus=All --maxMismatches=0.1 --baseQualityScoreC=20 \
--baseQualityScoreNextToC=10 --laneSpecificStatistics --bigBedFormat \
--deleteTemp --toolsDir=/data/shared/resources/tools \
--outputDir=/results_pipeline/$input/biseq_danRer10 \
--webOutputDir=/scratch/$input/biseq_danRer10/web \
--tempDir=/scratch/$input/biseq_danRer10/temp --timeDelay=0 \
--genomeFraction=50 --smartWindows=250000 --maxProcesses=4 \
--genomeDir=/data/shared/resources/genomes_split \
--inGenome=danRer10 --outGenome=danRer10
```
