# Supplementary material for: Parental micronutrient deficiency distorts liver DNA methylation and expression of lipid genes associated with a fatty-liver-like phenotype in offspring
Source: Sci Rep. 2018 Feb 14;8:3055. doi: 10.1038/s41598-018-21211-5 (PMC5812986; doi:10.1038/s41598-018-21211-5)
Supplement: Supplementary file 2 — Figure S12 [file 41598_2018_21211_MOESM2_ESM.pdf]

Supplementary Info, figure S12:

Parental micronutrient deficiency distorts liver DNA methylation and expression of lipid genes associated with a fatty-liver-like phenotype in offspring

Kaja H. Skjærven[1*], Lars Martin Jakt[2], Jorge M.O. Fernandes[2], John Arne Dahl[3], Anne-Catrin Adam[1], Johanna Klughammer[4], Christoph Bock[4] and Marit Espe[1]

[1]*Institute of Marine Research, IMR, Norway*

[2]*Faculty of Biosciences and Aquaculture, Nord University, Norway*

[3]*Department of Microbiology, Oslo University Hospital, Norway*

[4]*CeMM Research Center for Molecular Medicine of the Austrian Academy of Sciences, 1090 Vienna, Austria*

[*]Corresponding author:

Dr. Kaja Helvik Skjærven

Institute of Marine Research (IMR),

PO Box 1870 Nordnes,

5817 Bergen, Norway

Tel.:+4741458362; fax: +4755905299.

E-mail address: ksk@hi.no

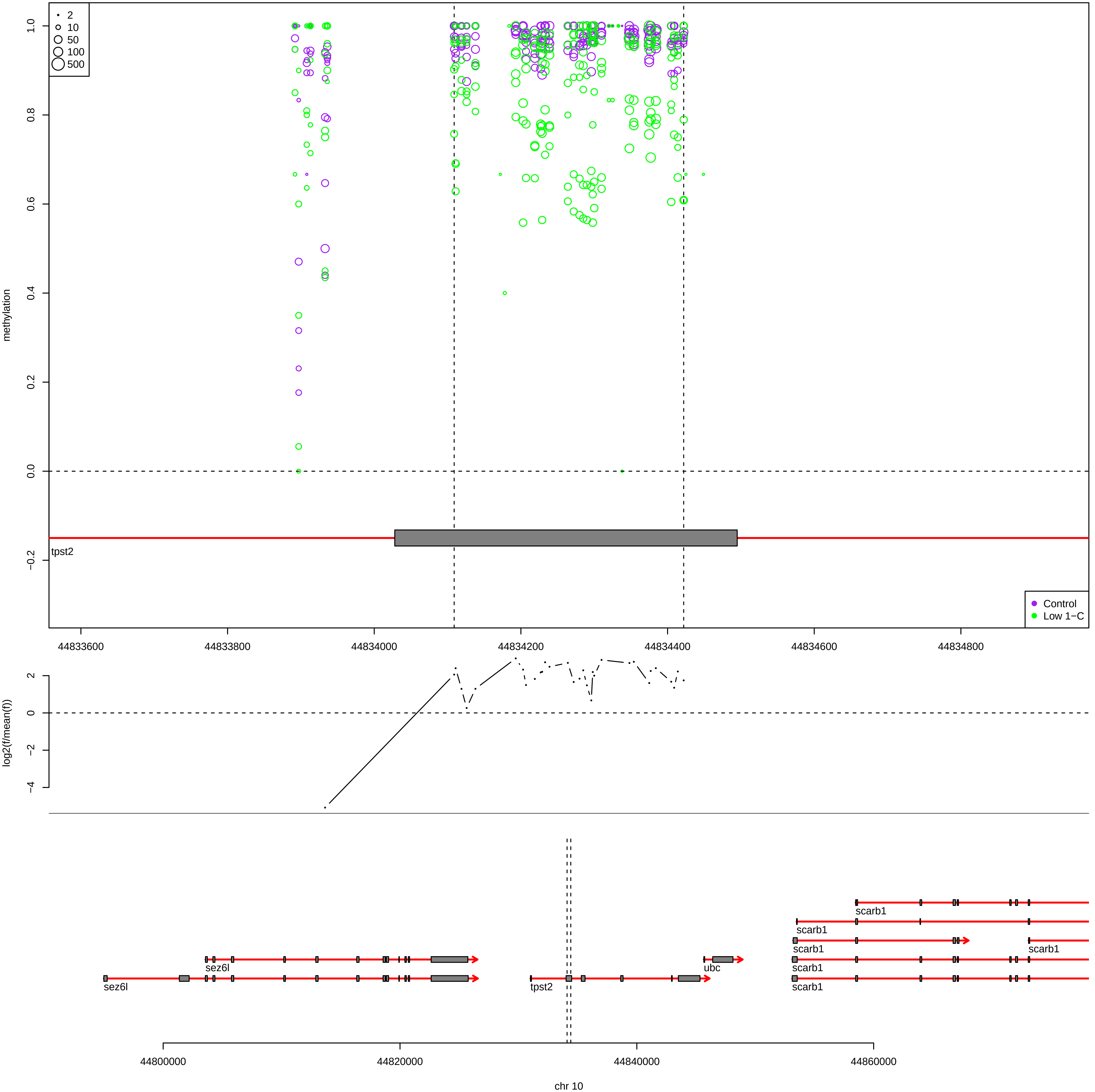

2
10
50
100
500
1.0
0.8
0.6
0.4
0.2
0.0
−0.2
methylation
tpst2
Control
Low 1−C
44833600
44833800
44834000
44834200
44834400
44834600
44834800
log2(f/mean(f))
2
0
−2
−4
scarb1
scarb1
scarb1
scarb1
scarb1
scarb1
sez6l
sez6l
tpst2
ubc
44800000
44820000
44840000
44860000
chr 10

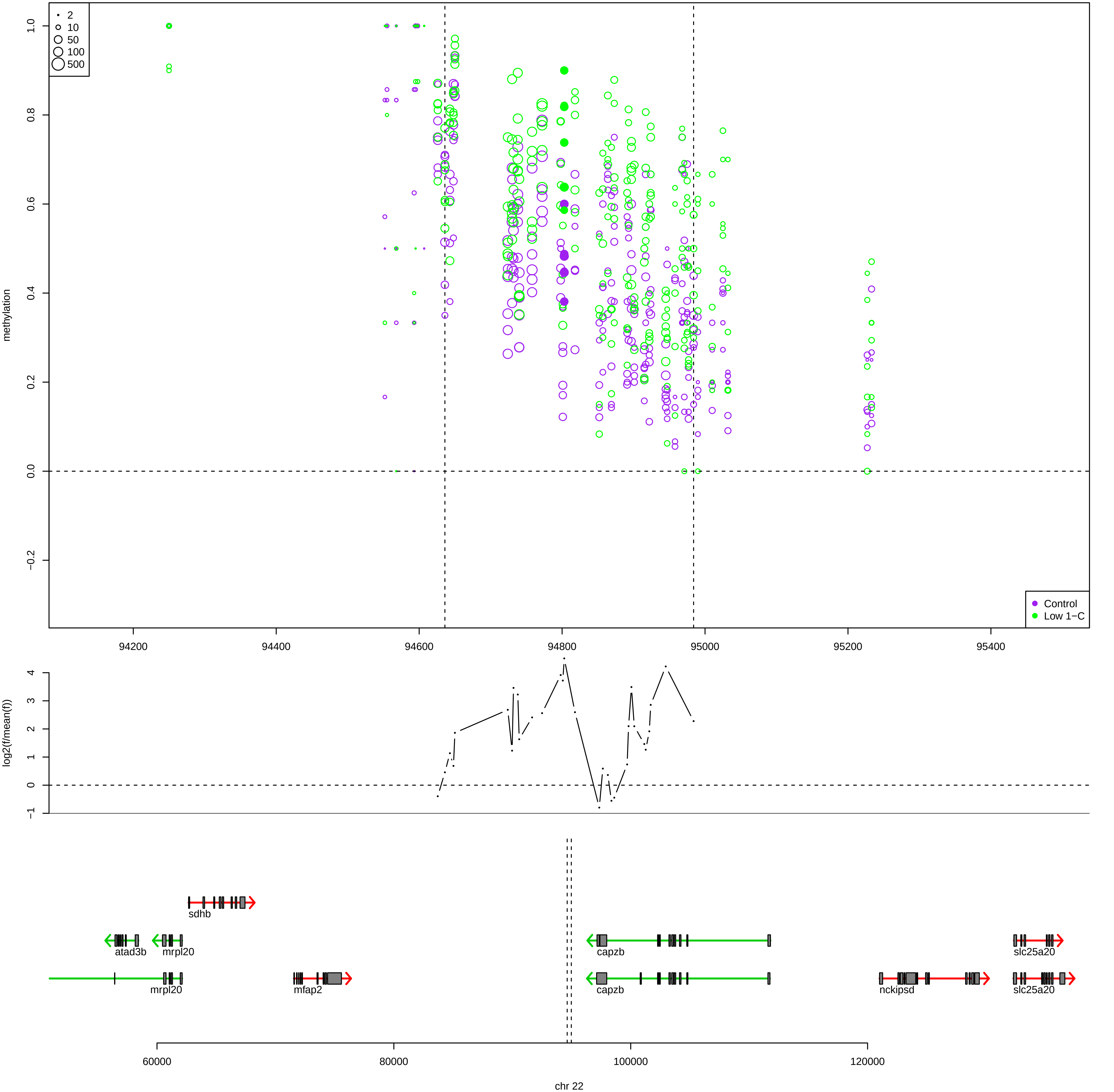

2
10
50
100
500
methylation
1.0
0.8
0.6
0.4
0.2
0.0
−0.2
Control
Low 1−C
94200
94400
94600
94800
95000
95200
95400
log2(f/mean(f))
4
3
2
1
0
−1
sdhb
atad3b
mrpl20
mrpl20
mfap2
capzb
capzb
nckipsd
slc25a20
slc25a20
60000
80000
100000
120000
chr 22

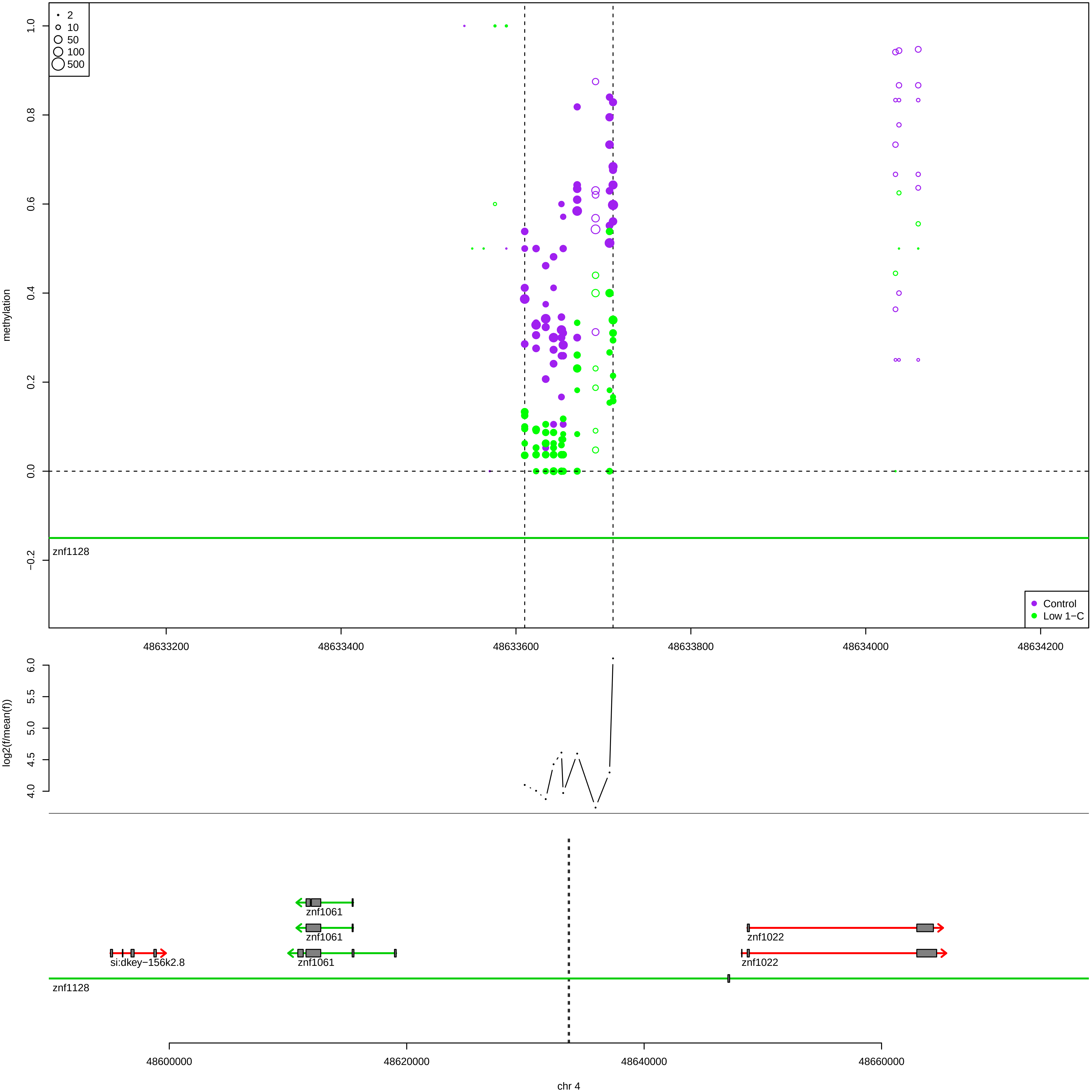

2
10
50
100
500
methylation
1.0
0.8
0.6
0.4
0.2
0.0
−0.2
znf1128
Control
Low 1−C
48633200
48633400
48633600
48633800
48634000
48634200
log2(f/mean(f))
6.0
5.5
5.0
4.5
4.0
znf1061
znf1061
znf1061
si:dkey−156k2.8
znf1022
znf1022
znf1128
48600000
48620000
48640000
48660000
chr 4

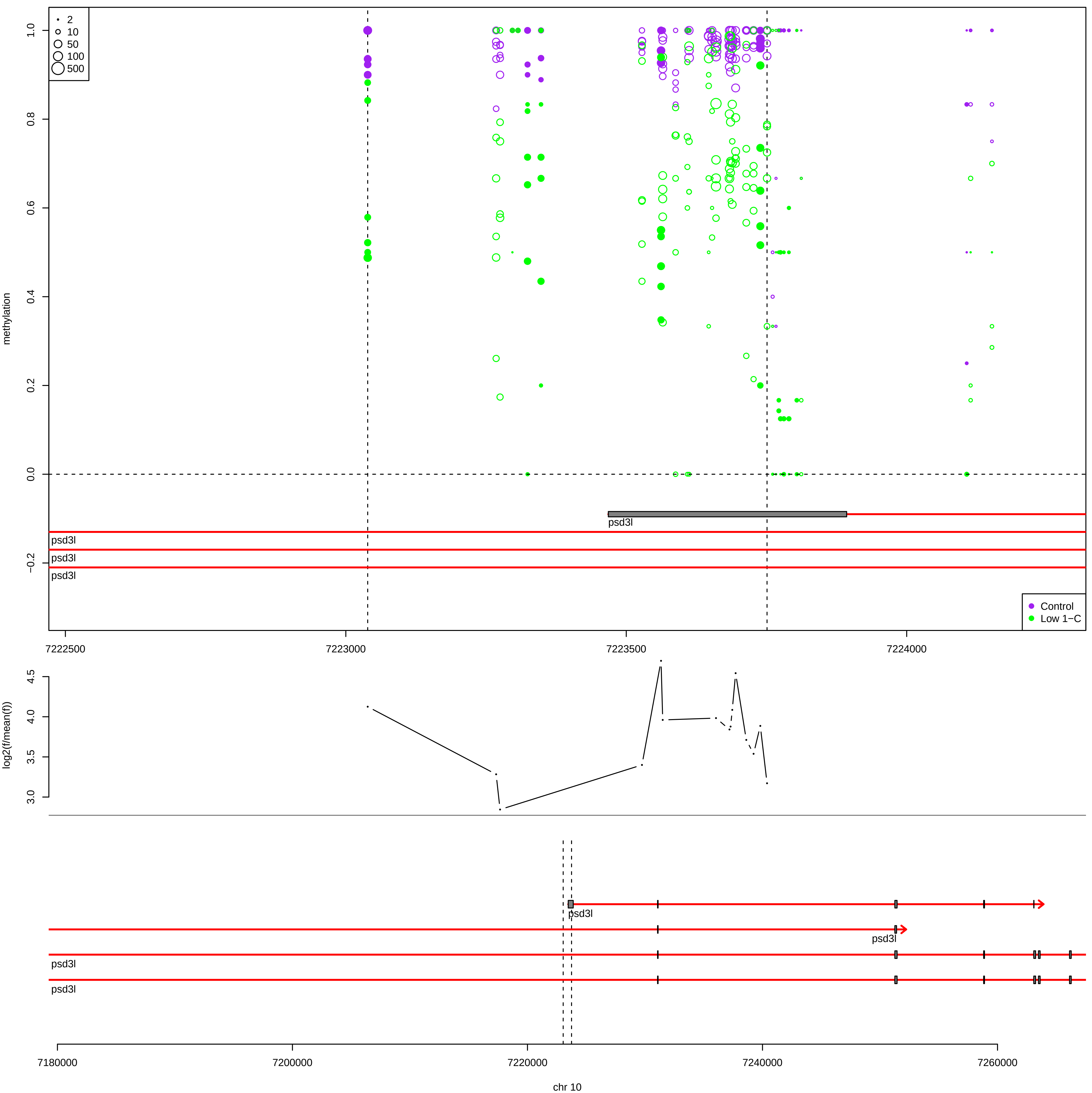

2
10
50
100
500
Control
Low 1−C
methylation
1.0
0.8
0.6
0.4
0.2
0.0
−0.2
psd3l
psd3l
psd3l
psd3l
7222500
7223000
7223500
7224000
log2(f/mean(f))
4.5
4.0
3.5
3.0
psd3l
psd3l
psd3l
psd3l
7180000
7200000
7220000
7240000
7260000
chr 10

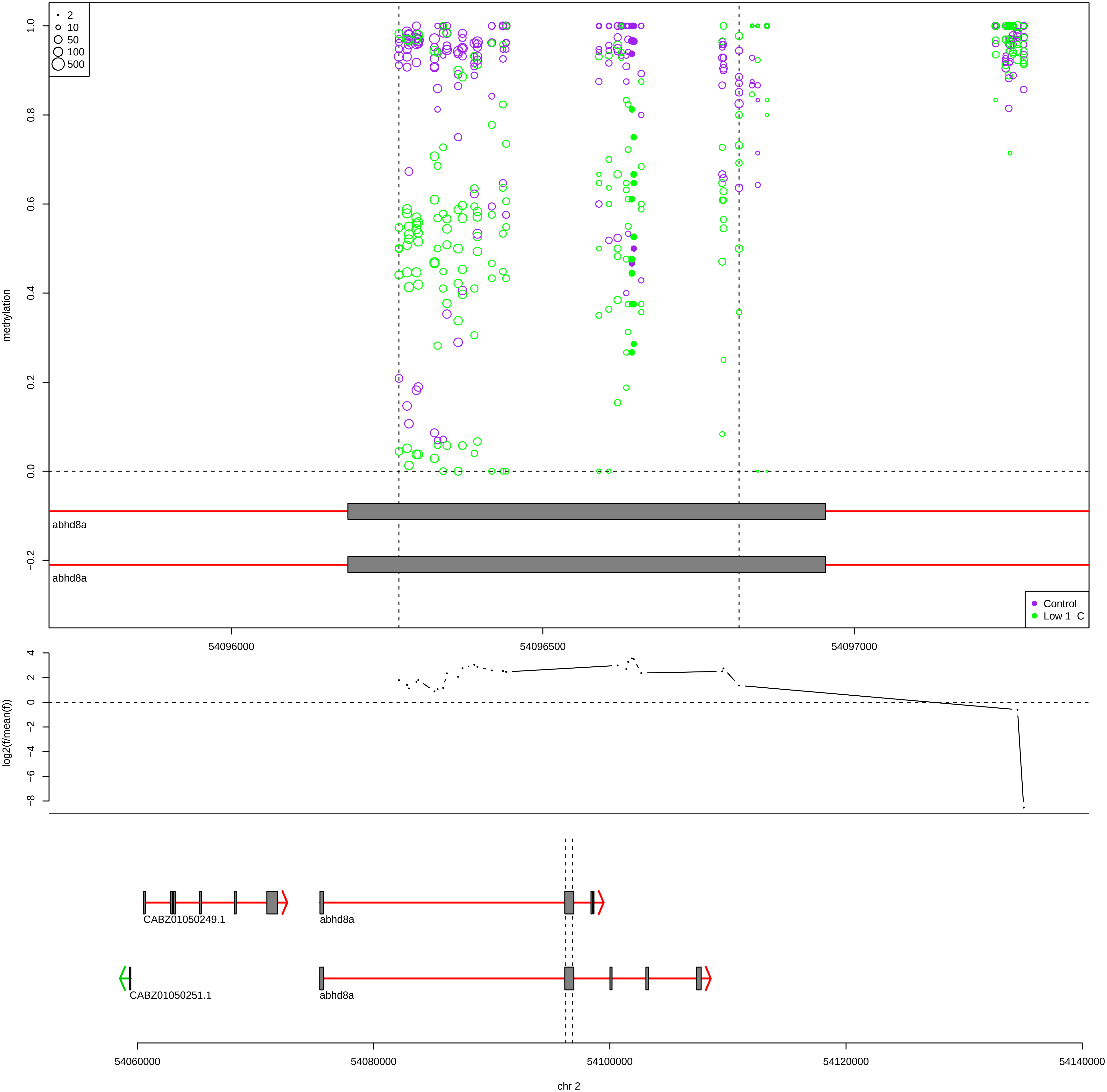
2
10
50
100
500
methylation
1.0
0.8
0.6
0.4
0.2
0.0
−0.2
abhd8a
abhd8a
Control
Low 1−C
54096000
54096500
54097000
log2(f/mean(f))
4
2
0
−2
−4
−6
−8
CABZ01050249.1
abhd8a
CABZ01050251.1
abhd8a
54060000
54080000
54100000
54120000
54140000
chr 2

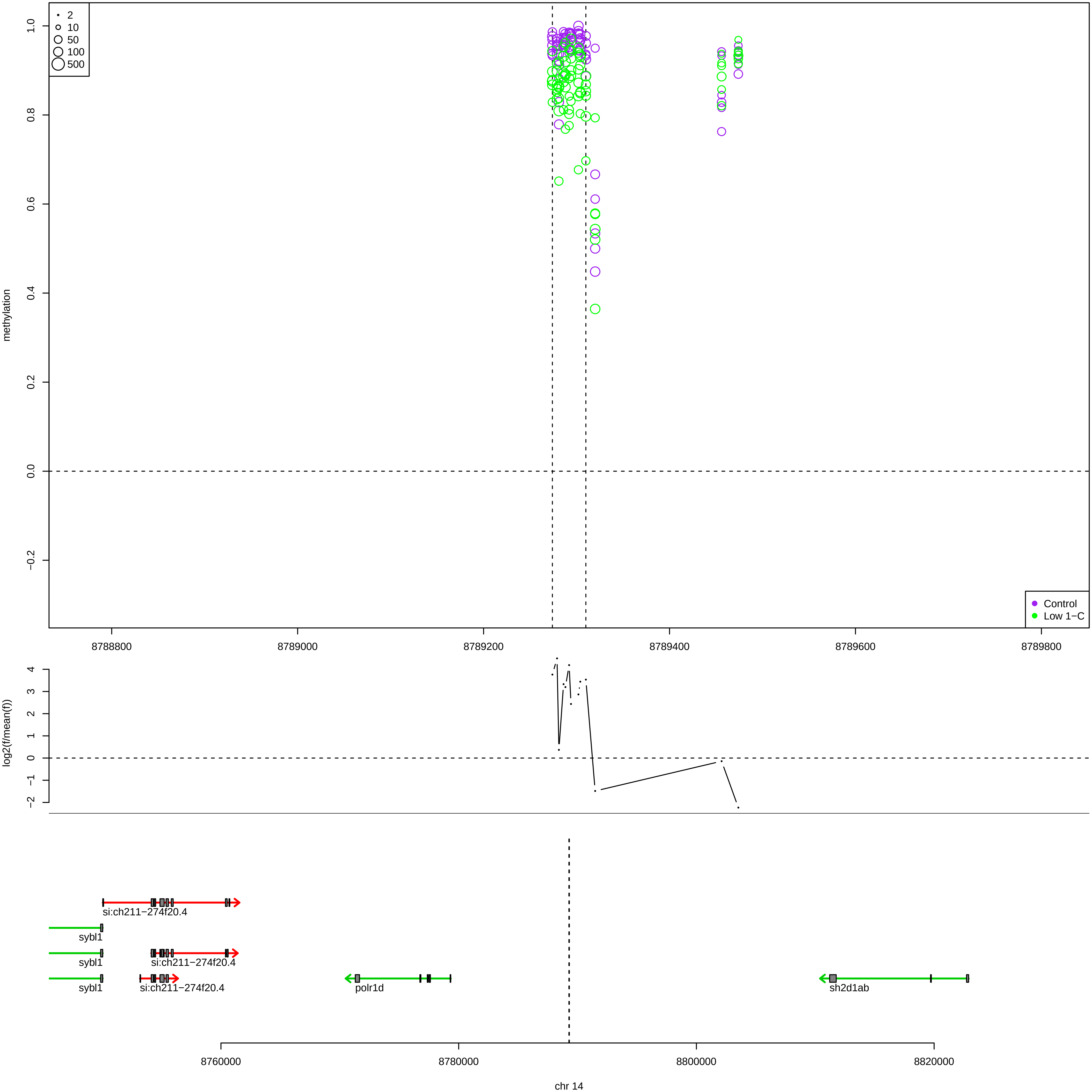

methylation
2
10
50
100
500
1.0
0.8
0.6
0.4
0.2
0.0
−0.2
Control
Low 1−C
8788800
8789000
8789200
8789400
8789600
8789800
log2(f/mean(f))
4
3
2
1
0
−1
−2
si:ch211−274f20.4
sybl1
sybl1
si:ch211−274f20.4
sybl1
si:ch211−274f20.4
polr1d
sh2d1ab
8760000
8780000
8800000
8820000
chr 14

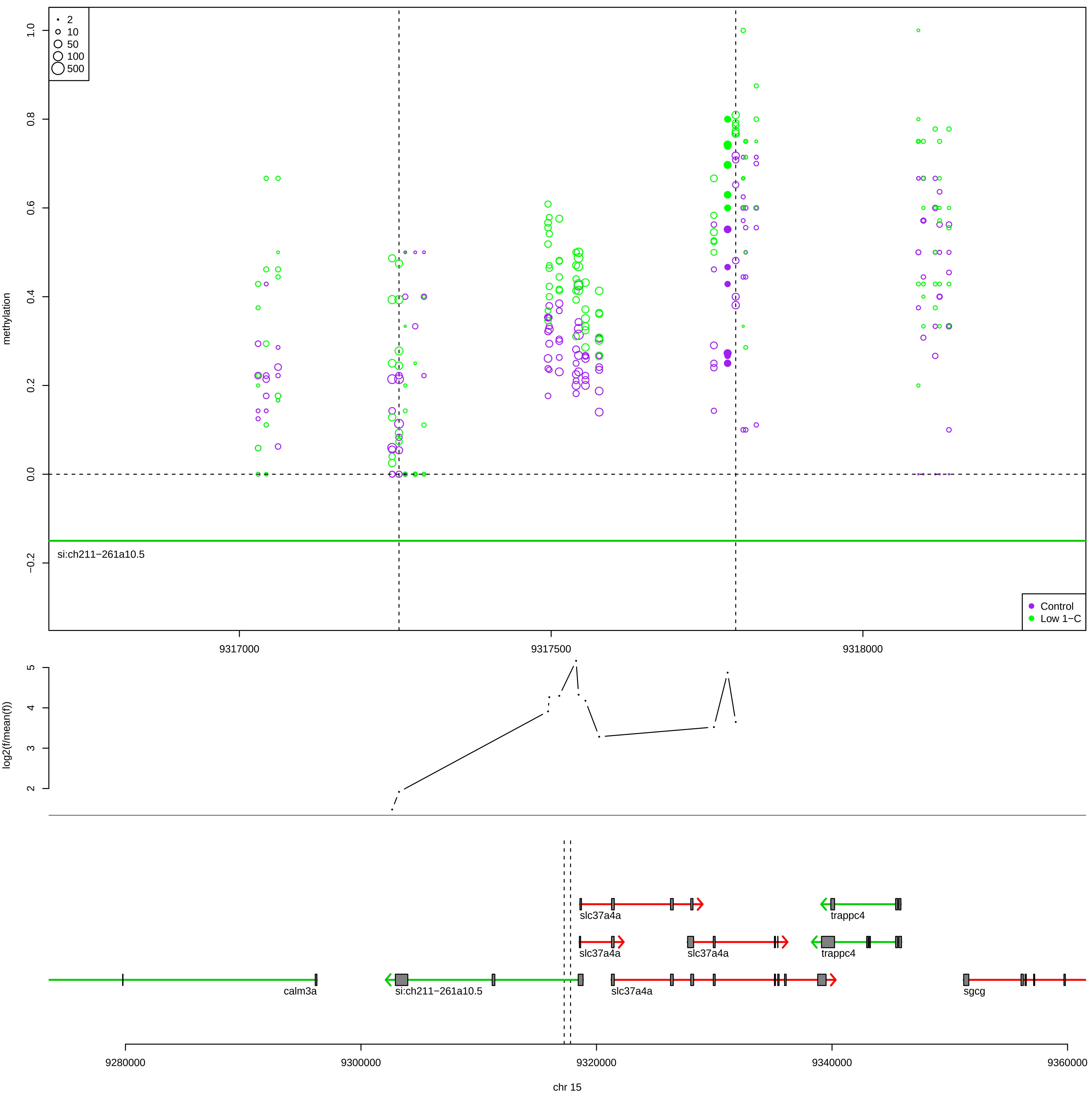

2
10
50
100
500
1.0
0.8
0.6
0.4
0.2
0.0
−0.2
methylation
si:ch211−261a10.5
Control
Low 1−C
9317000
9317500
9318000
log2(f/mean(f))
5
4
3
2
slc37a4a
trappc4
slc37a4a
slc37a4a
trappc4
calm3a
si:ch211−261a10.5
slc37a4a
sgcg
9280000
9300000
9320000
9340000
9360000
chr 15

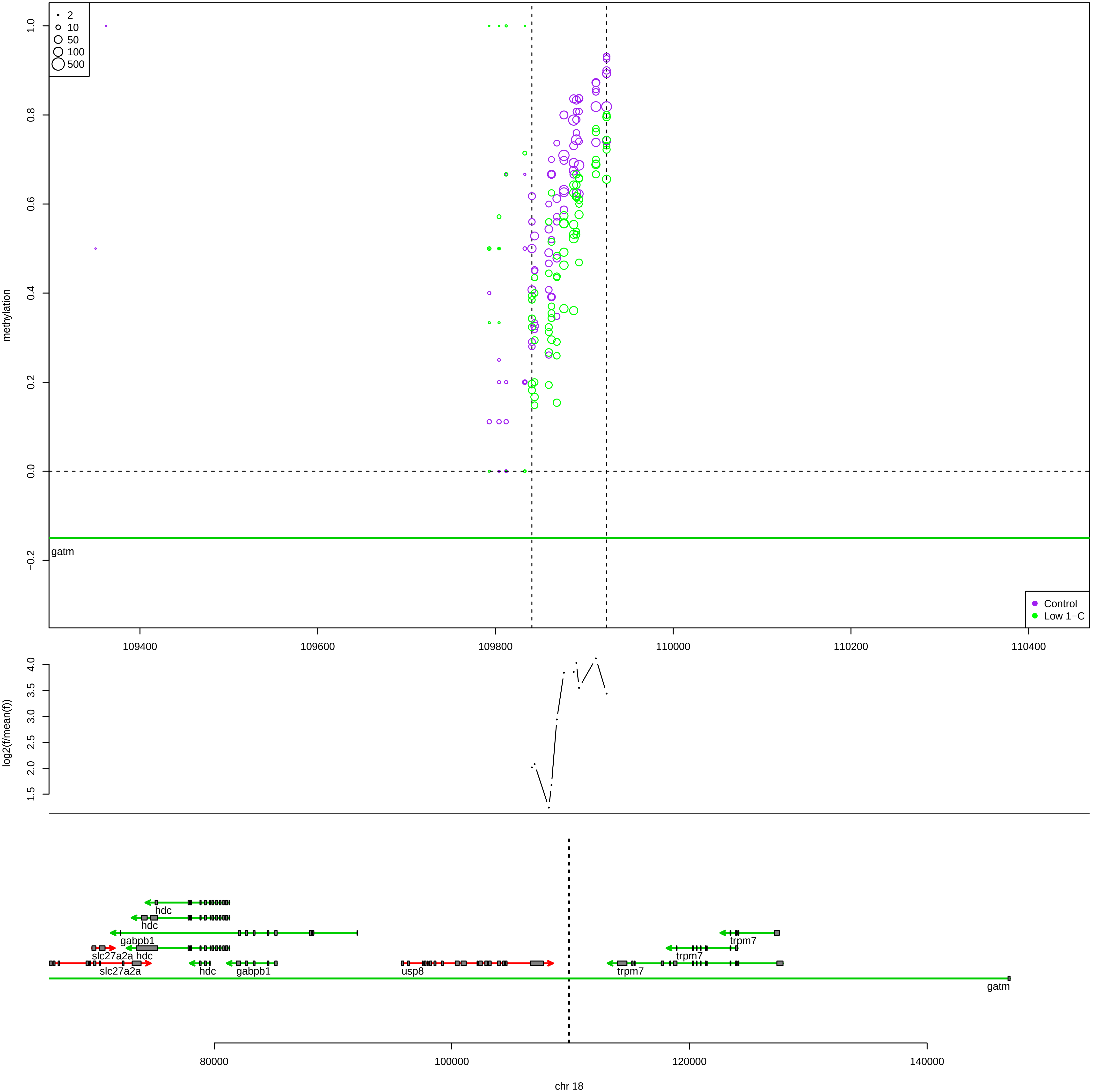

2
10
50
100
500
methylation
1.0
0.8
0.6
0.4
0.2
0.0
−0.2
gatm
Control
Low 1−C
109400
109600
109800
110000
110200
110400
log2(f/mean(f))
4.0
3.5
3.0
2.5
2.0
1.5
hdc
hdc
gabpb1
slc27a2a hdc
slc27a2a
hdc
gabpb1
usp8
trpm7
trpm7
trpm7
gatm
80000
100000
120000
140000
chr 18

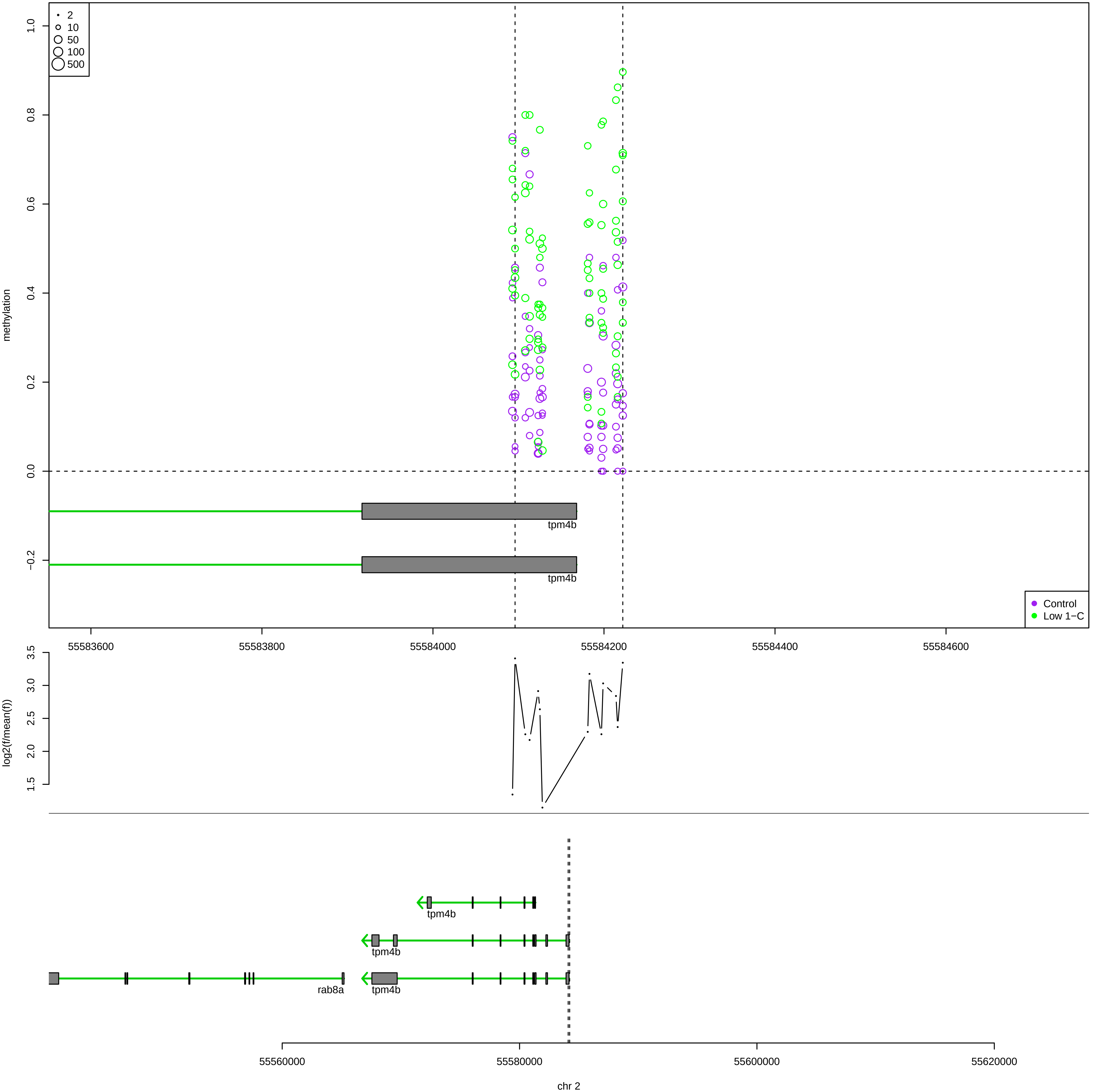
2
10
50
100
500
methylation
1.0
0.8
0.6
0.4
0.2
0.0
−0.2
tpm4b
tpm4b
Control
Low 1−C
55583600
55583800
55584000
55584200
55584400
55584600
log2(f/mean(f))
3.5
3.0
2.5
2.0
1.5
tpm4b
tpm4b
rab8a
tpm4b
55560000
55580000
55600000
55620000
chr 2

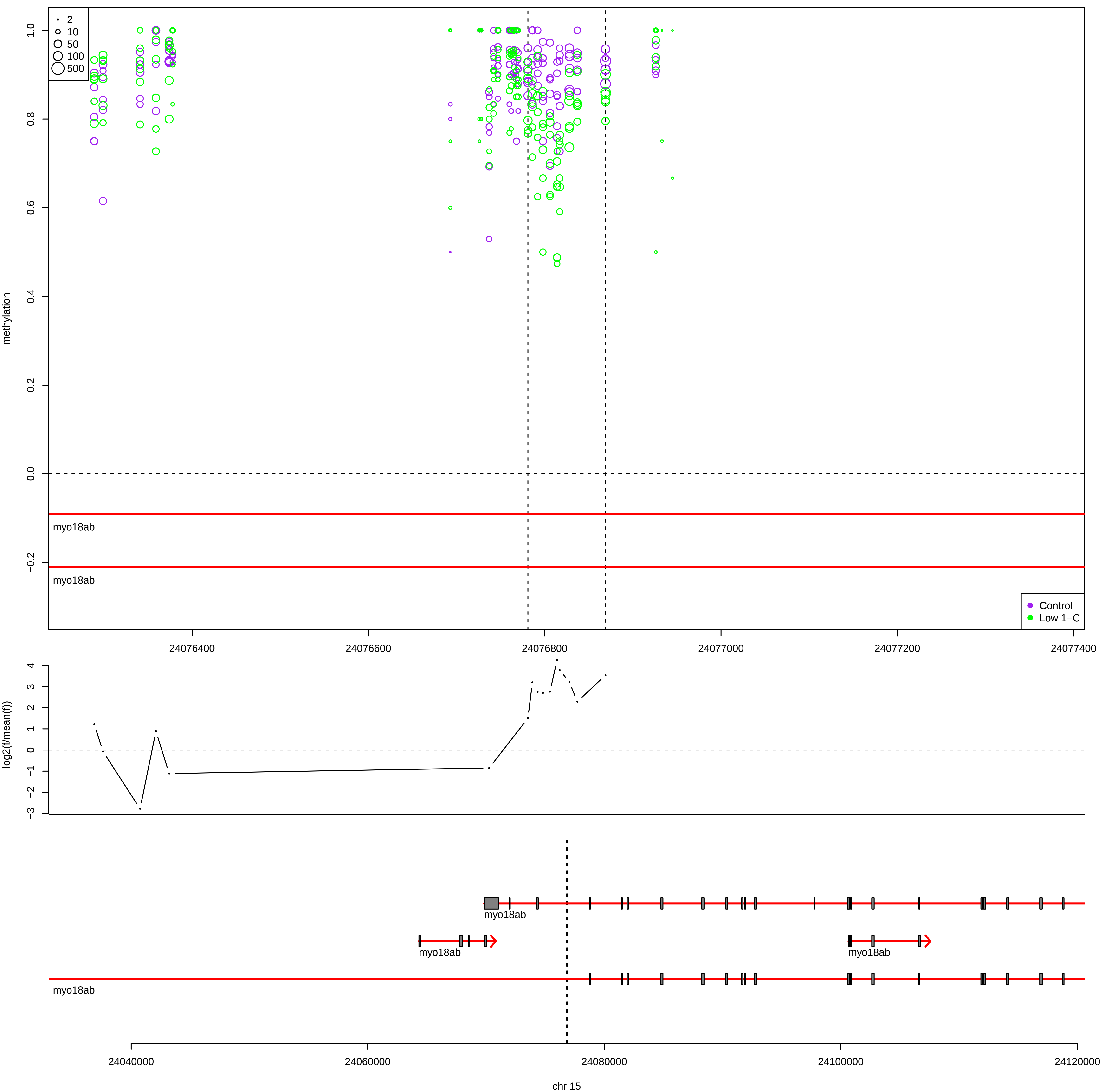

methylation
2
10
50
100
500
Control
Low 1−C
myo18ab
myo18ab
24076400
24076600
24076800
24077000
24077200
24077400
log2(f/mean(f))
myo18ab
myo18ab
myo18ab
myo18ab
24040000
24060000
24080000
24100000
24120000
chr 15

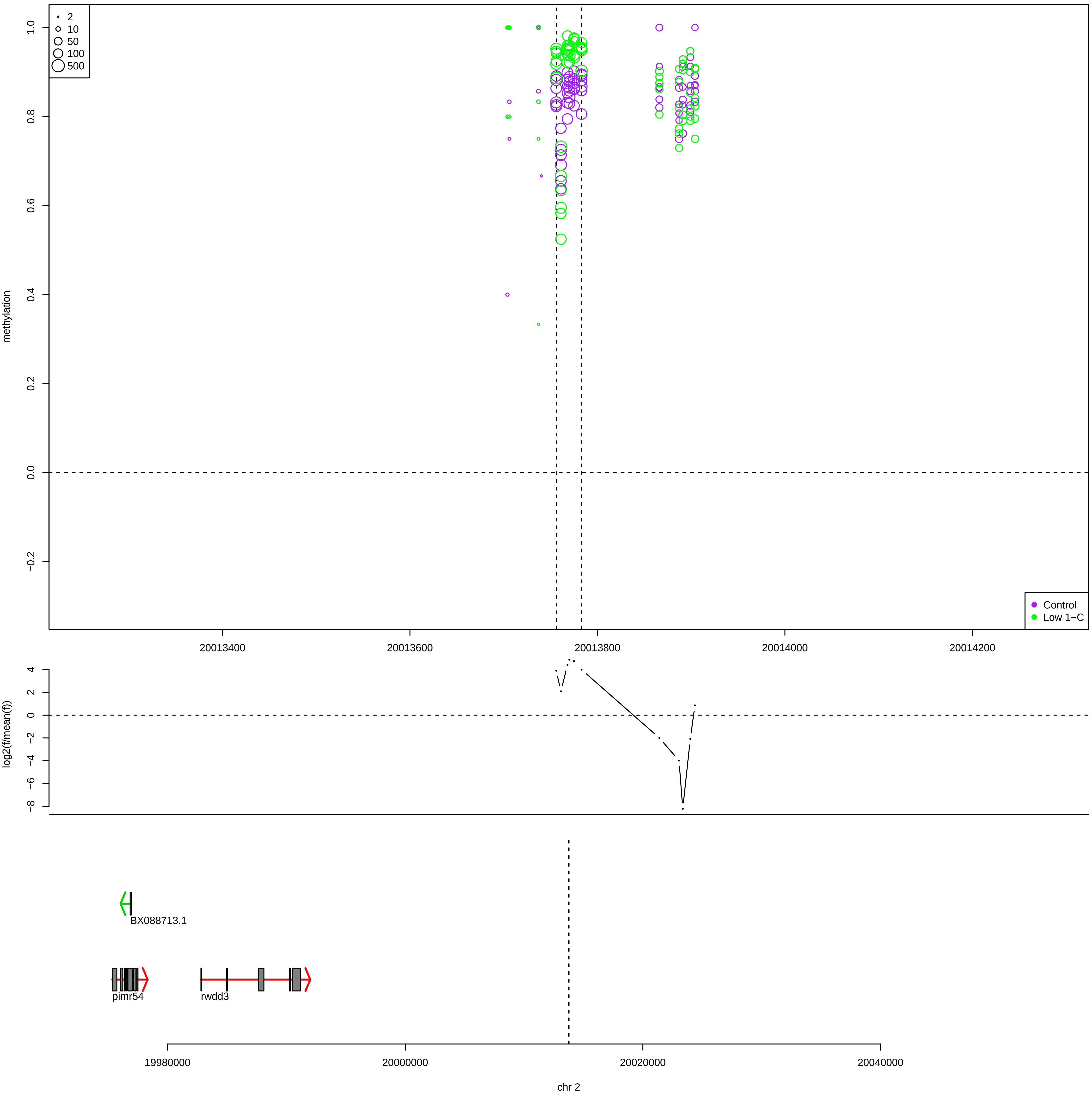

2
10
50
100
500
1.0
0.8
0.6
0.4
0.2
0.0
−0.2
methylation
Control
Low 1−C
20013400
20013600
20013800
20014000
20014200
4
2
0
−2
−4
−6
−8
log2(f/mean(f))
BX088713.1
pimr54
rwdd3
19980000
20000000
20020000
20040000
chr 2

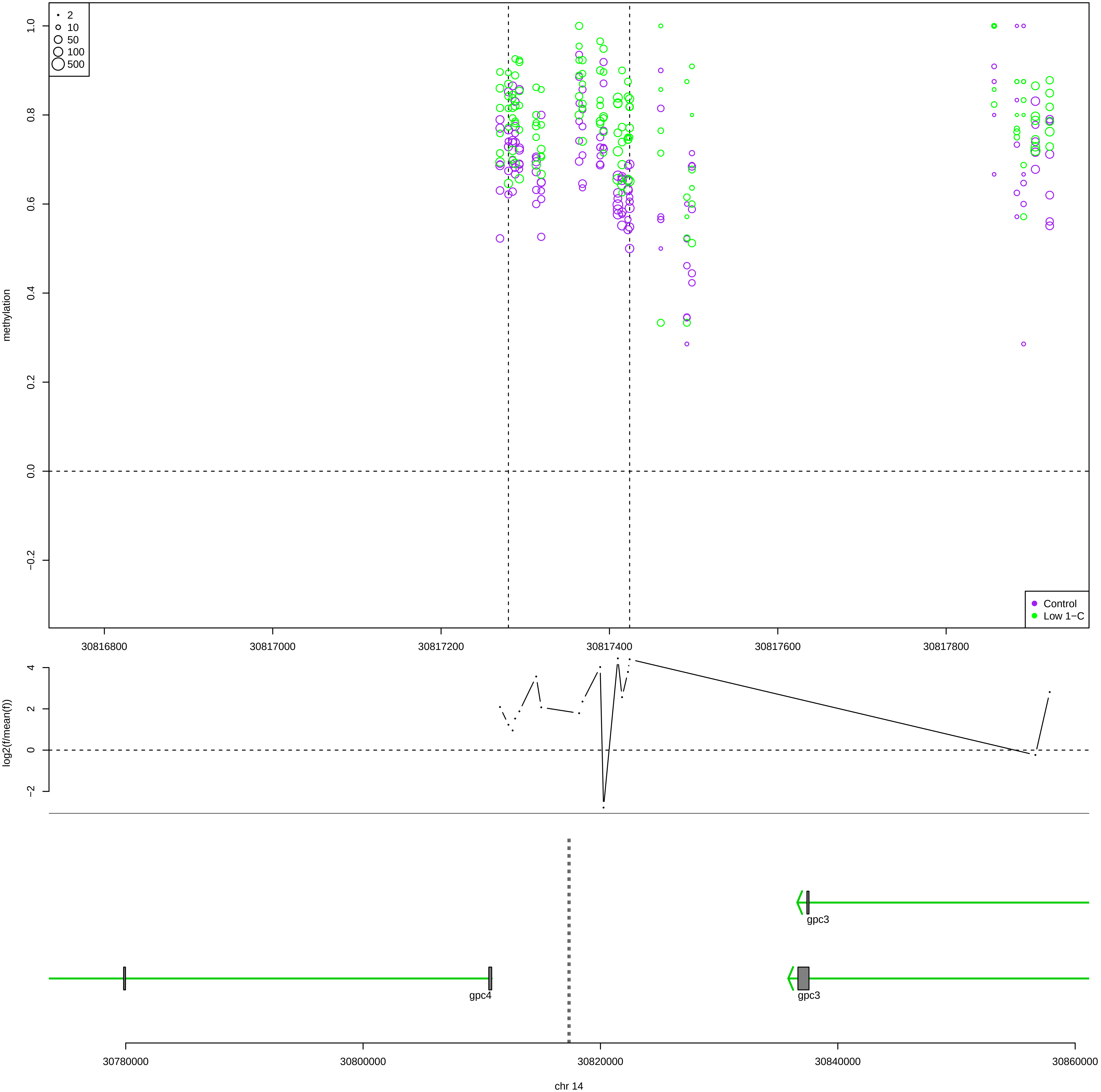

2
10
50
100
500
methylation
1.0
0.8
0.6
0.4
0.2
0.0
−0.2
Control
Low 1−C
30816800
30817000
30817200
30817400
30817600
30817800
log2(f/mean(f))
4
2
0
−2
gpc3
gpc4
gpc3
30780000
30800000
30820000
30840000
30860000
chr 14

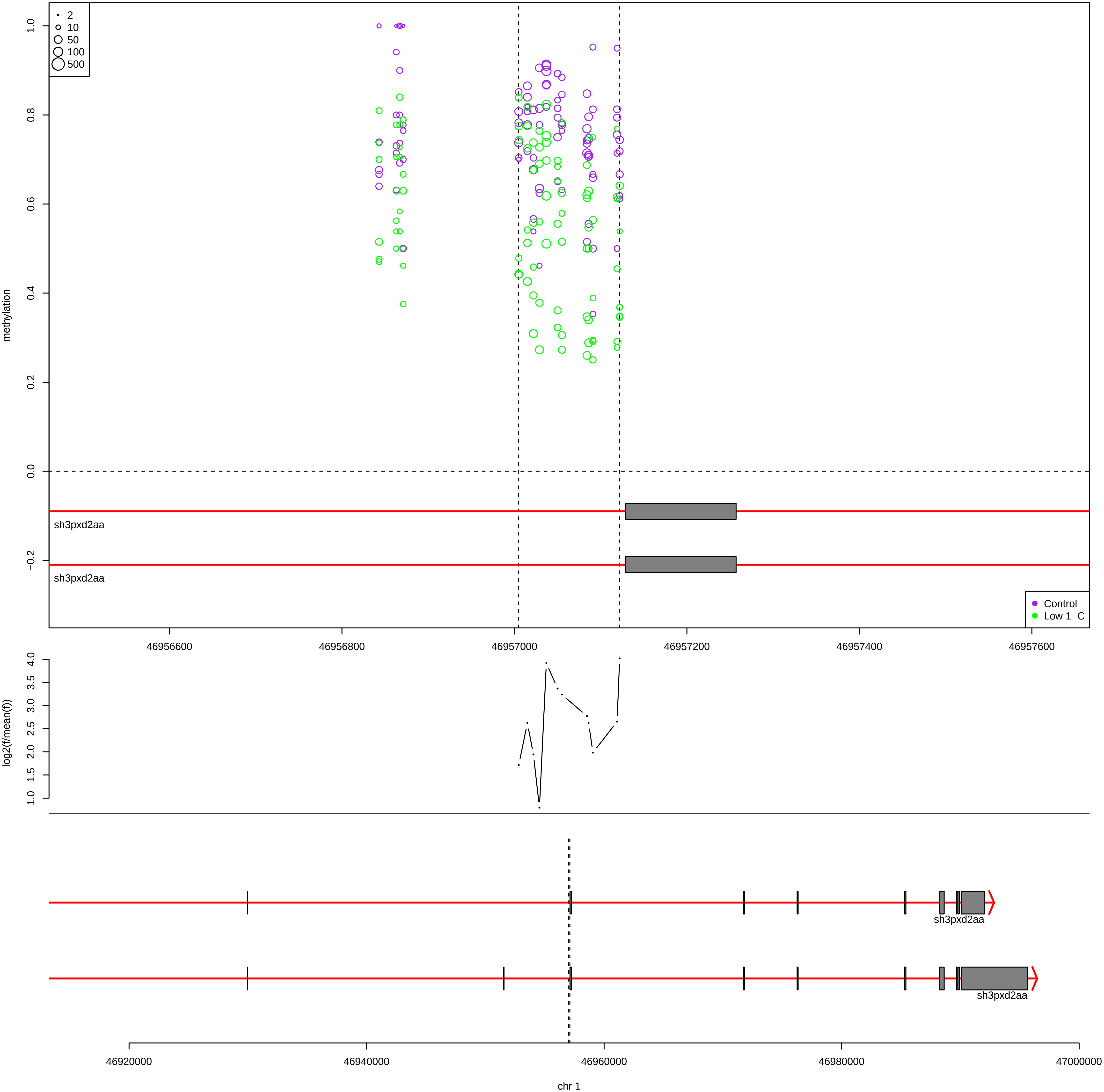

2
10
50
100
500
methylation
1.0
0.8
0.6
0.4
0.2
0.0
−0.2
sh3pxd2aa
sh3pxd2aa
Control
Low 1−C
46956600
46956800
46957000
46957200
46957400
46957600
log2(f/mean(f))
4.0
3.5
3.0
2.5
2.0
1.5
1.0
sh3pxd2aa
sh3pxd2aa
46920000
46940000
46960000
46980000
47000000
chr 1

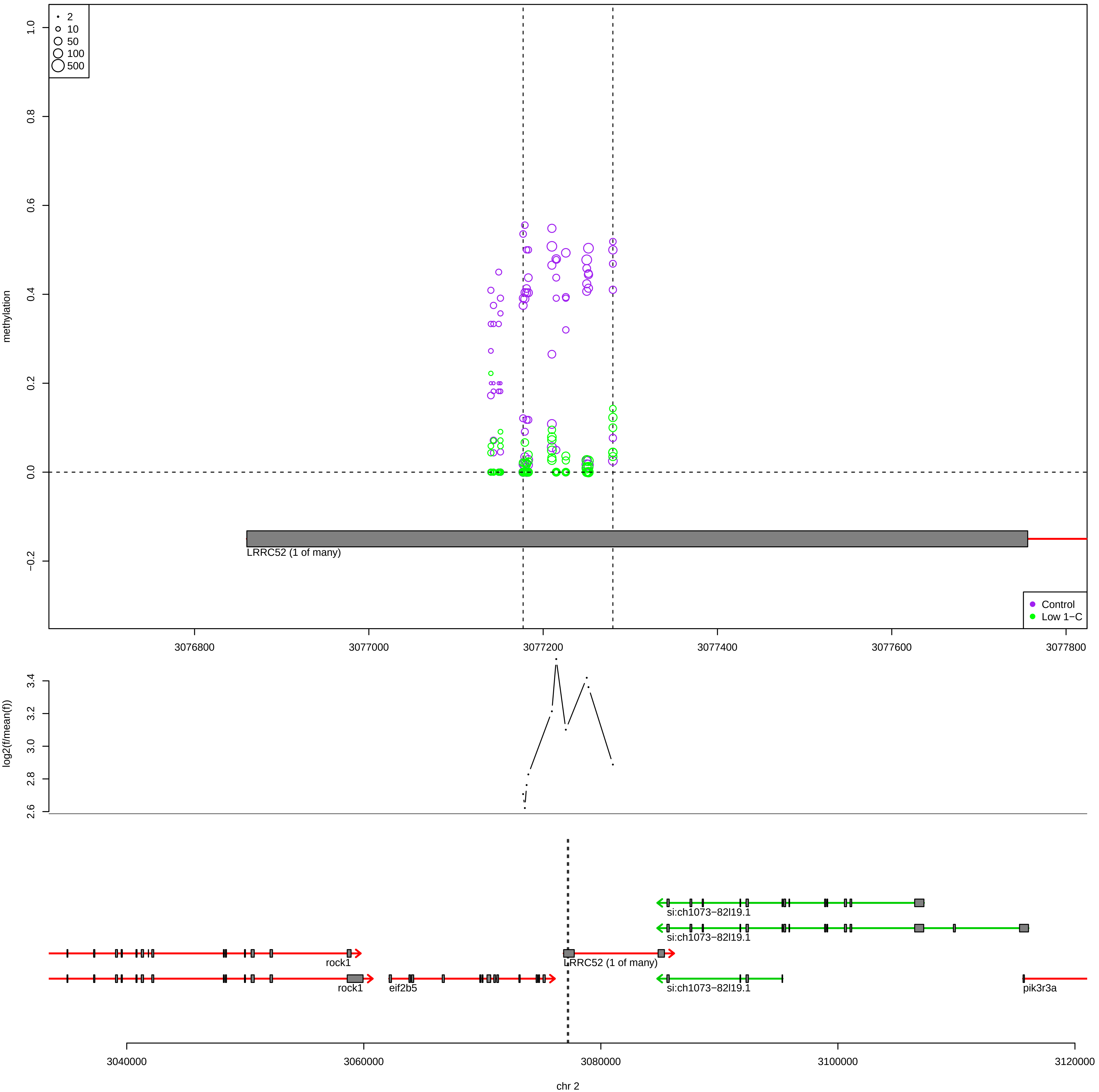

2
10
50
100
500
methylation
1.0
0.8
0.6
0.4
0.2
0.0
−0.2
LRRC52 (1 of many)
Control
Low 1−C
3076800
3077000
3077200
3077400
3077600
3077800
log2(f/mean(f))
3.4
3.2
3.0
2.8
2.6
si:ch1073−82I19.1
si:ch1073−82I19.1
rock1
LRRC52 (1 of many)
rock1
eif2b5
si:ch1073−82I19.1
pik3r3a
3040000
3060000
3080000
3100000
3120000
chr 2

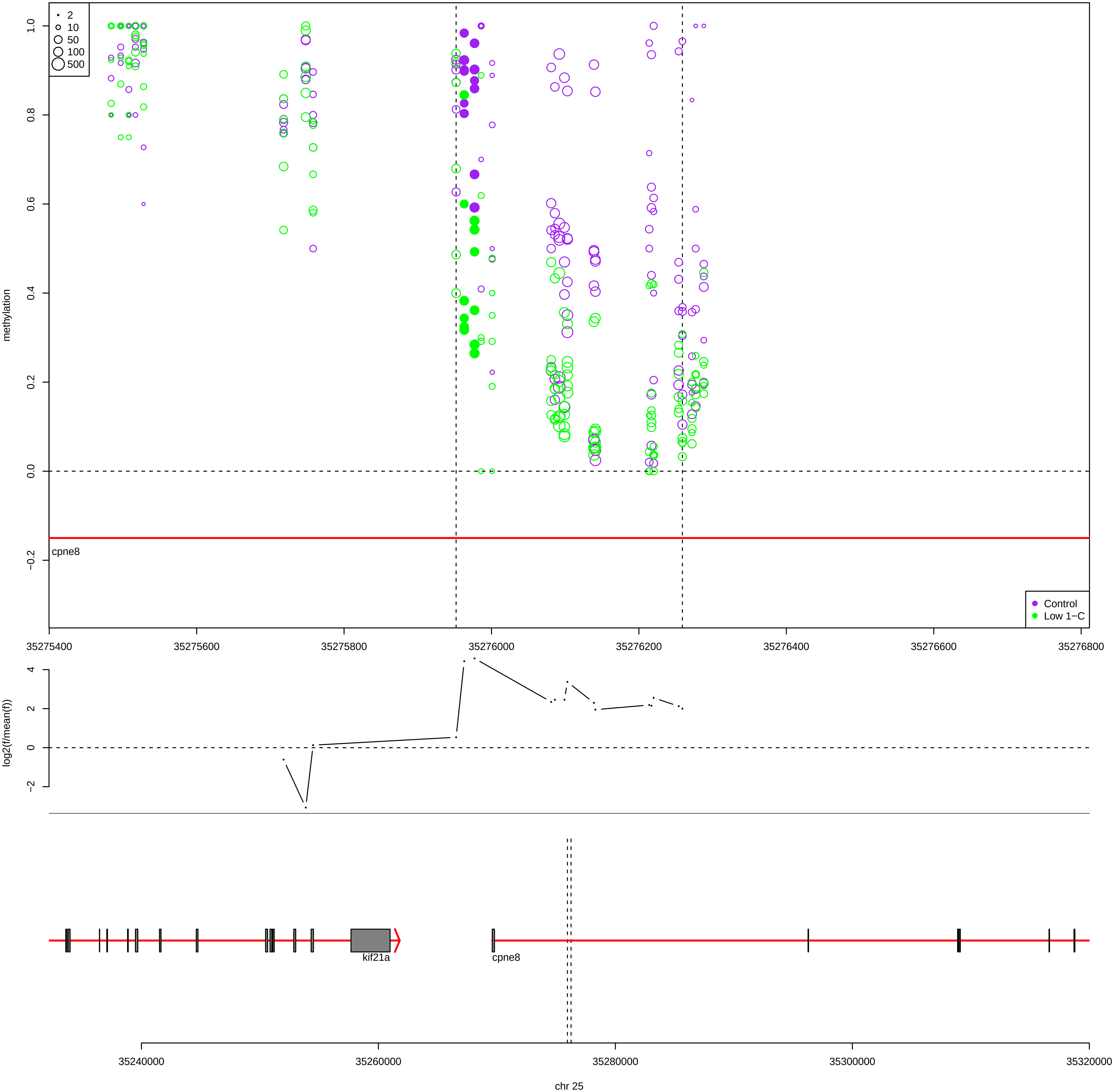

methylation
1.0
0.8
0.6
0.4
0.2
0.0
−0.2
2
10
50
100
500
cpne8
Control
Low 1−C
35275400
35275600
35275800
35276000
35276200
35276400
35276600
35276800
log2(f/mean(f))
4
2
0
−2
kif21a
cpne8
35240000
35260000
35280000
35300000
35320000
chr 25

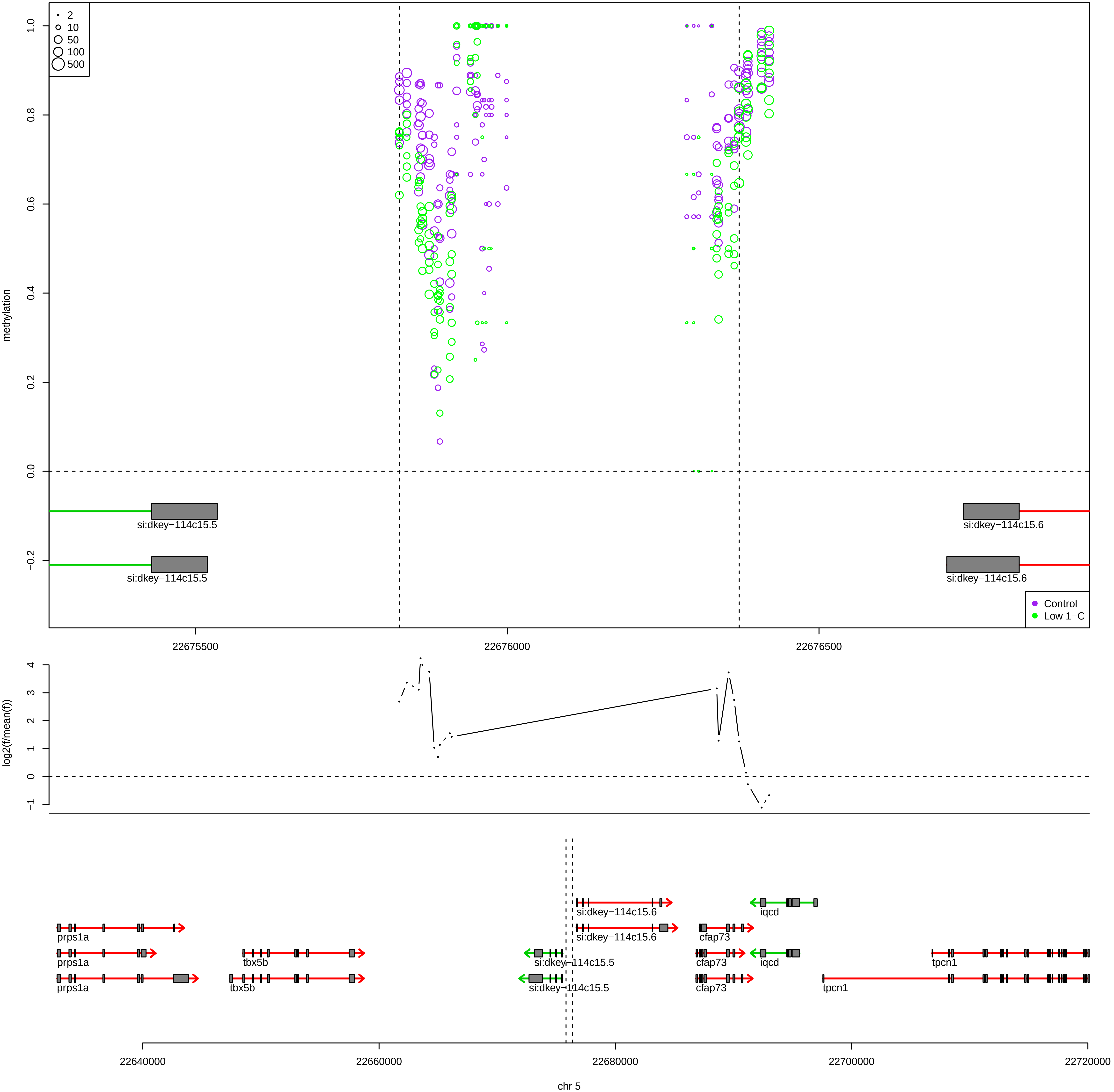

2
10
50
100
500
methylation
1.0
0.8
0.6
0.4
0.2
0.0
−0.2
si:dkey−114c15.5
si:dkey−114c15.5
si:dkey−114c15.6
si:dkey−114c15.6
Control
Low 1−C
22675500
22676000
22676500
log2(f/mean(f))
4
3
2
1
0
−1
si:dkey−114c15.6
iqcd
prps1a
si:dkey−114c15.6
cfap73
prps1a
tbx5b
si:dkey−114c15.5
cfap73
iqcd
tpcn1
prps1a
tbx5b
si:dkey−114c15.5
cfap73
tpcn1
22640000
22660000
22680000
22700000
22720000
chr 5

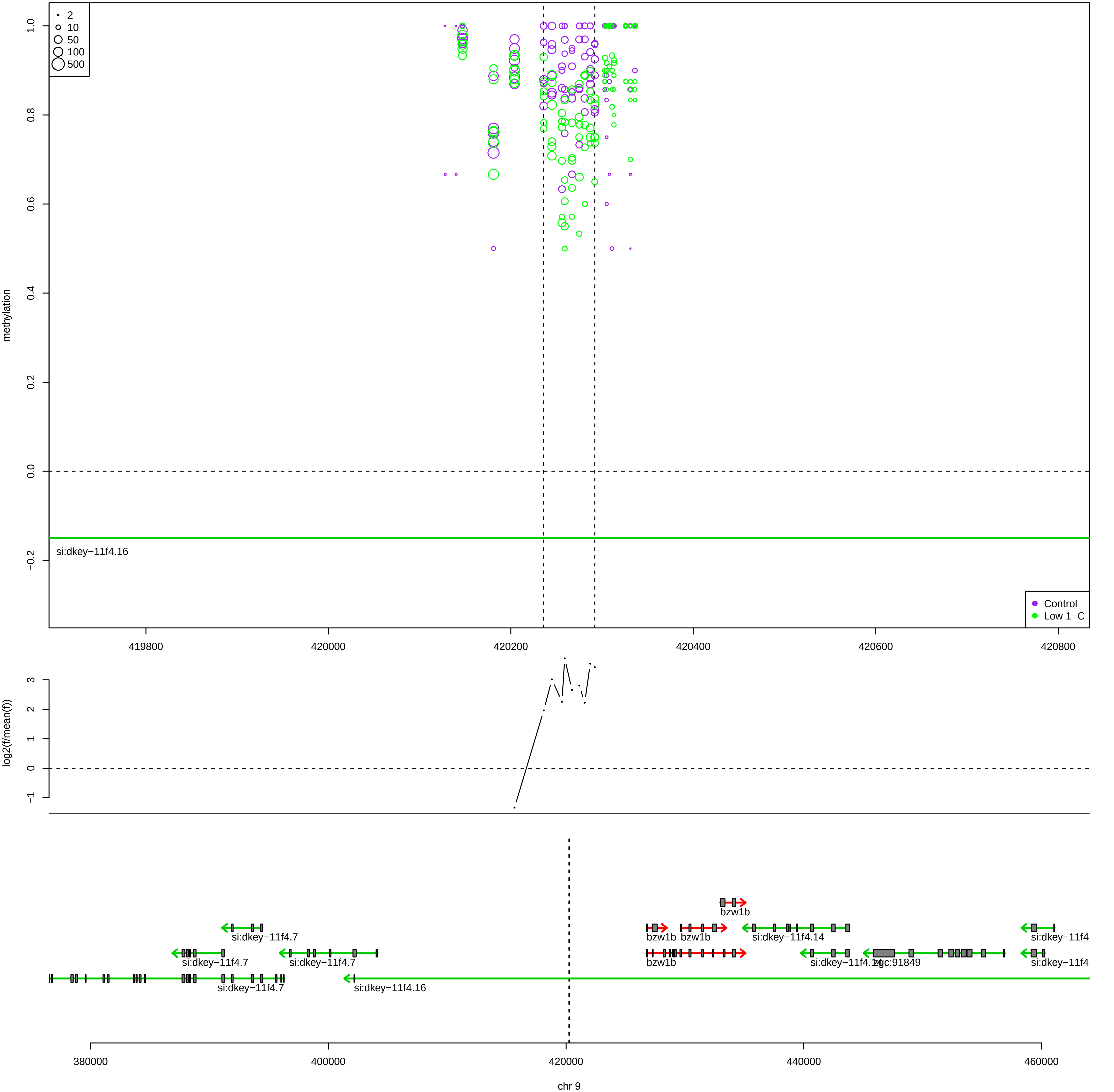

2
10
50
100
500
methylation
1.0
0.8
0.6
0.4
0.2
0.0
−0.2
si:dkey−11f4.16
Control
Low 1−C
419800
420000
420200
420400
420600
420800
log2(f/mean(f))
3
2
1
0
−1
bzw1b
si:dkey−11f4.7
bzw1b
bzw1b
si:dkey−11f4.14
si:dkey−11f4
si:dkey−11f4.7
si:dkey−11f4.7
bzw1b
si:dkey−11f4.14
zgc:91849
si:dkey−11f4
si:dkey−11f4.7
si:dkey−11f4.16
380000
400000
420000
440000
460000
chr 9

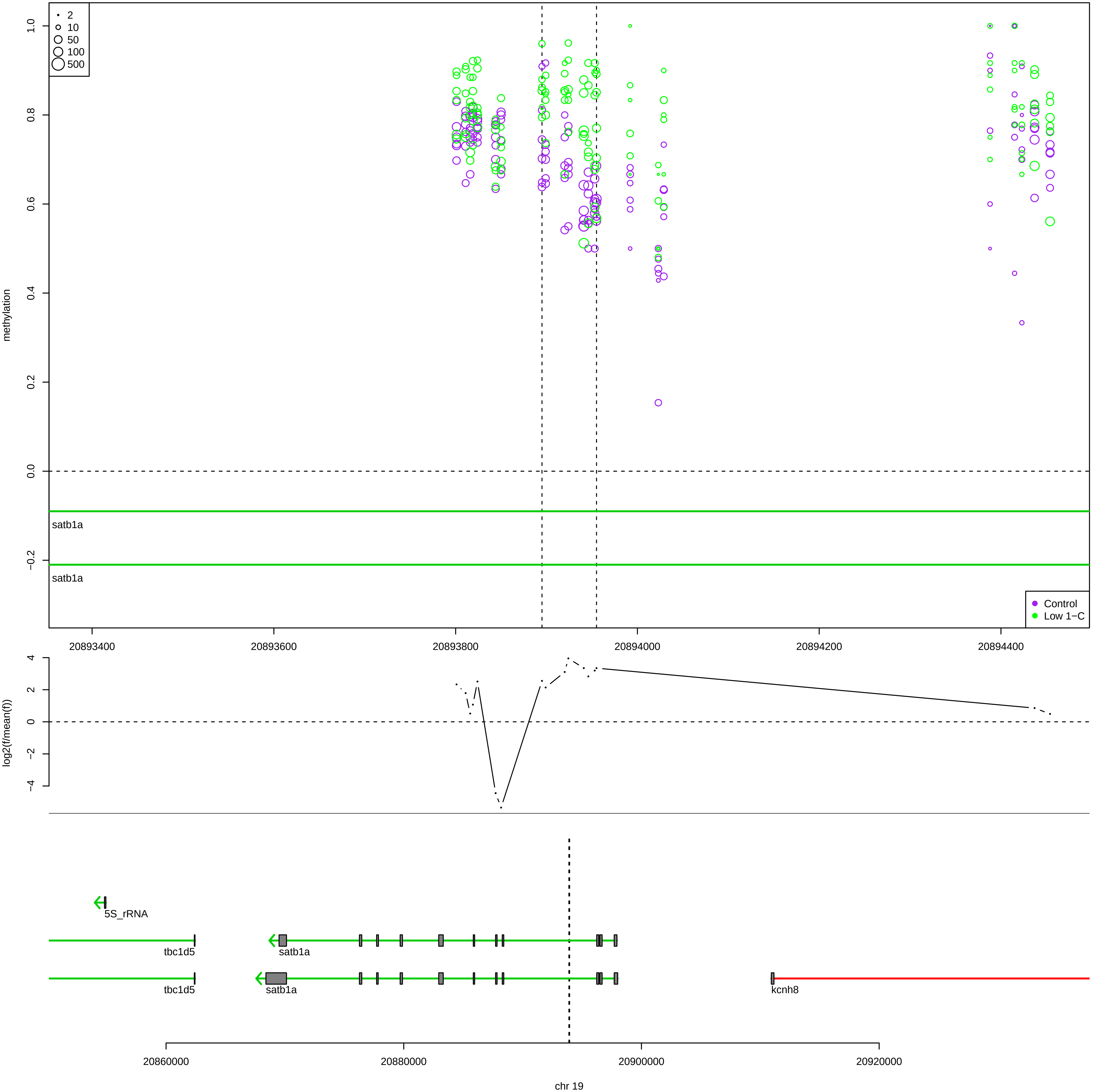

2
10
50
100
500
methylation
1.0
0.8
0.6
0.4
0.2
0.0
−0.2
satb1a
satb1a
Control
Low 1−C
20893400
20893600
20893800
20894000
20894200
20894400
log2(f/mean(f))
4
2
0
−2
−4
5S_rRNA
tbc1d5
satb1a
tbc1d5
satb1a
kcnh8
20860000
20880000
20900000
20920000
chr 19

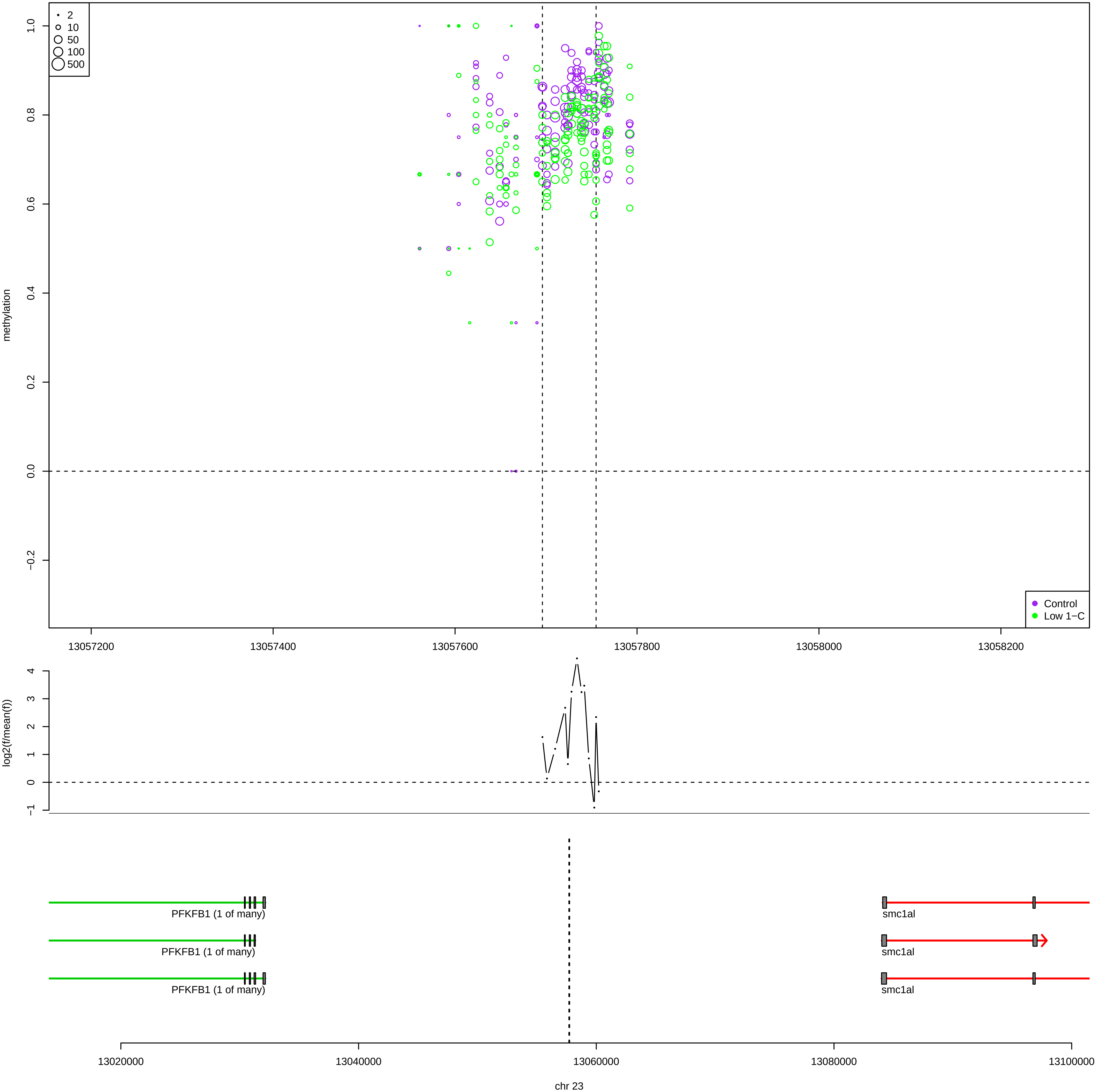

2
10
50
100
500
methylation
1.0
0.8
0.6
0.4
0.2
0.0
−0.2
Control
Low 1−C
13057200
13057400
13057600
13057800
13058000
13058200
log2(f/mean(f))
4
3
2
1
0
−1
PFKFB1 (1 of many)
PFKFB1 (1 of many)
PFKFB1 (1 of many)
smc1al
smc1al
smc1al
13020000
13040000
13060000
13080000
13100000
chr 23

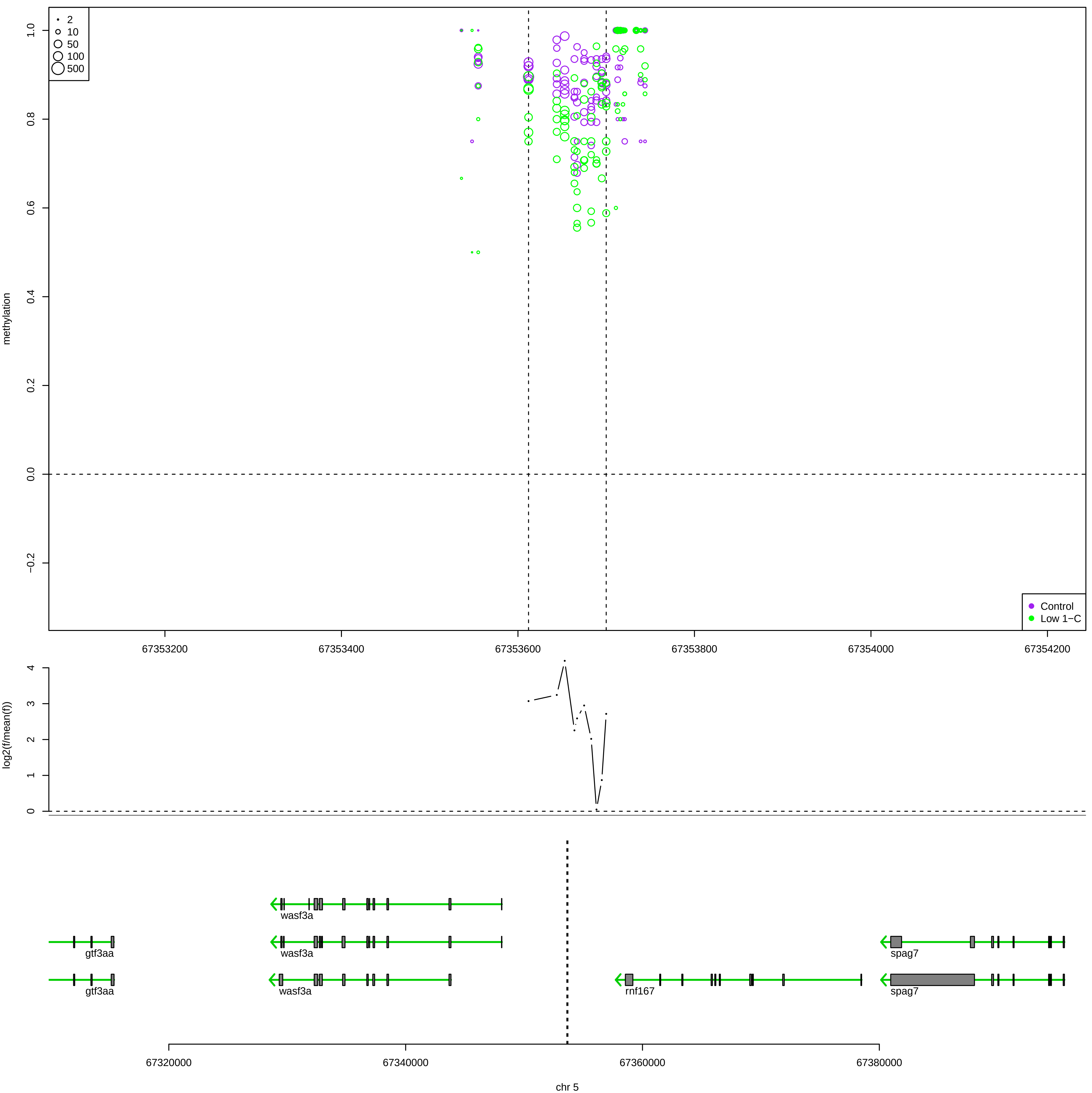
2
10
50
100
500
methylation
1.0
0.8
0.6
0.4
0.2
0.0
−0.2
Control
Low 1−C
67353200
67353400
67353600
67353800
67354000
67354200
log2(f/mean(f))
4
3
2
1
0
wasf3a
gtf3aa
wasf3a
spag7
gtf3aa
wasf3a
rnf167
spag7
67320000
67340000
67360000
67380000
chr 5

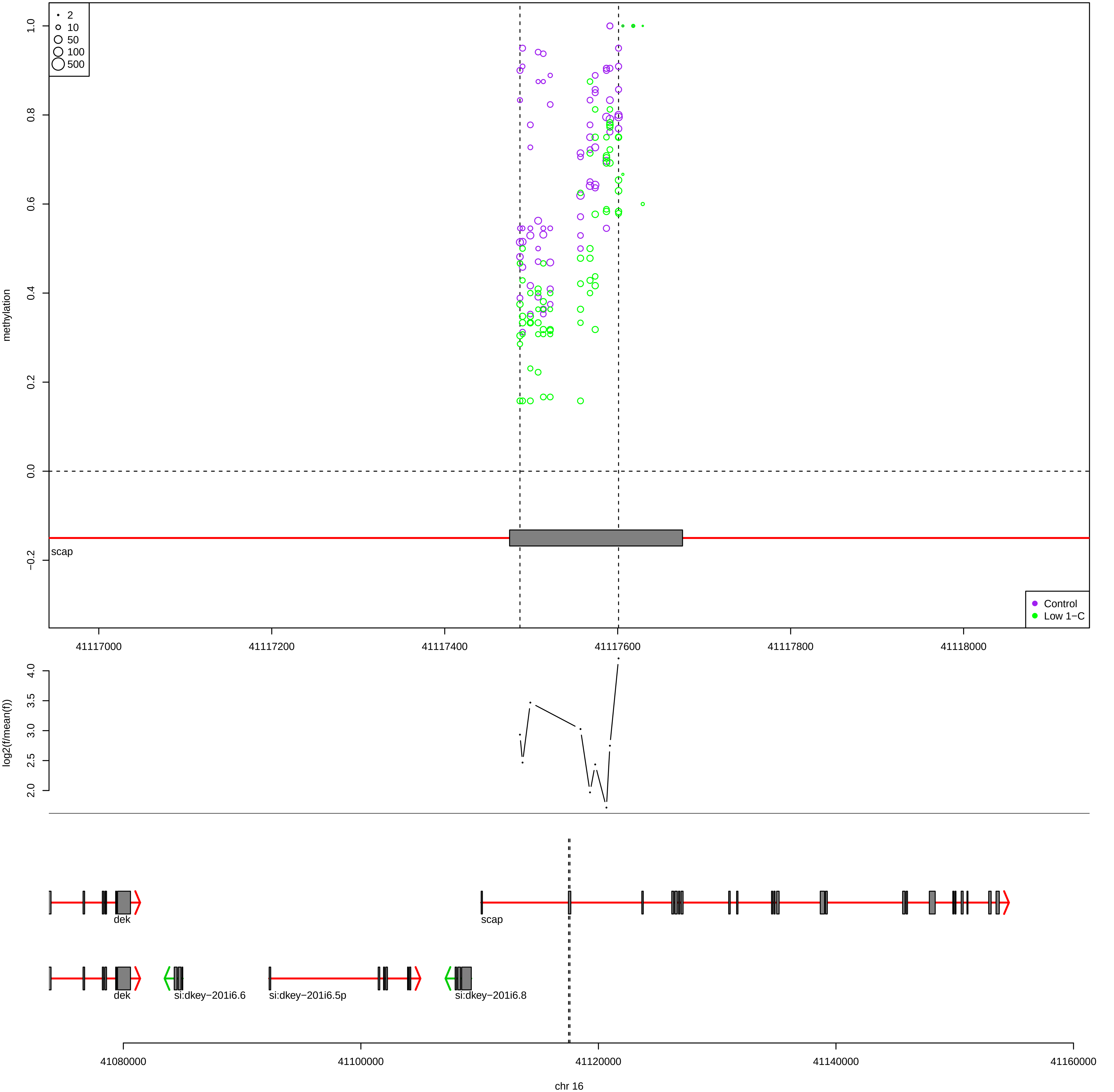

2
10
50
100
500
methylation
1.0
0.8
0.6
0.4
0.2
0.0
−0.2
scap
Control
Low 1−C
41117000
41117200
41117400
41117600
41117800
41118000
log2(f/mean(f))
4.0
3.5
3.0
2.5
2.0
dek
scap
dek
si:dkey−201i6.6
si:dkey−201i6.5p
si:dkey−201i6.8
41080000
41100000
41120000
41140000
41160000
chr 16

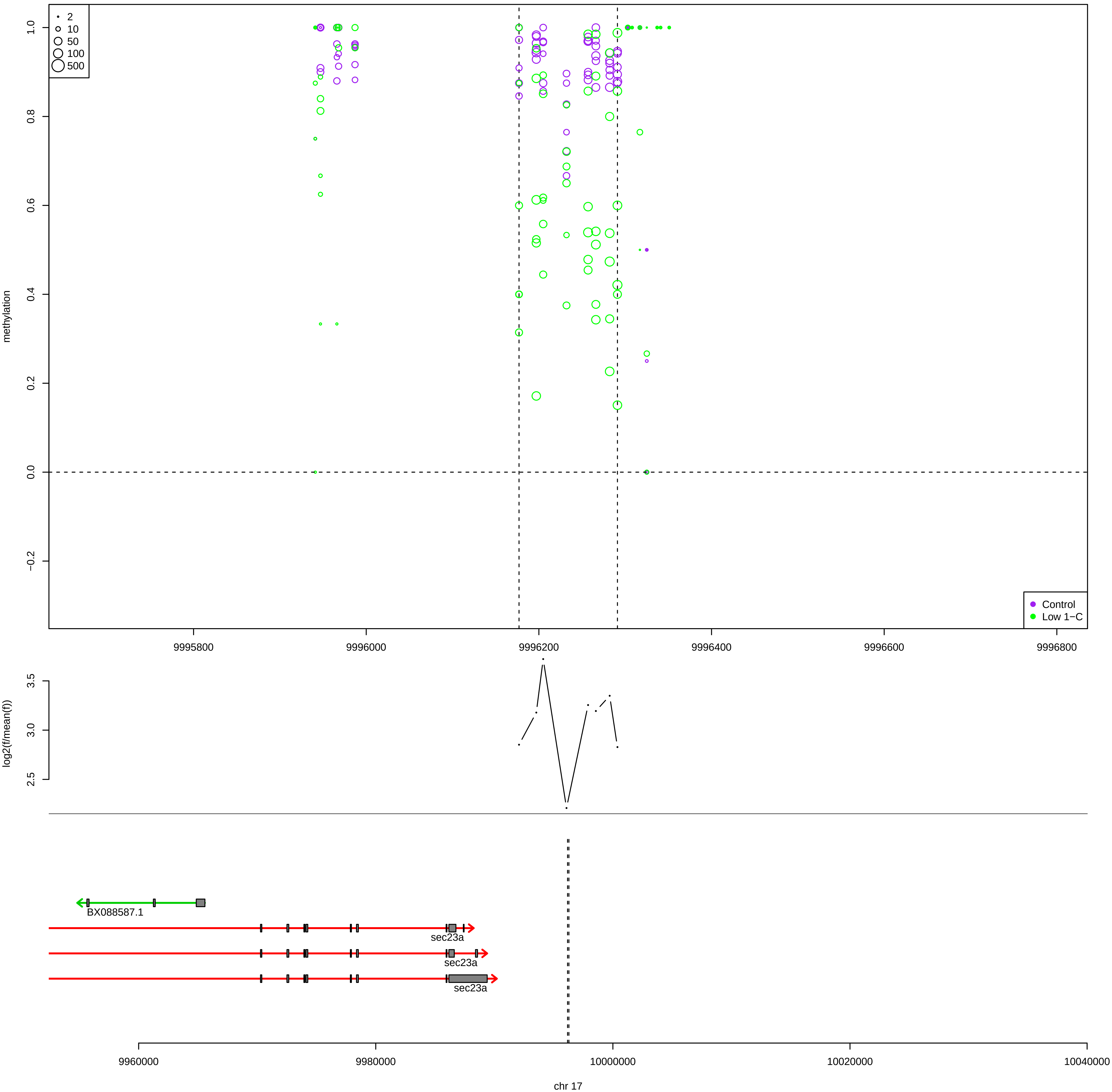

2
10
50
100
500
methylation
1.0
0.8
0.6
0.4
0.2
0.0
−0.2
Control
Low 1−C
9995800
9996000
9996200
9996400
9996600
9996800
log2(f/mean(f))
3.5
3.0
2.5
BX088587.1
sec23a
sec23a
sec23a
9960000
9980000
10000000
10020000
10040000
chr 17

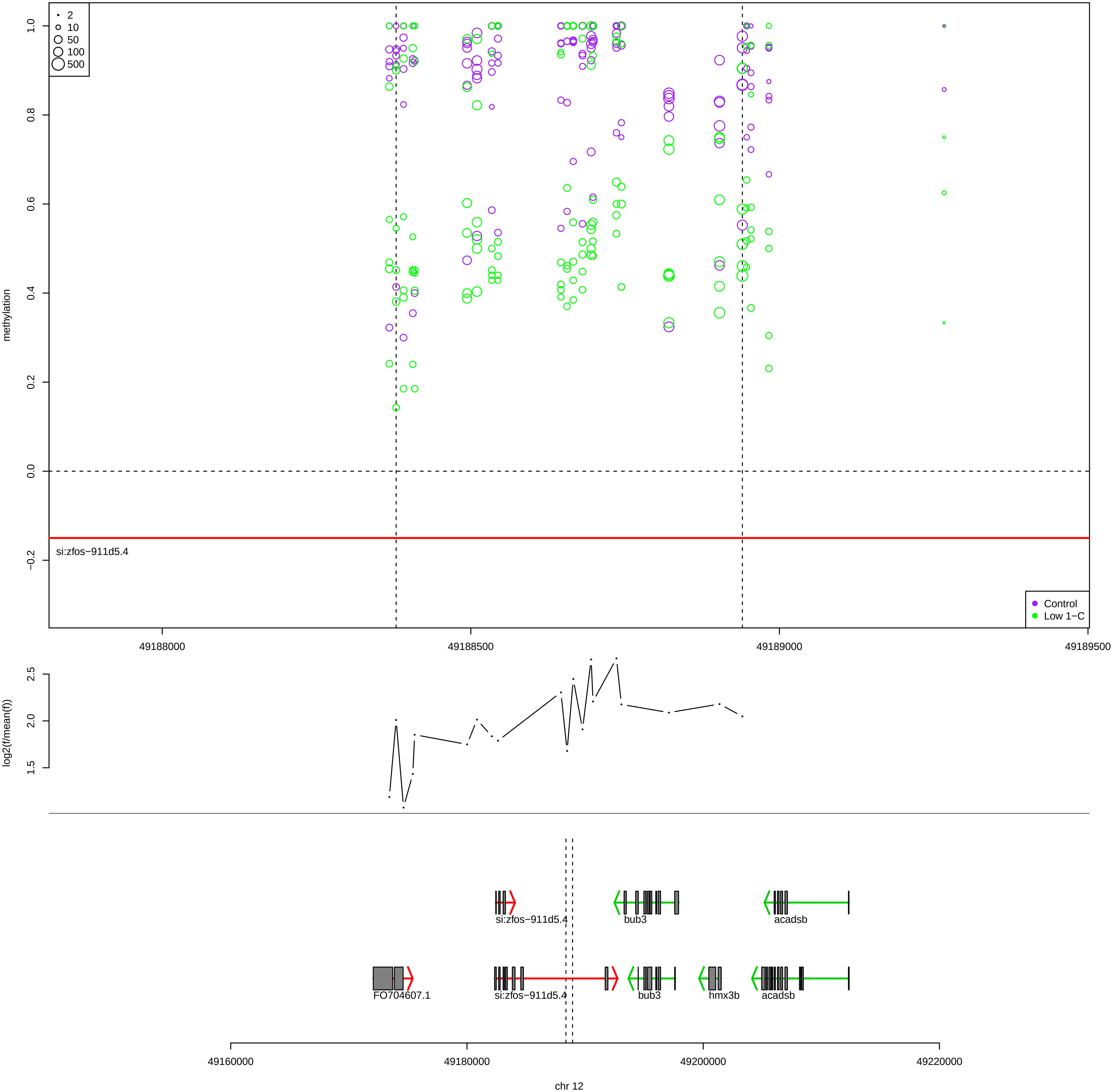

2
10
50
100
500
methylation
1.0
0.8
0.6
0.4
0.2
0.0
−0.2
si:zfos−911d5.4
Control
Low 1−C
49188000
49188500
49189000
49189500
log2(f/mean(f))
2.5
2.0
1.5
si:zfos−911d5.4
bub3
acadsb
FO704607.1
si:zfos−911d5.4
bub3
hmx3b
acadsb
49160000
49180000
49200000
49220000
chr 12

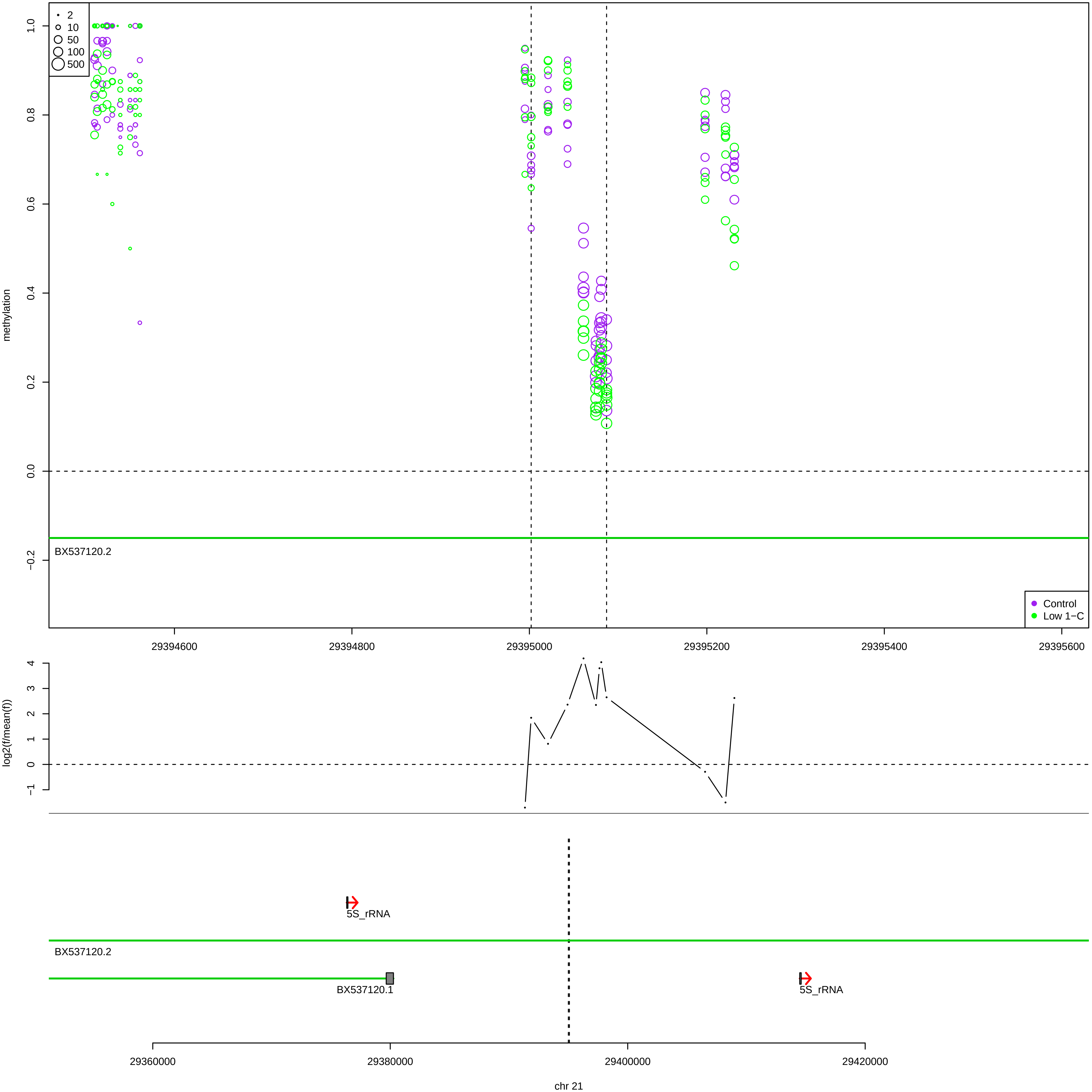

2
10
50
100
500
methylation
1.0
0.8
0.6
0.4
0.2
0.0
−0.2
BX537120.2
Control
Low 1−C
29394600
29394800
29395000
29395200
29395400
29395600
log2(f/mean(f))
4
3
2
1
0
−1
5S_rRNA
BX537120.2
BX537120.1
5S_rRNA
29360000
29380000
29400000
29420000
chr 21

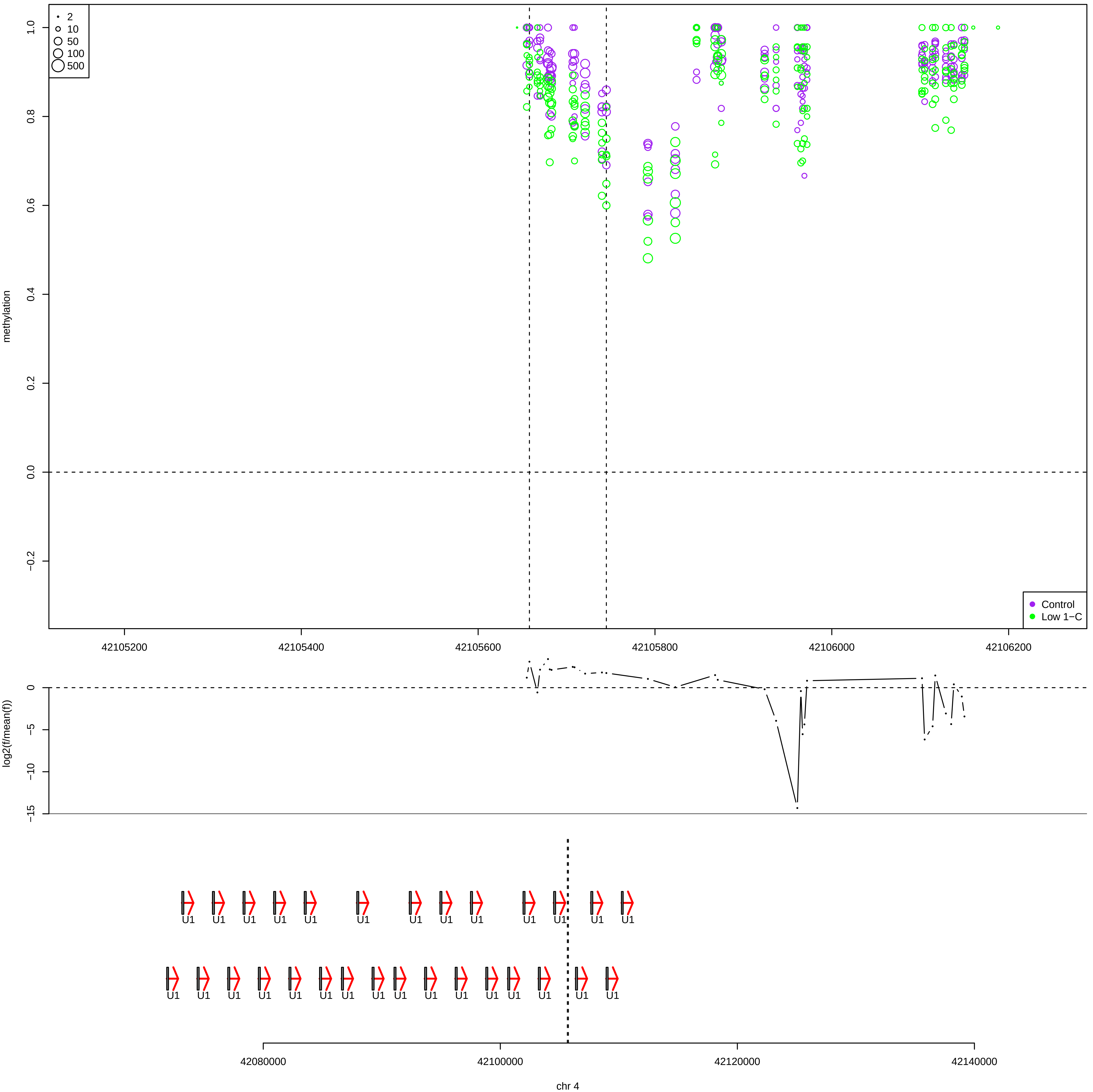
2
10
50
100
500
methylation
1.0
0.8
0.6
0.4
0.2
0.0
−0.2
Control
Low 1−C
42105200
42105400
42105600
42105800
42106000
42106200
log2(f/mean(f))
0
−5
−10
−15
U1
42080000
42100000
42120000
42140000
chr 4

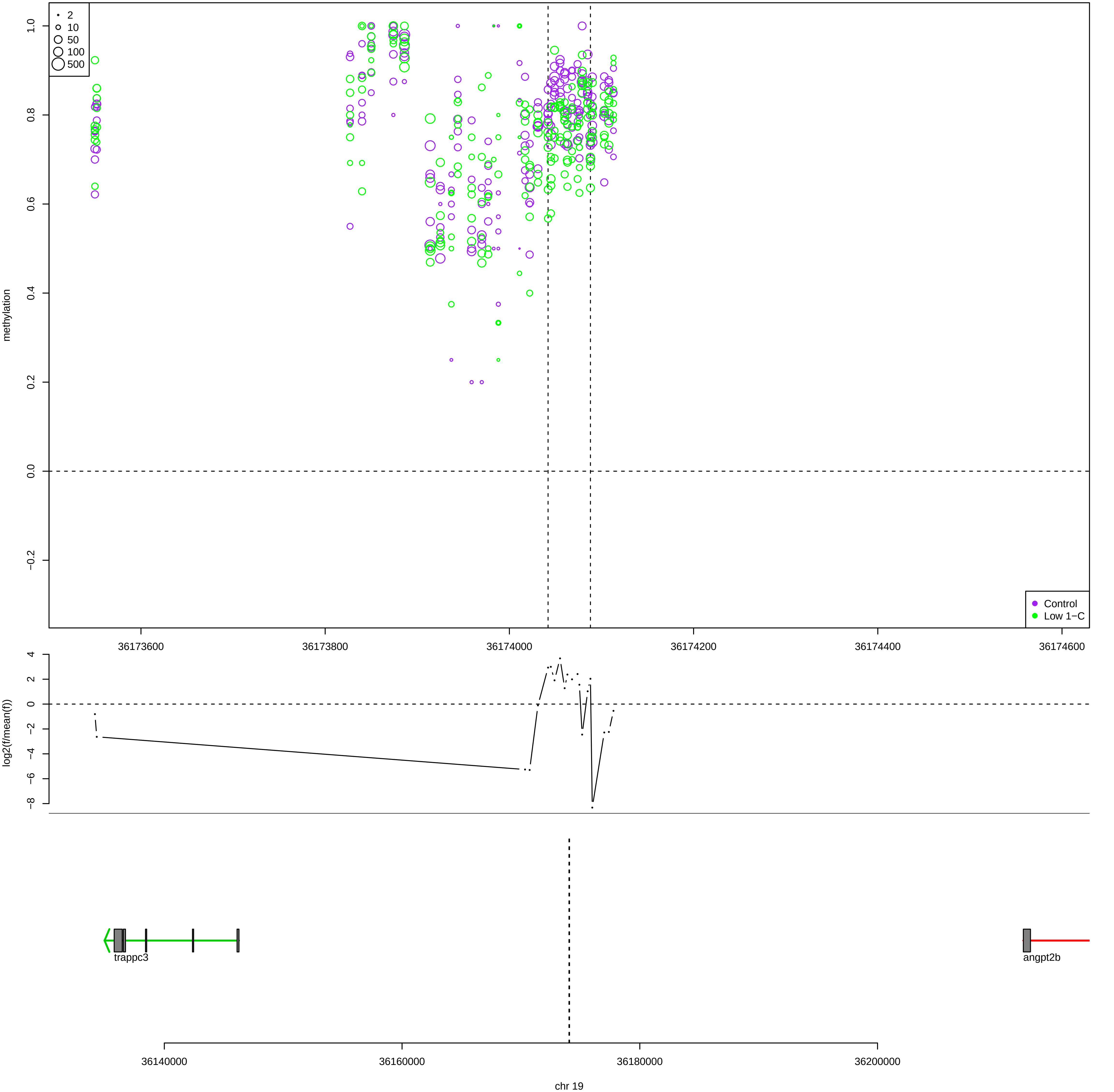

2
10
50
100
500
Control
Low 1−C
methylation
1.0
0.8
0.6
0.4
0.2
0.0
−0.2
36173600
36173800
36174000
36174200
36174400
36174600
log2(f/mean(f))
4
2
0
−2
−4
−6
−8
trappc3
angpt2b
36140000
36160000
36180000
36200000
chr 19

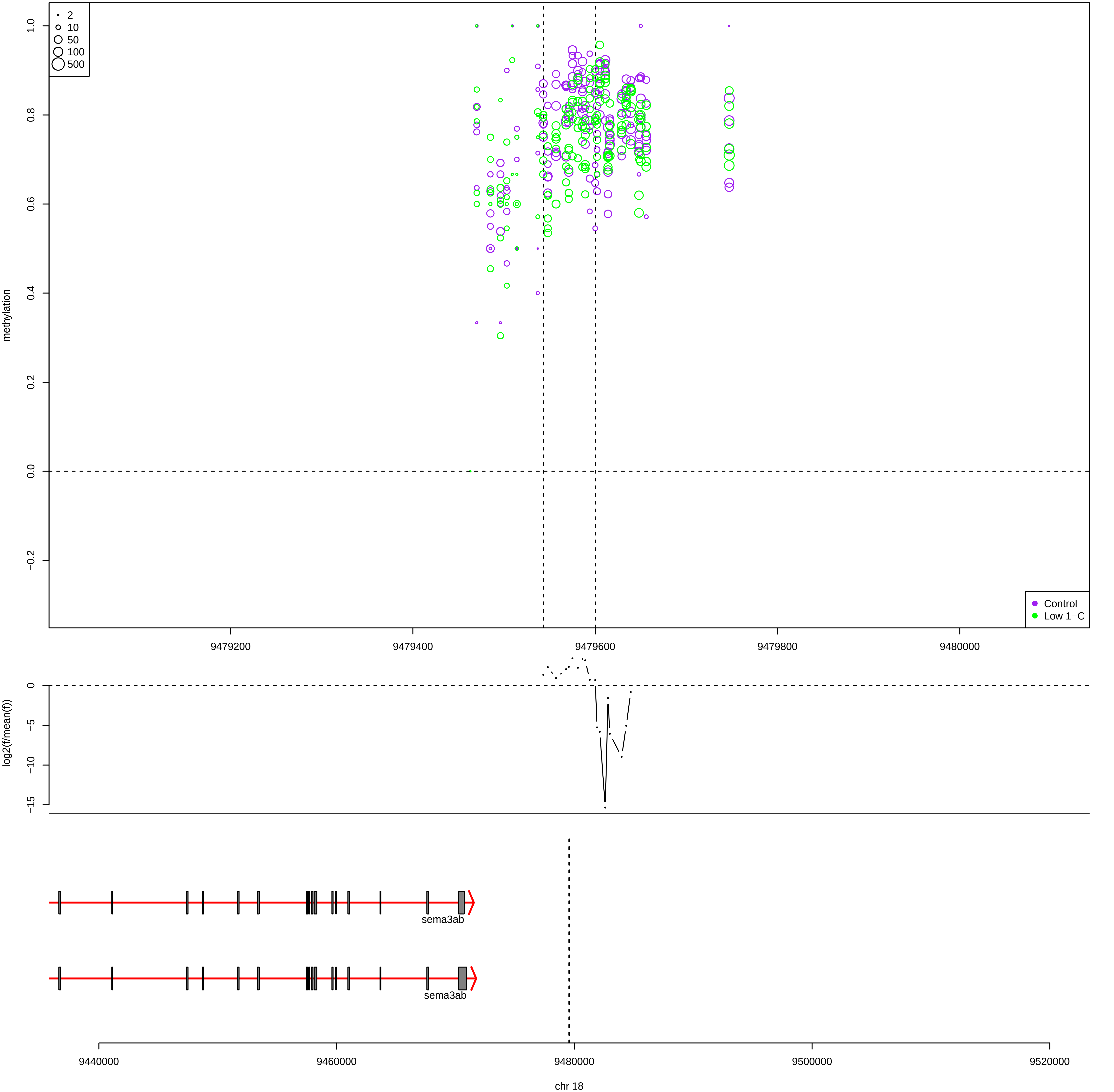

2
10
50
100
500
methylation
1.0
0.8
0.6
0.4
0.2
0.0
−0.2
Control
Low 1−C
9479200
9479400
9479600
9479800
9480000
log2(f/mean(f))
0
−5
−10
−15
sema3ab
sema3ab
9440000
9460000
9480000
9500000
9520000
chr 18

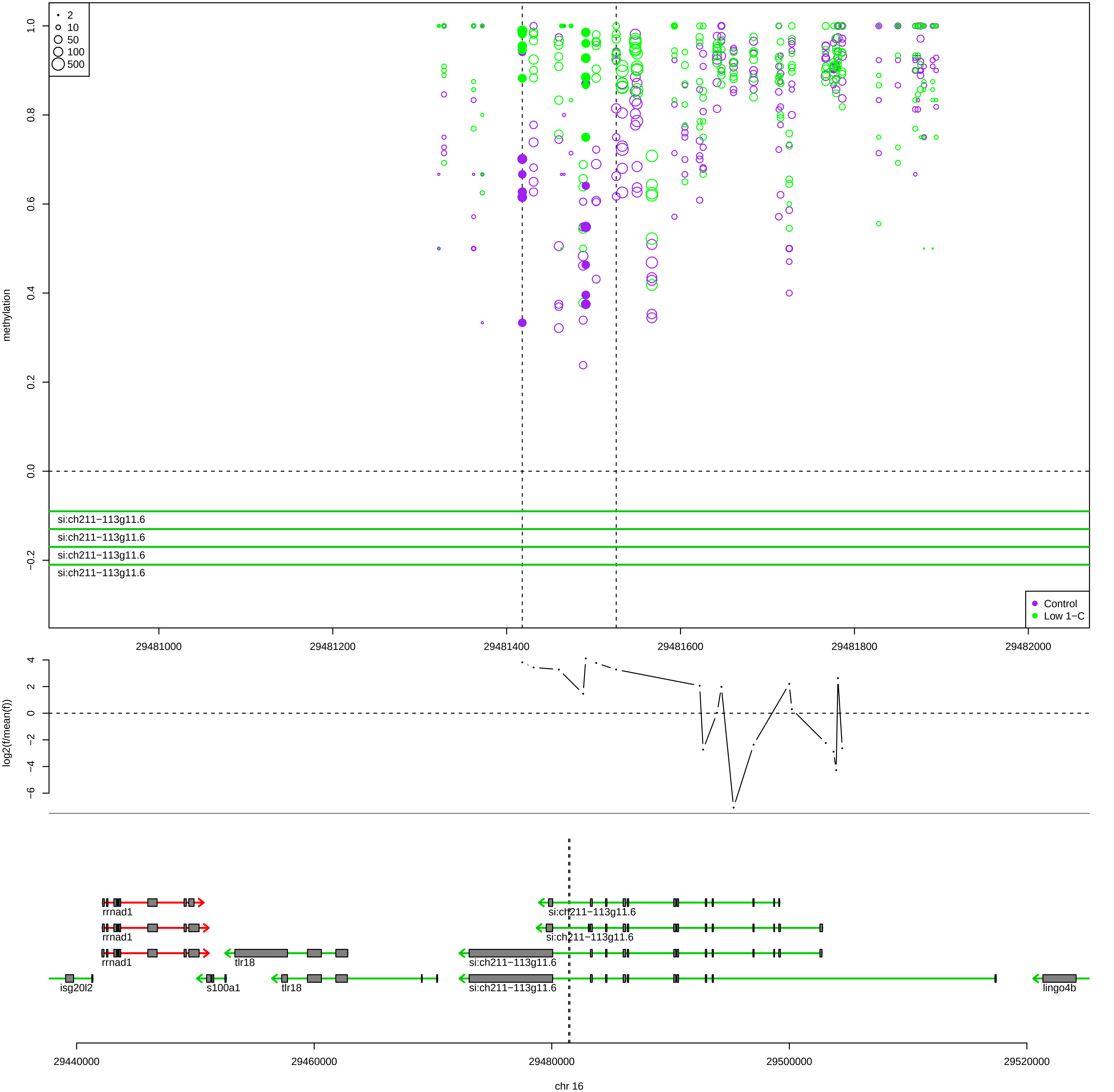
methylation
log2(f/mean(f))
si:ch211−113g11.6
Control
Low 1−C
29481000
29481200
29481400
29481600
29481800
29482000
rrnad1
tlr18
isg20l2
s100a1
lingo4b
29440000
29460000
29480000
29500000
29520000
chr 16

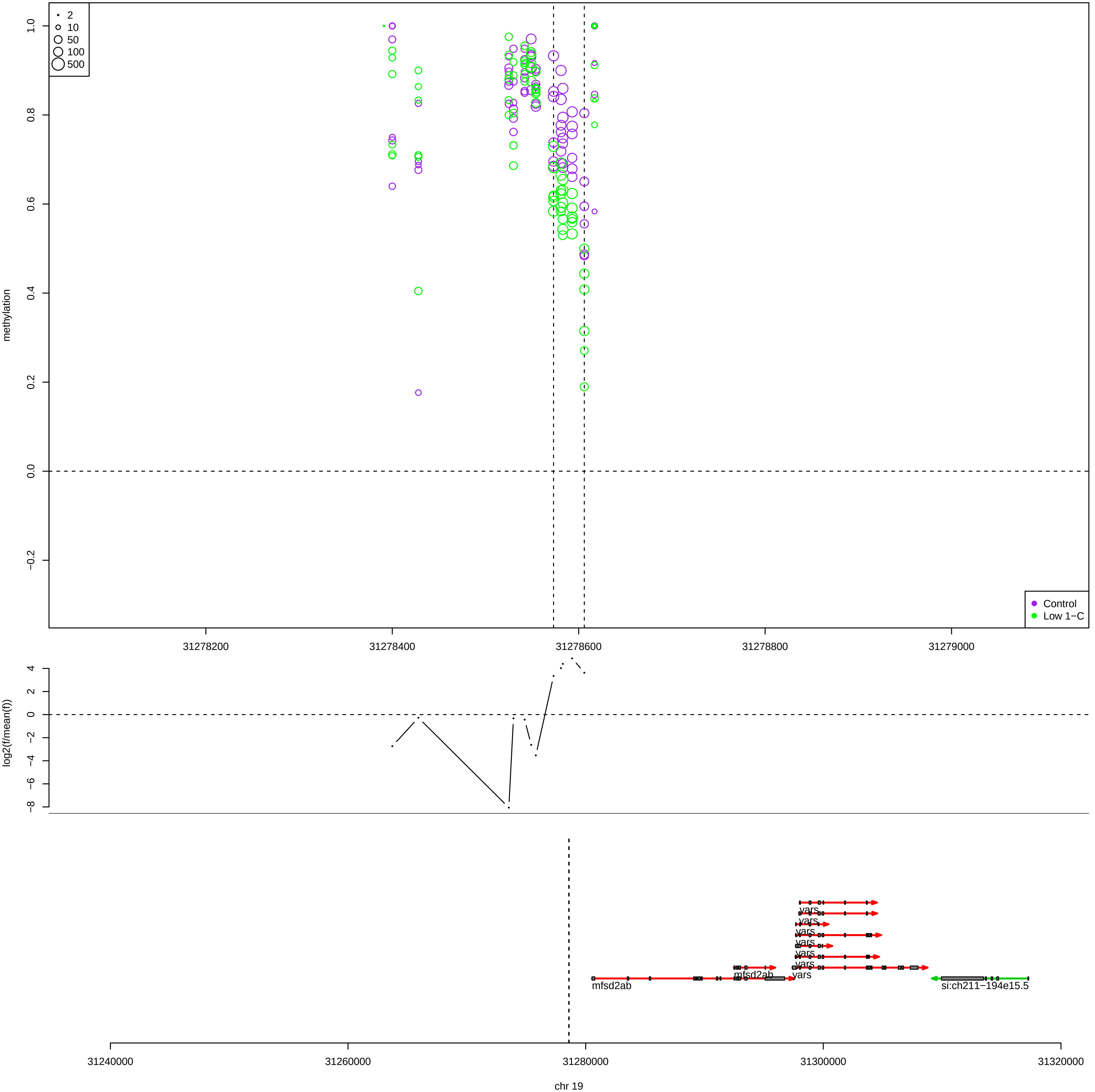

2
10
50
100
500
Control
Low 1−C
methylation
1.0
0.8
0.6
0.4
0.2
0.0
−0.2
31278200
31278400
31278600
31278800
31279000
log2(f/mean(f))
4
2
0
−2
−4
−6
−8
yars
mfsd2ab
mfsd2ab
si:ch211−194e15.5
31240000
31260000
31280000
31300000
31320000
chr 19

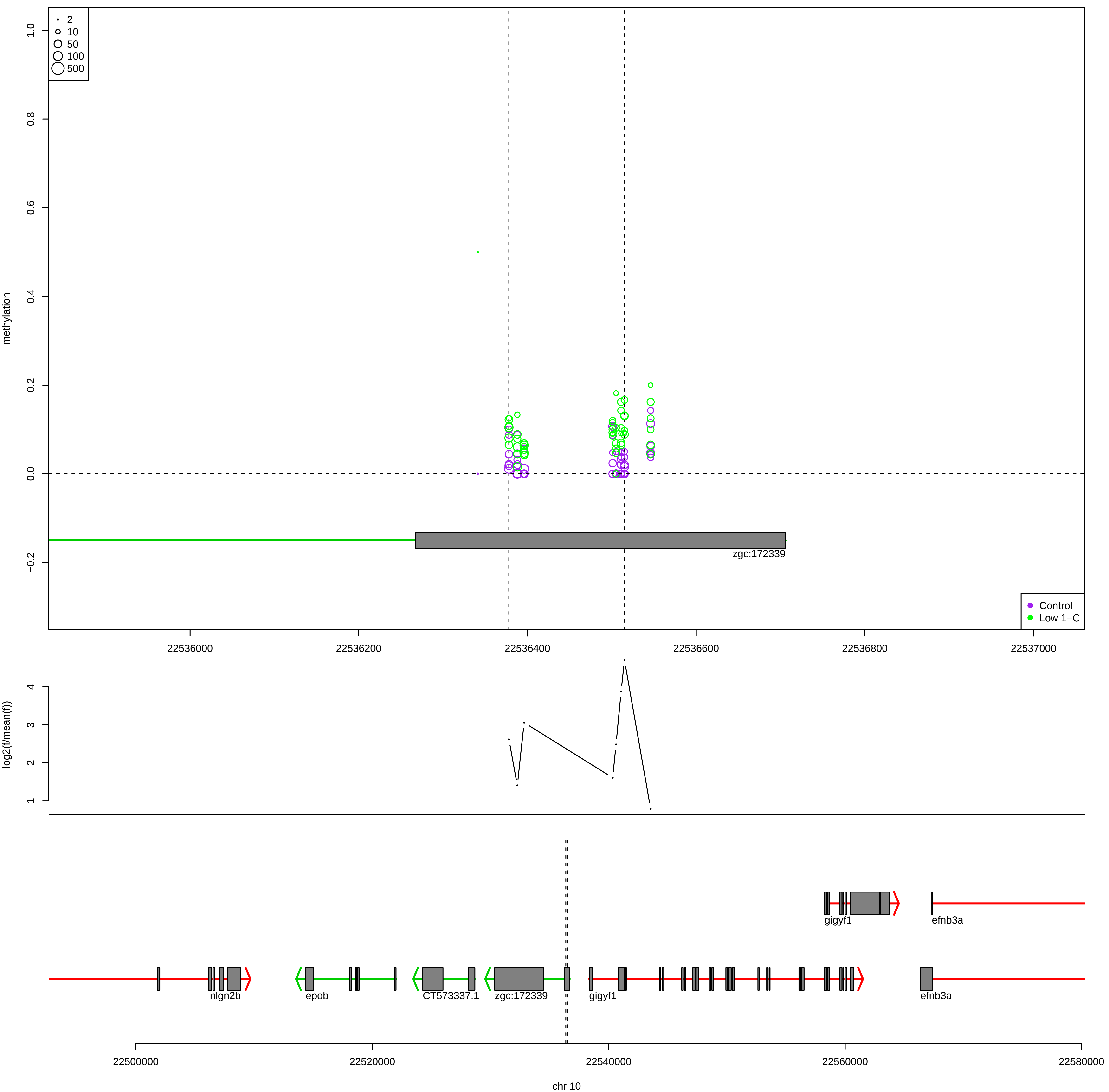

2
10
50
100
500
methylation
1.0
0.8
0.6
0.4
0.2
0.0
−0.2
zgc:172339
Control
Low 1−C
22536000
22536200
22536400
22536600
22536800
22537000
log2(f/mean(f))
4
3
2
1
gigyf1
efnb3a
nlgn2b
epob
CT573337.1
zgc:172339
gigyf1
efnb3a
22500000
22520000
22540000
22560000
22580000
chr 10

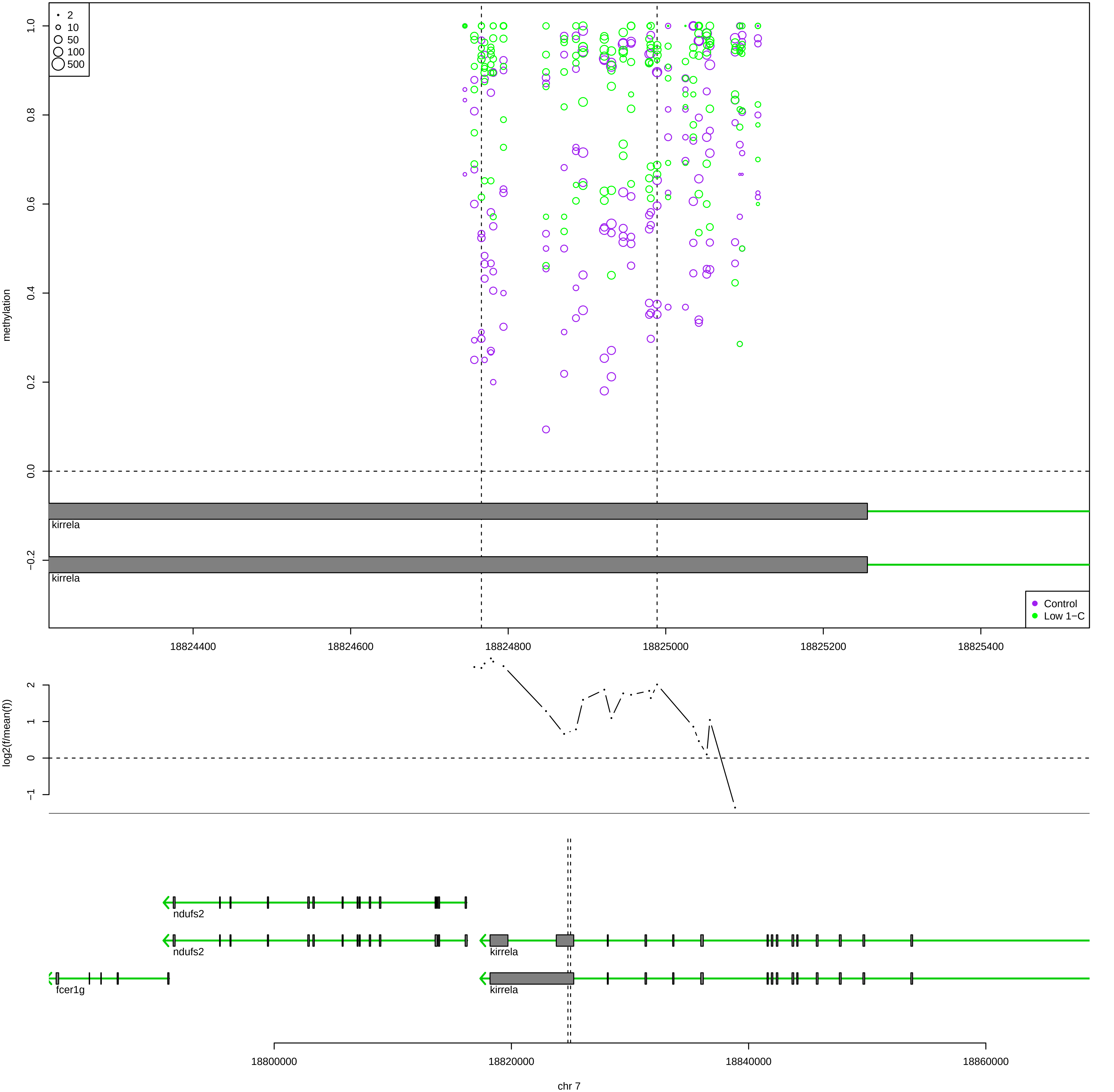

2
10
50
100
500
methylation
1.0
0.8
0.6
0.4
0.2
0.0
−0.2
kirrela
kirrela
Control
Low 1−C
18824400
18824600
18824800
18825000
18825200
18825400
log2(f/mean(f))
2
1
0
−1
ndufs2
ndufs2
kirrela
fcer1g
kirrela
18800000
18820000
18840000
18860000
chr 7

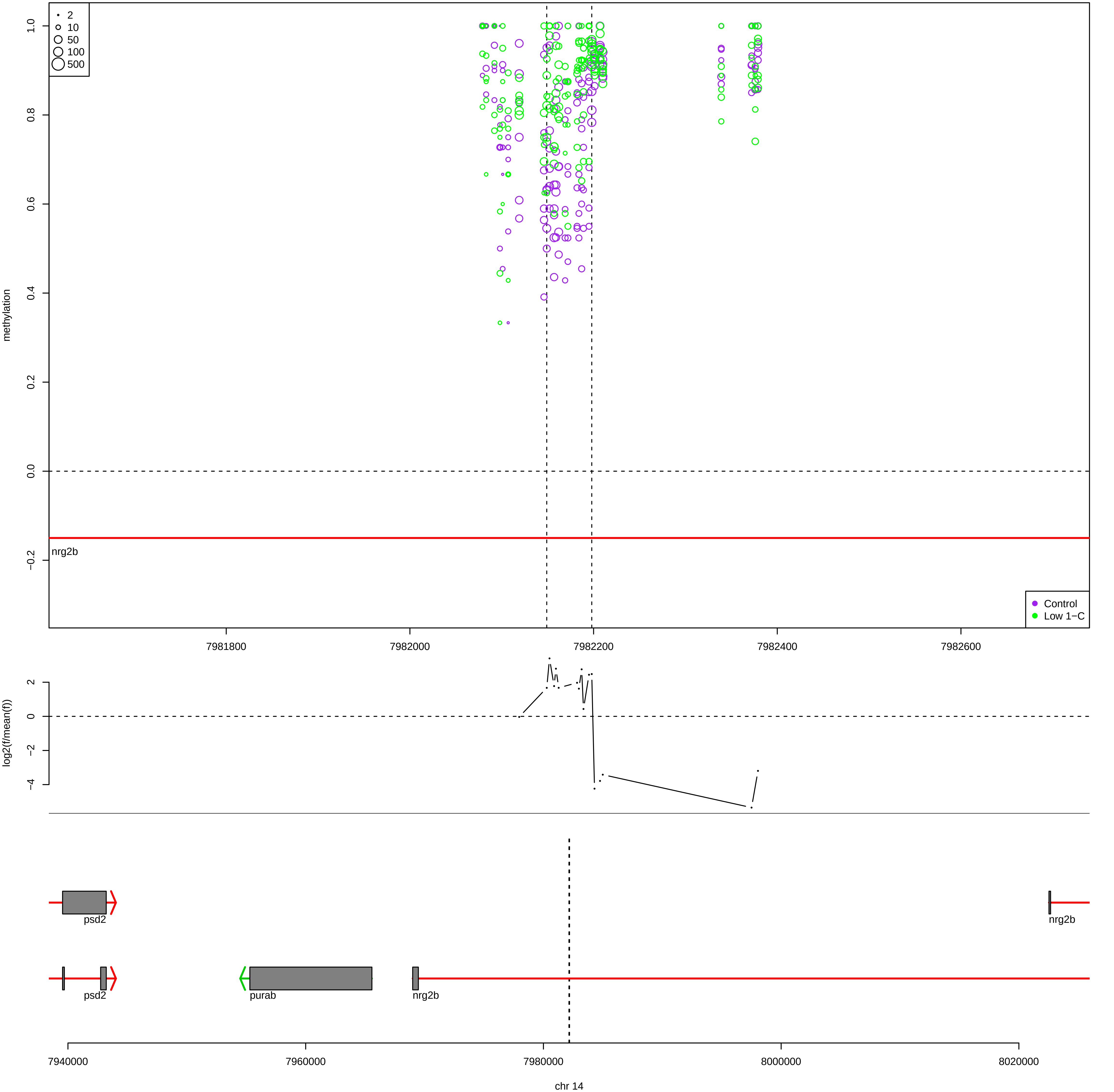

2
10
50
100
500
methylation
1.0
0.8
0.6
0.4
0.2
0.0
-0.2
nrg2b
Control
Low 1−C
7981800
7982000
7982200
7982400
7982600
log2(f/mean(f))
2
0
−2
−4
psd2
nrg2b
psd2
purab
nrg2b
7940000
7960000
7980000
8000000
8020000
chr 14

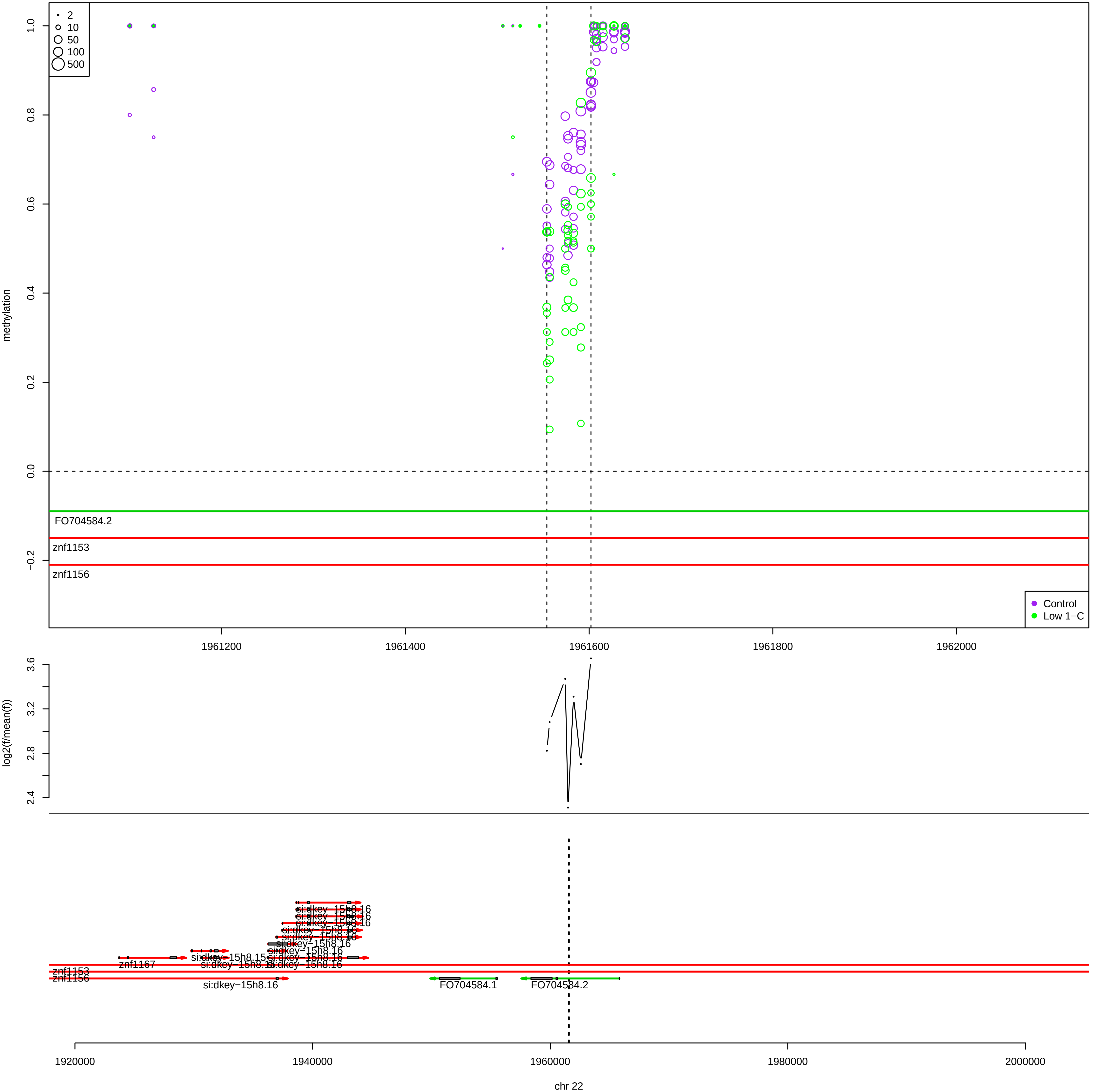

2
10
50
100
500
methylation
1.0
0.8
0.6
0.4
0.2
0.0
−0.2
FO704584.2
znf1153
znf1156
Control
Low 1−C
1961200
1961400
1961600
1961800
1962000
log2(f/mean(f))
3.6
3.2
2.8
2.4
si:dkey−15h8.16
znf1167
si:dkey−15h8.15
znf1153
znf1156
FO704584.1
FO704584.2
1920000
1940000
1960000
1980000
2000000
chr 22

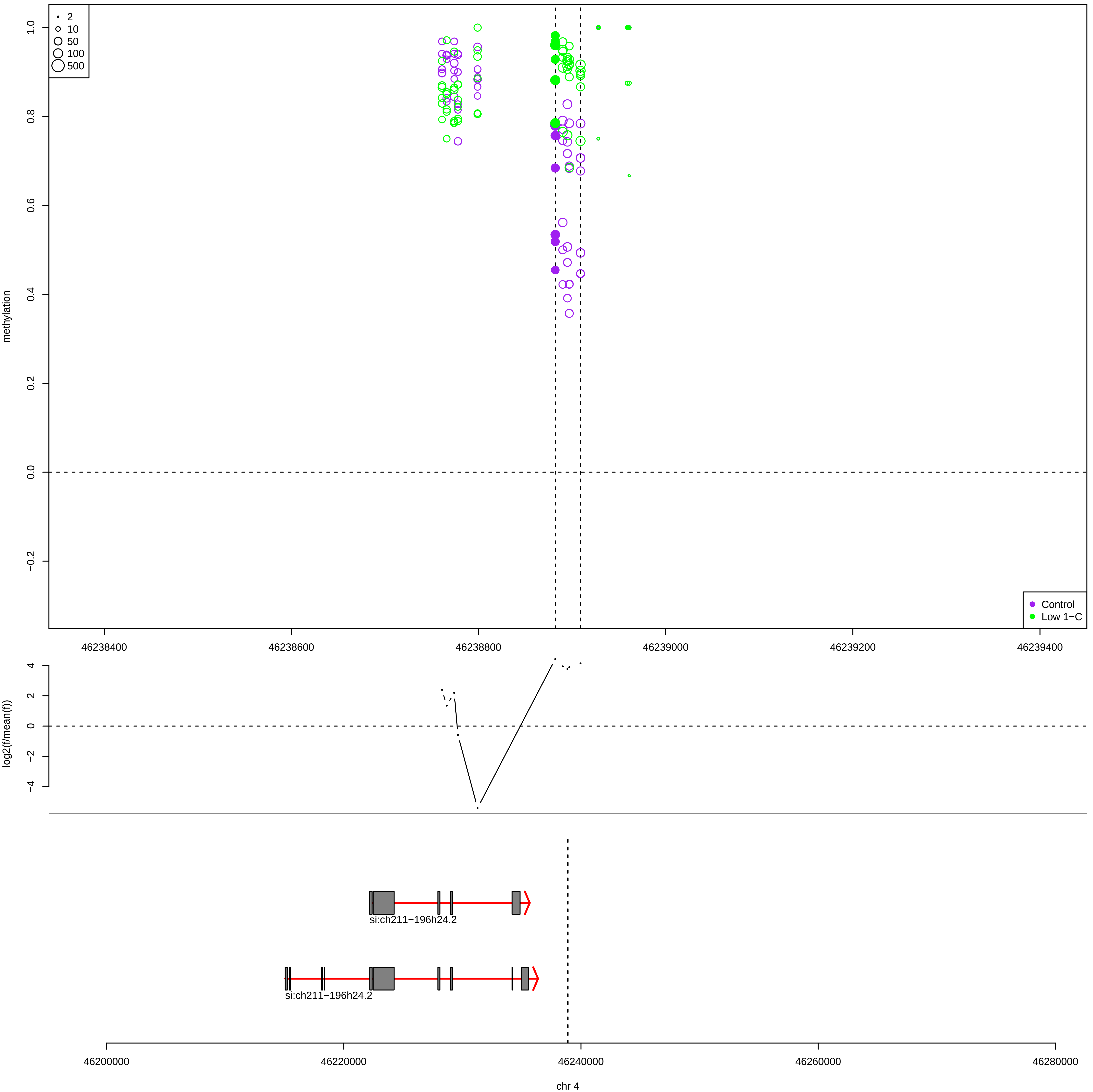

2
10
50
100
500
methylation
1.0
0.8
0.6
0.4
0.2
0.0
−0.2
Control
Low 1−C
46238400
46238600
46238800
46239000
46239200
46239400
log2(f/mean(f))
4
2
0
−2
−4
si:ch211−196h24.2
si:ch211−196h24.2
46200000
46220000
46240000
46260000
46280000
chr 4

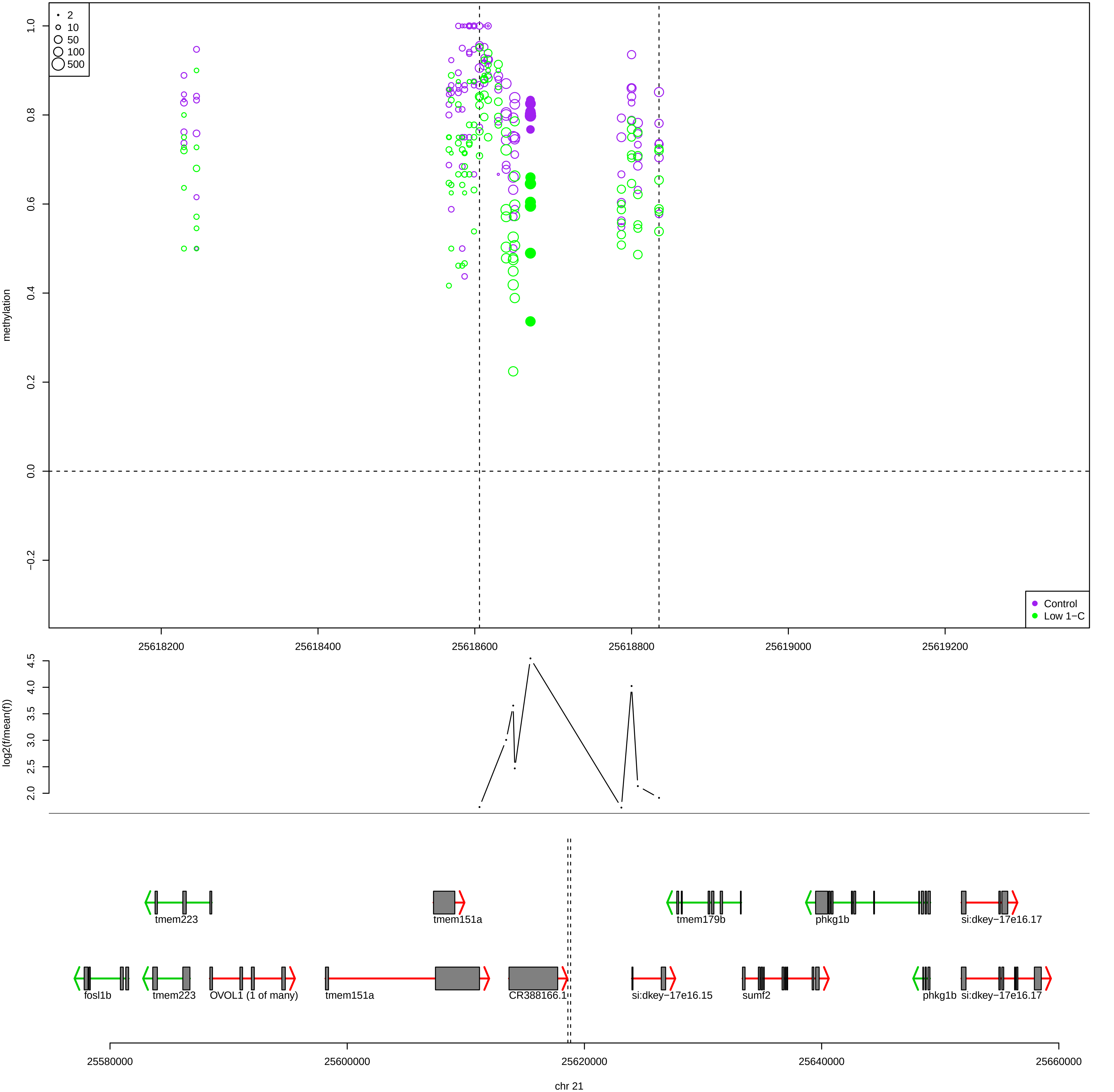

2
10
50
100
500
methylation
1.0
0.8
0.6
0.4
0.2
0.0
−0.2
Control
Low 1−C
25618200
25618400
25618600
25618800
25619000
25619200
log2(f/mean(f))
4.5
4.0
3.5
3.0
2.5
2.0
tmem223
tmem151a
tmem179b
phkg1b
si:dkey−17e16.17
fosl1b
tmem223
OVOL1 (1 of many)
tmem151a
CR388166.1
si:dkey−17e16.15
sumf2
phkg1b
si:dkey−17e16.17
25580000
25600000
25620000
25640000
25660000
chr 21

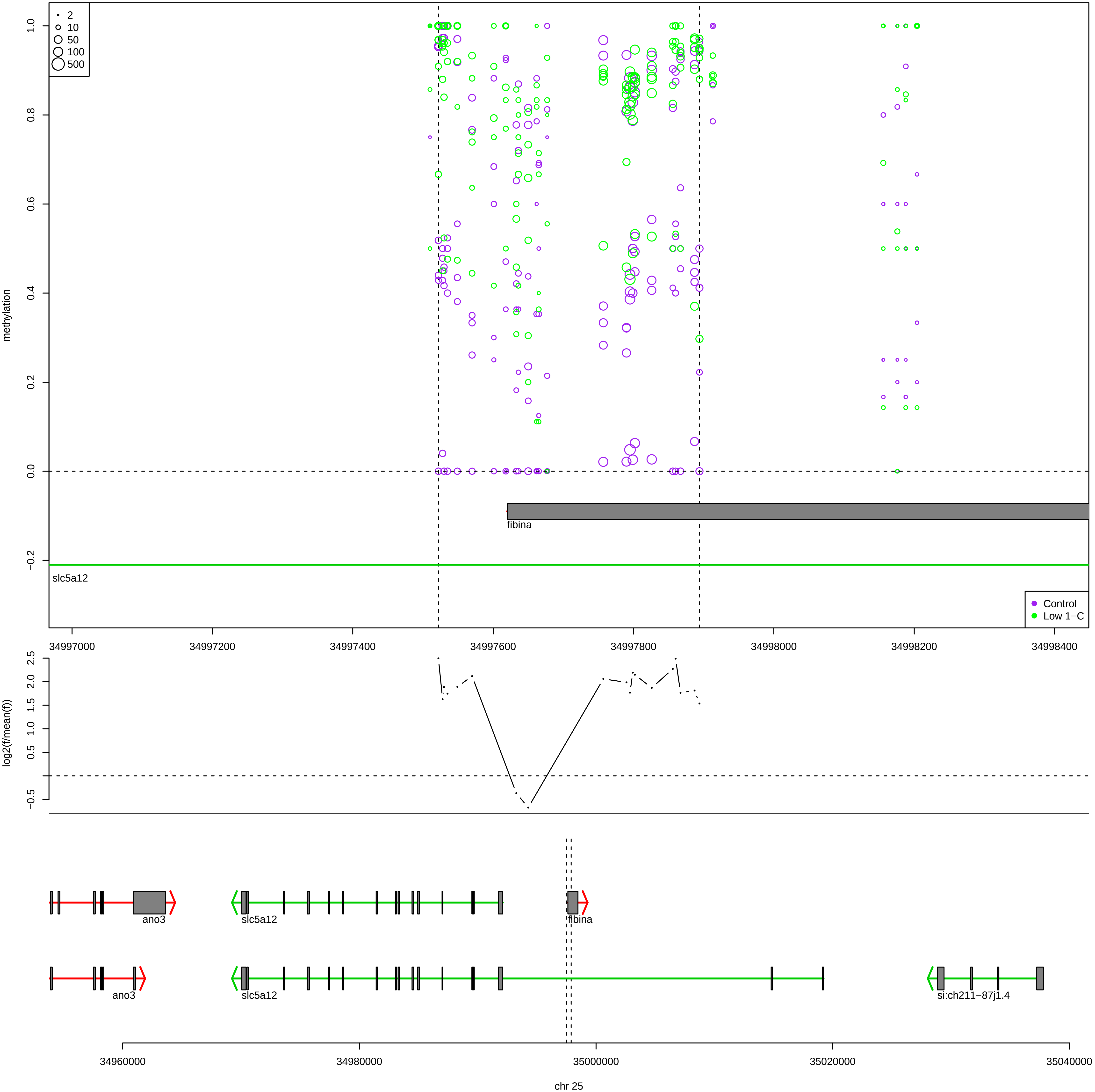

2
10
50
100
500
methylation
1.0
0.8
0.6
0.4
0.2
0.0
−0.2
fibina
slc5a12
Control
Low 1−C
34997000
34997200
34997400
34997600
34997800
34998000
34998200
34998400
log2(f/mean(f))
2.5
2.0
1.5
1.0
0.5
−0.5
ano3
slc5a12
fibina
ano3
slc5a12
si:ch211−87j1.4
34960000
34980000
35000000
35020000
35040000
chr 25

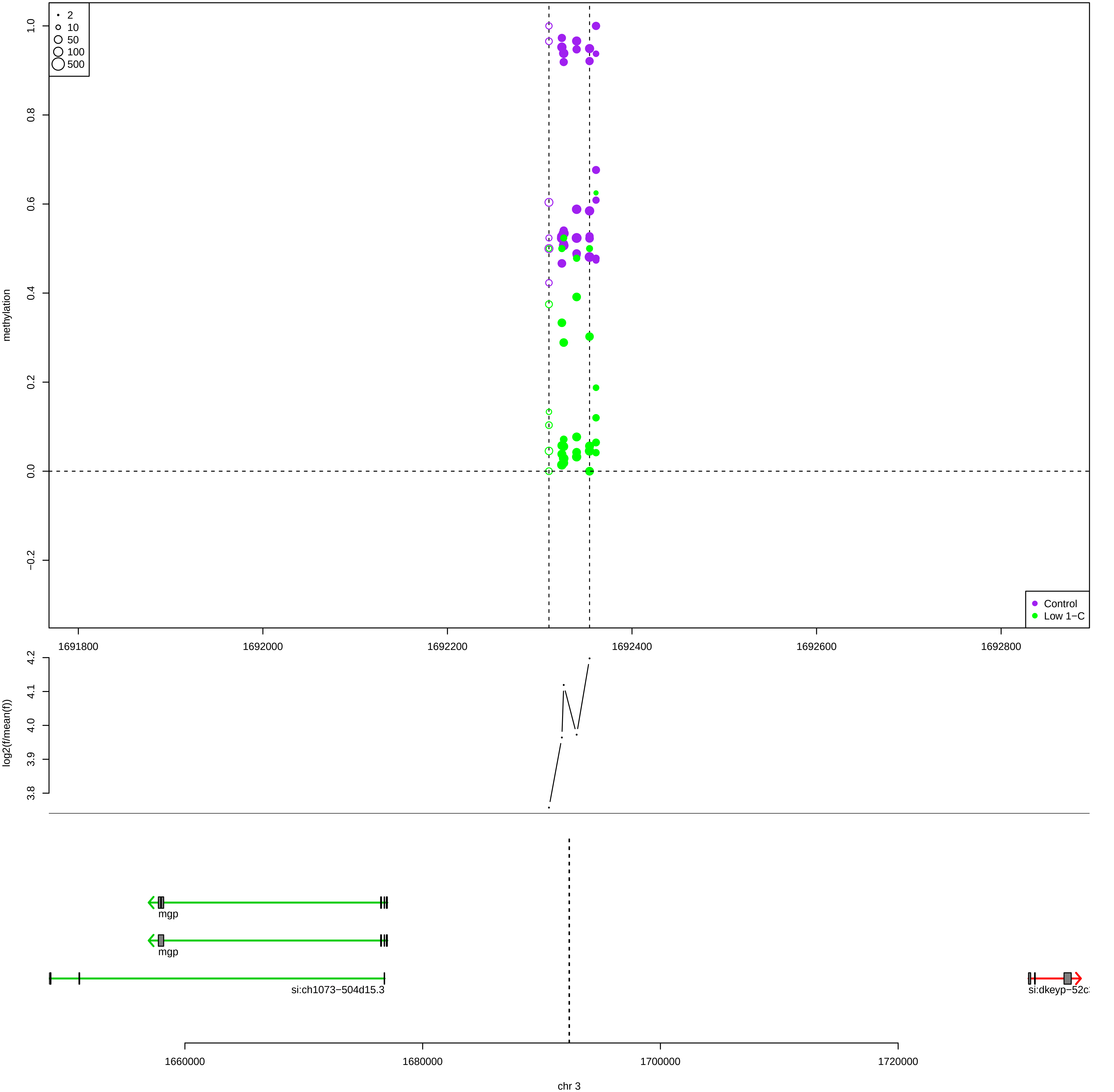
2
10
50
100
500
Control
Low 1−C
methylation
1.0
0.8
0.6
0.4
0.2
0.0
−0.2
1691800
1692000
1692200
1692400
1692600
1692800
log2(f/mean(f))
4.2
4.1
4.0
3.9
3.8
mgp
mgp
si:ch1073−504d15.3
1660000
1680000
1700000
1720000
chr 3

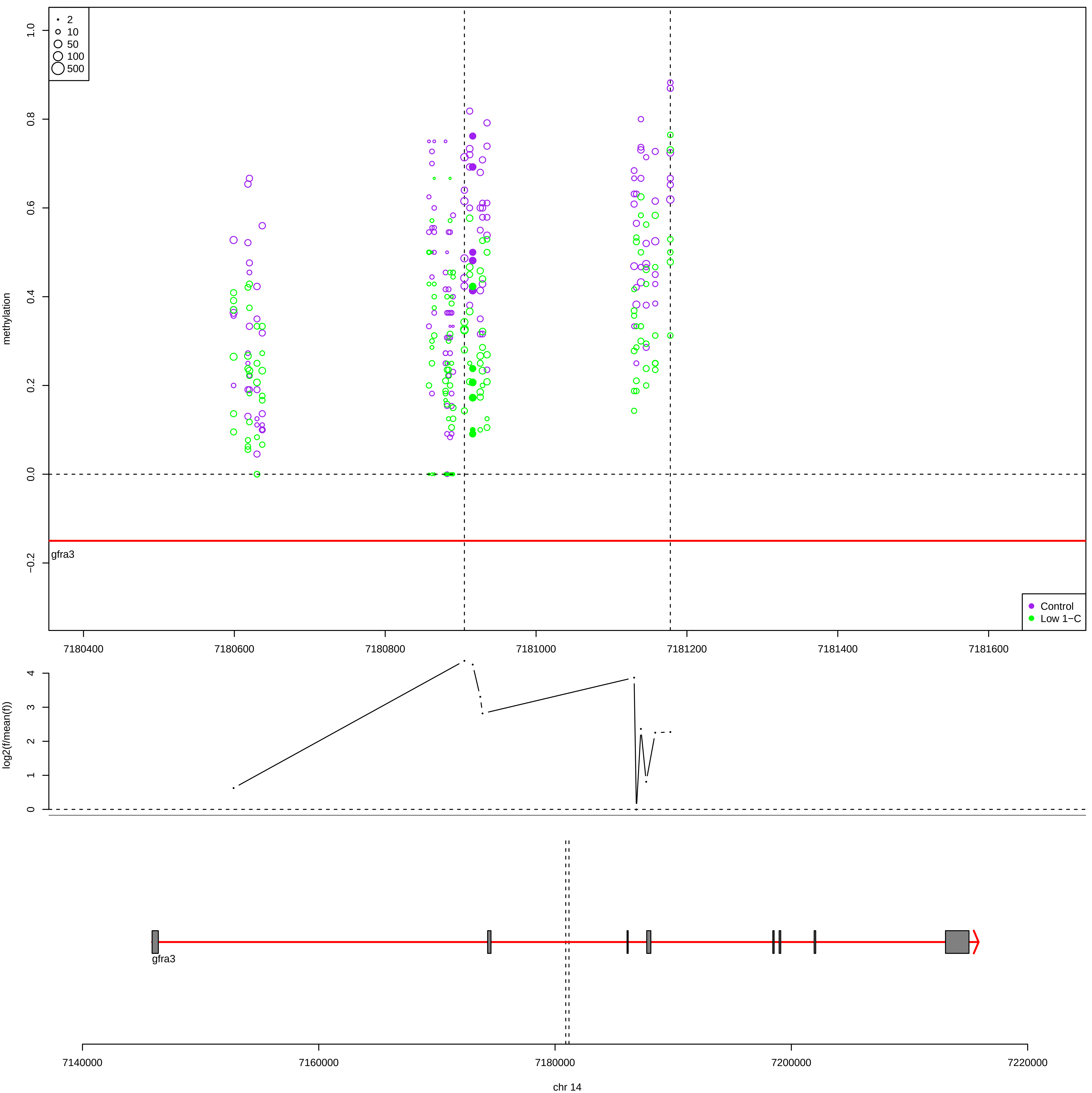

methylation
1.0
0.8
0.6
0.4
0.2
0.0
−0.2
2
10
50
100
500
gfra3
Control
Low 1−C
7180400
7180600
7180800
7181000
7181200
7181400
7181600
log2(f/mean(f))
4
3
2
1
0
gfra3
7140000
7160000
7180000
7200000
7220000
chr 14

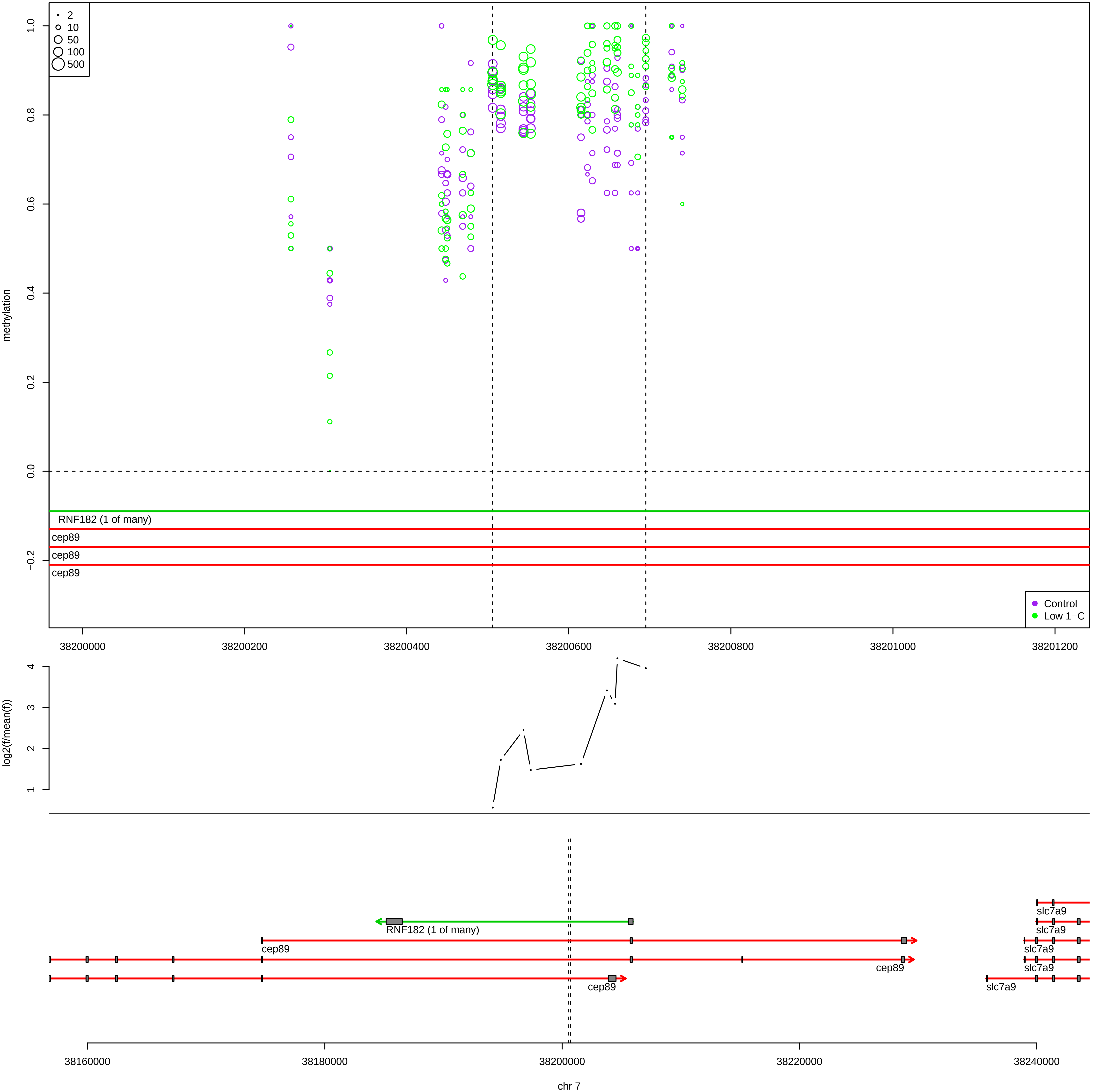

2
10
50
100
500
methylation
1.0
0.8
0.6
0.4
0.2
0.0
−0.2
RNF182 (1 of many)
cep89
cep89
cep89
Control
Low 1−C
38200000
38200200
38200400
38200600
38200800
38201000
38201200
log2(f/mean(f))
4
3
2
1
RNF182 (1 of many)
cep89
cep89
cep89
slc7a9
slc7a9
slc7a9
slc7a9
slc7a9
38160000
38180000
38200000
38220000
38240000
chr 7

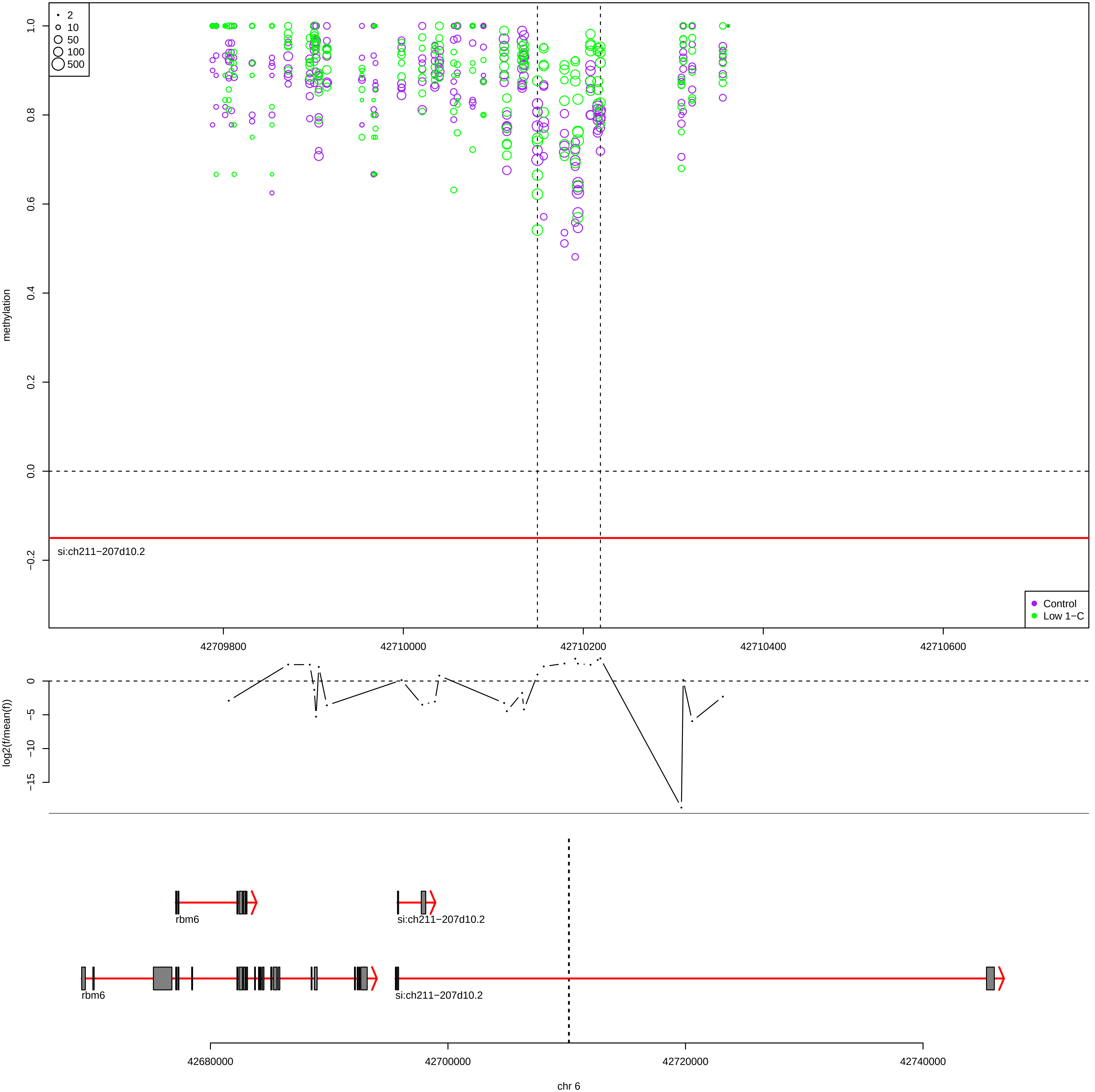

2
10
50
100
500
methylation
1.0
0.8
0.6
0.4
0.2
0.0
−0.2
si:ch211−207d10.2
Control
Low 1−C
42709800
42710000
42710200
42710400
42710600
log2(f/mean(f))
0
−5
−10
−15
rbm6
si:ch211−207d10.2
rbm6
si:ch211−207d10.2
42680000
42700000
42720000
42740000
chr 6

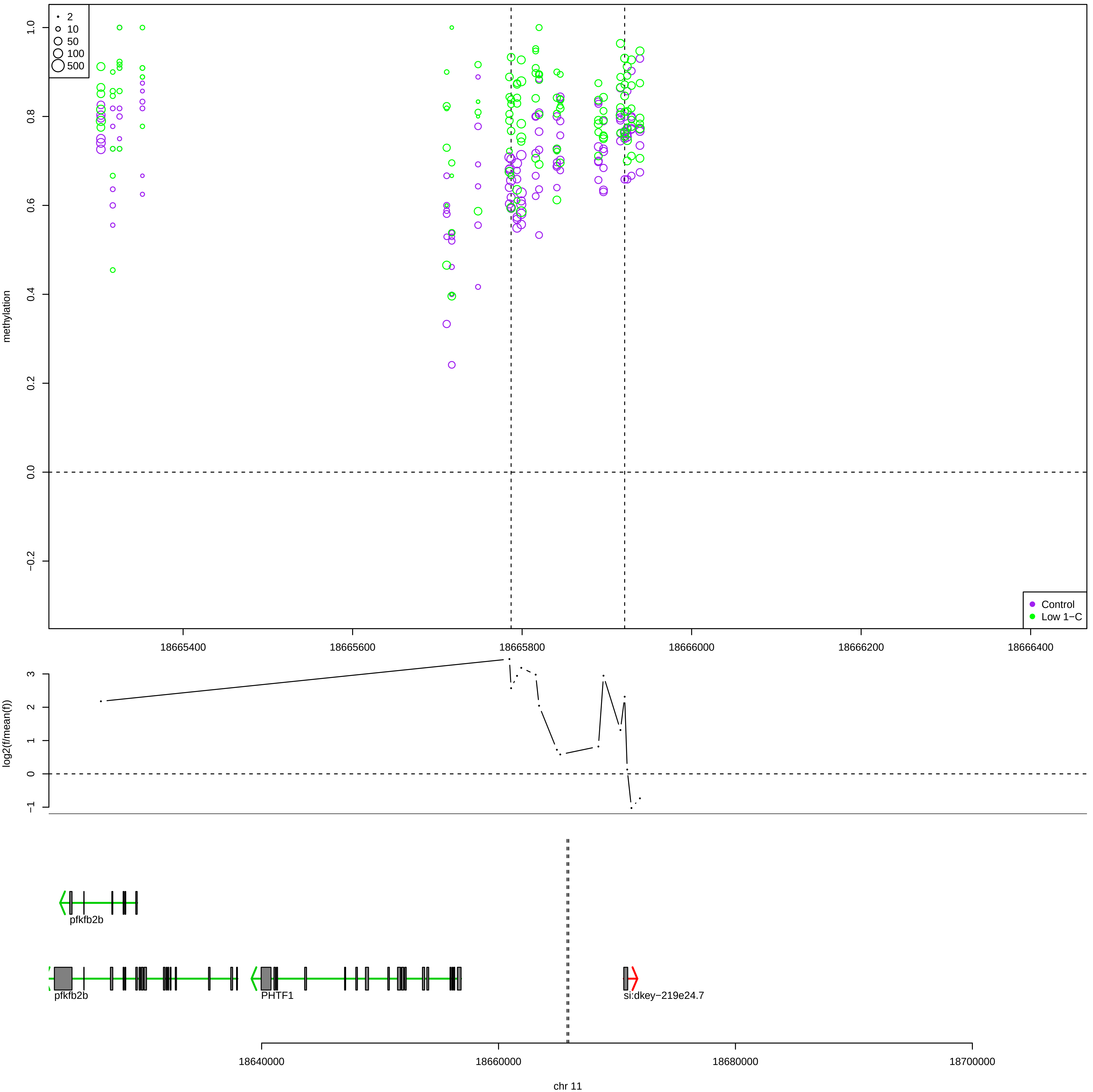

2
10
50
100
500
methylation
1.0
0.8
0.6
0.4
0.2
0.0
−0.2
Control
Low 1−C
18665400
18665600
18665800
18666000
18666200
18666400
log2(f/mean(f))
3
2
1
0
−1
pfkfb2b
pfkfb2b
PHTF1
si:dkey−219e24.7
18640000
18660000
18680000
18700000
chr 11

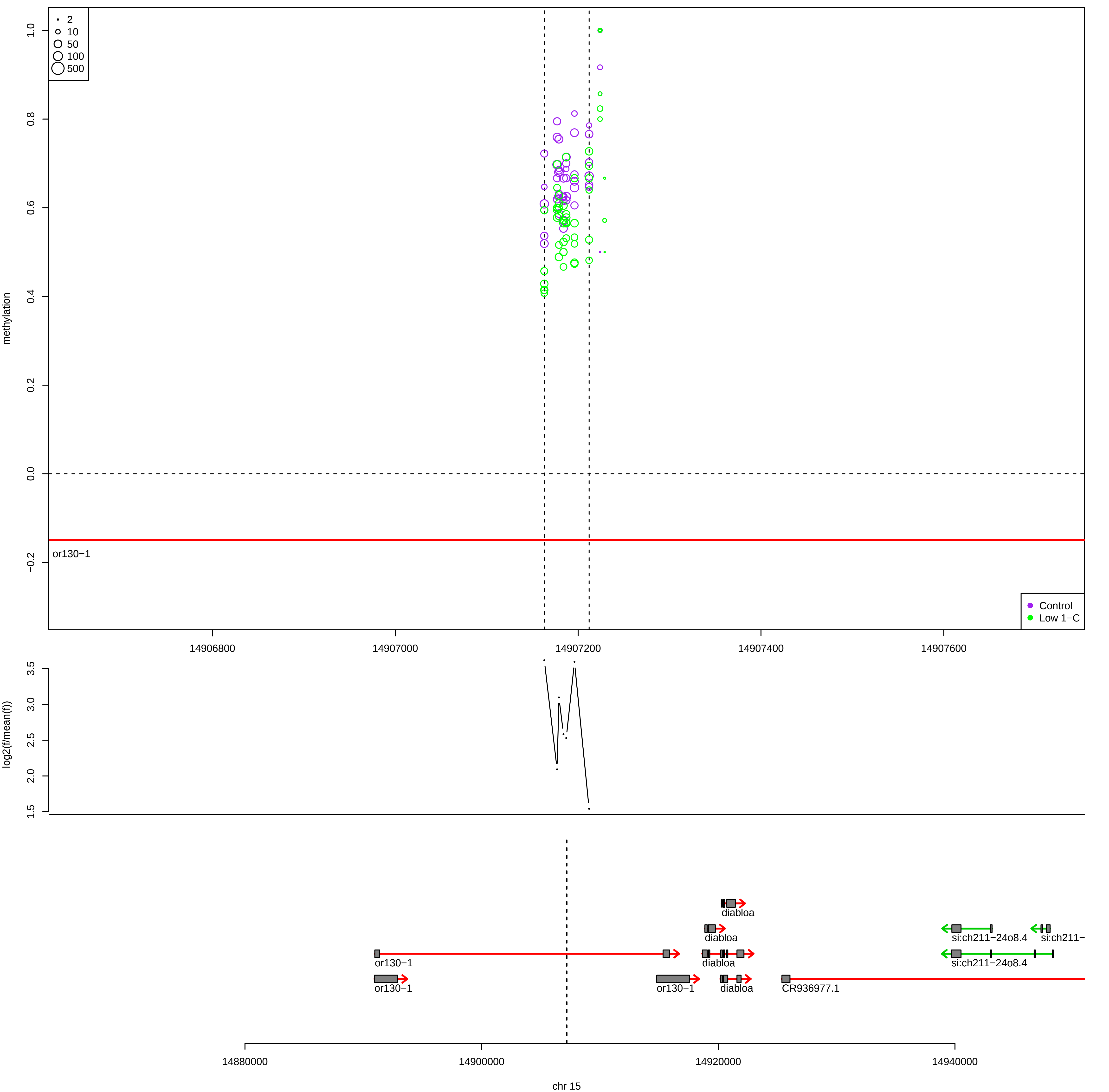

2
10
50
100
500
methylation
1.0
0.8
0.6
0.4
0.2
0.0
−0.2
or130−1
Control
Low 1−C
14906800
14907000
14907200
14907400
14907600
log2(f/mean(f))
3.5
3.0
2.5
2.0
1.5
diabloa
diabloa
si:ch211−24o8.4
si:ch211−
or130−1
diabloa
si:ch211−24o8.4
or130−1
or130−1
diabloa
CR936977.1
14880000
14900000
14920000
14940000
chr 15

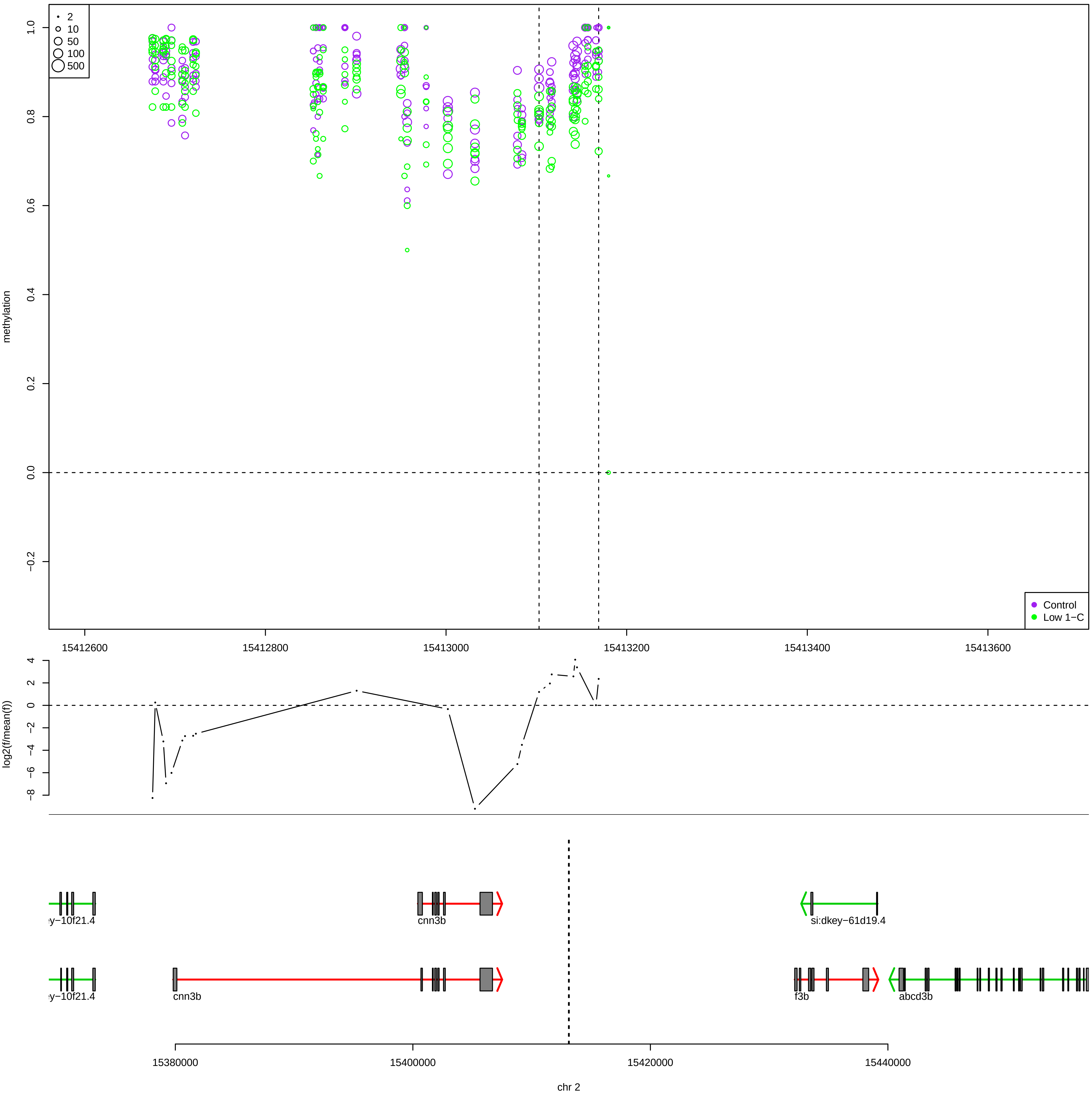

2
10
50
100
500
Control
Low 1−C
methylation
1.0
0.8
0.6
0.4
0.2
0.0
−0.2
15412600
15412800
15413000
15413200
15413400
15413600
log2(f/mean(f))
4
2
0
−2
−4
−6
−8
y−10f21.4
cnn3b
si:dkey−61d19.4
y−10f21.4
cnn3b
f3b
abcd3b
15380000
15400000
15420000
15440000
chr 2

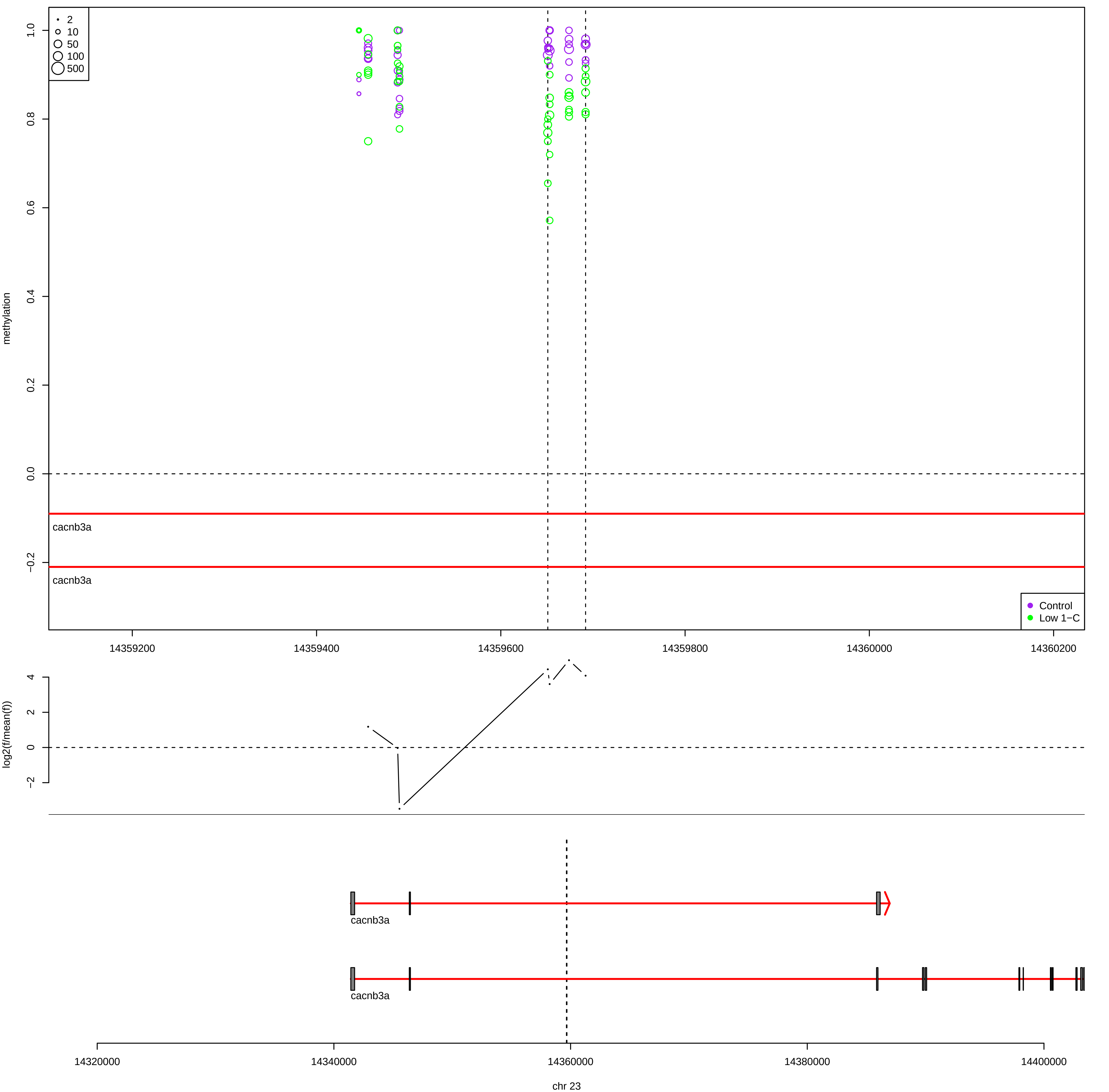

2
10
50
100
500
methylation
1.0
0.8
0.6
0.4
0.2
0.0
−0.2
cacnb3a
cacnb3a
Control
Low 1−C
14359200
14359400
14359600
14359800
14360000
14360200
log2(f/mean(f))
4
2
0
−2
cacnb3a
cacnb3a
14320000
14340000
14360000
14380000
14400000
chr 23

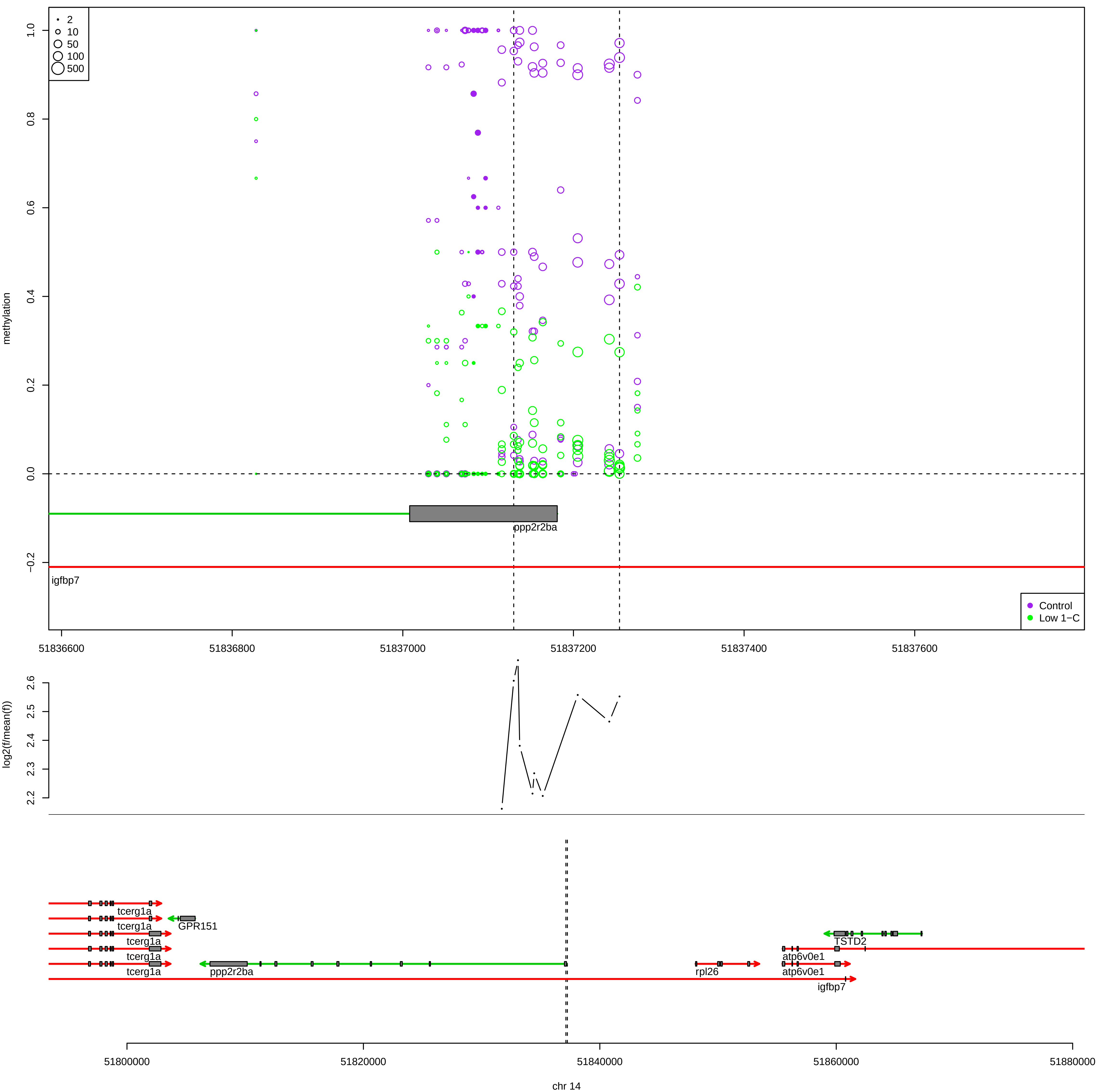

2
10
50
100
500
methylation
1.0
0.8
0.6
0.4
0.2
0.0
−0.2
ppp2r2ba
igfbp7
Control
Low 1−C
51836600
51836800
51837000
51837200
51837400
51837600
log2(f/mean(f))
2.6
2.5
2.4
2.3
2.2
tcerg1a
tcerg1a
GPR151
tcerg1a
tcerg1a
tcerg1a
ppp2r2ba
rpl26
TSTD2
atp6v0e1
atp6v0e1
igfbp7
51800000
51820000
51840000
51860000
51880000
chr 14

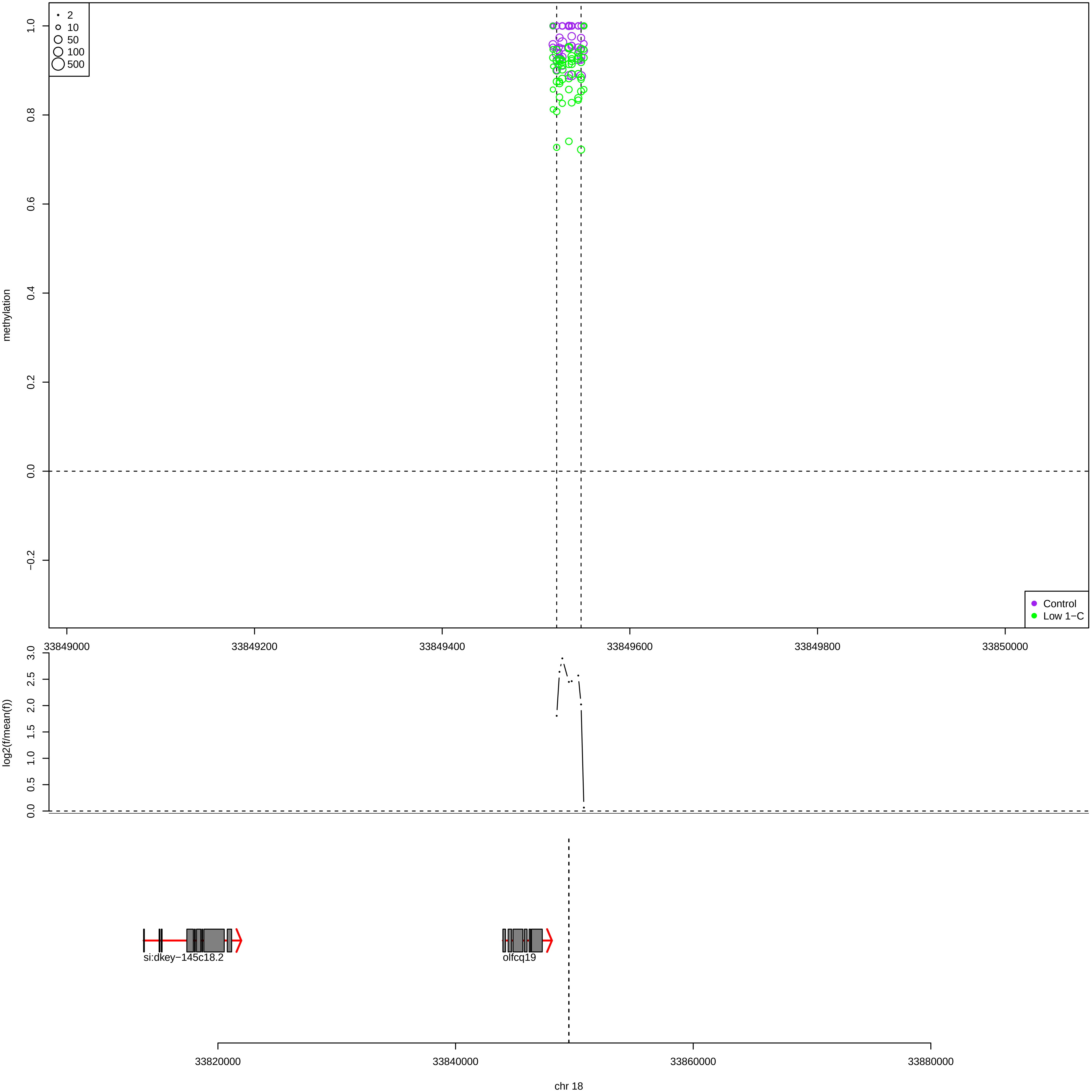

2
10
50
100
500
methylation
1.0
0.8
0.6
0.4
0.2
0.0
−0.2
Control
Low 1−C
33849000
33849200
33849400
33849600
33849800
33850000
log2(f/mean(f))
3.0
2.5
2.0
1.5
1.0
0.5
0.0
si:dkey−145c18.2
olfcq19
33820000
33840000
33860000
33880000
chr 18

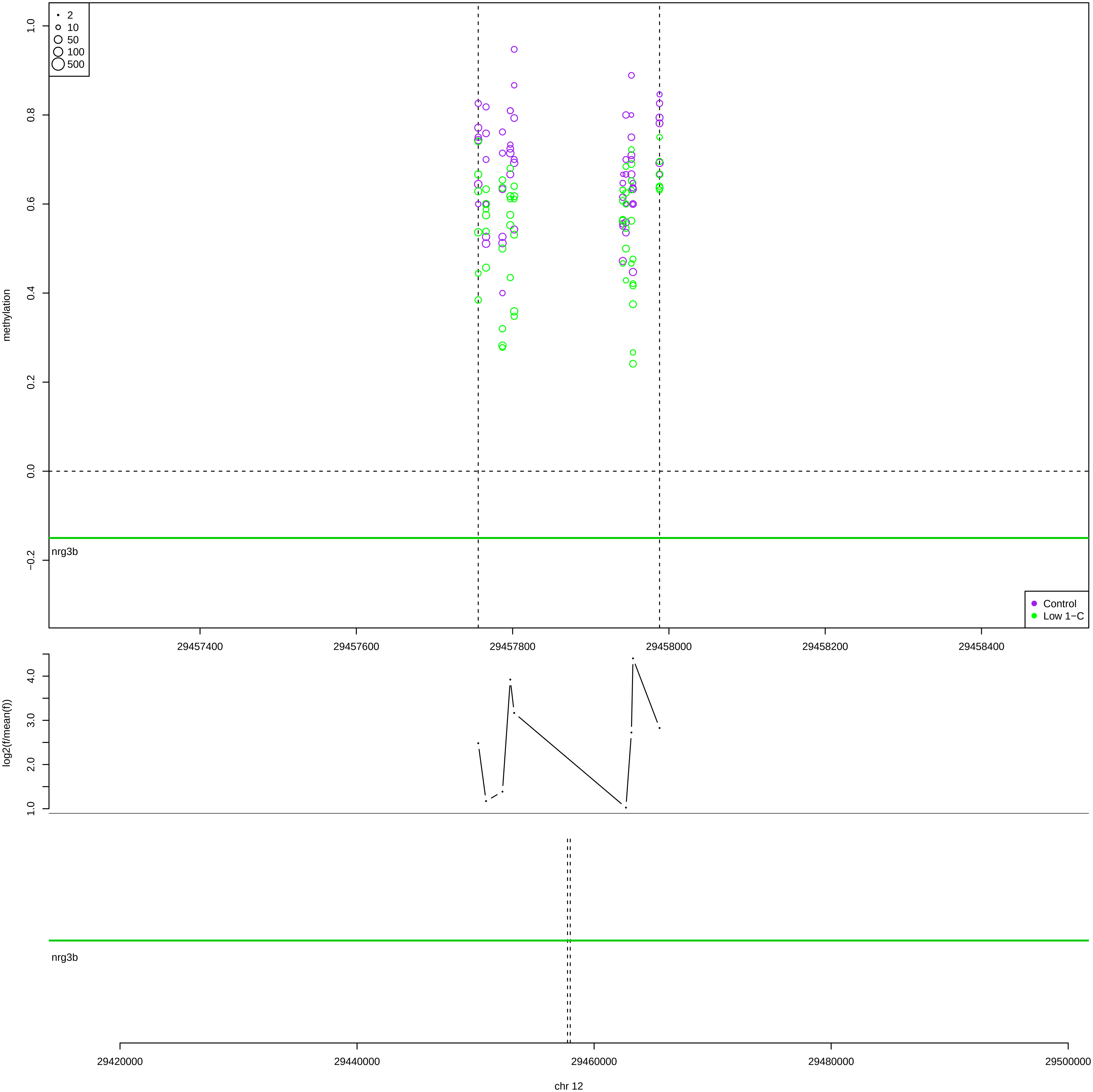

2
10
50
100
500
1.0
0.8
0.6
0.4
0.2
0.0
−0.2
methylation
nrg3b
Control
Low 1−C
29457400
29457600
29457800
29458000
29458200
29458400
4.0
3.0
2.0
1.0
log2(f/mean(f))
nrg3b
29420000
29440000
29460000
29480000
29500000
chr 12

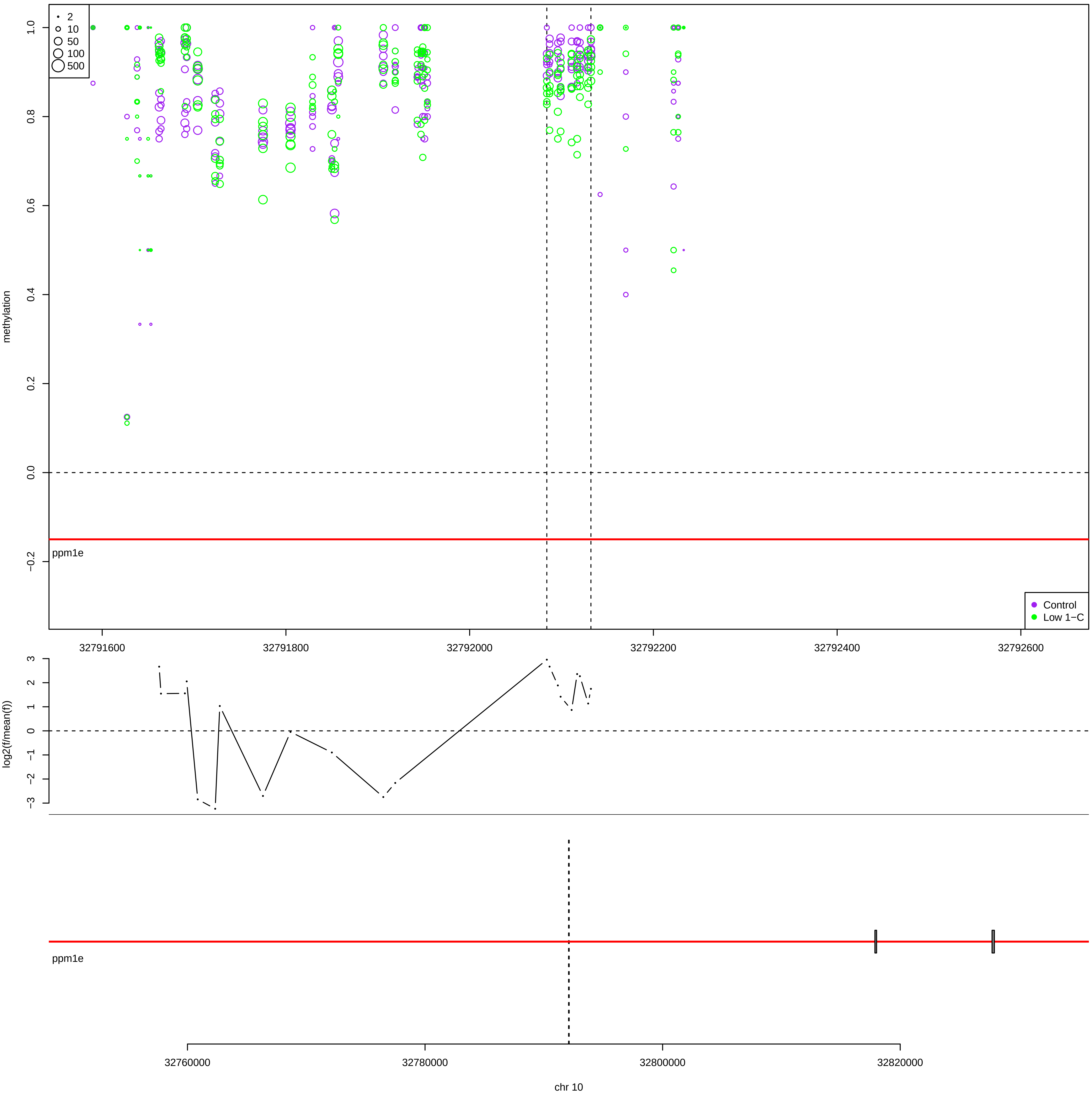

methylation
1.0
0.8
0.6
0.4
0.2
0.0
−0.2
2
10
50
100
500
ppm1e
Control
Low 1−C
32791600
32791800
32792000
32792200
32792400
32792600
log2(f/mean(f))
3
2
1
0
−1
−2
−3
ppm1e
32760000
32780000
32800000
32820000
chr 10

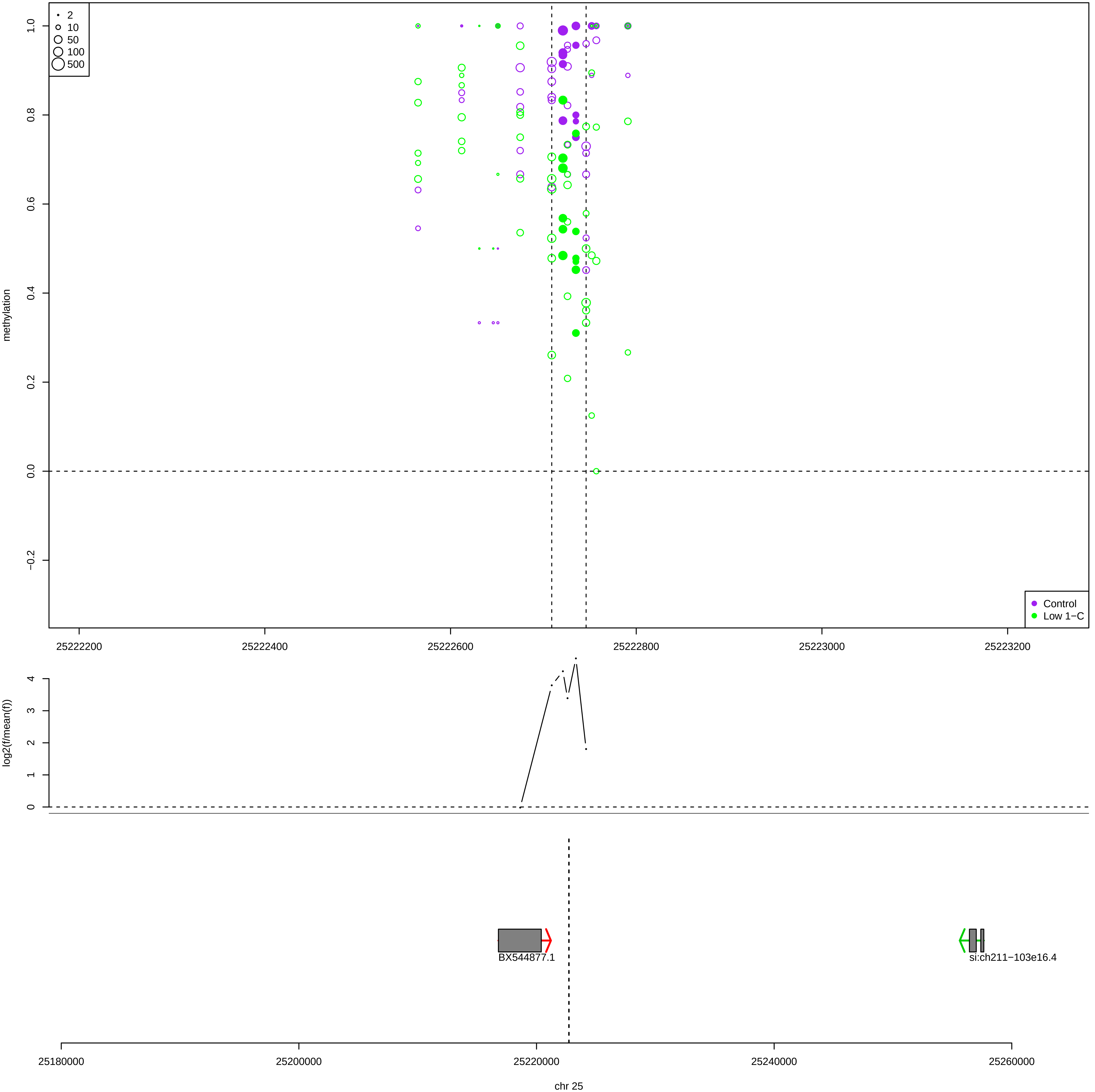

2
10
50
100
500
methylation
Control
Low 1−C
25222200
25222400
25222600
25222800
25223000
25223200
log2(f/mean(f))
BX544877.1
si:ch211−103e16.4
25180000
25200000
25220000
25240000
25260000
chr 25

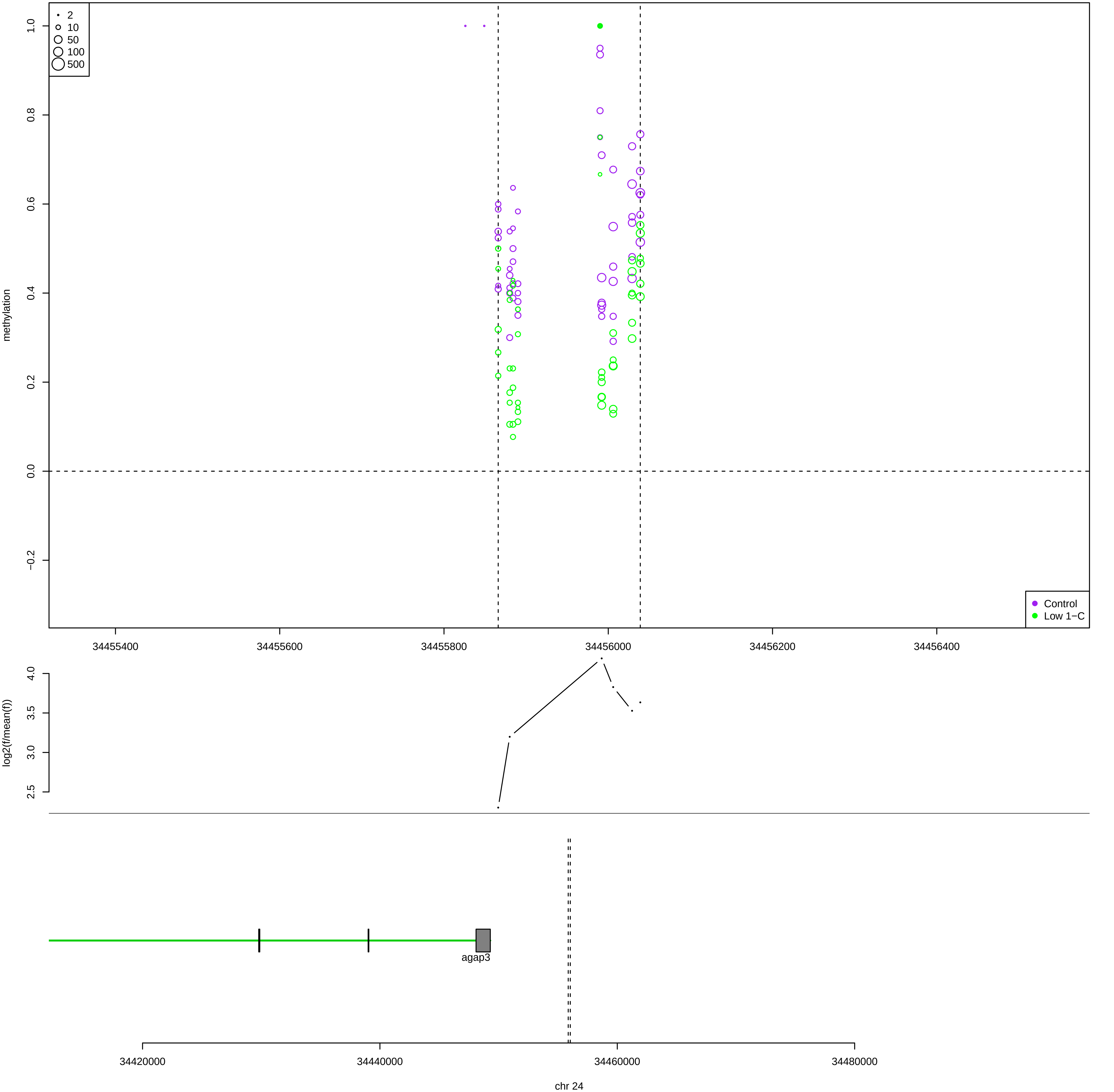

2
10
50
100
500
methylation
1.0
0.8
0.6
0.4
0.2
0.0
−0.2
Control
Low 1−C
34455400
34455600
34455800
34456000
34456200
34456400
log2(f/mean(f))
4.0
3.5
3.0
2.5
agap3
34420000
34440000
34460000
34480000
chr 24

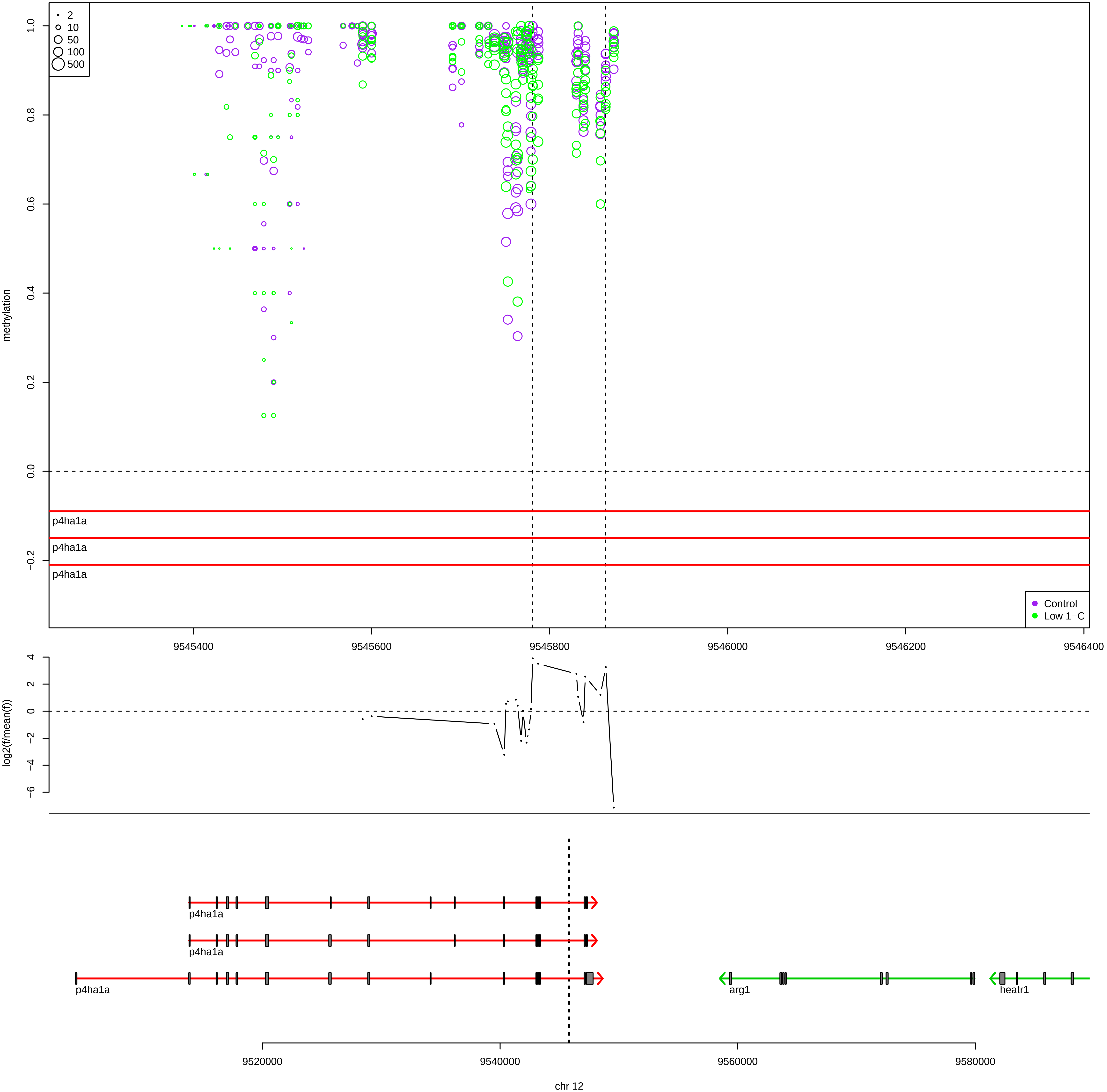

methylation
1.0
0.8
0.6
0.4
0.2
0.0
−0.2
2
10
50
100
500
p4ha1a
p4ha1a
p4ha1a
Control
Low 1−C
9545400
9545600
9545800
9546000
9546200
9546400
log2(f/mean(f))
4
2
0
−2
−4
−6
p4ha1a
p4ha1a
p4ha1a
arg1
heatr1
9520000
9540000
9560000
9580000
chr 12

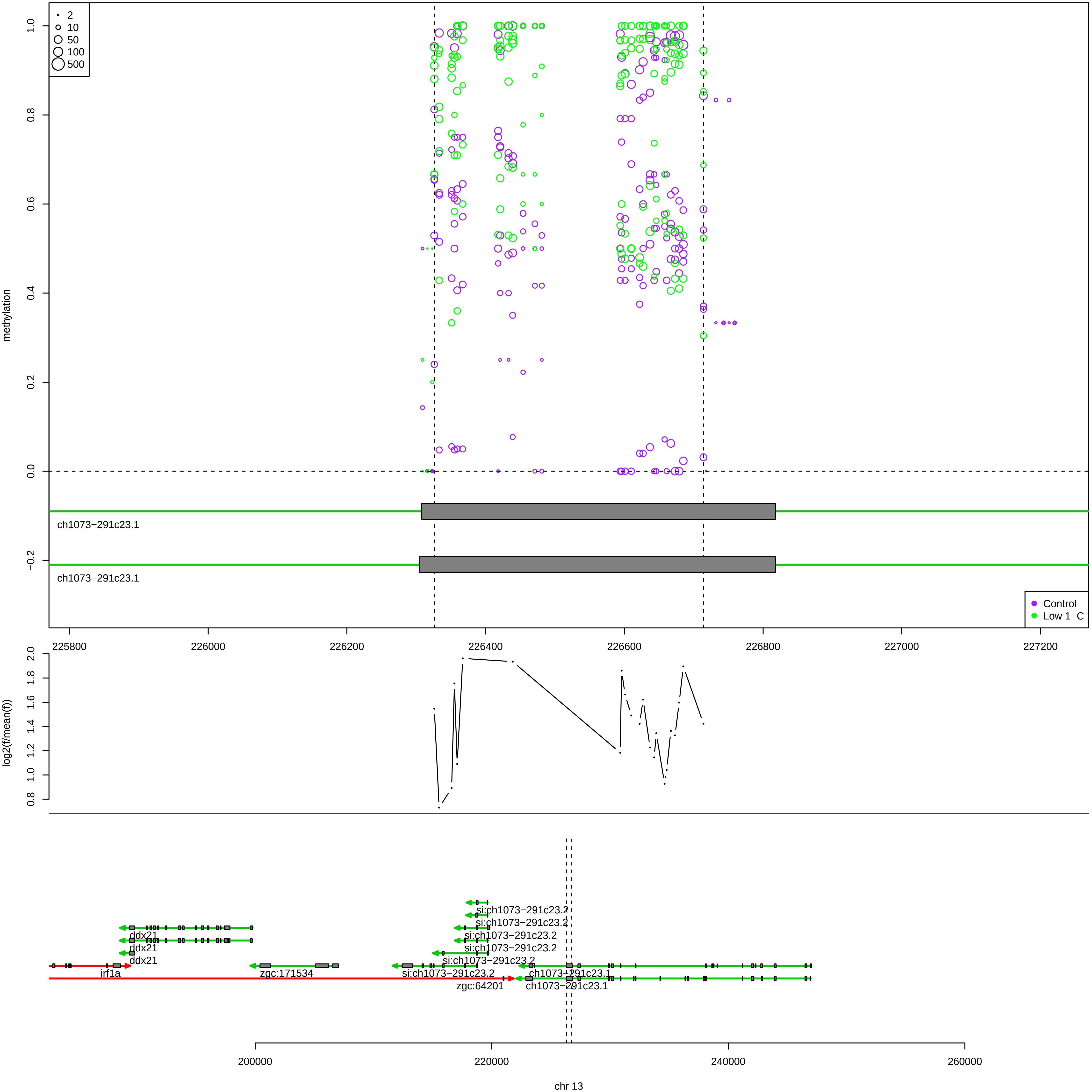

2
10
50
100
500
methylation
Control
Low 1−C
ch1073−291c23.1
ch1073−291c23.1
log2(f/mean(f))
ddx21
ddx21
ddx21
irf1a
zgc:171534
si:ch1073−291c23.2
si:ch1073−291c23.2
si:ch1073−291c23.2
si:ch1073−291c23.2
si:ch1073−291c23.2
si:ch1073−291c23.2
zgc:64201
ch1073−291c23.1
ch1073−291c23.1
chr 13

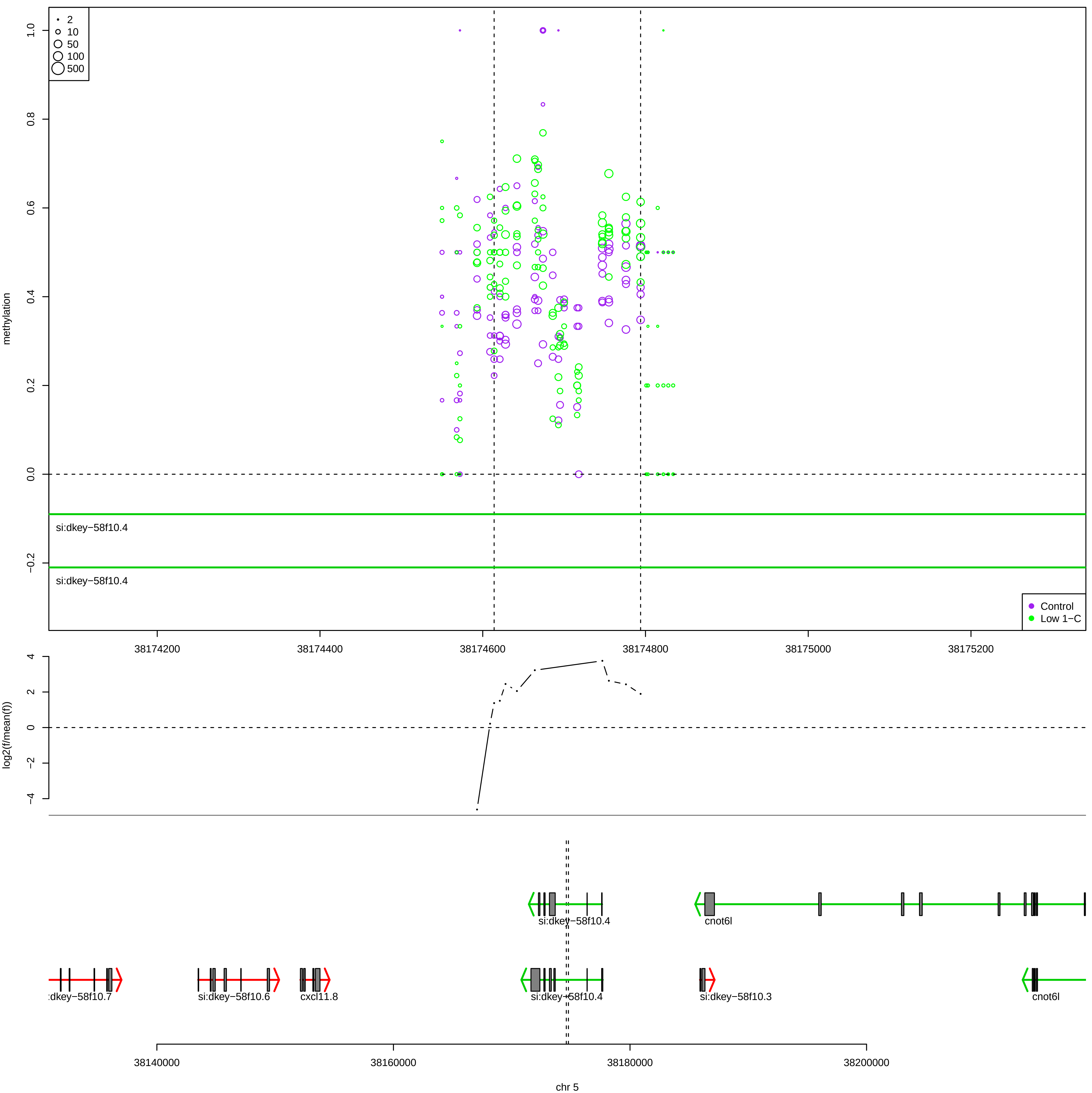

2
10
50
100
500
Control
Low 1−C
methylation
1.0
0.8
0.6
0.4
0.2
0.0
−0.2
si:dkey−58f10.4
si:dkey−58f10.4
38174200
38174400
38174600
38174800
38175000
38175200
log2(f/mean(f))
4
2
0
−2
−4
si:dkey−58f10.4
cnot6l
:dkey−58f10.7
si:dkey−58f10.6
cxcl11.8
si:dkey−58f10.4
si:dkey−58f10.3
cnot6l
38140000
38160000
38180000
38200000
chr 5

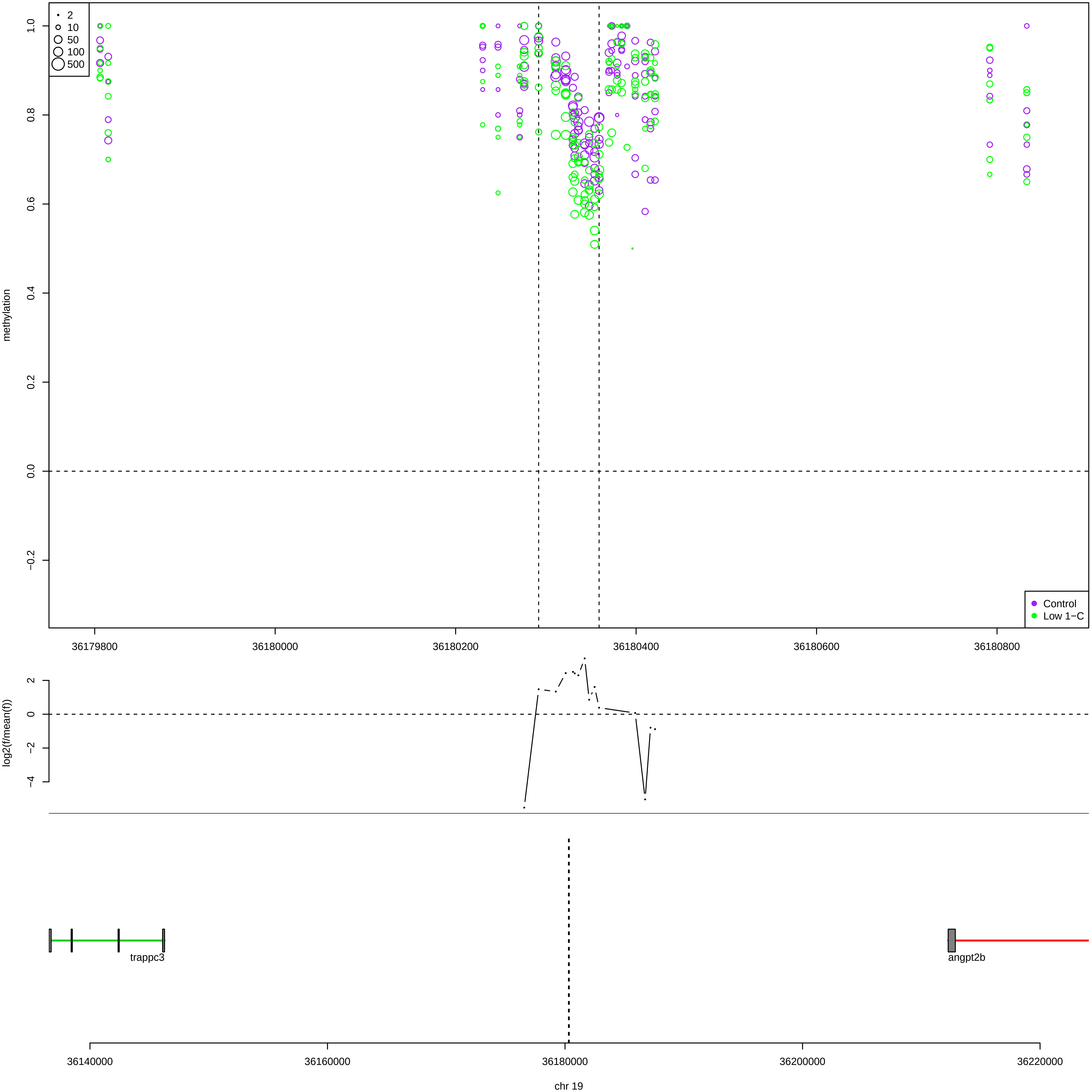

2
10
50
100
500
methylation
1.0
0.8
0.6
0.4
0.2
0.0
−0.2
Control
Low 1−C
36179800
36180000
36180200
36180400
36180600
36180800
log2(f/mean(f))
2
0
−2
−4
trappc3
angpt2b
36140000
36160000
36180000
36200000
36220000
chr 19

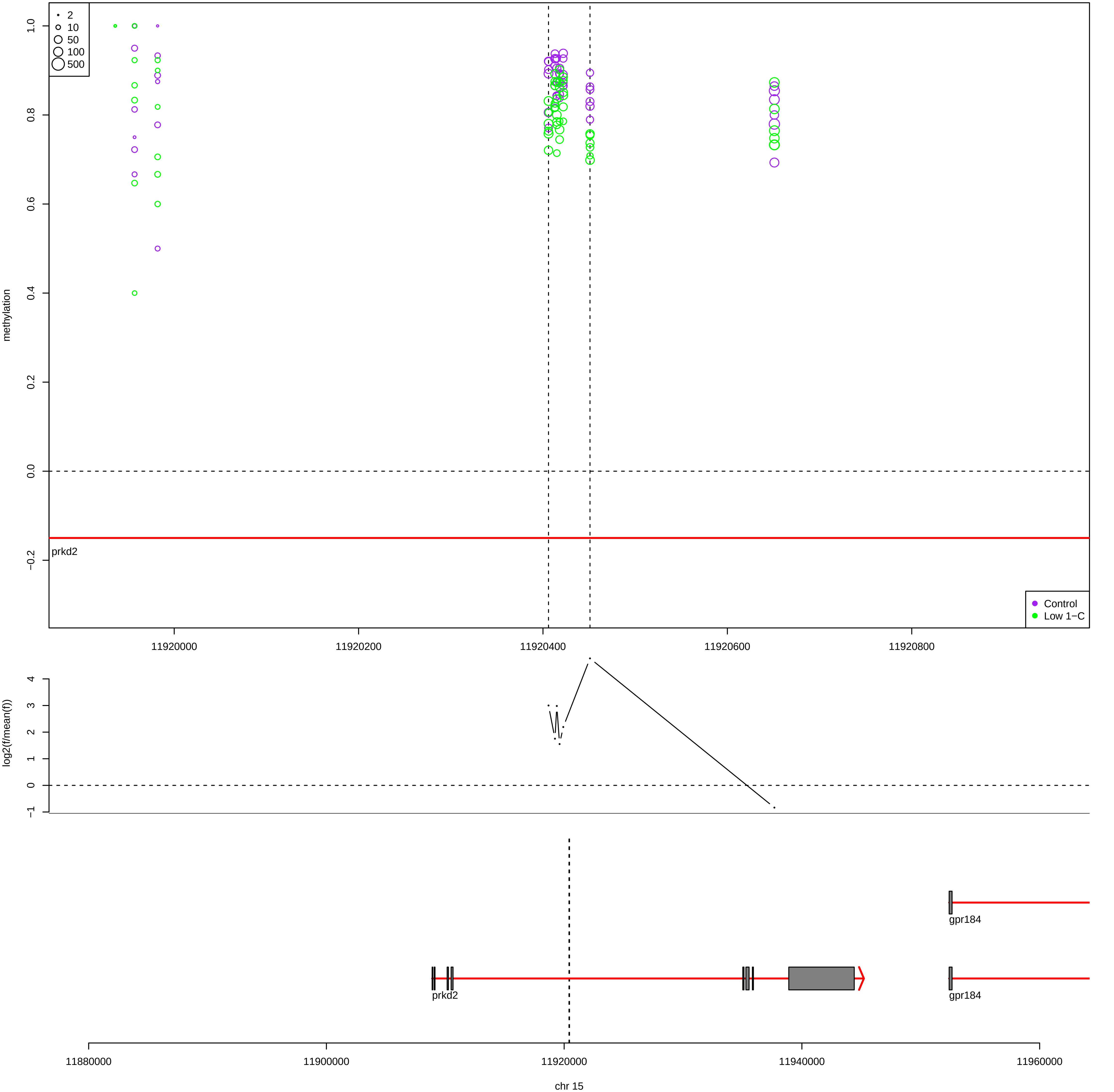

methylation
1.0
0.8
0.6
0.4
0.2
0.0
−0.2
2
10
50
100
500
prkd2
Control
Low 1−C
11920000
11920200
11920400
11920600
11920800
log2(f/mean(f))
4
3
2
1
0
−1
gpr184
prkd2
gpr184
11880000
11900000
11920000
11940000
11960000
chr 15

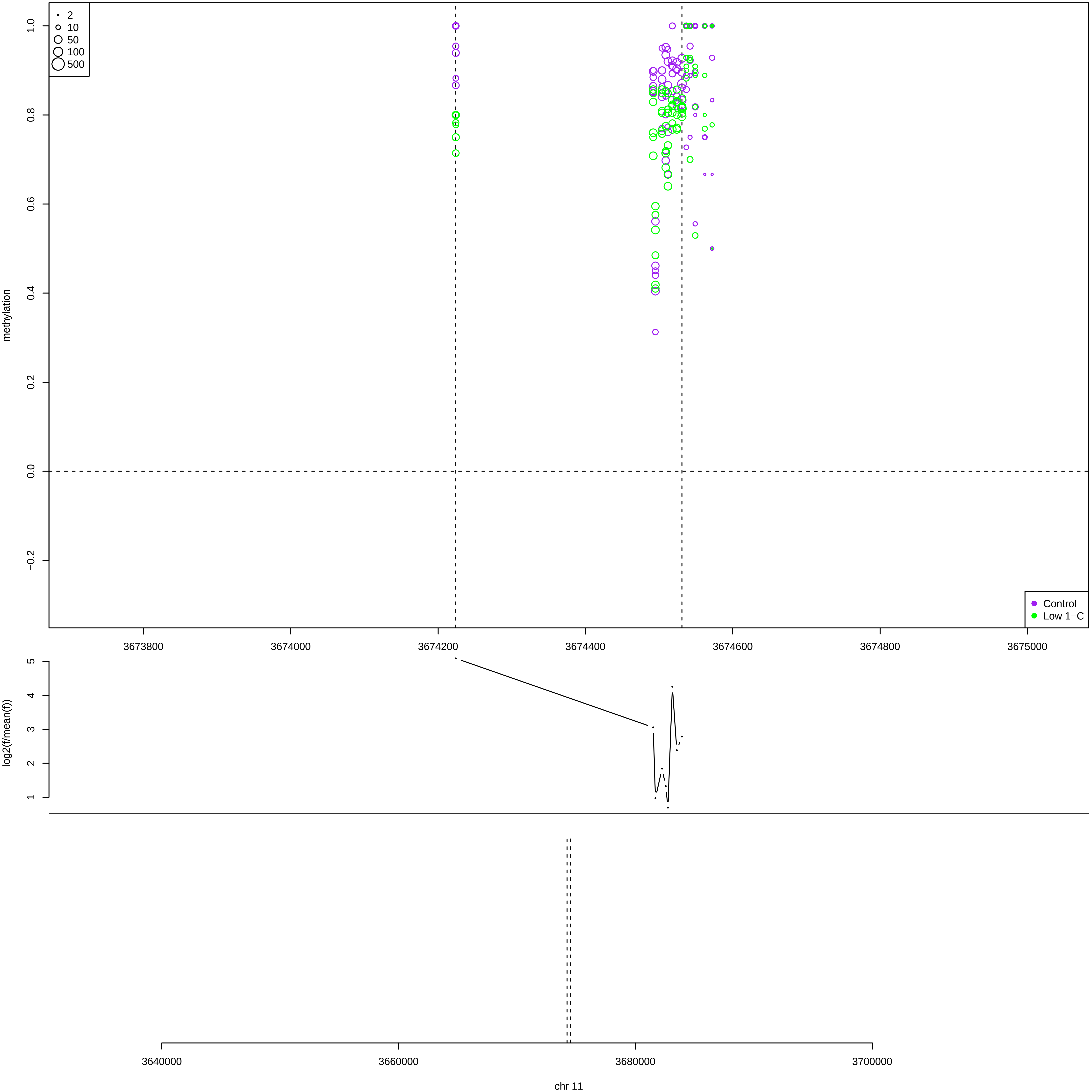

2
10
50
100
500
methylation
1.0
0.8
0.6
0.4
0.2
0.0
−0.2
Control
Low 1−C
3673800
3674000
3674200
3674400
3674600
3674800
3675000
log2(f/mean(f))
5
4
3
2
1
3640000
3660000
3680000
3700000
chr 11

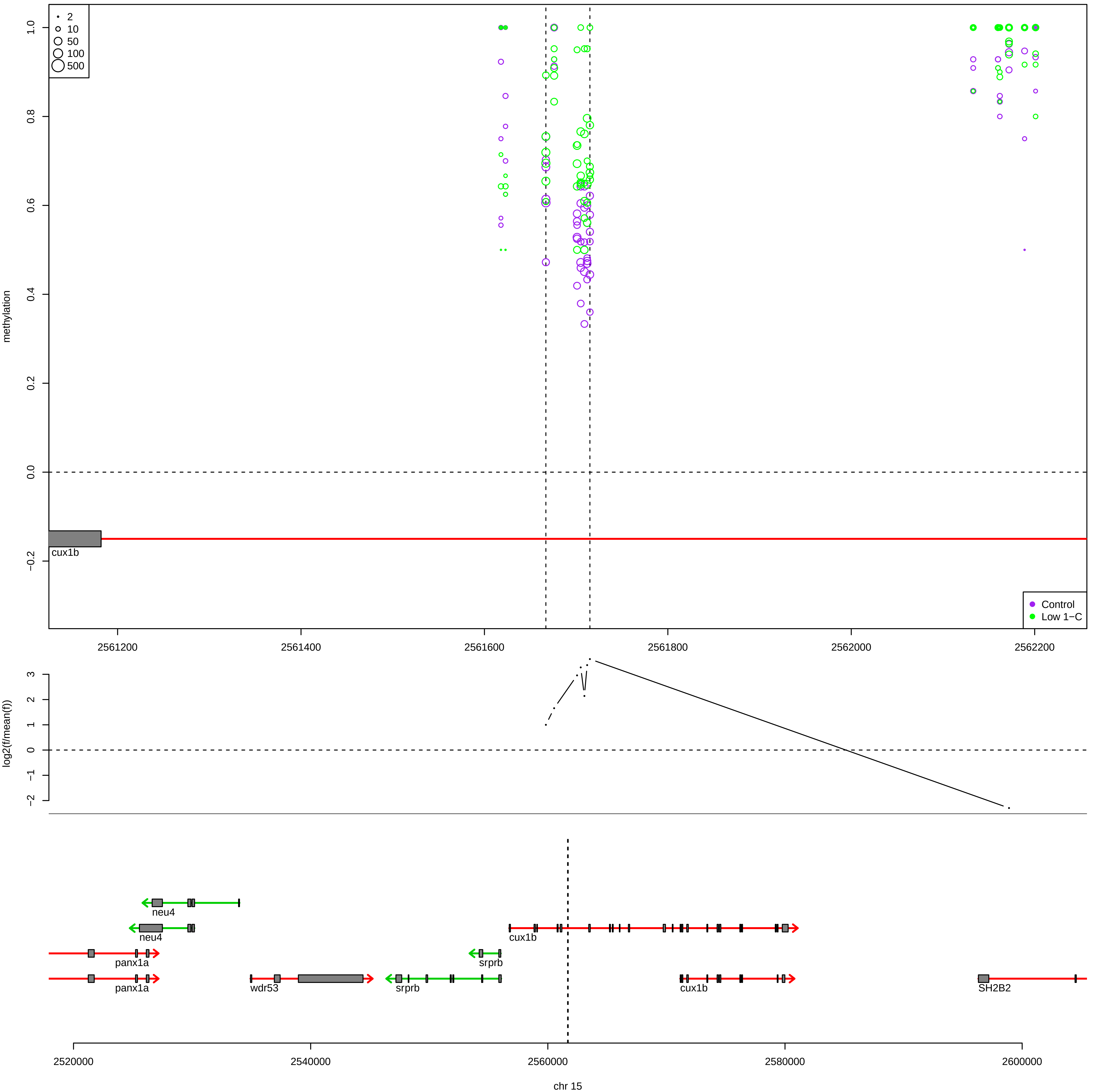

2
10
50
100
500
1.0
0.8
0.6
0.4
0.2
0.0
−0.2
methylation
cux1b
Control
Low 1−C
2561200
2561400
2561600
2561800
2562000
2562200
log2(f/mean(f))
3
2
1
0
−1
−2
neu4
neu4
cux1b
panx1a
srprb
panx1a
wdr53
srprb
cux1b
SH2B2
2520000
2540000
2560000
2580000
2600000
chr 15

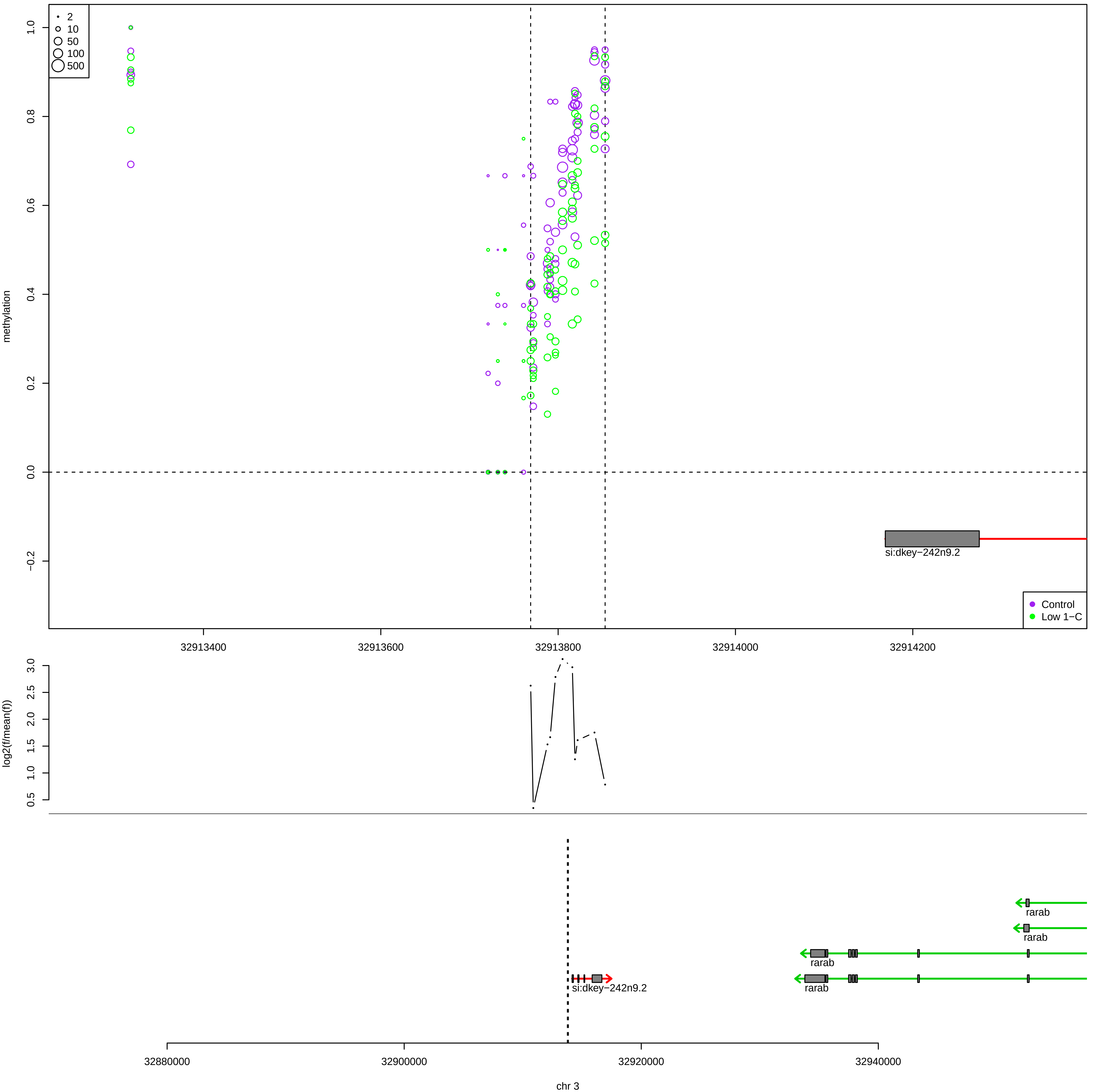

2
10
50
100
500
methylation
1.0
0.8
0.6
0.4
0.2
0.0
−0.2
si:dkey−242n9.2
Control
Low 1−C
32913400
32913600
32913800
32914000
32914200
log2(f/mean(f))
3.0
2.5
2.0
1.5
1.0
0.5
rarab
rarab
rarab
rarab
si:dkey−242n9.2
32880000
32900000
32920000
32940000
chr 3

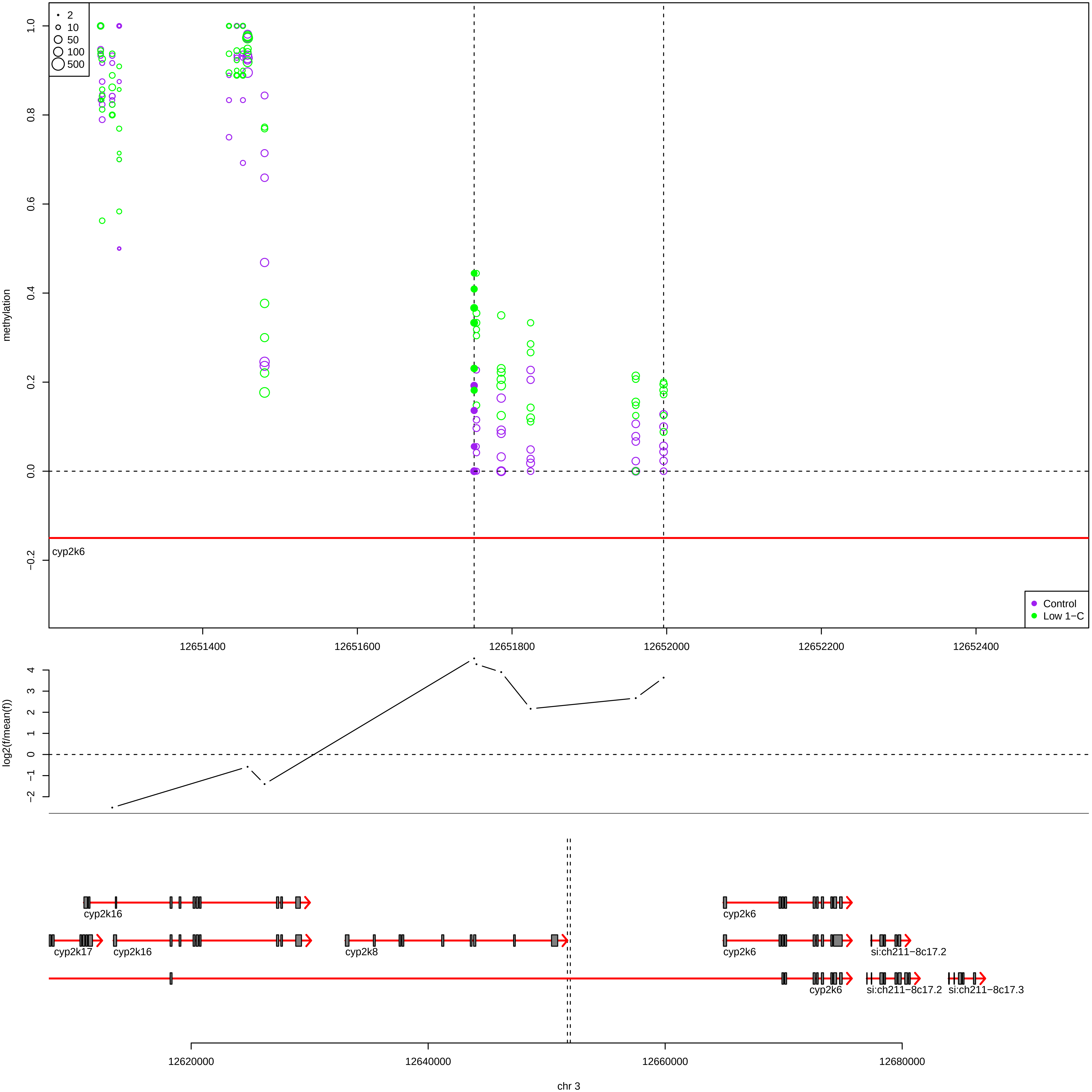

methylation
2
10
50
100
500
1.0
0.8
0.6
0.4
0.2
0.0
−0.2
cyp2k6
Control
Low 1−C
12651400
12651600
12651800
12652000
12652200
12652400
log2(f/mean(f))
4
3
2
1
0
−1
−2
cyp2k16
cyp2k6
cyp2k17
cyp2k16
cyp2k8
cyp2k6
si:ch211−8c17.2
cyp2k6
si:ch211−8c17.2
si:ch211−8c17.3
12620000
12640000
12660000
12680000
chr 3

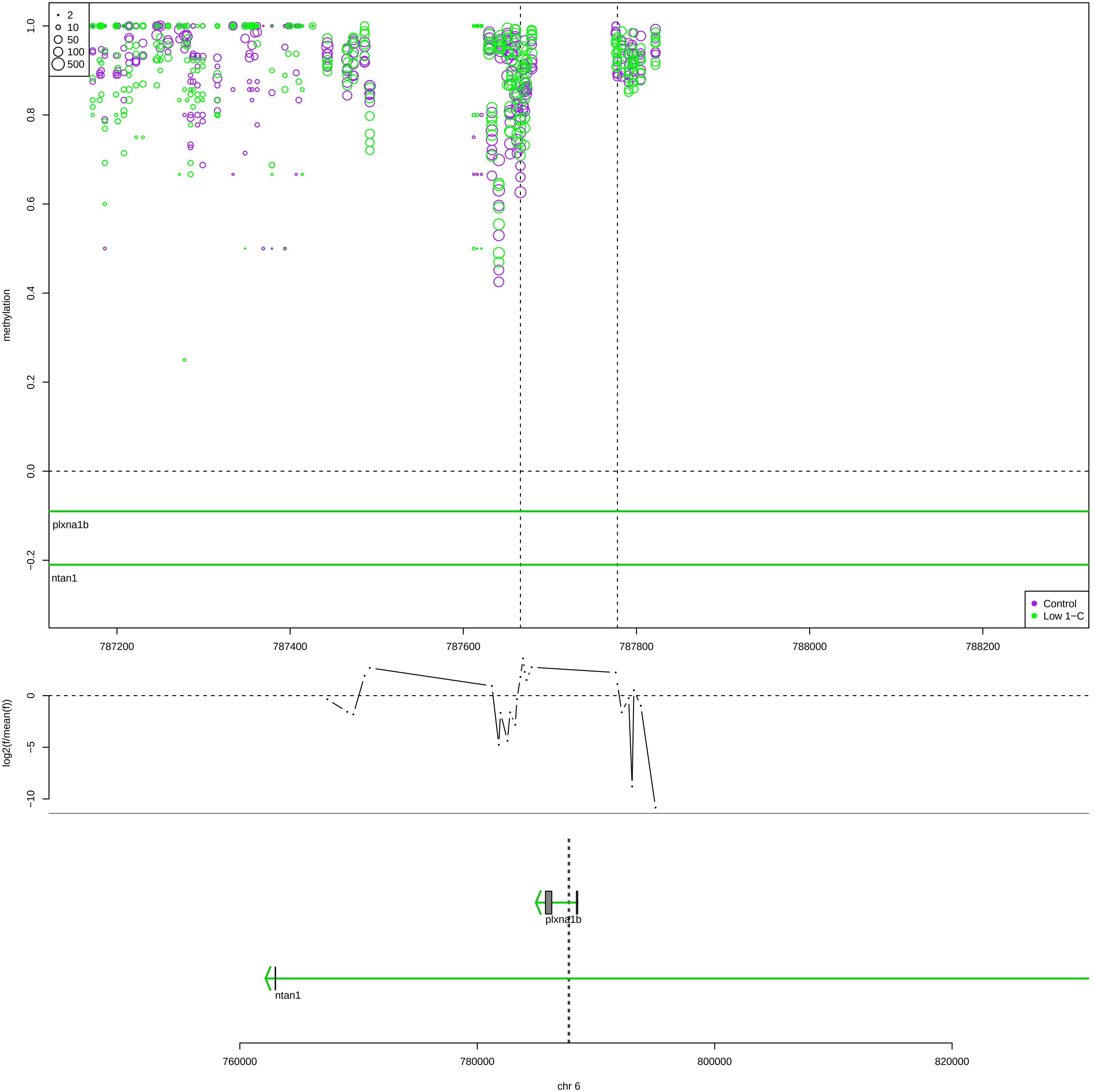

2
10
50
100
500
1.0
0.8
0.6
0.4
0.2
0.0
−0.2
methylation
plxna1b
ntan1
Control
Low 1−C
787200
787400
787600
787800
788000
788200
0
-5
-10
log2(f/mean(f))
plxna1b
ntan1
760000
780000
800000
820000
chr 6

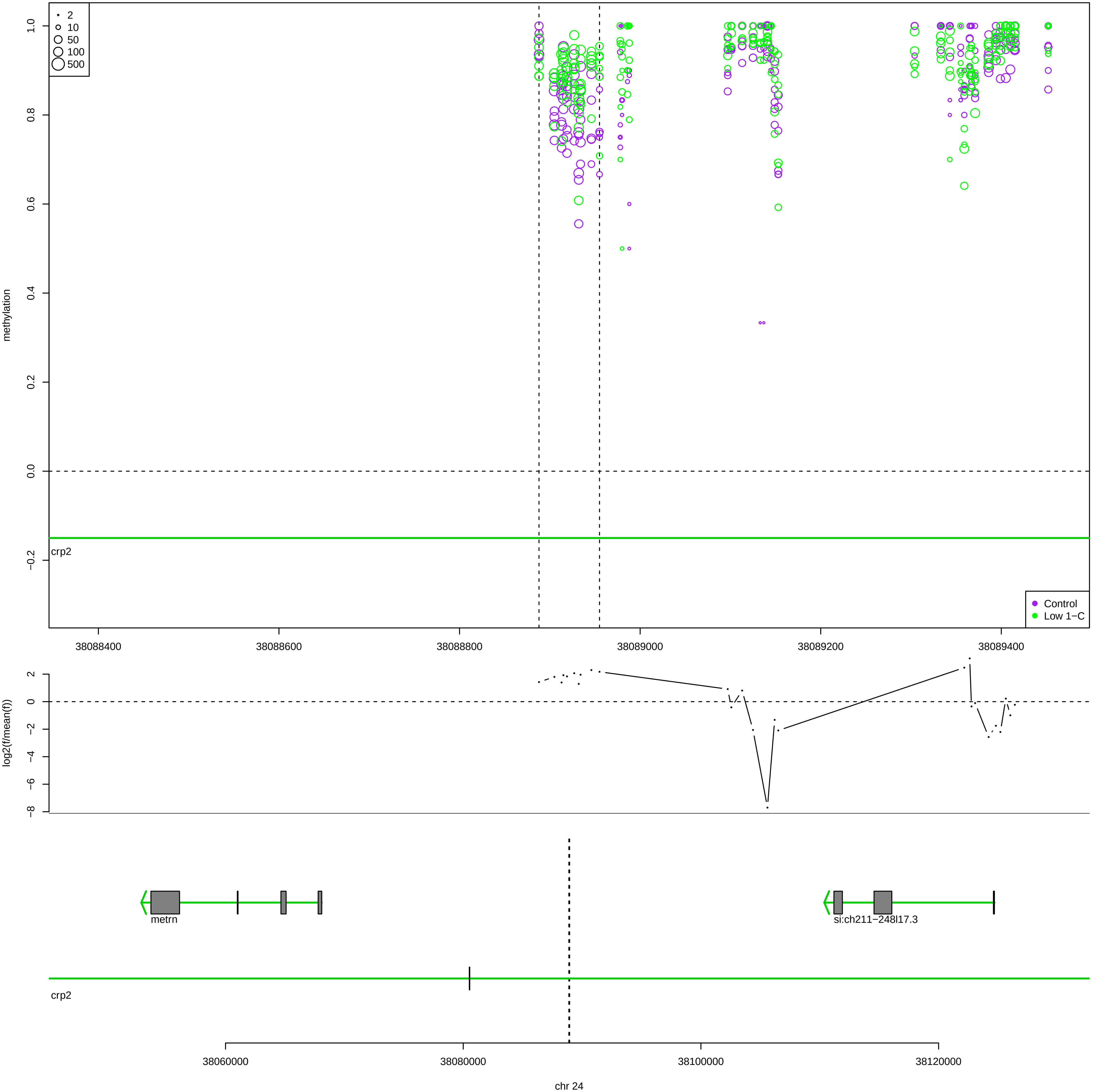

2
10
50
100
500
methylation
1.0
0.8
0.6
0.4
0.2
0.0
−0.2
crp2
Control
Low 1−C
38088400
38088600
38088800
38089000
38089200
38089400
log2(f/mean(f))
2
0
−2
−4
−6
−8
metrn
si:ch211−248l17.3
crp2
38060000
38080000
38100000
38120000
chr 24

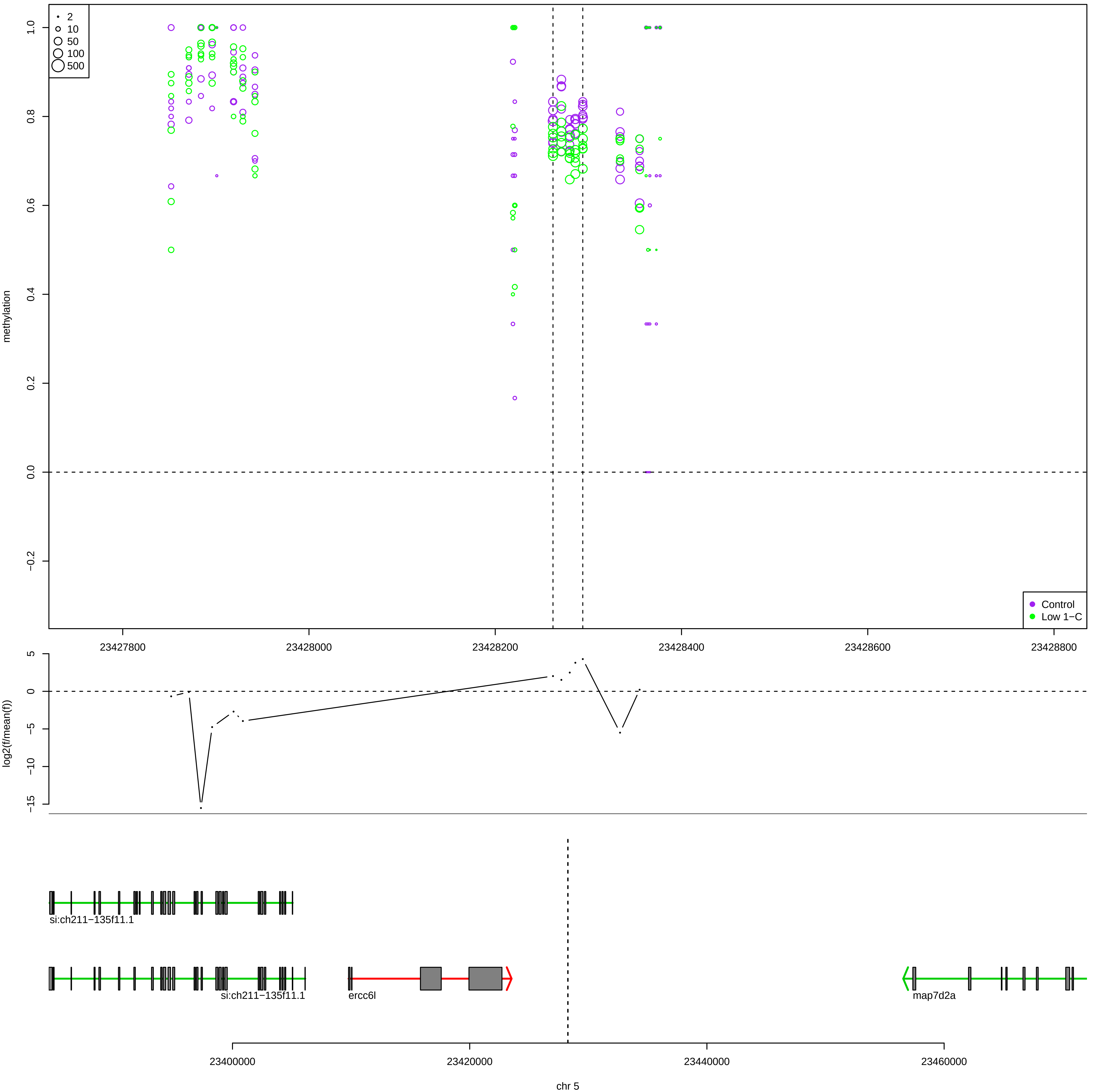

2
10
50
100
500
methylation
1.0
0.8
0.6
0.4
0.2
0.0
−0.2
Control
Low 1−C
23427800
23428000
23428200
23428400
23428600
23428800
log2(f/mean(f))
5
0
−5
−10
−15
si:ch211−135f11.1
si:ch211−135f11.1
ercc6l
map7d2a
23400000
23420000
23440000
23460000
chr 5

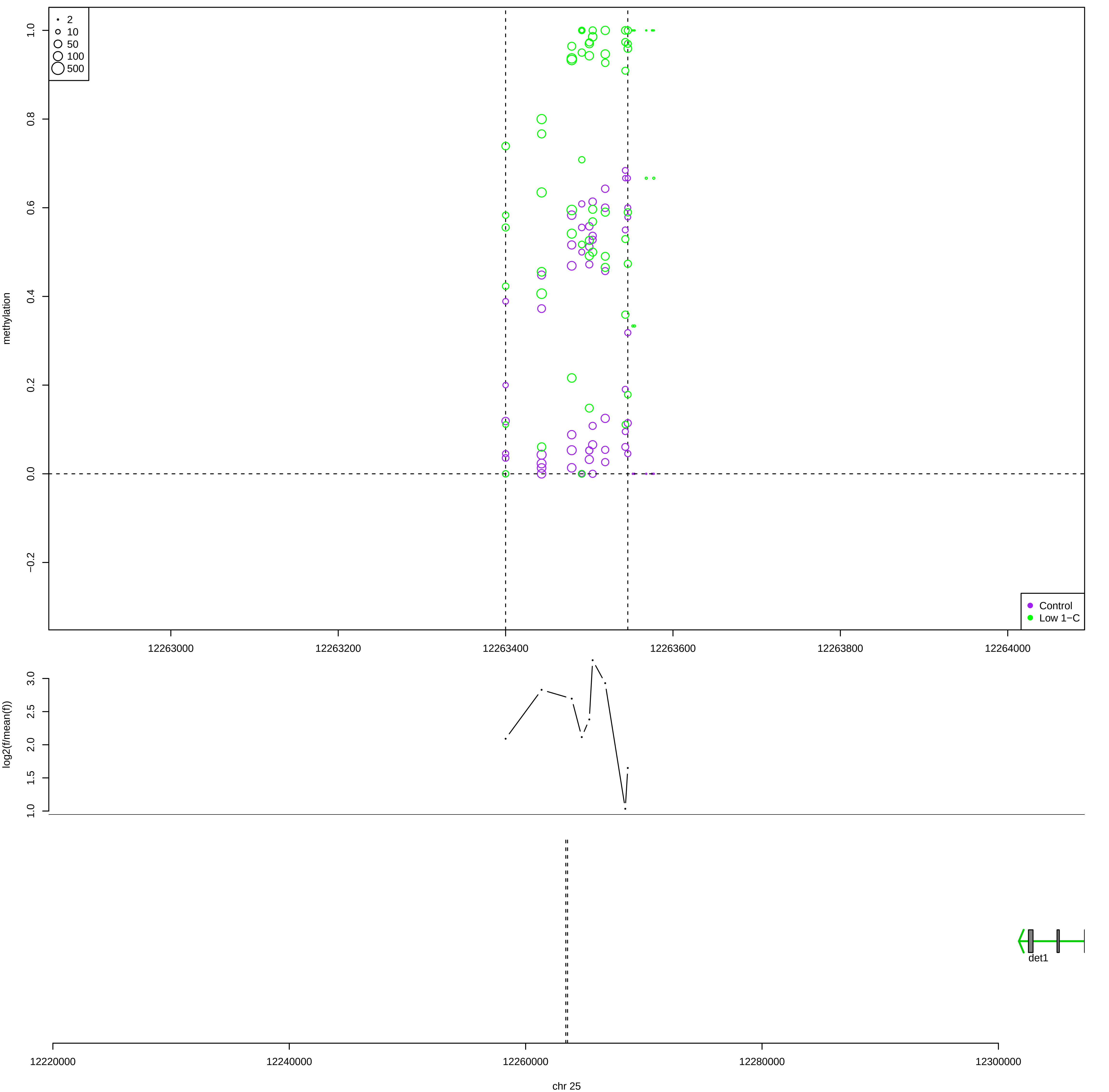
2
10
50
100
500
methylation
1.0
0.8
0.6
0.4
0.2
0.0
−0.2
Control
Low 1−C
12263000
12263200
12263400
12263600
12263800
12264000
log2(f/mean(f))
3.0
2.5
2.0
1.5
1.0
det1
12220000
12240000
12260000
12280000
12300000
chr 25

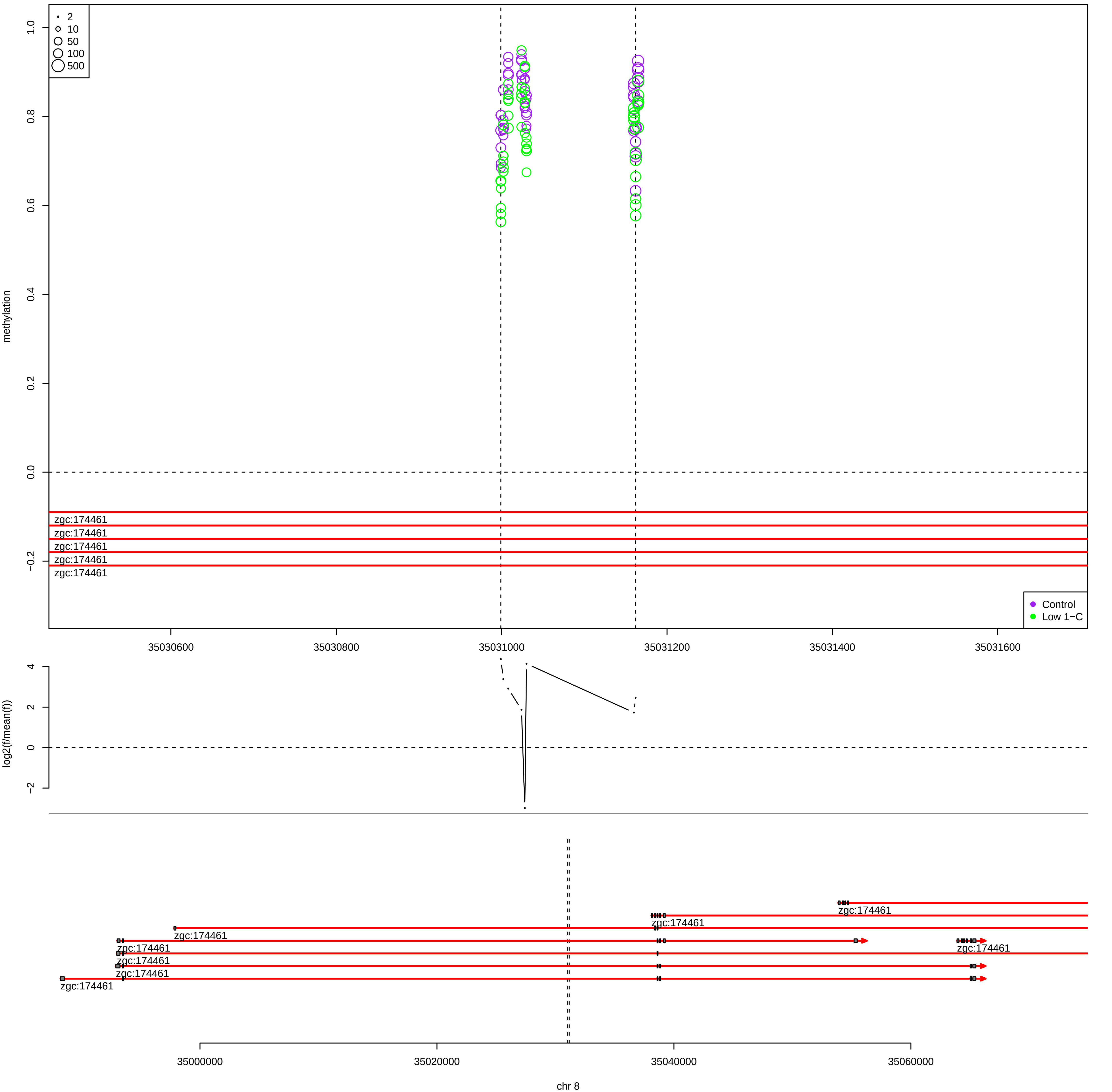
2
10
50
100
500
methylation
1.0
0.8
0.6
0.4
0.2
0.0
−0.2
zgc:174461
zgc:174461
zgc:174461
zgc:174461
zgc:174461
Control
Low 1−C
35030600
35030800
35031000
35031200
35031400
35031600
log2(f/mean(f))
4
2
0
−2
zgc:174461
zgc:174461
zgc:174461
zgc:174461
zgc:174461
zgc:174461
zgc:174461
zgc:174461
35000000
35020000
35040000
35060000
chr 8

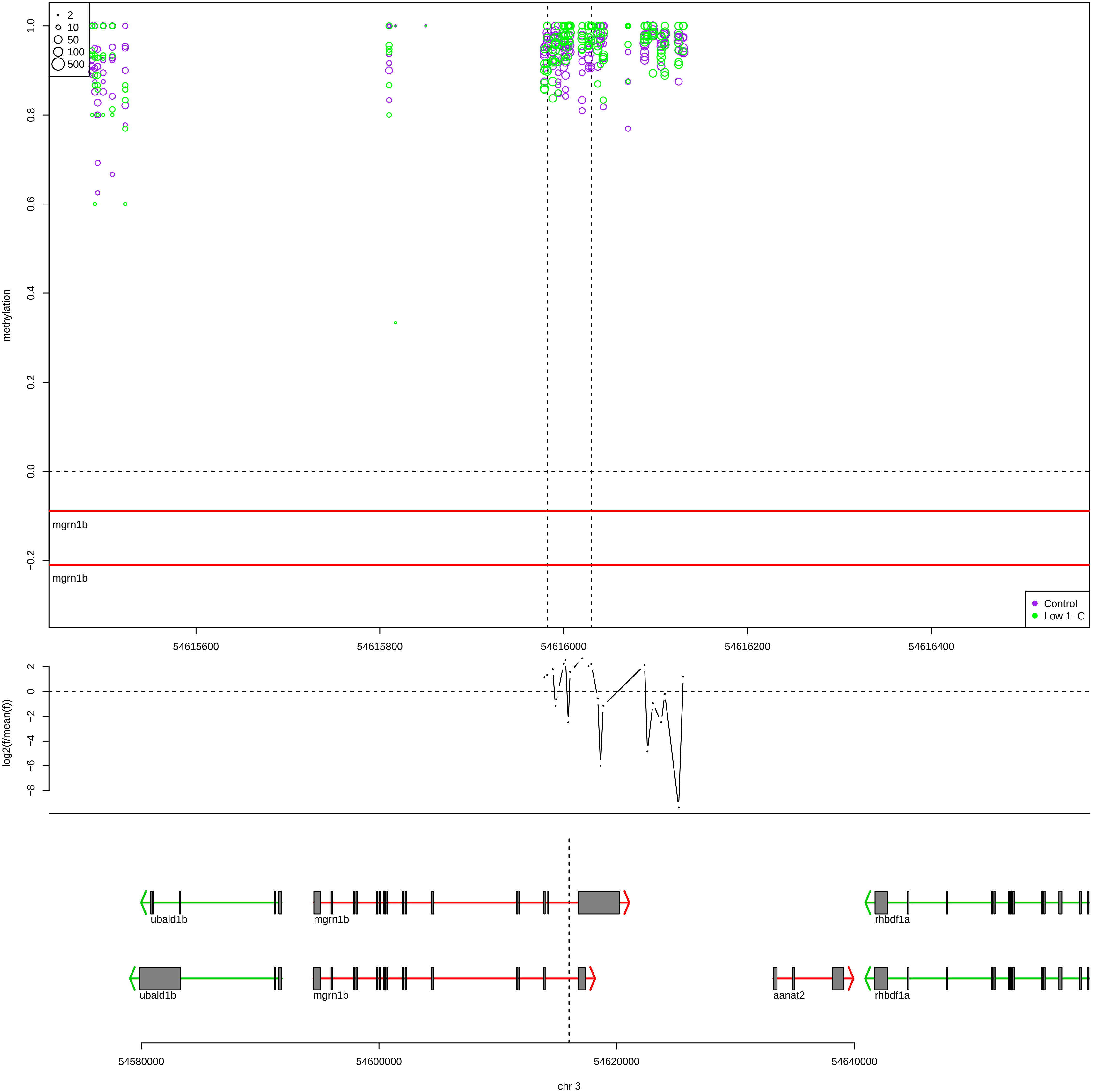
2
10
50
100
500
methylation
1.0
0.8
0.6
0.4
0.2
0.0
−0.2
mgrn1b
mgrn1b
Control
Low 1−C
54615600
54615800
54616000
54616200
54616400
log2(f/mean(f))
2
0
−2
−4
−6
−8
ubald1b
mgrn1b
rhbdf1a
ubald1b
mgrn1b
aanat2
rhbdf1a
54580000
54600000
54620000
54640000
chr 3

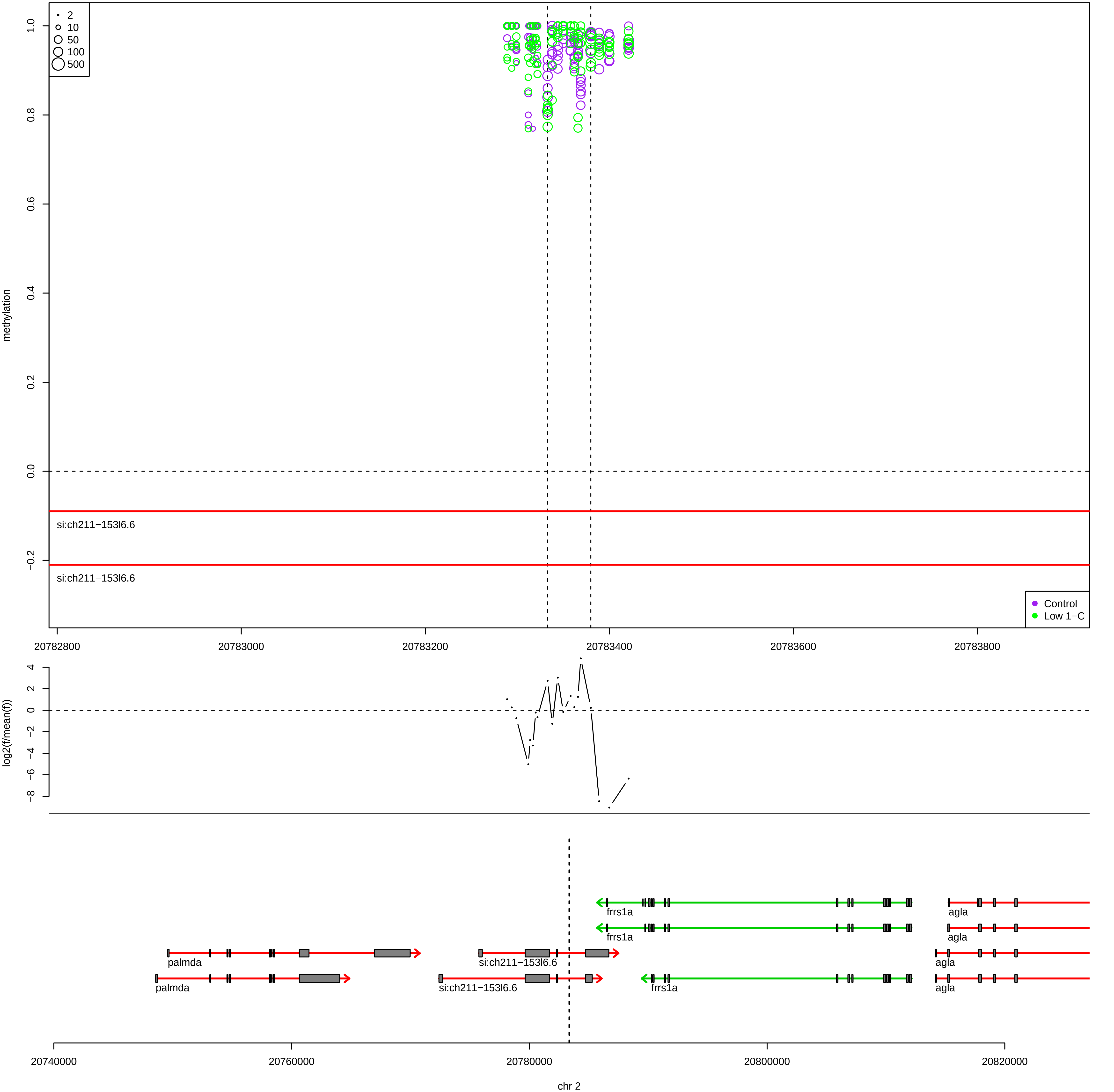

2
10
50
100
500
methylation
1.0
0.8
0.6
0.4
0.2
0.0
−0.2
si:ch211−153l6.6
si:ch211−153l6.6
Control
Low 1−C
20782800
20783000
20783200
20783400
20783600
20783800
log2(f/mean(f))
4
2
0
−2
−4
−6
−8
frrs1a
frrs1a
frrs1a
agla
agla
agla
agla
palmda
palmda
si:ch211−153l6.6
si:ch211−153l6.6
20740000
20760000
20780000
20800000
20820000
chr 2

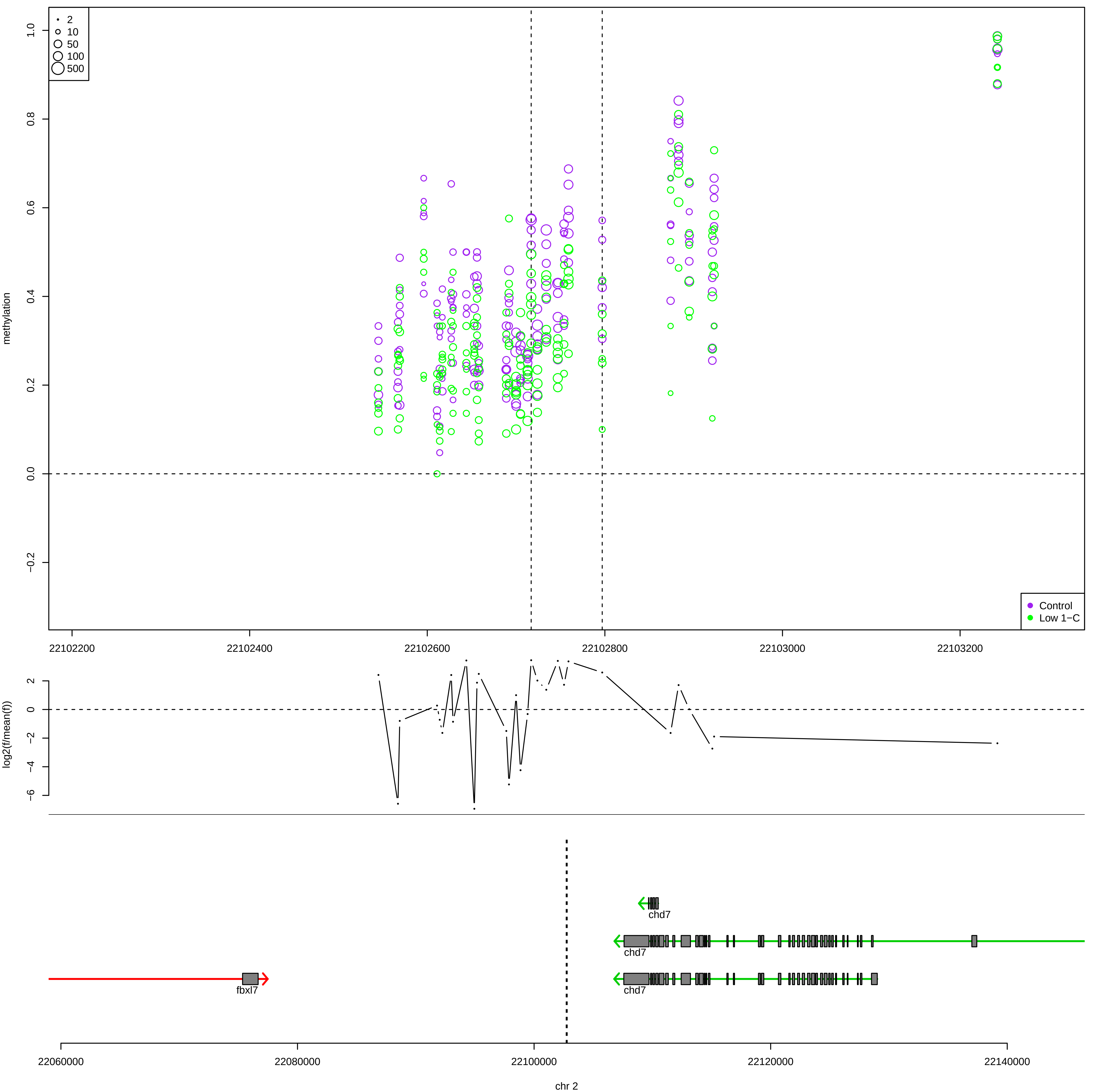

2
10
50
100
500
methylation
1.0
0.8
0.6
0.4
0.2
0.0
-0.2
Control
Low 1−C
22102200
22102400
22102600
22102800
22103000
22103200
log2(f/mean(f))
2
0
-2
-4
-6
chd7
chd7
chd7
fbxl7
22060000
22080000
22100000
22120000
22140000
chr 2

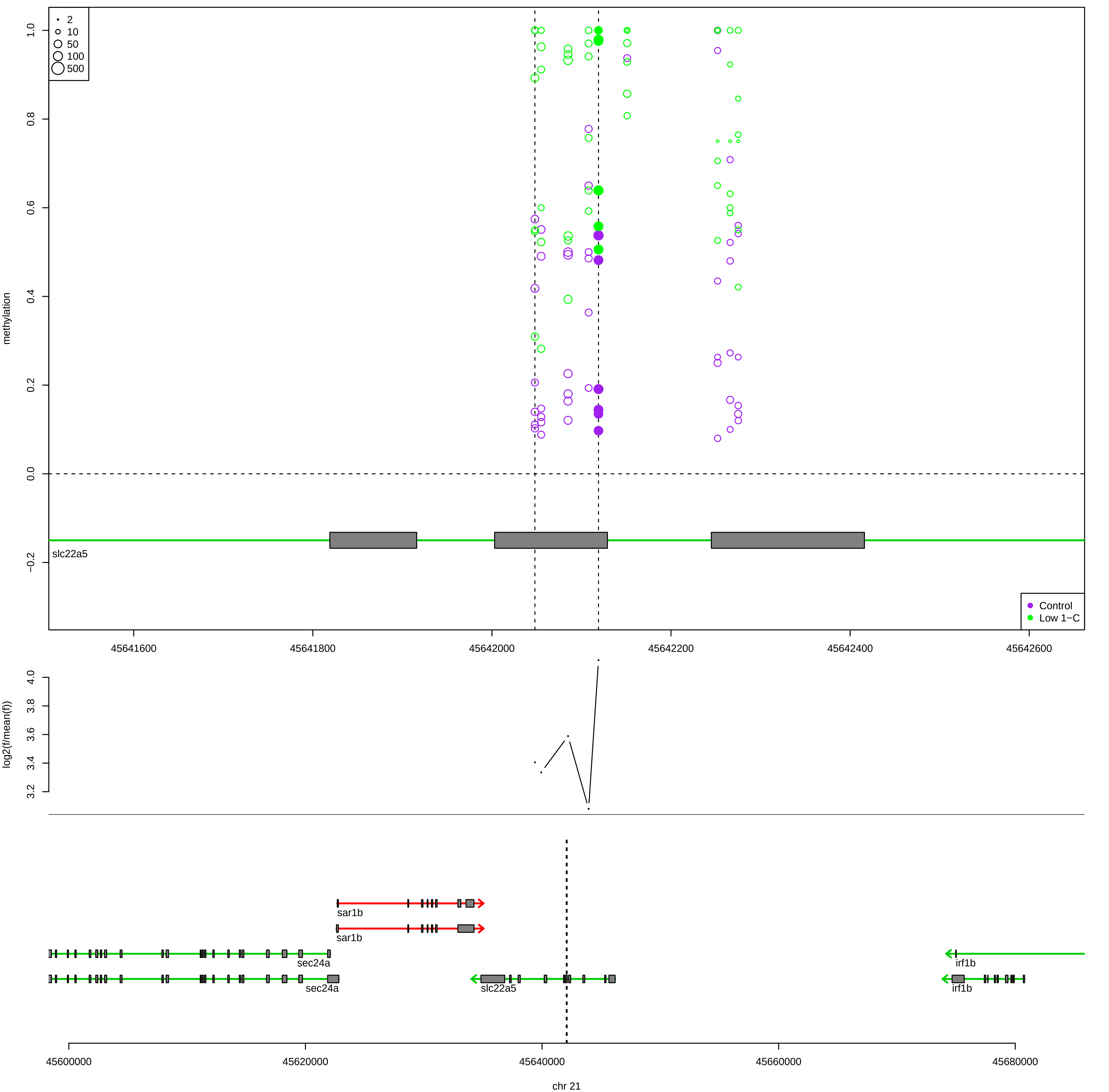

2
10
50
100
500
methylation
1.0
0.8
0.6
0.4
0.2
0.0
−0.2
slc22a5
Control
Low 1−C
45641600
45641800
45642000
45642200
45642400
45642600
log2(f/mean(f))
4.0
3.8
3.6
3.4
3.2
sar1b
sar1b
sec24a
sec24a
slc22a5
irf1b
irf1b
45600000
45620000
45640000
45660000
45680000
chr 21

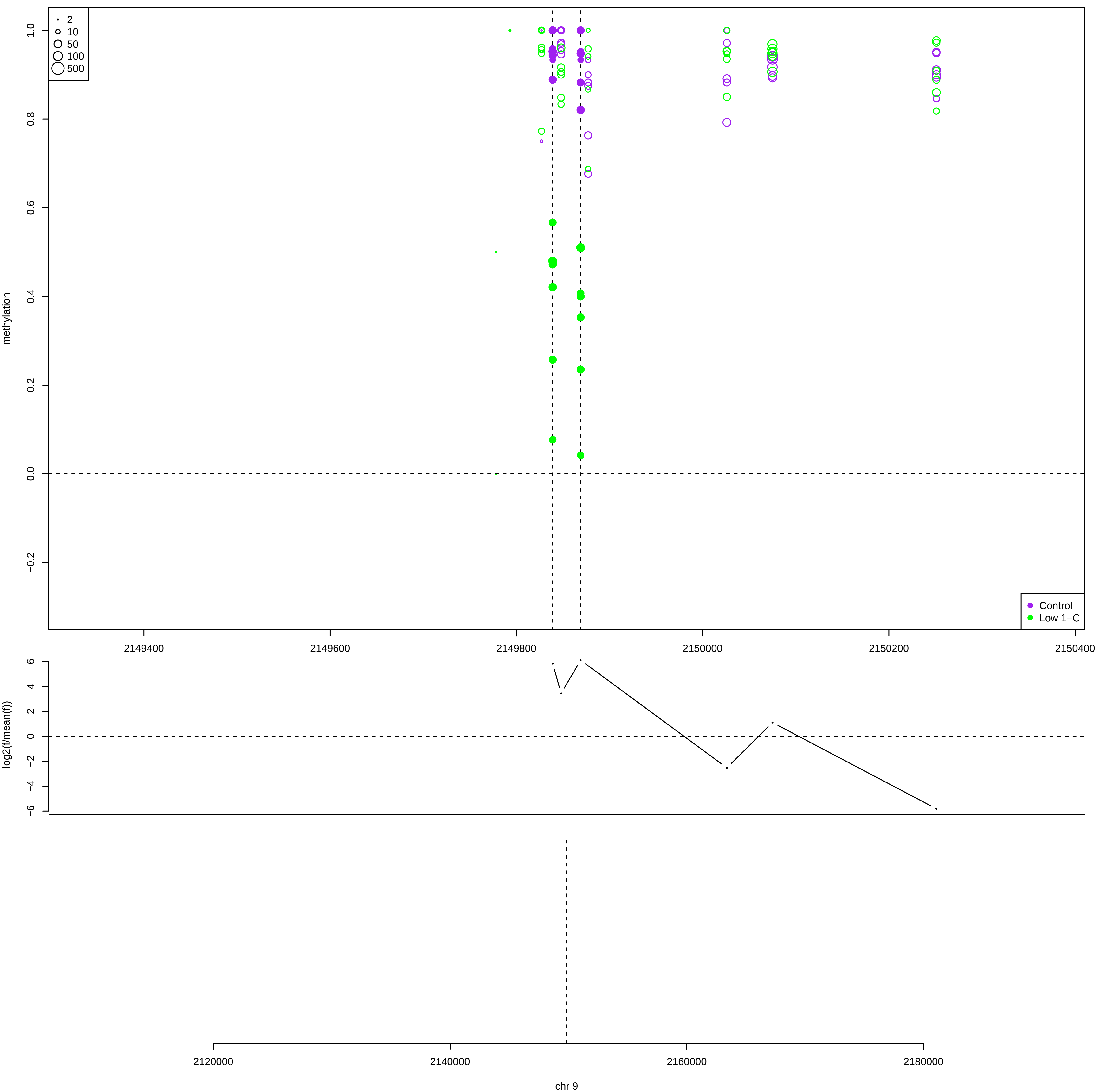
methylation
log2(f/mean(f))
2
10
50
100
500
Control
Low 1−C
2149400
2149600
2149800
2150000
2150200
2150400
2120000
2140000
2160000
2180000
chr 9

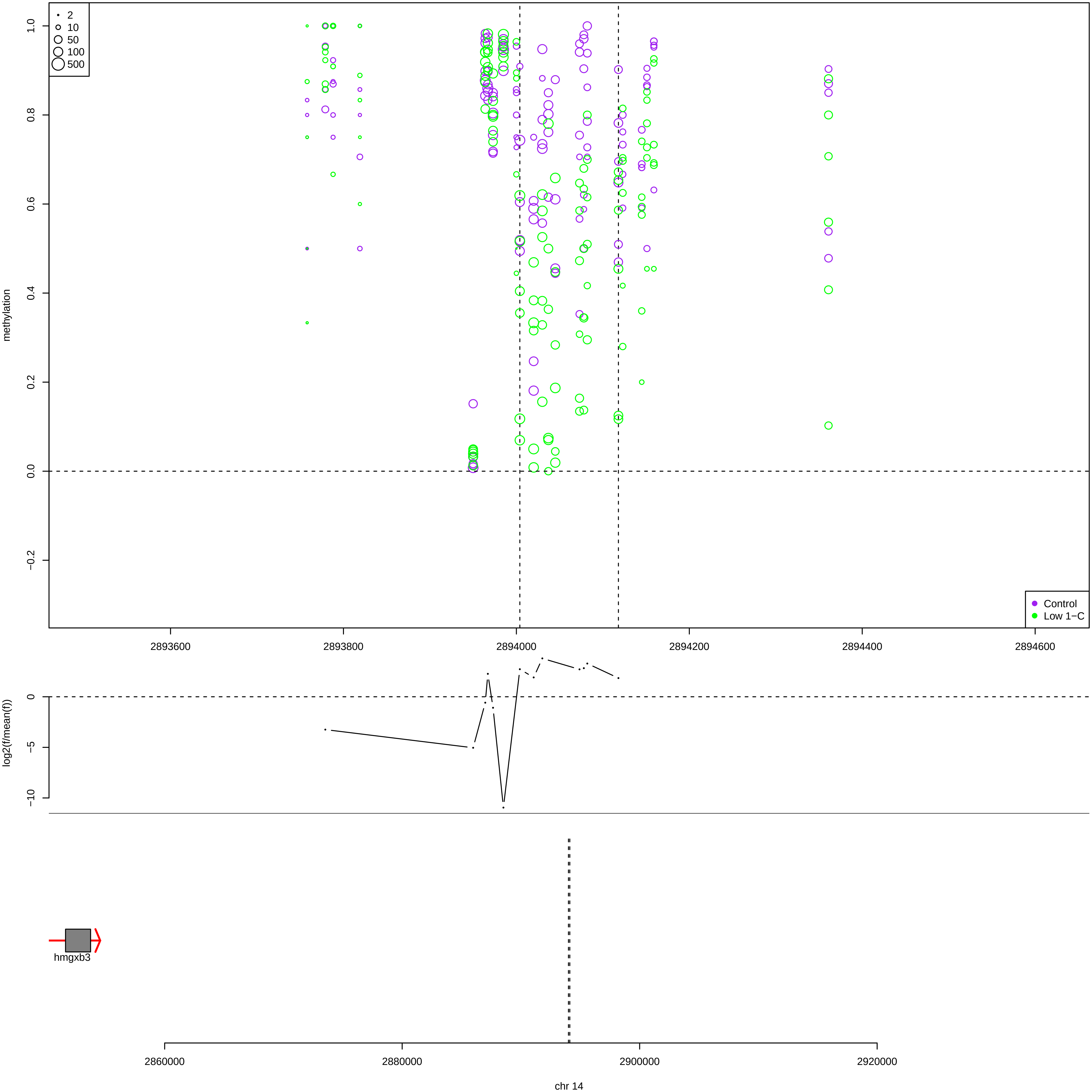
2
10
50
100
500
Control
Low 1−C
methylation
1.0
0.8
0.6
0.4
0.2
0.0
−0.2
2893600
2893800
2894000
2894200
2894400
2894600
log2(f/mean(f))
0
−5
−10
hmgxb3
2860000
2880000
2900000
2920000
chr 14

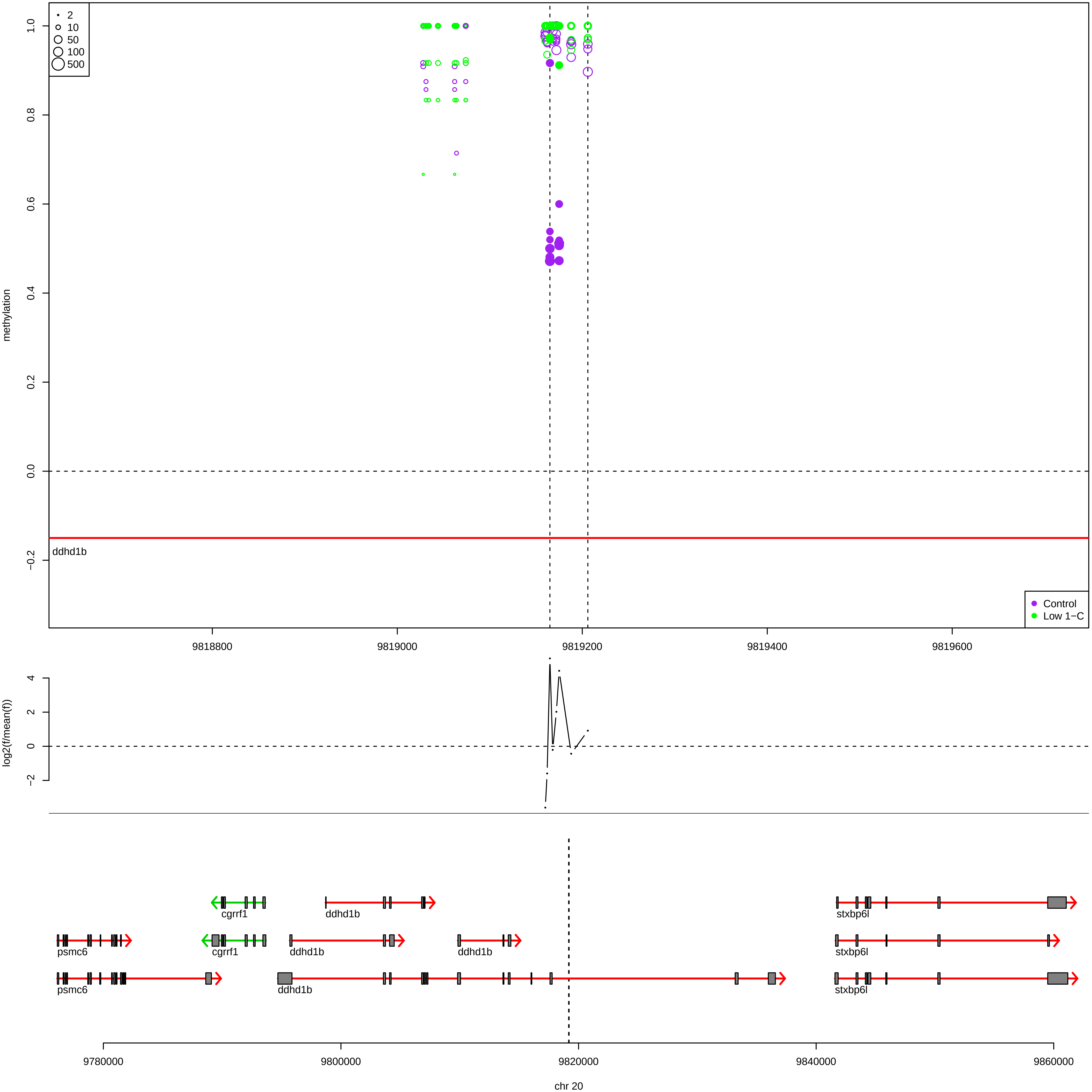
methylation
1.0
0.8
0.6
0.4
0.2
0.0
−0.2
2
10
50
100
500
ddhd1b
Control
Low 1−C
9818800
9819000
9819200
9819400
9819600
log2(f/mean(f))
4
2
0
−2
cgrrf1
ddhd1b
stxbp6l
psmc6
cgrrf1
ddhd1b
ddhd1b
stxbp6l
psmc6
ddhd1b
stxbp6l
9780000
9800000
9820000
9840000
9860000
chr 20

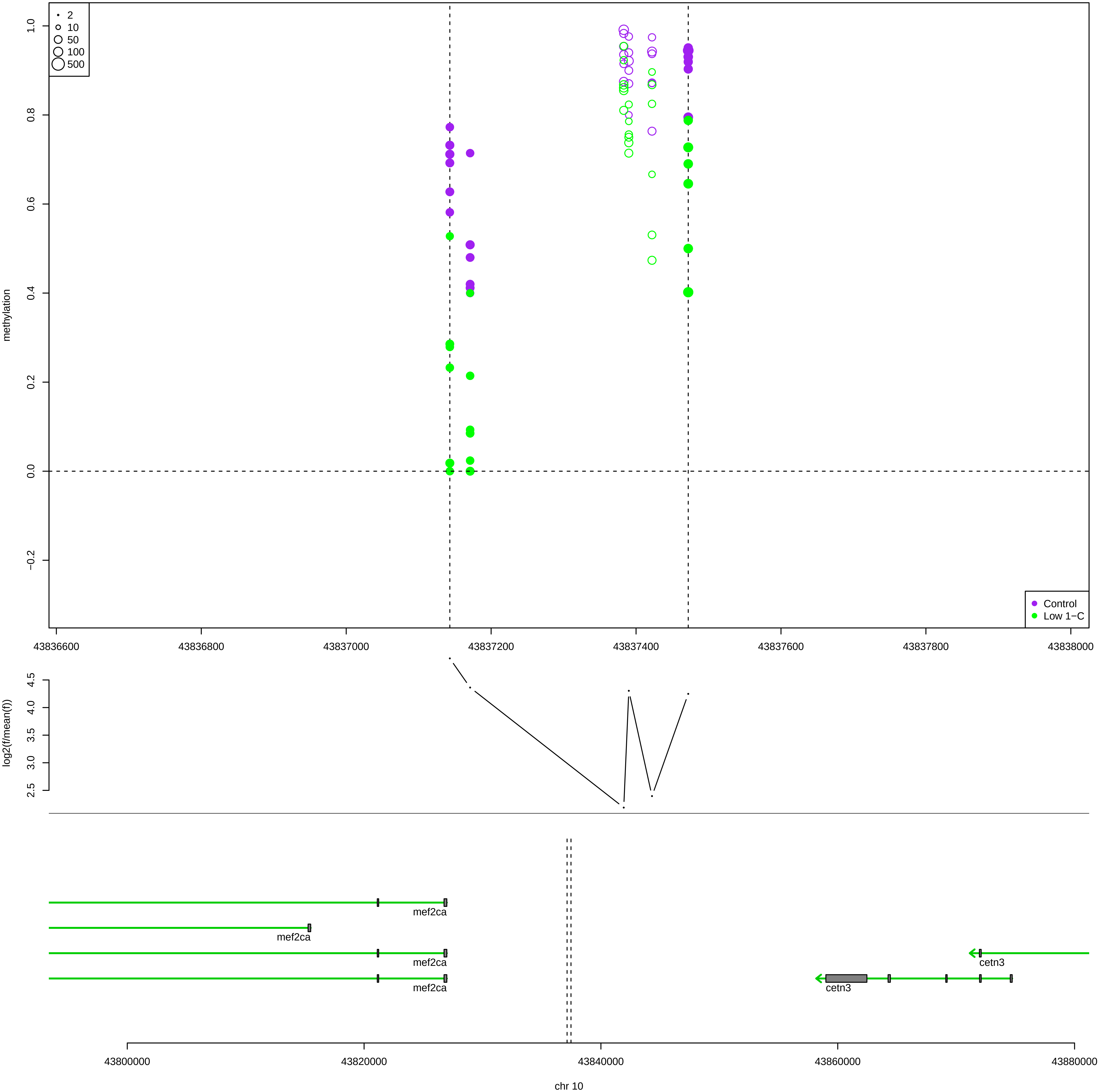

2
10
50
100
500
methylation
1.0
0.8
0.6
0.4
0.2
0.0
−0.2
Control
Low 1−C
43836600
43836800
43837000
43837200
43837400
43837600
43837800
43838000
log2(f/mean(f))
4.5
4.0
3.5
3.0
2.5
mef2ca
mef2ca
mef2ca
mef2ca
cetn3
cetn3
43800000
43820000
43840000
43860000
43880000
chr 10

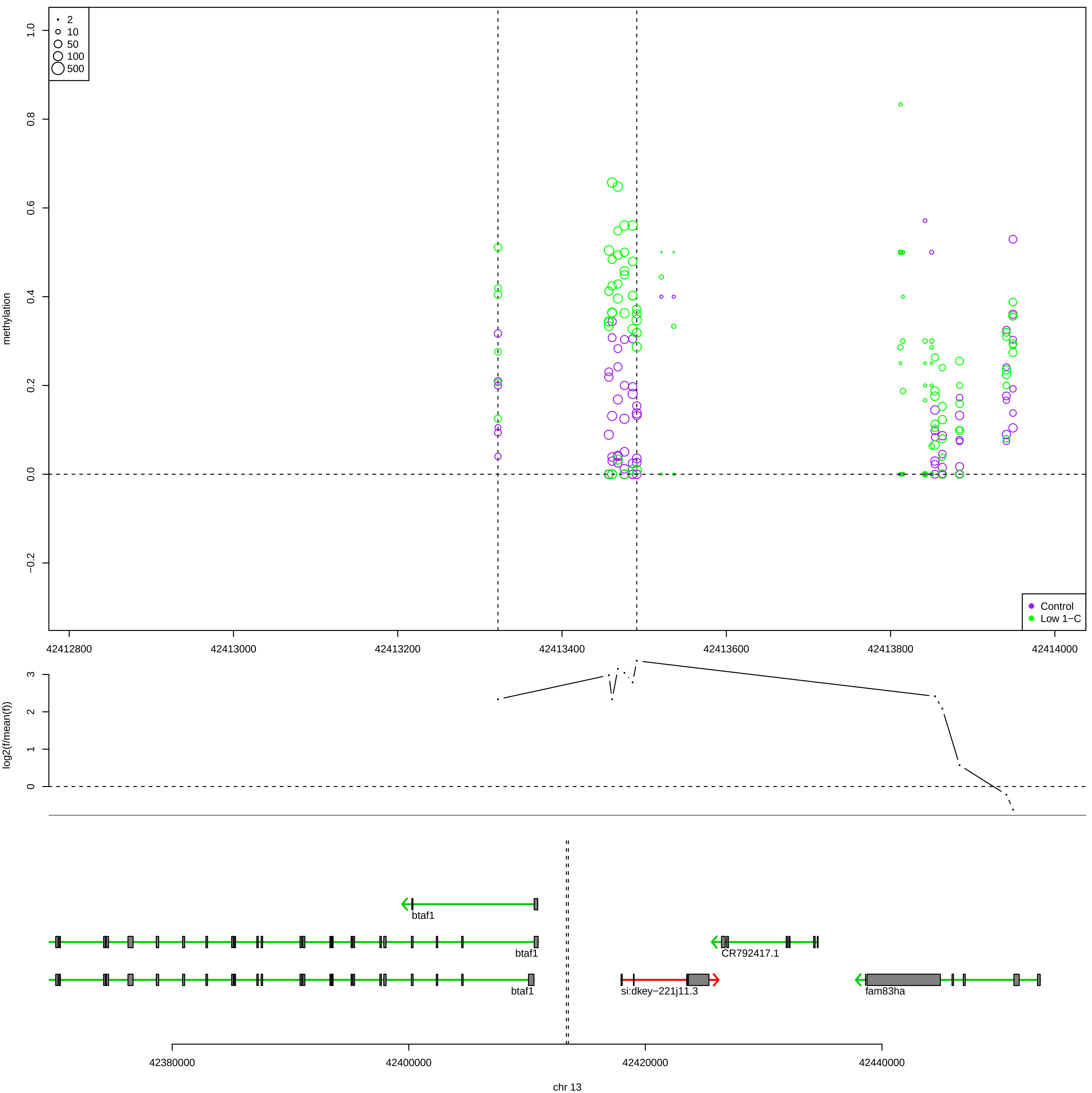

2
10
50
100
500
methylation
1.0
0.8
0.6
0.4
0.2
0.0
−0.2
Control
Low 1−C
42412800
42413000
42413200
42413400
42413600
42413800
42414000
log2(f/mean(f))
3
2
1
0
btaf1
btaf1
btaf1
CR792417.1
si:dkey−221j11.3
fam83ha
42380000
42400000
42420000
42440000
chr 13

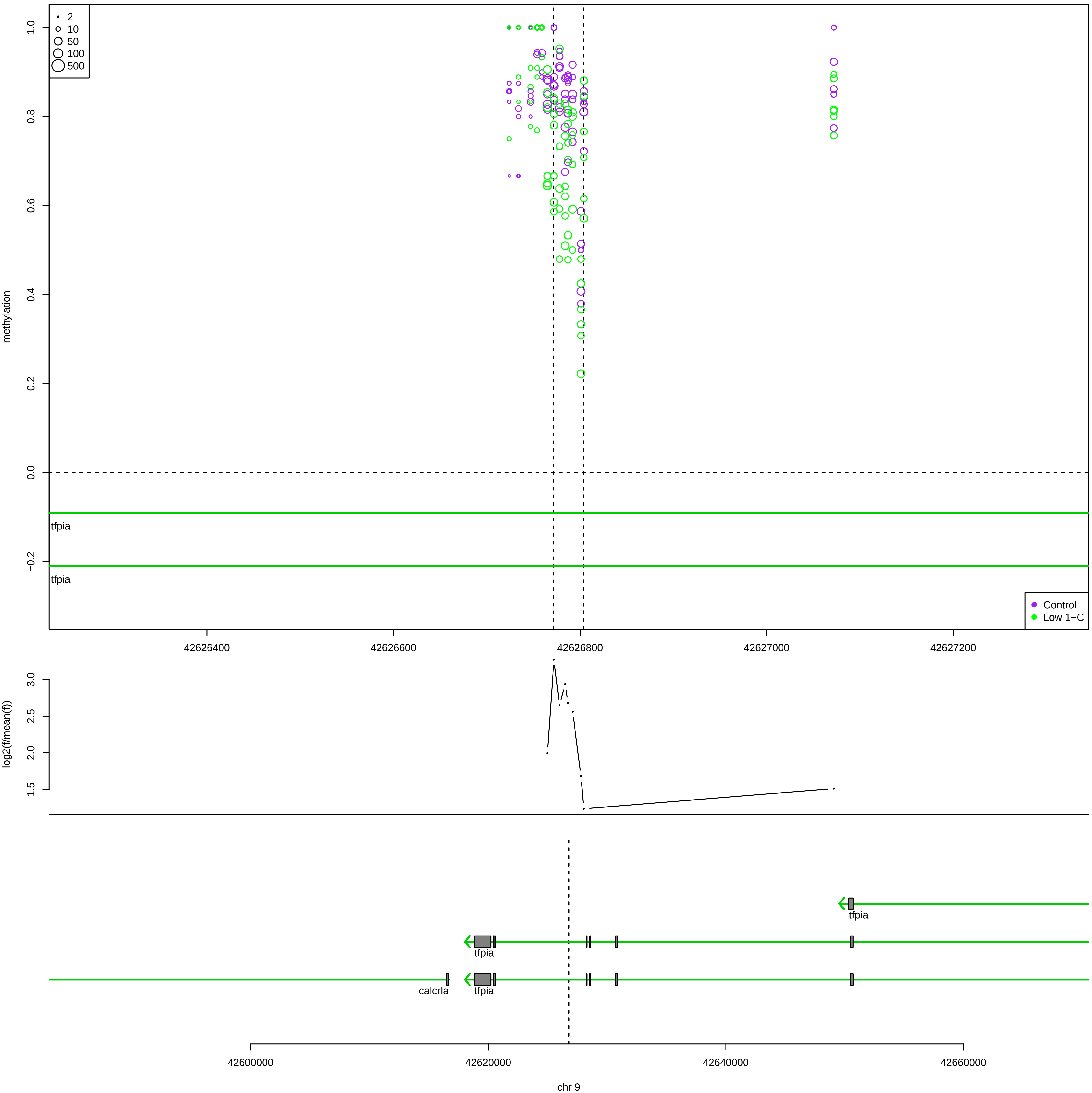

methylation
1.0
0.8
0.6
0.4
0.2
0.0
−0.2
2
10
50
100
500
tfpia
tfpia
Control
Low 1−C
42626400
42626600
42626800
42627000
42627200
log2(f/mean(f))
3.0
2.5
2.0
1.5
tfpia
tfpia
calcrla
tfpia
42600000
42620000
42640000
42660000
chr 9

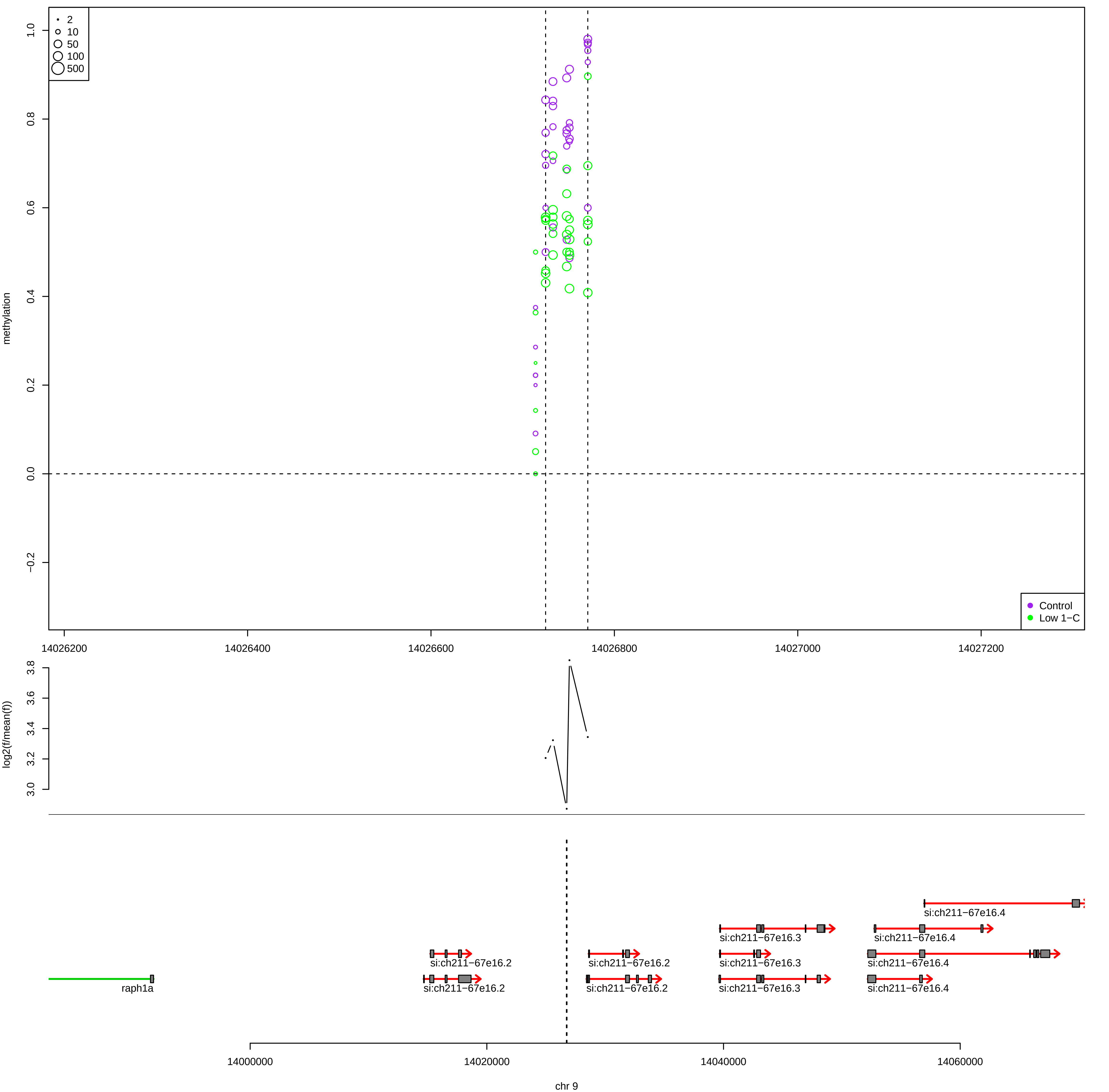

2
10
50
100
500
methylation
1.0
0.8
0.6
0.4
0.2
0.0
−0.2
Control
Low 1−C
14026200
14026400
14026600
14026800
14027000
14027200
log2(f/mean(f))
3.8
3.6
3.4
3.2
3.0
si:ch211−67e16.4
si:ch211−67e16.3
si:ch211−67e16.2
raph1a
14000000
14020000
14040000
14060000
chr 9

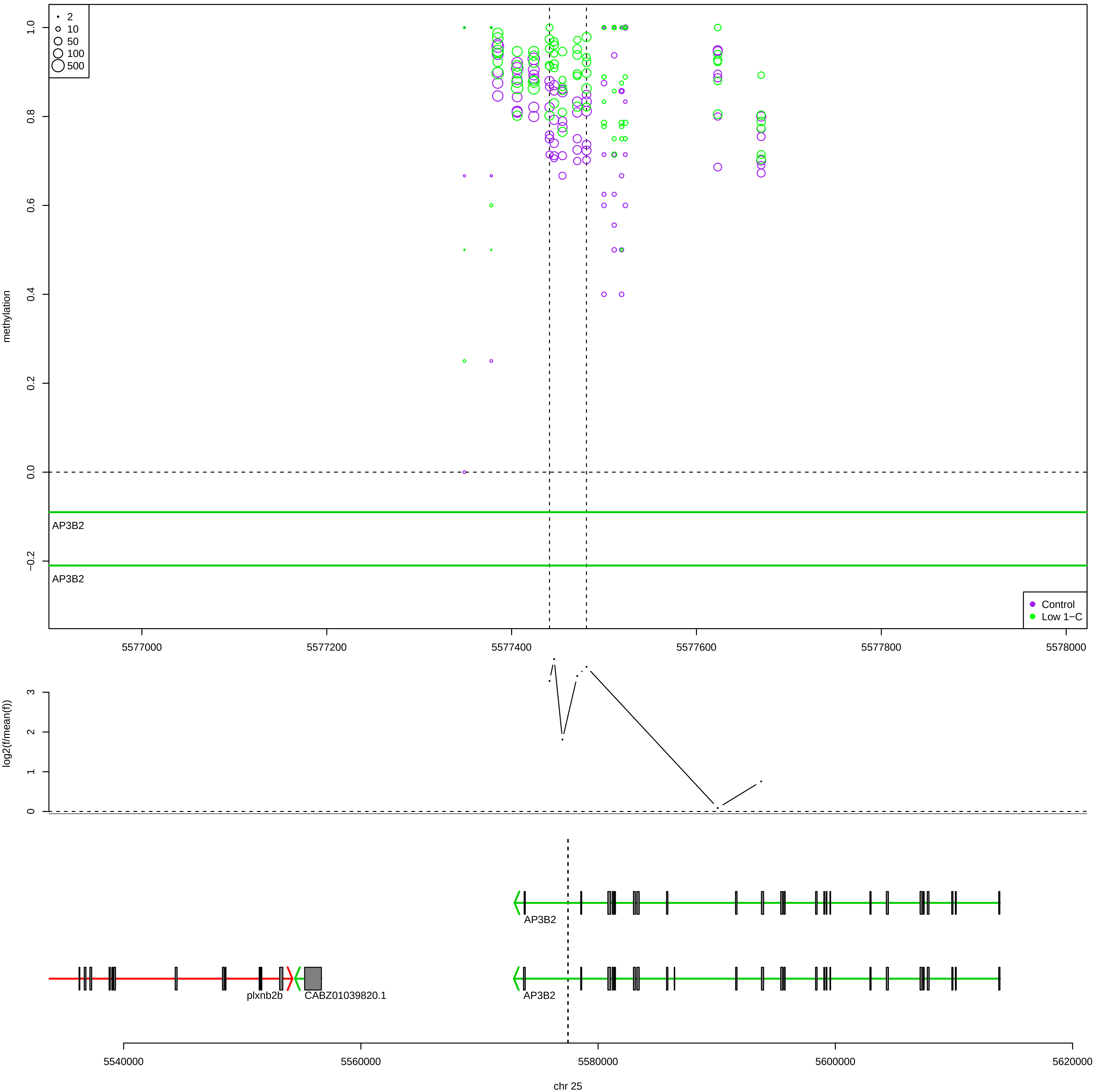

2
10
50
100
500
methylation
1.0
0.8
0.6
0.4
0.2
0.0
−0.2
AP3B2
AP3B2
Control
Low 1−C
5577000
5577200
5577400
5577600
5577800
5578000
log2(f/mean(f))
3
2
1
0
AP3B2
plxnb2b
CABZ01039820.1
AP3B2
5540000
5560000
5580000
5600000
5620000
chr 25

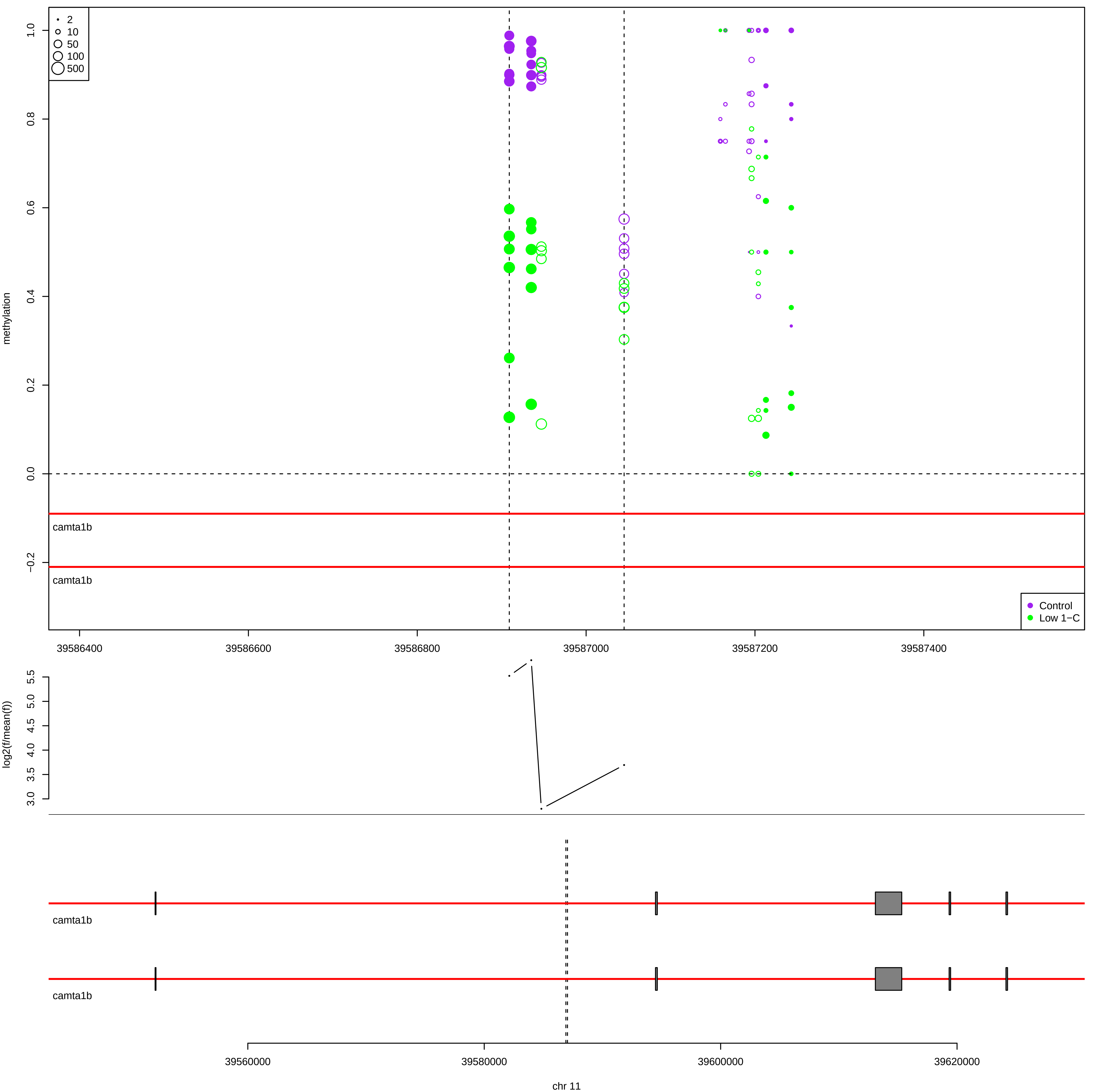

2
10
50
100
500
methylation
1.0
0.8
0.6
0.4
0.2
0.0
−0.2
camta1b
camta1b
Control
Low 1−C
39586400
39586600
39586800
39587000
39587200
39587400
log2(f/mean(f))
5.5
5.0
4.5
4.0
3.5
3.0
camta1b
camta1b
39560000
39580000
39600000
39620000
chr 11

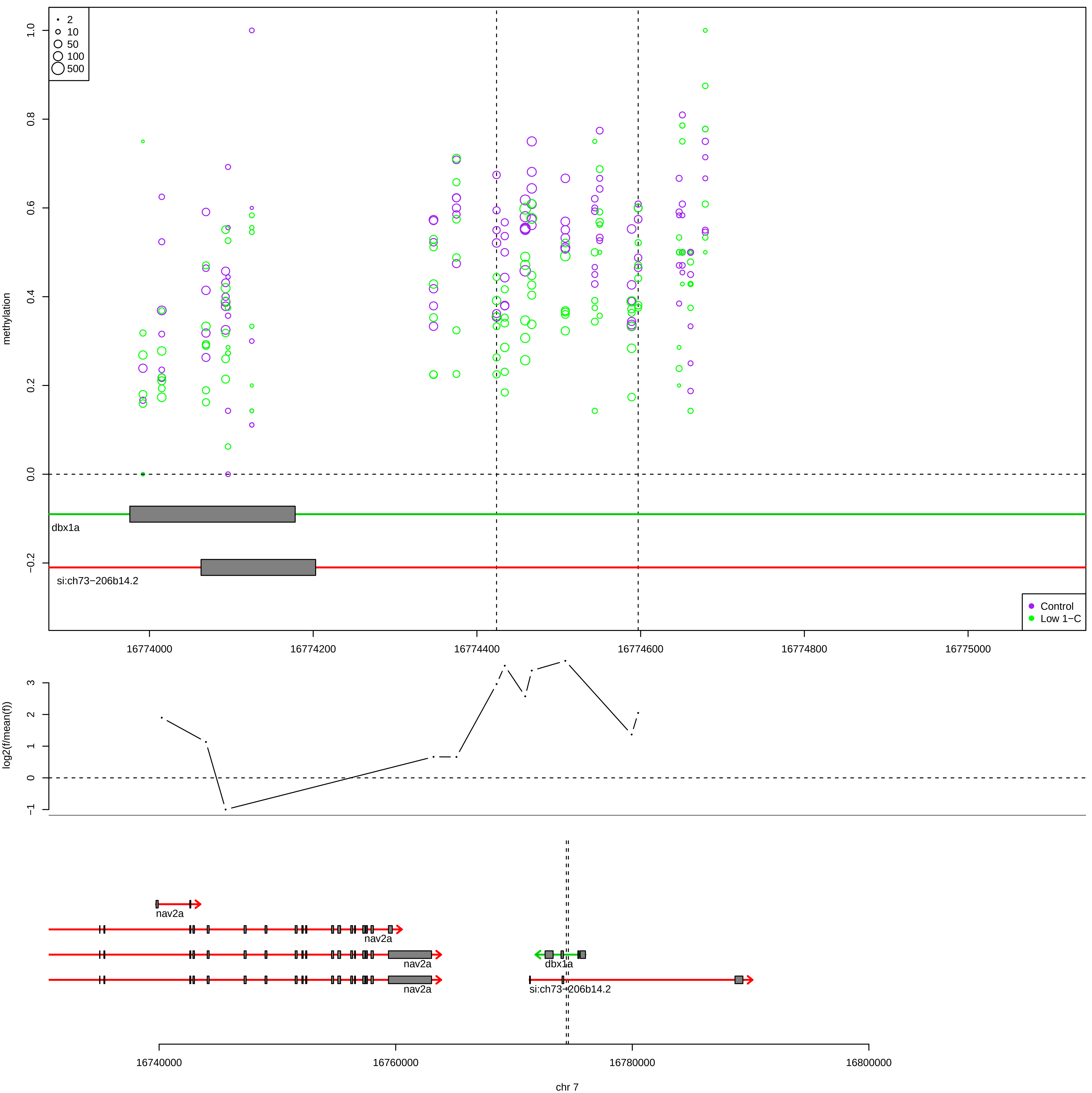

methylation
1.0
0.8
0.6
0.4
0.2
0.0
−0.2
2
10
50
100
500
dbx1a
si:ch73−206b14.2
Control
Low 1−C
16774000
16774200
16774400
16774600
16774800
16775000
log2(f/mean(f))
3
2
1
0
−1
nav2a
nav2a
nav2a
nav2a
dbx1a
si:ch73−206b14.2
16740000
16760000
16780000
16800000
chr 7

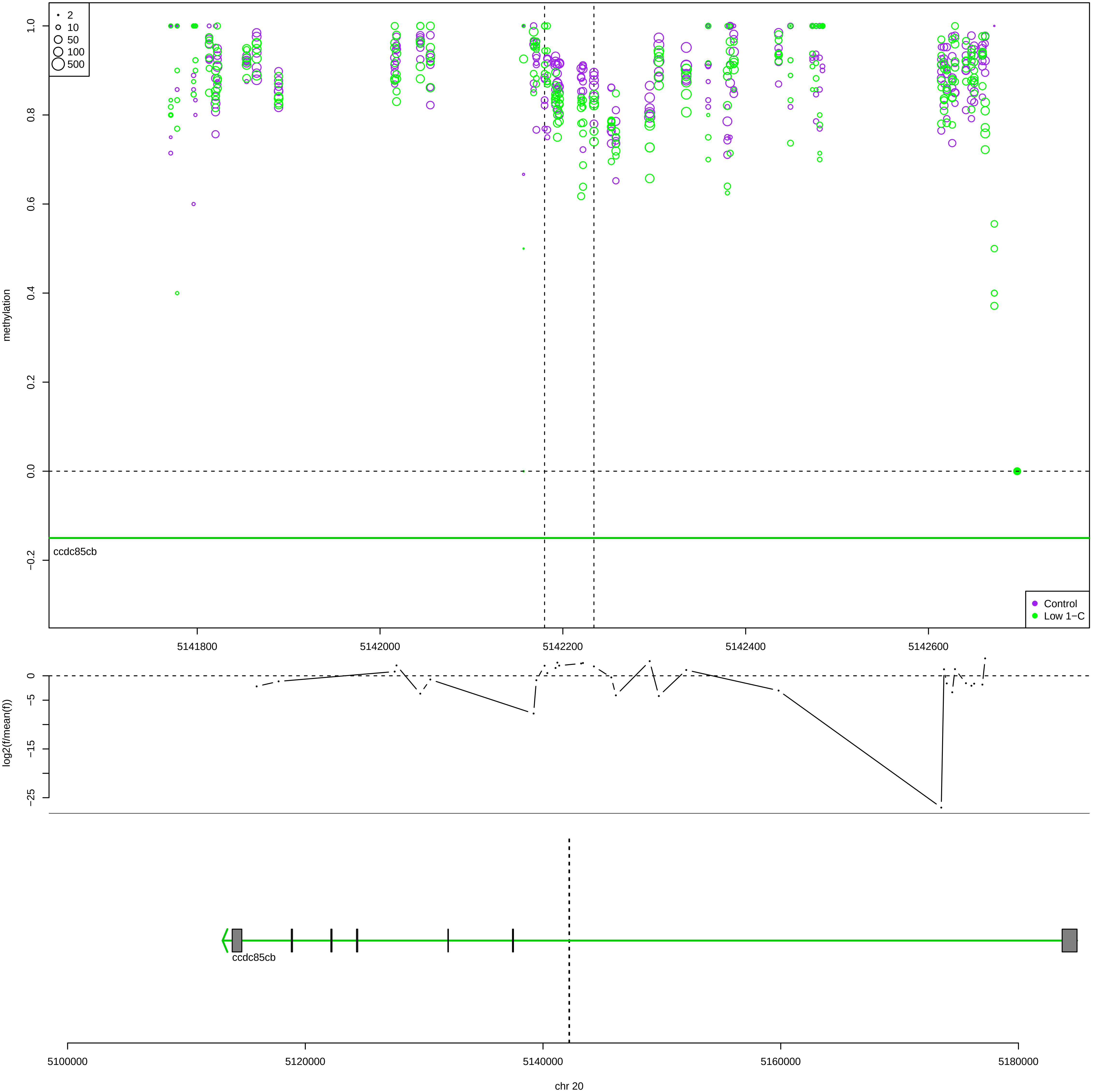

2
10
50
100
500
methylation
1.0
0.8
0.6
0.4
0.2
0.0
−0.2
ccdc85cb
Control
Low 1−C
5141800
5142000
5142200
5142400
5142600
log2(f/mean(f))
0
−5
−15
−25
ccdc85cb
5100000
5120000
5140000
5160000
5180000
chr 20

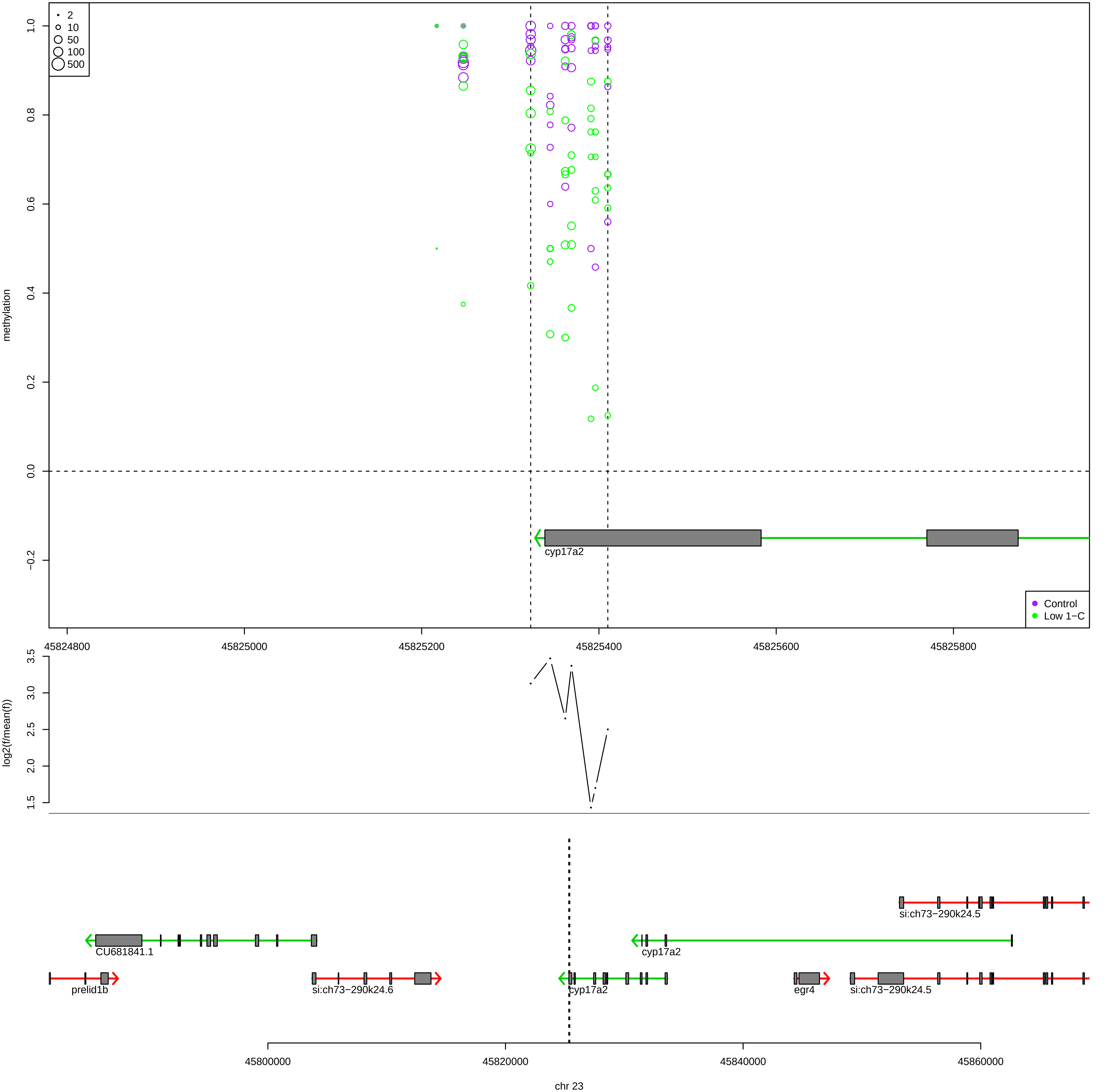

2
10
50
100
500
methylation
1.0
0.8
0.6
0.4
0.2
0.0
−0.2
cyp17a2
Control
Low 1−C
45824800
45825000
45825200
45825400
45825600
45825800
log2(f/mean(f))
3.5
3.0
2.5
2.0
1.5
si:ch73−290k24.5
CU681841.1
cyp17a2
prelid1b
si:ch73−290k24.6
cyp17a2
egr4
si:ch73−290k24.5
45800000
45820000
45840000
45860000
chr 23

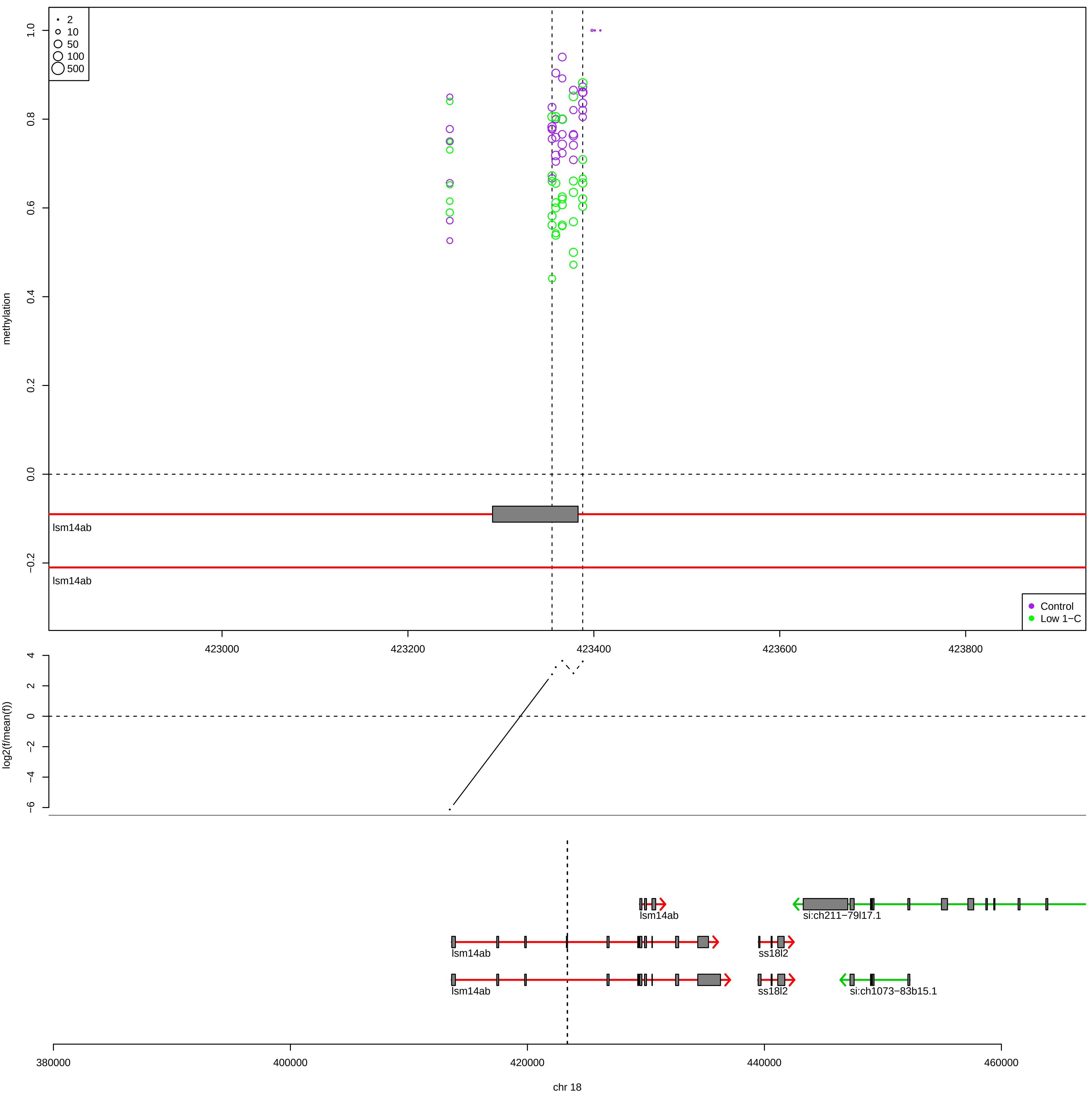

2
10
50
100
500
methylation
1.0
0.8
0.6
0.4
0.2
0.0
−0.2
lsm14ab
lsm14ab
Control
Low 1−C
423000
423200
423400
423600
423800
log2(f/mean(f))
4
2
0
−2
−4
−6
lsm14ab
si:ch211−79l17.1
lsm14ab
ss18l2
lsm14ab
ss18l2
si:ch1073−83b15.1
380000
400000
420000
440000
460000
chr 18

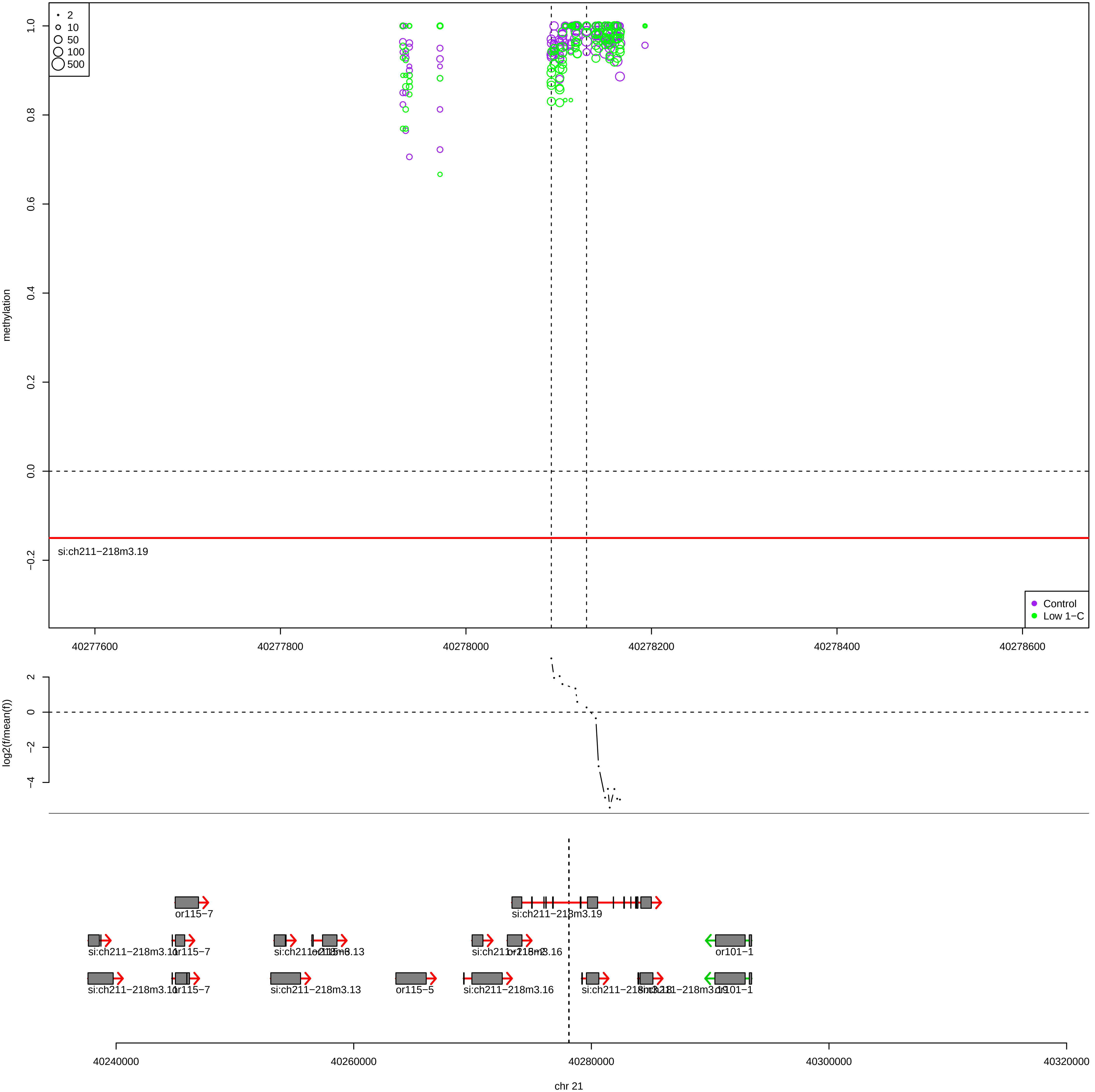

2
10
50
100
500
methylation
1.0
0.8
0.6
0.4
0.2
0.0
−0.2
si:ch211−218m3.19
Control
Low 1−C
40277600
40277800
40278000
40278200
40278400
40278600
log2(f/mean(f))
2
0
−2
−4
or115−7
si:ch211−218m3.19
si:ch211−218m3.10
or115−7
si:ch211−218m3.13
si:ch211−218m3.16
or101−1
si:ch211−218m3.10
or115−7
si:ch211−218m3.13
or115−5
si:ch211−218m3.16
si:ch211−218m3.18
si:ch211−218m3.19
or101−1
40240000
40260000
40280000
40300000
40320000
chr 21

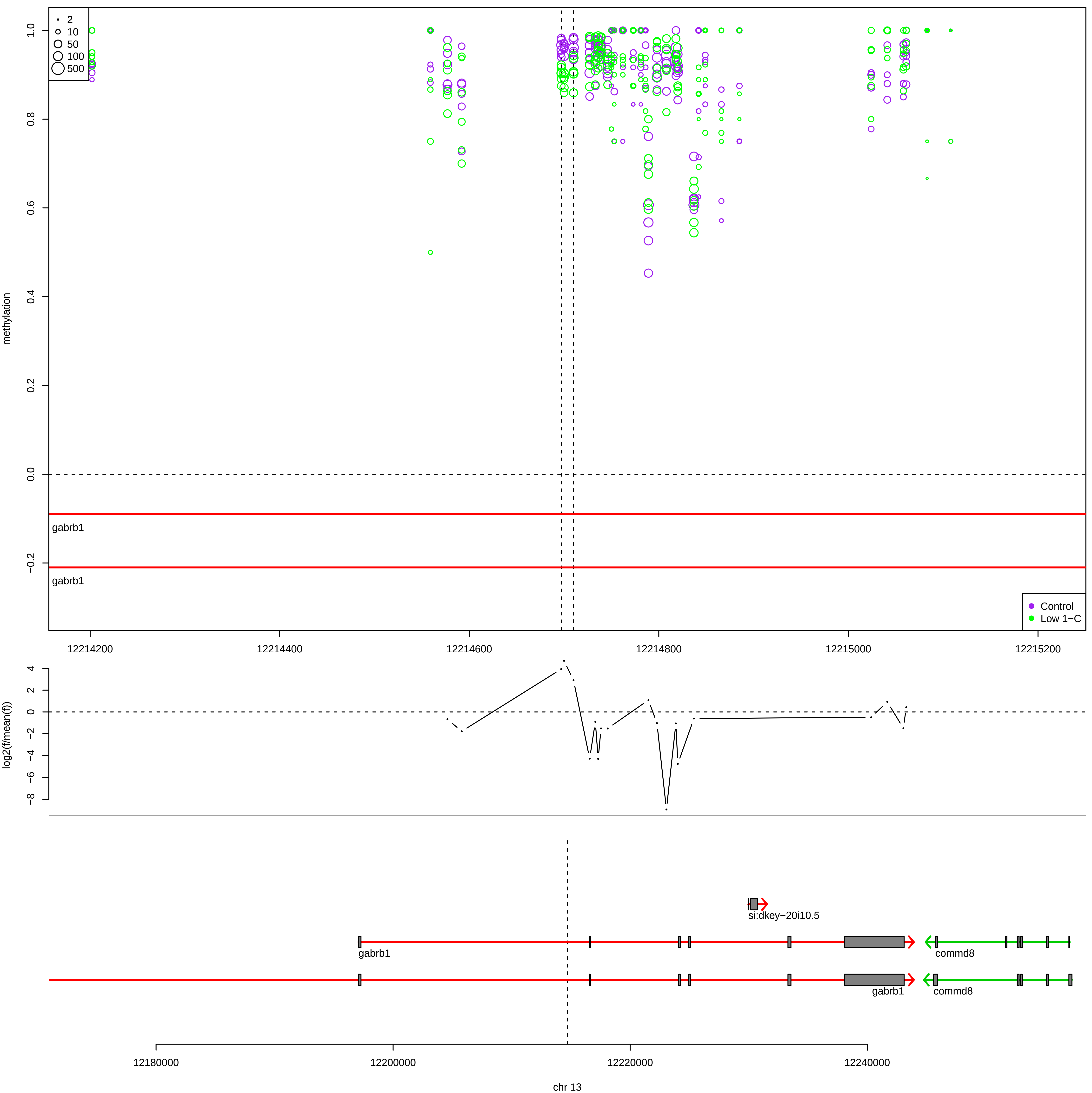

2
10
50
100
500
methylation
1.0
0.8
0.6
0.4
0.2
0.0
−0.2
gabrb1
gabrb1
Control
Low 1−C
12214200
12214400
12214600
12214800
12215000
12215200
log2(f/mean(f))
4
2
0
−2
−4
−6
−8
si:dkey−20i10.5
gabrb1
commd8
gabrb1
commd8
12180000
12200000
12220000
12240000
chr 13

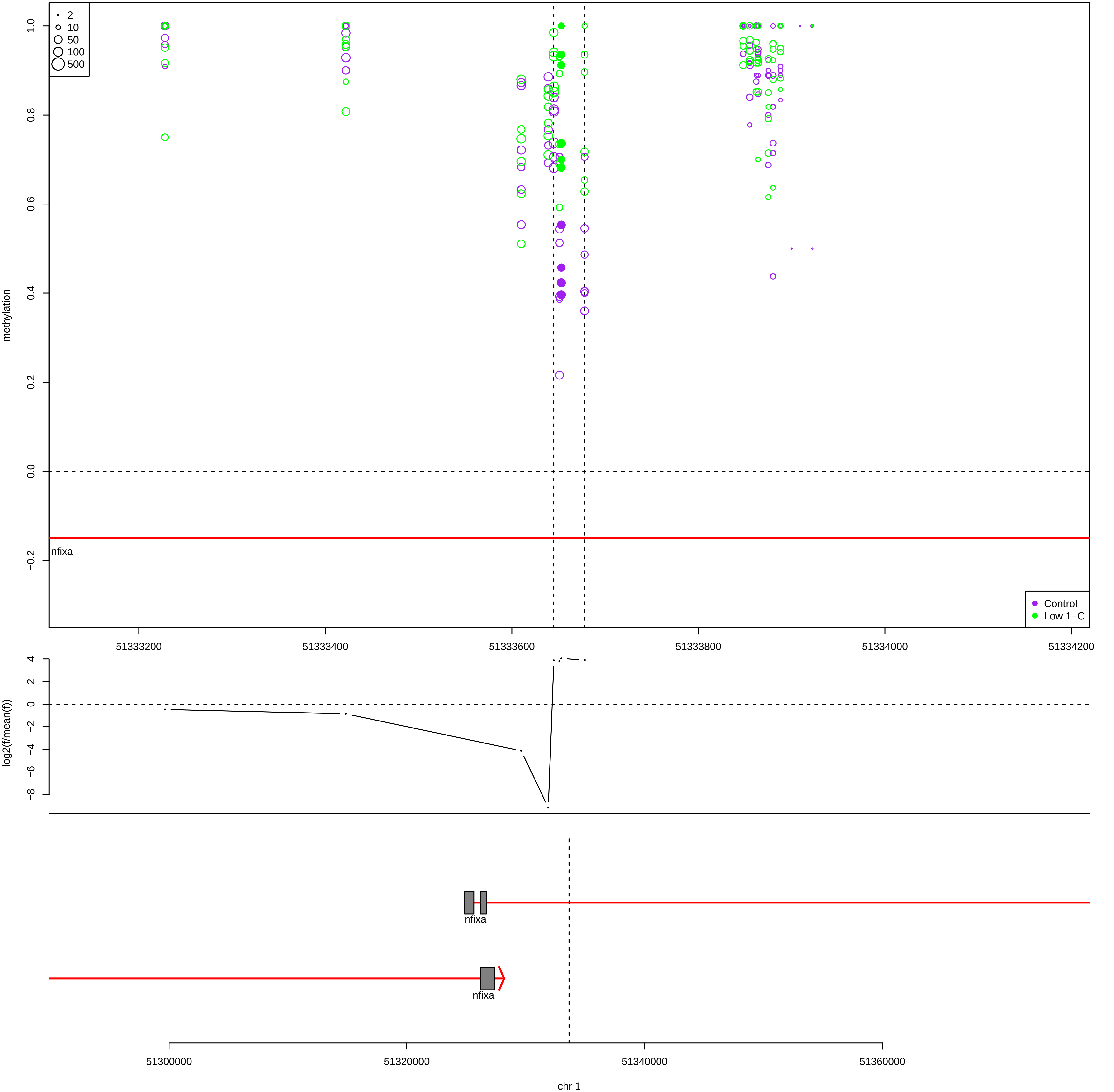

methylation
1.0
0.8
0.6
0.4
0.2
0.0
−0.2
2
10
50
100
500
nfixa
Control
Low 1−C
51333200
51333400
51333600
51333800
51334000
51334200
log2(f/mean(f))
4
2
0
−2
−4
−6
−8
nfixa
nfixa
51300000
51320000
51340000
51360000
chr 1

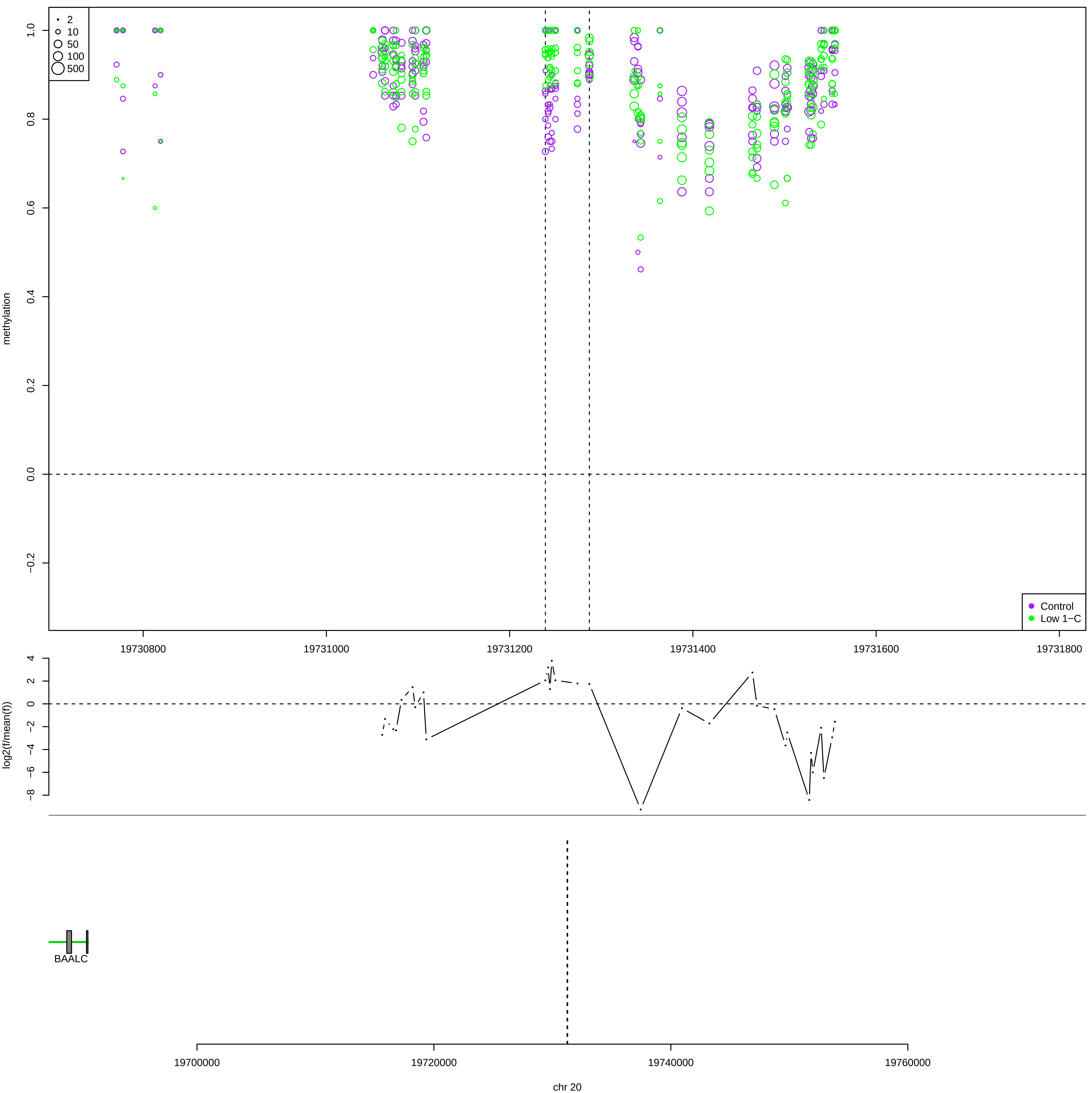

2
10
50
100
500
Control
Low 1−C
methylation
log2(f/mean(f))
19730800
19731000
19731200
19731400
19731600
19731800
BAALC
19700000
19720000
19740000
19760000
chr 20

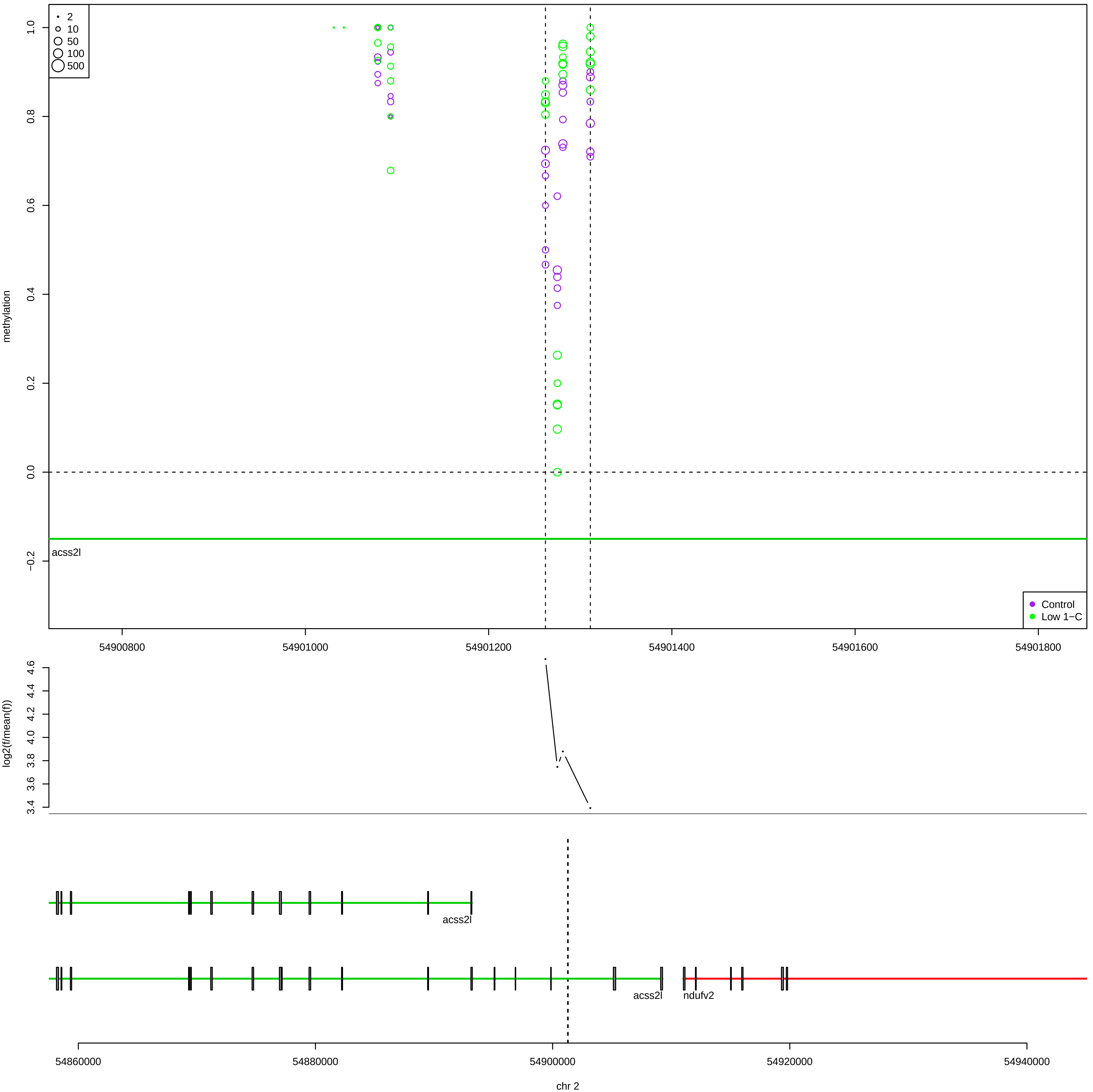

methylation
2
10
50
100
500
1.0
0.8
0.6
0.4
0.2
0.0
−0.2
acss2l
Control
Low 1−C
54900800
54901000
54901200
54901400
54901600
54901800
log2(f/mean(f))
4.6
4.4
4.2
4.0
3.8
3.6
3.4
acss2l
acss2l
ndufv2
54860000
54880000
54900000
54920000
54940000
chr 2

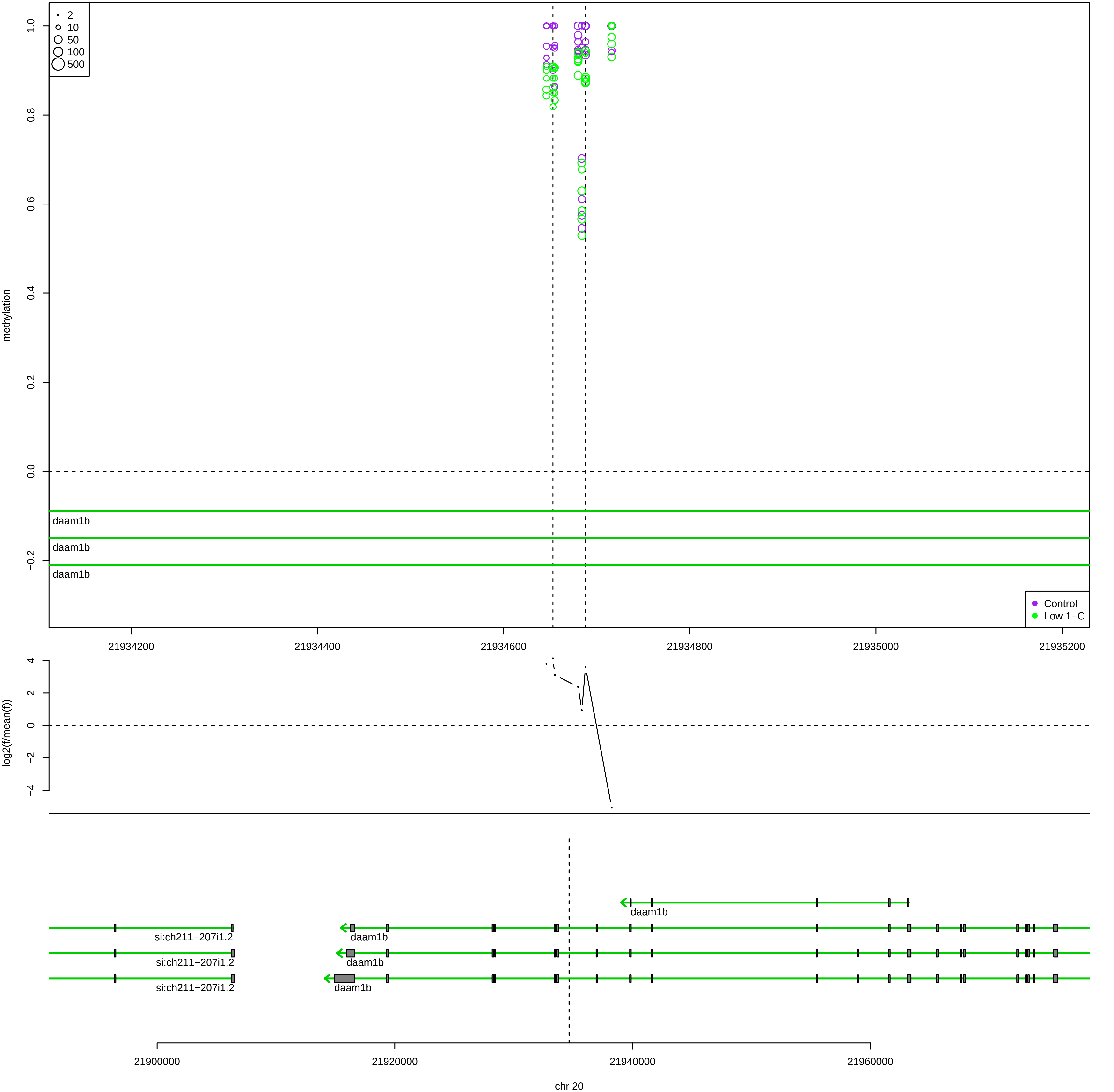

2
10
50
100
500
1.0
0.8
0.6
0.4
0.2
0.0
−0.2
methylation
daam1b
daam1b
daam1b
Control
Low 1−C
21934200
21934400
21934600
21934800
21935000
21935200
4
2
0
−2
−4
log2(f/mean(f))
daam1b
si:ch211−207i1.2
daam1b
si:ch211−207i1.2
daam1b
si:ch211−207i1.2
daam1b
21900000
21920000
21940000
21960000
chr 20

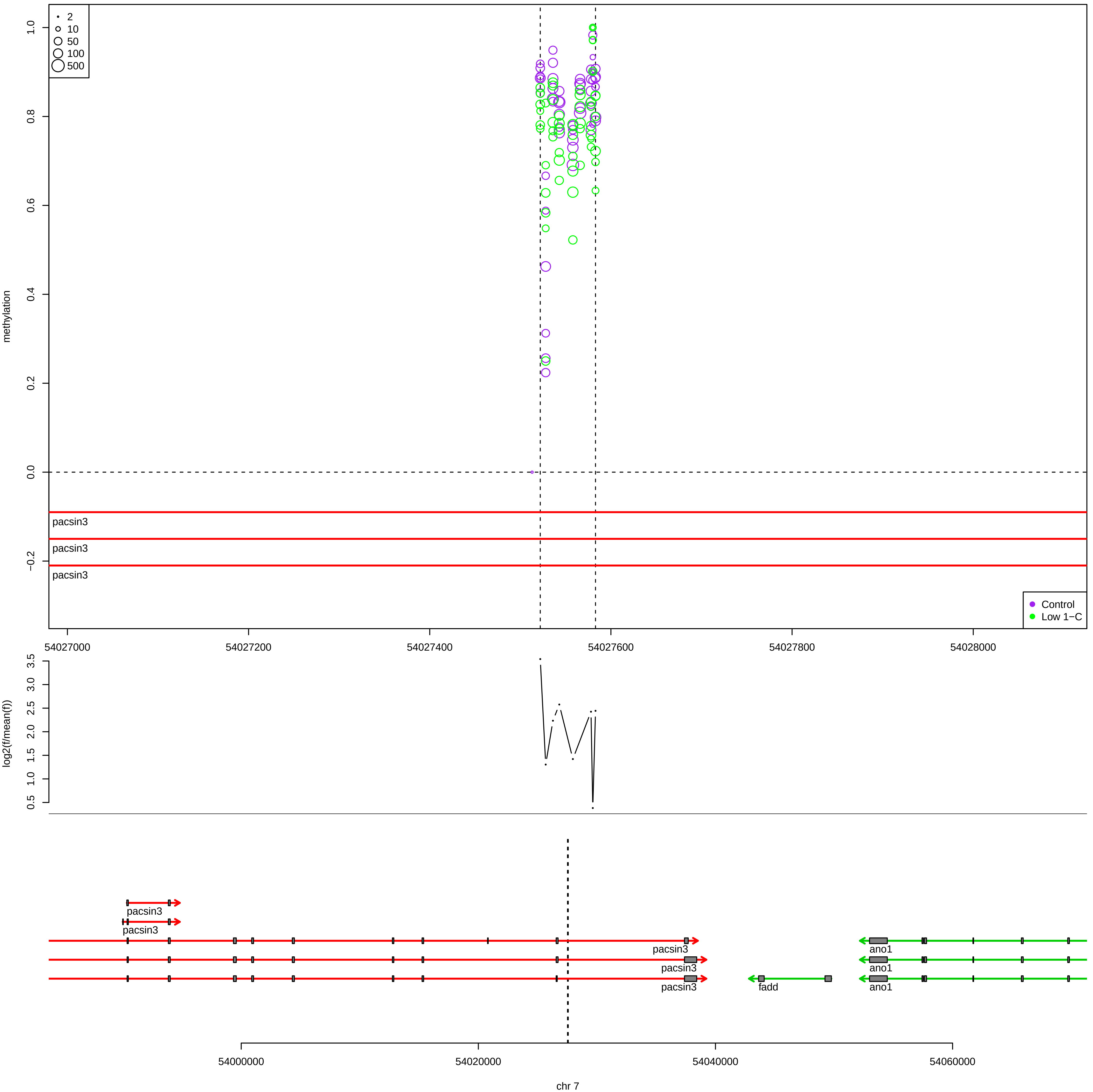

2
10
50
100
500
methylation
1.0
0.8
0.6
0.4
0.2
0.0
−0.2
pacsin3
pacsin3
pacsin3
Control
Low 1−C
54027000
54027200
54027400
54027600
54027800
54028000
log2(f/mean(f))
3.5
3.0
2.5
2.0
1.5
1.0
0.5
pacsin3
pacsin3
pacsin3
pacsin3
pacsin3
fadd
ano1
ano1
ano1
54000000
54020000
54040000
54060000
chr 7

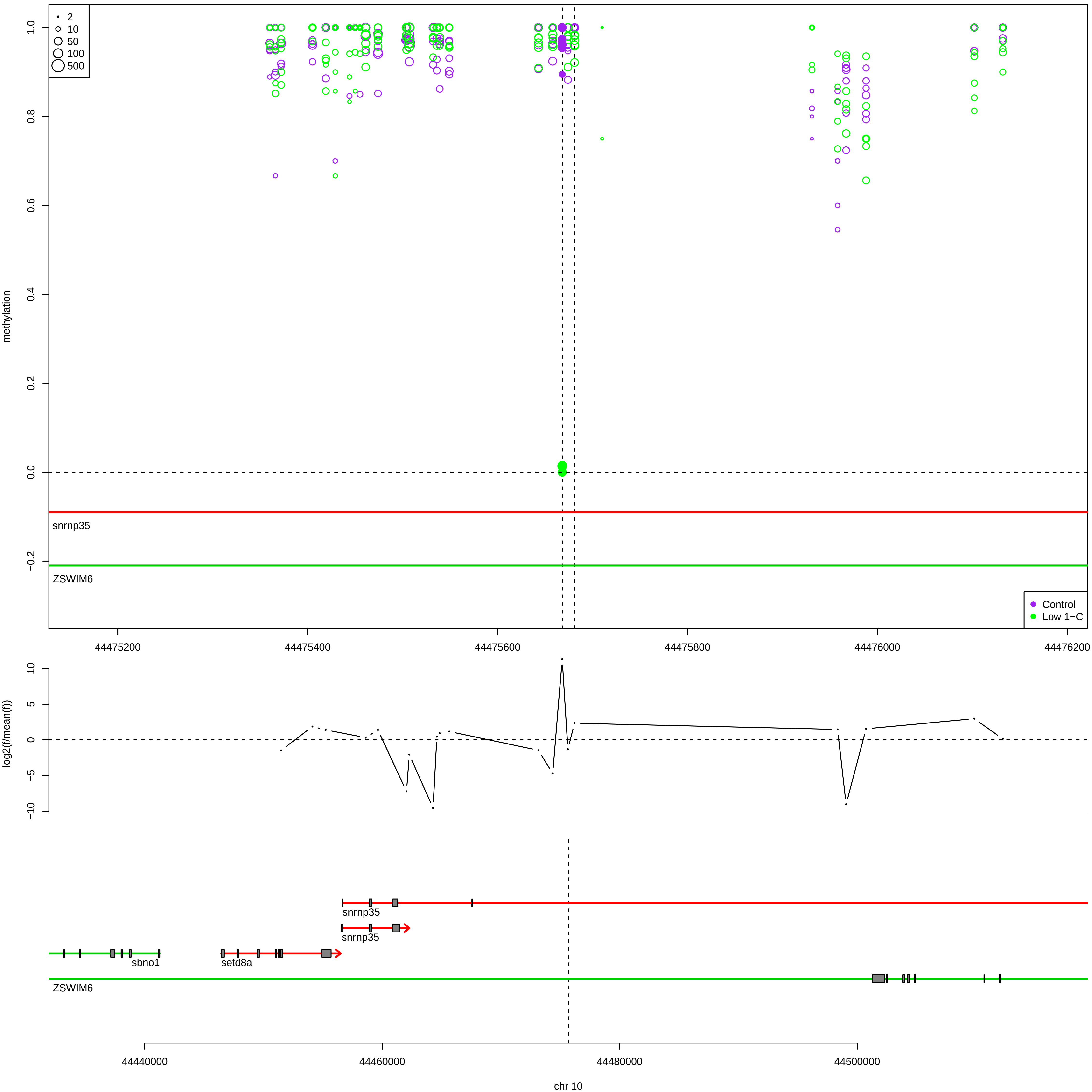

2
10
50
100
500
methylation
1.0
0.8
0.6
0.4
0.2
0.0
−0.2
snrnp35
ZSWIM6
Control
Low 1−C
44475200
44475400
44475600
44475800
44476000
44476200
log2(f/mean(f))
10
5
0
-5
-10
snrnp35
snrnp35
sbno1
setd8a
ZSWIM6
44440000
44460000
44480000
44500000
chr 10

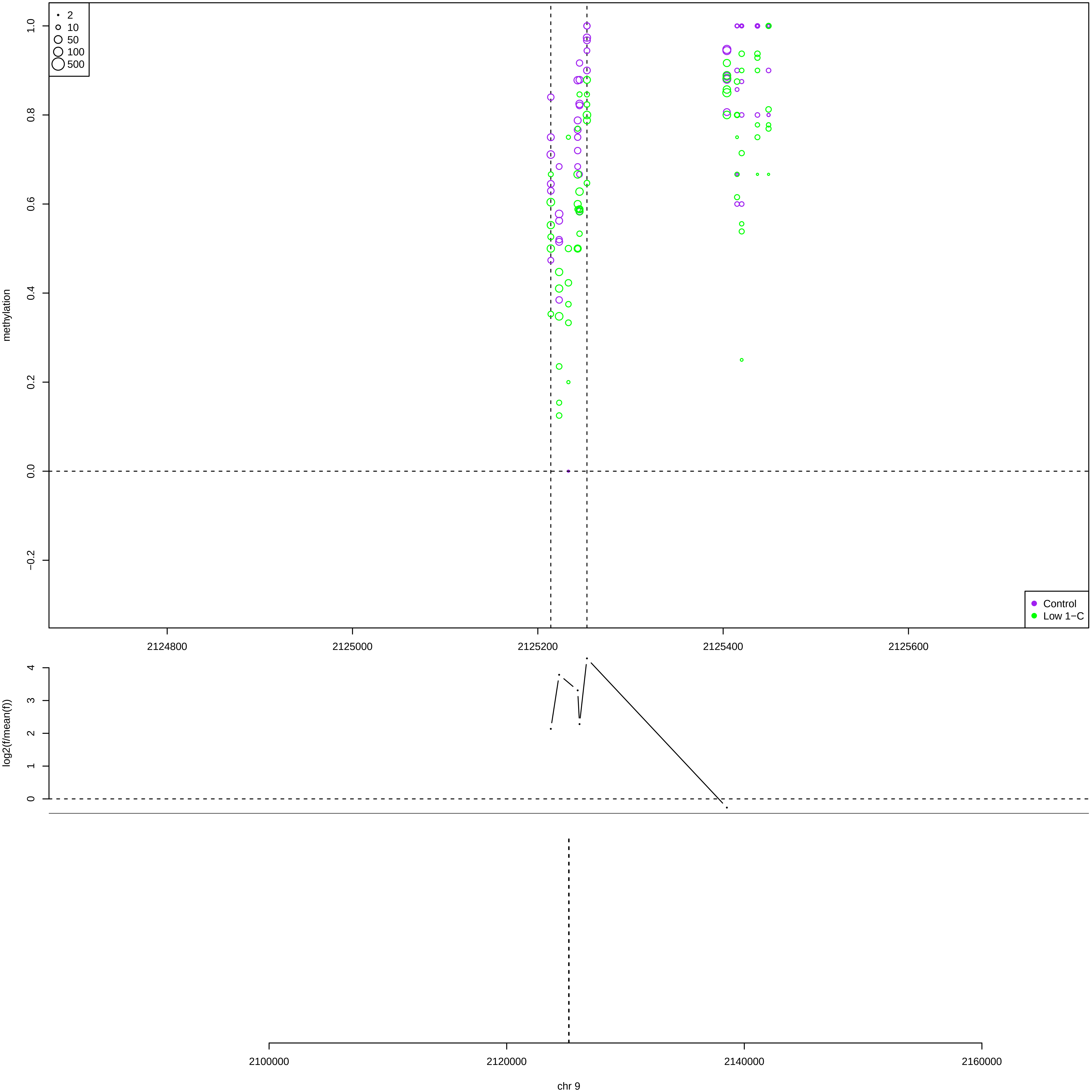

2
10
50
100
500
methylation
1.0
0.8
0.6
0.4
0.2
0.0
−0.2
Control
Low 1−C
2124800
2125000
2125200
2125400
2125600
log2(f/mean(f))
4
3
2
1
0
2100000
2120000
2140000
2160000
chr 9

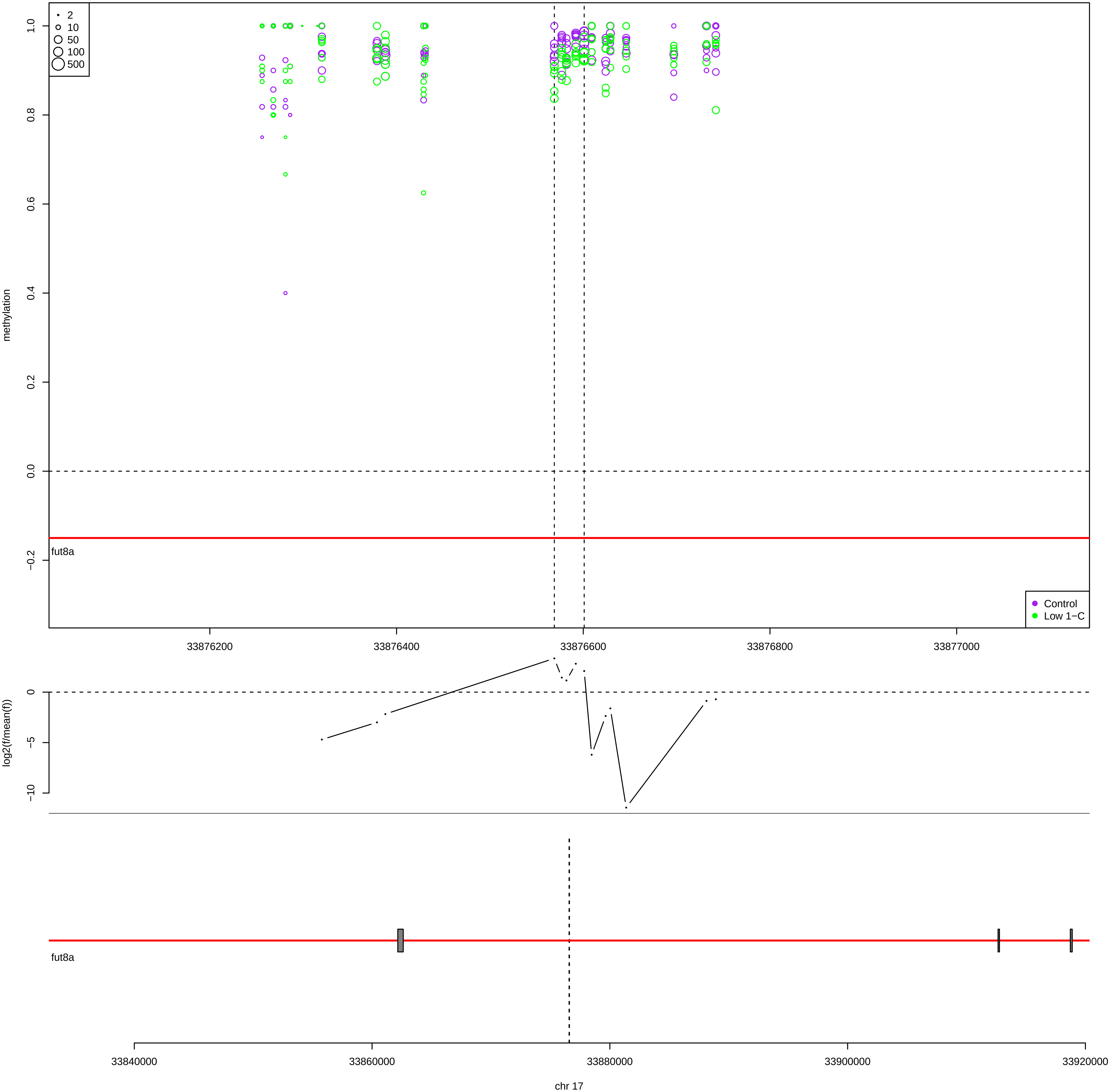

2
10
50
100
500
methylation
1.0
0.8
0.6
0.4
0.2
0.0
-0.2
fut8a
Control
Low 1−C
33876200
33876400
33876600
33876800
33877000
log2(f/mean(f))
0
-5
-10
fut8a
33840000
33860000
33880000
33900000
33920000
chr 17

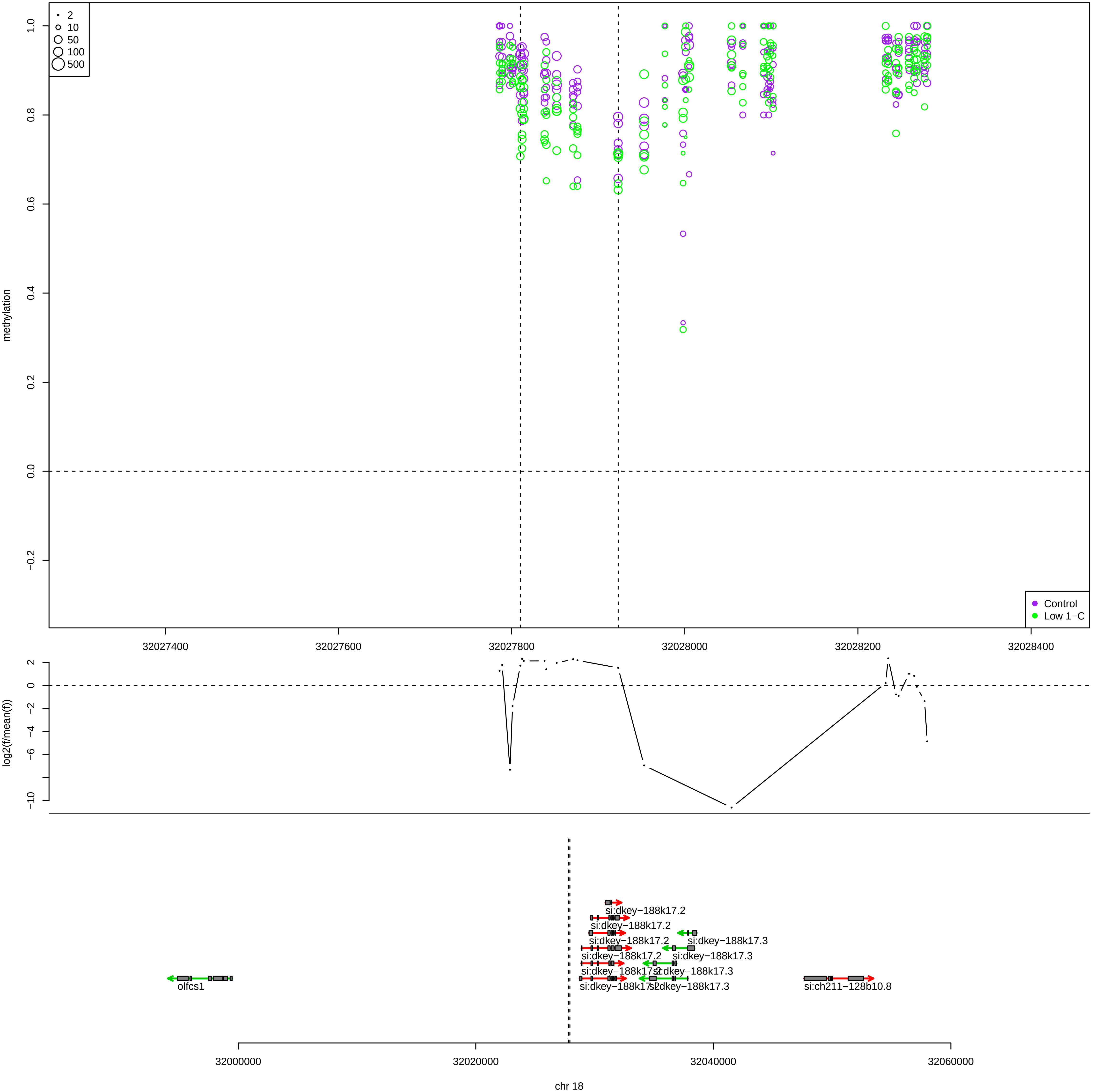

methylation
2
10
50
100
500
Control
Low 1−C
32027400
32027600
32027800
32028000
32028200
32028400
log2(f/mean(f))
si:dkey−188k17.2
si:dkey−188k17.3
olfcs1
si:ch211−128b10.8
32000000
32020000
32040000
32060000
chr 18

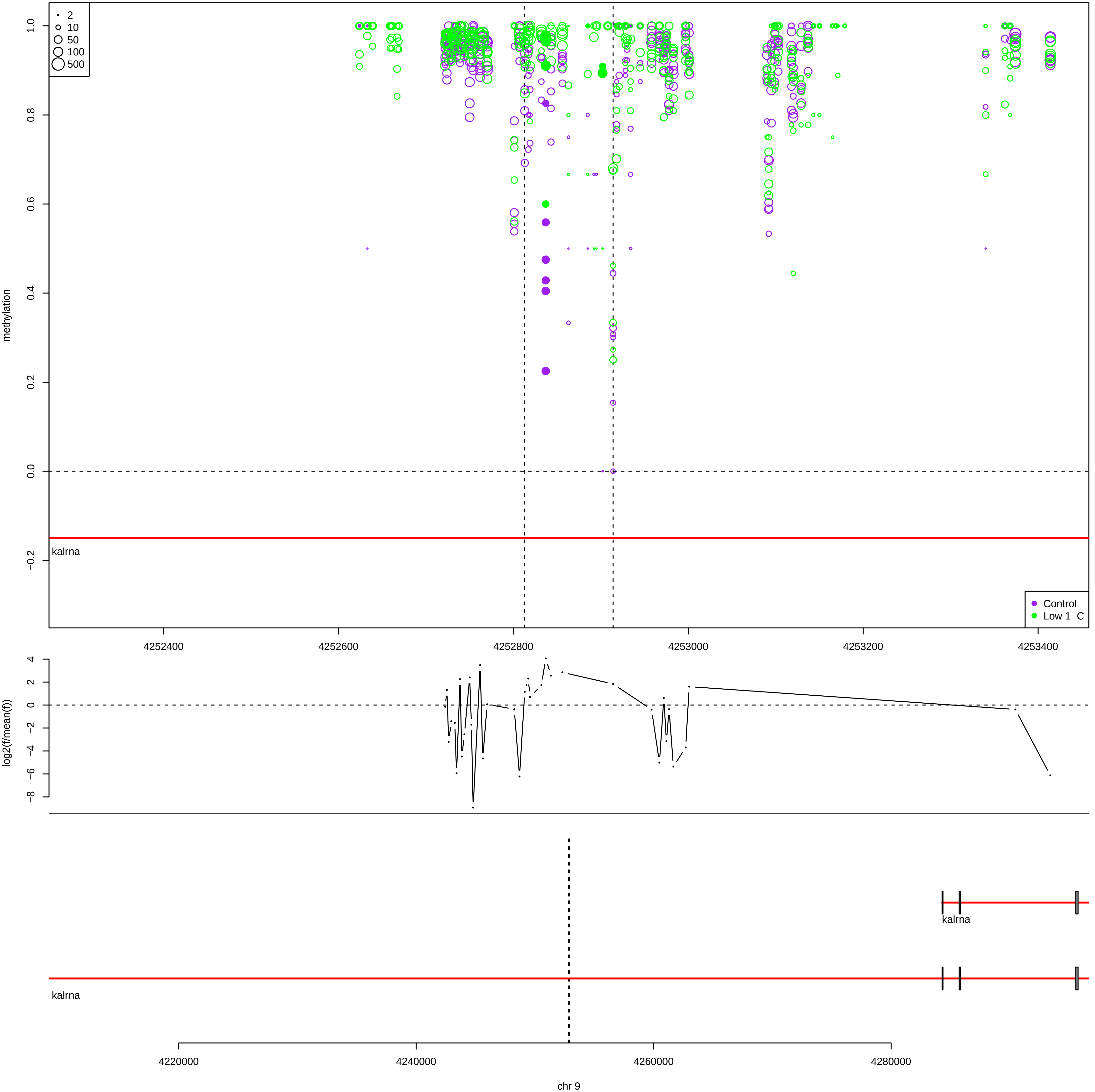

2
10
50
100
500
methylation
1.0
0.8
0.6
0.4
0.2
0.0
−0.2
kalrna
Control
Low 1−C
4252400
4252600
4252800
4253000
4253200
4253400
log2(f/mean(f))
4
2
0
−2
−4
−6
−8
kalrna
kalrna
4220000
4240000
4260000
4280000
chr 9

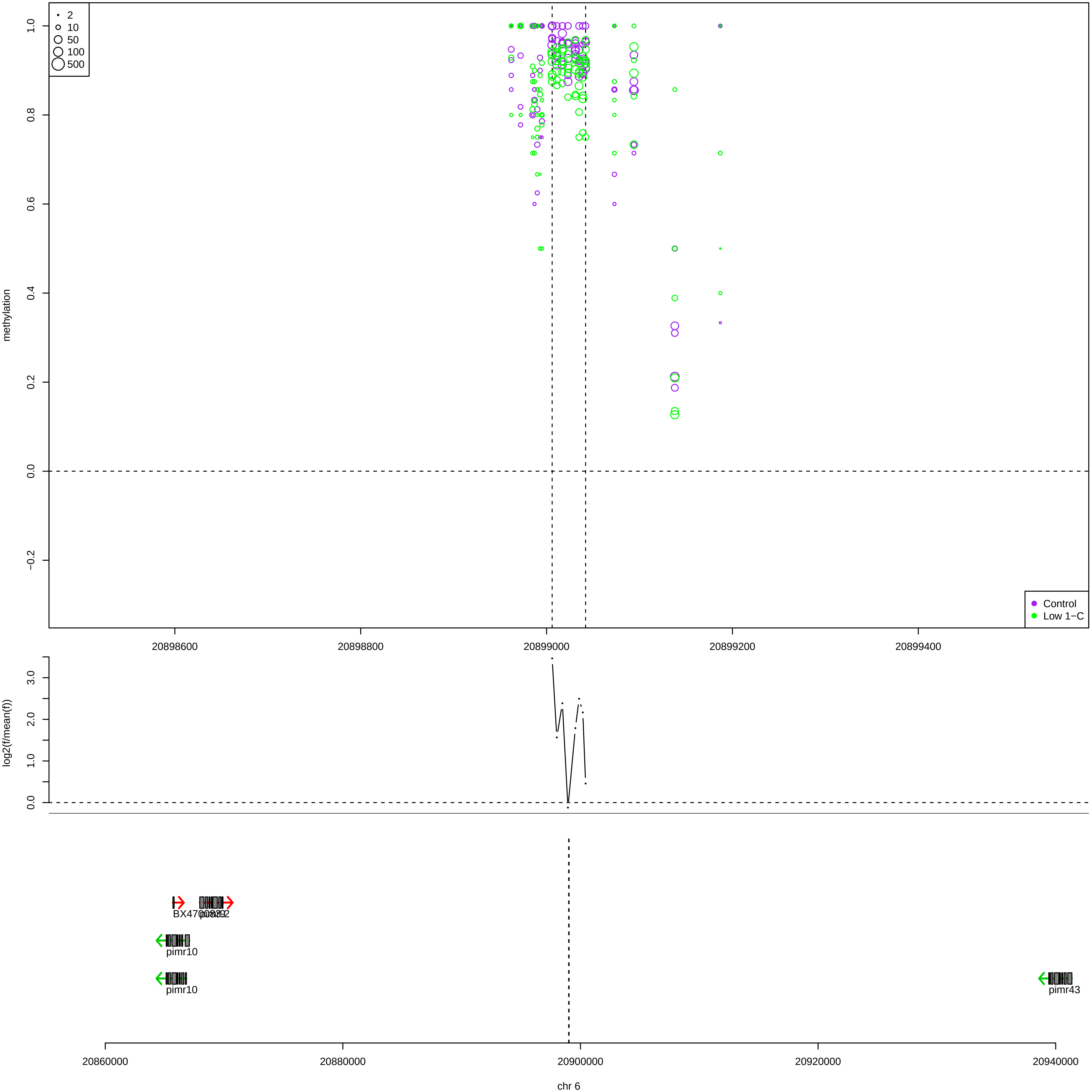

2
10
50
100
500
methylation
Control
Low 1−C
20898600
20898800
20899000
20899200
20899400
log2(f/mean(f))
BX470
pimr10
pimr10
pimr43
20860000
20880000
20900000
20920000
20940000
chr 6

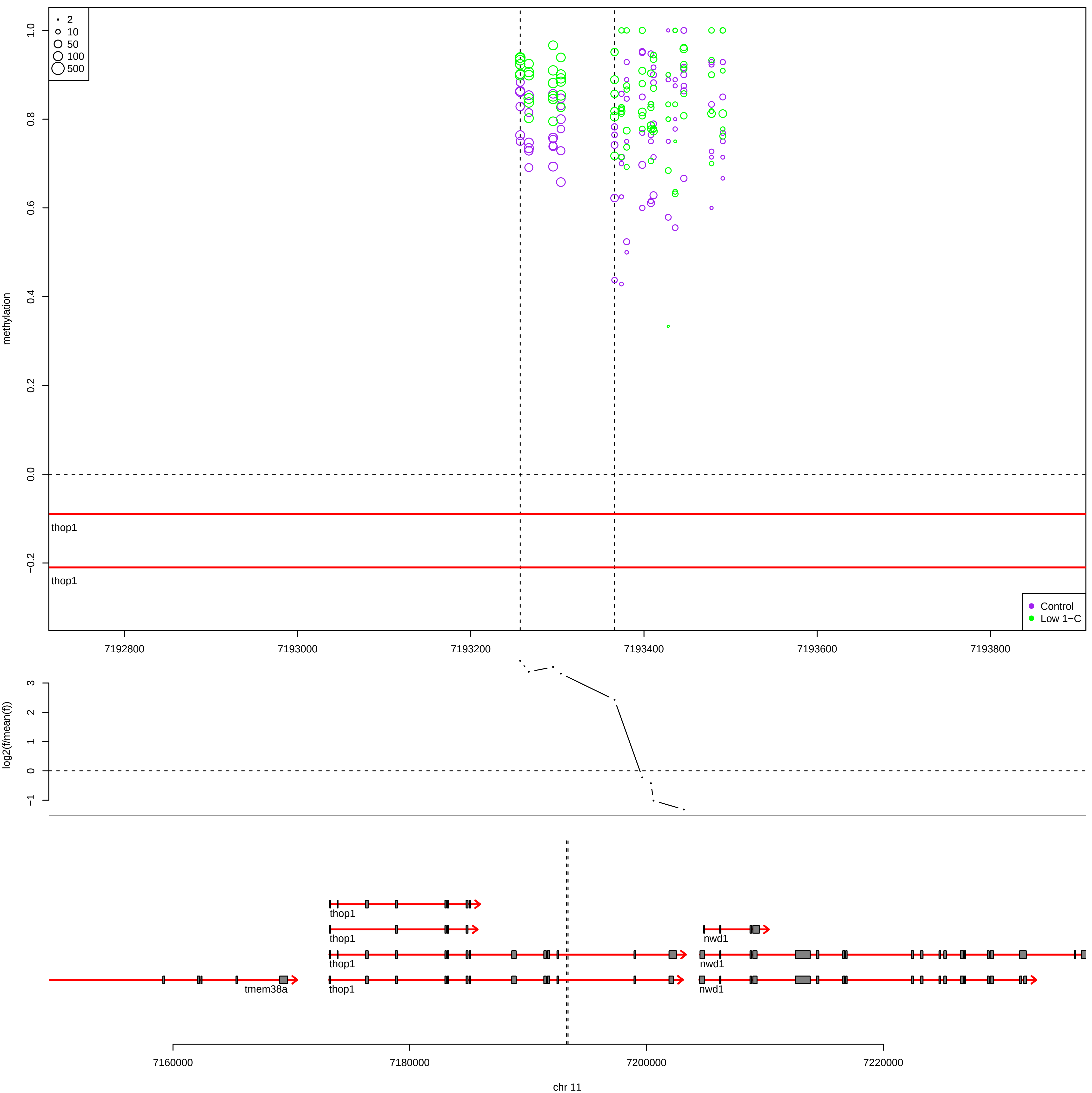

2
10
50
100
500
methylation
1.0
0.8
0.6
0.4
0.2
0.0
−0.2
thop1
thop1
Control
Low 1−C
7192800
7193000
7193200
7193400
7193600
7193800
log2(f/mean(f))
3
2
1
0
−1
thop1
thop1
thop1
thop1
nwd1
nwd1
nwd1
tmem38a
7160000
7180000
7200000
7220000
chr 11

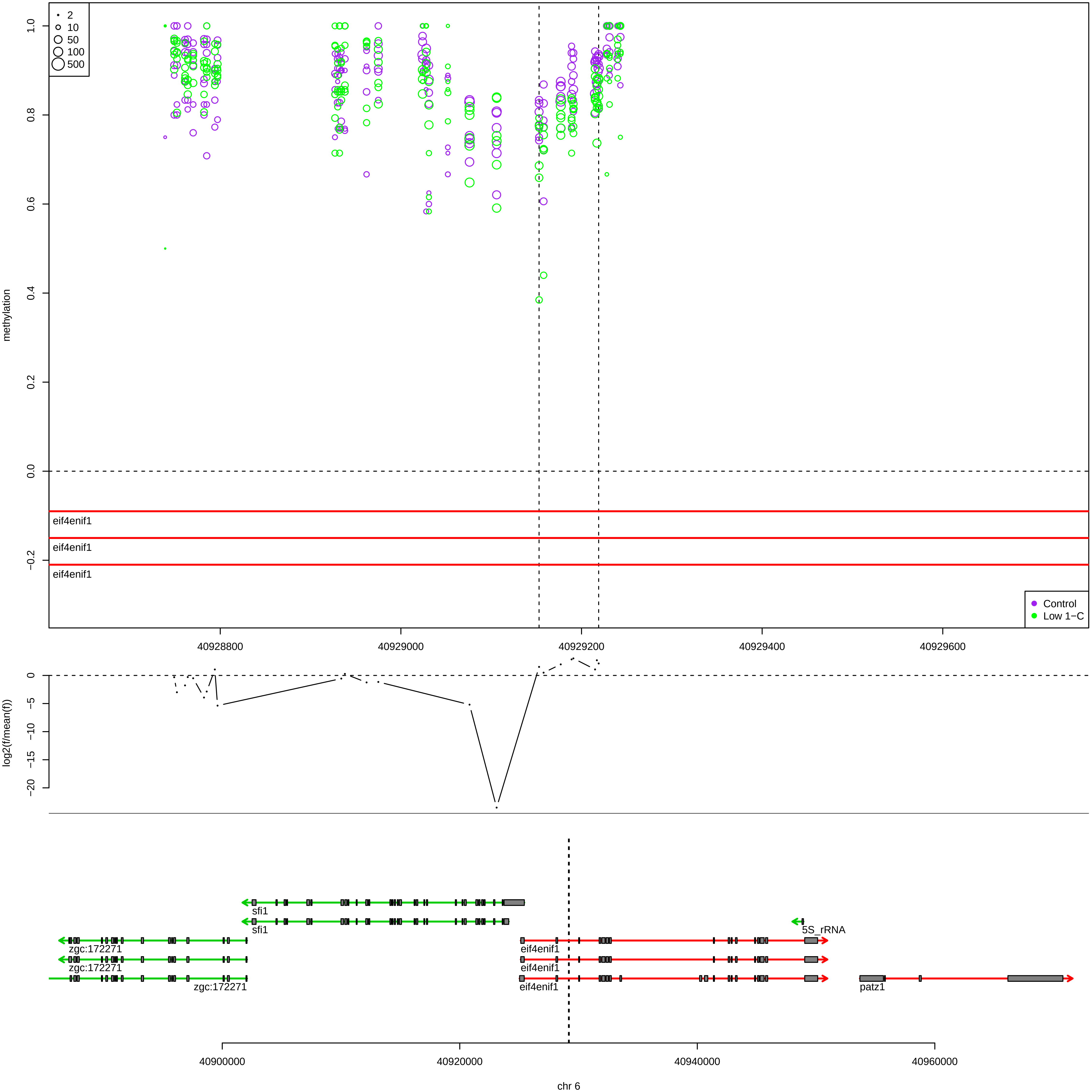

methylation
2
10
50
100
500
Control
Low 1−C
eif4enif1
eif4enif1
eif4enif1
1.0
0.8
0.6
0.4
0.2
0.0
−0.2
40928800
40929000
40929200
40929400
40929600
log2(f/mean(f))
0
−5
−10
−15
−20
sfi1
sfi1
5S_rRNA
zgc:172271
zgc:172271
zgc:172271
eif4enif1
eif4enif1
eif4enif1
patz1
40900000
40920000
40940000
40960000
chr 6

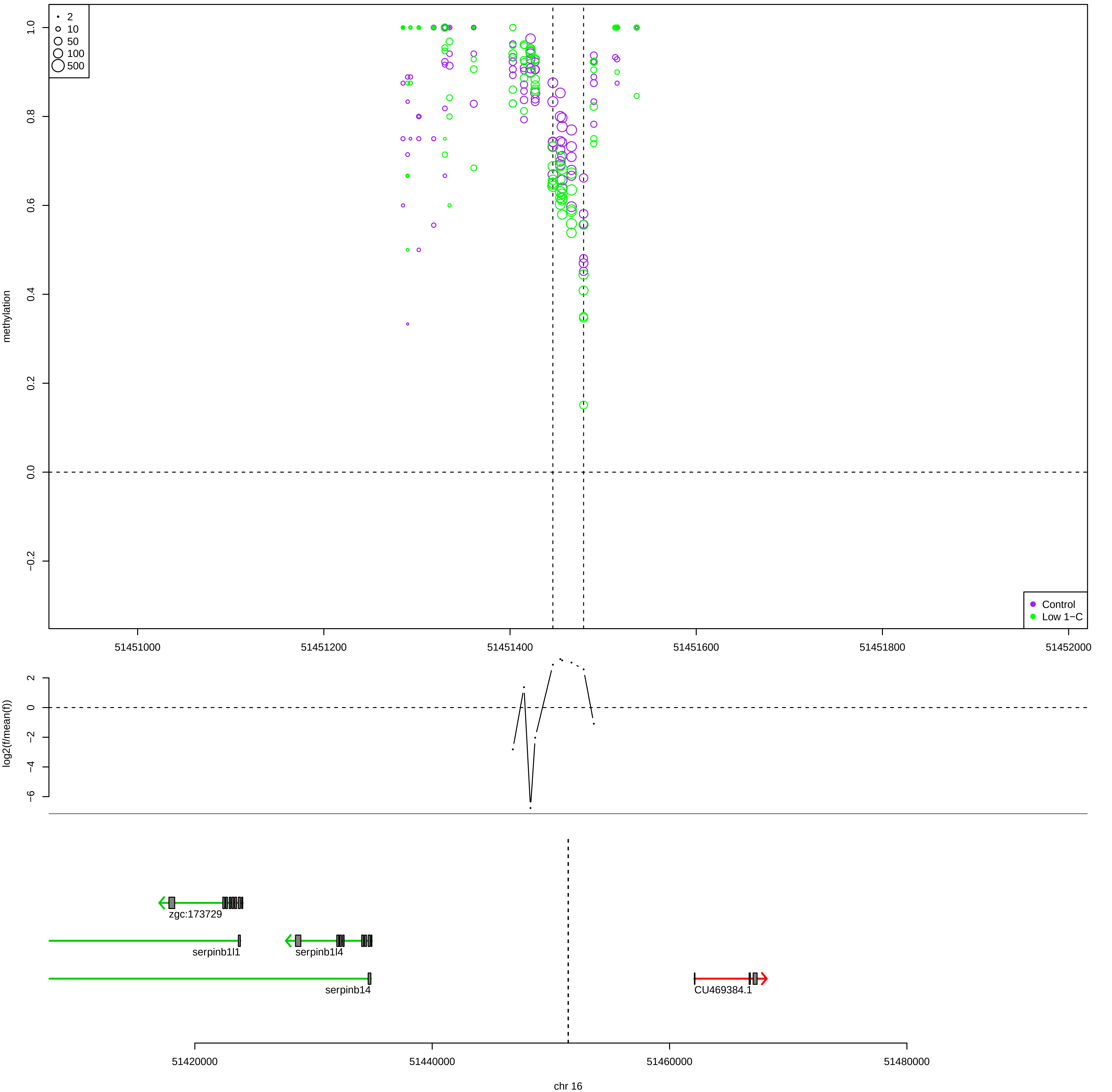

2
10
50
100
500
methylation
1.0
0.8
0.6
0.4
0.2
0.0
−0.2
Control
Low 1−C
51451000
51451200
51451400
51451600
51451800
51452000
log2(f/mean(f))
2
0
−2
−4
−6
zgc:173729
serpinb1l1
serpinb1l4
serpinb14
CU469384.1
51420000
51440000
51460000
51480000
chr 16

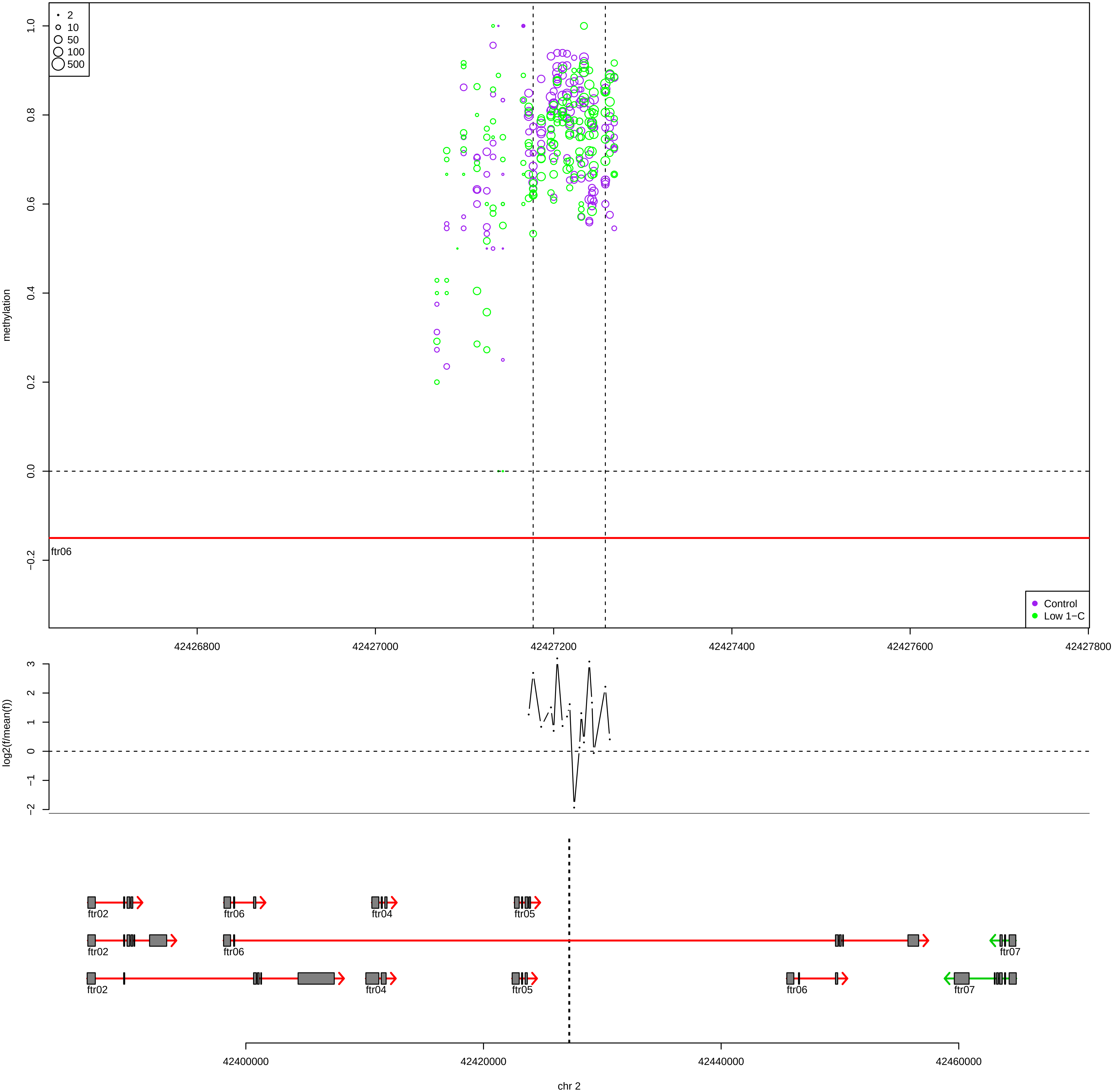

2
10
50
100
500
methylation
1.0
0.8
0.6
0.4
0.2
0.0
−0.2
ftr06
Control
Low 1−C
42426800
42427000
42427200
42427400
42427600
42427800
log2(f/mean(f))
3
2
1
0
−1
−2
ftr02
ftr06
ftr04
ftr05
ftr07
42400000
42420000
42440000
42460000
chr 2

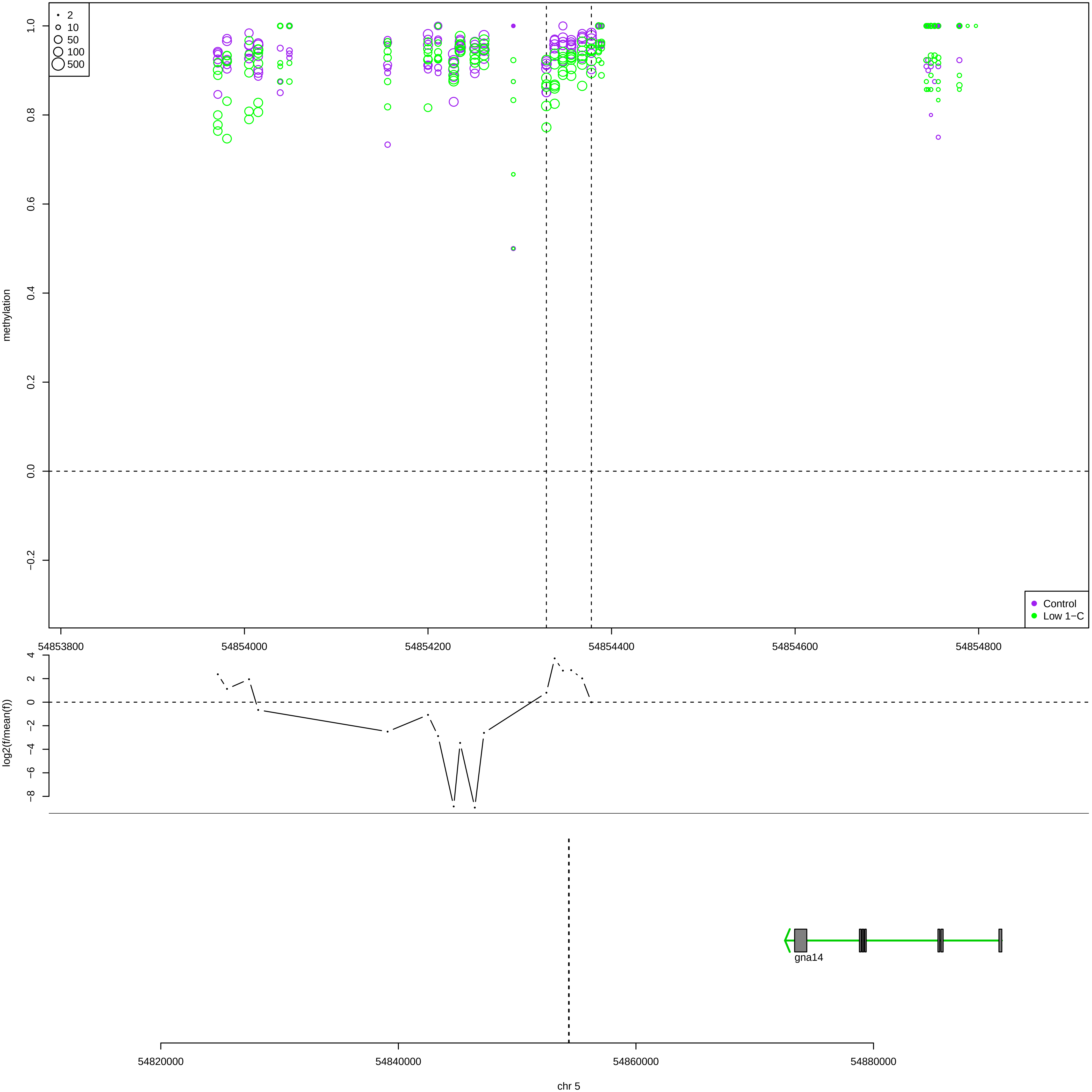

2
10
50
100
500
Control
Low 1−C
methylation
1.0
0.8
0.6
0.4
0.2
0.0
−0.2
54853800
54854000
54854200
54854400
54854600
54854800
log2(f/mean(f))
4
2
0
−2
−4
−6
−8
gna14
54820000
54840000
54860000
54880000
chr 5

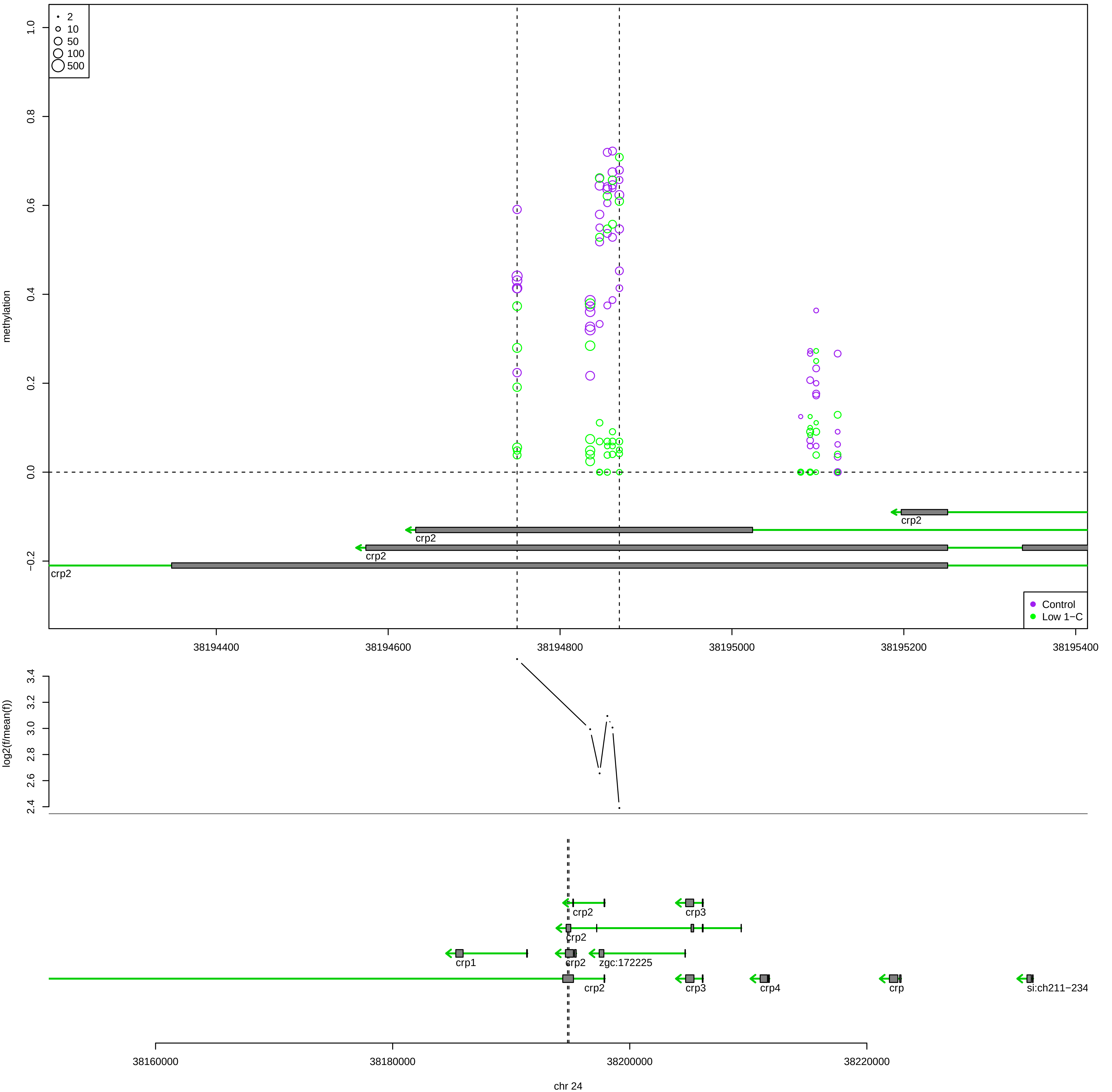

2
10
50
100
500
methylation
1.0
0.8
0.6
0.4
0.2
0.0
−0.2
crp2
crp2
crp2
crp2
Control
Low 1−C
38194400
38194600
38194800
38195000
38195200
38195400
log2(f/mean(f))
3.4
3.2
3.0
2.8
2.6
2.4
crp2
crp3
crp2
crp1
crp2
zgc:172225
crp2
crp3
crp4
crp
si:ch211−234
38160000
38180000
38200000
38220000
chr 24
