# Supplementary material for: Parental micronutrient deficiency distorts liver DNA methylation and expression of lipid genes associated with a fatty-liver-like phenotype in offspring
Source: Sci Rep. 2018 Feb 14;8:3055. doi: 10.1038/s41598-018-21211-5 (PMC5812986; doi:10.1038/s41598-018-21211-5)
Supplement: Supplementary file 3 — Figure S13 [file 41598_2018_21211_MOESM3_ESM.pdf]

Supplementary Info, figure S13:

Parental micronutrient deficiency distorts liver DNA methylation and expression of lipid genes associated with a fatty-liver-like phenotype in offspring

Kaja H. Skjærven[1*], Lars Martin Jakt[2], Jorge M.O. Fernandes[2], John Arne Dahl[3], Anne-Catrin Adam[1], Johanna Klughammer[4], Christoph Bock[4] and Marit Espe[1]

[1]*Institute of Marine Research, IMR, Norway*

[2]*Faculty of Biosciences and Aquaculture, Nord University, Norway*

[3]*Department of Microbiology, Oslo University Hospital, Norway*

[4]*CeMM Research Center for Molecular Medicine of the Austrian Academy of Sciences, 1090 Vienna, Austria*

[*]Corresponding author:

Dr. Kaja Helvik Skjærven

Institute of Marine Research (IMR),

PO Box 1870 Nordnes,

5817 Bergen, Norway

Tel.:+4741458362; fax: +4755905299.

E-mail address: ksk@hi.no

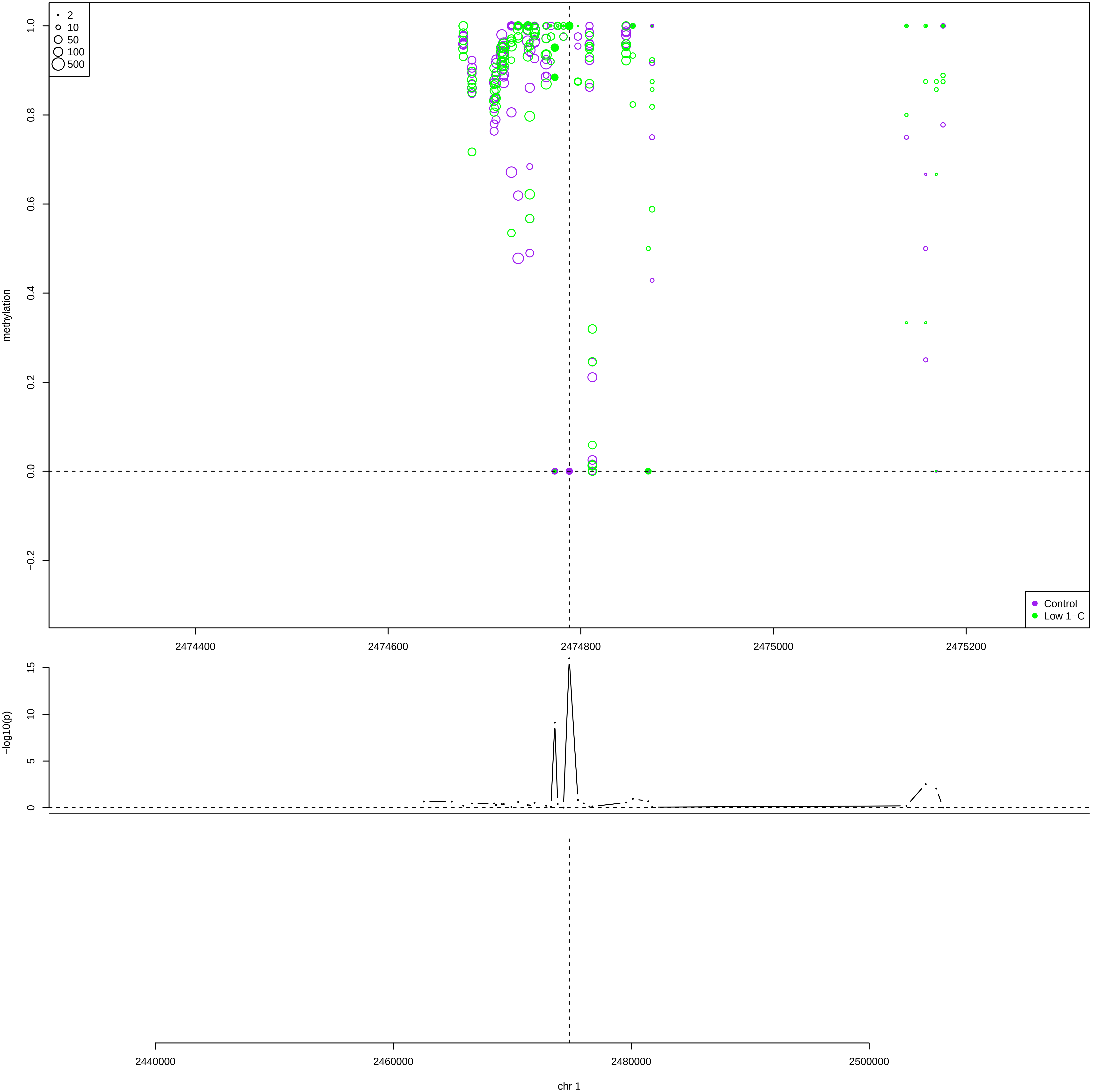

2
10
50
100
500
methylation
1.0
0.8
0.6
0.4
0.2
0.0
−0.2
Control
Low 1−C
2474400
2474600
2474800
2475000
2475200
−log10(p)
15
10
5
0
2440000
2460000
2480000
2500000
chr 1

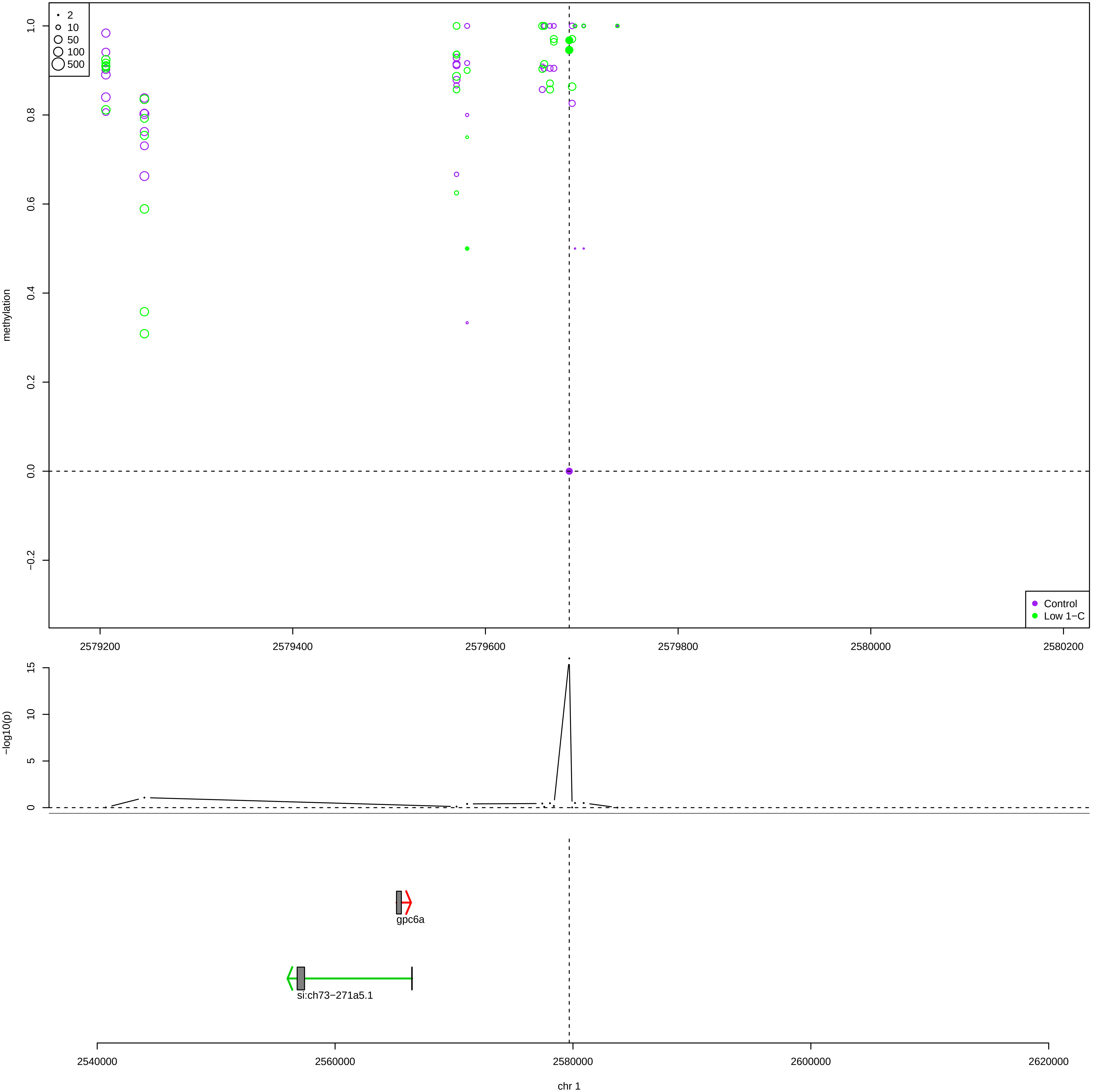

2
10
50
100
500
methylation
1.0
0.8
0.6
0.4
0.2
0.0
−0.2
Control
Low 1−C
2579200
2579400
2579600
2579800
2580000
2580200
−log10(p)
15
10
5
0
gpc6a
si:ch73−271a5.1
2540000
2560000
2580000
2600000
2620000
chr 1

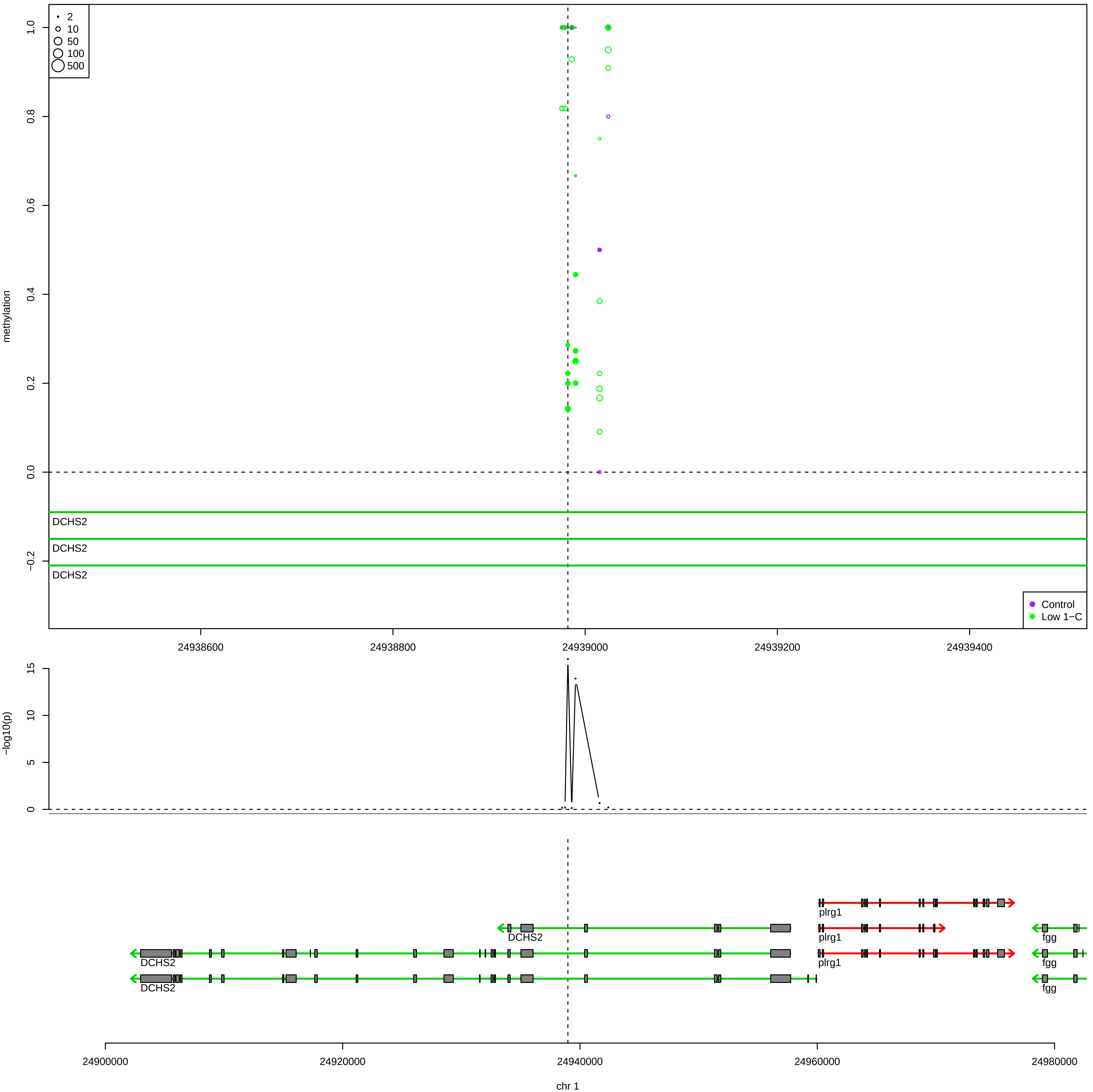

2
10
50
100
500
methylation
1.0
0.8
0.6
0.4
0.2
0.0
−0.2
DCHS2
DCHS2
DCHS2
Control
Low 1−C
24938600
24938800
24939000
24939200
24939400
−log10(p)
15
10
5
0
plrg1
plrg1
plrg1
DCHS2
DCHS2
DCHS2
fgg
fgg
fgg
24900000
24920000
24940000
24960000
24980000
chr 1

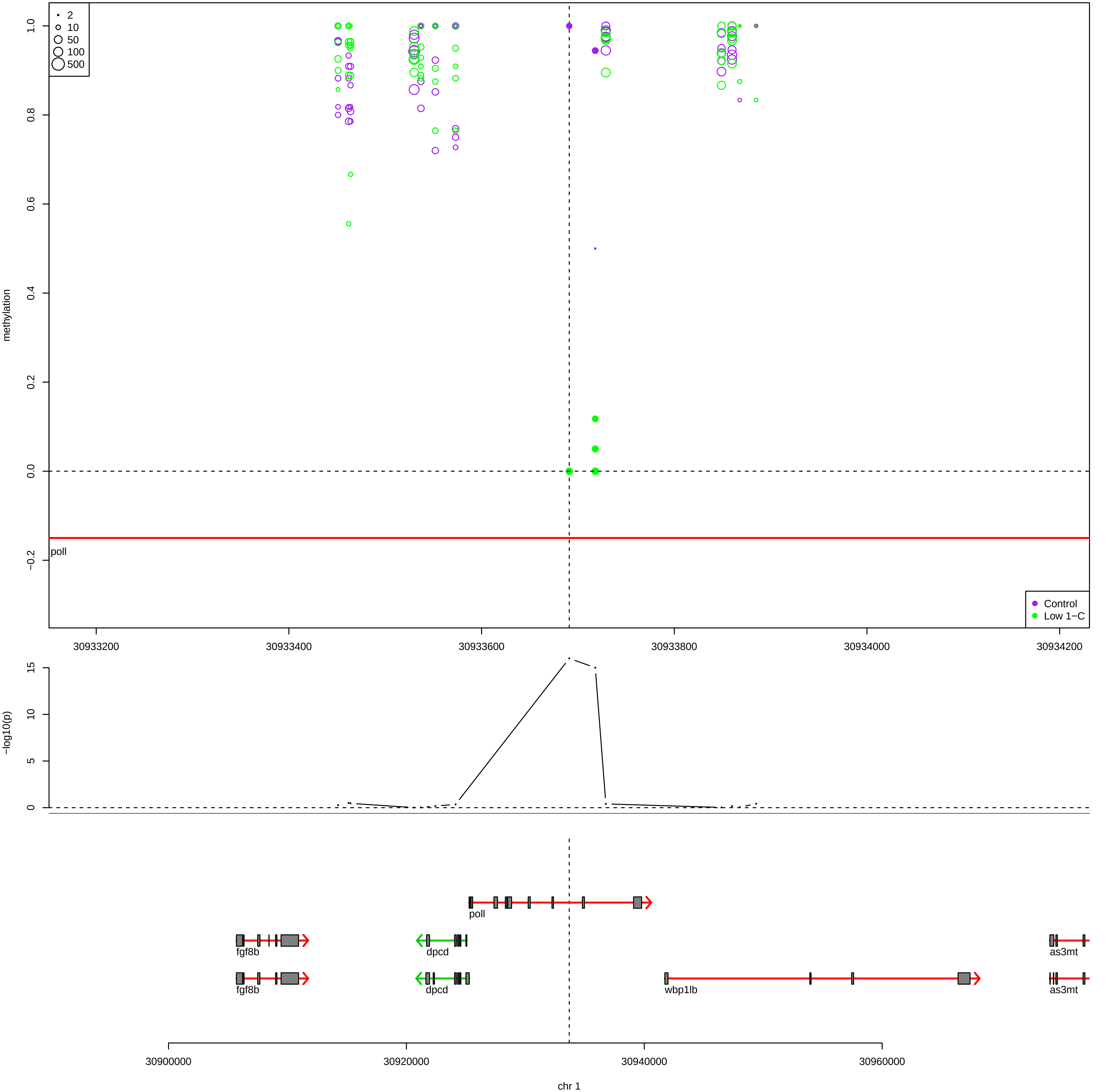

methylation
1.0
0.8
0.6
0.4
0.2
0.0
−0.2
2
10
50
100
500
poll
Control
Low 1−C
30933200
30933400
30933600
30933800
30934000
30934200
−log10(p)
15
10
5
0
poll
fgf8b
dpcd
as3mt
fgf8b
dpcd
wbp1lb
as3mt
30900000
30920000
30940000
30960000
chr 1

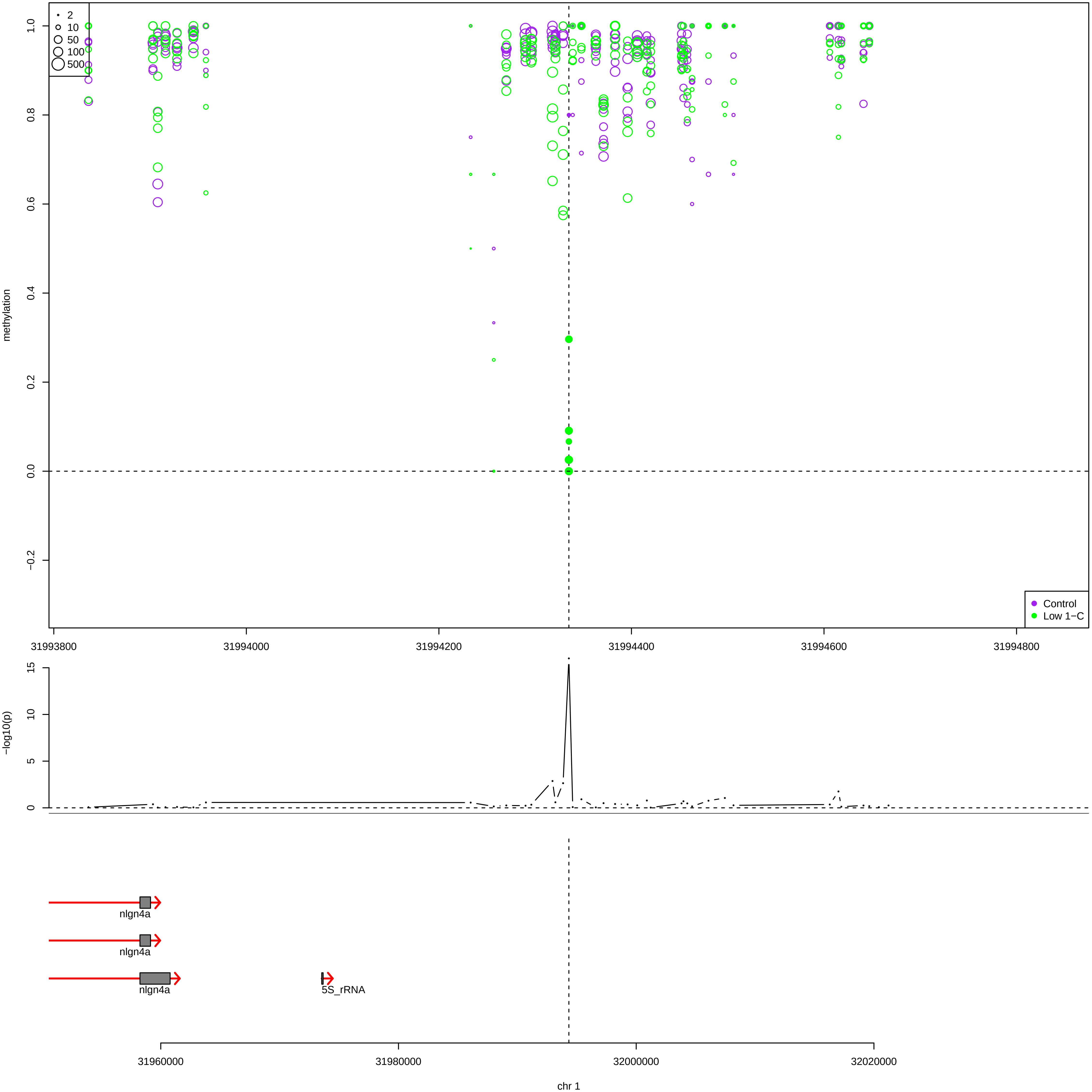

2
10
50
100
500
1.0
0.8
0.6
0.4
0.2
0.0
−0.2
methylation
Control
Low 1−C
31993800
31994000
31994200
31994400
31994600
31994800
15
10
5
0
−log10(p)
nlgn4a
nlgn4a
nlgn4a
5S_rRNA
31960000
31980000
32000000
32020000
chr 1

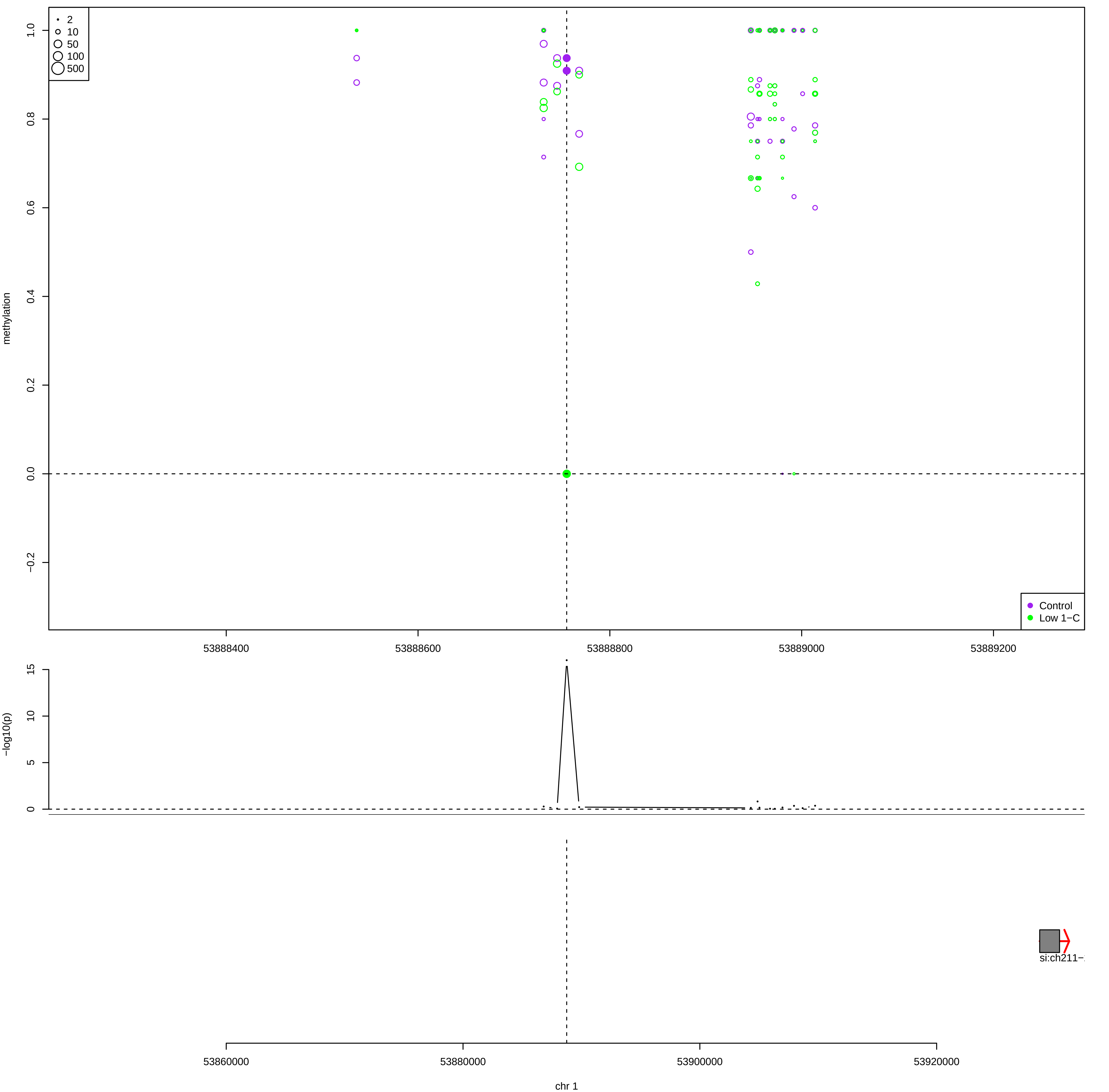

2
10
50
100
500
methylation
1.0
0.8
0.6
0.4
0.2
0.0
−0.2
Control
Low 1−C
53888400
53888600
53888800
53889000
53889200
−log10(p)
15
10
5
0
si:ch211−
53860000
53880000
53900000
53920000
chr 1

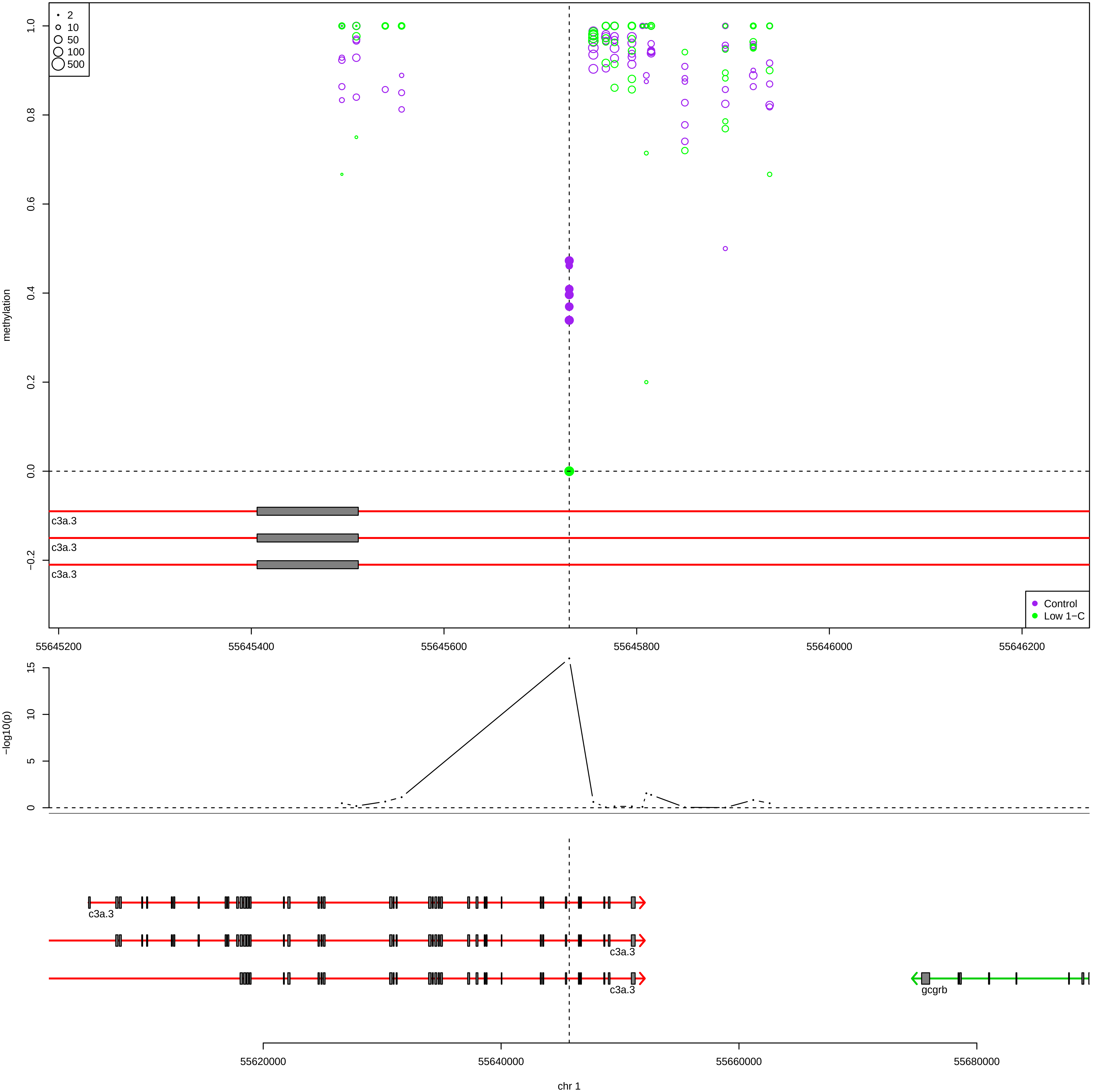

2
10
50
100
500
Control
Low 1−C
methylation
1.0
0.8
0.6
0.4
0.2
0.0
−0.2
c3a.3
c3a.3
c3a.3
55645200
55645400
55645600
55645800
55646000
55646200
−log10(p)
15
10
5
0
c3a.3
c3a.3
c3a.3
gcgrb
55620000
55640000
55660000
55680000
chr 1

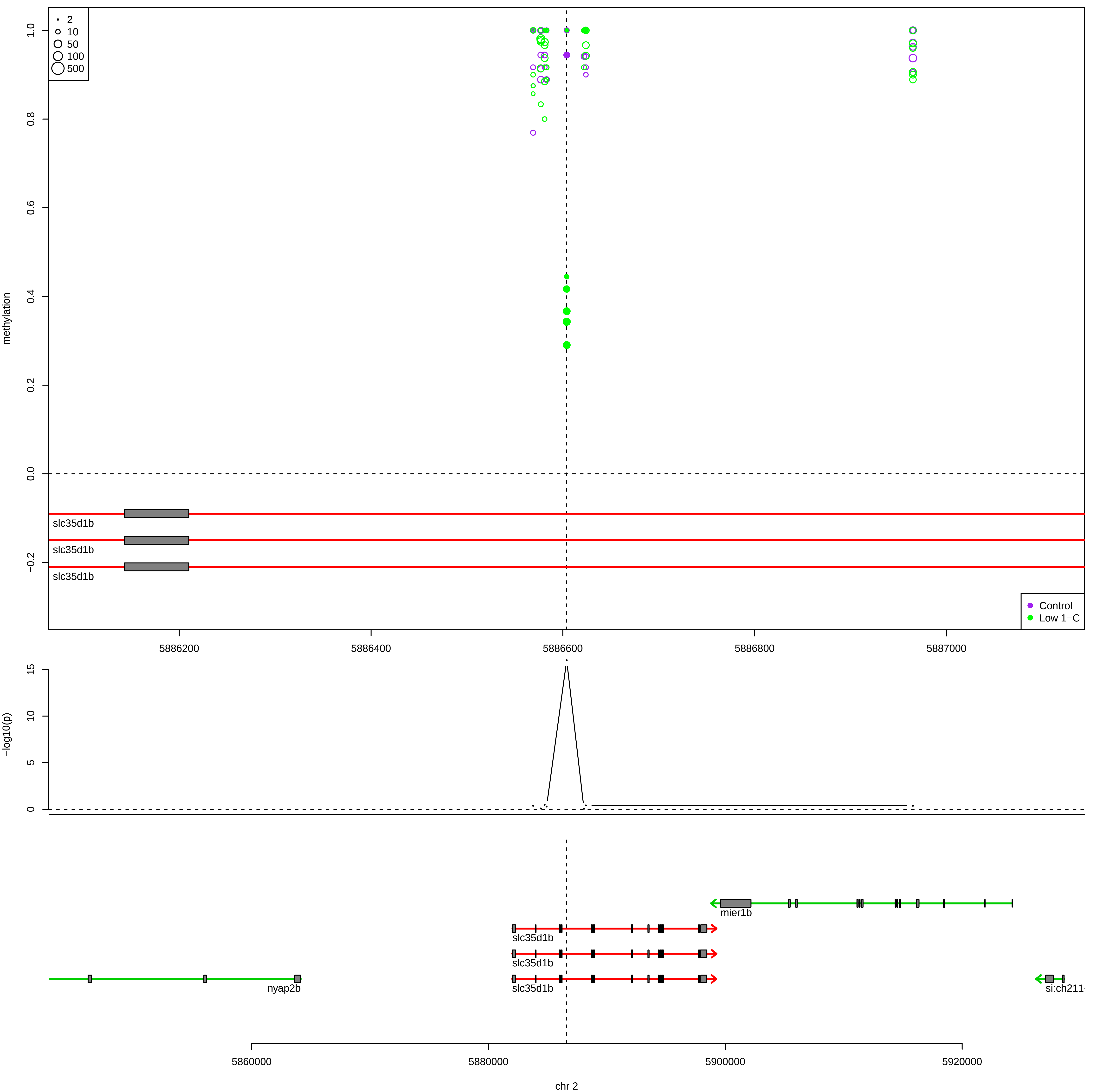

methylation
1.0
0.8
0.6
0.4
0.2
0.0
−0.2
2
10
50
100
500
slc35d1b
slc35d1b
slc35d1b
Control
Low 1−C
5886200
5886400
5886600
5886800
5887000
−log10(p)
15
10
5
0
mier1b
slc35d1b
slc35d1b
slc35d1b
nyap2b
si:ch211
5860000
5880000
5900000
5920000
chr 2

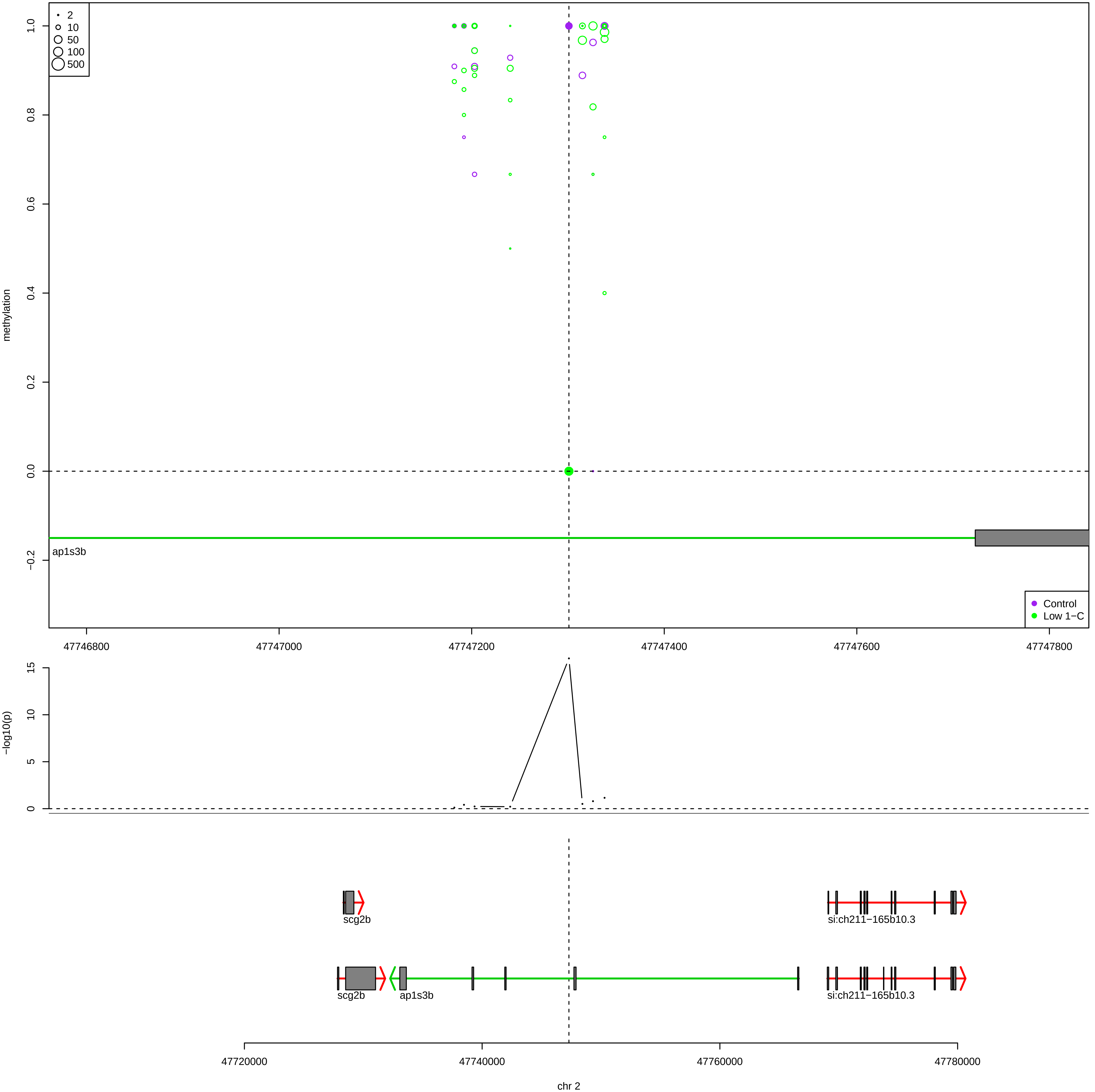

2
10
50
100
500
methylation
1.0
0.8
0.6
0.4
0.2
0.0
−0.2
ap1s3b
Control
Low 1−C
47746800
47747000
47747200
47747400
47747600
47747800
−log10(p)
15
10
5
0
scg2b
si:ch211−165b10.3
scg2b
ap1s3b
si:ch211−165b10.3
47720000
47740000
47760000
47780000
chr 2

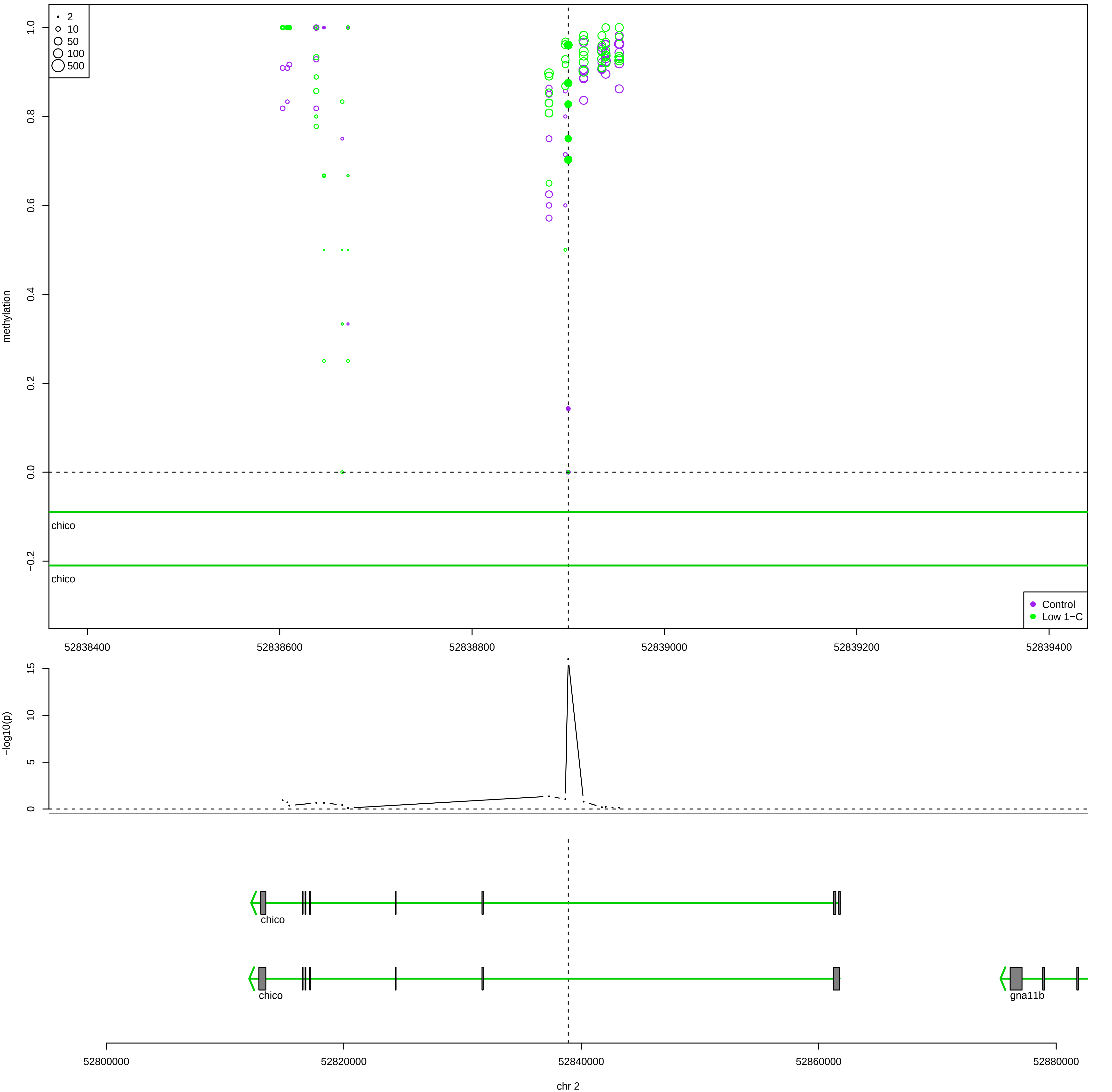

2
10
50
100
500
methylation
1.0
0.8
0.6
0.4
0.2
0.0
−0.2
chico
chico
Control
Low 1−C
52838400
52838600
52838800
52839000
52839200
52839400
−log10(p)
15
10
5
0
chico
chico
gna11b
52800000
52820000
52840000
52860000
52880000
chr 2

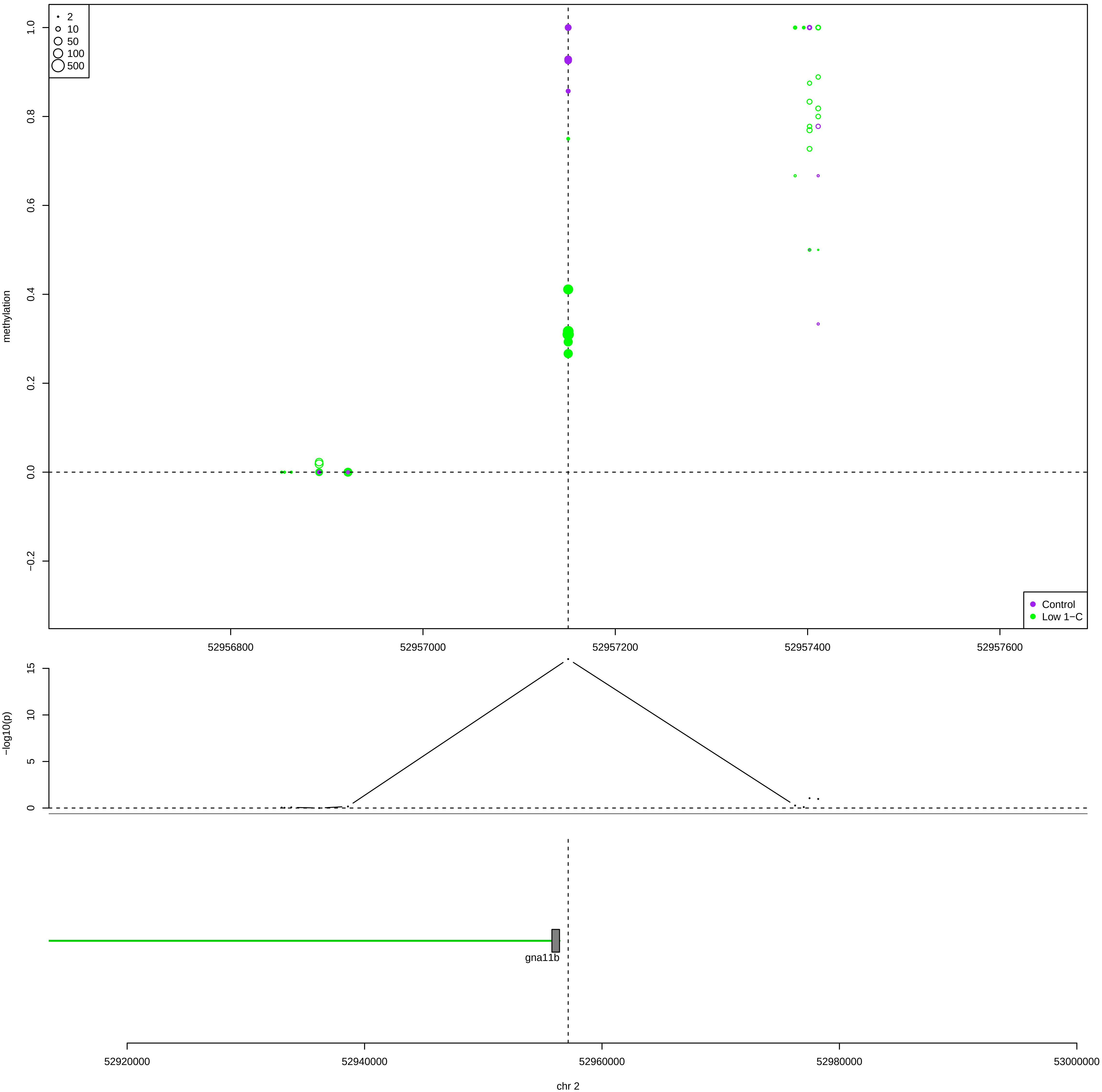

2
10
50
100
500
methylation
1.0
0.8
0.6
0.4
0.2
0.0
−0.2
Control
Low 1−C
52956800
52957000
52957200
52957400
52957600
−log10(p)
15
10
5
0
gna11b
52920000
52940000
52960000
52980000
53000000
chr 2

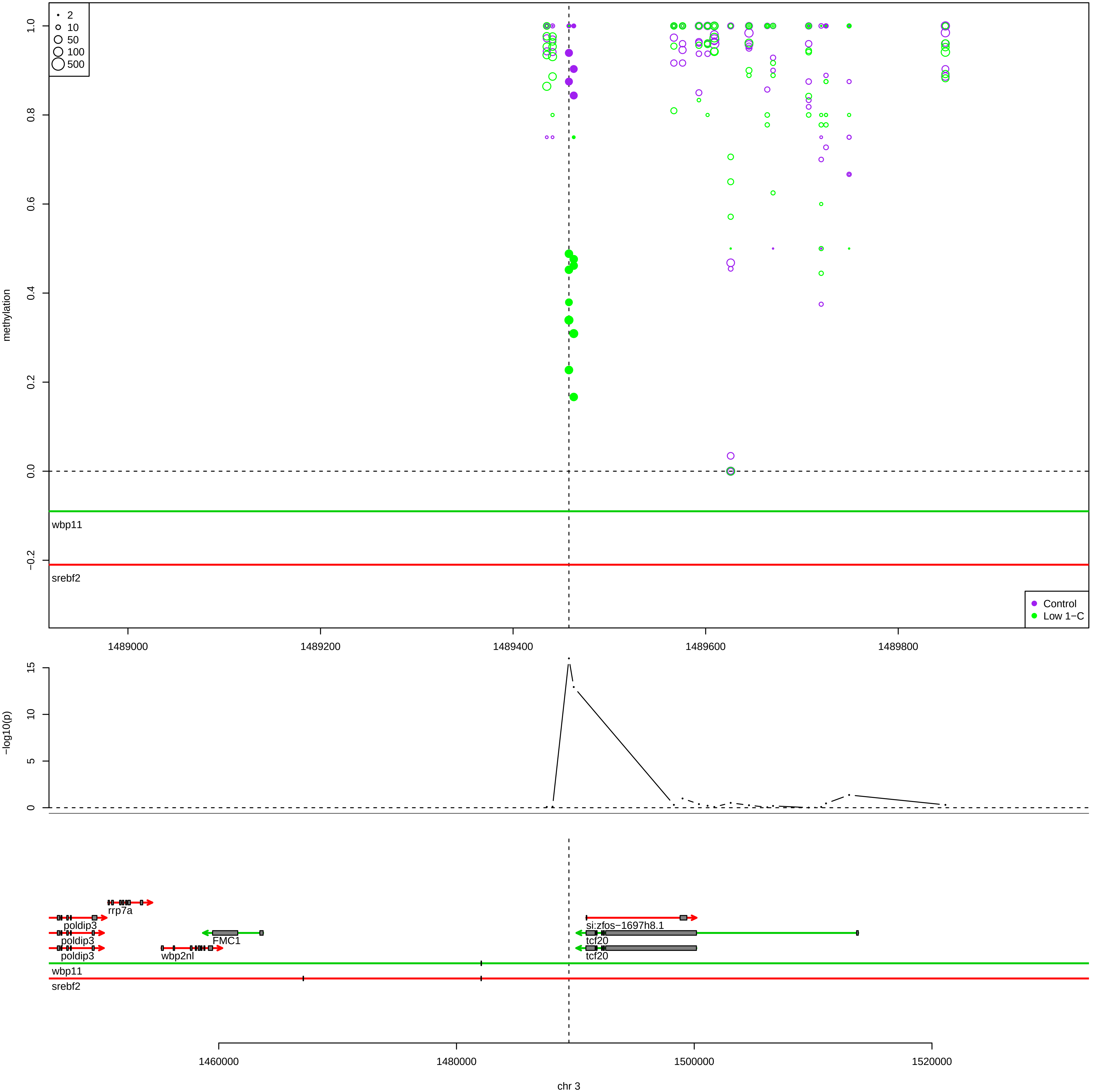

2
10
50
100
500
methylation
1.0
0.8
0.6
0.4
0.2
0.0
−0.2
wbp11
srebf2
Control
Low 1−C
1489000
1489200
1489400
1489600
1489800
−log10(p)
15
10
5
0
rrp7a
poldip3
poldip3
poldip3
FMC1
wbp2nl
si:zfos−1697h8.1
tcf20
tcf20
wbp11
srebf2
1460000
1480000
1500000
1520000
chr 3

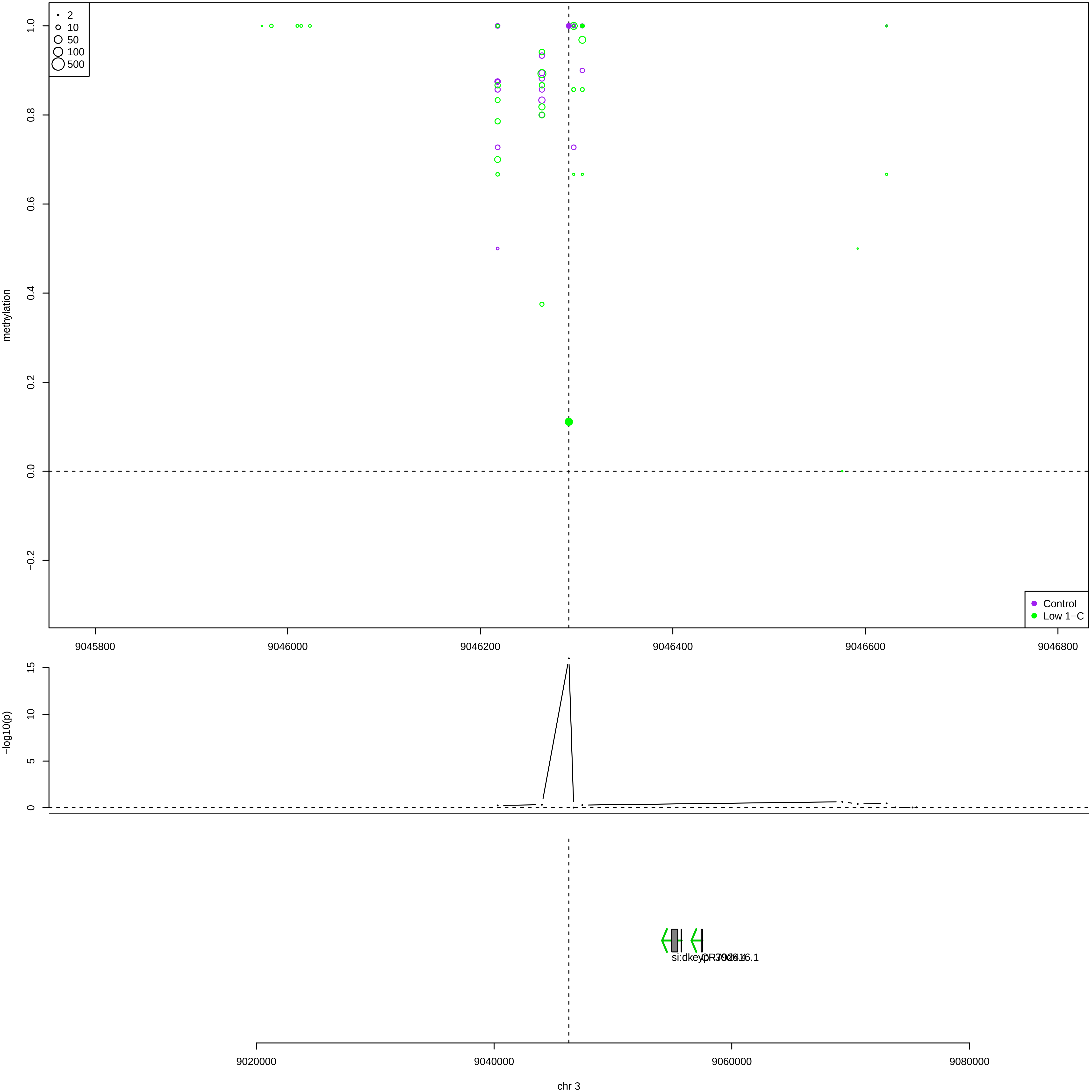

2
10
50
100
500
methylation
1.0
0.8
0.6
0.4
0.2
0.0
−0.2
Control
Low 1−C
9045800
9046000
9046200
9046400
9046600
9046800
−log10(p)
15
10
5
0
9020000
9040000
9060000
9080000
chr 3

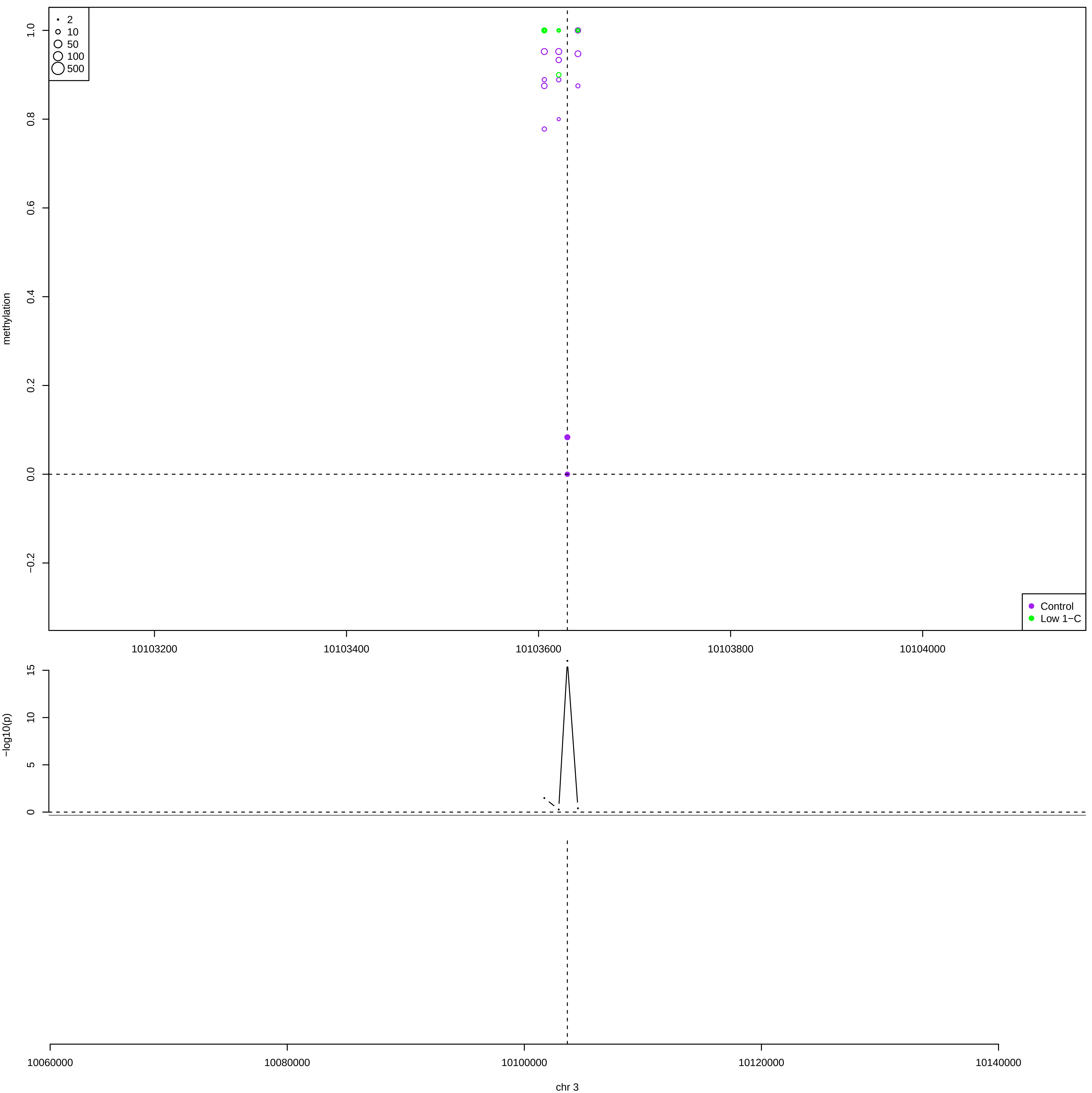

2
10
50
100
500
methylation
1.0
0.8
0.6
0.4
0.2
0.0
−0.2
Control
Low 1−C
10103200
10103400
10103600
10103800
10104000
−log10(p)
15
10
5
0
10060000
10080000
10100000
10120000
10140000
chr 3

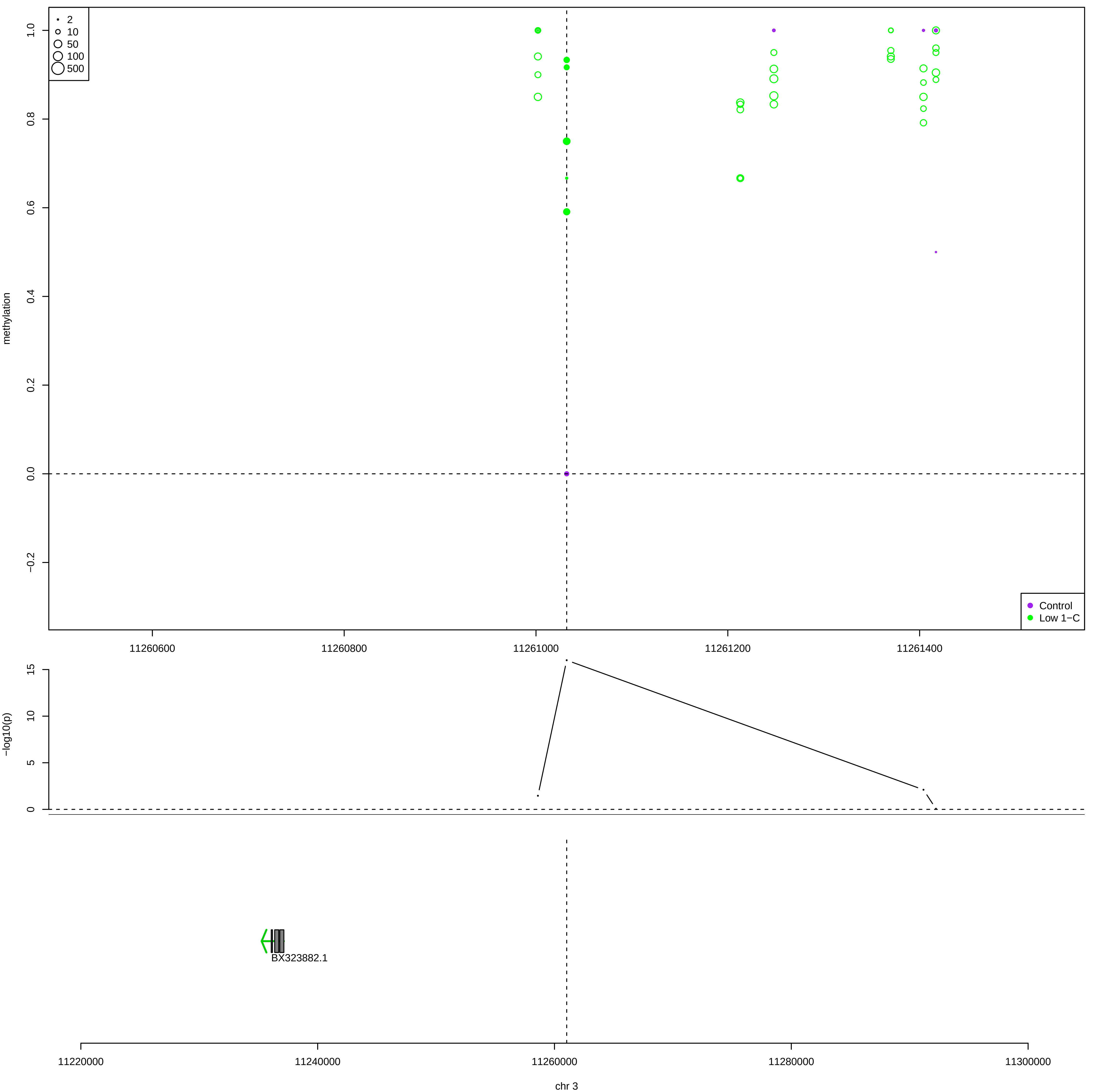

2
10
50
100
500
methylation
1.0
0.8
0.6
0.4
0.2
0.0
−0.2
Control
Low 1−C
11260600
11260800
11261000
11261200
11261400
−log10(p)
15
10
5
0
BX323882.1
11220000
11240000
11260000
11280000
11300000
chr 3

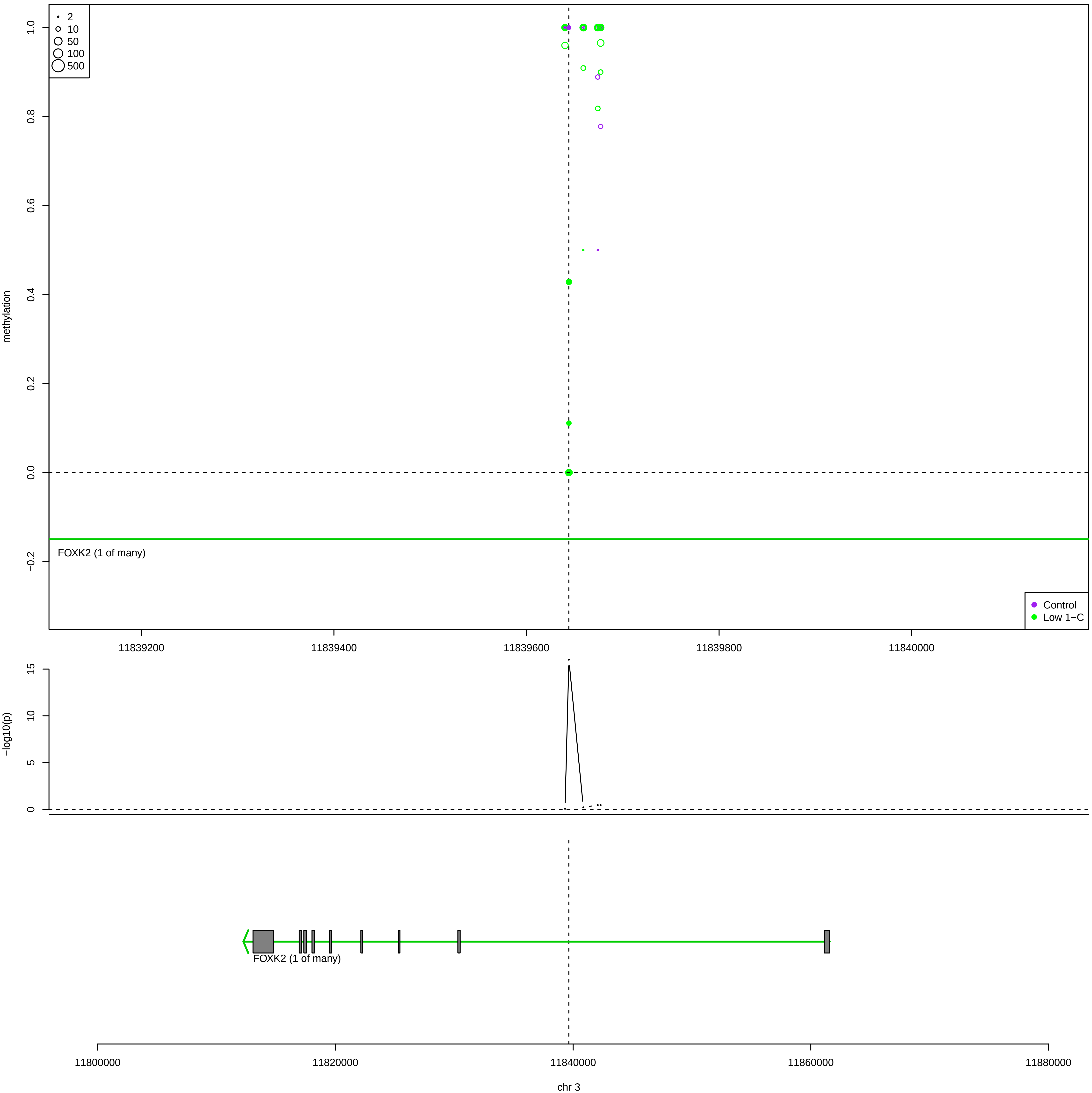

2
10
50
100
500
methylation
1.0
0.8
0.6
0.4
0.2
0.0
−0.2
FOXK2 (1 of many)
Control
Low 1−C
11839200
11839400
11839600
11839800
11840000
−log10(p)
15
10
5
0
FOXK2 (1 of many)
11800000
11820000
11840000
11860000
11880000
chr 3

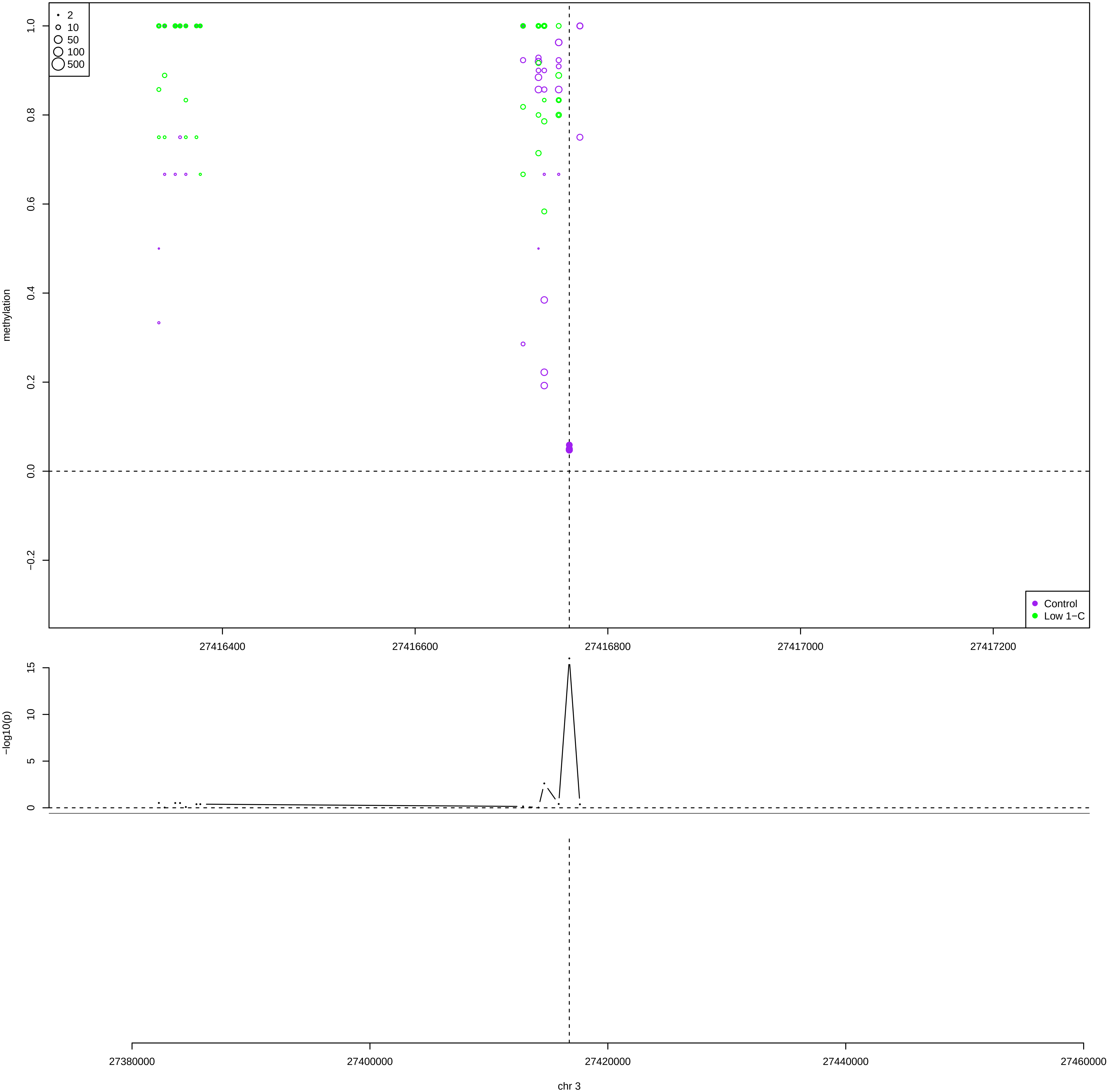

2
10
50
100
500
Control
Low 1−C
methylation
1.0
0.8
0.6
0.4
0.2
0.0
−0.2
27416400
27416600
27416800
27417000
27417200
−log10(p)
15
10
5
0
27380000
27400000
27420000
27440000
27460000
chr 3

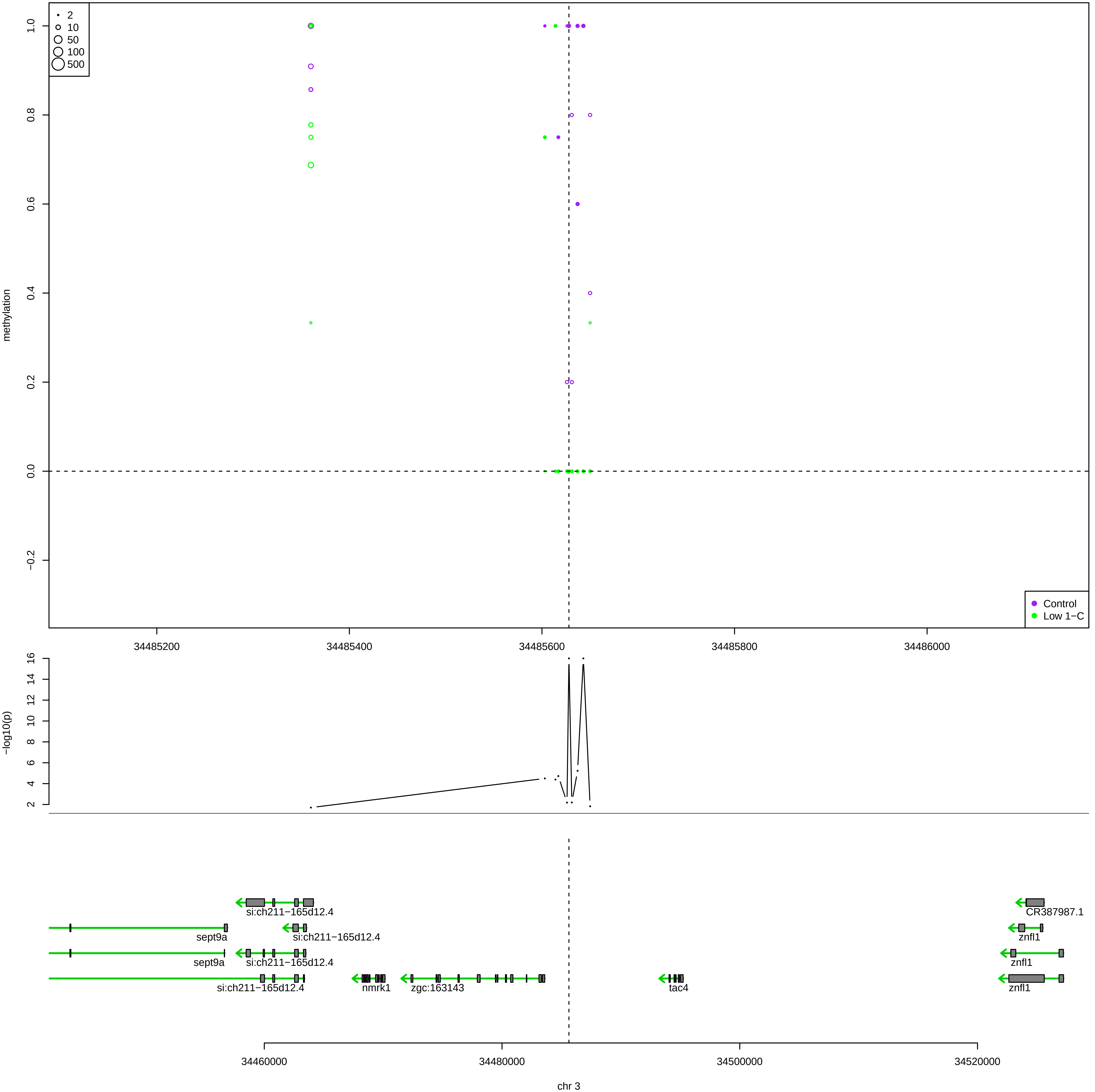

2
10
50
100
500
methylation
1.0
0.8
0.6
0.4
0.2
0.0
−0.2
Control
Low 1−C
34485200
34485400
34485600
34485800
34486000
−log10(p)
16
14
12
10
8
6
4
2
si:ch211−165d12.4
sept9a
si:ch211−165d12.4
sept9a
si:ch211−165d12.4
si:ch211−165d12.4
nmrk1
zgc:163143
tac4
CR387987.1
znfl1
znfl1
znfl1
34460000
34480000
34500000
34520000
chr 3

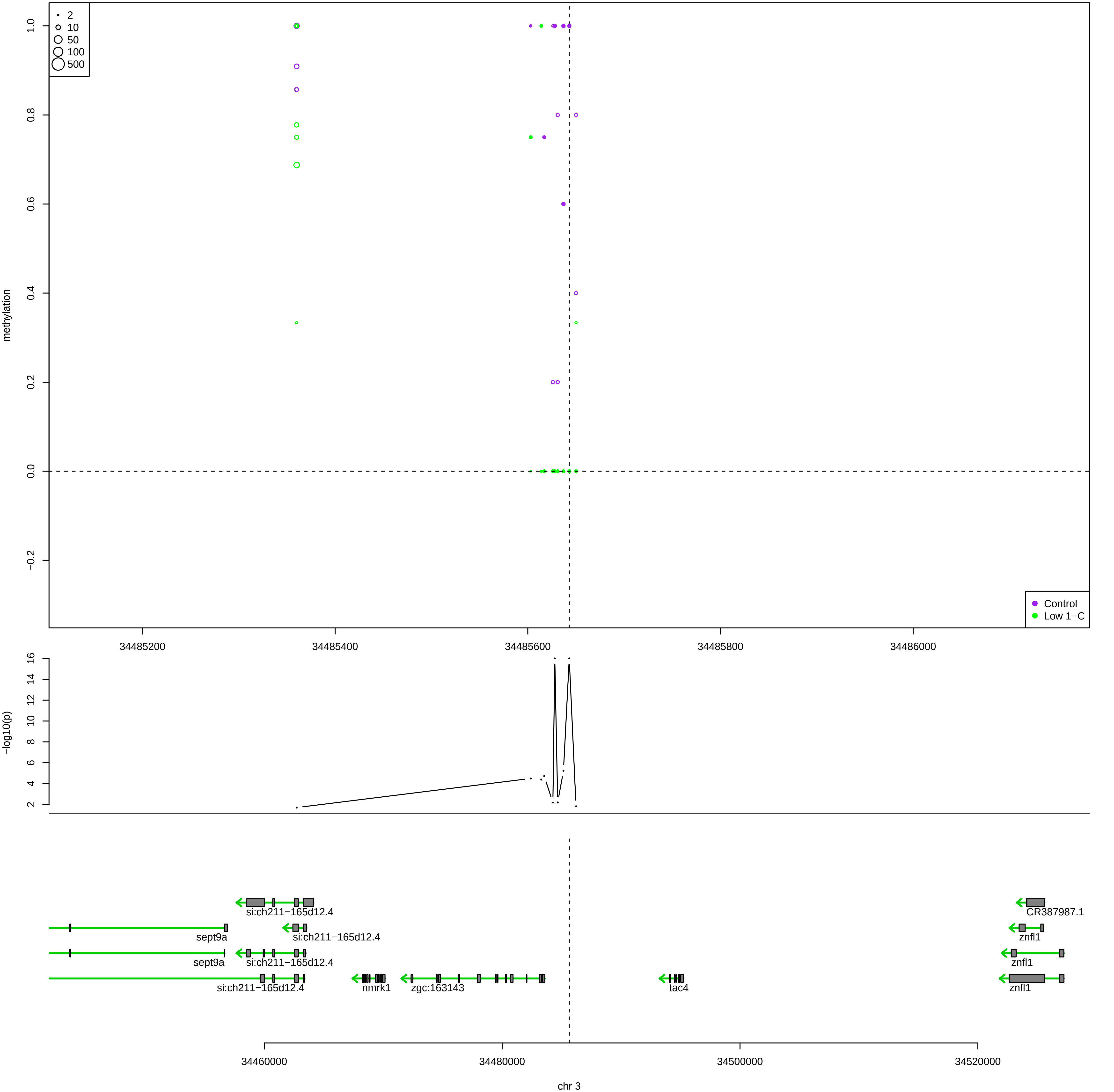

2
10
50
100
500
methylation
1.0
0.8
0.6
0.4
0.2
0.0
−0.2
Control
Low 1−C
34485200
34485400
34485600
34485800
34486000
−log10(p)
16
14
12
10
8
6
4
2
si:ch211−165d12.4
sept9a
si:ch211−165d12.4
sept9a
si:ch211−165d12.4
si:ch211−165d12.4
nmrk1
zgc:163143
tac4
CR387987.1
znfl1
znfl1
znfl1
34460000
34480000
34500000
34520000
chr 3

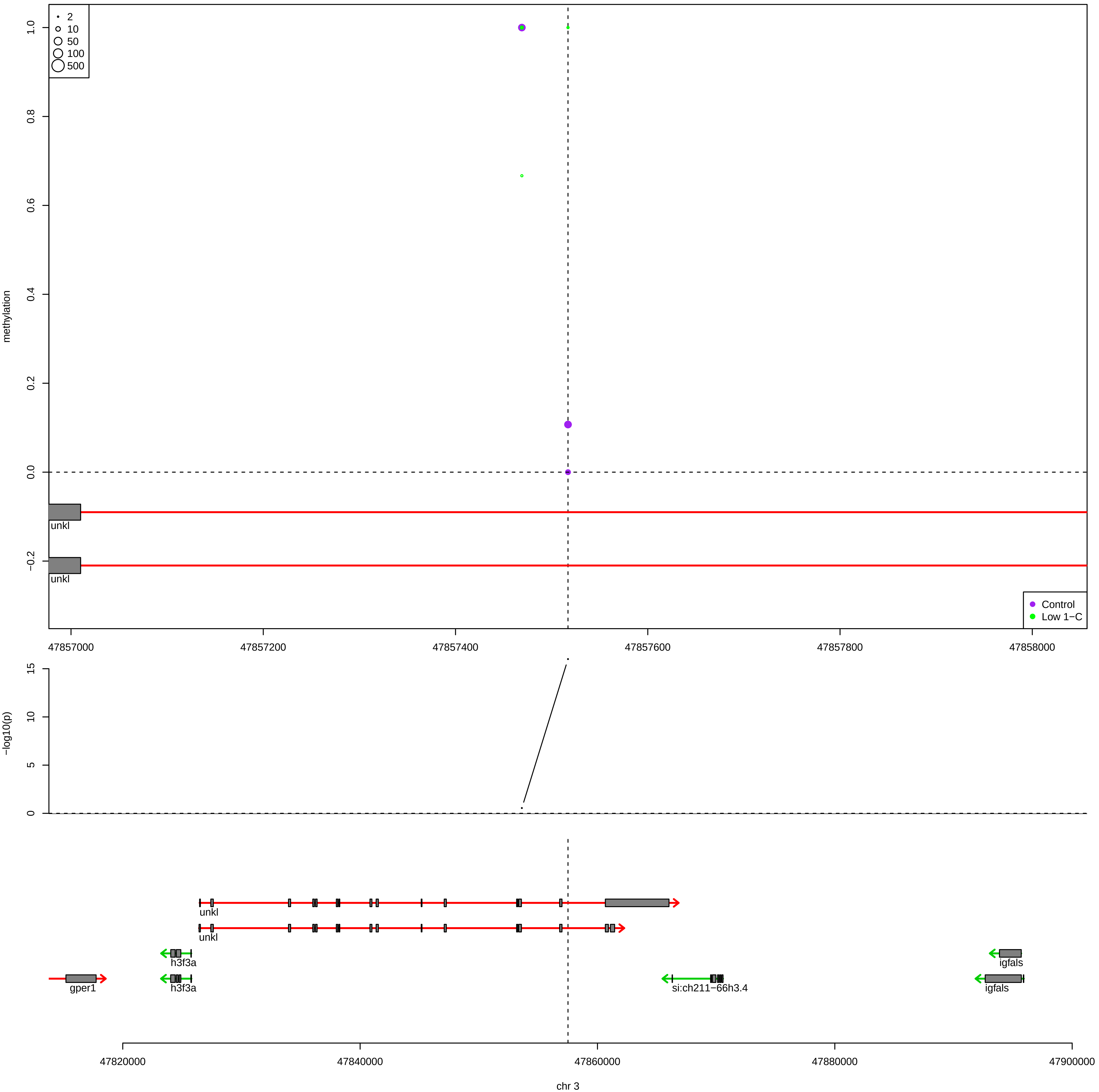

2
10
50
100
500
methylation
1.0
0.8
0.6
0.4
0.2
0.0
−0.2
unkl
unkl
Control
Low 1−C
47857000
47857200
47857400
47857600
47857800
47858000
−log10(p)
15
10
5
0
unkl
unkl
h3f3a
h3f3a
gper1
si:ch211−66h3.4
igfals
igfals
47820000
47840000
47860000
47880000
47900000
chr 3

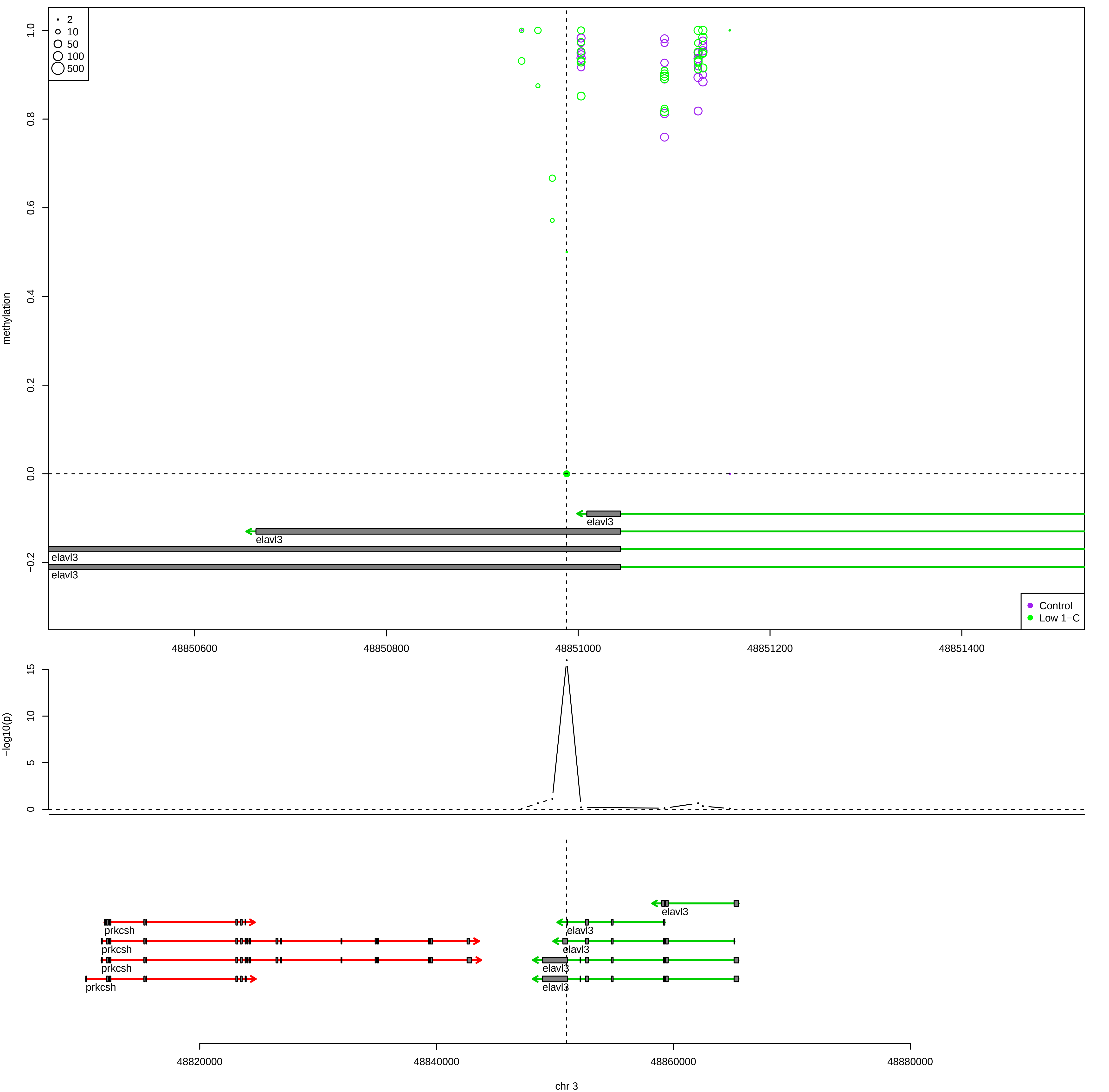

methylation
2
10
50
100
500
Control
Low 1−C
elavl3
elavl3
elavl3
elavl3
48850600
48850800
48851000
48851200
48851400
−log10(p)
prkcsh
prkcsh
prkcsh
prkcsh
elavl3
elavl3
elavl3
elavl3
elavl3
48820000
48840000
48860000
48880000
chr 3

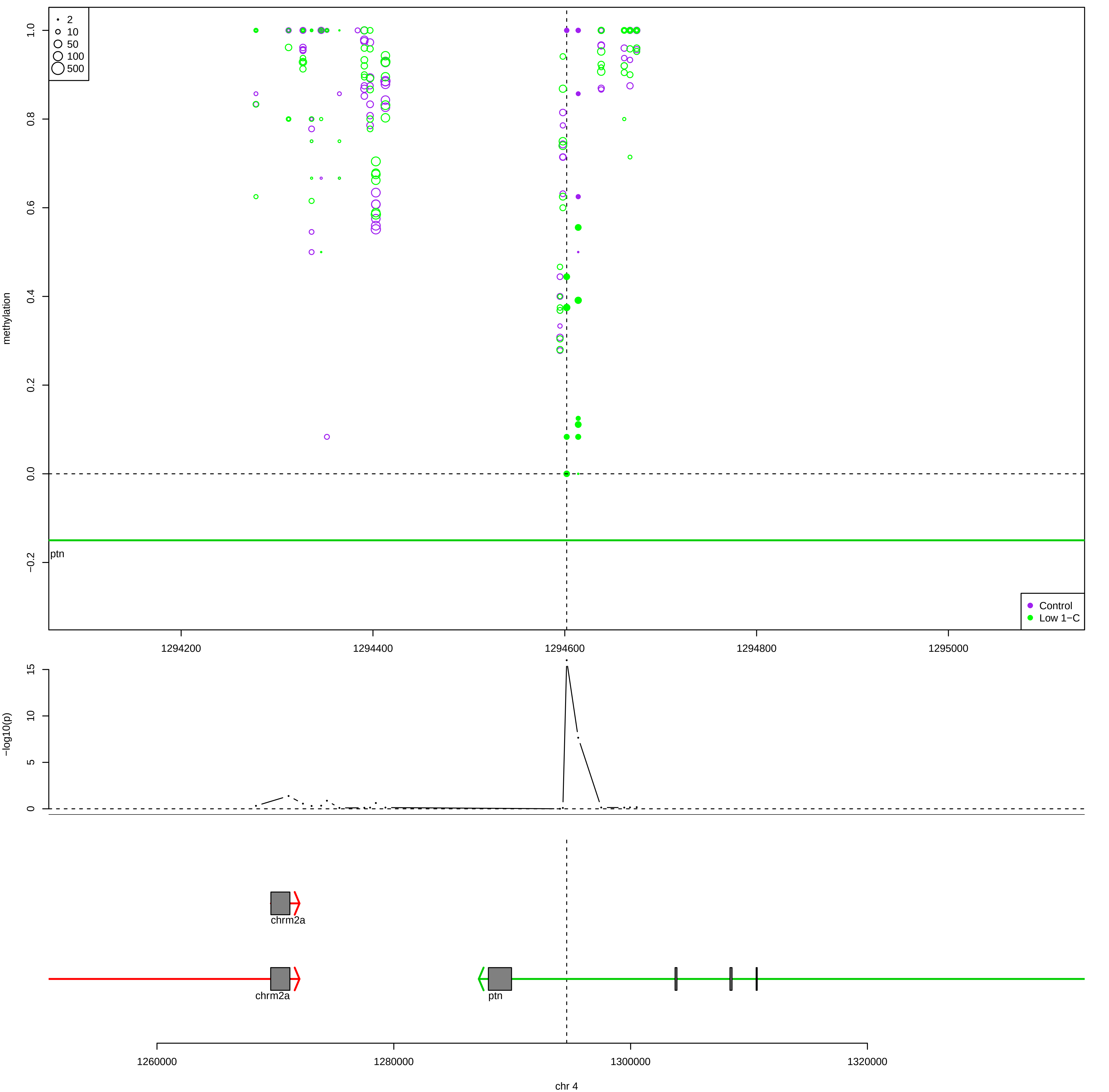

2
10
50
100
500
methylation
1.0
0.8
0.6
0.4
0.2
0.0
−0.2
ptn
Control
Low 1−C
1294200
1294400
1294600
1294800
1295000
−log10(p)
15
10
5
0
chrm2a
chrm2a
ptn
1260000
1280000
1300000
1320000
chr 4

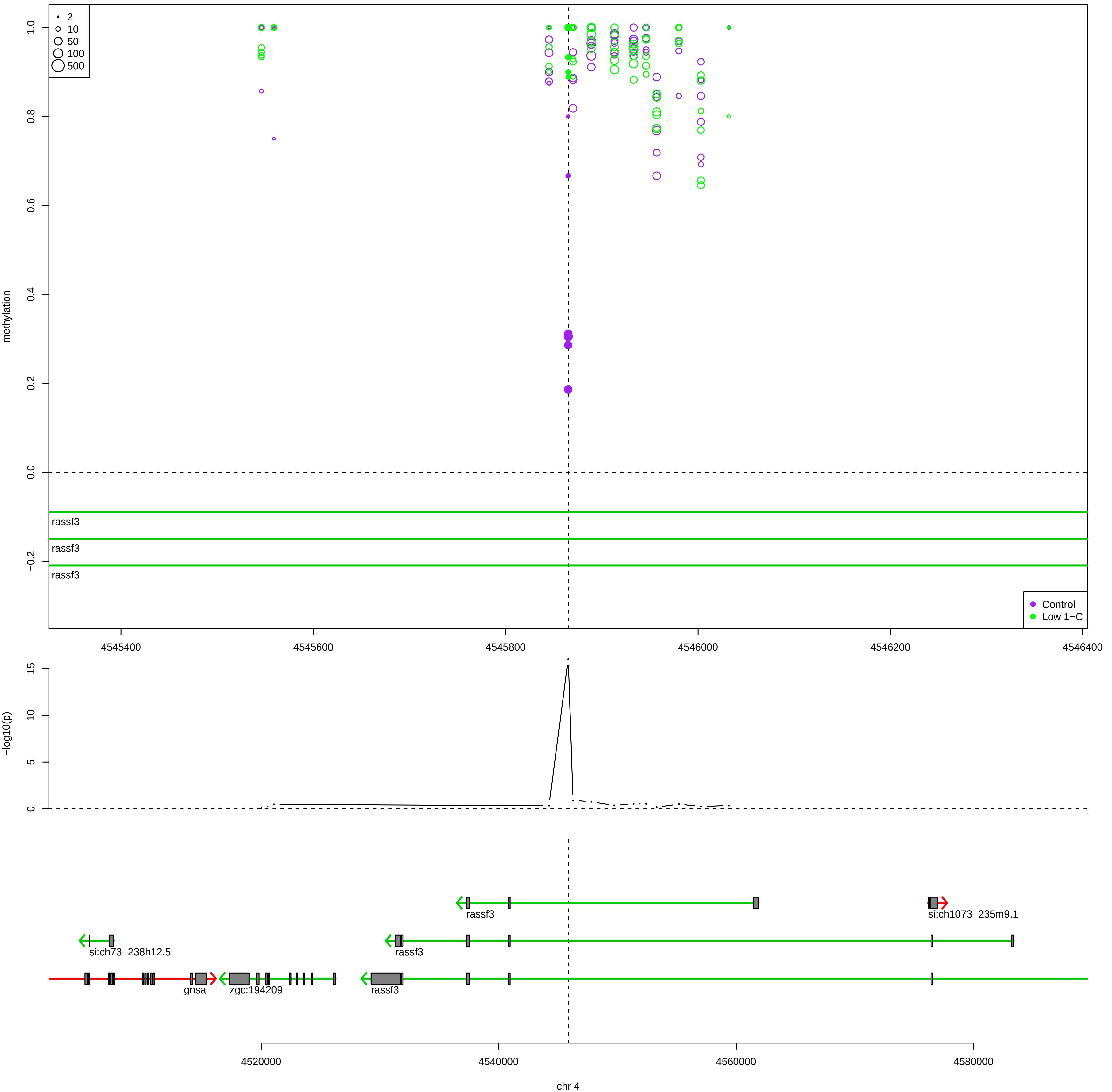

2
10
50
100
500
methylation
1.0
0.8
0.6
0.4
0.2
0.0
−0.2
rassf3
rassf3
rassf3
Control
Low 1−C
4545400
4545600
4545800
4546000
4546200
4546400
−log10(p)
15
10
5
0
rassf3
si:ch1073−235m9.1
si:ch73−238h12.5
rassf3
gnsa
zgc:194209
rassf3
4520000
4540000
4560000
4580000
chr 4

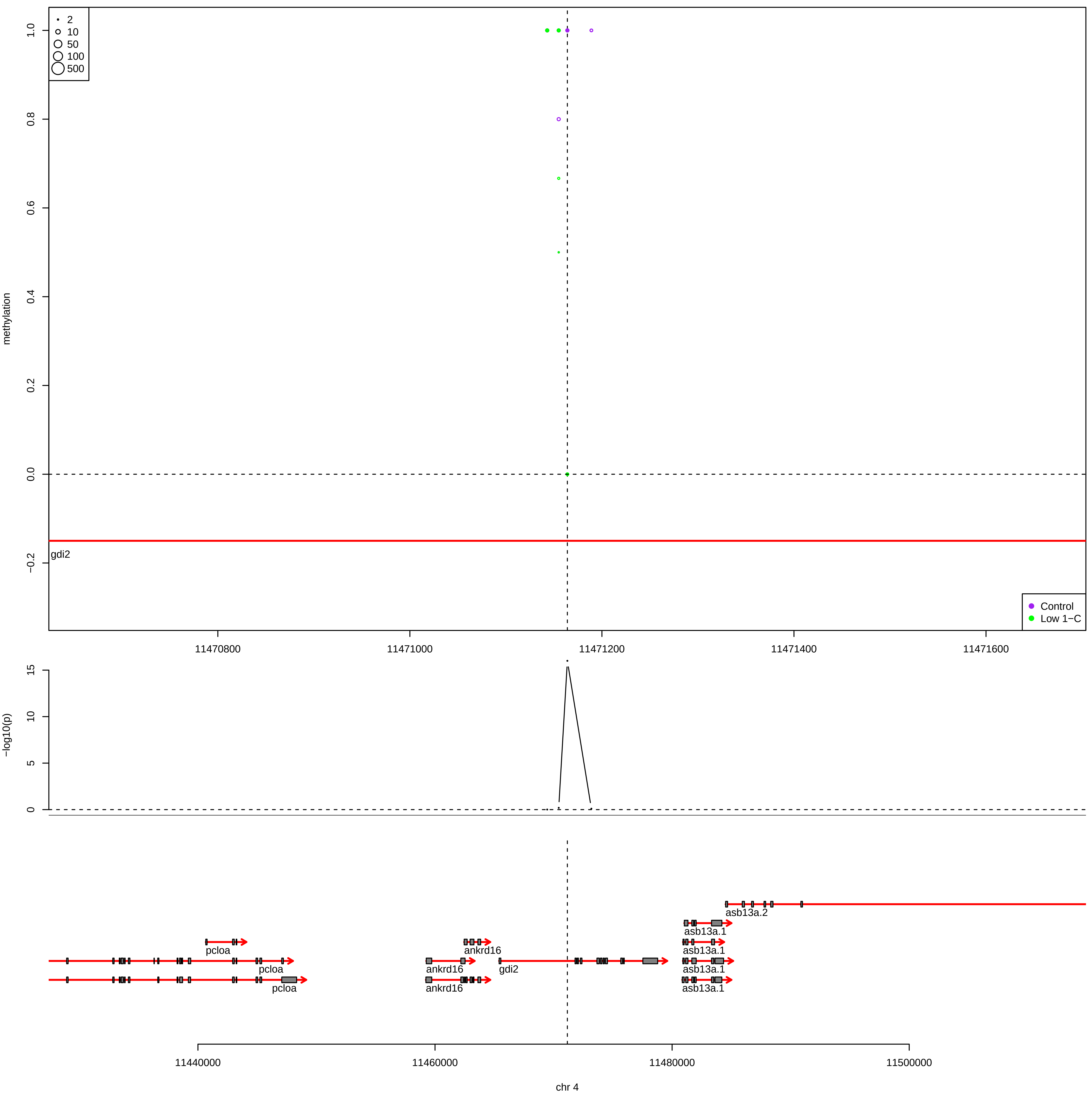

2
10
50
100
500
methylation
1.0
0.8
0.6
0.4
0.2
0.0
−0.2
gdi2
Control
Low 1−C
11470800
11471000
11471200
11471400
11471600
−log10(p)
15
10
5
0
asb13a.2
asb13a.1
pcloa
ankrd16
gdi2
11440000
11460000
11480000
11500000
chr 4

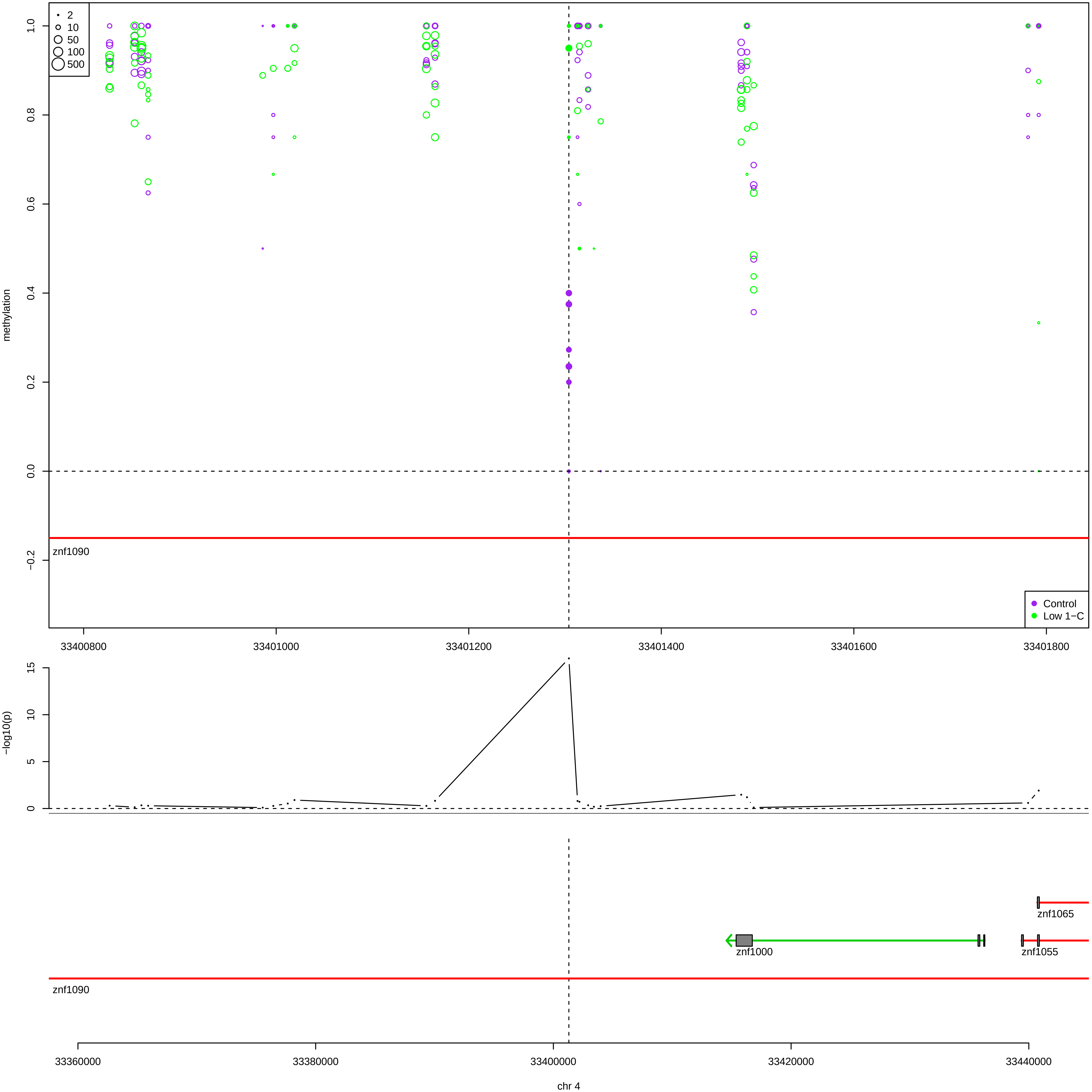

methylation
2
10
50
100
500
1.0
0.8
0.6
0.4
0.2
0.0
−0.2
znf1090
Control
Low 1−C
33400800
33401000
33401200
33401400
33401600
33401800
−log10(p)
15
10
5
0
znf1065
znf1000
znf1055
znf1090
33360000
33380000
33400000
33420000
33440000
chr 4

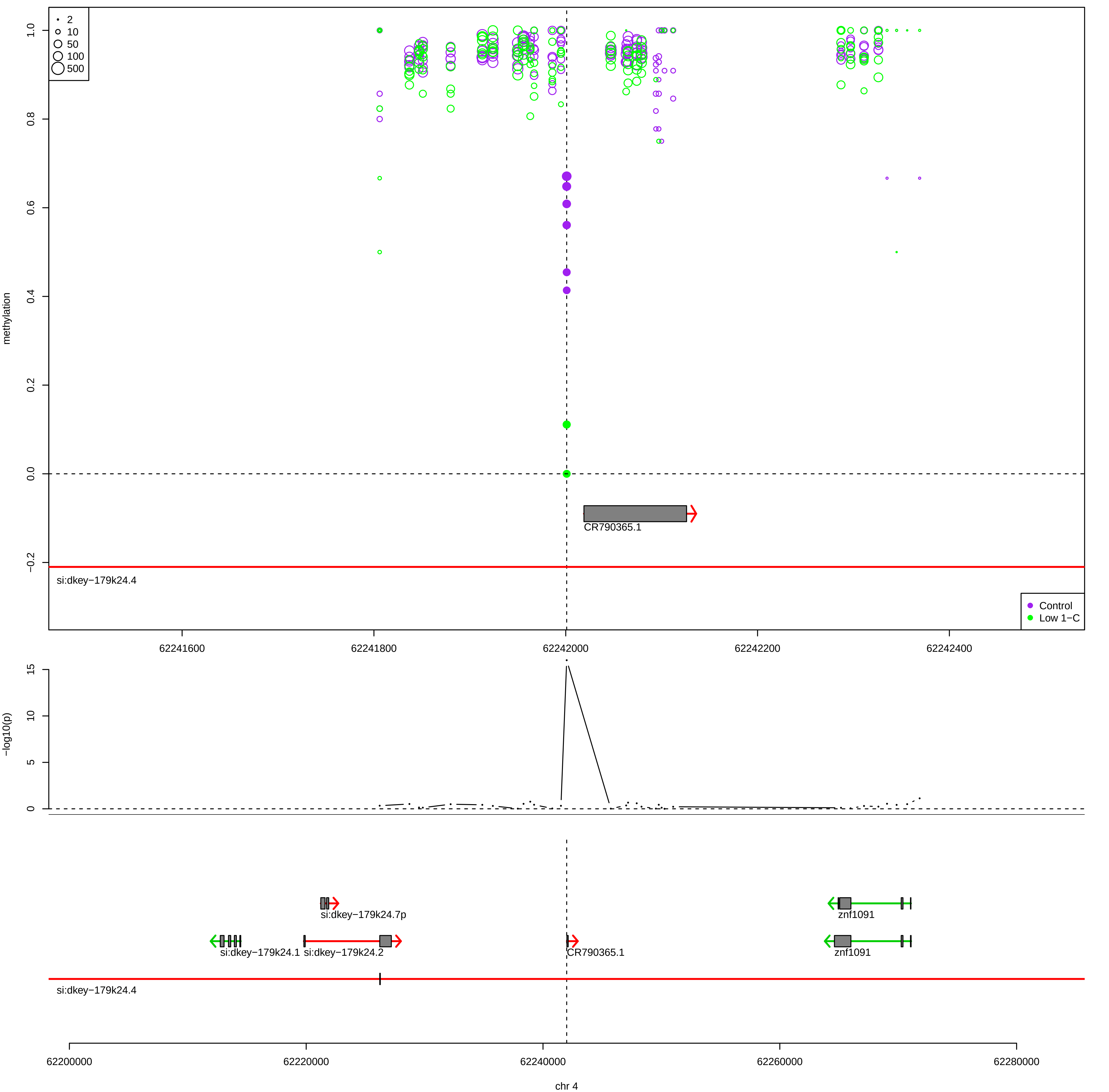

2
10
50
100
500
methylation
1.0
0.8
0.6
0.4
0.2
0.0
−0.2
CR790365.1
si:dkey−179k24.4
Control
Low 1−C
62241600
62241800
62242000
62242200
62242400
−log10(p)
15
10
5
0
si:dkey−179k24.7p
znf1091
si:dkey−179k24.1
si:dkey−179k24.2
CR790365.1
znf1091
si:dkey−179k24.4
62200000
62220000
62240000
62260000
62280000
chr 4

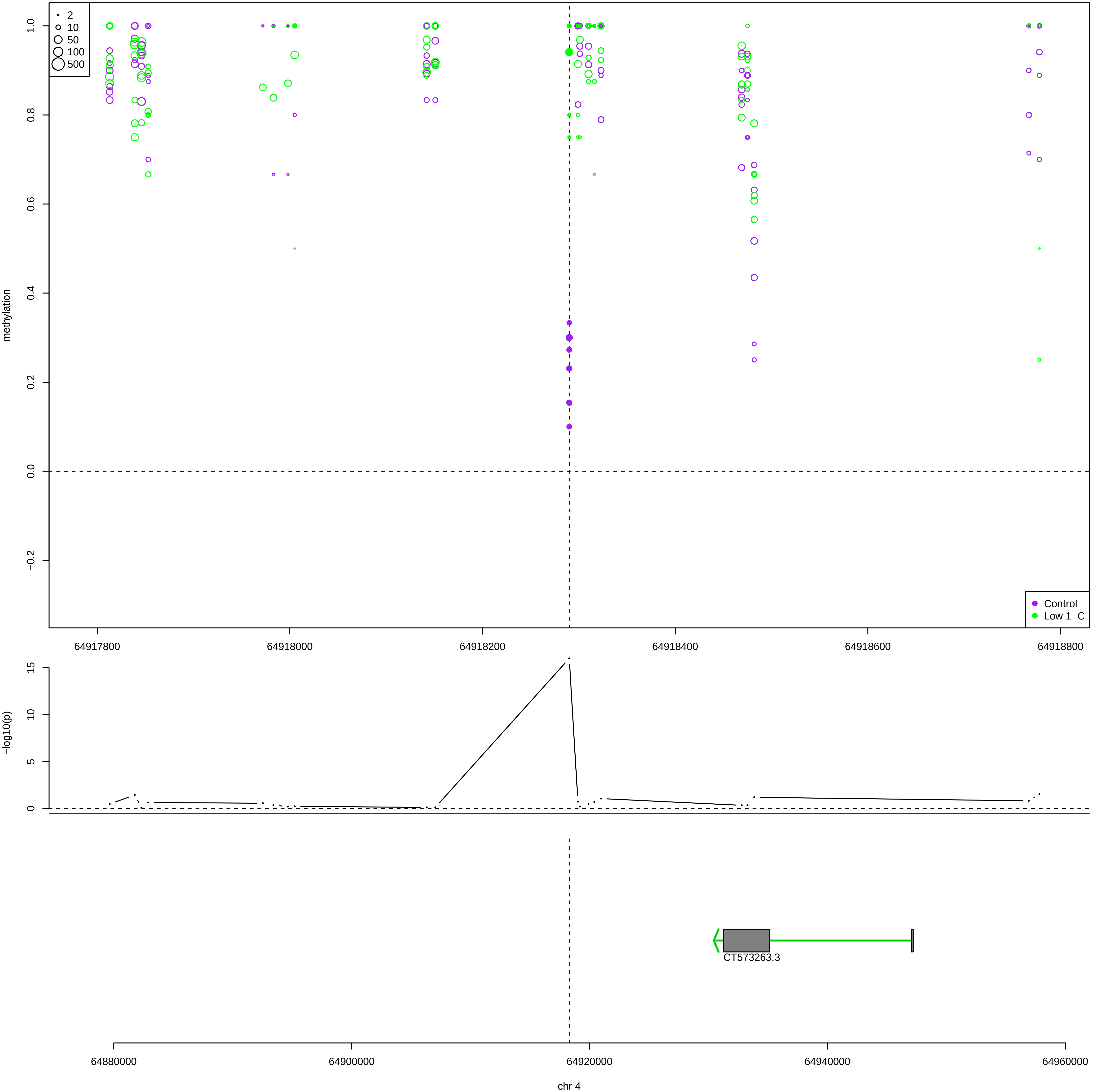

2
10
50
100
500
methylation
1.0
0.8
0.6
0.4
0.2
0.0
−0.2
Control
Low 1−C
64917800
64918000
64918200
64918400
64918600
64918800
−log10(p)
15
10
5
0
CT573263.3
64880000
64900000
64920000
64940000
64960000
chr 4

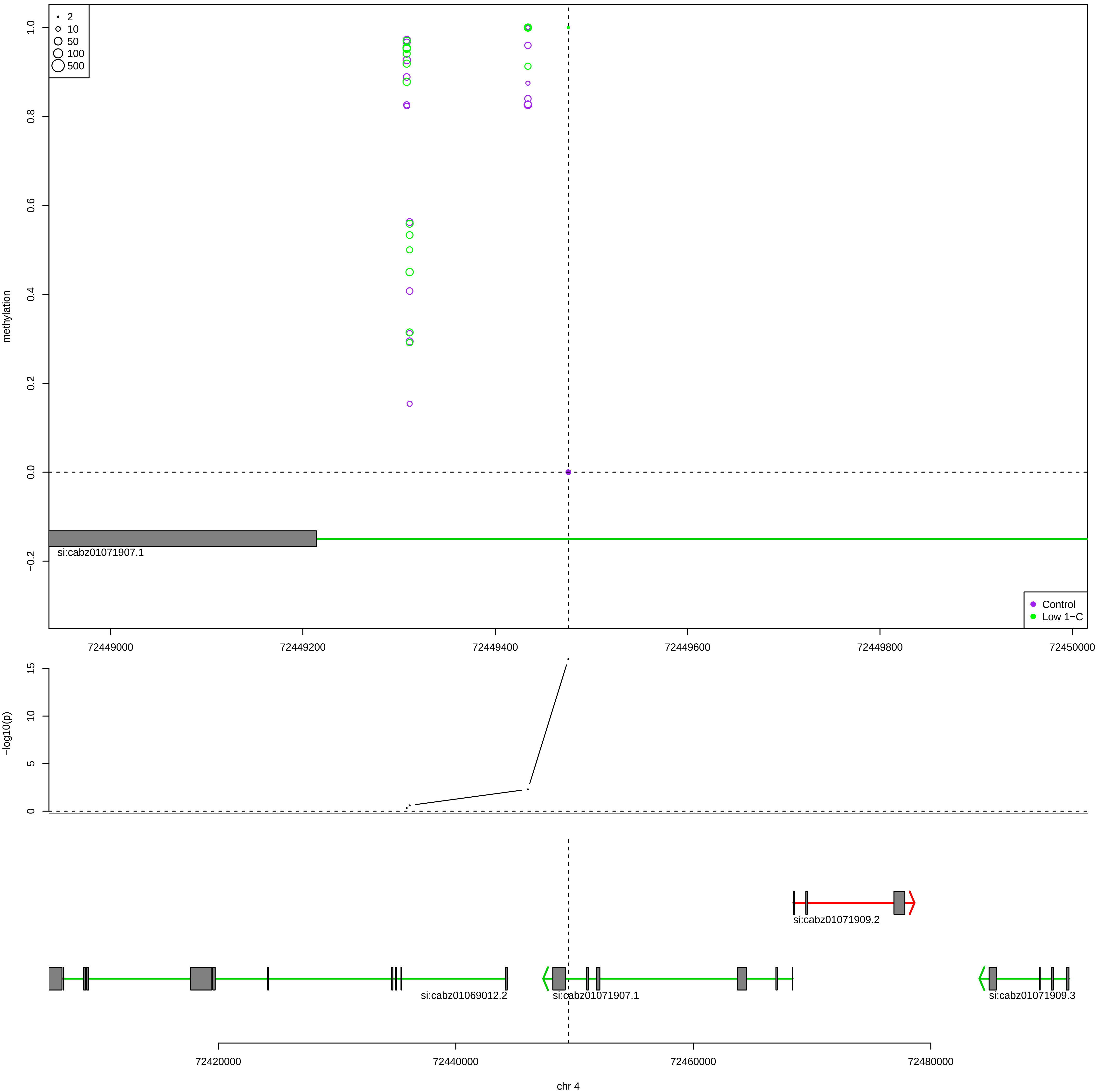

2
10
50
100
500
methylation
1.0
0.8
0.6
0.4
0.2
0.0
−0.2
si:cabz01071907.1
Control
Low 1−C
72449000
72449200
72449400
72449600
72449800
72450000
−log10(p)
15
10
5
0
si:cabz01071909.2
si:cabz01069012.2
si:cabz01071907.1
si:cabz01071909.3
72420000
72440000
72460000
72480000
chr 4

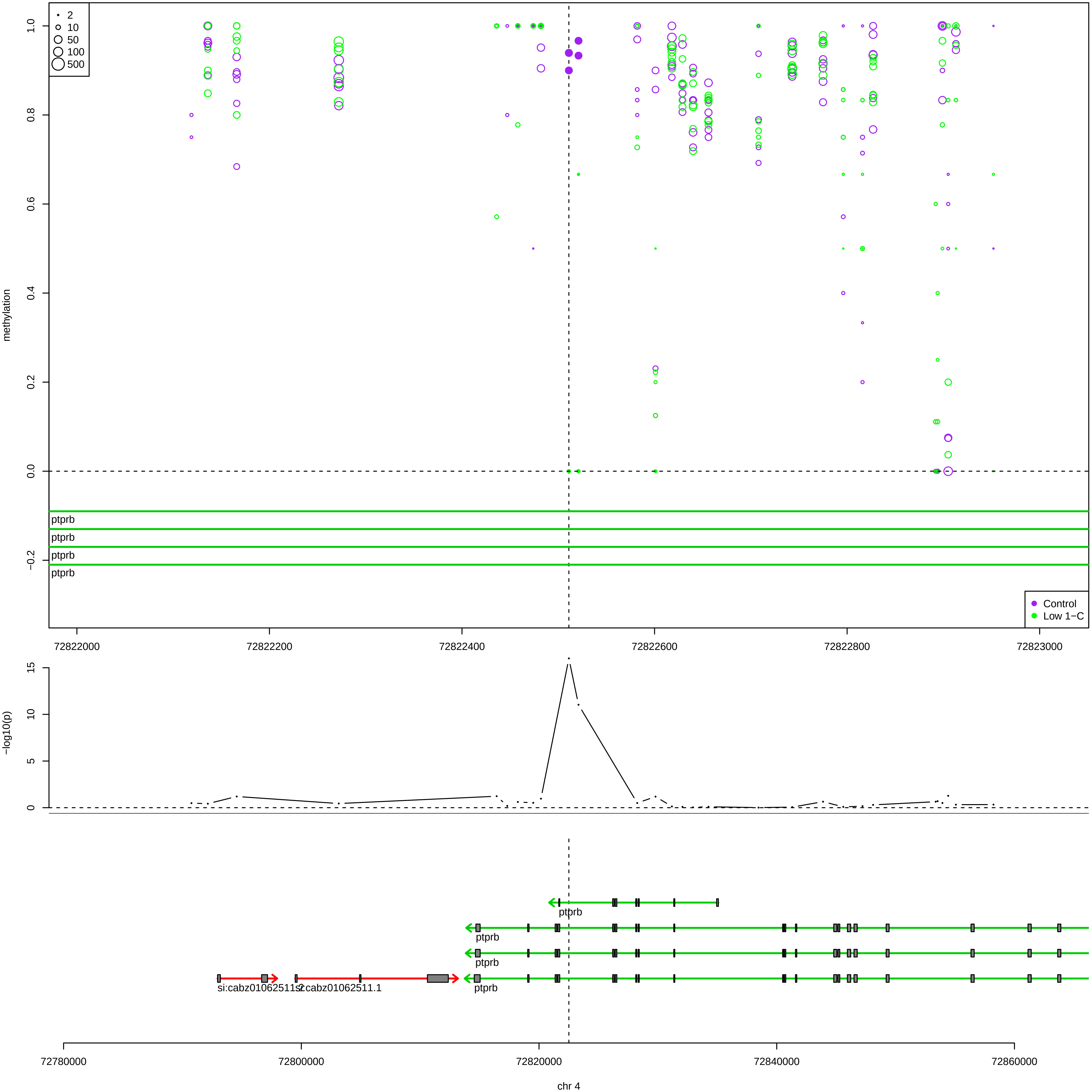

2
10
50
100
500
methylation
1.0
0.8
0.6
0.4
0.2
0.0
−0.2
ptprb
ptprb
ptprb
ptprb
Control
Low 1−C
72822000
72822200
72822400
72822600
72822800
72823000
−log10(p)
15
10
5
0
ptprb
ptprb
ptprb
ptprb
si:cabz01062511.2
si:cabz01062511.1
72780000
72800000
72820000
72840000
72860000
chr 4

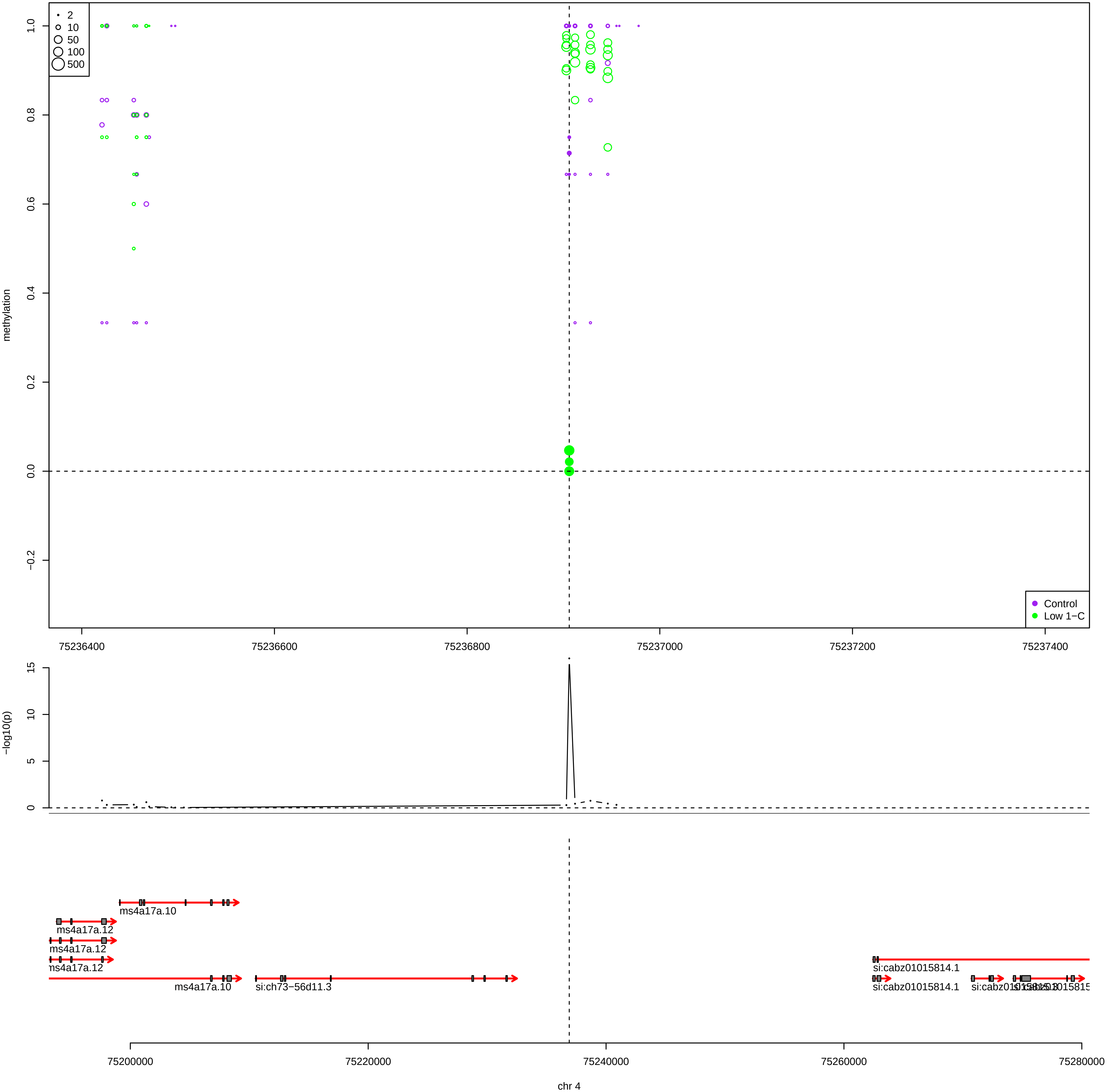

methylation
1.0
0.8
0.6
0.4
0.2
0.0
−0.2
2
10
50
100
500
Control
Low 1−C
75236400
75236600
75236800
75237000
75237200
75237400
−log10(p)
15
10
5
0
ms4a17a.10
ms4a17a.12
ms4a17a.12
ms4a17a.12
ms4a17a.10
si:ch73−56d11.3
si:cabz01015814.1
si:cabz01015814.1
75200000
75220000
75240000
75260000
75280000
chr 4

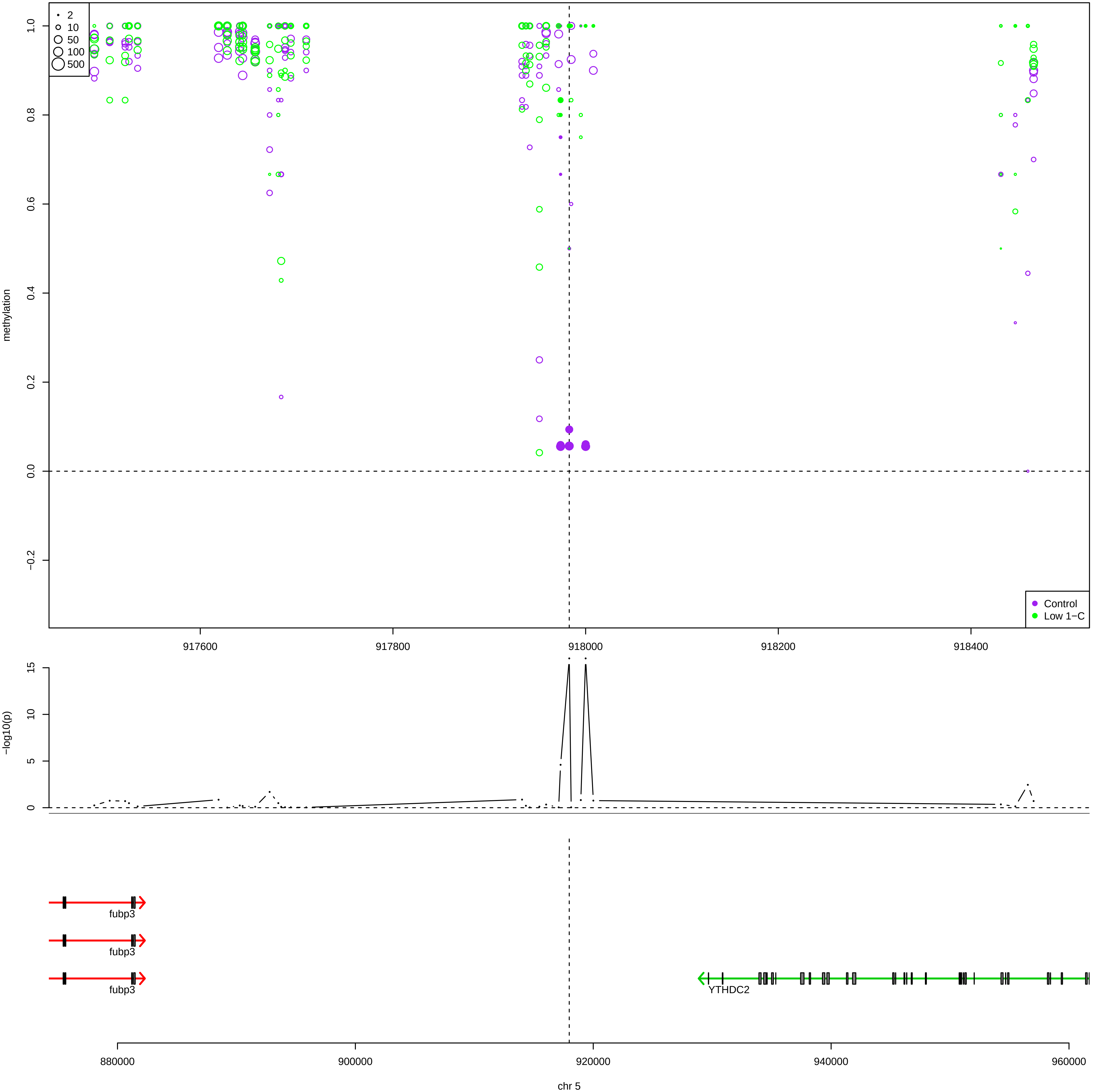
2
10
50
100
500
methylation
1.0
0.8
0.6
0.4
0.2
0.0
-0.2
Control
Low 1-C
917600
917800
918000
918200
918400
-log10(p)
15
10
5
0
fubp3
fubp3
fubp3
YTHDC2
880000
900000
920000
940000
960000
chr 5

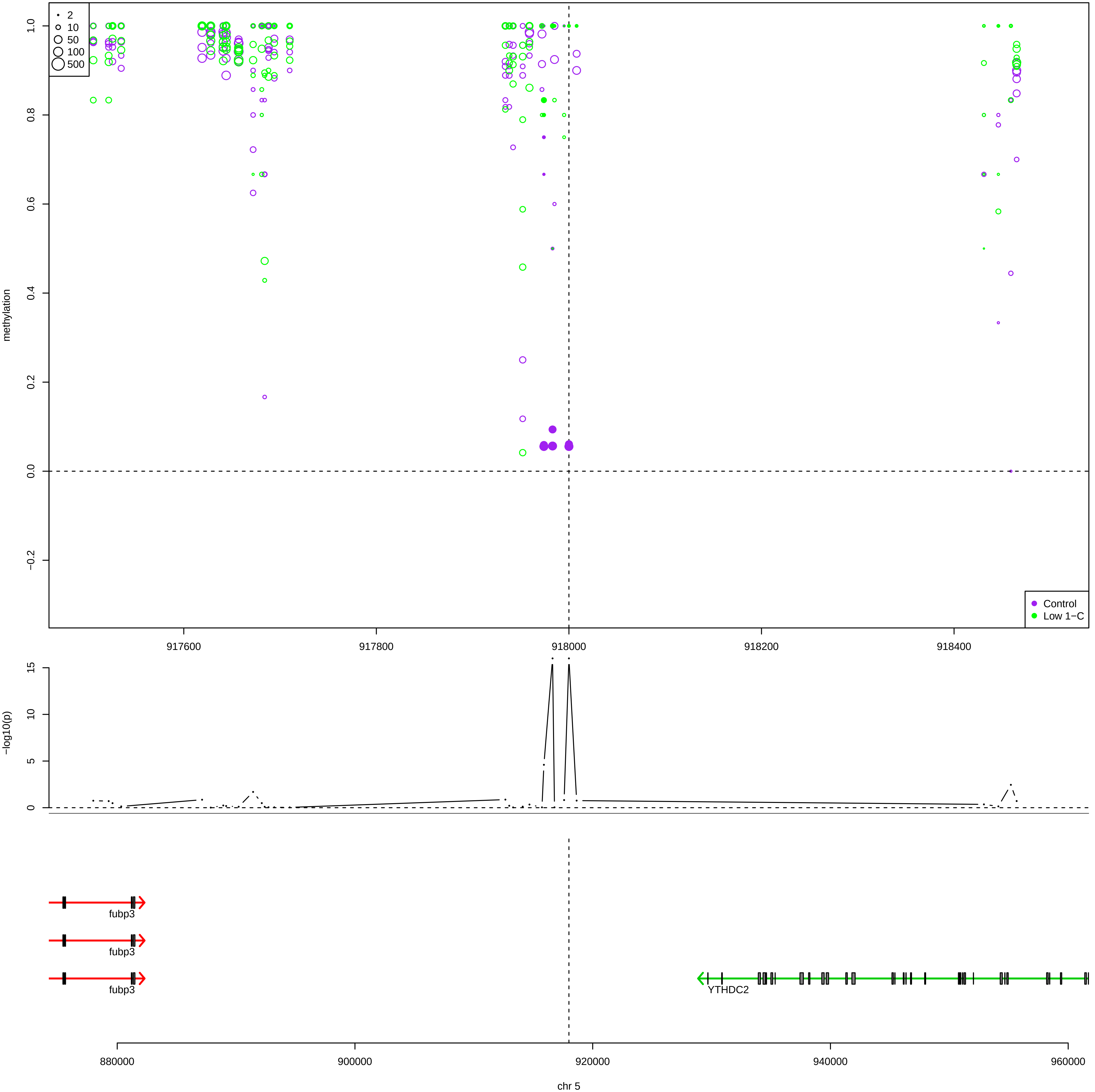

2
10
50
100
500
methylation
1.0
0.8
0.6
0.4
0.2
0.0
−0.2
Control
Low 1−C
917600
917800
918000
918200
918400
−log10(p)
15
10
5
0
fubp3
fubp3
fubp3
YTHDC2
880000
900000
920000
940000
960000
chr 5

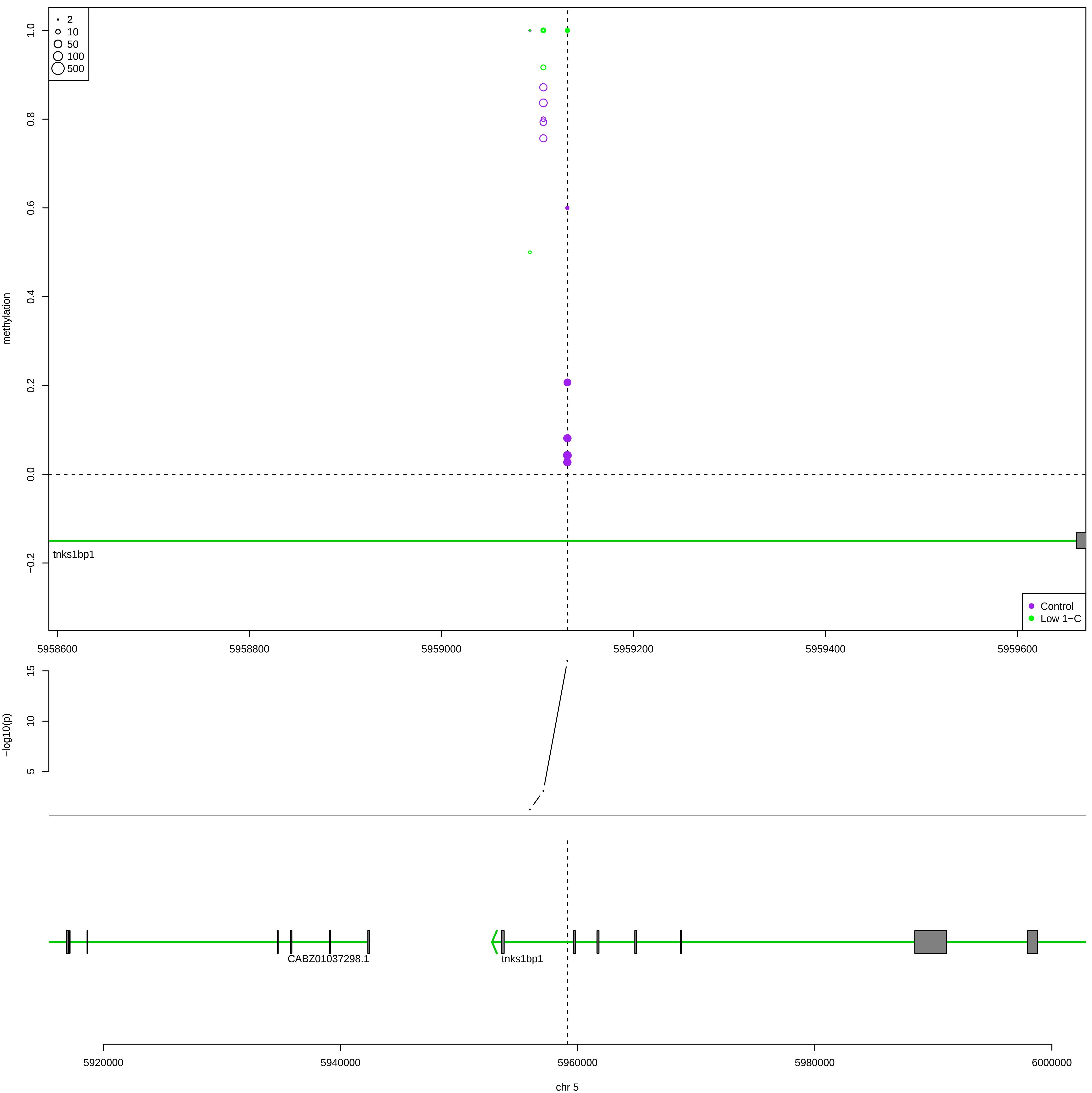

2
10
50
100
500
methylation
1.0
0.8
0.6
0.4
0.2
0.0
−0.2
tnks1bp1
Control
Low 1−C
5958600
5958800
5959000
5959200
5959400
5959600
−log10(p)
15
10
5
CABZ01037298.1
tnks1bp1
5920000
5940000
5960000
5980000
6000000
chr 5

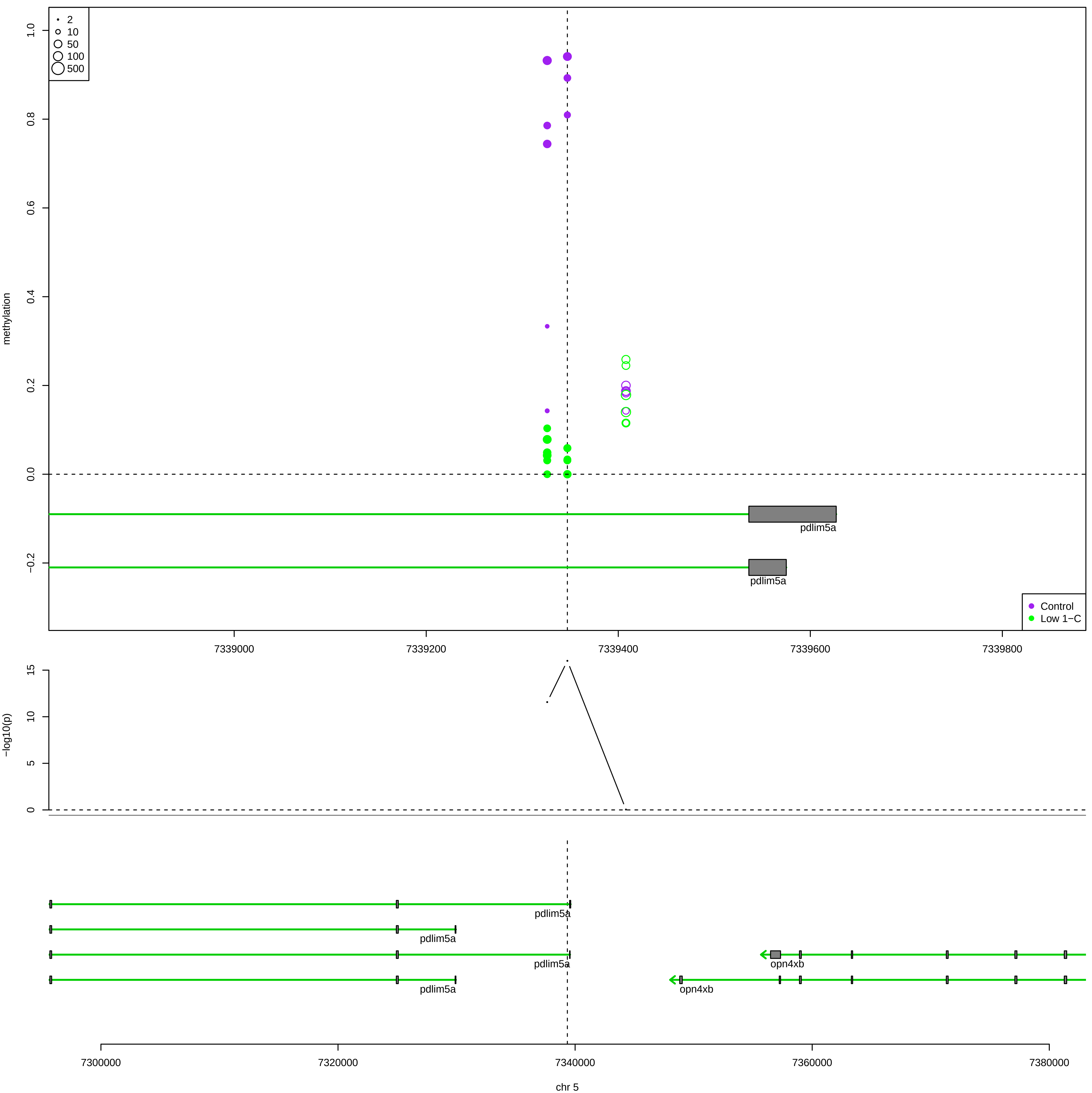

methylation
1.0
0.8
0.6
0.4
0.2
0.0
−0.2
2
10
50
100
500
Control
Low 1−C
pdlim5a
pdlim5a
7339000
7339200
7339400
7339600
7339800
−log10(p)
15
10
5
0
pdlim5a
pdlim5a
pdlim5a
pdlim5a
opn4xb
opn4xb
7300000
7320000
7340000
7360000
7380000
chr 5

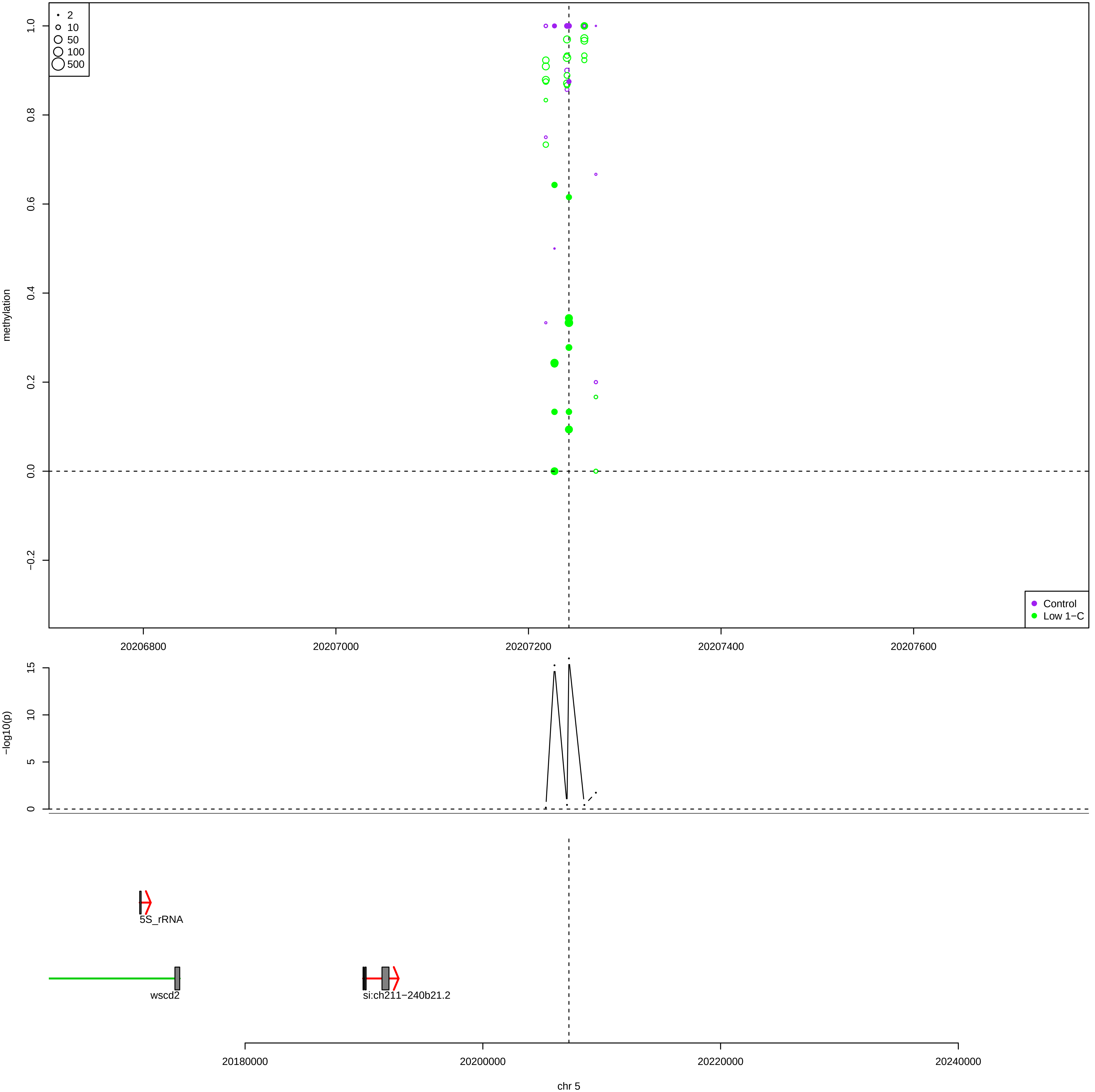

2
10
50
100
500
methylation
1.0
0.8
0.6
0.4
0.2
0.0
−0.2
Control
Low 1−C
20206800
20207000
20207200
20207400
20207600
−log10(p)
15
10
5
0
5S_rRNA
wscd2
si:ch211−240b21.2
20180000
20200000
20220000
20240000
chr 5

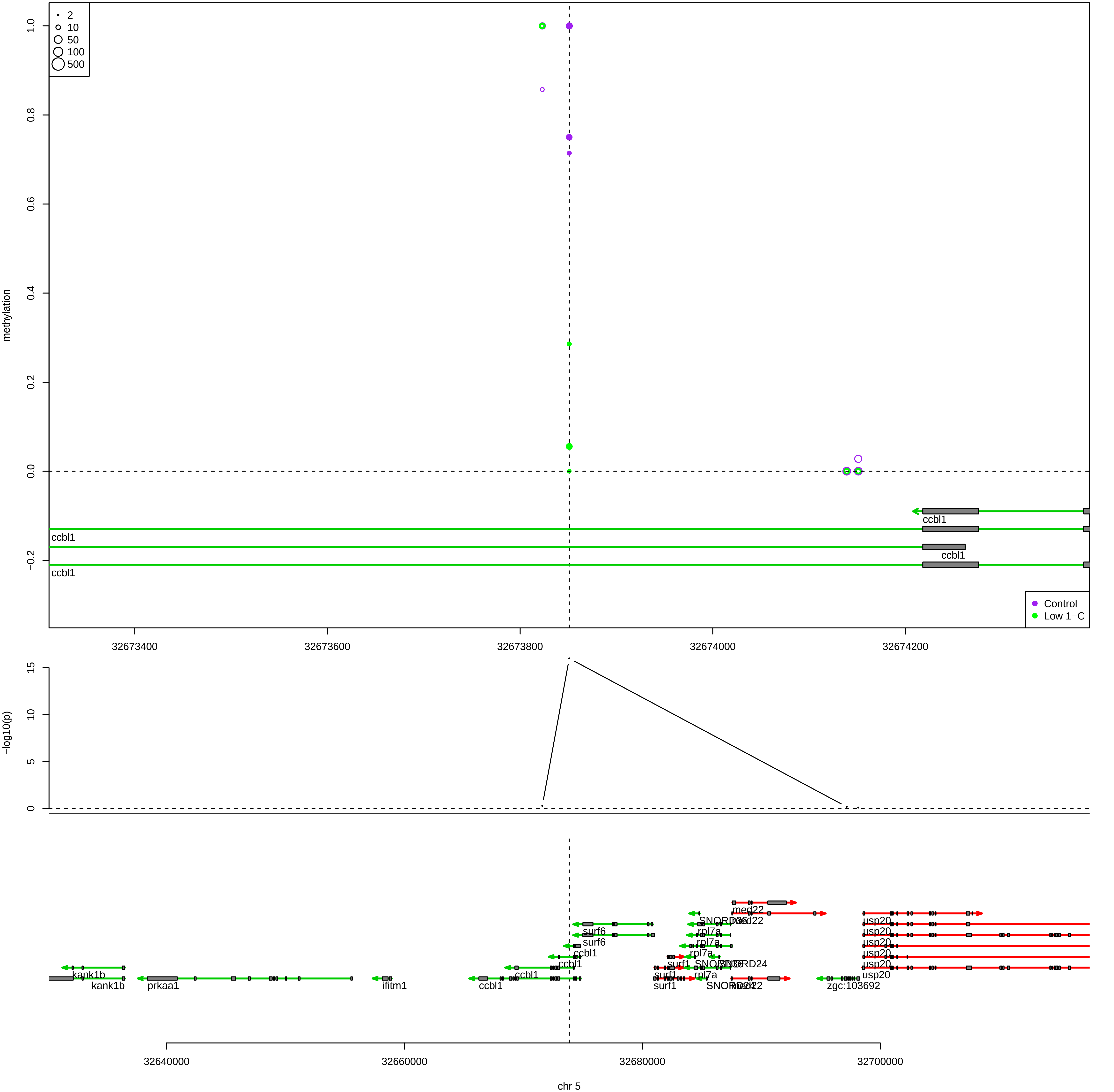

methylation
1.0
0.8
0.6
0.4
0.2
0.0
−0.2
2
10
50
100
500
ccbl1
Control
Low 1−C
32673400
32673600
32673800
32674000
32674200
−log10(p)
15
10
5
0
kank1b
prkaa1
ifitm1
surf6
rpl7a
med22
usp20
surf1
SNORD24
zgc:103692
32640000
32660000
32680000
32700000
chr 5

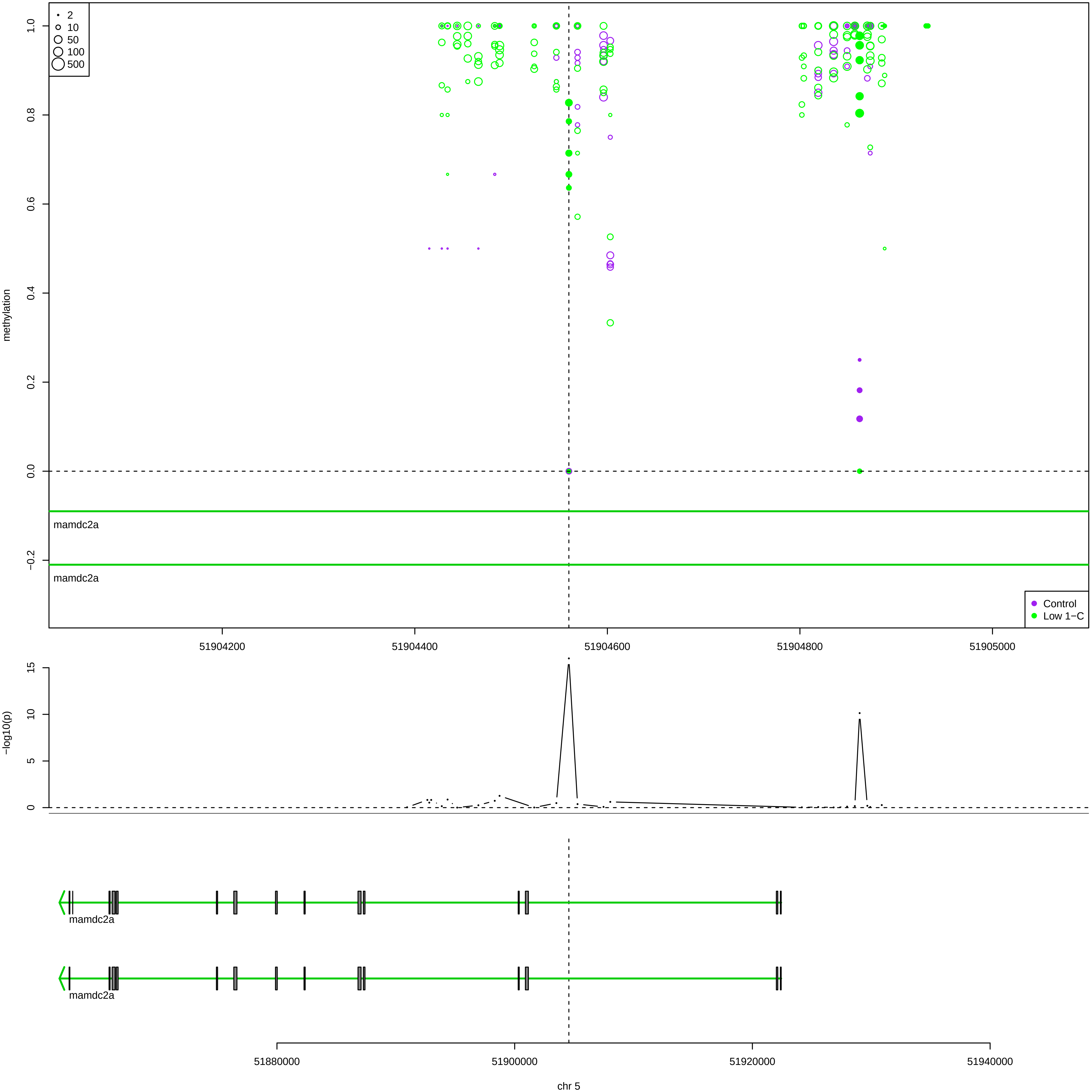

2
10
50
100
500
methylation
1.0
0.8
0.6
0.4
0.2
0.0
−0.2
mamdc2a
mamdc2a
Control
Low 1−C
51904200
51904400
51904600
51904800
51905000
−log10(p)
15
10
5
0
mamdc2a
mamdc2a
51880000
51900000
51920000
51940000
chr 5

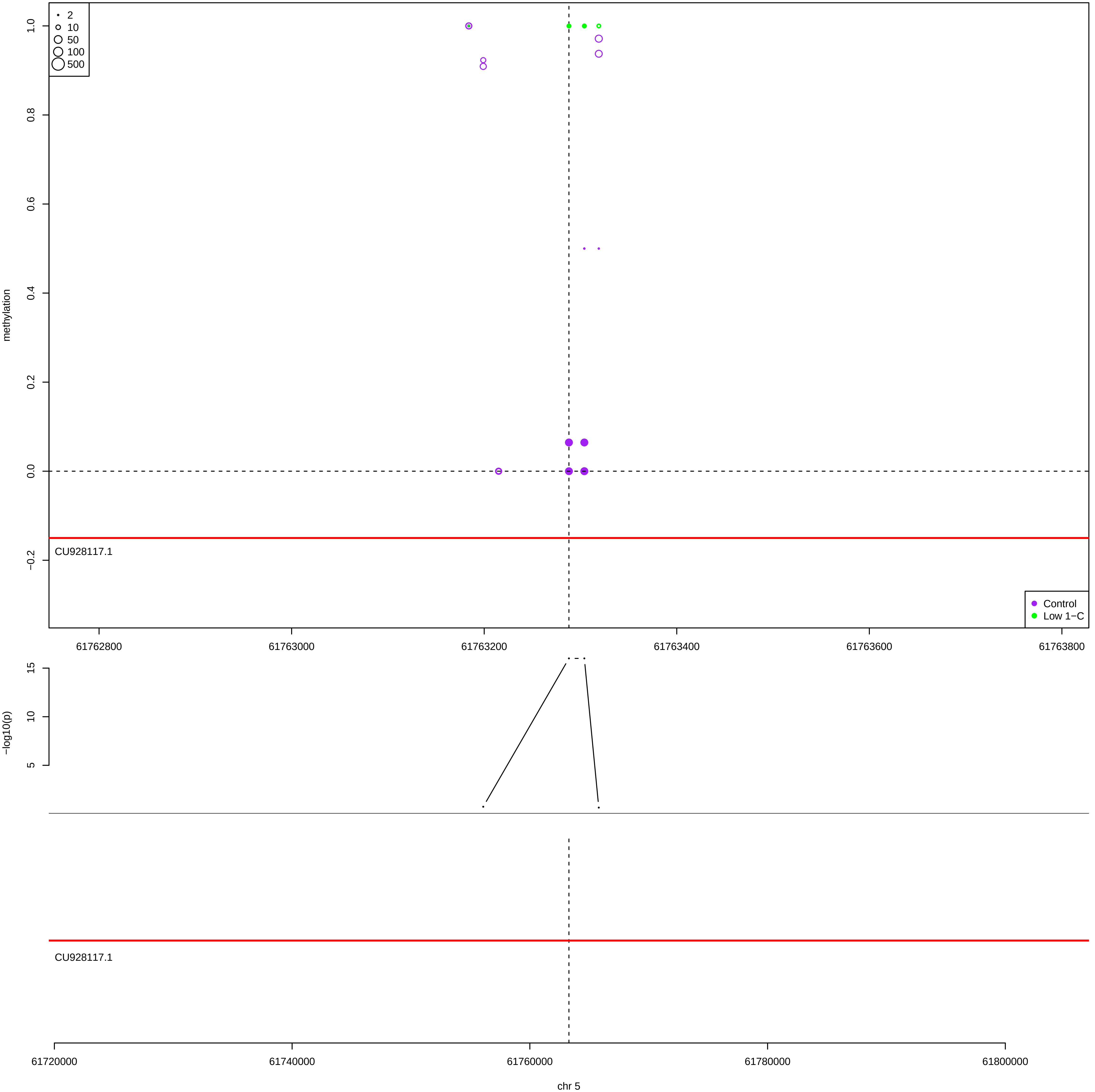

2
10
50
100
500
1.0
0.8
0.6
0.4
0.2
0.0
−0.2
methylation
CU928117.1
Control
Low 1−C
61762800
61763000
61763200
61763400
61763600
61763800
15
10
5
−log10(p)
CU928117.1
61720000
61740000
61760000
61780000
61800000
chr 5

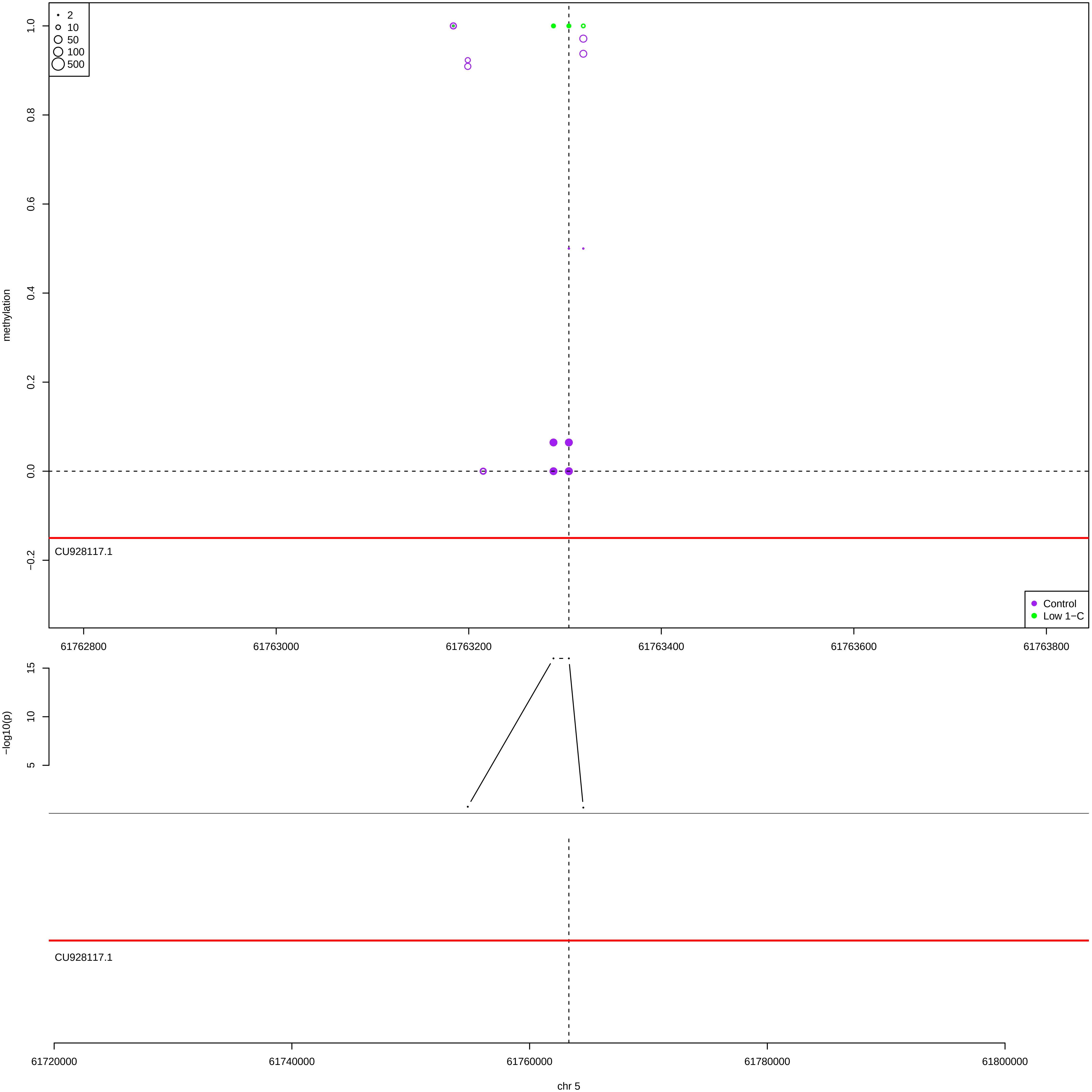

2
10
50
100
500
methylation
1.0
0.8
0.6
0.4
0.2
0.0
−0.2
CU928117.1
Control
Low 1−C
61762800
61763000
61763200
61763400
61763600
61763800
−log10(p)
15
10
5
CU928117.1
61720000
61740000
61760000
61780000
61800000
chr 5

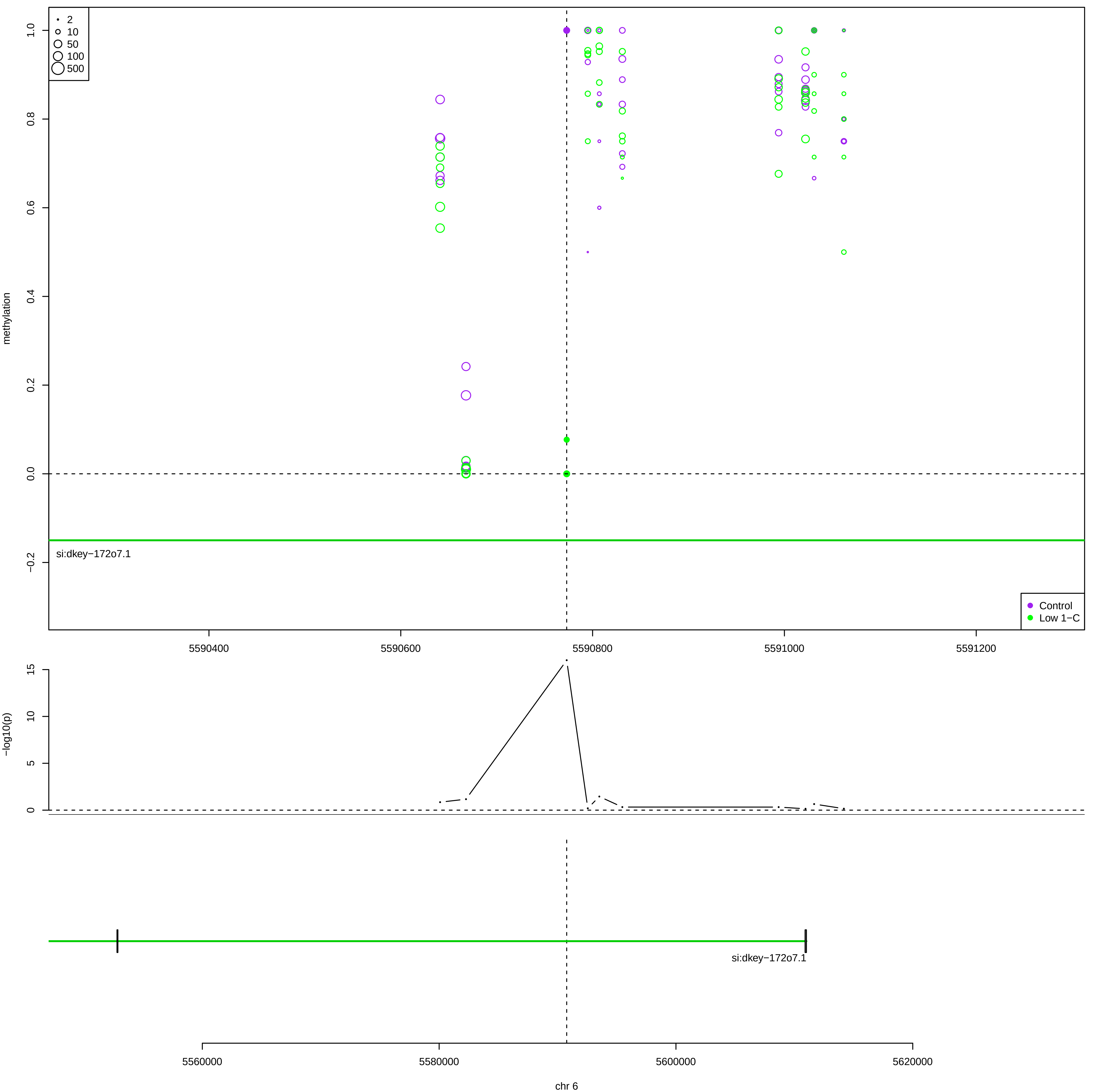

2
10
50
100
500
methylation
1.0
0.8
0.6
0.4
0.2
0.0
−0.2
si:dkey−172o7.1
Control
Low 1−C
5590400
5590600
5590800
5591000
5591200
−log10(p)
15
10
5
0
si:dkey−172o7.1
5560000
5580000
5600000
5620000
chr 6

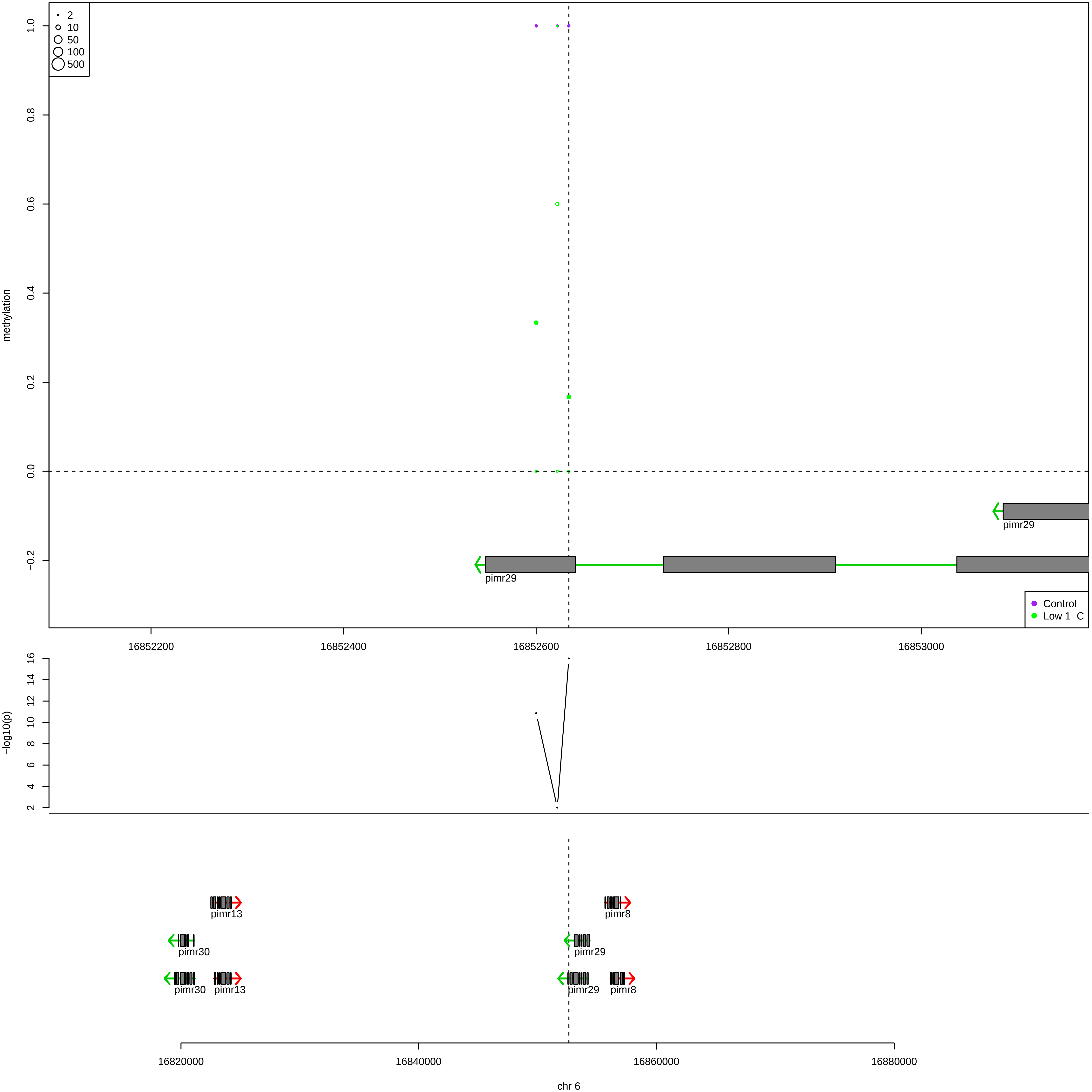

2
10
50
100
500
methylation
1.0
0.8
0.6
0.4
0.2
0.0
−0.2
pimr29
pimr29
Control
Low 1−C
16852200
16852400
16852600
16852800
16853000
−log10(p)
16
14
12
10
8
6
4
2
pimr13
pimr8
pimr30
pimr29
pimr30
pimr13
pimr29
pimr8
16820000
16840000
16860000
16880000
chr 6

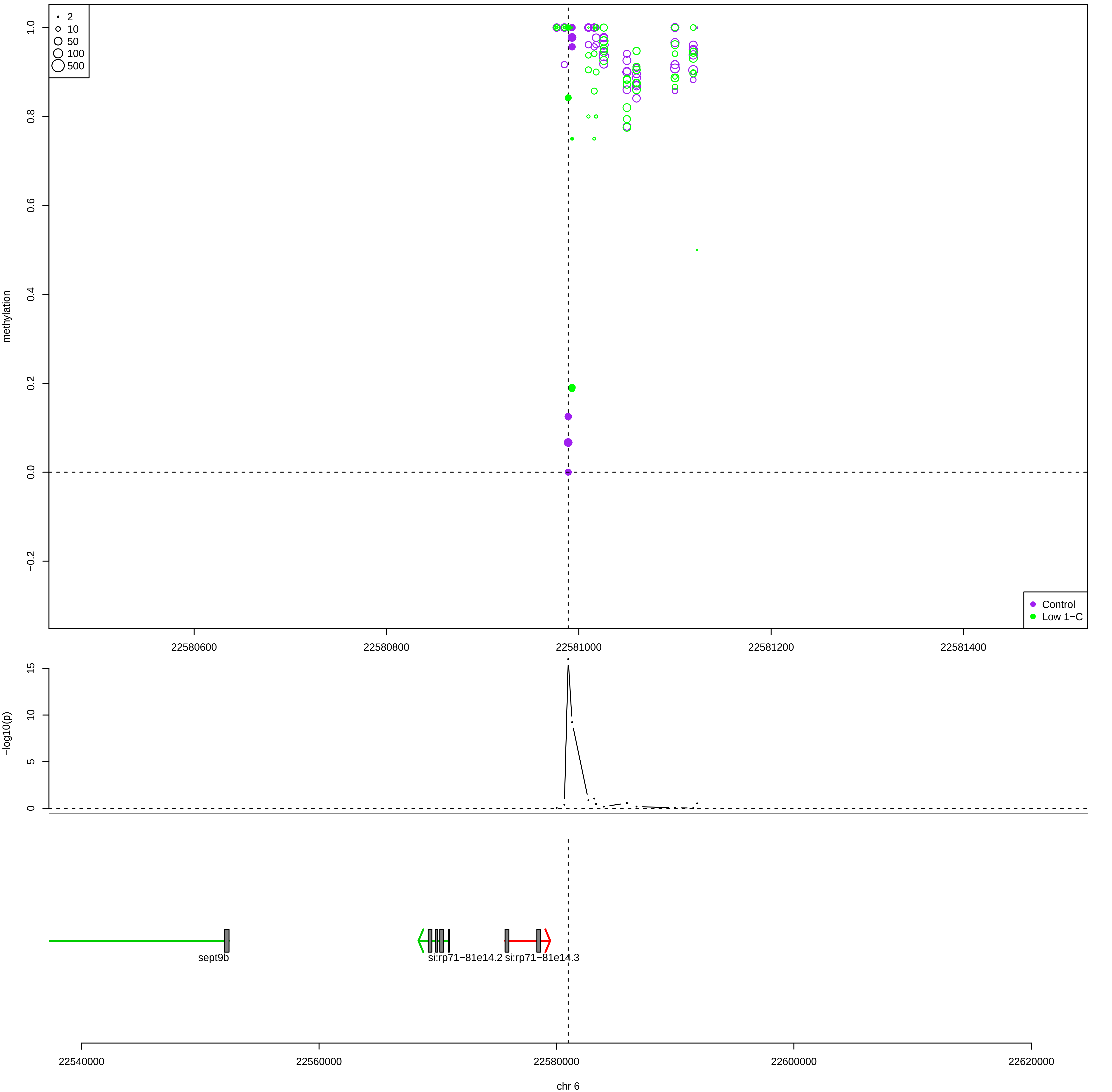

2
10
50
100
500
Control
Low 1−C
methylation
1.0
0.8
0.6
0.4
0.2
0.0
−0.2
22580600
22580800
22581000
22581200
22581400
−log10(p)
15
10
5
0
sept9b
si:rp71−81e14.2
si:rp71−81e14.3
22540000
22560000
22580000
22600000
22620000
chr 6

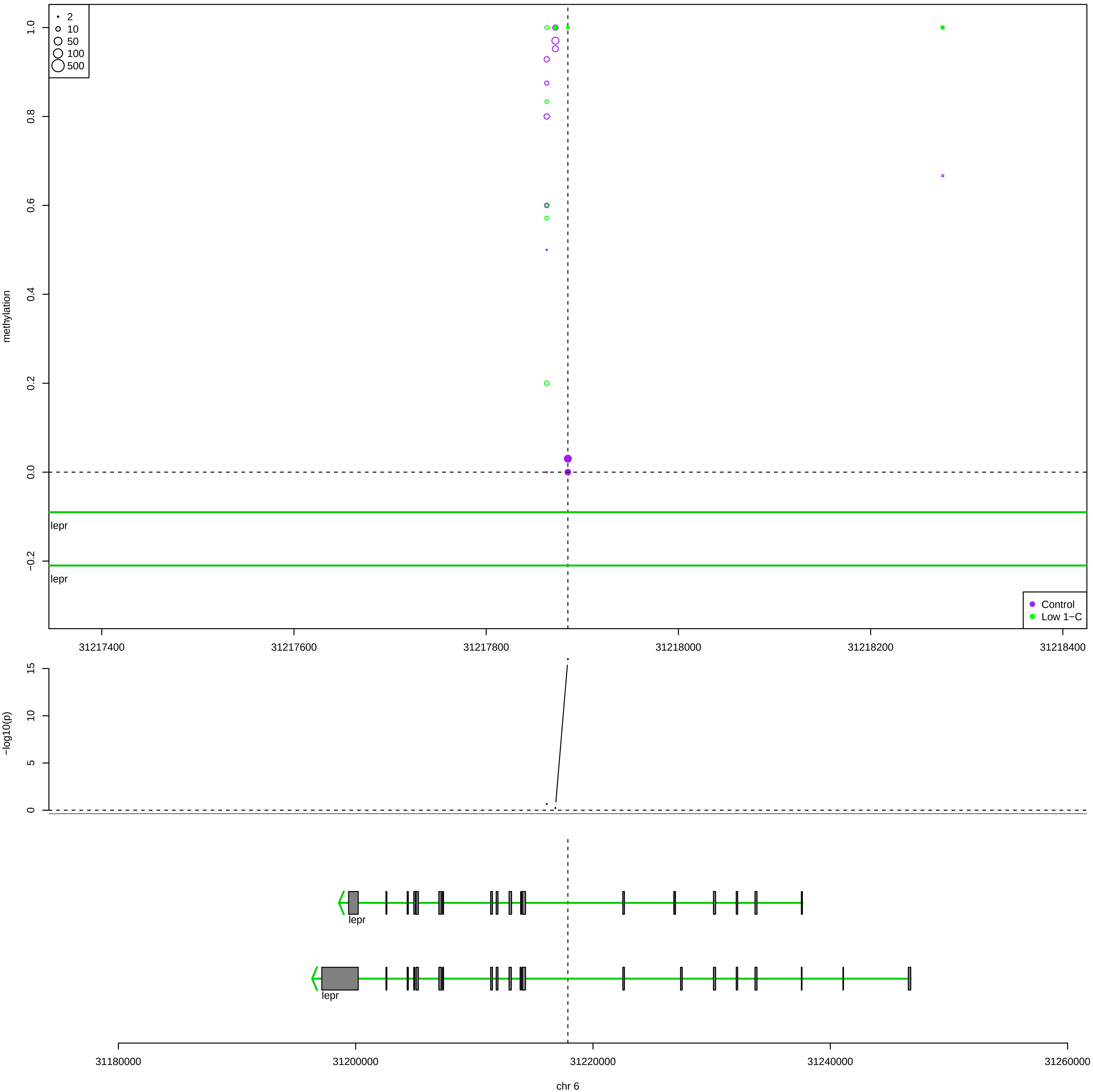

methylation
1.0
0.8
0.6
0.4
0.2
0.0
−0.2
2
10
50
100
500
lepr
lepr
Control
Low 1−C
31217400
31217600
31217800
31218000
31218200
31218400
−log10(p)
15
10
5
0
lepr
lepr
31180000
31200000
31220000
31240000
31260000
chr 6

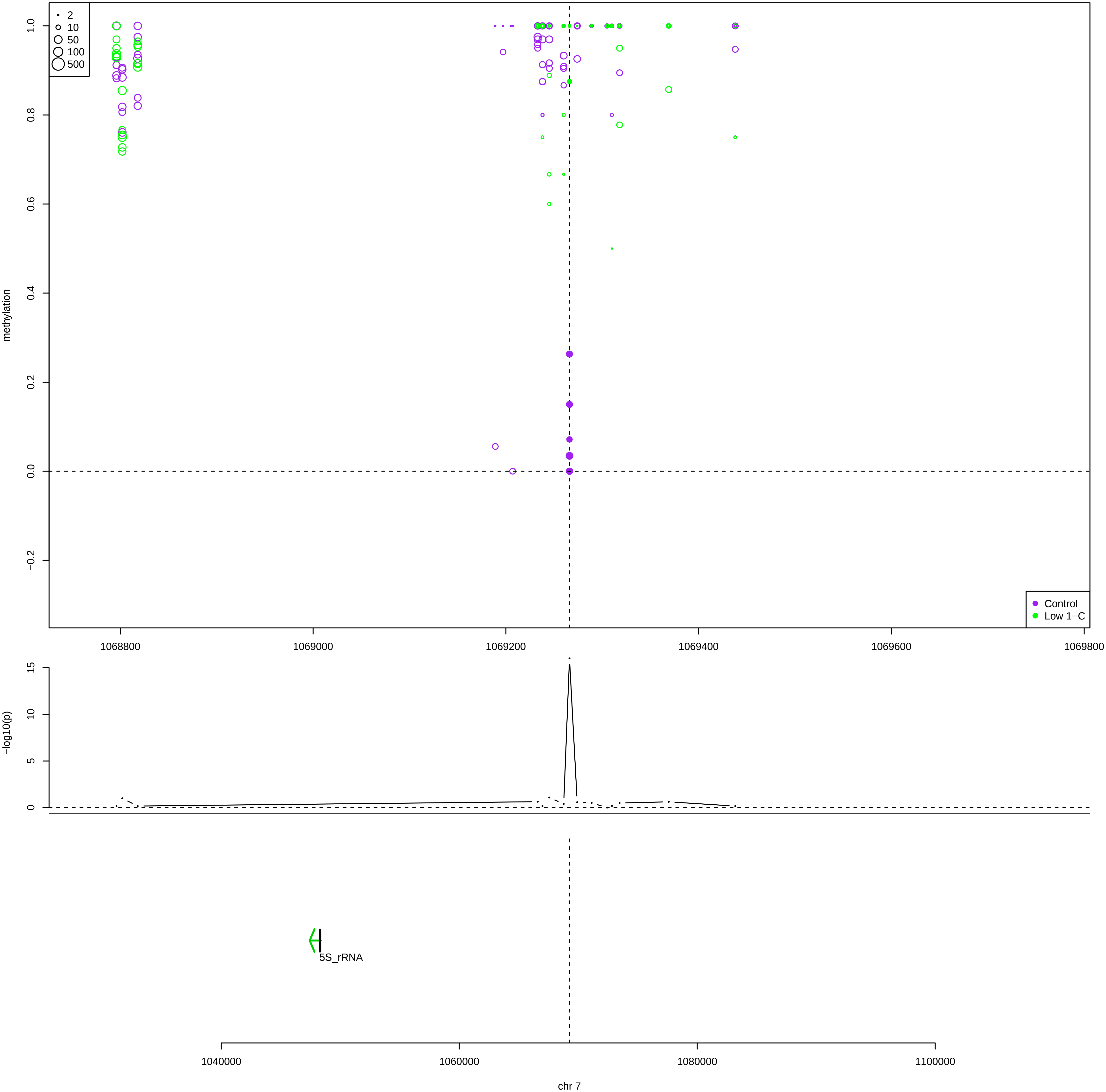

2
10
50
100
500
methylation
1.0
0.8
0.6
0.4
0.2
0.0
−0.2
Control
Low 1−C
1068800
1069000
1069200
1069400
1069600
1069800
−log10(p)
15
10
5
0
5S_rRNA
1040000
1060000
1080000
1100000
chr 7

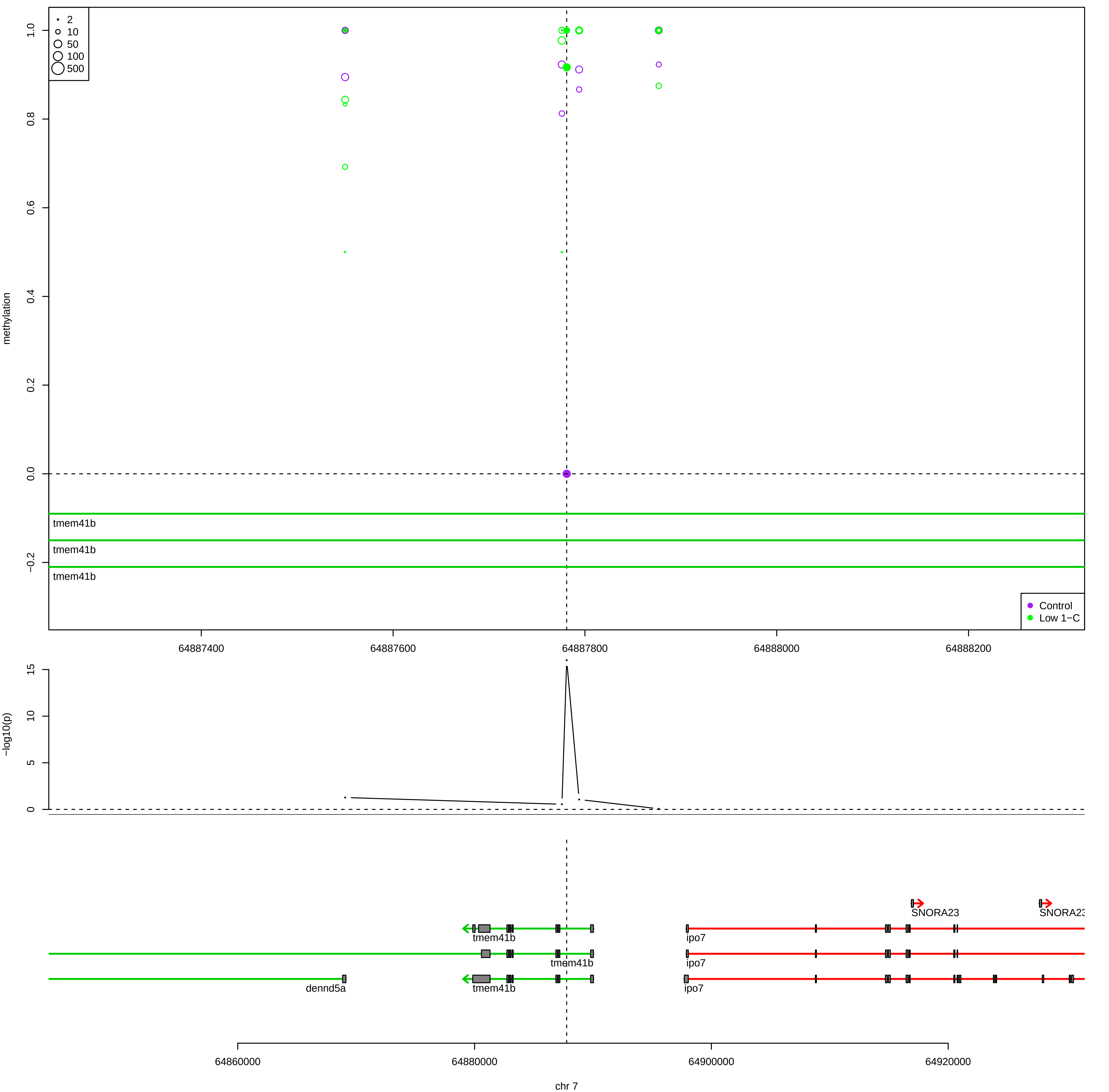
methylation
2
10
50
100
500
Control
Low 1−C
tmem41b
tmem41b
tmem41b
64887400
64887600
64887800
64888000
64888200
−log10(p)
SNORA23
SNORA23
tmem41b
tmem41b
tmem41b
ipo7
ipo7
ipo7
dennd5a
64860000
64880000
64900000
64920000
chr 7

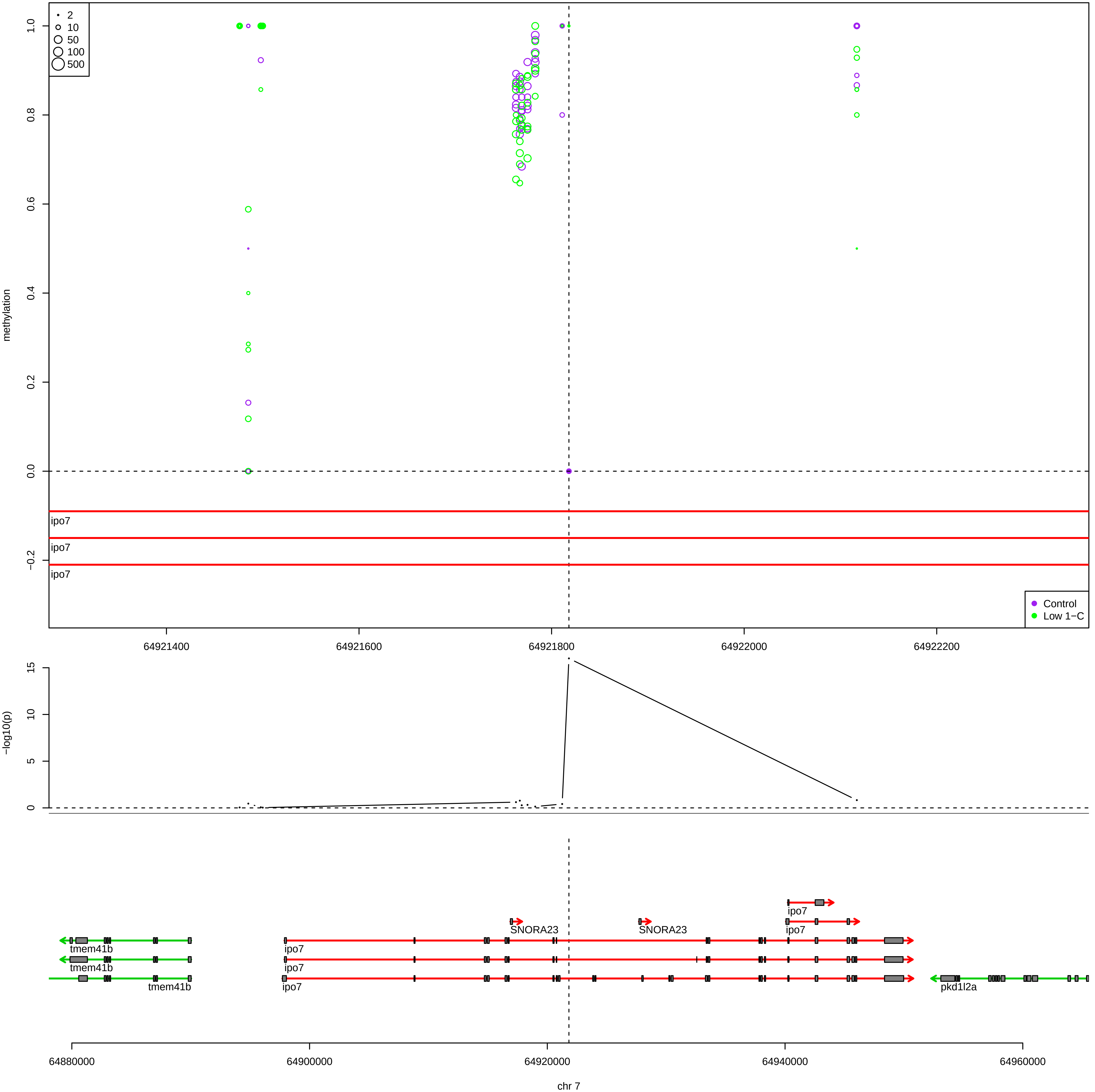

methylation
1.0
0.8
0.6
0.4
0.2
0.0
−0.2
2
10
50
100
500
ipo7
ipo7
ipo7
Control
Low 1−C
64921400
64921600
64921800
64922000
64922200
−log10(p)
15
10
5
0
tmem41b
tmem41b
tmem41b
ipo7
ipo7
ipo7
SNORA23
SNORA23
ipo7
ipo7
pkd1l2a
64880000
64900000
64920000
64940000
64960000
chr 7

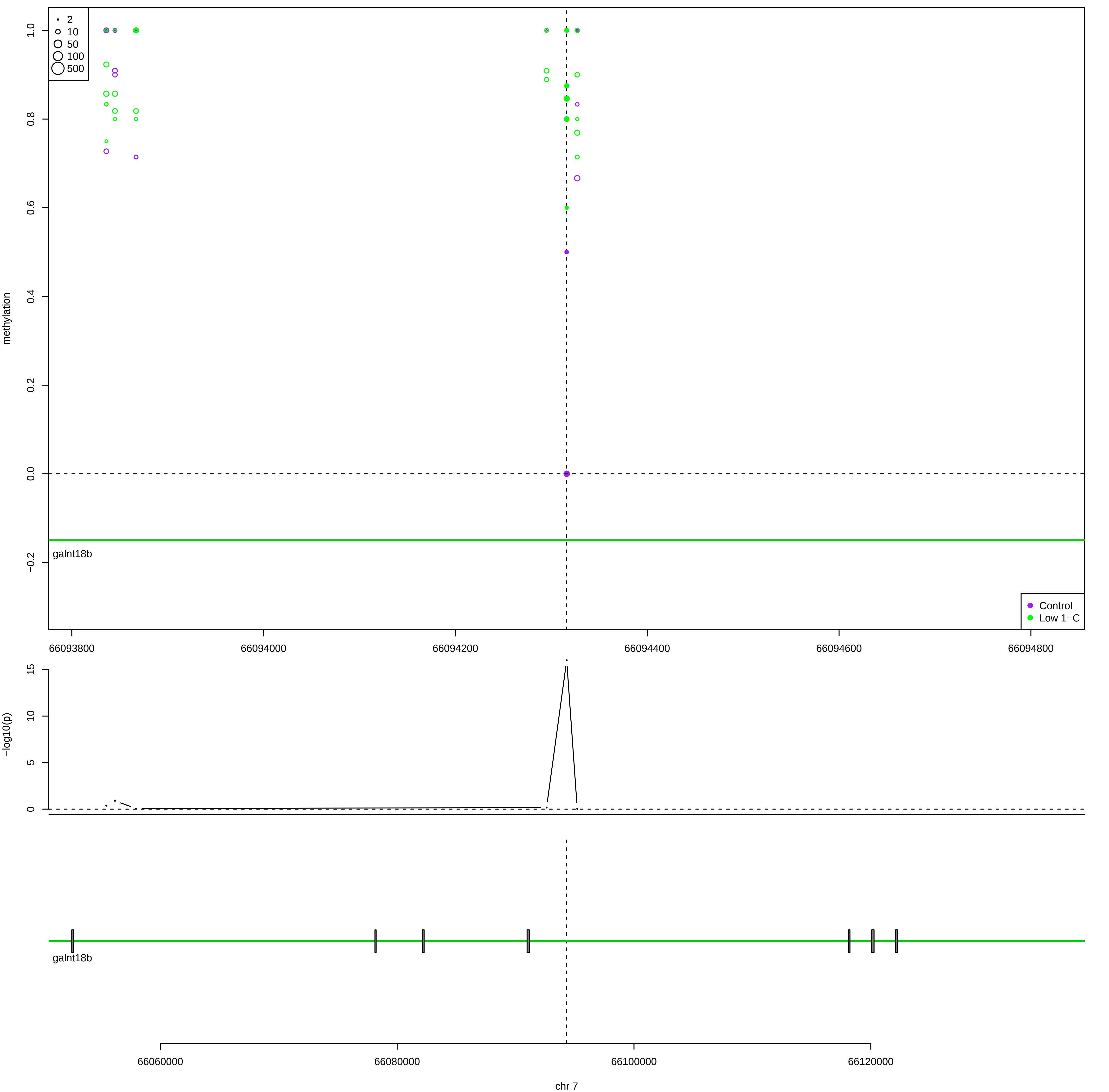

2
10
50
100
500
methylation
1.0
0.8
0.6
0.4
0.2
0.0
−0.2
galnt18b
Control
Low 1−C
66093800
66094000
66094200
66094400
66094600
66094800
−log10(p)
15
10
5
0
galnt18b
66060000
66080000
66100000
66120000
chr 7

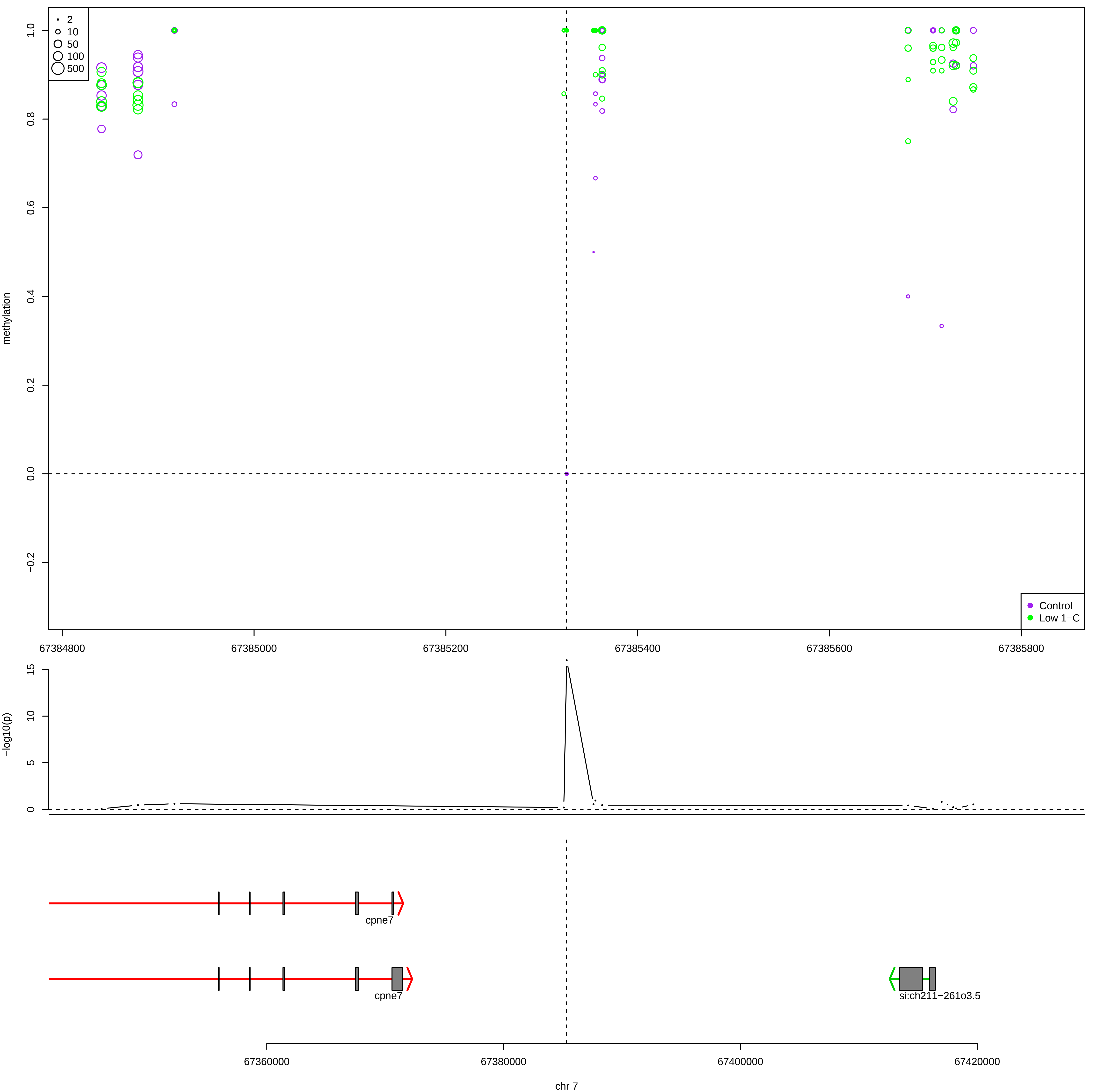

methylation
2
10
50
100
500
1.0
0.8
0.6
0.4
0.2
0.0
-0.2
Control
Low 1−C
67384800
67385000
67385200
67385400
67385600
67385800
−log10(p)
15
10
5
0
cpne7
cpne7
si:ch211−261o3.5
67360000
67380000
67400000
67420000
chr 7

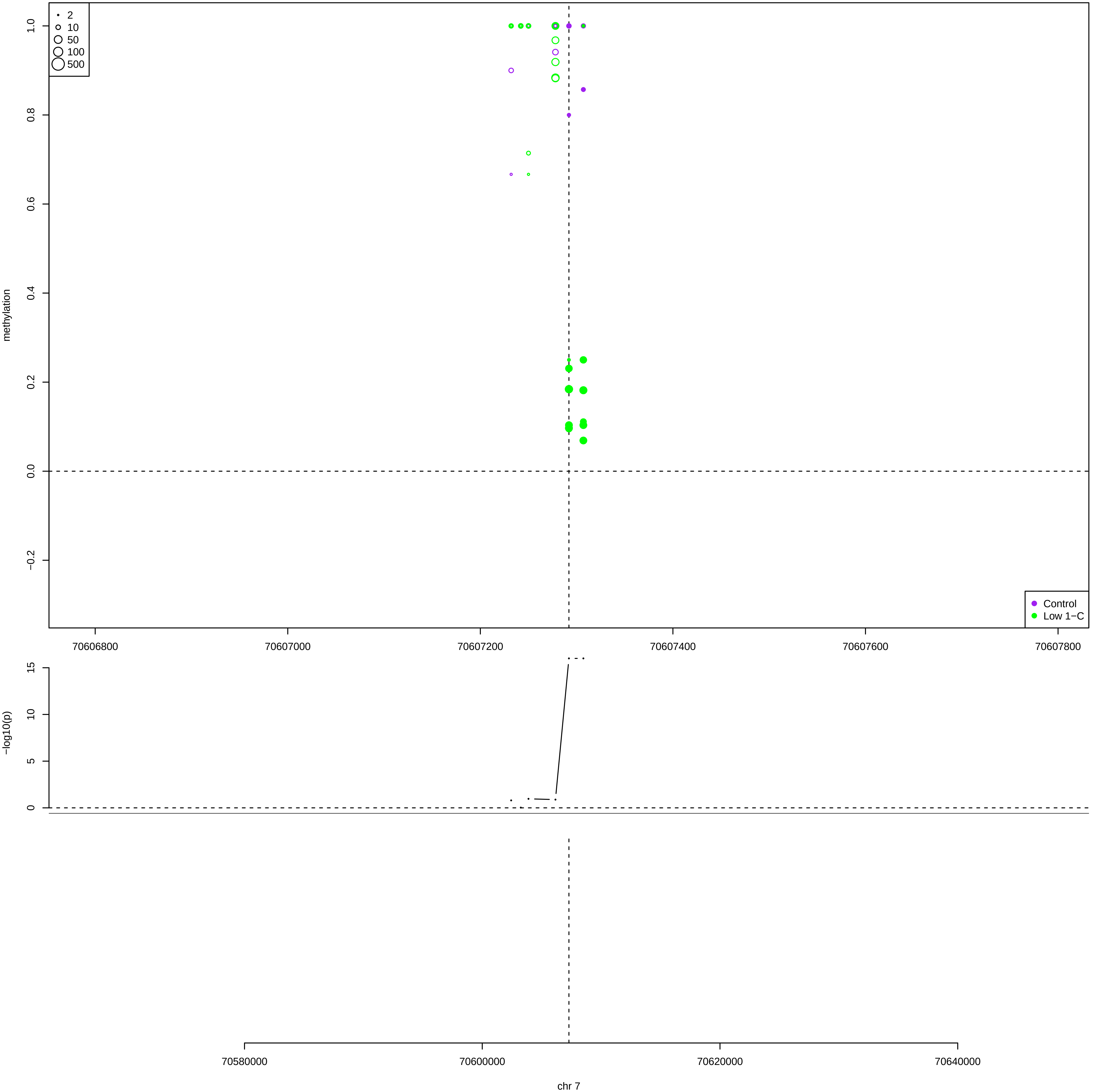

2
10
50
100
500
methylation
1.0
0.8
0.6
0.4
0.2
0.0
−0.2
Control
Low 1−C
70606800
70607000
70607200
70607400
70607600
70607800
−log10(p)
15
10
5
0
70580000
70600000
70620000
70640000
chr 7

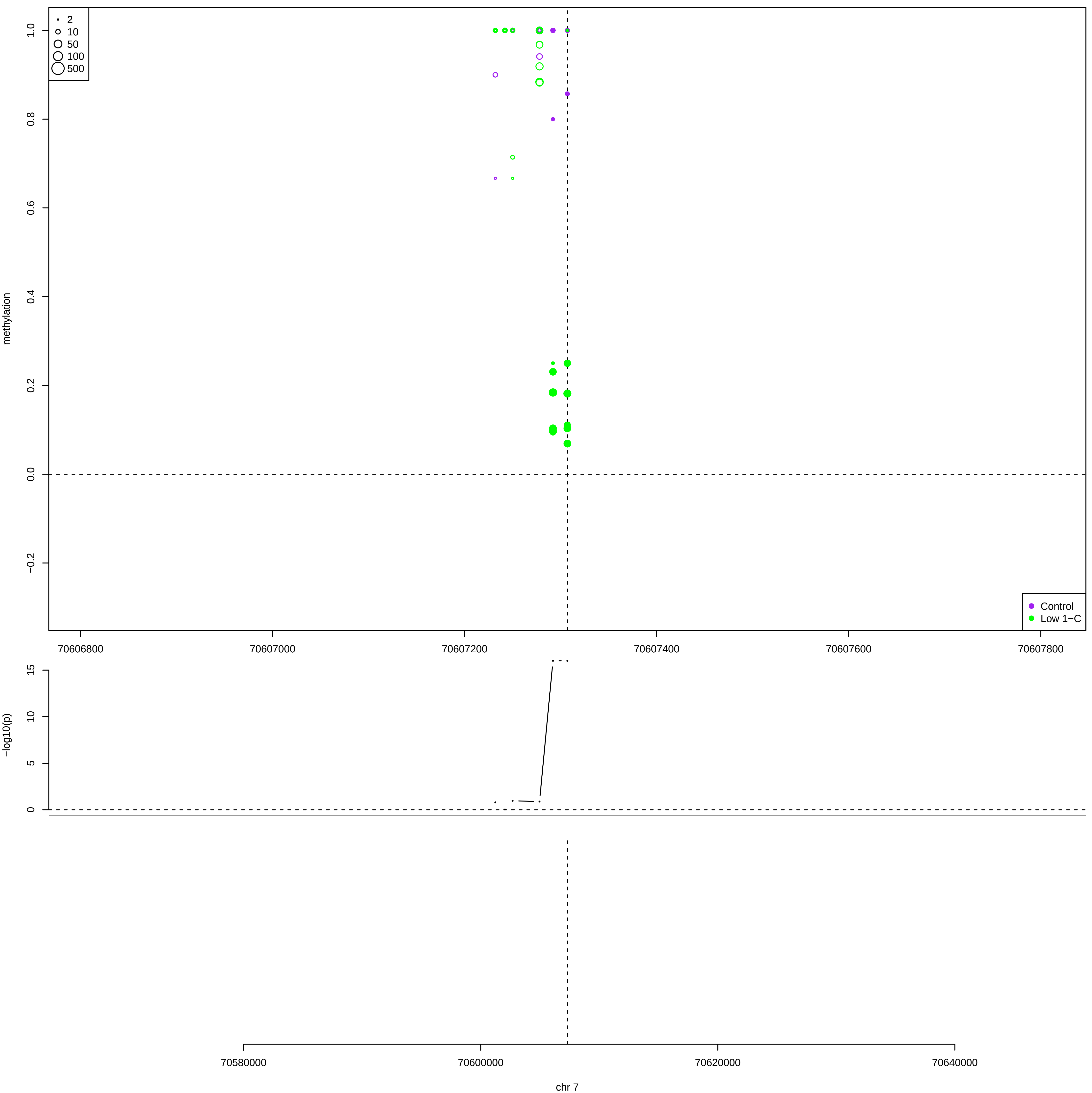

2
10
50
100
500
Control
Low 1−C
methylation
1.0
0.8
0.6
0.4
0.2
0.0
−0.2
70606800
70607000
70607200
70607400
70607600
70607800
−log10(p)
15
10
5
0
70580000
70600000
70620000
70640000
chr 7

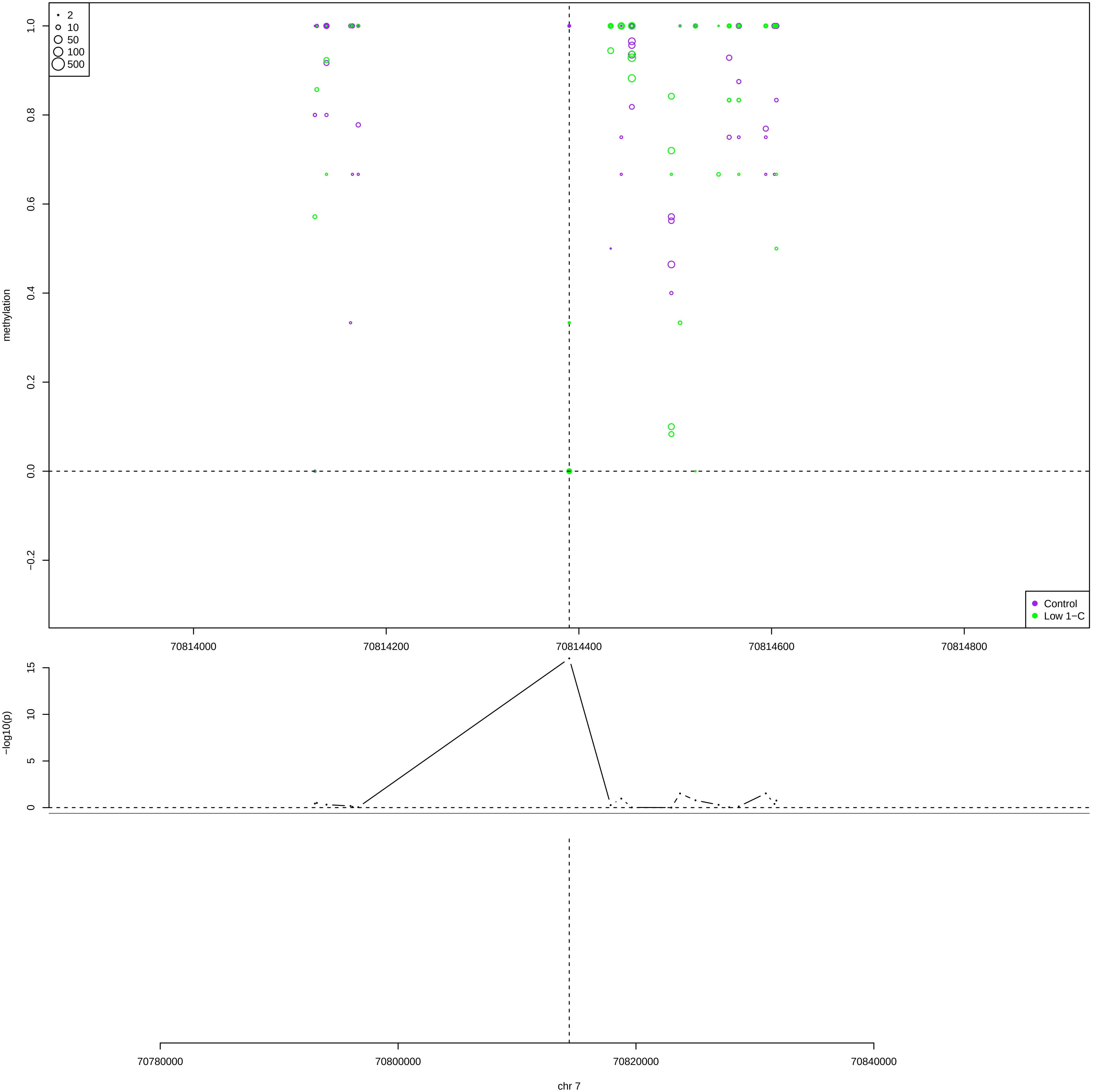
2
10
50
100
500
methylation
1.0
0.8
0.6
0.4
0.2
0.0
−0.2
Control
Low 1−C
70814000
70814200
70814400
70814600
70814800
−log10(p)
15
10
5
0
70780000
70800000
70820000
70840000
chr 7

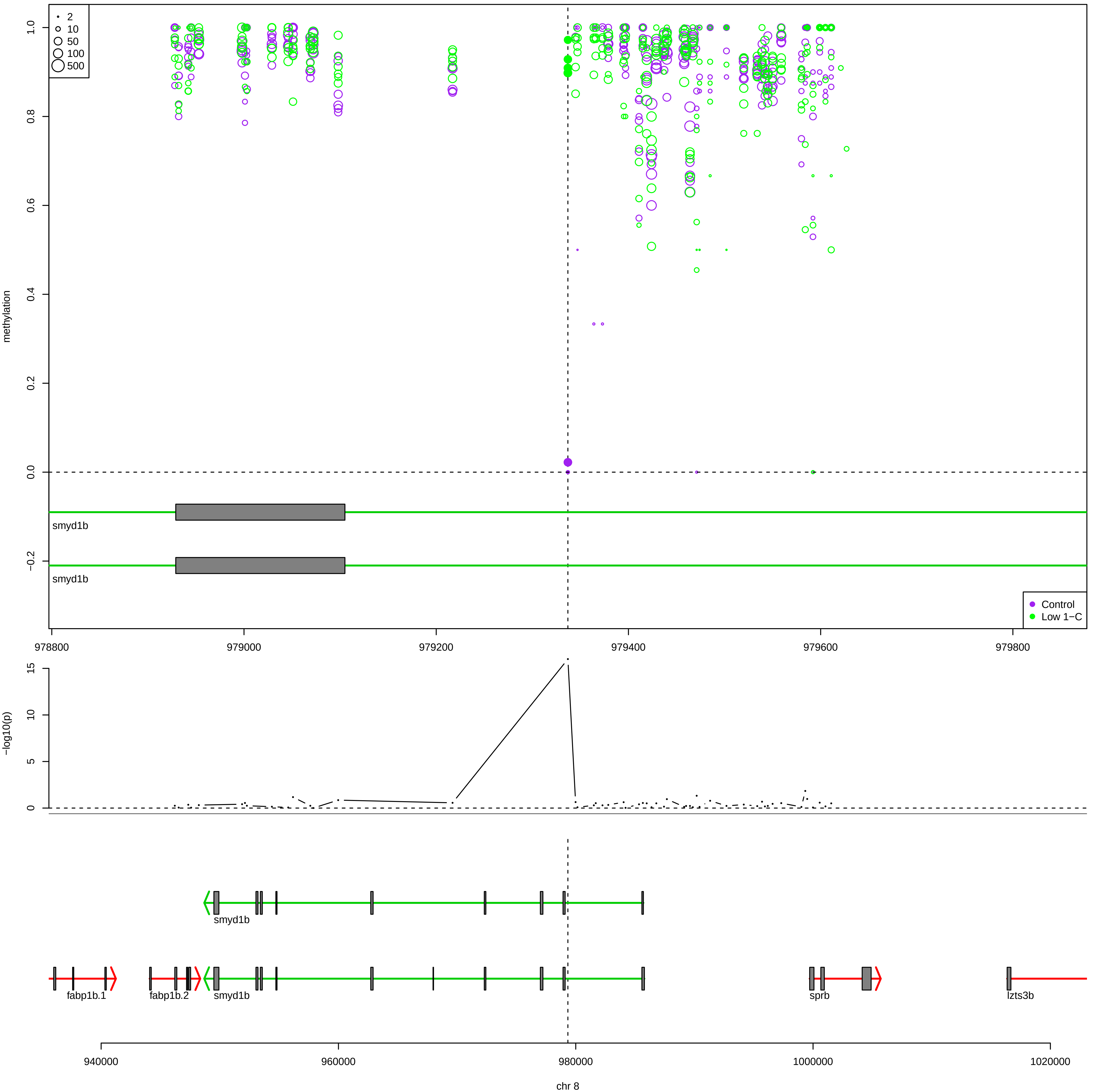

2
10
50
100
500
methylation
1.0
0.8
0.6
0.4
0.2
0.0
−0.2
smyd1b
smyd1b
Control
Low 1−C
978800
979000
979200
979400
979600
979800
−log10(p)
15
10
5
0
smyd1b
fabp1b.1
fabp1b.2
smyd1b
sprb
lzts3b
940000
960000
980000
1000000
1020000
chr 8

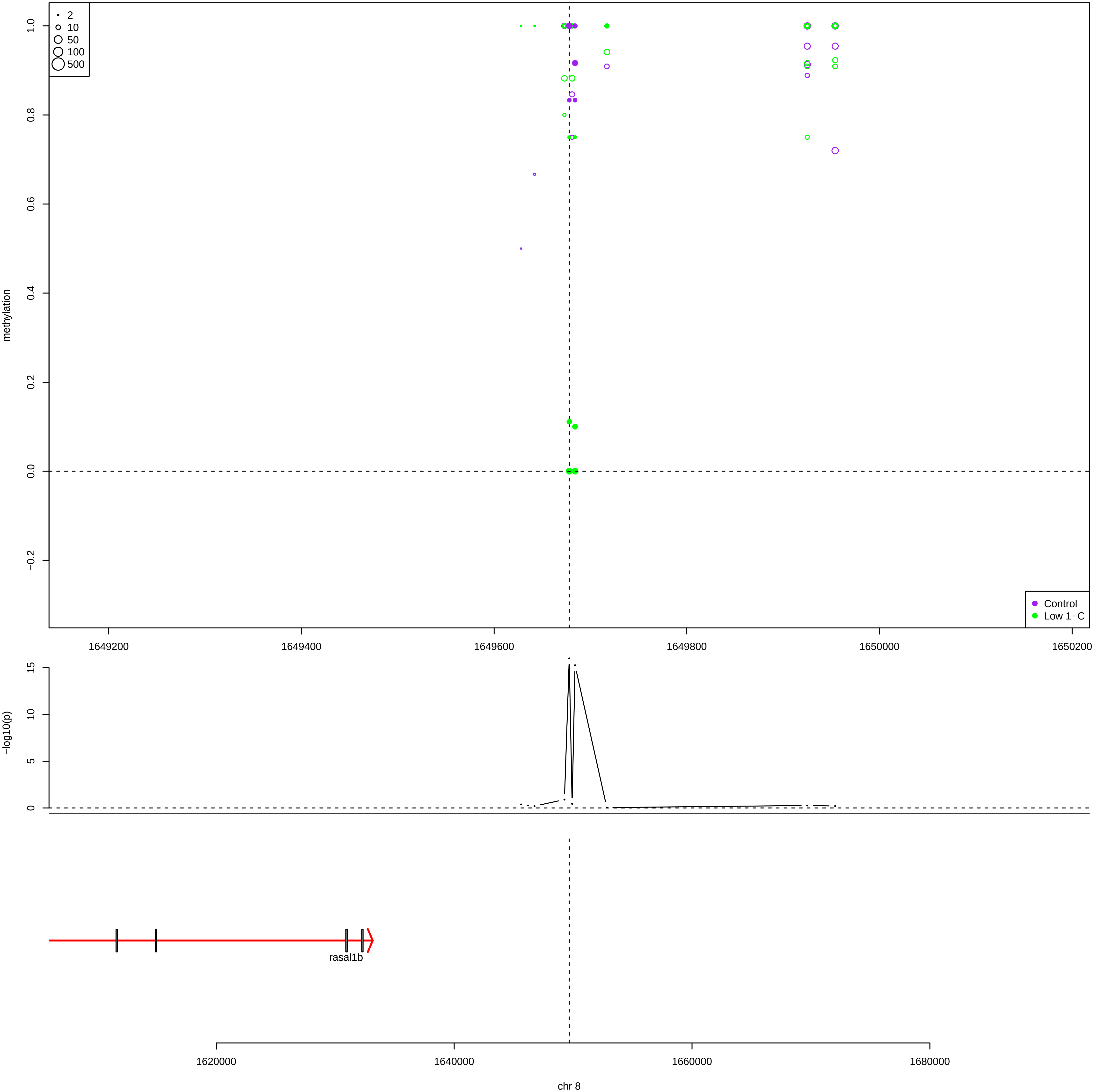

2
10
50
100
500
methylation
1.0
0.8
0.6
0.4
0.2
0.0
−0.2
Control
Low 1−C
1649200
1649400
1649600
1649800
1650000
1650200
−log10(p)
15
10
5
0
rasal1b
1620000
1640000
1660000
1680000
chr 8

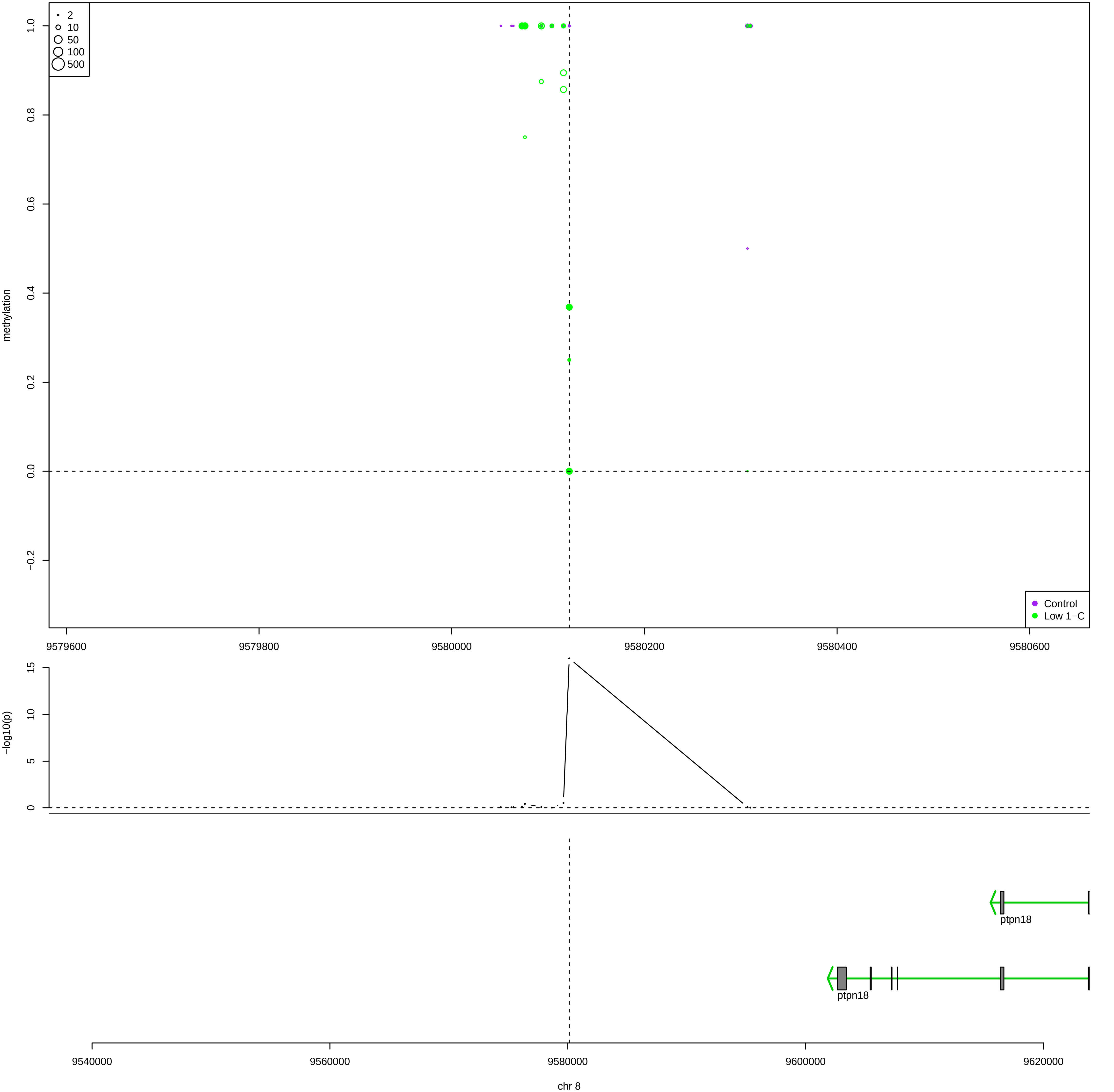

2
10
50
100
500
methylation
1.0
0.8
0.6
0.4
0.2
0.0
−0.2
Control
Low 1−C
9579600
9579800
9580000
9580200
9580400
9580600
−log10(p)
15
10
5
0
ptpn18
ptpn18
9540000
9560000
9580000
9600000
9620000
chr 8

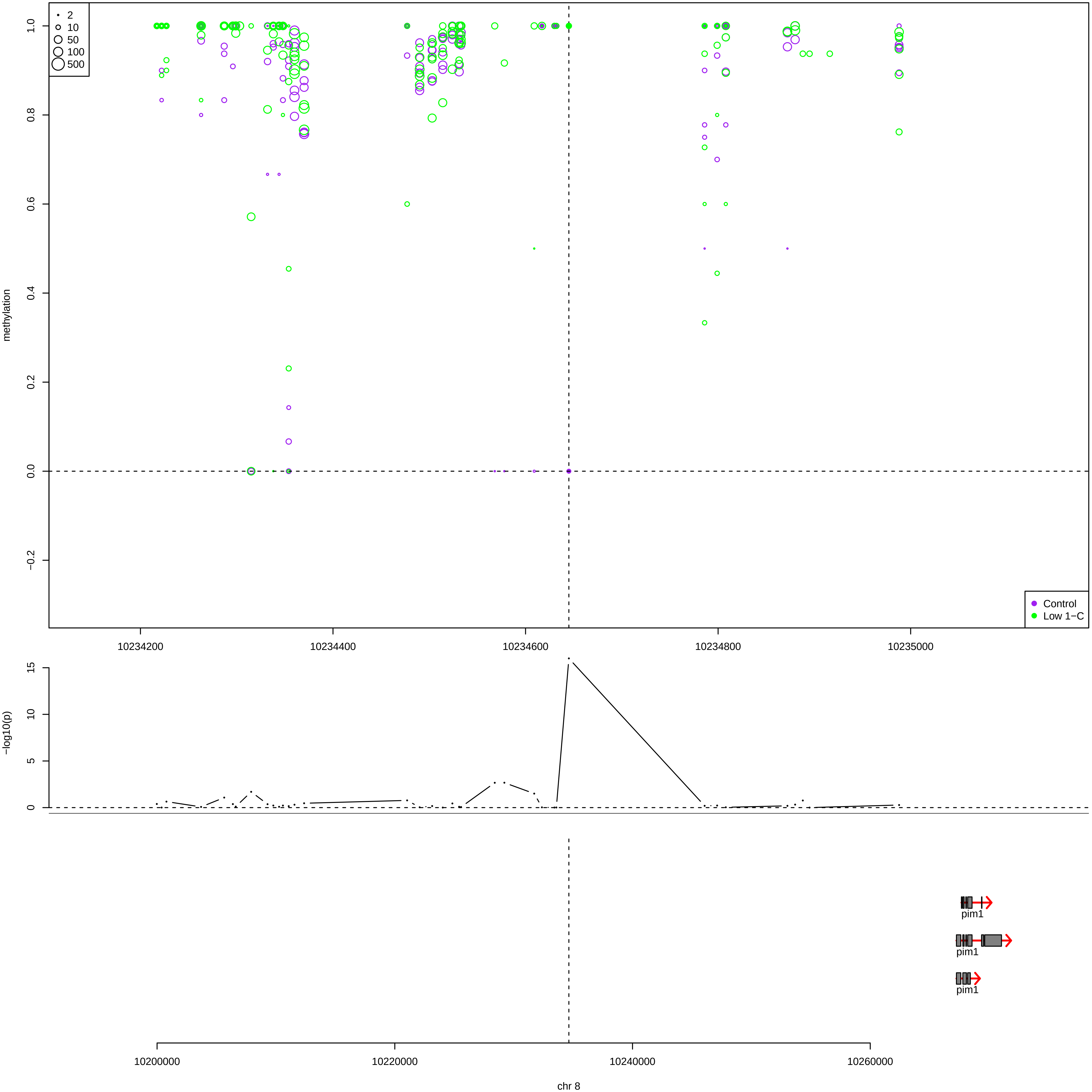

2
10
50
100
500
methylation
1.0
0.8
0.6
0.4
0.2
0.0
−0.2
Control
Low 1−C
10234200
10234400
10234600
10234800
10235000
−log10(p)
15
10
5
0
pim1
pim1
pim1
10200000
10220000
10240000
10260000
chr 8

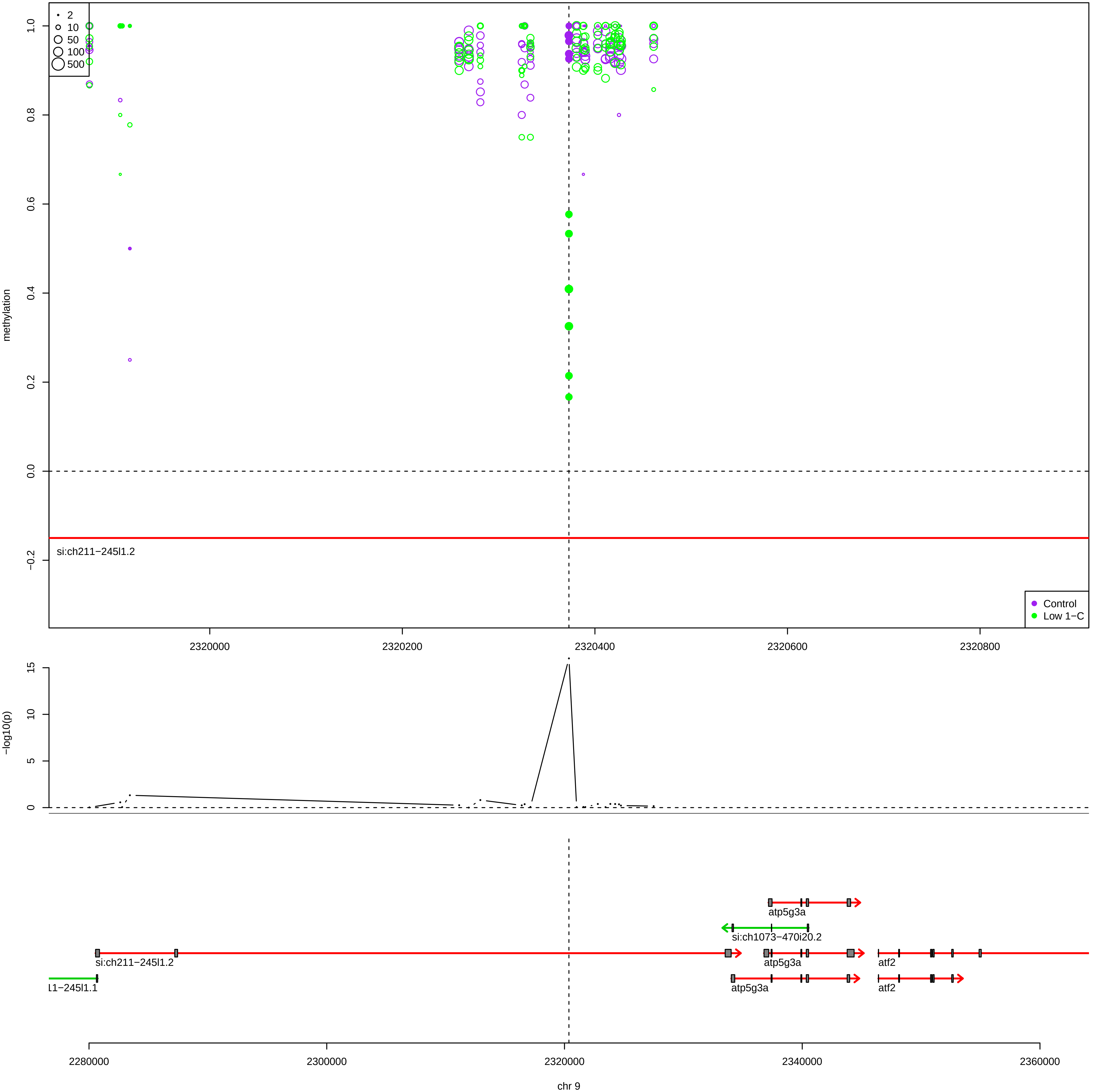
2
10
50
100
500
1.0
0.8
0.6
0.4
0.2
0.0
−0.2
methylation
si:ch211−245l1.2
Control
Low 1−C
2320000
2320200
2320400
2320600
2320800
15
10
5
0
−log10(p)
atp5g3a
si:ch1073−470i20.2
si:ch211−245l1.2
atp5g3a
atf2
l1−245l1.1
atp5g3a
atf2
2280000
2300000
2320000
2340000
2360000
chr 9

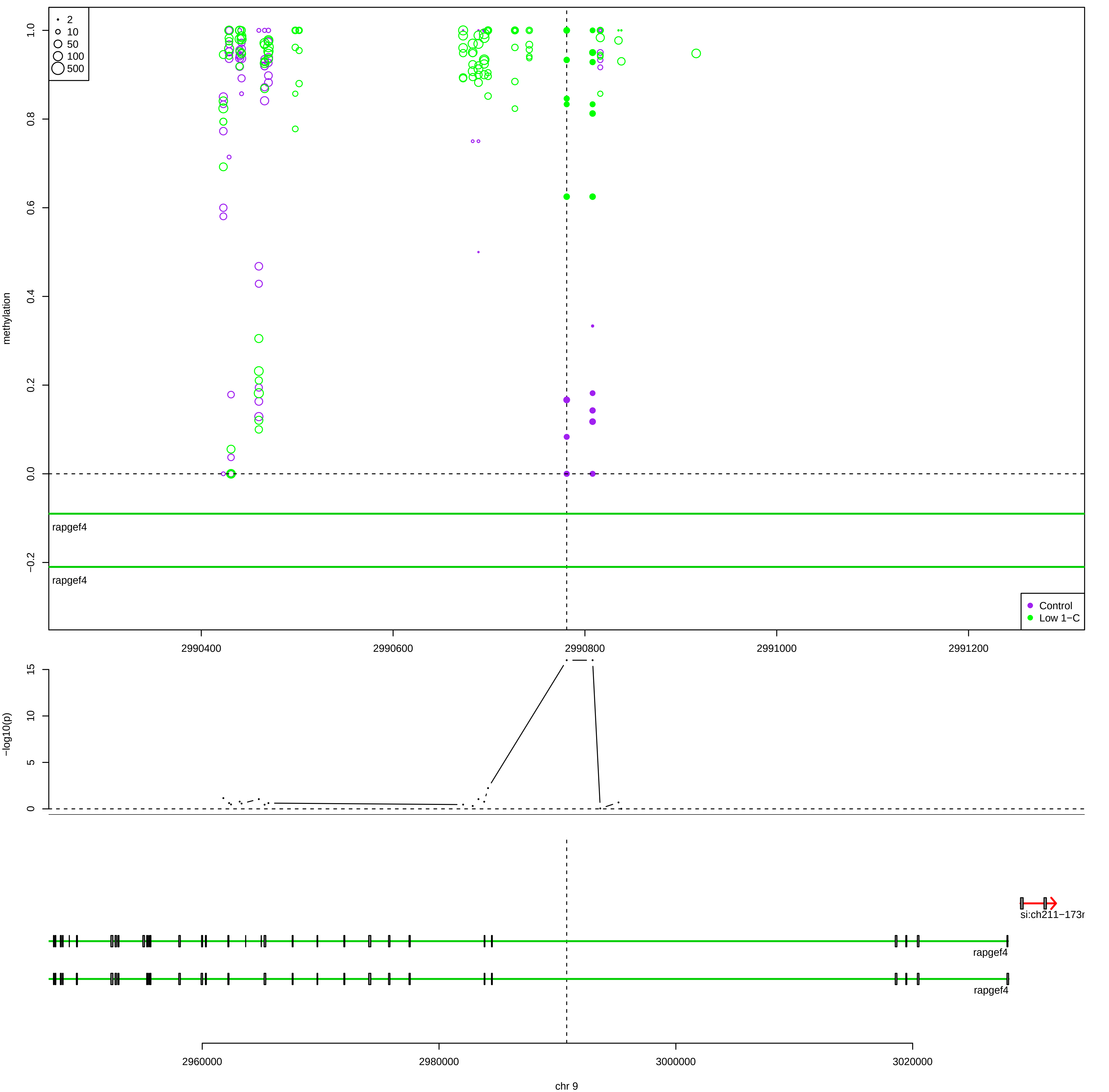

2
10
50
100
500
methylation
1.0
0.8
0.6
0.4
0.2
0.0
−0.2
rapgef4
rapgef4
Control
Low 1−C
2990400
2990600
2990800
2991000
2991200
−log10(p)
15
10
5
0
si:ch211−173n
rapgef4
rapgef4
2960000
2980000
3000000
3020000
chr 9

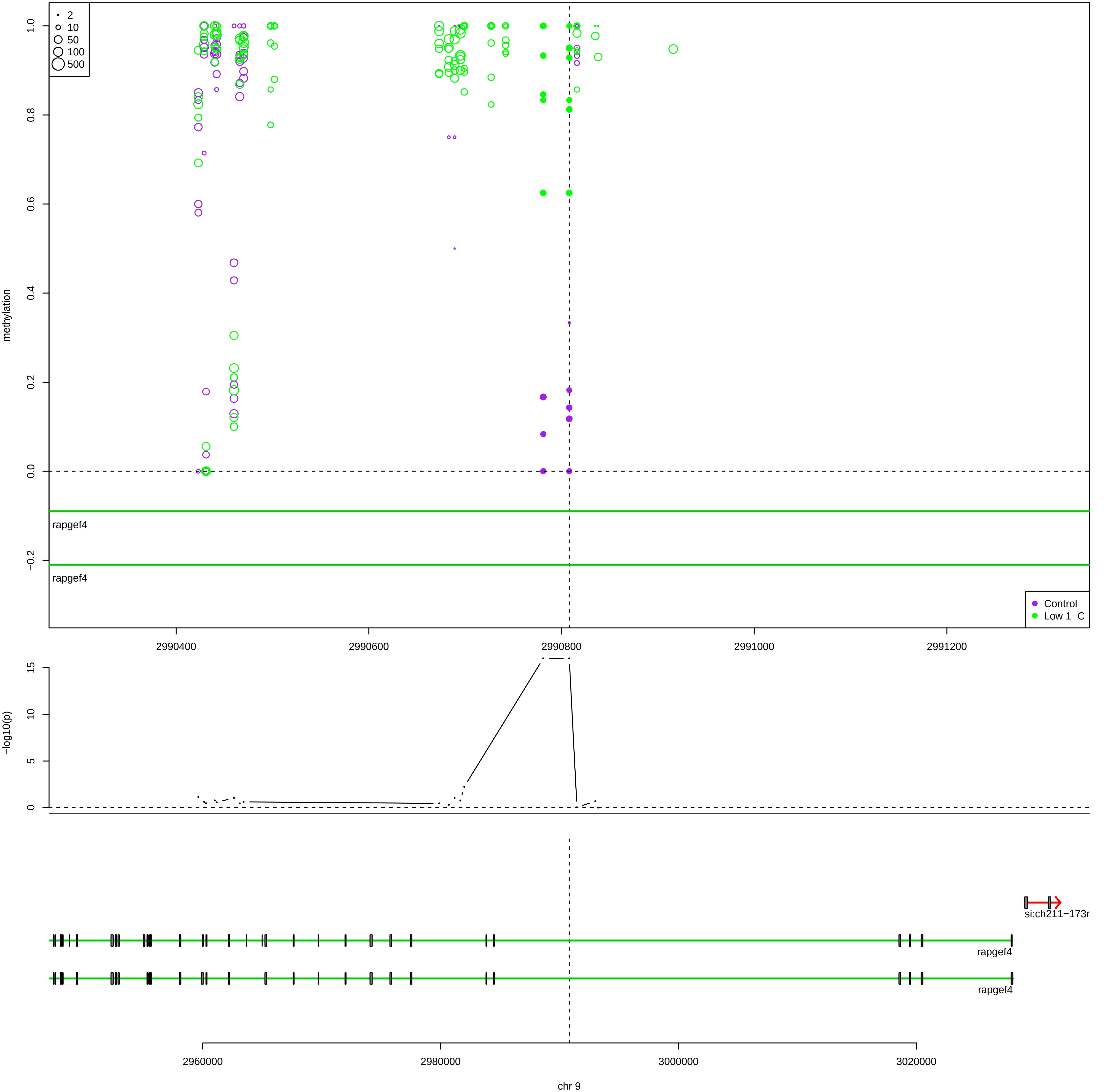

2
10
50
100
500
methylation
1.0
0.8
0.6
0.4
0.2
0.0
−0.2
rapgef4
rapgef4
Control
Low 1−C
2990400
2990600
2990800
2991000
2991200
−log10(p)
15
10
5
0
si:ch211−173r
rapgef4
rapgef4
2960000
2980000
3000000
3020000
chr 9

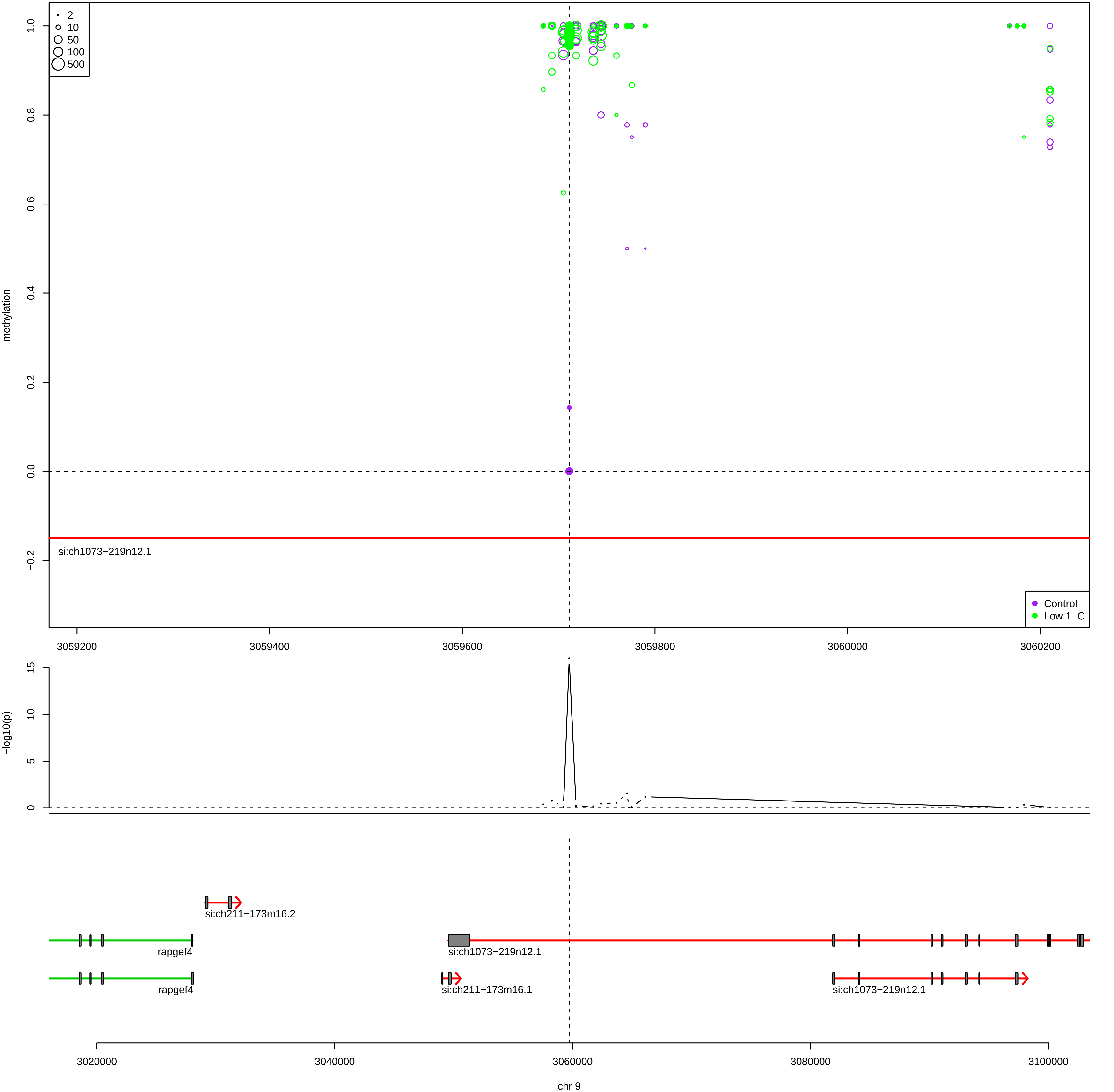

2
10
50
100
500
methylation
1.0
0.8
0.6
0.4
0.2
0.0
−0.2
si:ch1073−219n12.1
Control
Low 1−C
3059200
3059400
3059600
3059800
3060000
3060200
−log10(p)
15
10
5
0
si:ch211−173m16.2
rapgef4
si:ch1073−219n12.1
rapgef4
si:ch211−173m16.1
si:ch1073−219n12.1
3020000
3040000
3060000
3080000
3100000
chr 9

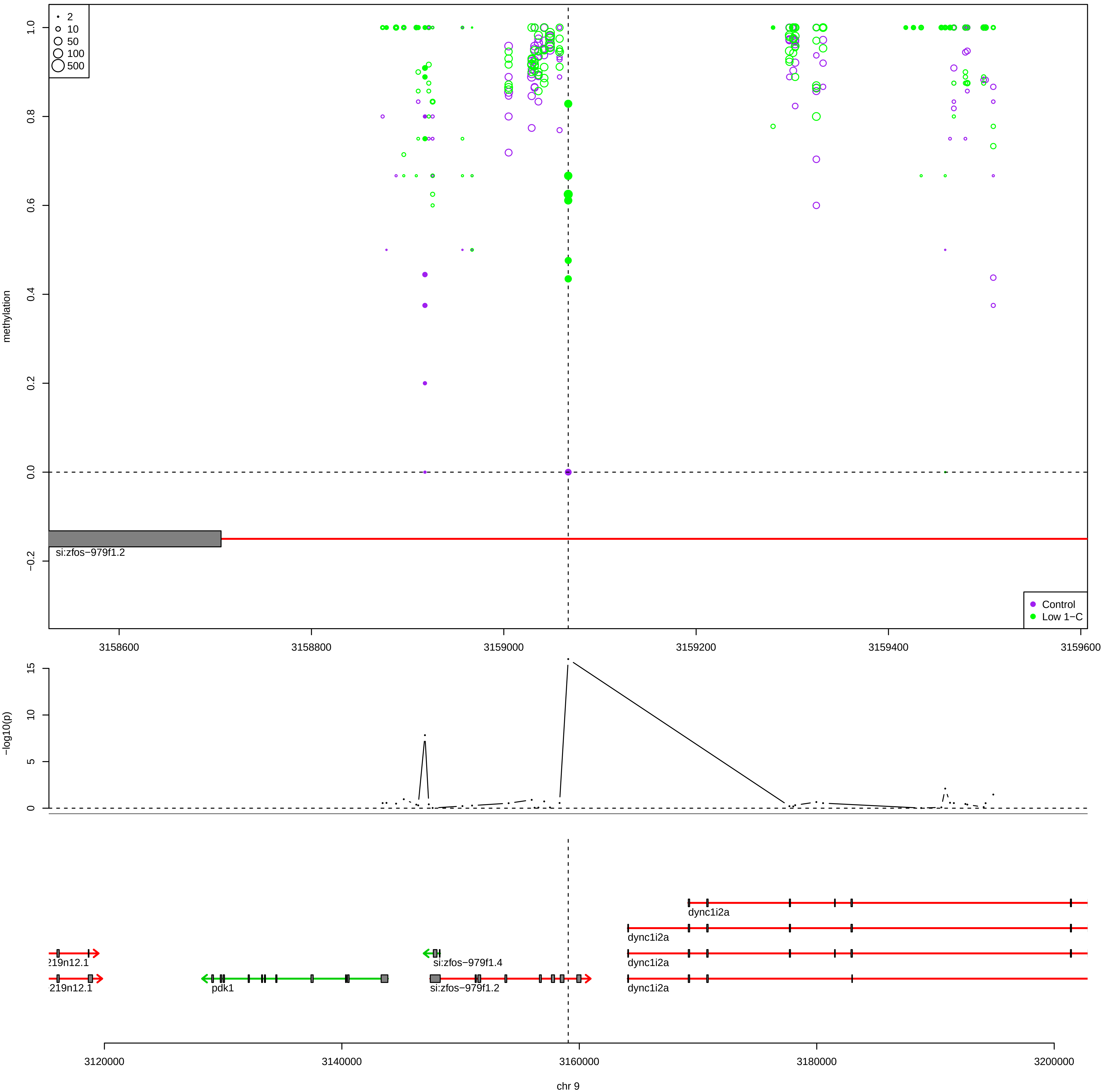
2
10
50
100
500
methylation
1.0
0.8
0.6
0.4
0.2
0.0
−0.2
si:zfos−979f1.2
Control
Low 1−C
3158600
3158800
3159000
3159200
3159400
3159600
−log10(p)
15
10
5
0
dync1i2a
dync1i2a
dync1i2a
dync1i2a
219n12.1
219n12.1
pdk1
si:zfos−979f1.4
si:zfos−979f1.2
3120000
3140000
3160000
3180000
3200000
chr 9

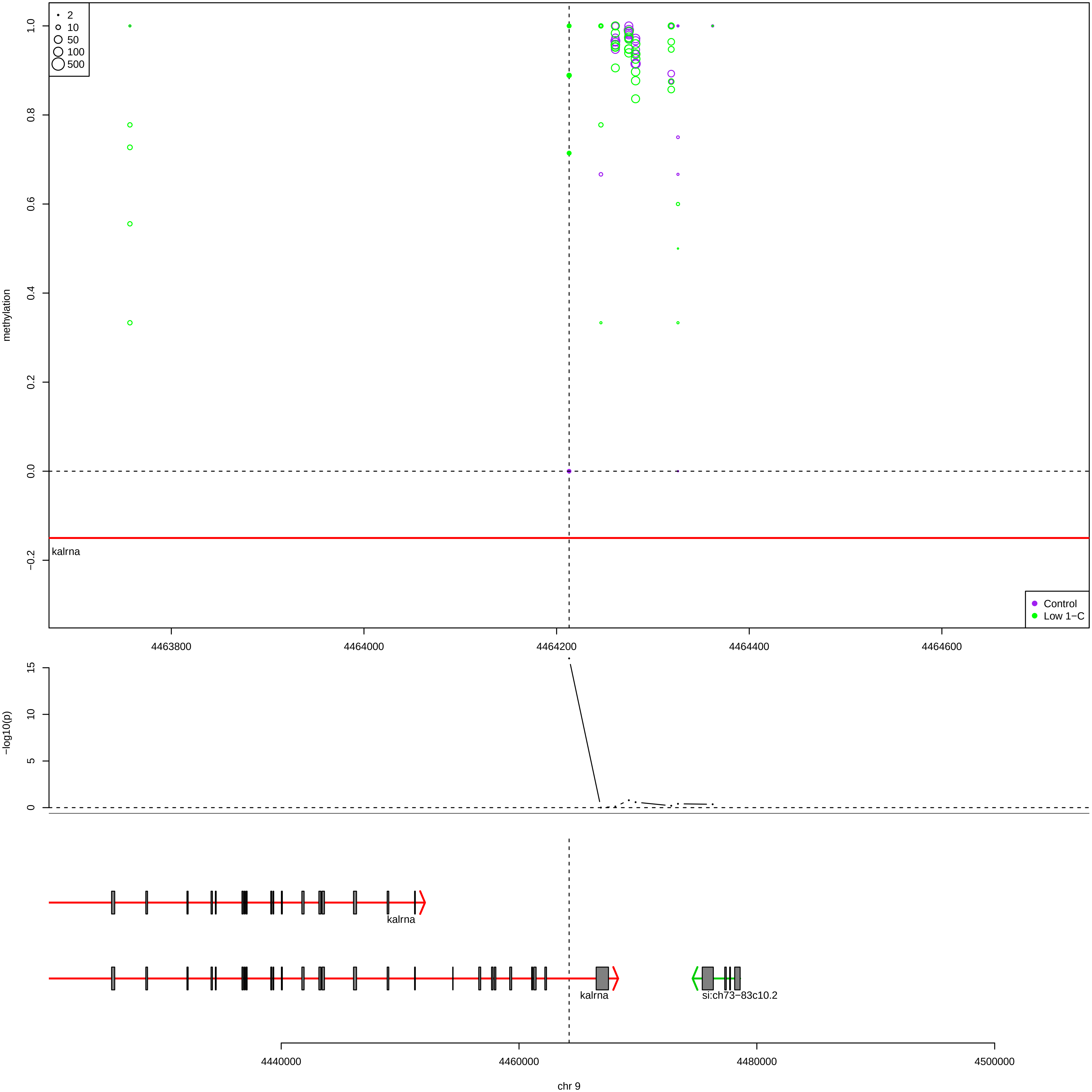

2
10
50
100
500
methylation
1.0
0.8
0.6
0.4
0.2
0.0
−0.2
kalrna
Control
Low 1−C
4463800
4464000
4464200
4464400
4464600
−log10(p)
15
10
5
0
kalrna
kalrna
si:ch73−83c10.2
4440000
4460000
4480000
4500000
chr 9

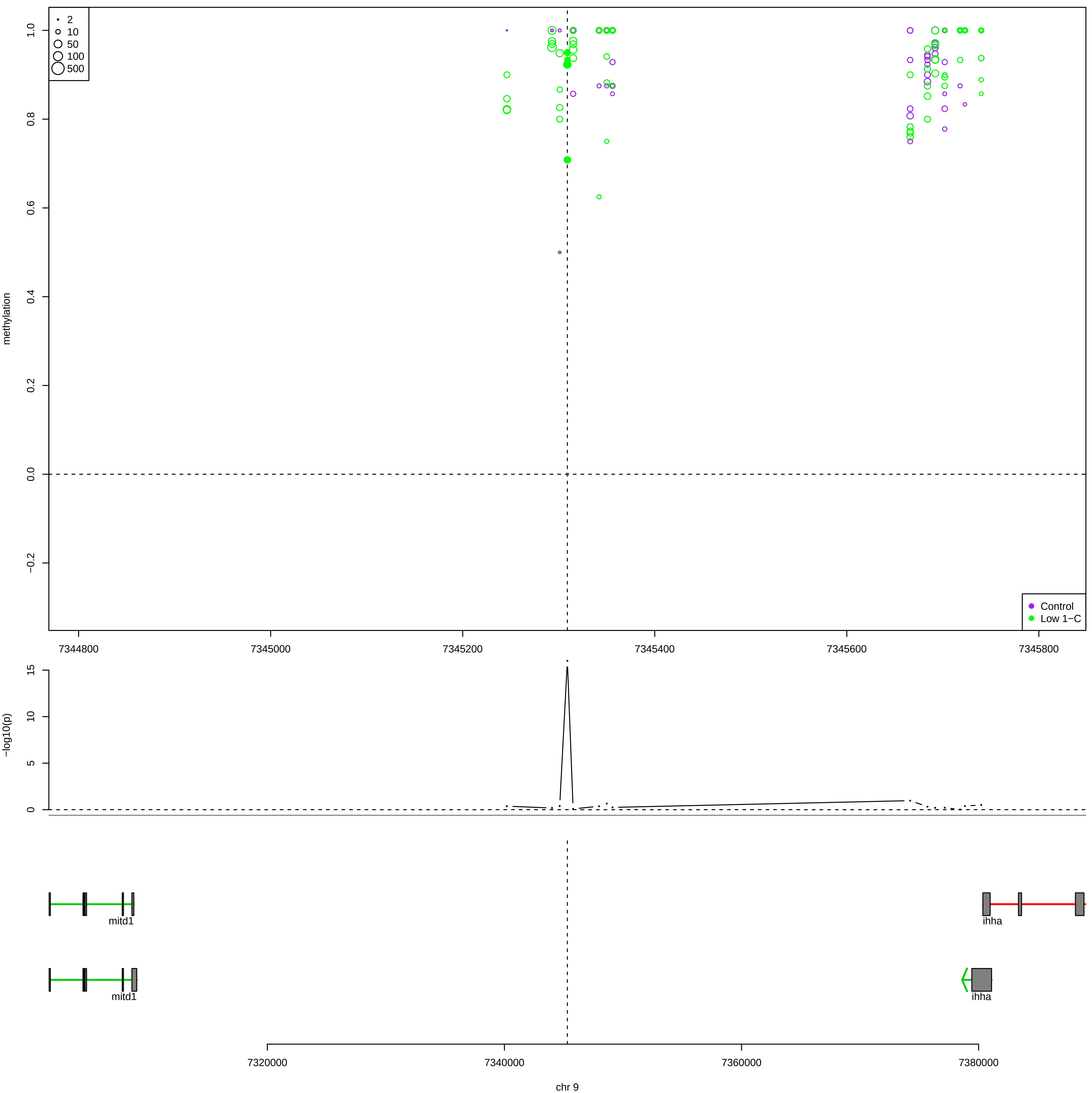
methylation
1.0
0.8
0.6
0.4
0.2
0.0
−0.2
2
10
50
100
500
Control
Low 1−C
7344800
7345000
7345200
7345400
7345600
7345800
−log10(p)
15
10
5
0
mitd1
mitd1
ihha
ihha
7320000
7340000
7360000
7380000
chr 9

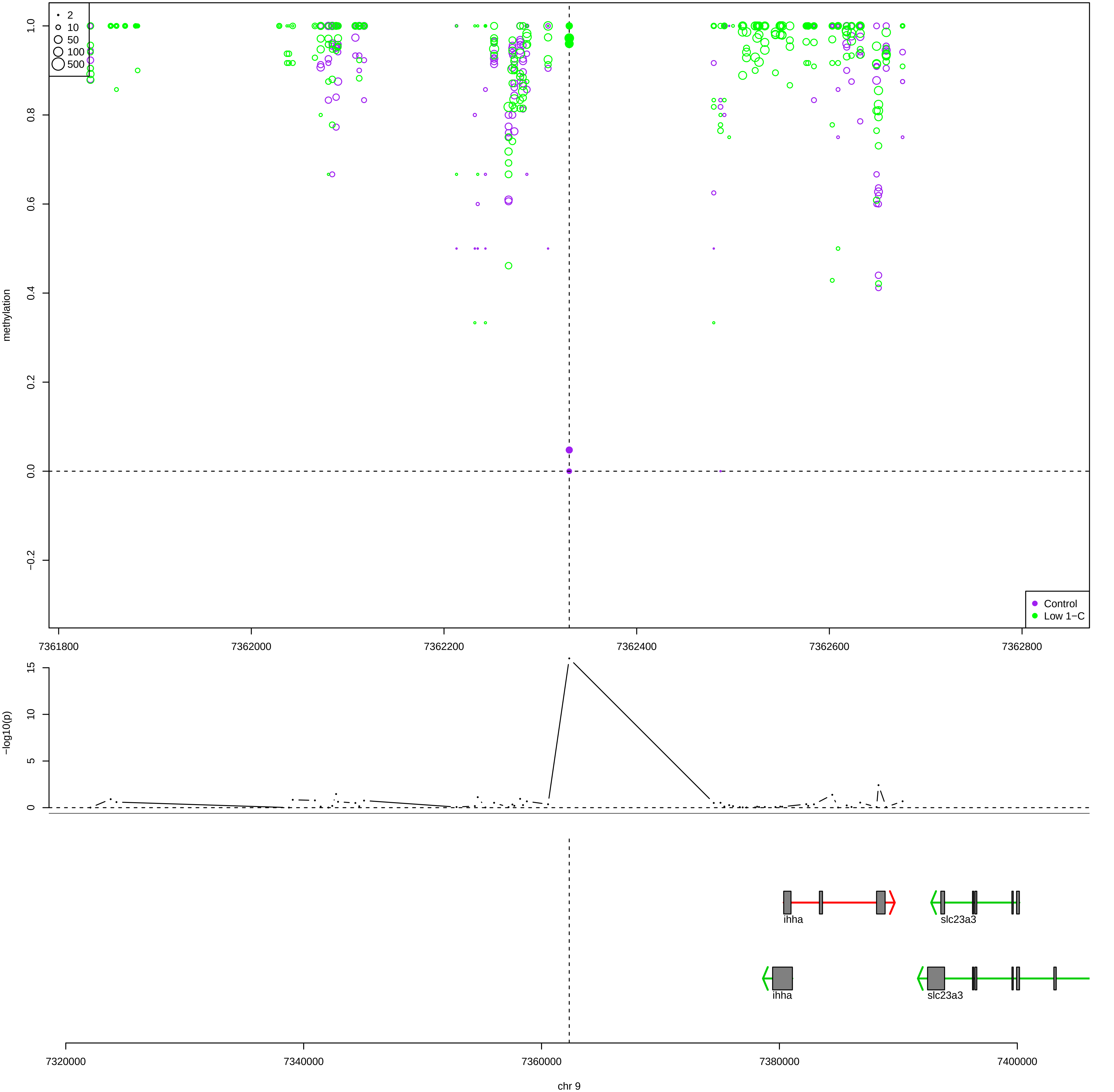

2
10
50
100
500
methylation
1.0
0.8
0.6
0.4
0.2
0.0
−0.2
Control
Low 1−C
7361800
7362000
7362200
7362400
7362600
7362800
−log10(p)
15
10
5
0
ihha
slc23a3
ihha
slc23a3
7320000
7340000
7360000
7380000
7400000
chr 9

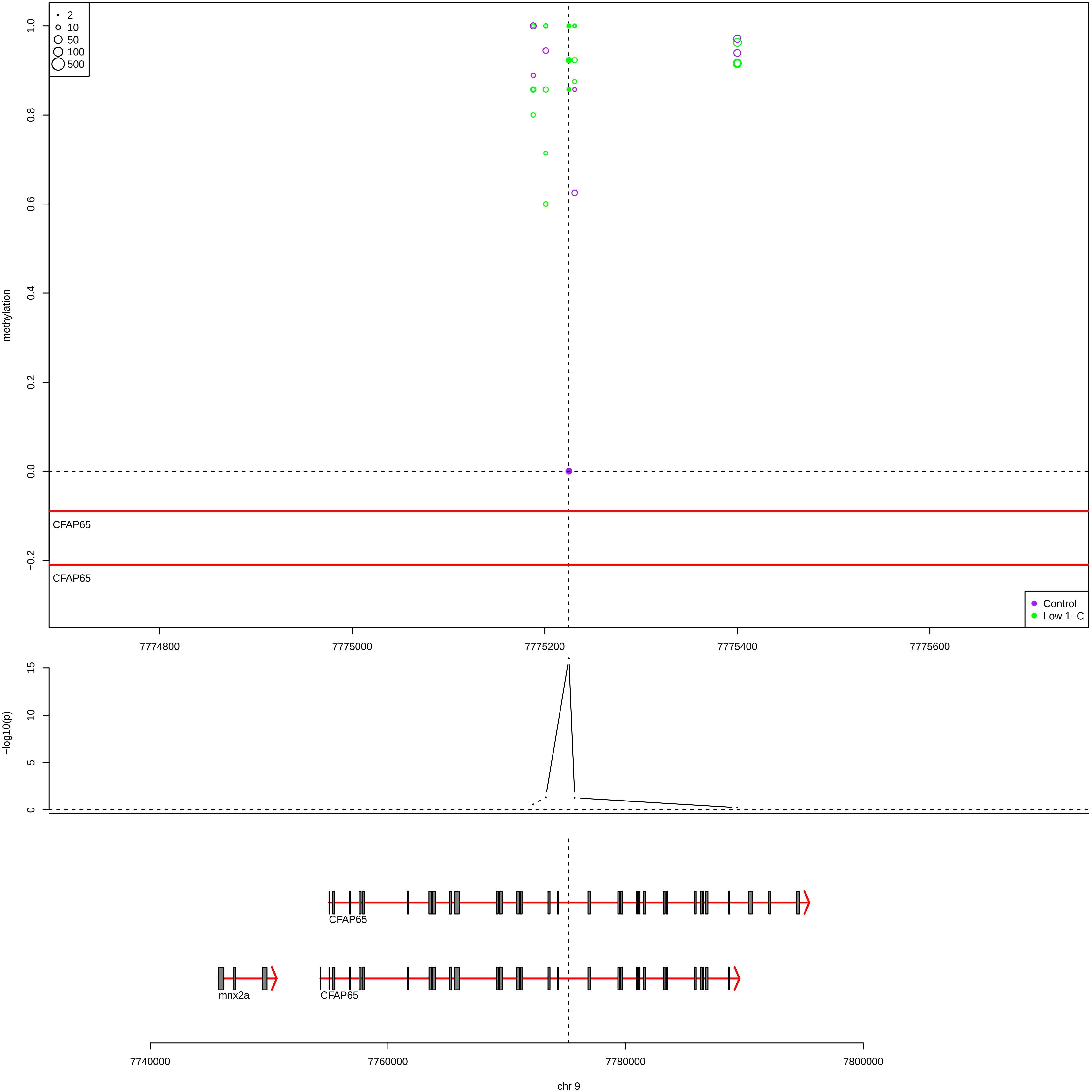

2
10
50
100
500
methylation
1.0
0.8
0.6
0.4
0.2
0.0
−0.2
CFAP65
CFAP65
Control
Low 1−C
7774800
7775000
7775200
7775400
7775600
−log10(p)
15
10
5
0
CFAP65
mnx2a
CFAP65
7740000
7760000
7780000
7800000
chr 9

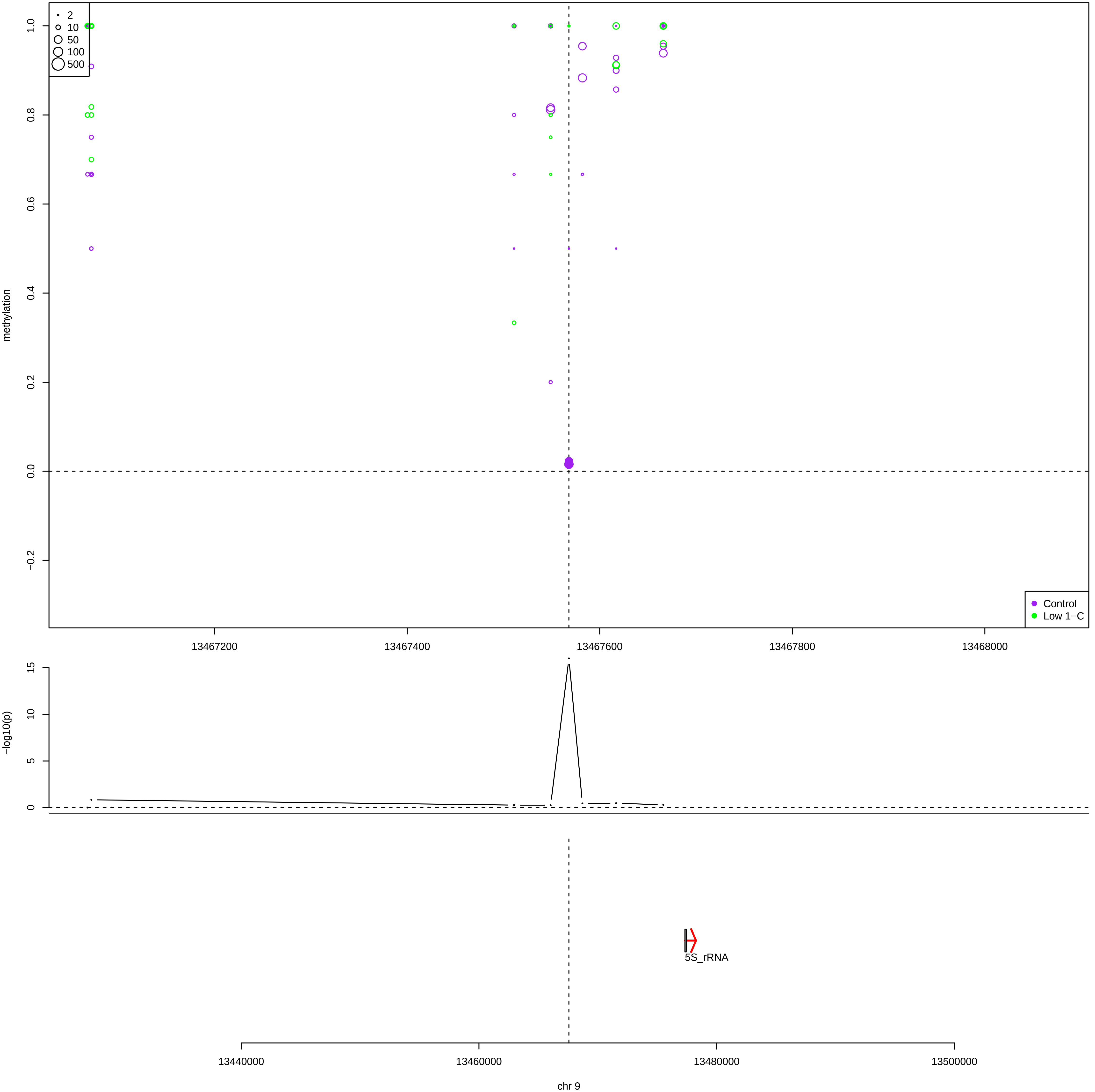

2
10
50
100
500
Control
Low 1−C
methylation
1.0
0.8
0.6
0.4
0.2
0.0
−0.2
13467200
13467400
13467600
13467800
13468000
−log10(p)
15
10
5
0
5S_rRNA
13440000
13460000
13480000
13500000
chr 9

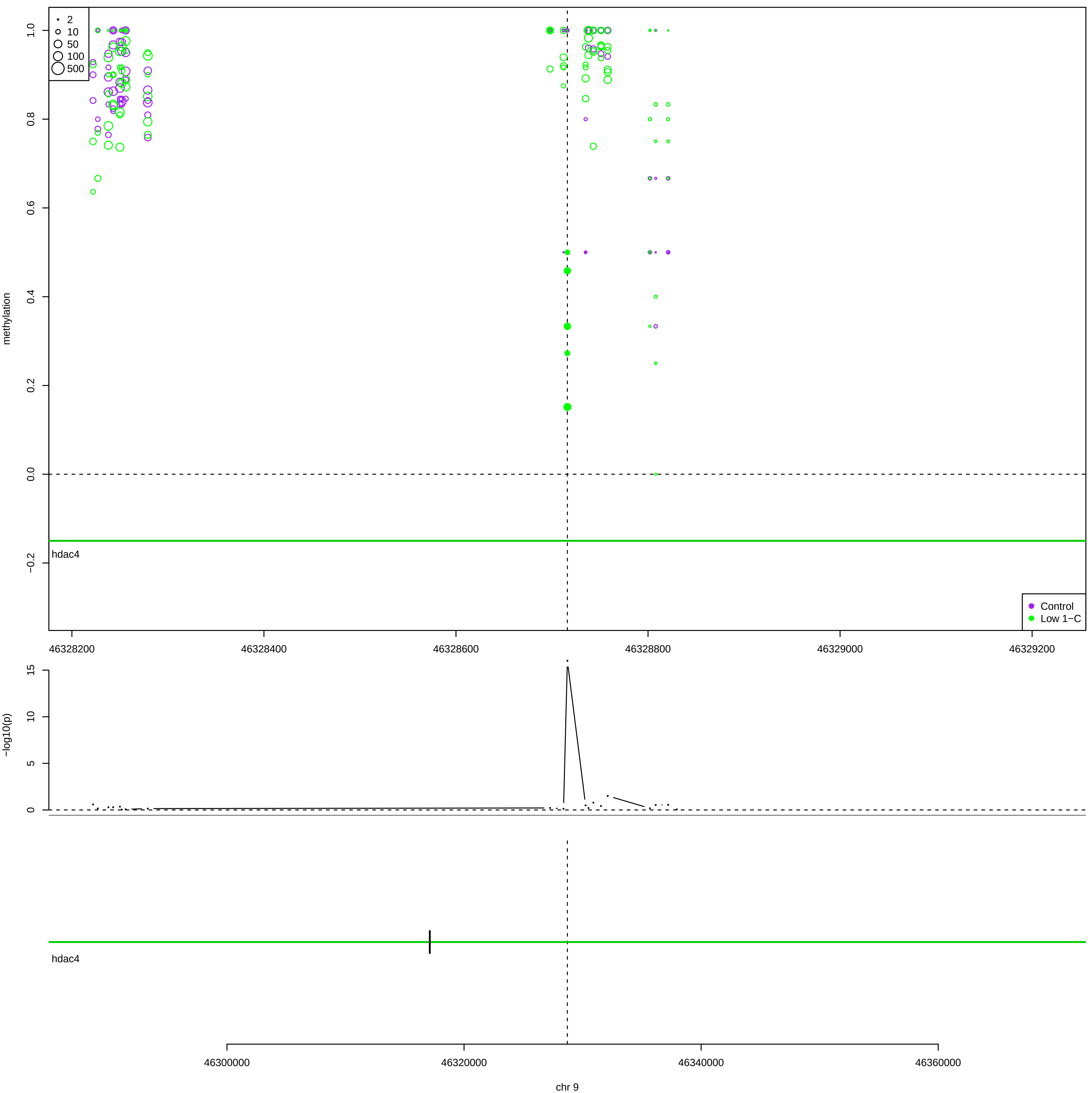

methylation
2
10
50
100
500
hdac4
Control
Low 1−C
46328200
46328400
46328600
46328800
46329000
46329200
−log10(p)
hdac4
46300000
46320000
46340000
46360000
chr 9

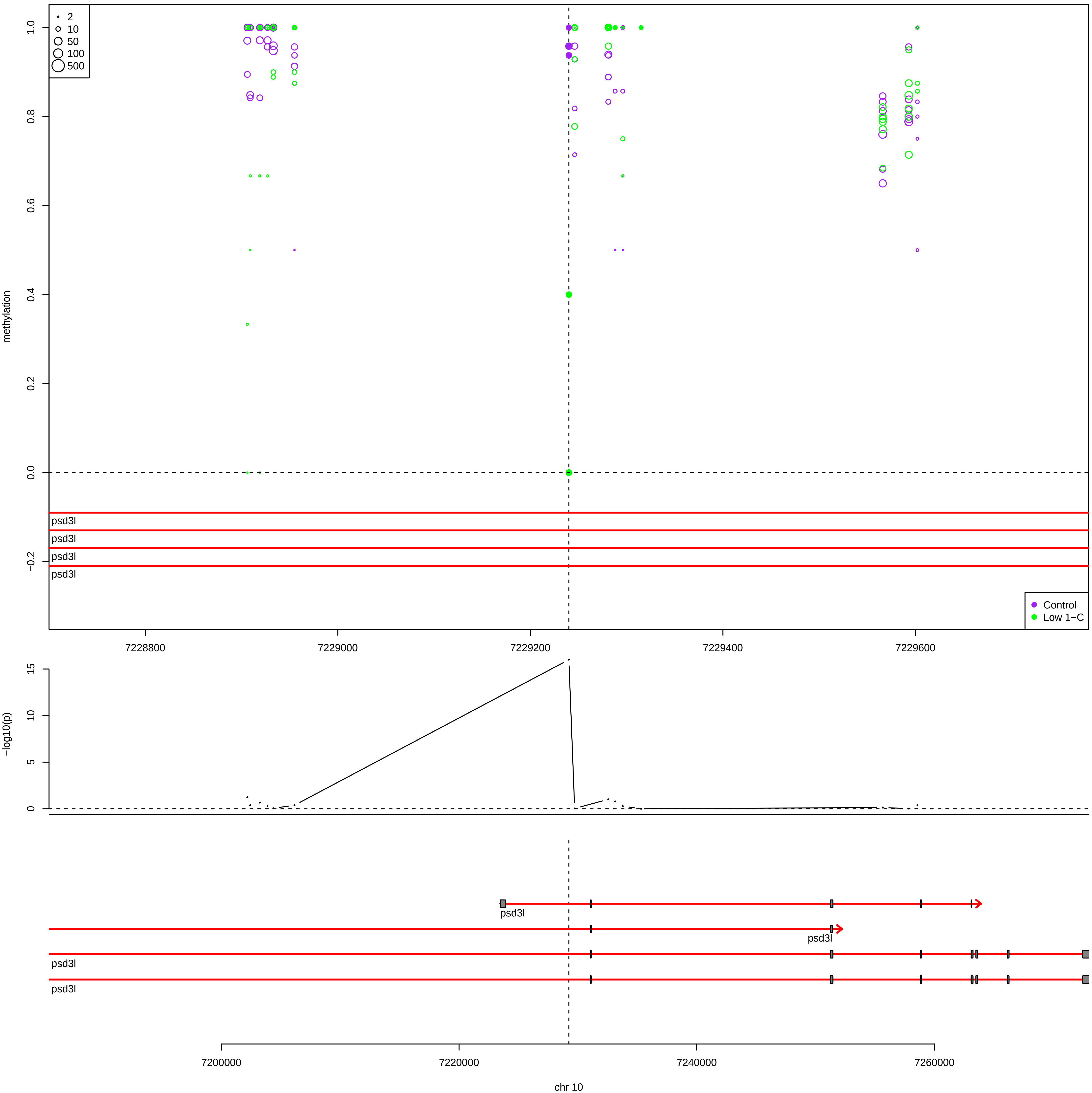

2
10
50
100
500
methylation
1.0
0.8
0.6
0.4
0.2
0.0
−0.2
psd3l
psd3l
psd3l
psd3l
Control
Low 1−C
7228800
7229000
7229200
7229400
7229600
−log10(p)
15
10
5
0
psd3l
psd3l
psd3l
psd3l
7200000
7220000
7240000
7260000
chr 10

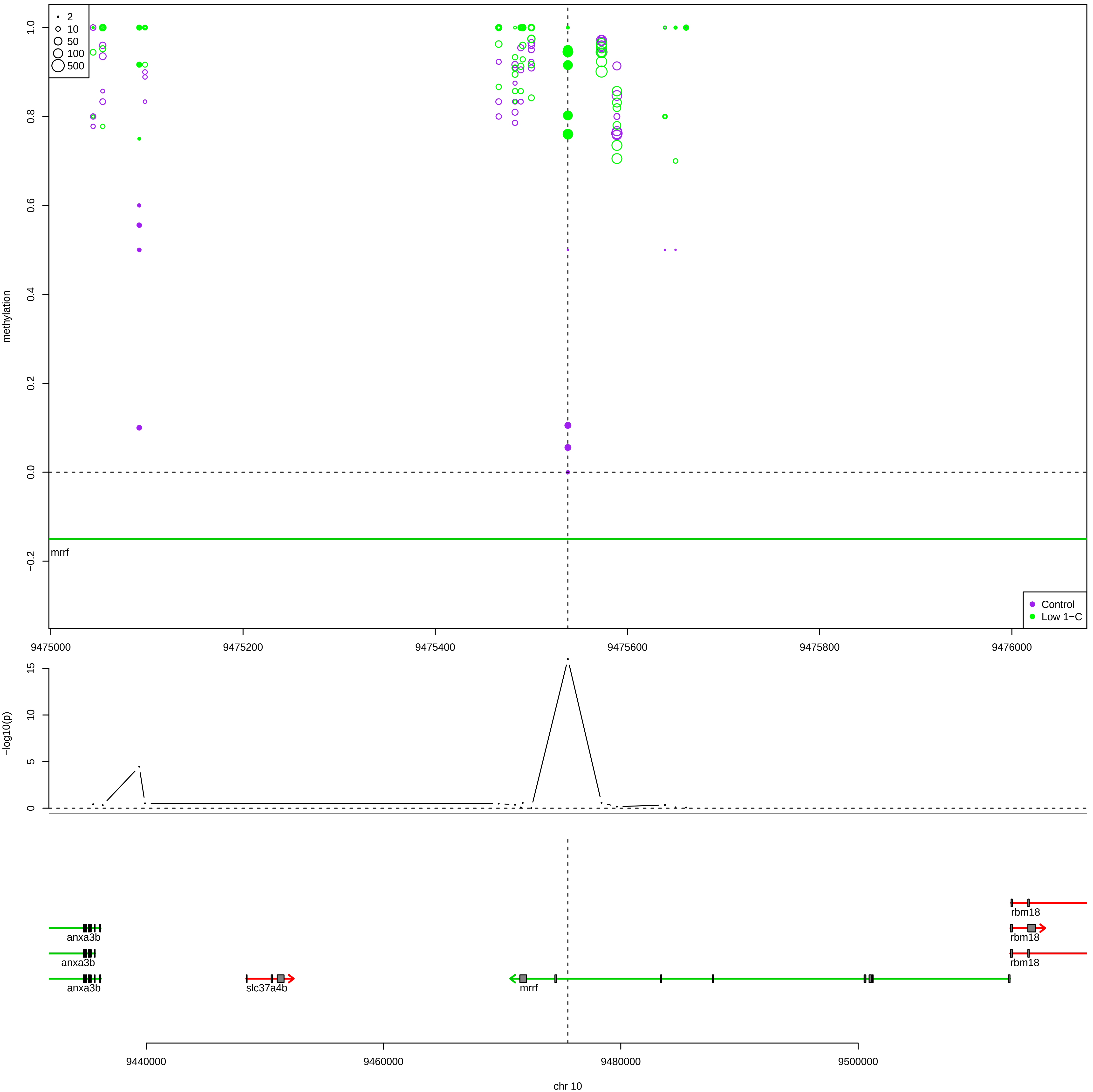

2
10
50
100
500
methylation
1.0
0.8
0.6
0.4
0.2
0.0
−0.2
mrrf
Control
Low 1−C
9475000
9475200
9475400
9475600
9475800
9476000
−log10(p)
15
10
5
0
rbm18
anxa3b
rbm18
anxa3b
rbm18
anxa3b
slc37a4b
mrrf
9440000
9460000
9480000
9500000
chr 10

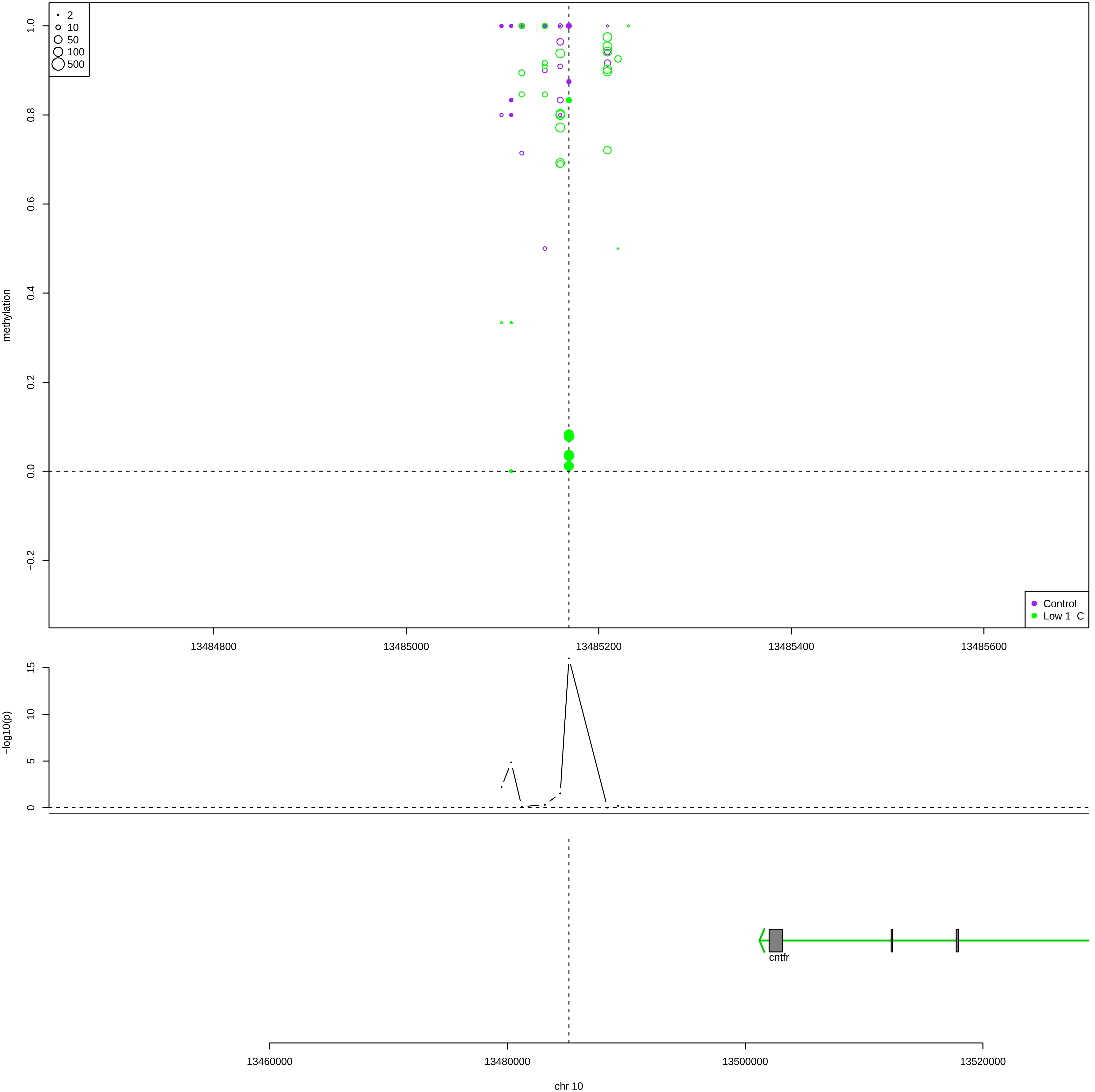

2
10
50
100
500
methylation
1.0
0.8
0.6
0.4
0.2
0.0
−0.2
Control
Low 1−C
13484800
13485000
13485200
13485400
13485600
−log10(p)
15
10
5
0
cntfr
13460000
13480000
13500000
13520000
chr 10

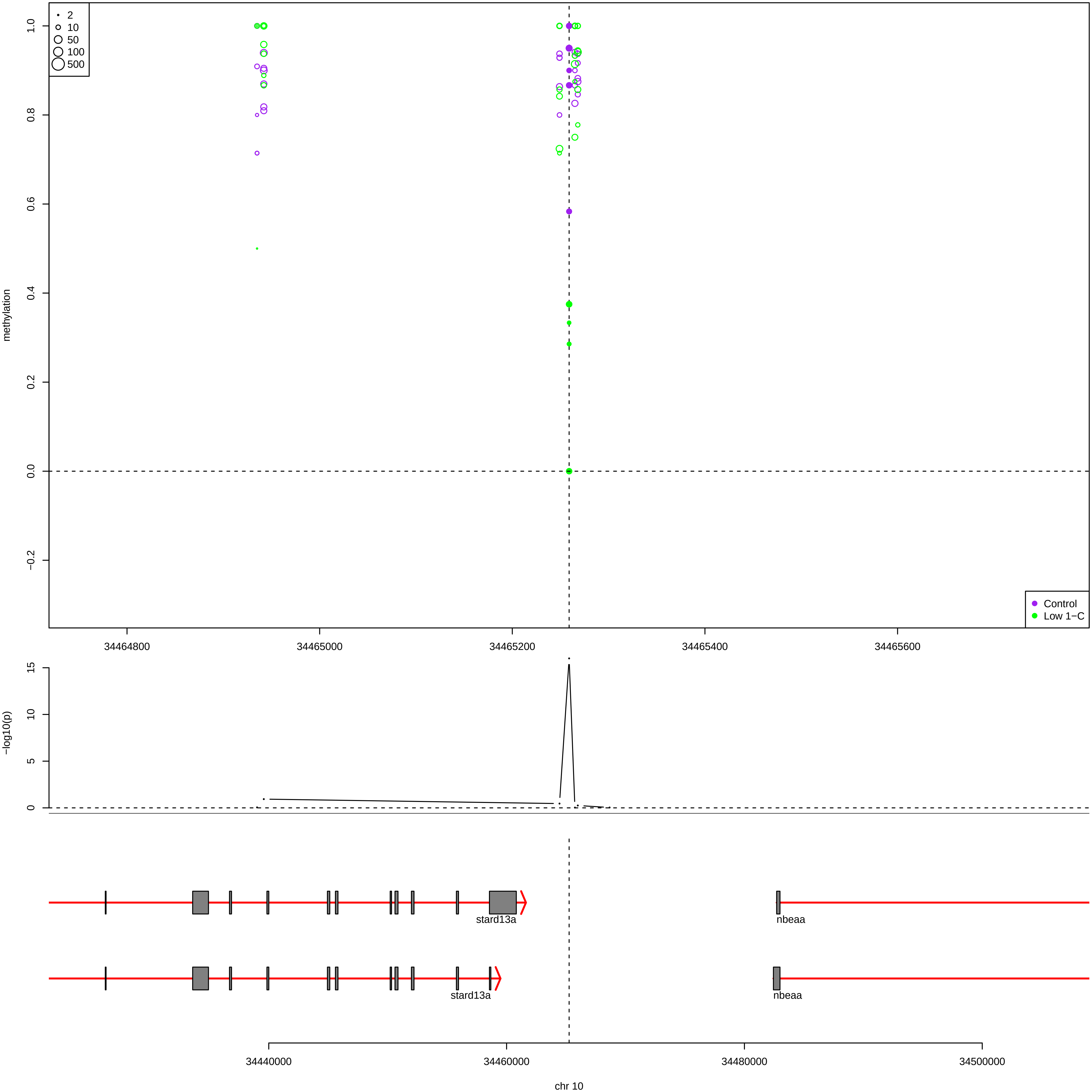

methylation
1.0
0.8
0.6
0.4
0.2
0.0
−0.2
2
10
50
100
500
Control
Low 1−C
34464800
34465000
34465200
34465400
34465600
−log10(p)
15
10
5
0
stard13a
nbeaa
stard13a
nbeaa
34440000
34460000
34480000
34500000
chr 10

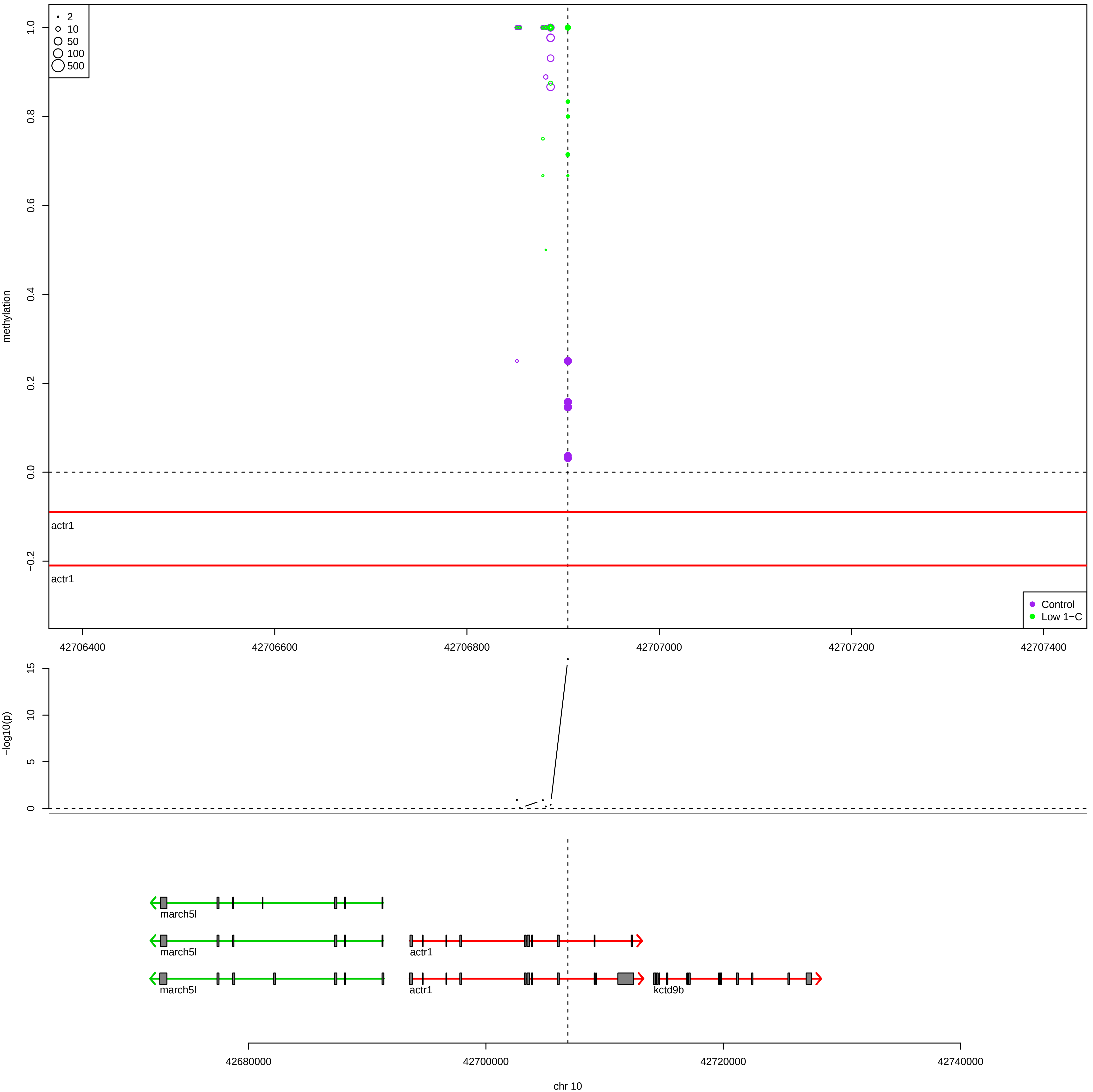

methylation
1.0
0.8
0.6
0.4
0.2
0.0
−0.2
2
10
50
100
500
actr1
actr1
Control
Low 1−C
42706400
42706600
42706800
42707000
42707200
42707400
−log10(p)
15
10
5
0
march5l
march5l
actr1
march5l
actr1
kctd9b
42680000
42700000
42720000
42740000
chr 10

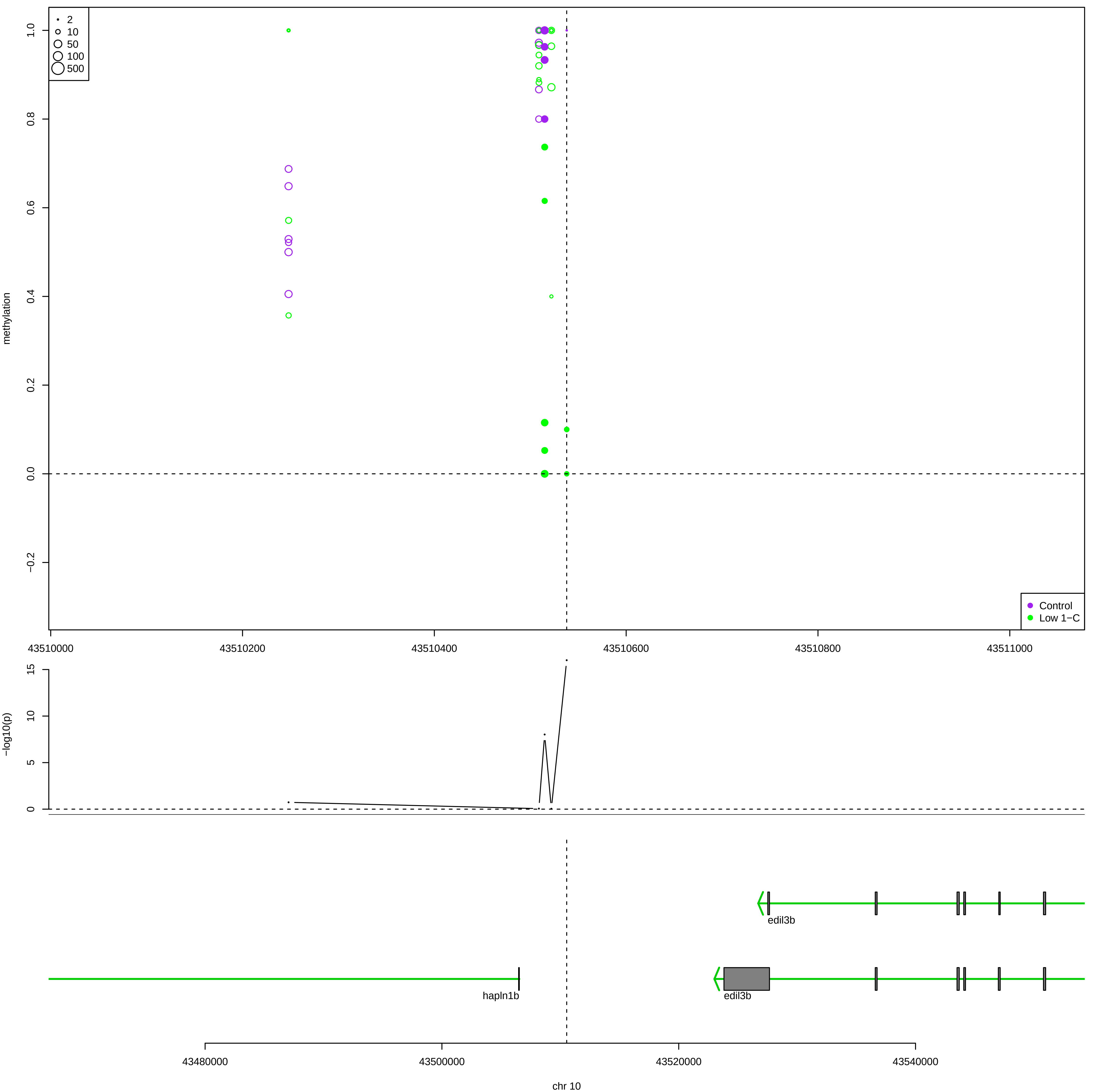

2
10
50
100
500
methylation
1.0
0.8
0.6
0.4
0.2
0.0
−0.2
Control
Low 1−C
43510000
43510200
43510400
43510600
43510800
43511000
−log10(p)
15
10
5
0
edil3b
hapln1b
edil3b
43480000
43500000
43520000
43540000
chr 10

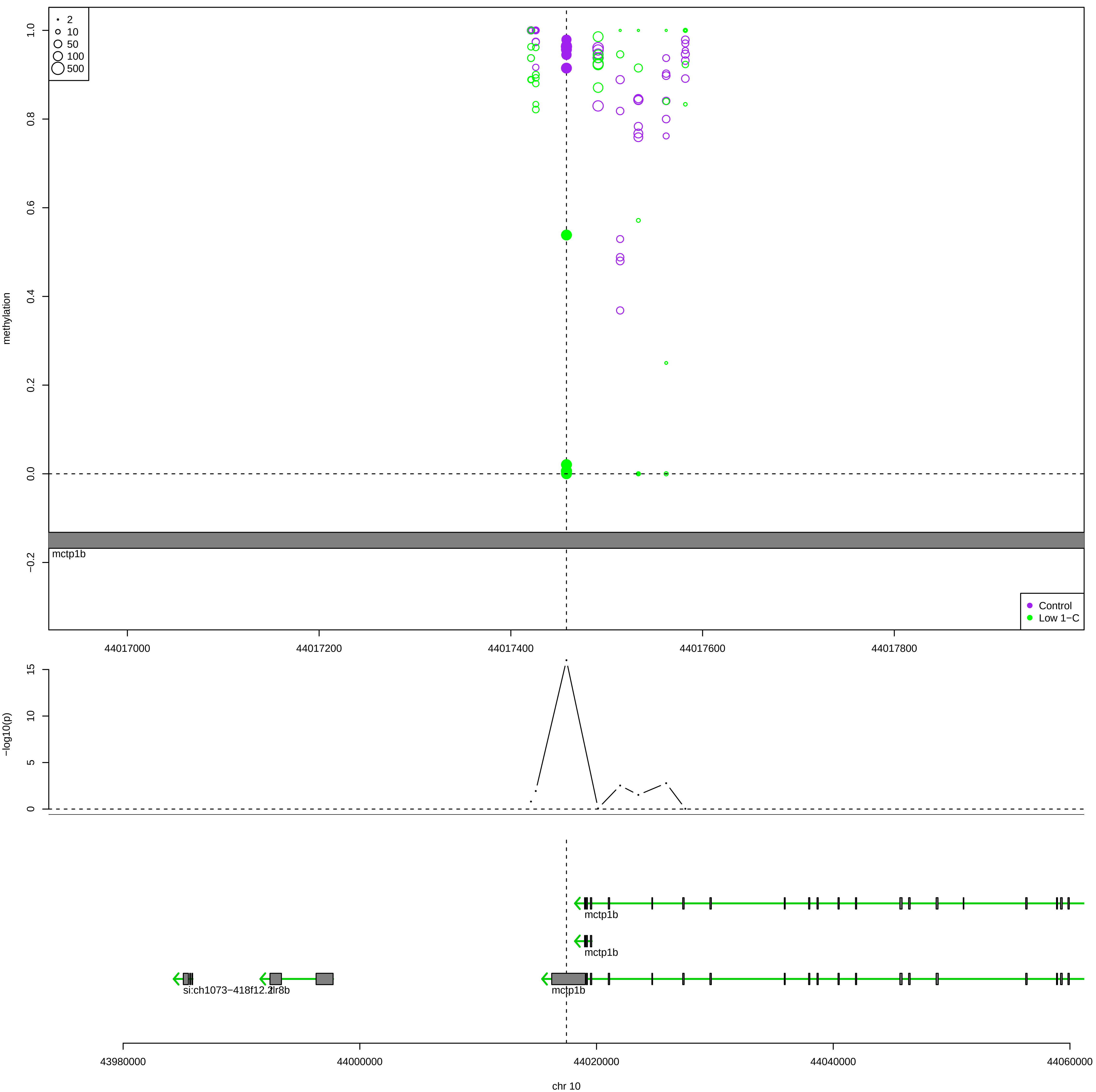
2
10
50
100
500
methylation
1.0
0.8
0.6
0.4
0.2
0.0
−0.2
mctp1b
Control
Low 1−C
44017000
44017200
44017400
44017600
44017800
−log10(p)
15
10
5
0
mctp1b
mctp1b
si:ch1073−418f12.2
lr8b
mctp1b
43980000
44000000
44020000
44040000
44060000
chr 10

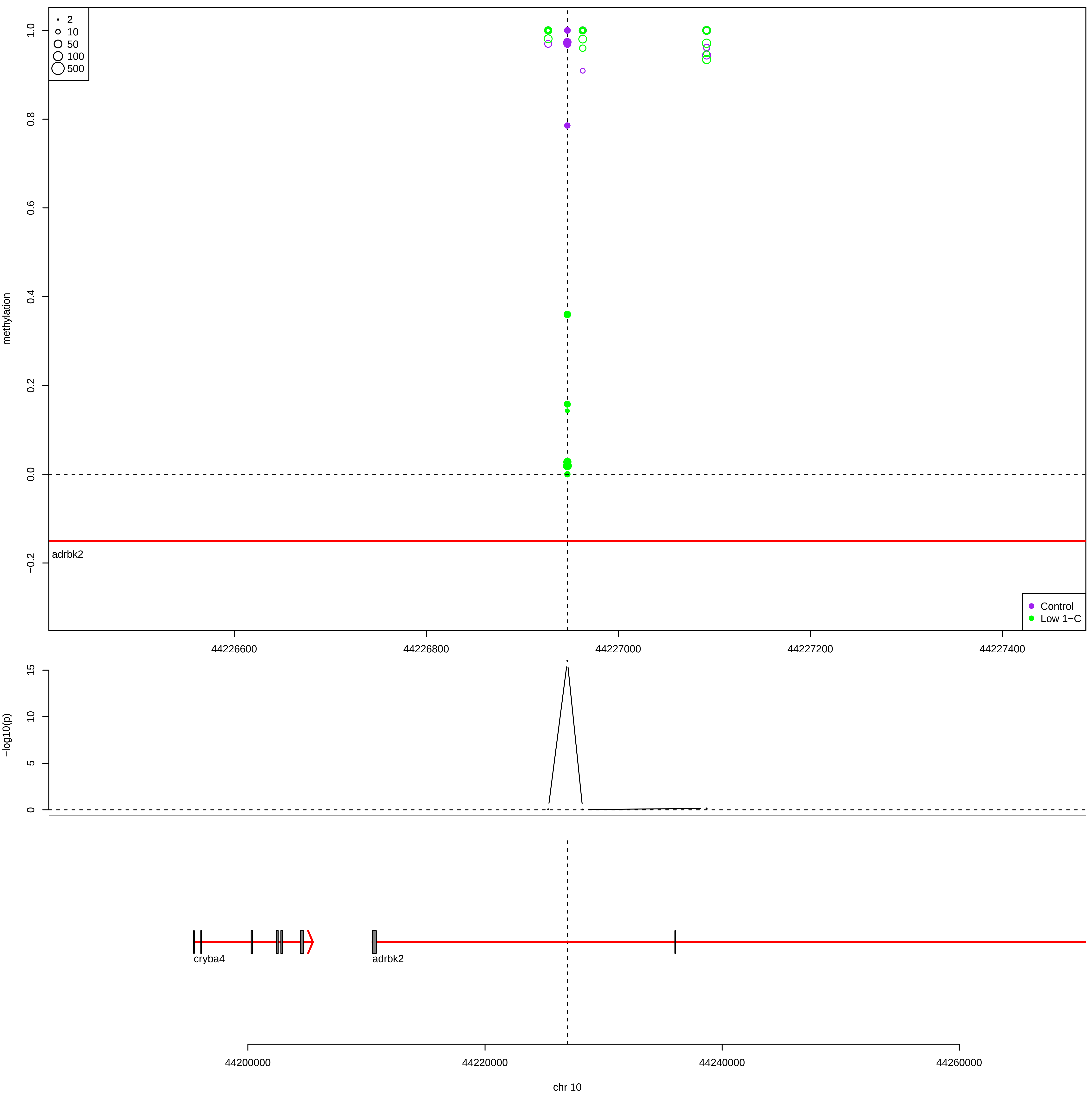

2
10
50
100
500
methylation
1.0
0.8
0.6
0.4
0.2
0.0
−0.2
adrbk2
Control
Low 1−C
44226600
44226800
44227000
44227200
44227400
−log10(p)
15
10
5
0
cryba4
adrbk2
44200000
44220000
44240000
44260000
chr 10

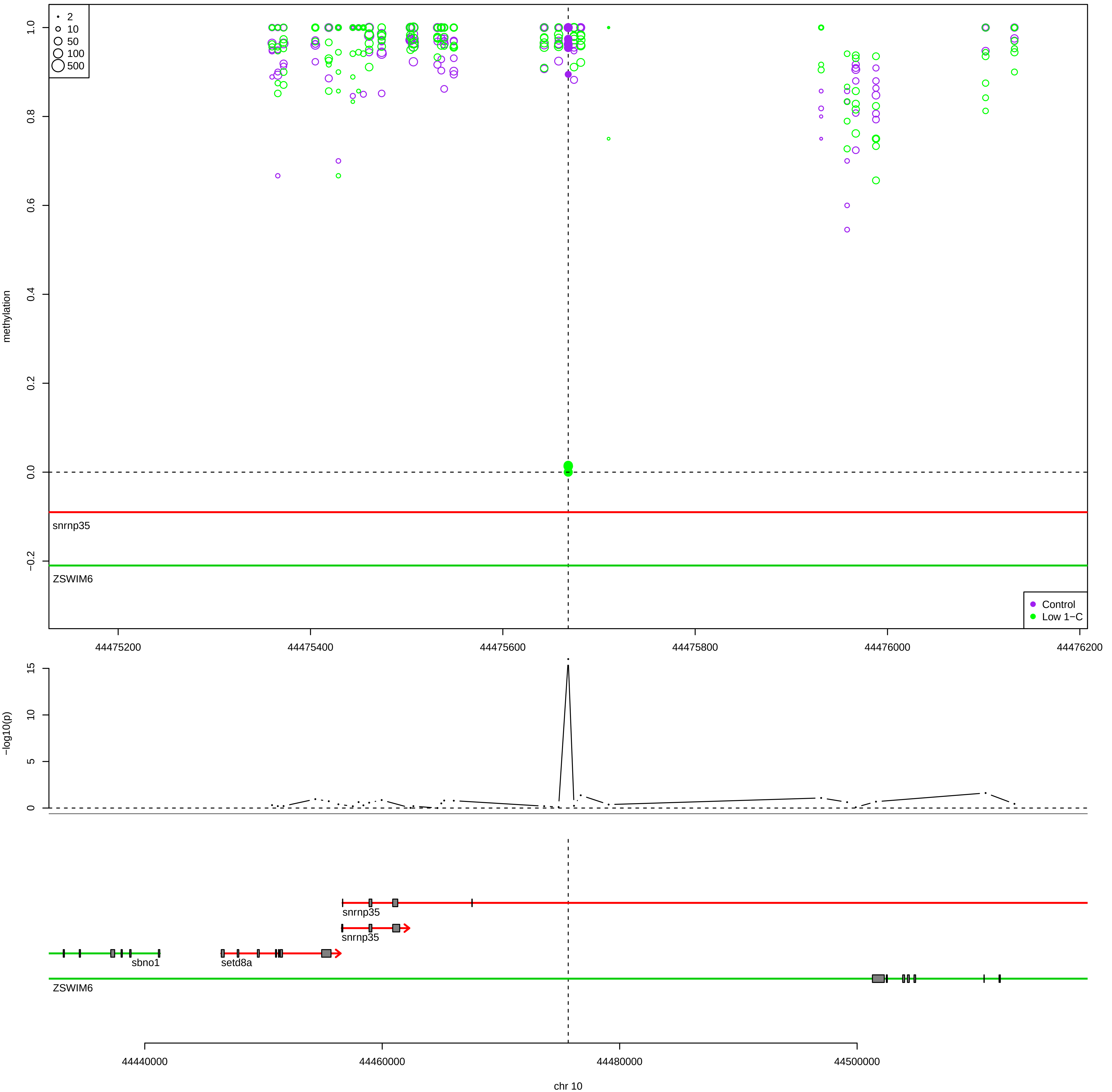

2
10
50
100
500
methylation
1.0
0.8
0.6
0.4
0.2
0.0
−0.2
snrnp35
ZSWIM6
Control
Low 1−C
44475200
44475400
44475600
44475800
44476000
44476200
−log10(p)
15
10
5
0
snrnp35
snrnp35
sbno1
setd8a
ZSWIM6
44440000
44460000
44480000
44500000
chr 10

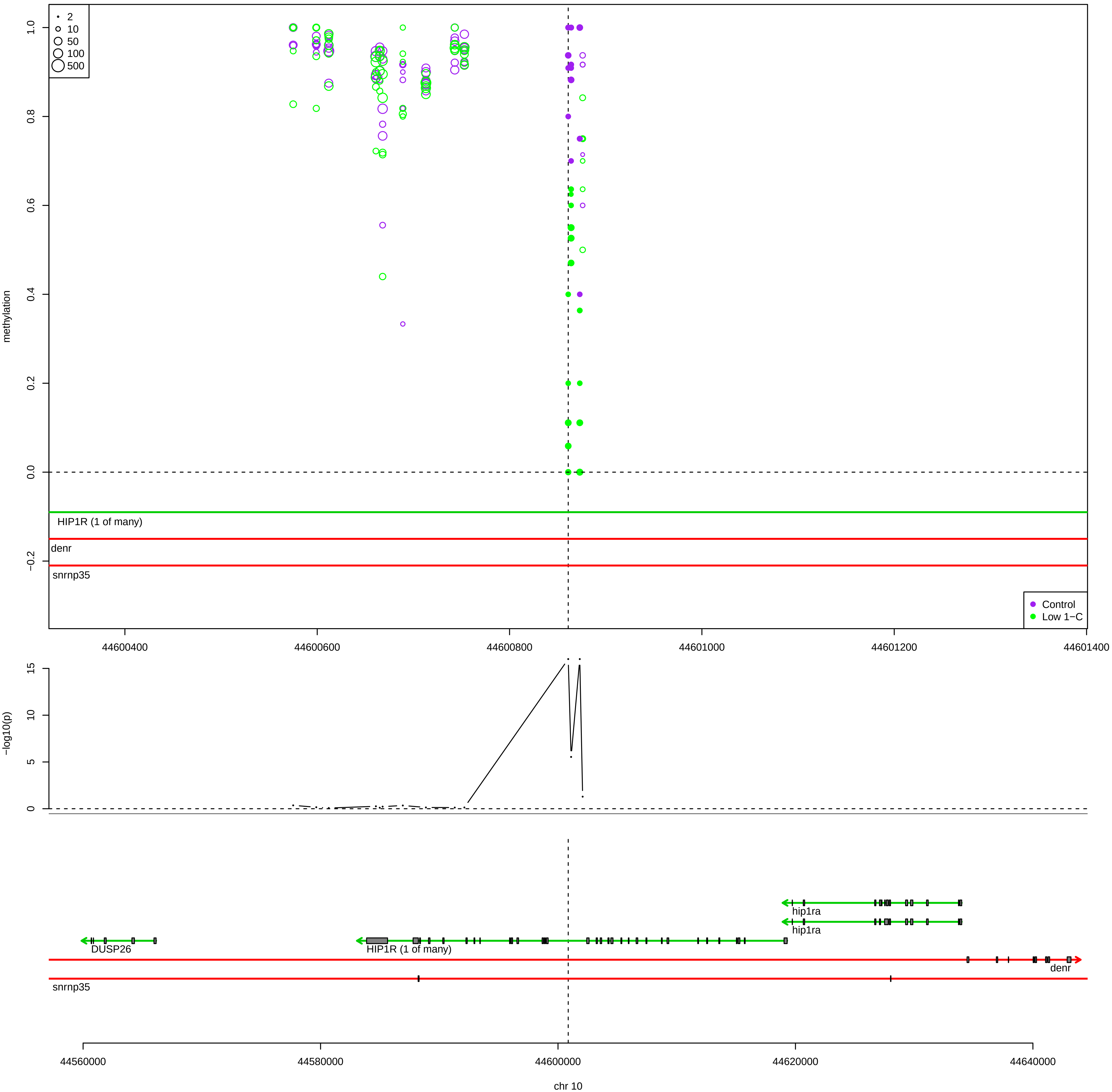

2
10
50
100
500
methylation
1.0
0.8
0.6
0.4
0.2
0.0
−0.2
HIP1R (1 of many)
denr
snrnp35
Control
Low 1−C
44600400
44600600
44600800
44601000
44601200
44601400
−log10(p)
15
10
5
0
hip1ra
hip1ra
DUSP26
HIP1R (1 of many)
denr
snrnp35
44560000
44580000
44600000
44620000
44640000
chr 10

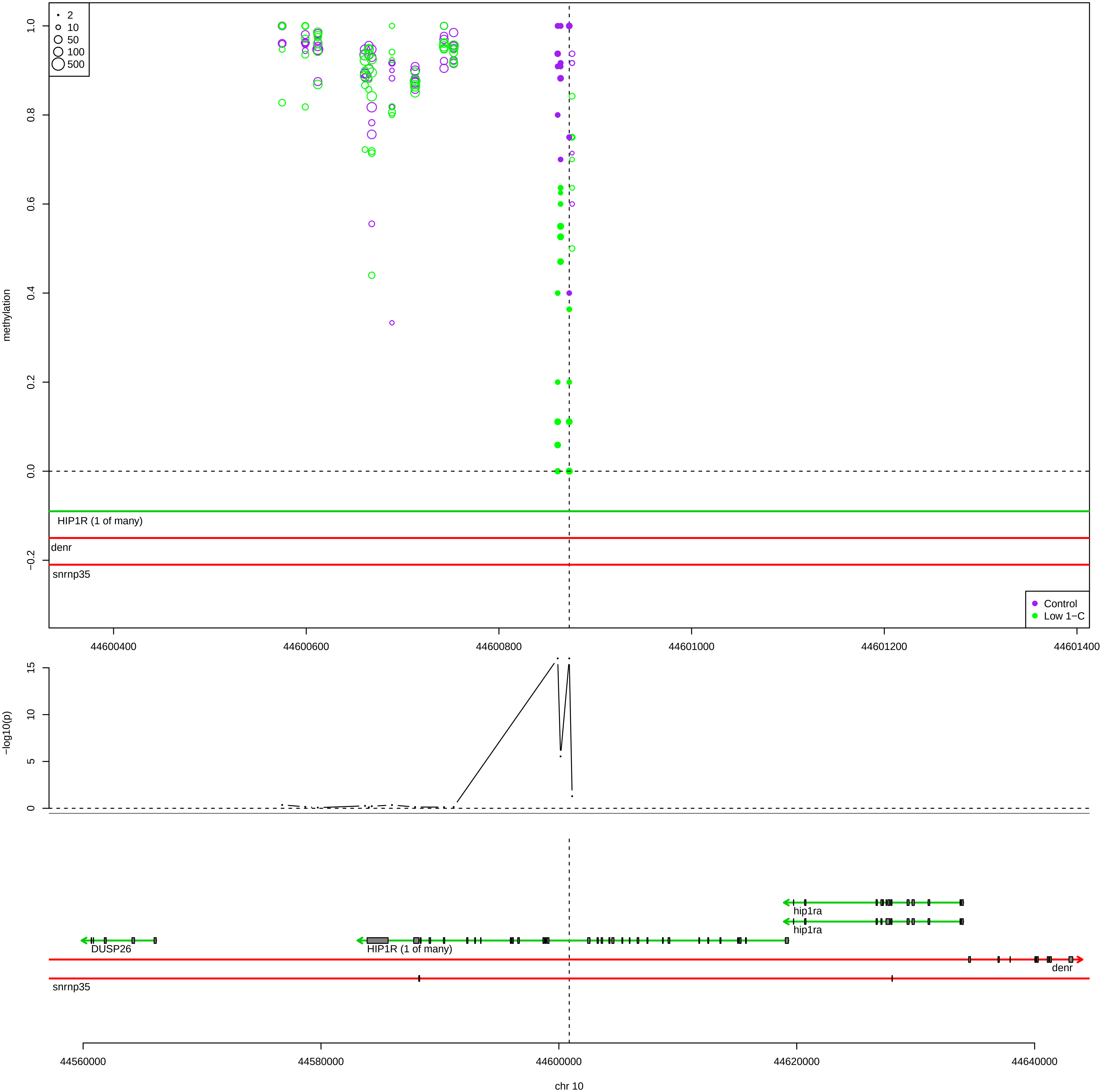

2
10
50
100
500
methylation
1.0
0.8
0.6
0.4
0.2
0.0
−0.2
HIP1R (1 of many)
denr
snrnp35
Control
Low 1−C
44600400
44600600
44600800
44601000
44601200
44601400
−log10(p)
15
10
5
0
hip1ra
hip1ra
DUSP26
HIP1R (1 of many)
denr
snrnp35
44560000
44580000
44600000
44620000
44640000
chr 10

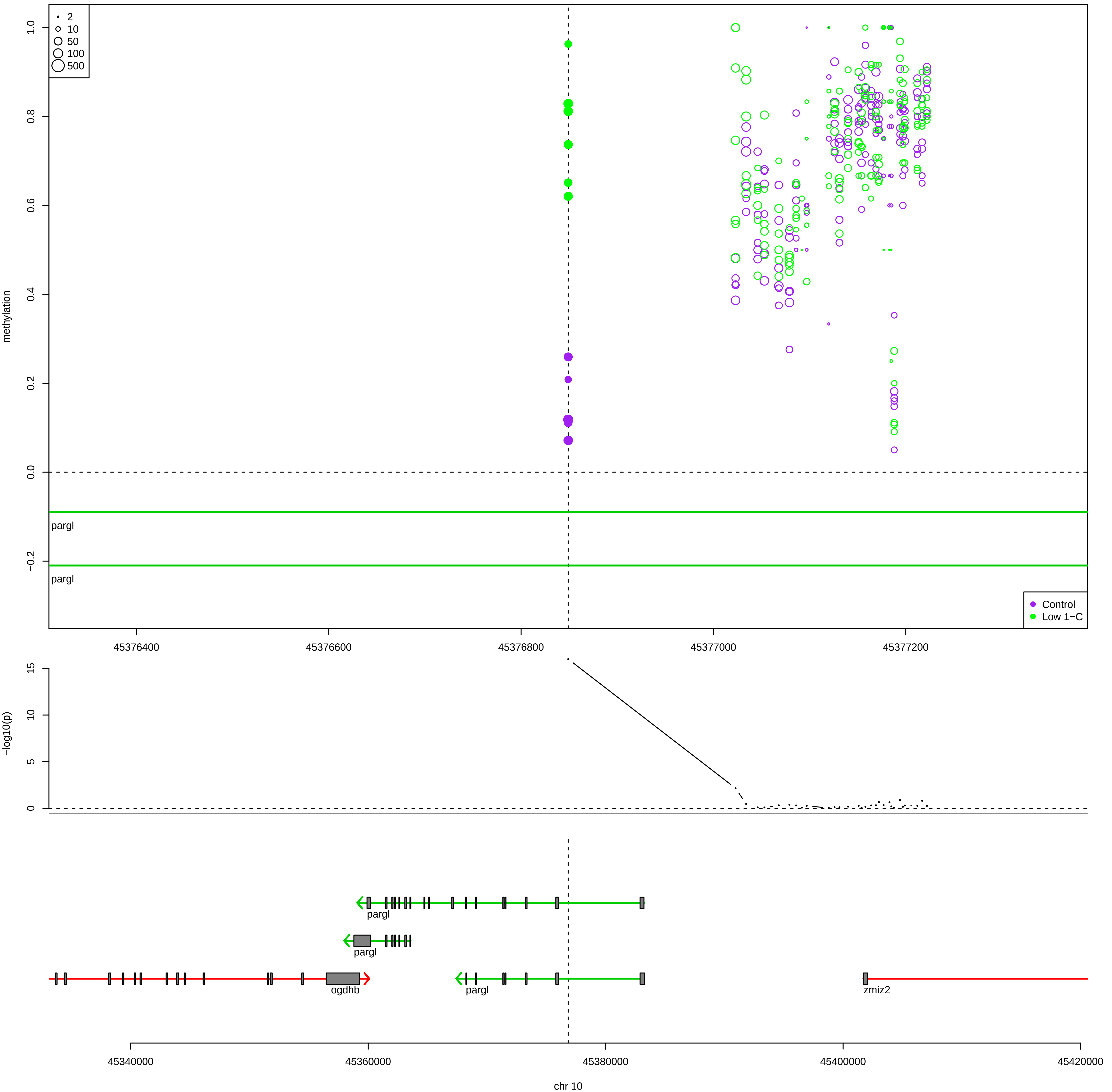

2
10
50
100
500
methylation
1.0
0.8
0.6
0.4
0.2
0.0
−0.2
pargl
pargl
Control
Low 1−C
45376400
45376600
45376800
45377000
45377200
−log10(p)
15
10
5
0
pargl
pargl
ogdhb
pargl
zmiz2
45340000
45360000
45380000
45400000
45420000
chr 10

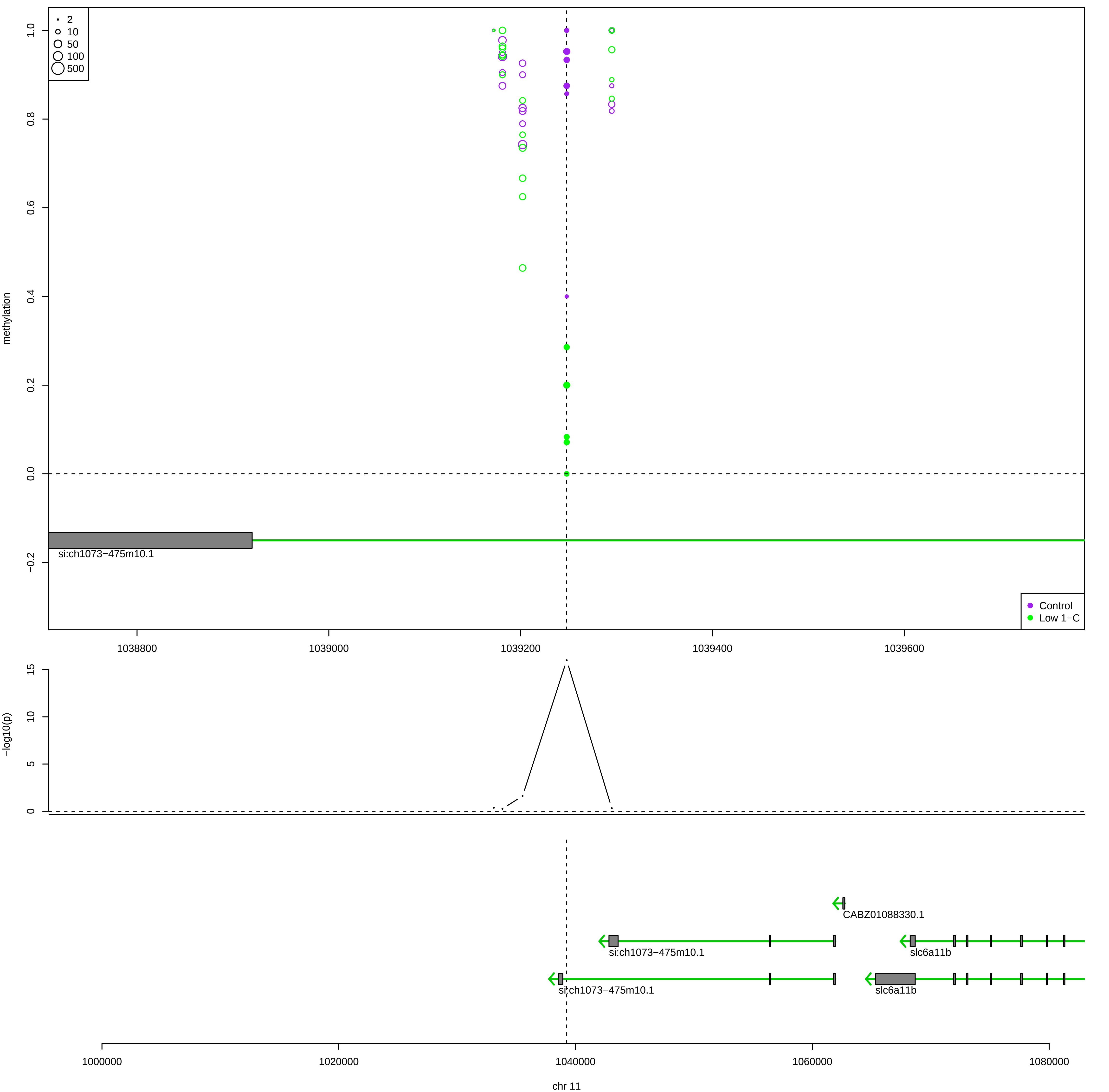

2
10
50
100
500
methylation
1.0
0.8
0.6
0.4
0.2
0.0
−0.2
si:ch1073−475m10.1
Control
Low 1−C
1038800
1039000
1039200
1039400
1039600
−log10(p)
15
10
5
0
CABZ01088330.1
si:ch1073−475m10.1
slc6a11b
si:ch1073−475m10.1
slc6a11b
1000000
1020000
1040000
1060000
1080000
chr 11

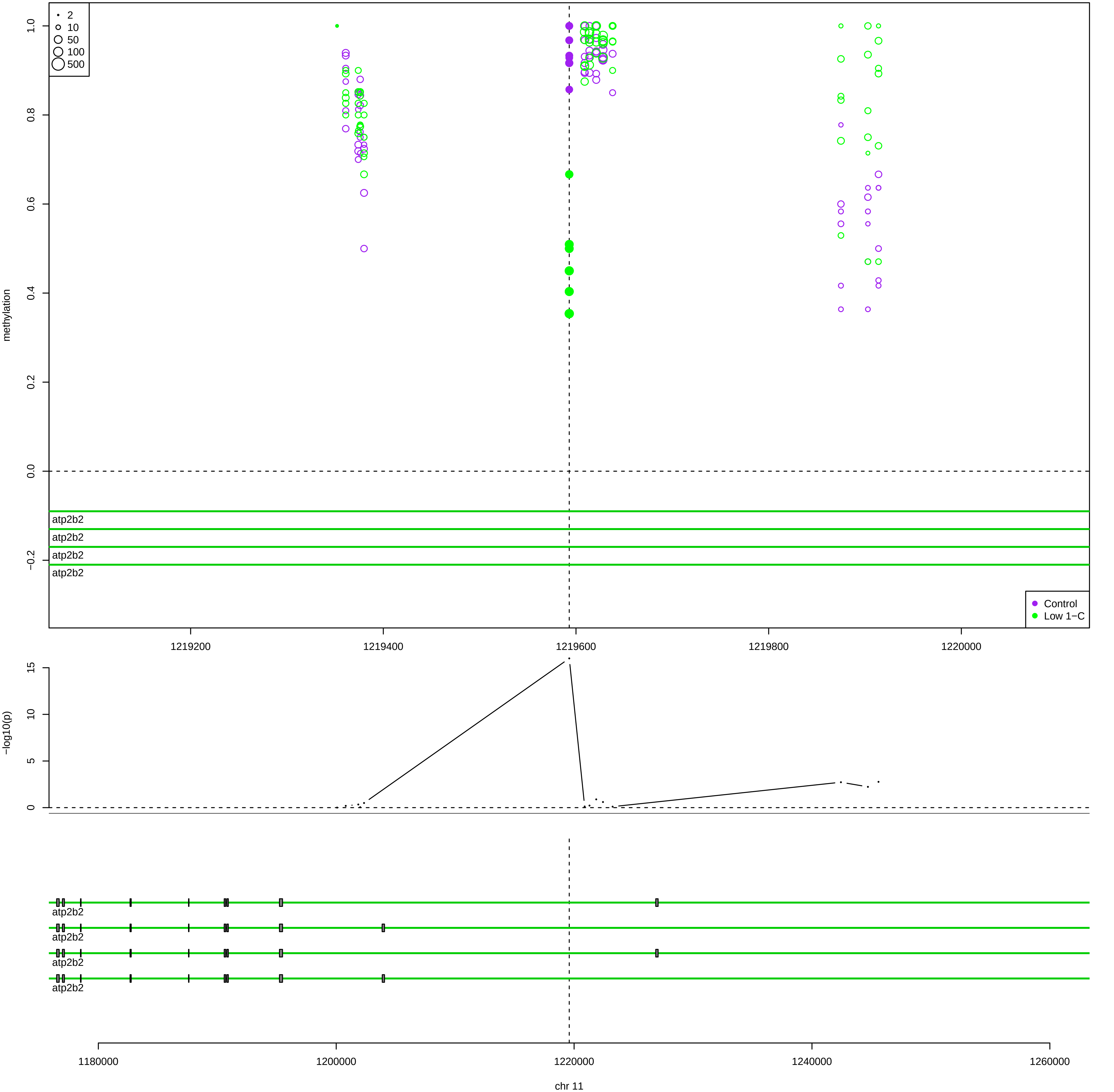
2
10
50
100
500
methylation
1.0
0.8
0.6
0.4
0.2
0.0
−0.2
atp2b2
atp2b2
atp2b2
atp2b2
Control
Low 1−C
1219200
1219400
1219600
1219800
1220000
−log10(p)
15
10
5
0
atp2b2
atp2b2
atp2b2
atp2b2
1180000
1200000
1220000
1240000
1260000
chr 11

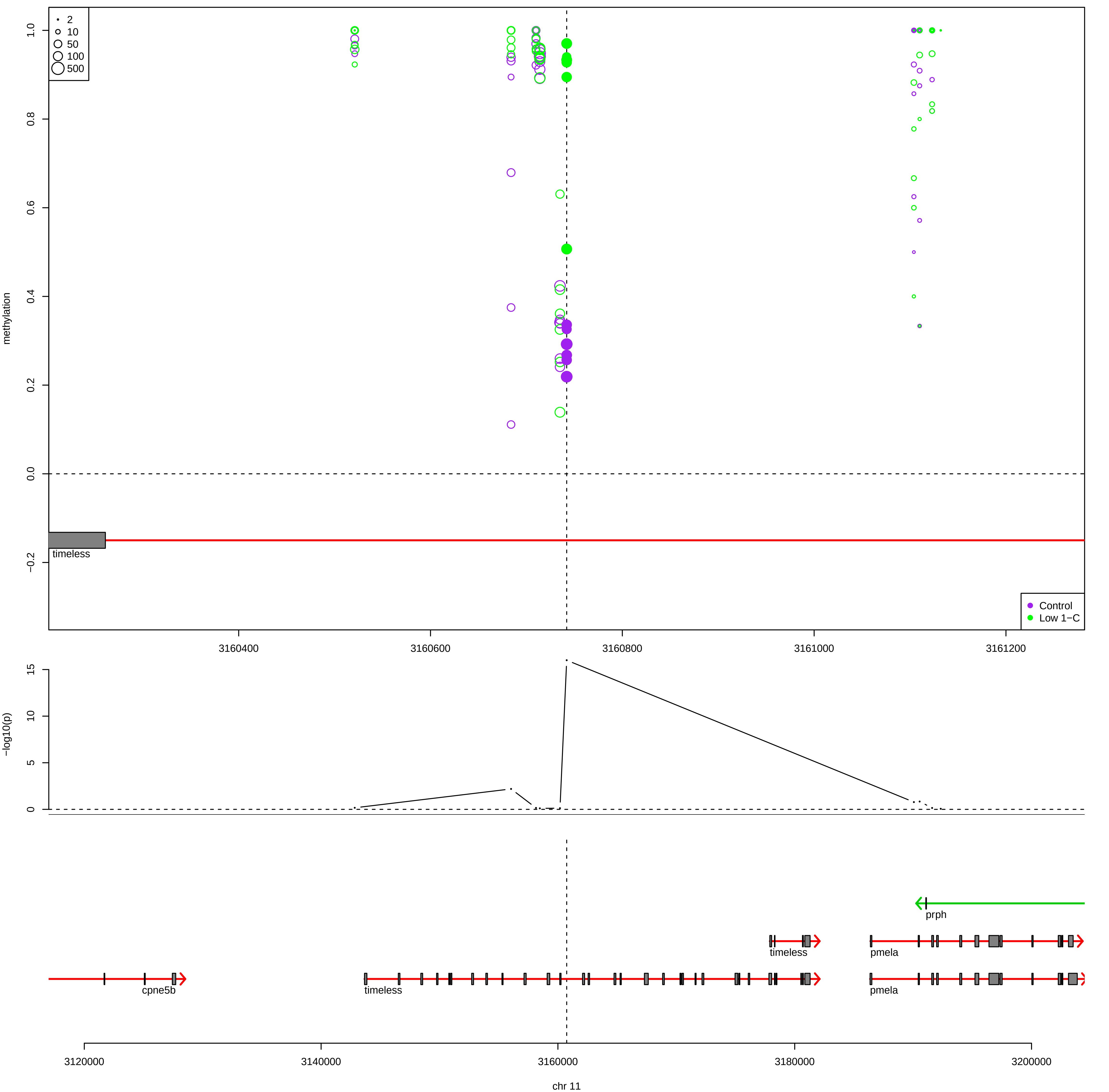

2
10
50
100
500
methylation
1.0
0.8
0.6
0.4
0.2
0.0
−0.2
timeless
Control
Low 1−C
3160400
3160600
3160800
3161000
3161200
−log10(p)
15
10
5
0
prph
timeless
pmela
cpne5b
timeless
pmela
3120000
3140000
3160000
3180000
3200000
chr 11

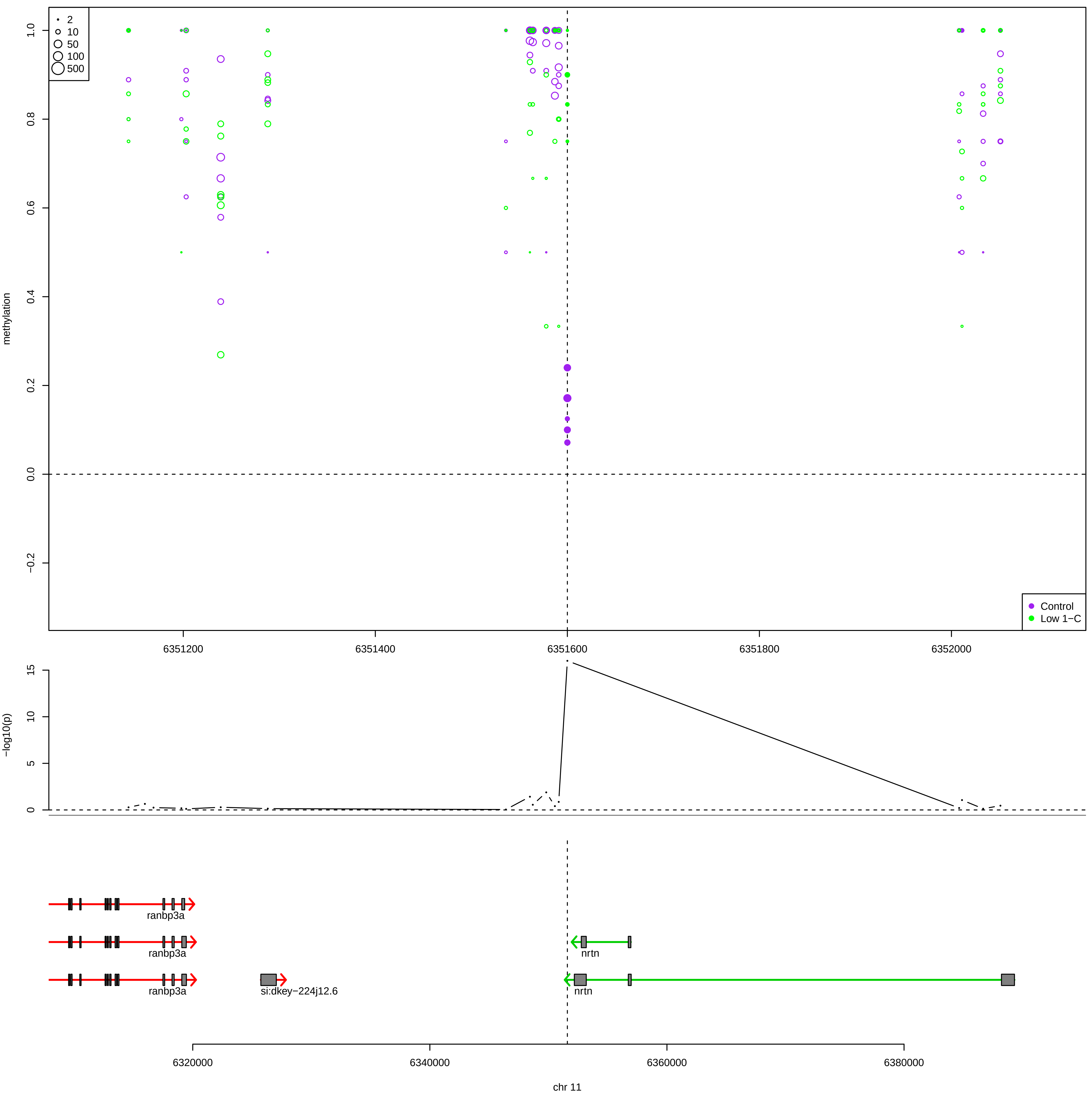

2
10
50
100
500
methylation
1.0
0.8
0.6
0.4
0.2
0.0
-0.2
Control
Low 1−C
6351200
6351400
6351600
6351800
6352000
-log10(p)
15
10
5
0
ranbp3a
ranbp3a
ranbp3a
si:dkey−224j12.6
nrtn
nrtn
6320000
6340000
6360000
6380000
chr 11

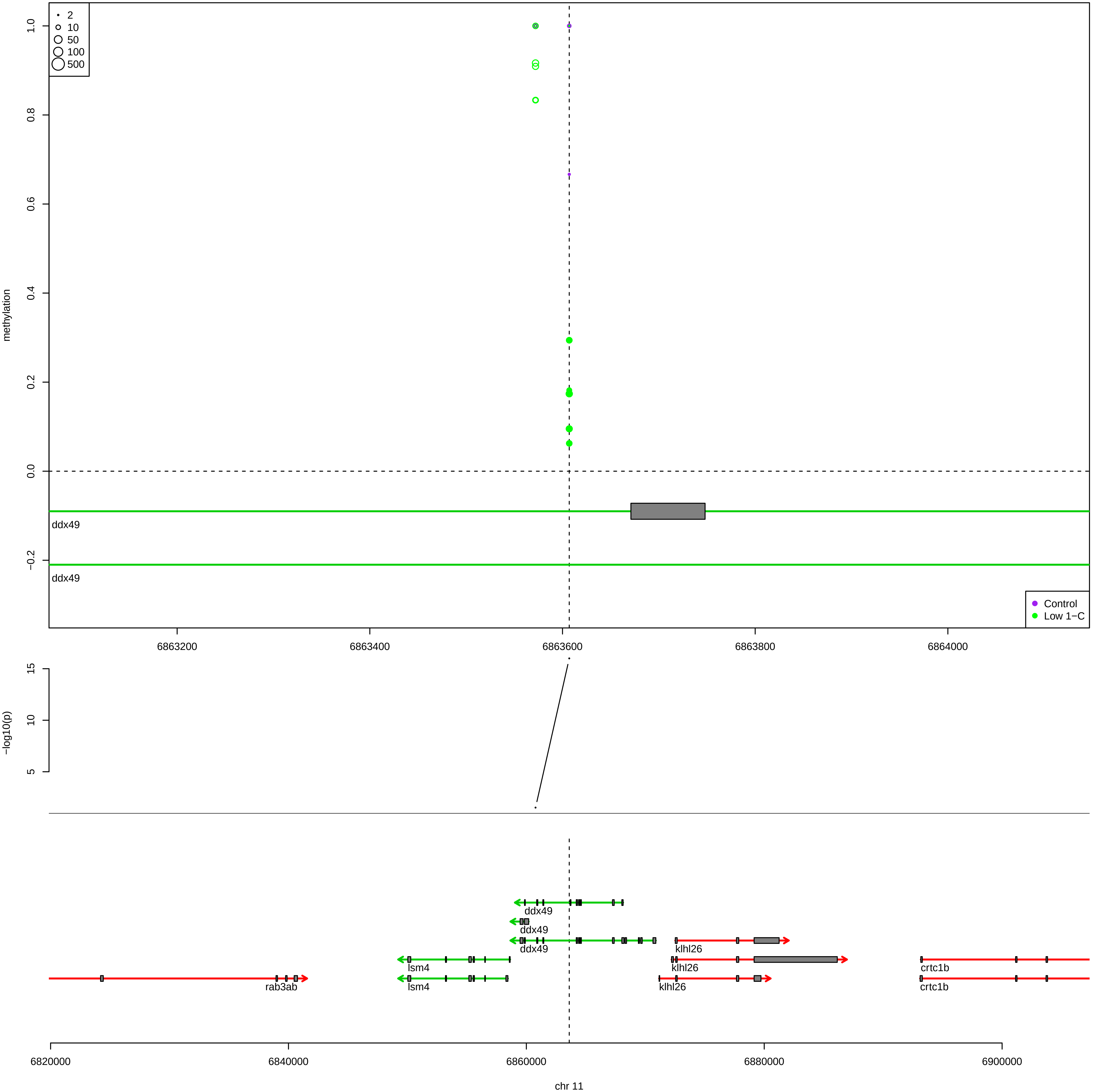

methylation
1.0
0.8
0.6
0.4
0.2
0.0
−0.2
2
10
50
100
500
ddx49
ddx49
Control
Low 1−C
6863200
6863400
6863600
6863800
6864000
−log10(p)
15
10
5
ddx49
ddx49
ddx49
klhl26
klhl26
klhl26
lsm4
lsm4
rab3ab
crtc1b
crtc1b
6820000
6840000
6860000
6880000
6900000
chr 11

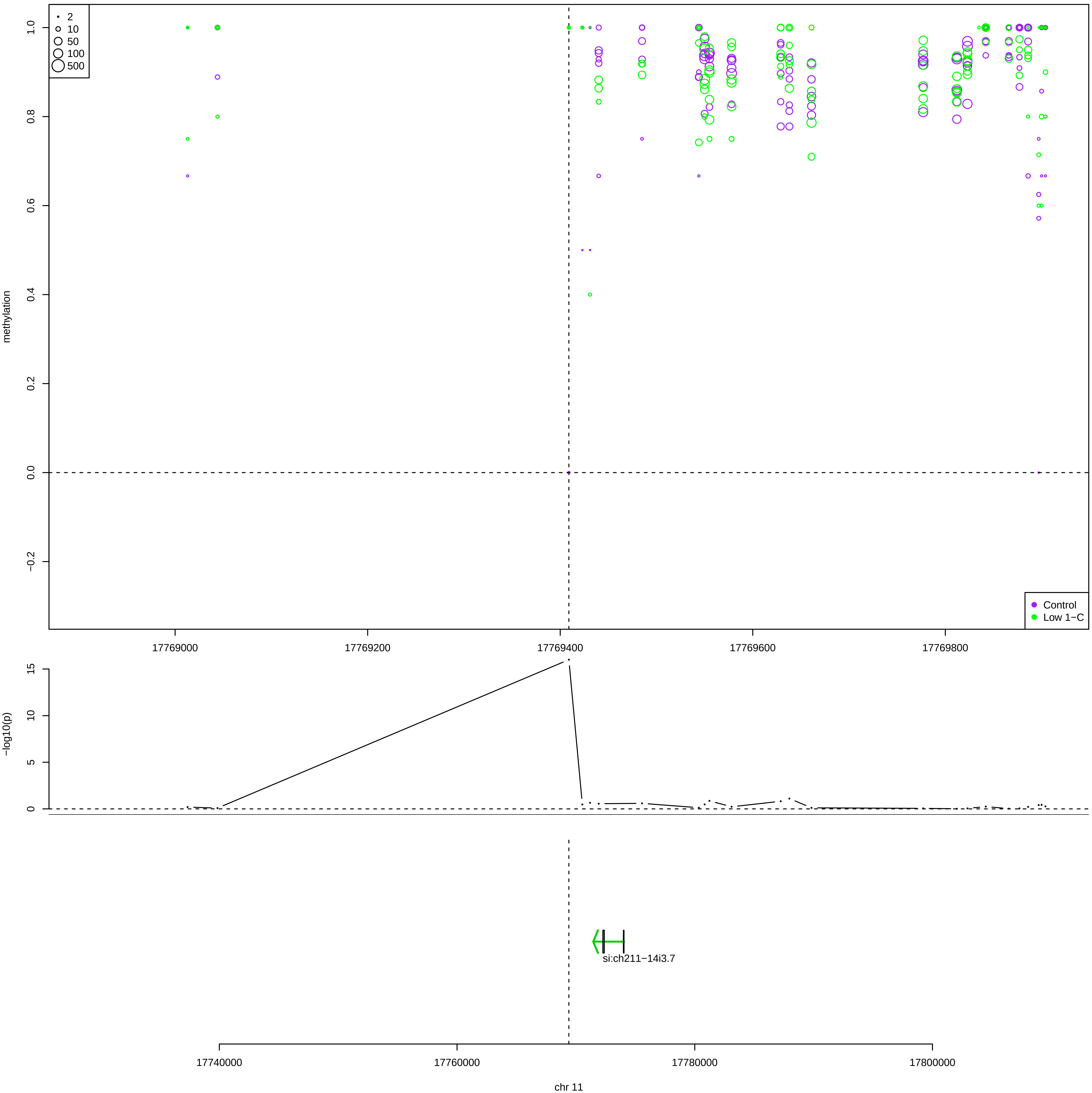
2
10
50
100
500
methylation
1.0
0.8
0.6
0.4
0.2
0.0
−0.2
Control
Low 1−C
17769000
17769200
17769400
17769600
17769800
−log10(p)
15
10
5
0
si:ch211−14i3.7
17740000
17760000
17780000
17800000
chr 11

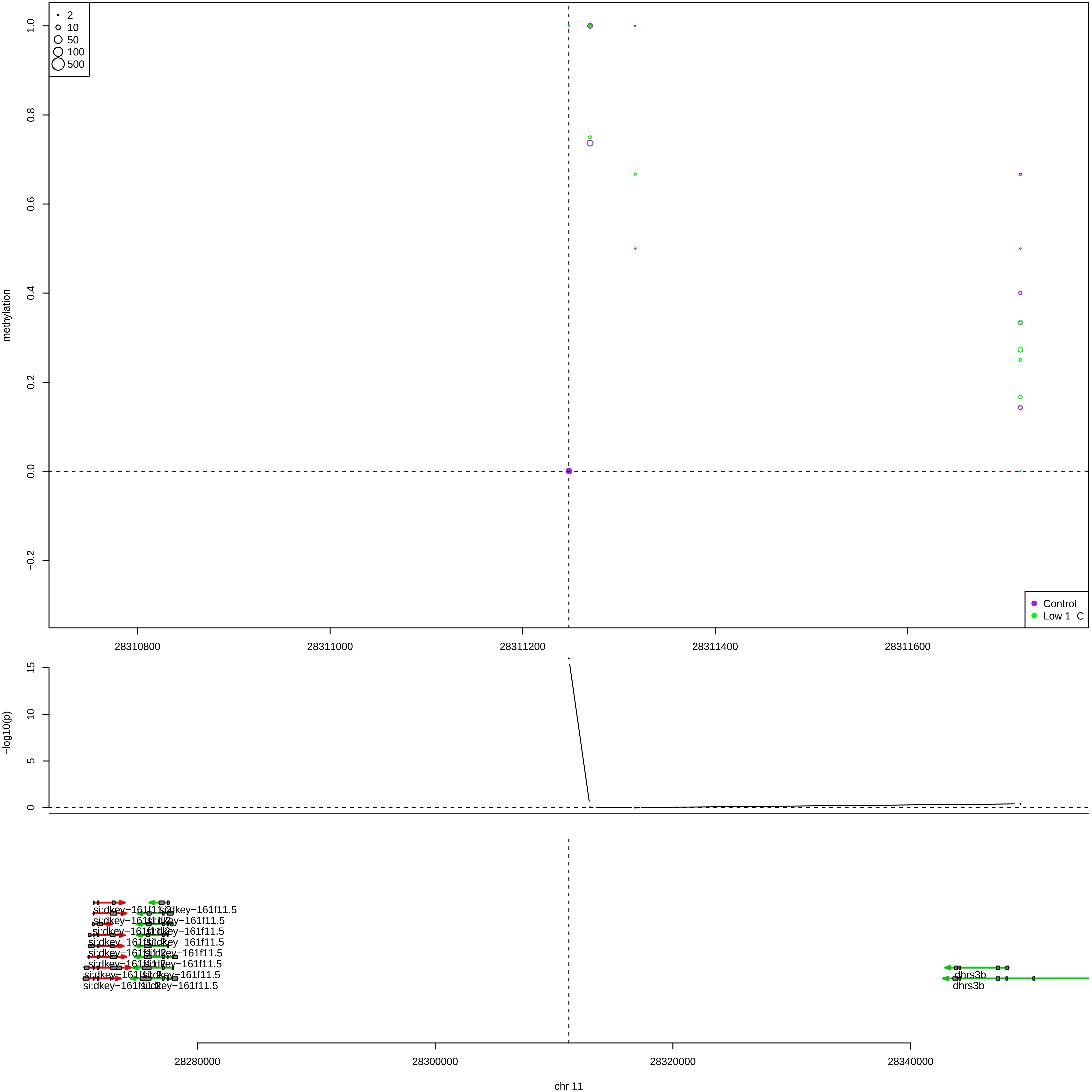

methylation
2
10
50
100
500
Control
Low 1−C
1.0
0.8
0.6
0.4
0.2
0.0
−0.2
28310800
28311000
28311200
28311400
28311600
−log10(p)
15
10
5
0
si:dkey−161f11.2
si:dkey−161f11.5
dhrs3b
28280000
28300000
28320000
28340000
chr 11

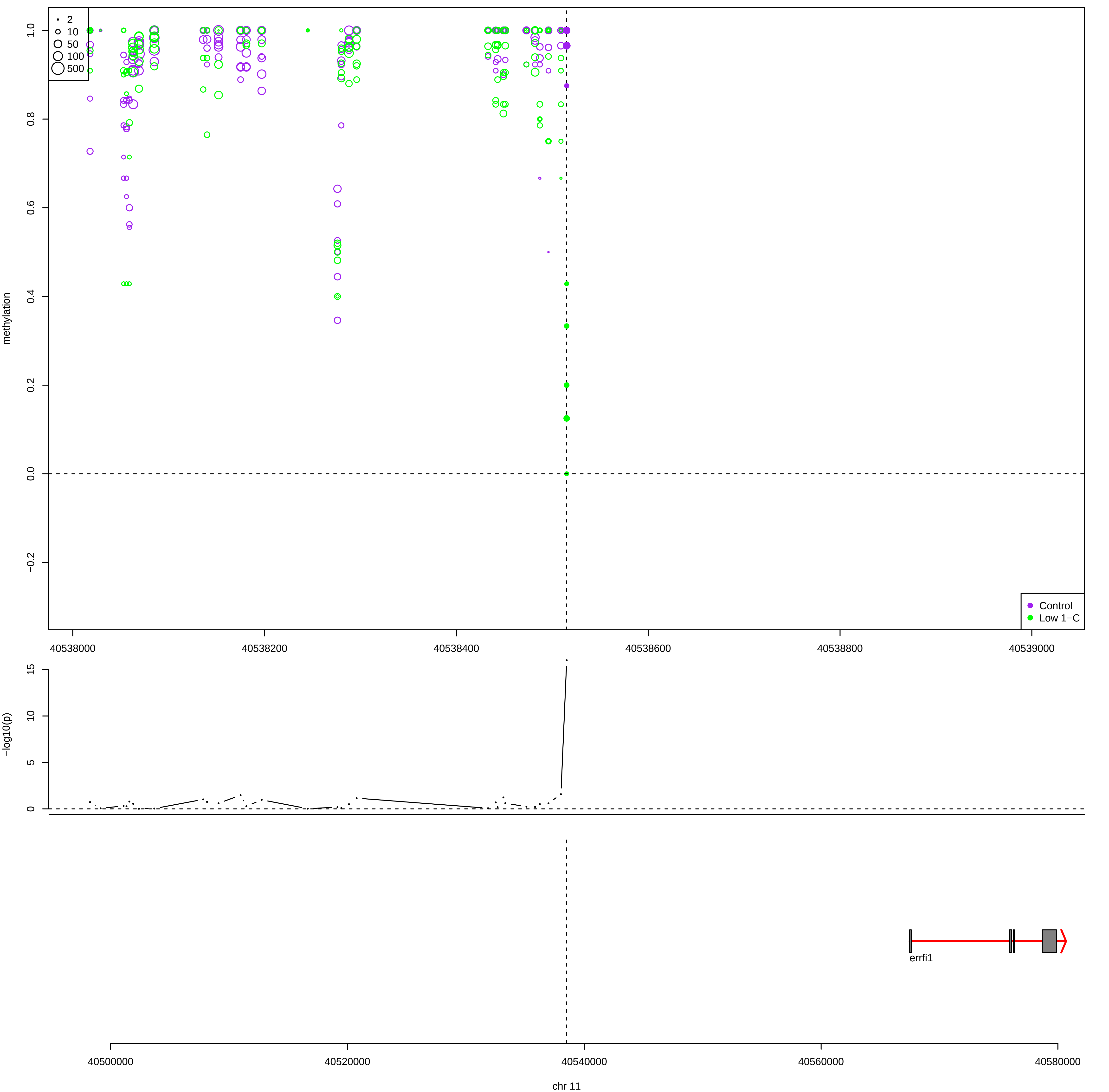

methylation
1.0
0.8
0.6
0.4
0.2
0.0
−0.2
2
10
50
100
500
Control
Low 1−C
40538000
40538200
40538400
40538600
40538800
40539000
−log10(p)
15
10
5
0
errfi1
40500000
40520000
40540000
40560000
40580000
chr 11

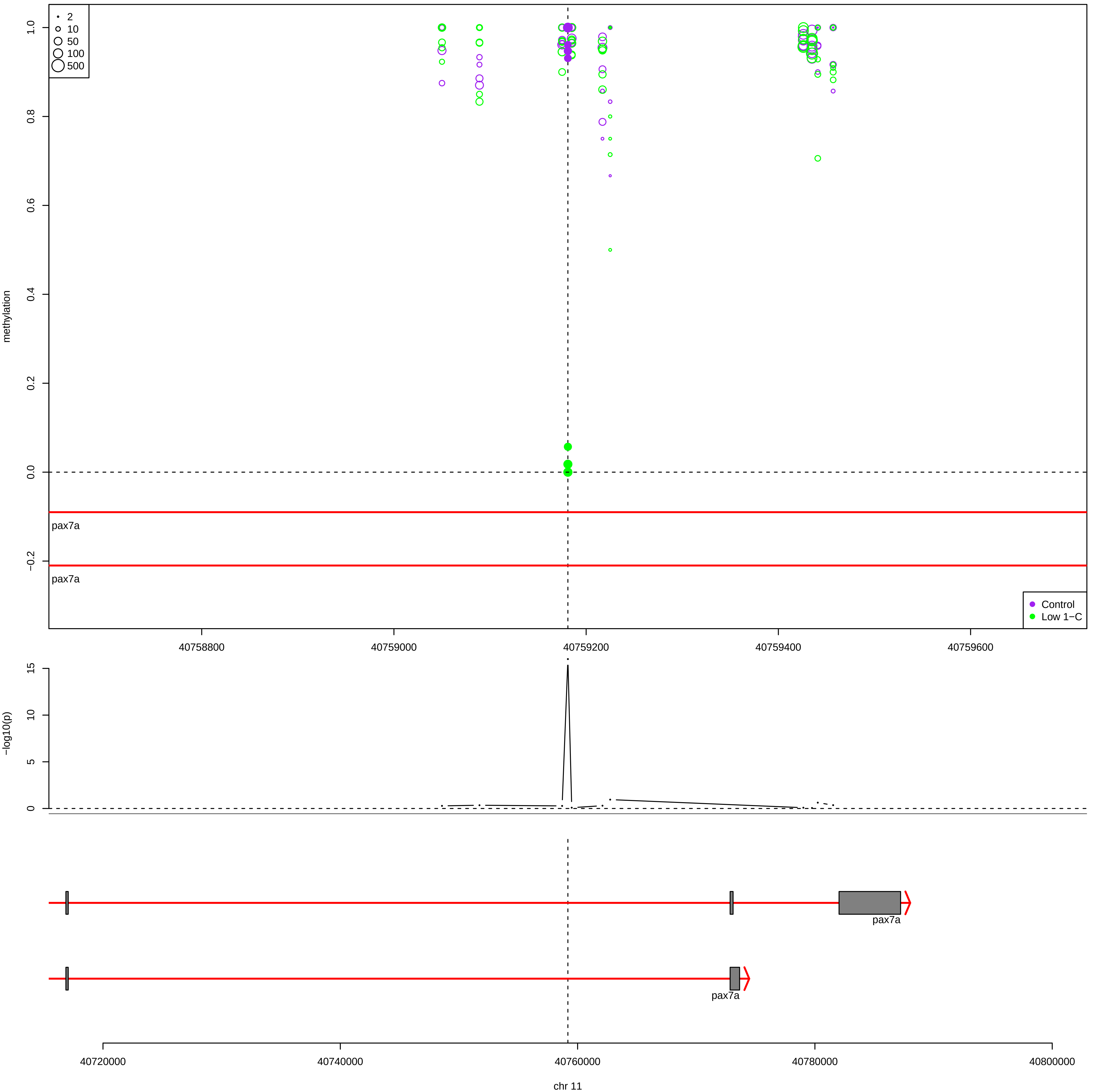

2
10
50
100
500
methylation
1.0
0.8
0.6
0.4
0.2
0.0
−0.2
pax7a
pax7a
Control
Low 1−C
40758800
40759000
40759200
40759400
40759600
−log10(p)
15
10
5
0
pax7a
pax7a
40720000
40740000
40760000
40780000
40800000
chr 11

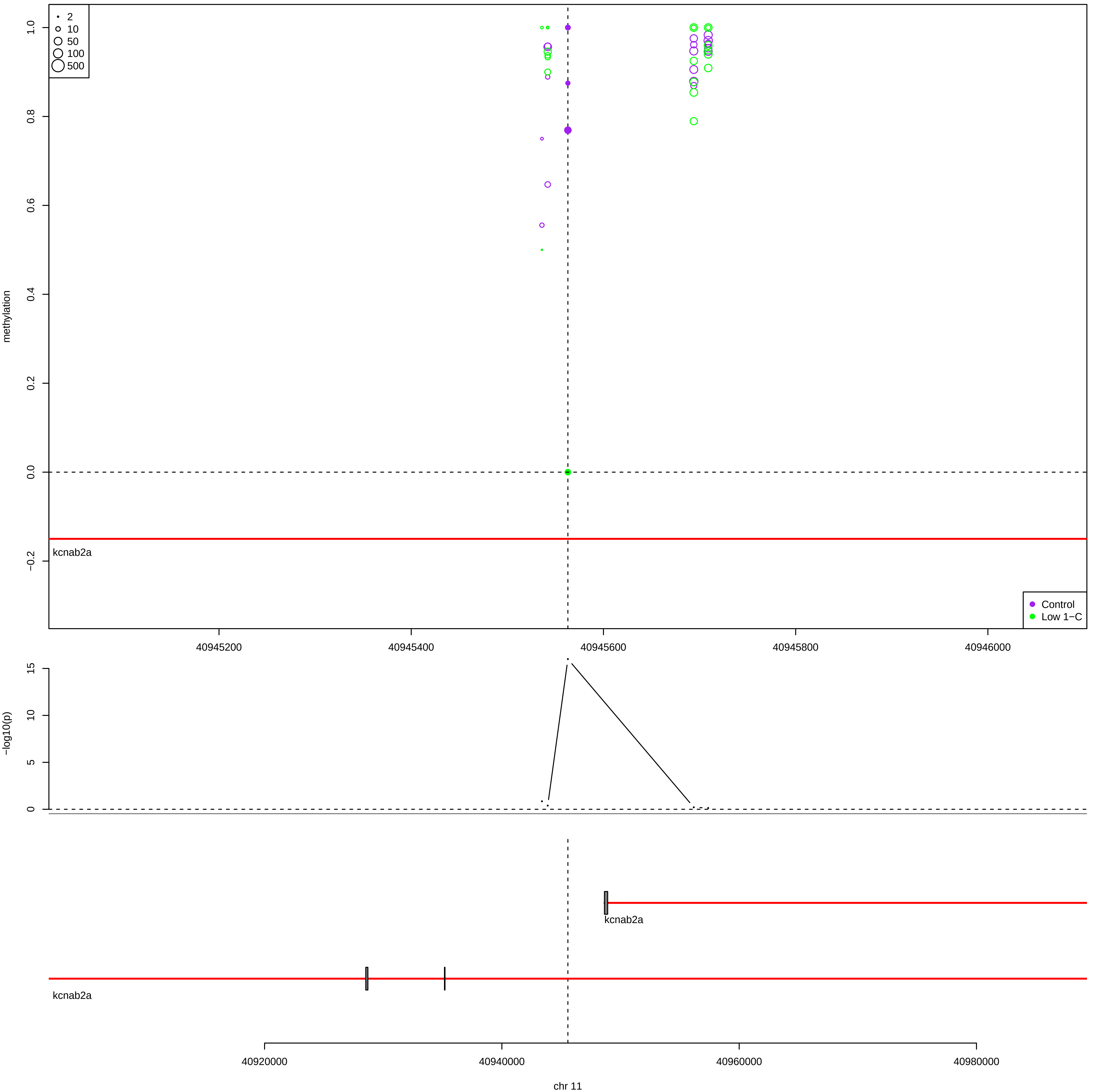

2
10
50
100
500
methylation
1.0
0.8
0.6
0.4
0.2
0.0
−0.2
kcnab2a
Control
Low 1−C
40945200
40945400
40945600
40945800
40946000
−log10(p)
15
10
5
0
kcnab2a
kcnab2a
40920000
40940000
40960000
40980000
chr 11

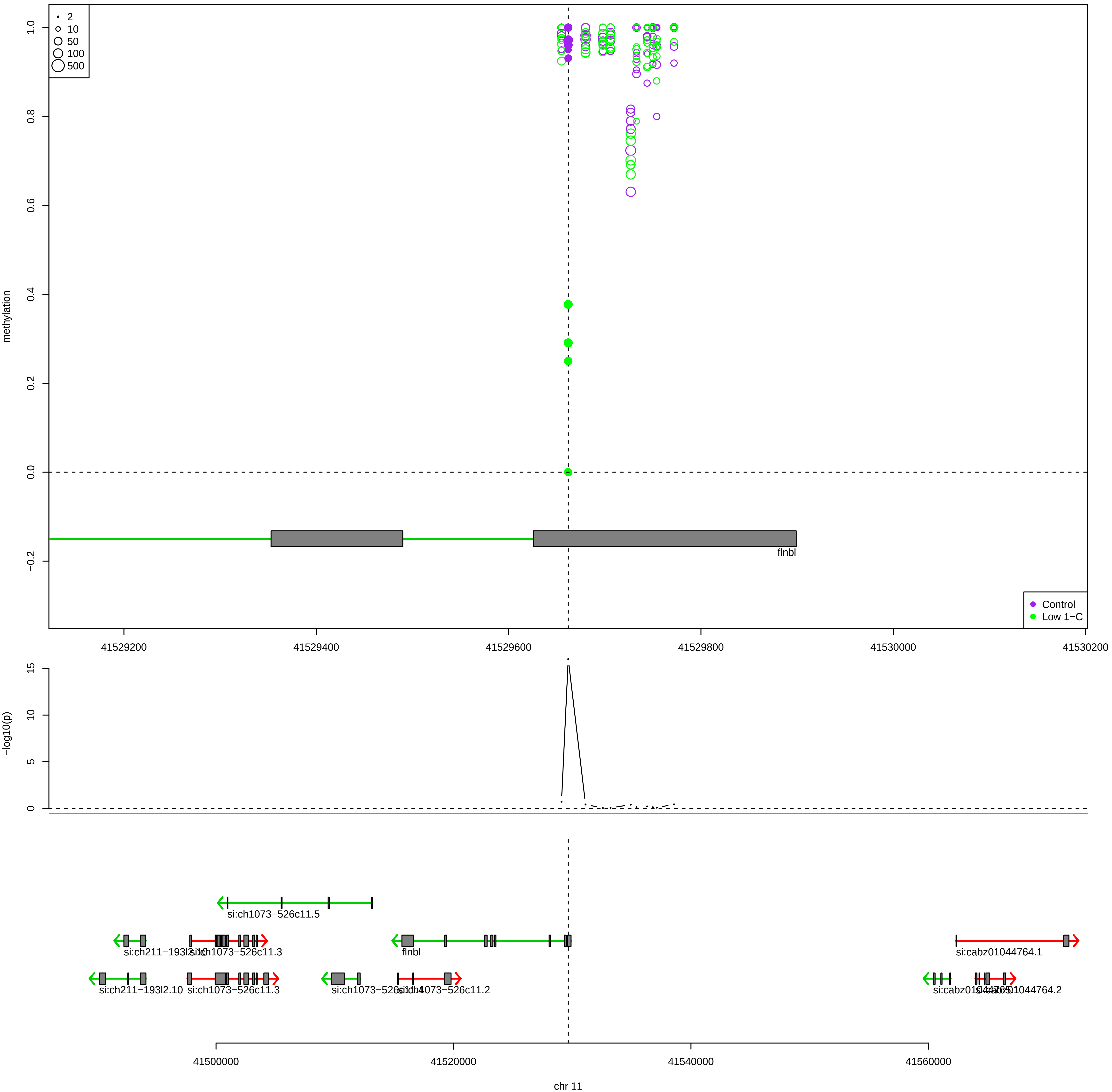

2
10
50
100
500
methylation
1.0
0.8
0.6
0.4
0.2
0.0
−0.2
flnbl
Control
Low 1−C
41529200
41529400
41529600
41529800
41530000
41530200
−log10(p)
15
10
5
0
si:ch1073−526c11.5
si:ch211−193l2.10
si:ch1073−526c11.3
flnbl
si:cabz01044764.1
si:ch211−193l2.10
si:ch1073−526c11.3
si:ch1073−526c11.4
si:ch1073−526c11.2
41500000
41520000
41540000
41560000
chr 11

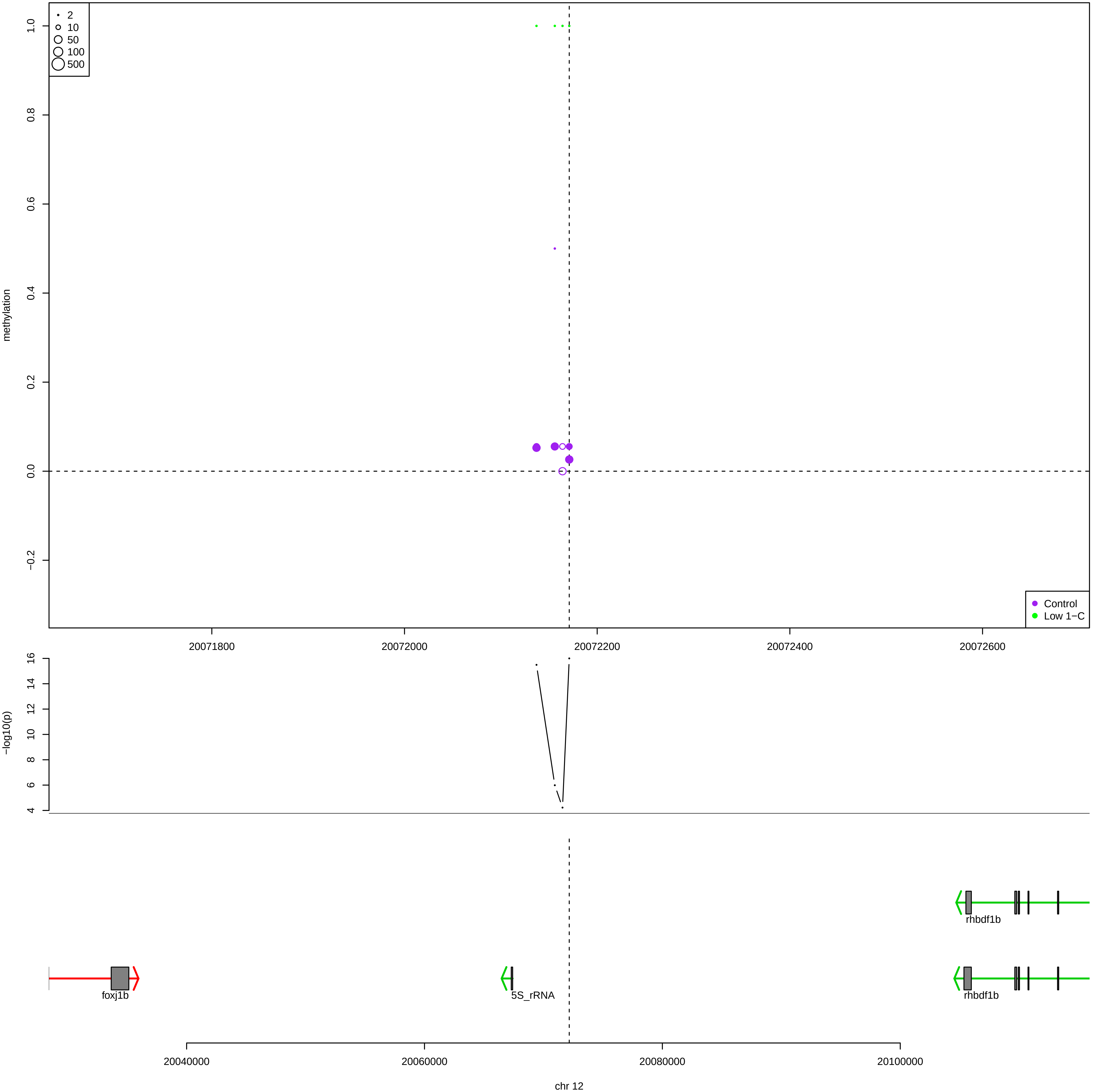

methylation
1.0
0.8
0.6
0.4
0.2
0.0
−0.2
2
10
50
100
500
Control
Low 1−C
20071800
20072000
20072200
20072400
20072600
−log10(p)
16
14
12
10
8
6
4
rhbdf1b
foxj1b
5S_rRNA
rhbdf1b
20040000
20060000
20080000
20100000
chr 12

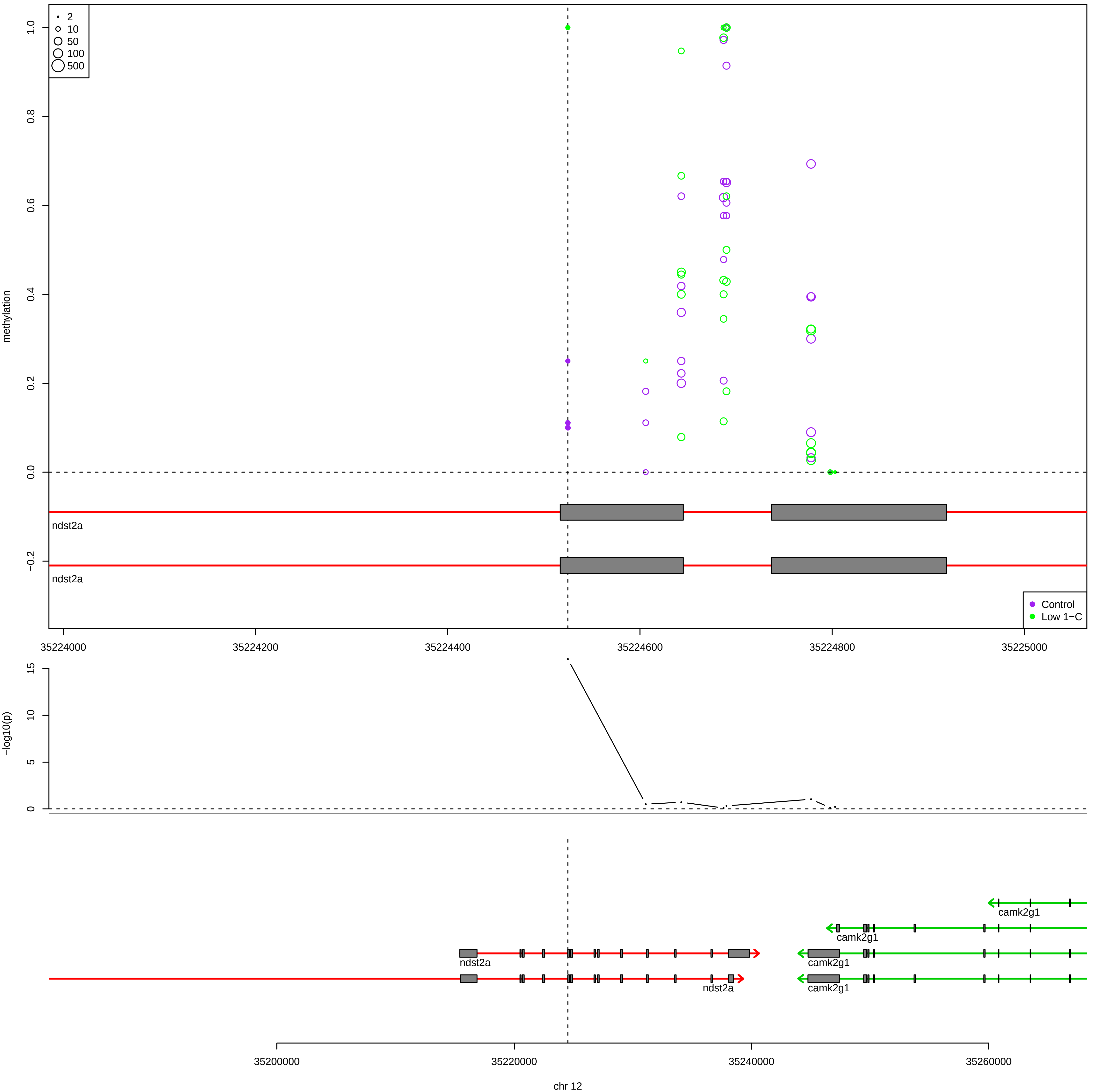

2
10
50
100
500
methylation
1.0
0.8
0.6
0.4
0.2
0.0
−0.2
ndst2a
ndst2a
Control
Low 1−C
35224000
35224200
35224400
35224600
35224800
35225000
−log10(p)
15
10
5
0
camk2g1
camk2g1
ndst2a
camk2g1
ndst2a
camk2g1
35200000
35220000
35240000
35260000
chr 12

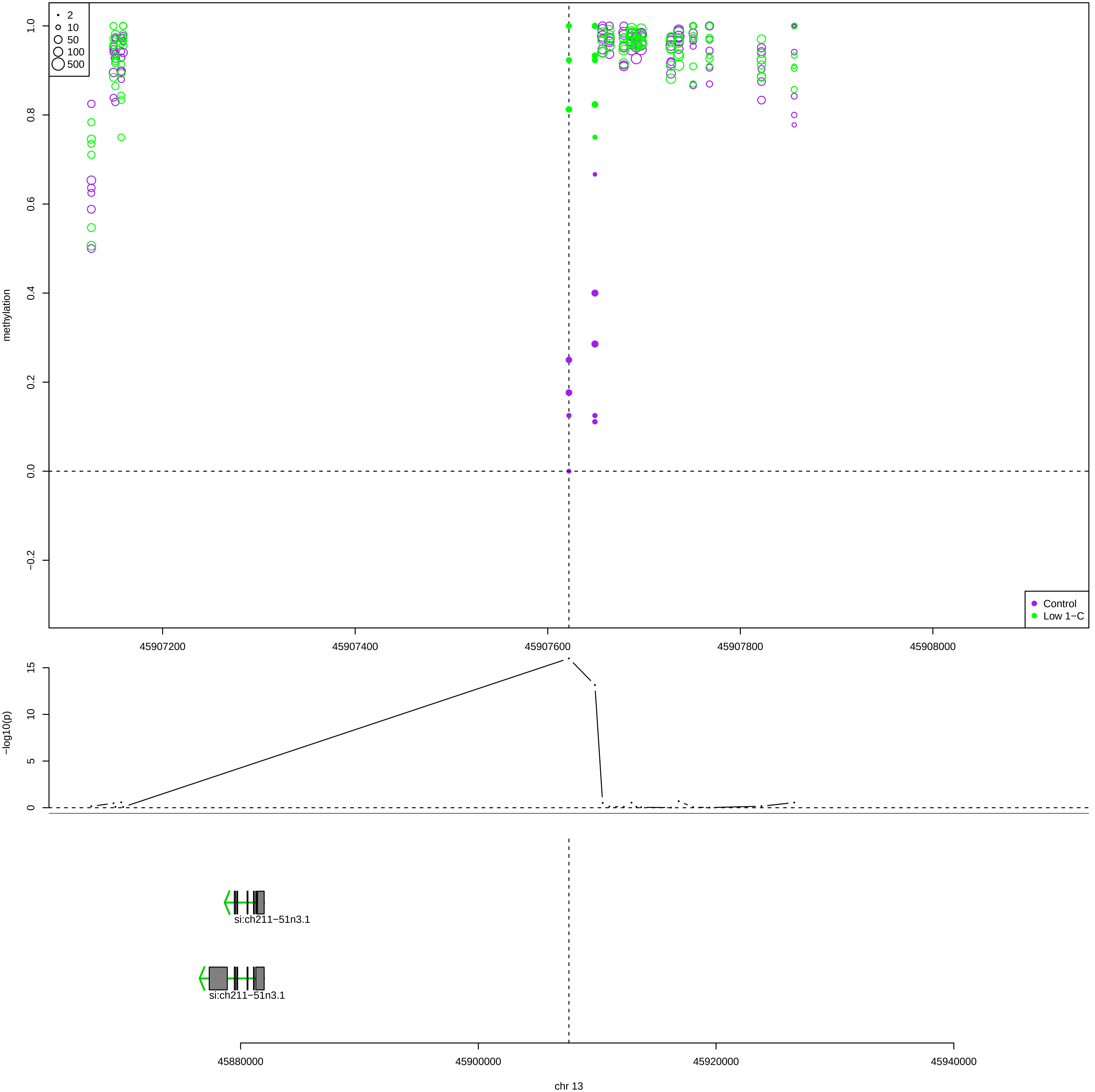

2
10
50
100
500
1.0
0.8
0.6
0.4
0.2
0.0
−0.2
methylation
Control
Low 1−C
45907200
45907400
45907600
45907800
45908000
15
10
5
0
−log10(p)
si:ch211−51n3.1
si:ch211−51n3.1
45880000
45900000
45920000
45940000
chr 13

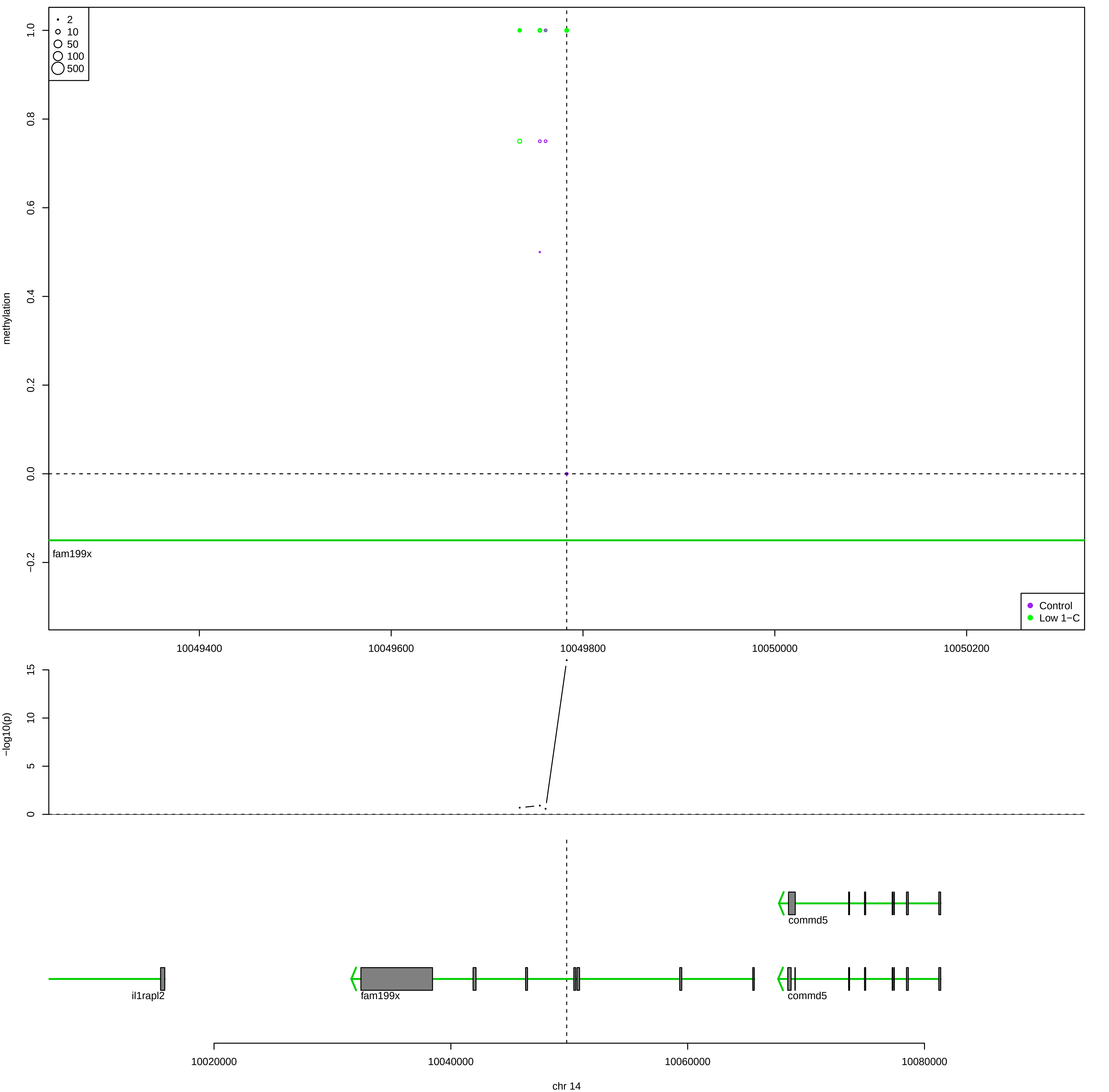

2
10
50
100
500
methylation
1.0
0.8
0.6
0.4
0.2
0.0
−0.2
fam199x
Control
Low 1−C
10049400
10049600
10049800
10050000
10050200
−log10(p)
15
10
5
0
commd5
il1rapl2
fam199x
commd5
10020000
10040000
10060000
10080000
chr 14

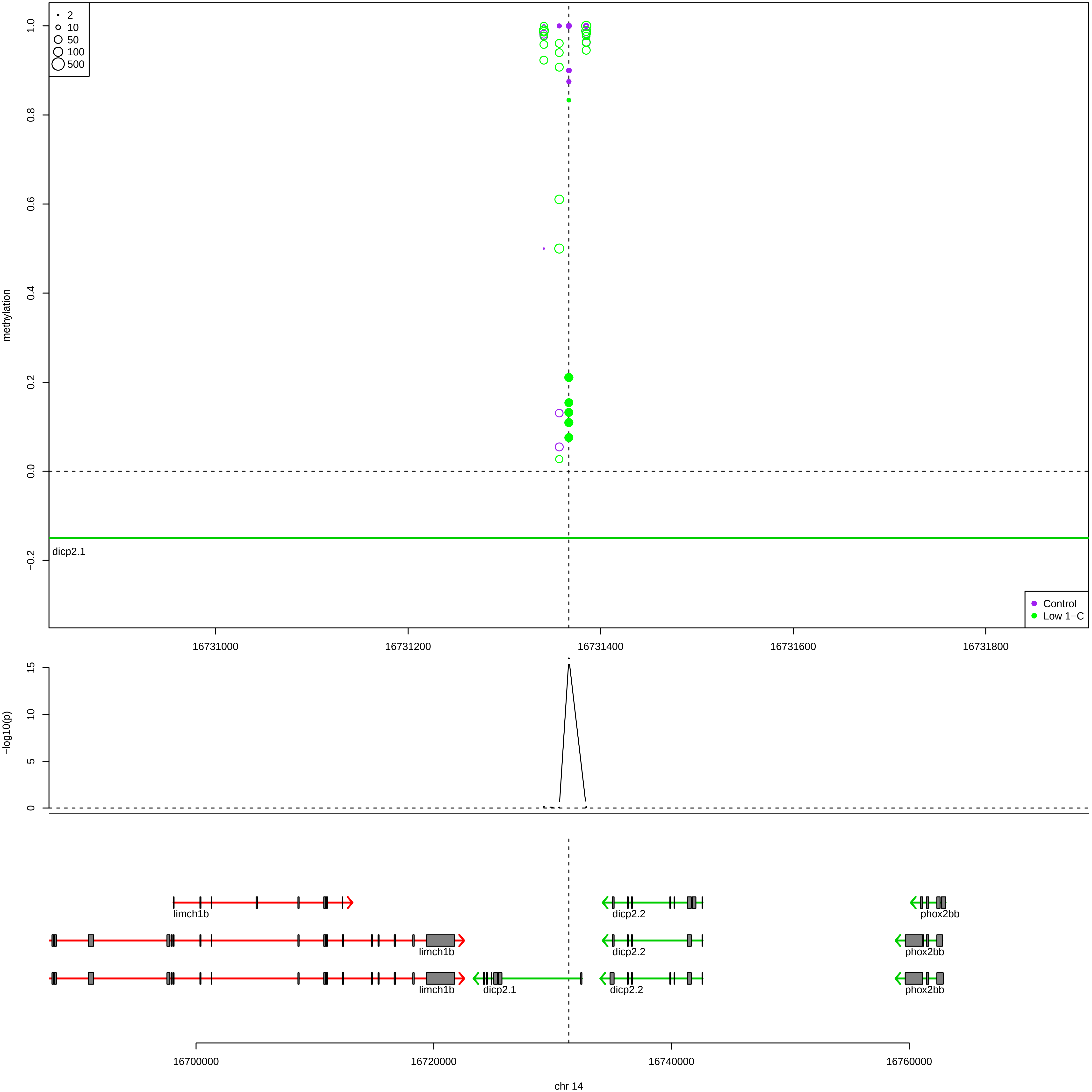

2
10
50
100
500
methylation
1.0
0.8
0.6
0.4
0.2
0.0
−0.2
dicp2.1
Control
Low 1−C
16731000
16731200
16731400
16731600
16731800
−log10(p)
15
10
5
0
limch1b
limch1b
limch1b
dicp2.1
dicp2.2
dicp2.2
dicp2.2
phox2bb
phox2bb
phox2bb
16700000
16720000
16740000
16760000
chr 14

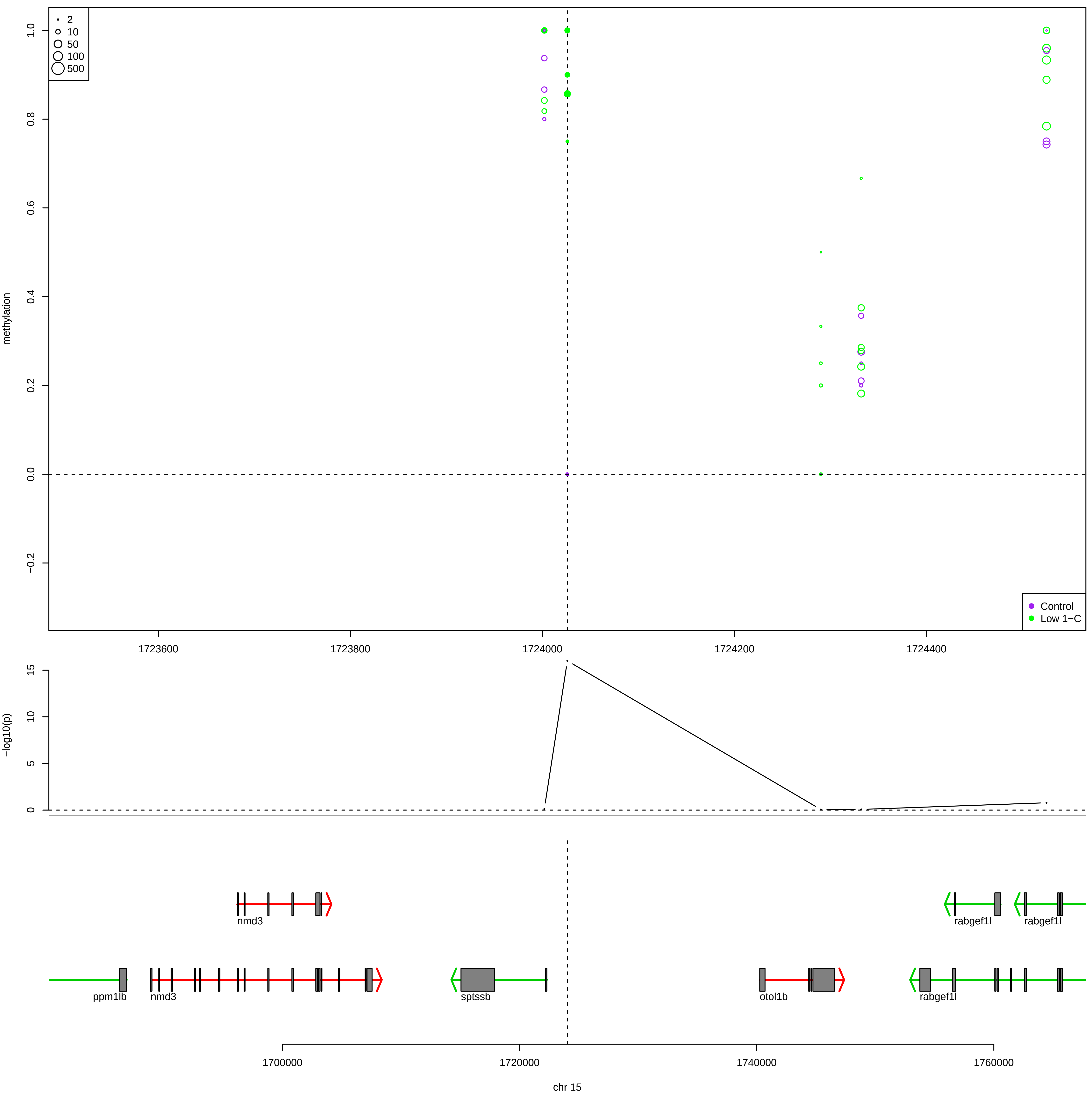

2
10
50
100
500
methylation
1.0
0.8
0.6
0.4
0.2
0.0
−0.2
Control
Low 1−C
1723600
1723800
1724000
1724200
1724400
−log10(p)
15
10
5
0
nmd3
rabgef1l
rabgef1l
ppm1lb
nmd3
sptssb
otol1b
rabgef1l
1700000
1720000
1740000
1760000
chr 15

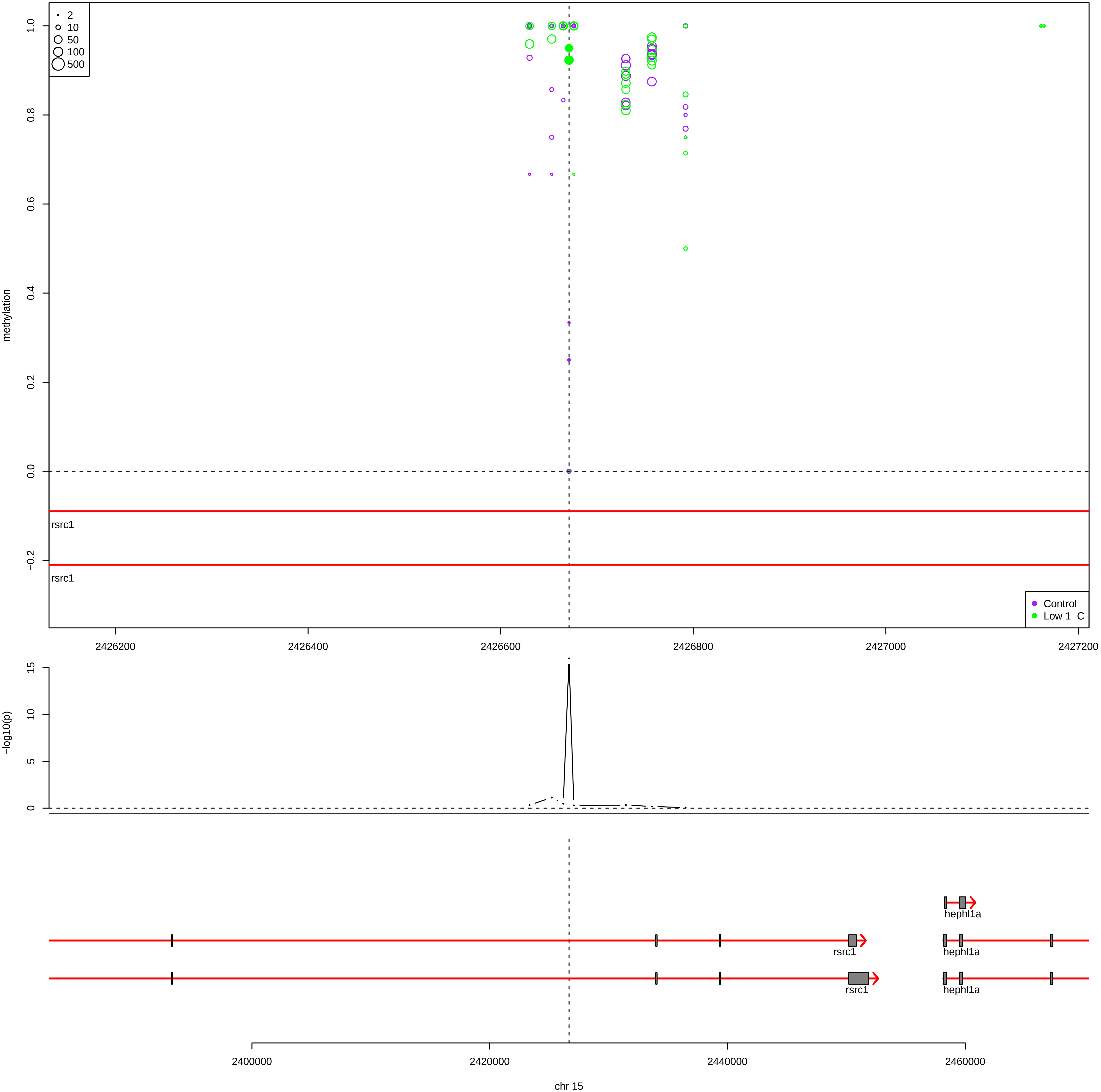

2
10
50
100
500
methylation
1.0
0.8
0.6
0.4
0.2
0.0
−0.2
rsrc1
rsrc1
Control
Low 1−C
2426200
2426400
2426600
2426800
2427000
2427200
−log10(p)
15
10
5
0
hephl1a
rsrc1
hephl1a
rsrc1
hephl1a
2400000
2420000
2440000
2460000
chr 15

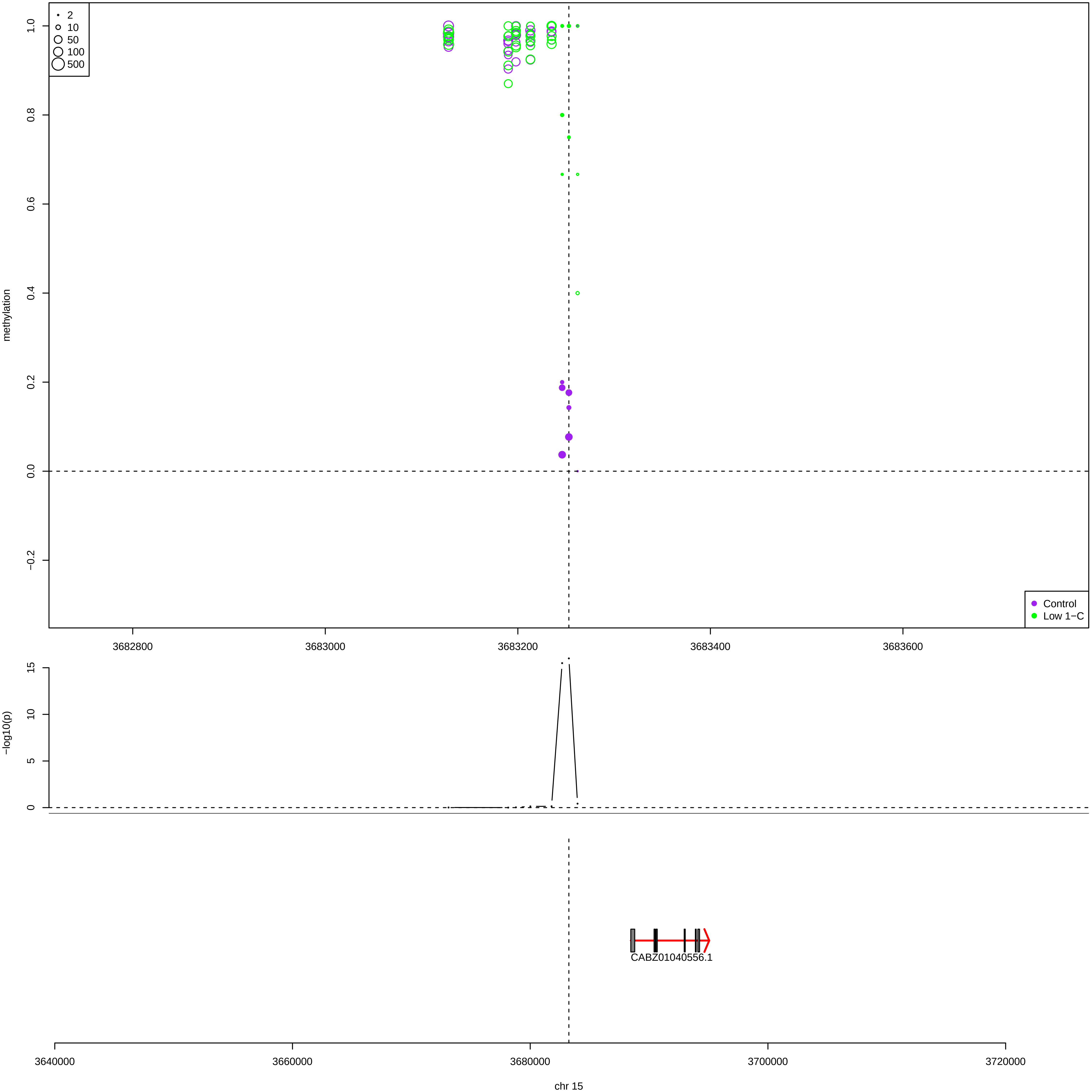

2
10
50
100
500
methylation
1.0
0.8
0.6
0.4
0.2
0.0
−0.2
Control
Low 1−C
3682800
3683000
3683200
3683400
3683600
−log10(p)
15
10
5
0
CABZ01040556.1
3640000
3660000
3680000
3700000
3720000
chr 15

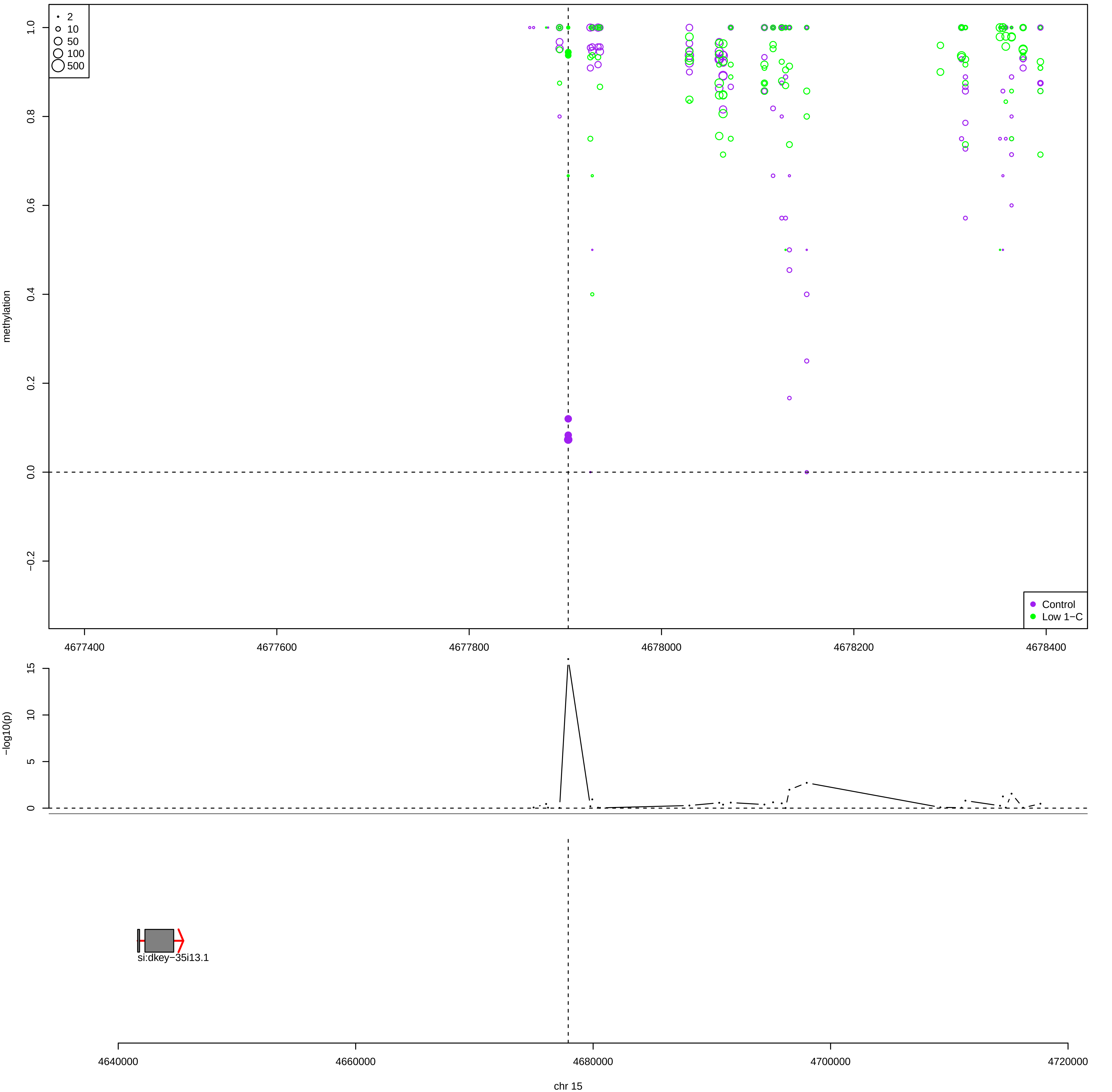

2
10
50
100
500
methylation
1.0
0.8
0.6
0.4
0.2
0.0
−0.2
Control
Low 1−C
4677400
4677600
4677800
4678000
4678200
4678400
−log10(p)
15
10
5
0
si:dkey−35i13.1
4640000
4660000
4680000
4700000
4720000
chr 15

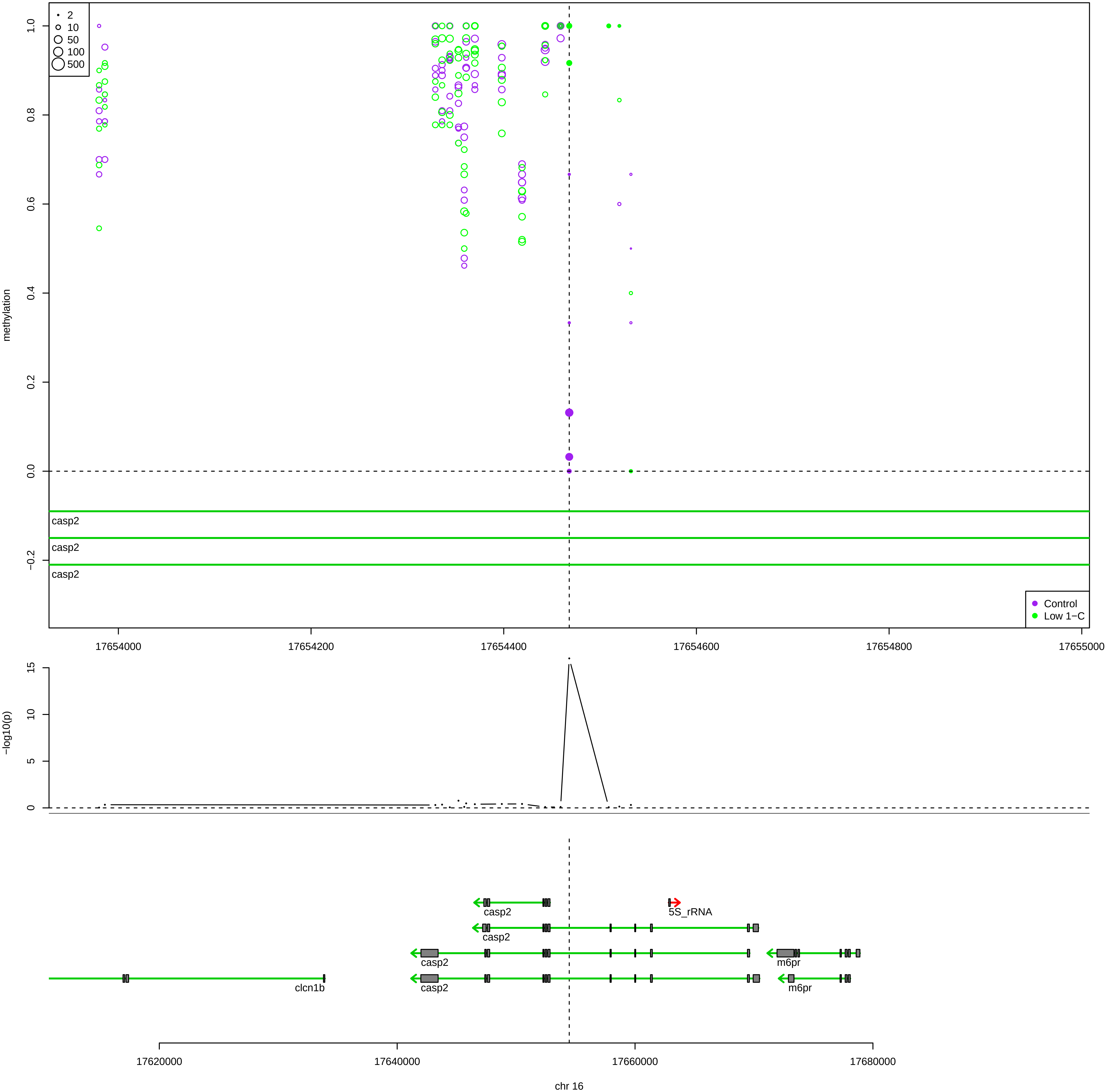

2
10
50
100
500
methylation
1.0
0.8
0.6
0.4
0.2
0.0
−0.2
casp2
casp2
casp2
Control
Low 1−C
17654000
17654200
17654400
17654600
17654800
17655000
−log10(p)
15
10
5
0
casp2
5S_rRNA
casp2
casp2
m6pr
clcn1b
casp2
m6pr
17620000
17640000
17660000
17680000
chr 16

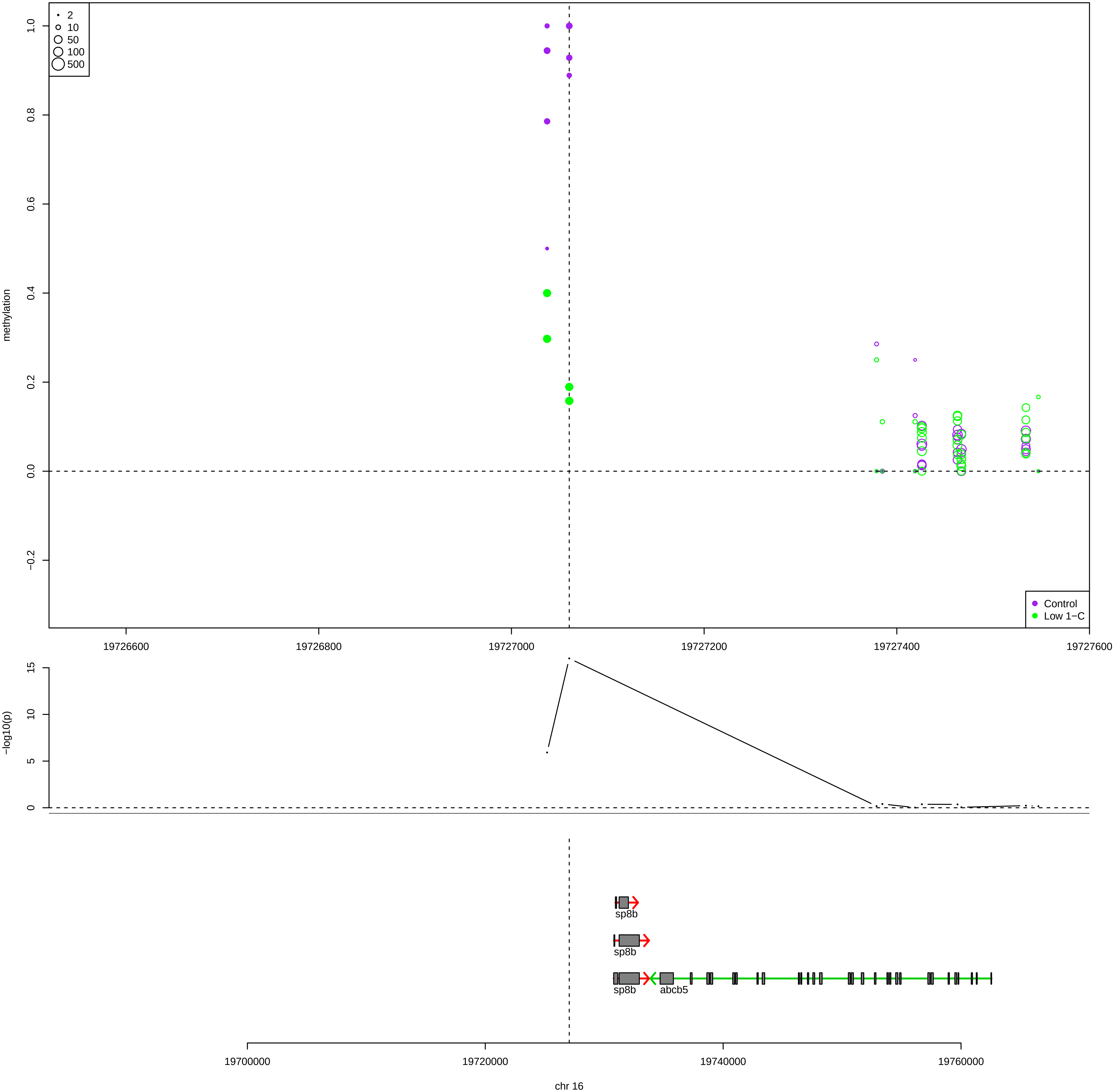

2
10
50
100
500
methylation
1.0
0.8
0.6
0.4
0.2
0.0
−0.2
Control
Low 1−C
19726600
19726800
19727000
19727200
19727400
19727600
−log10(p)
15
10
5
0
sp8b
sp8b
sp8b
abcb5
19700000
19720000
19740000
19760000
chr 16
