# Supplementary material for: Parental micronutrient deficiency distorts liver DNA methylation and expression of lipid genes associated with a fatty-liver-like phenotype in offspring
Source: Sci Rep. 2018 Feb 14;8:3055. doi: 10.1038/s41598-018-21211-5 (PMC5812986; doi:10.1038/s41598-018-21211-5)
Supplement: Supplementary file 4 — Figure S14 [file 41598_2018_21211_MOESM4_ESM.pdf]

Supplementary Info, figure S14:

Parental micronutrient deficiency distorts liver DNA methylation and expression of lipid genes associated with a fatty-liver-like phenotype in offspring

Kaja H. Skjærven[1*], Lars Martin Jakt[2], Jorge M.O. Fernandes[2], John Arne Dahl[3], Anne-Catrin Adam[1], Johanna Klughammer[4], Christoph Bock[4] and Marit Espe[1]

[1]*Institute of Marine Research, IMR, Norway*

[2]*Faculty of Biosciences and Aquaculture, Nord University, Norway*

[3]*Department of Microbiology, Oslo University Hospital, Norway*

[4]*CeMM Research Center for Molecular Medicine of the Austrian Academy of Sciences, 1090 Vienna, Austria*

[*]Corresponding author:

Dr. Kaja Helvik Skjærven

Institute of Marine Research (IMR),

PO Box 1870 Nordnes,

5817 Bergen, Norway

Tel.:+4741458362; fax: +4755905299.

E-mail address: ksk@hi.no

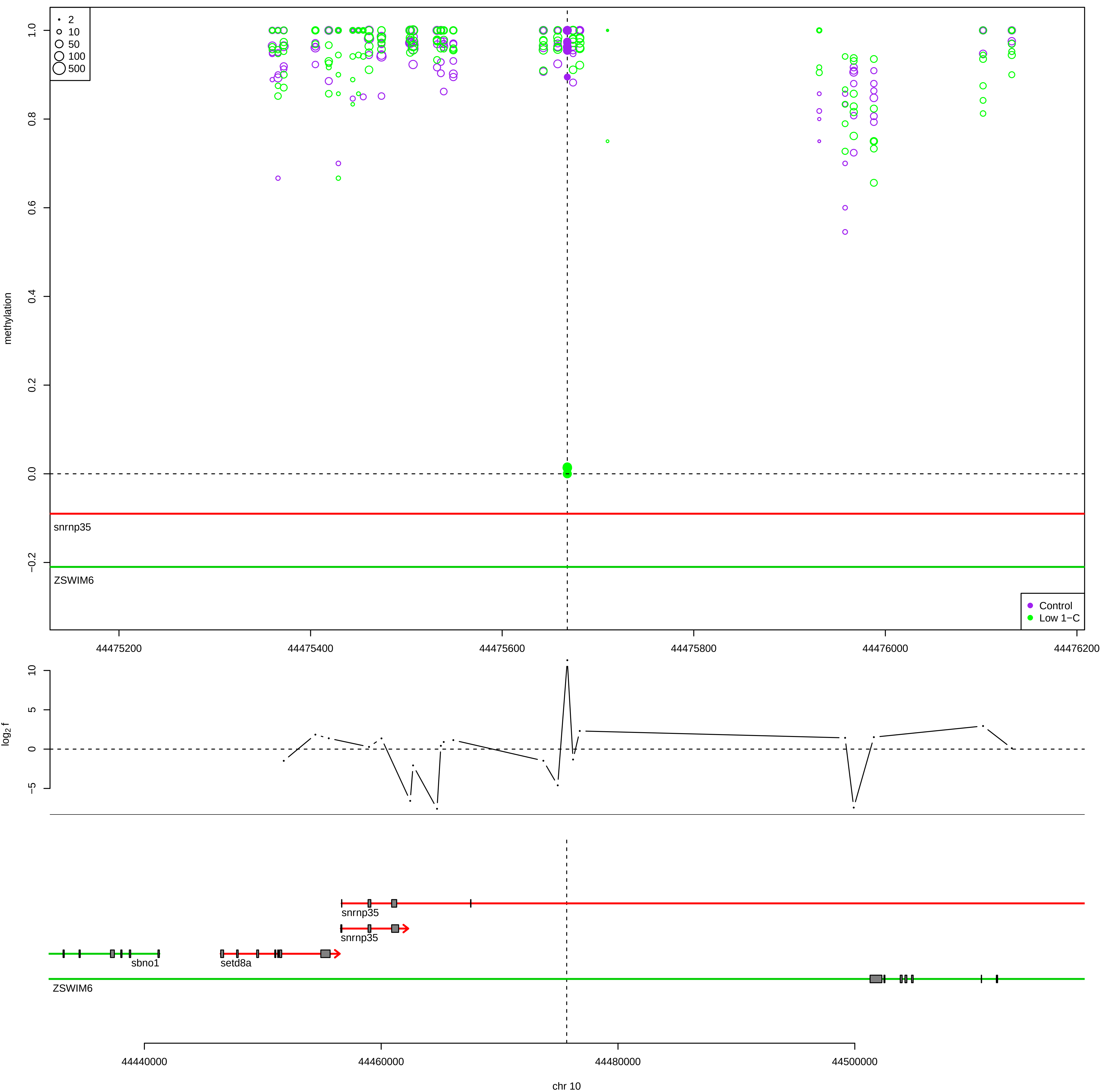

2
10
50
100
500
methylation
1.0
0.8
0.6
0.4
0.2
0.0
−0.2
snrnp35
ZSWIM6
Control
Low 1−C
44475200
44475400
44475600
44475800
44476000
44476200
log2 f
10
5
0
−5
snrnp35
snrnp35
sbno1
setd8a
ZSWIM6
44440000
44460000
44480000
44500000
chr 10

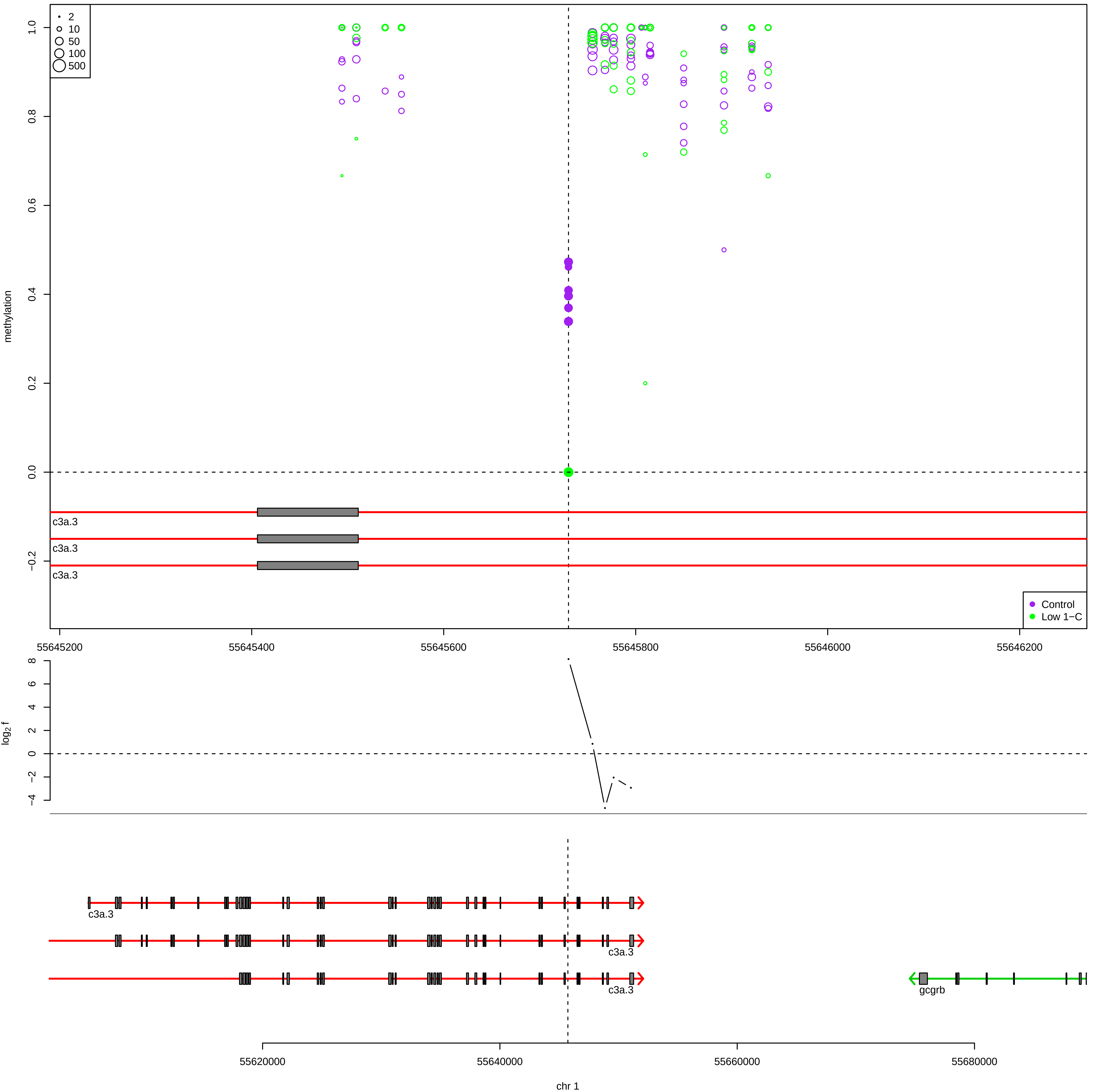

2
10
50
100
500
methylation
1.0
0.8
0.6
0.4
0.2
0.0
−0.2
c3a.3
c3a.3
c3a.3
Control
Low 1−C
55645200
55645400
55645600
55645800
55646000
55646200
log_2 f
8
6
4
2
0
−2
−4
c3a.3
c3a.3
c3a.3
gcgrb
55620000
55640000
55660000
55680000
chr 1

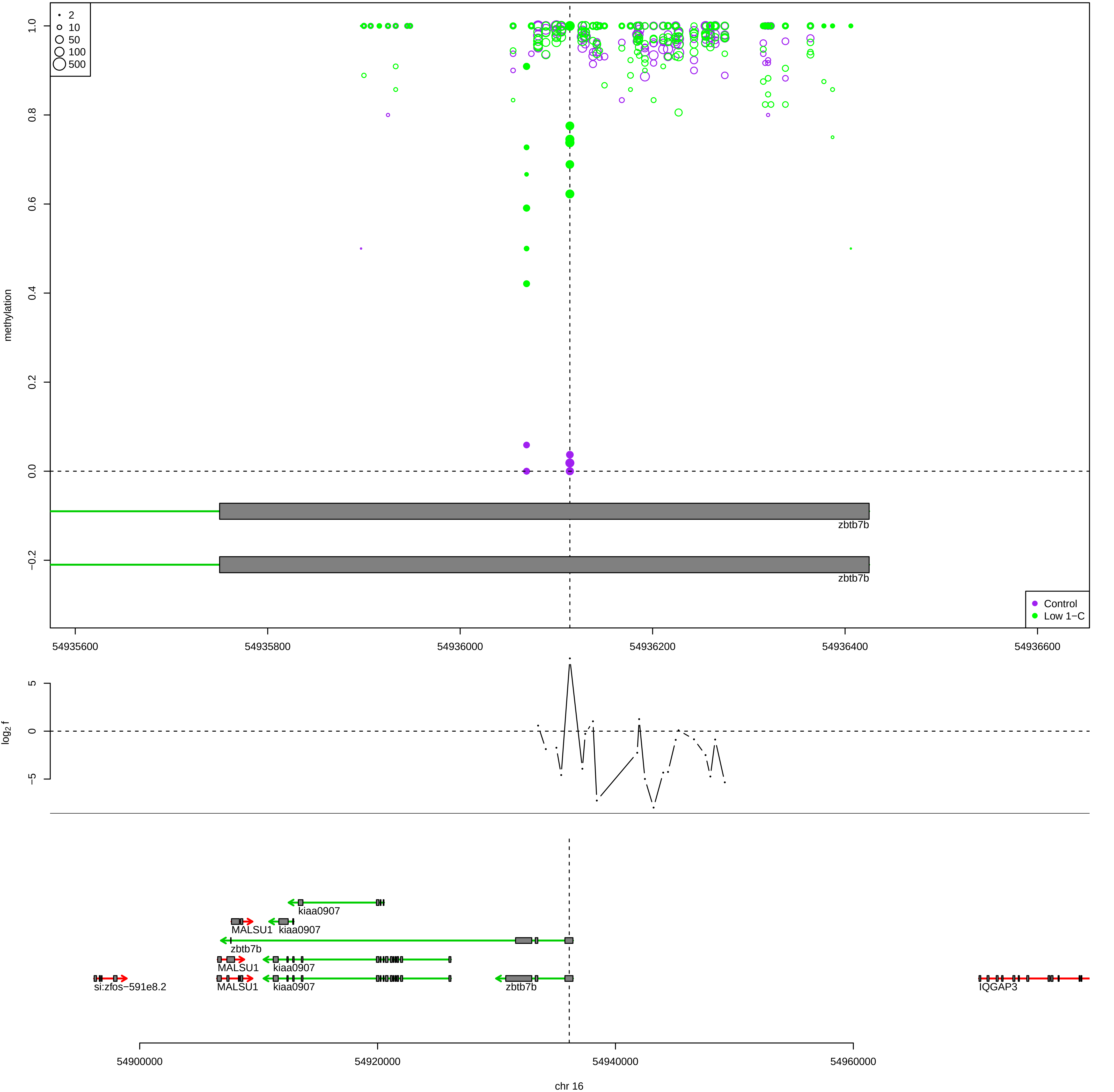

2
10
50
100
500
methylation
1.0
0.8
0.6
0.4
0.2
0.0
−0.2
zbtb7b
zbtb7b
Control
Low 1−C
54935600
54935800
54936000
54936200
54936400
54936600
$\log_2 f$
5
0
−5
kiaa0907
MALSU1
kiaa0907
zbtb7b
MALSU1
kiaa0907
si:zfos−591e8.2
MALSU1
kiaa0907
zbtb7b
IQGAP3
54900000
54920000
54940000
54960000
chr 16

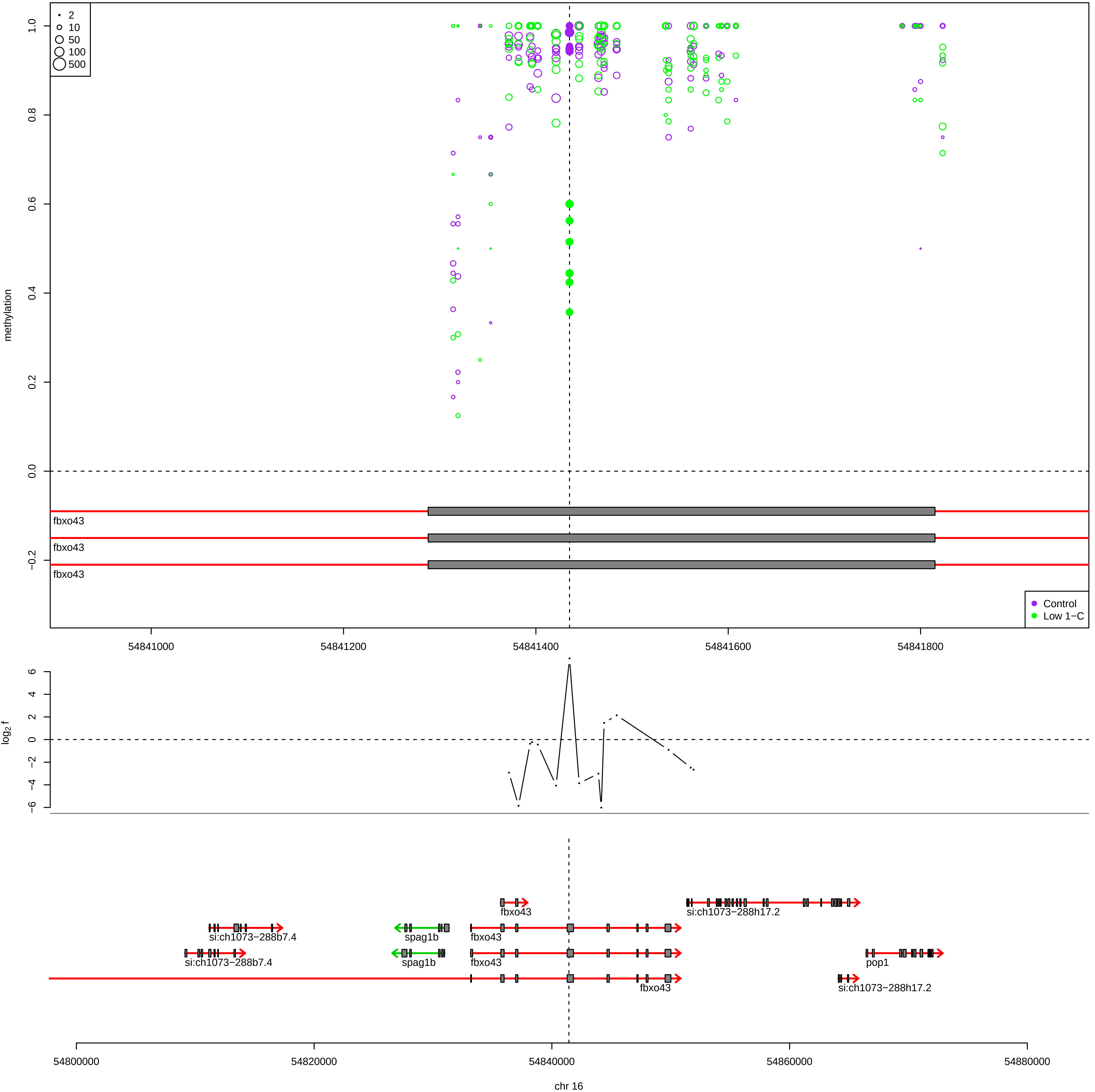

2
10
50
100
500
methylation
fbxo43
fbxo43
fbxo43
Control
Low 1−C
54841000
54841200
54841400
54841600
54841800
log2 f
si:ch1073−288b7.4
si:ch1073−288b7.4
spag1b
spag1b
fbxo43
fbxo43
fbxo43
fbxo43
si:ch1073−288h17.2
si:ch1073−288h17.2
pop1
54800000
54820000
54840000
54860000
54880000
chr 16

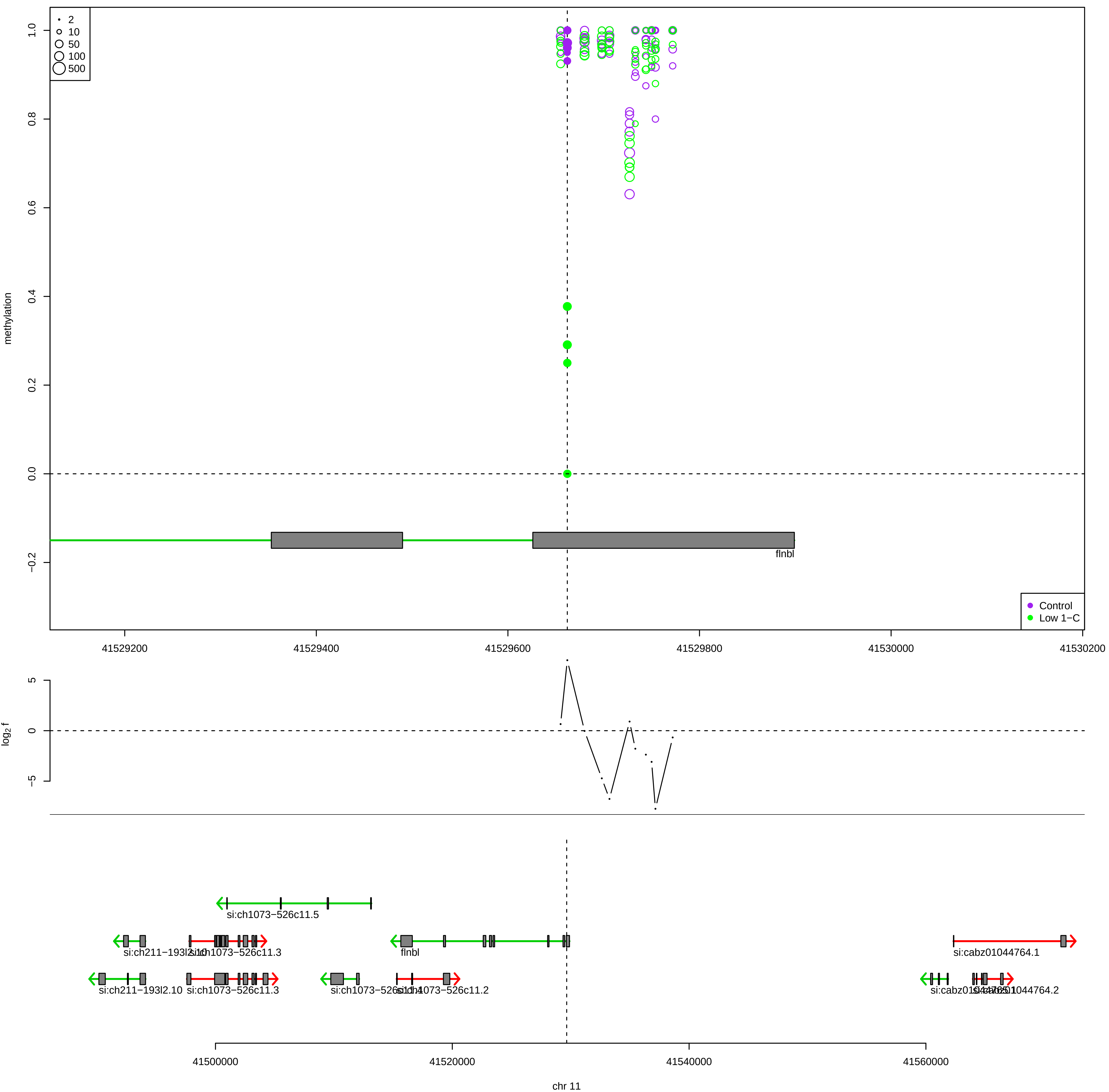

2
10
50
100
500
methylation
1.0
0.8
0.6
0.4
0.2
0.0
−0.2
flnbl
Control
Low 1−C
41529200
41529400
41529600
41529800
41530000
41530200
log2 f
5
0
−5
si:ch1073−526c11.5
si:ch211−193l2.10
si:ch1073−526c11.3
flnbl
si:cabz01044764.1
si:ch211−193l2.10
si:ch1073−526c11.3
si:ch1073−526c11.1
si:ch1073−526c11.2
si:cabz01044765.1
si:cabz01044764.2
41500000
41520000
41540000
41560000
chr 11

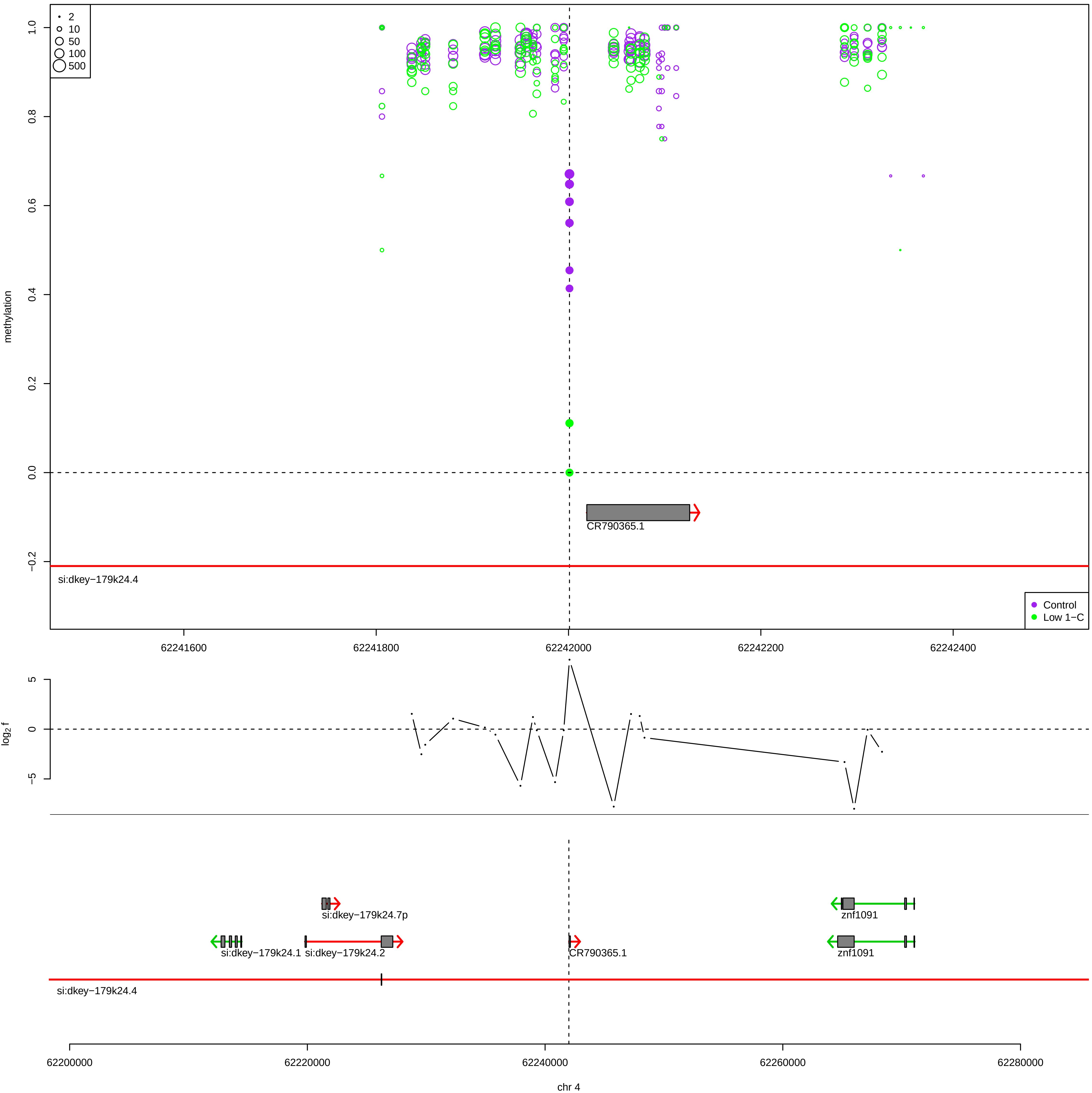

2
10
50
100
500
methylation
1.0
0.8
0.6
0.4
0.2
0.0
−0.2
CR790365.1
si:dkey−179k24.4
Control
Low 1−C
62241600
62241800
62242000
62242200
62242400
log2 f
5
0
−5
si:dkey−179k24.7p
znf1091
si:dkey−179k24.1
si:dkey−179k24.2
CR790365.1
znf1091
si:dkey−179k24.4
62200000
62220000
62240000
62260000
62280000
chr 4

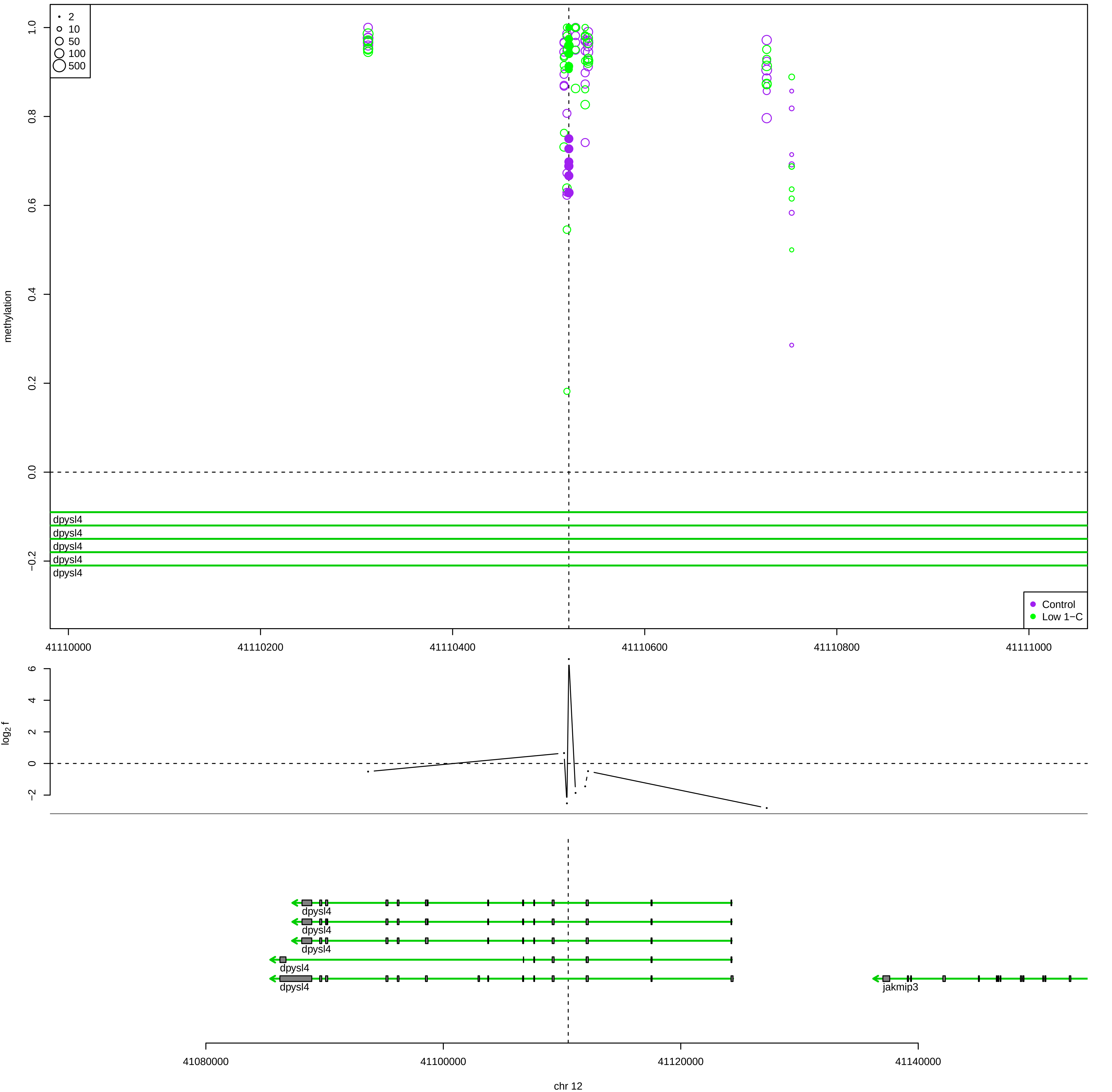

methylation
2
10
50
100
500
dpysl4
dpysl4
dpysl4
dpysl4
dpysl4
Control
Low 1−C
41110000
41110200
41110400
41110600
41110800
41111000
log2 f
dpysl4
dpysl4
dpysl4
dpysl4
dpysl4
jakmip3
41080000
41100000
41120000
41140000
chr 12

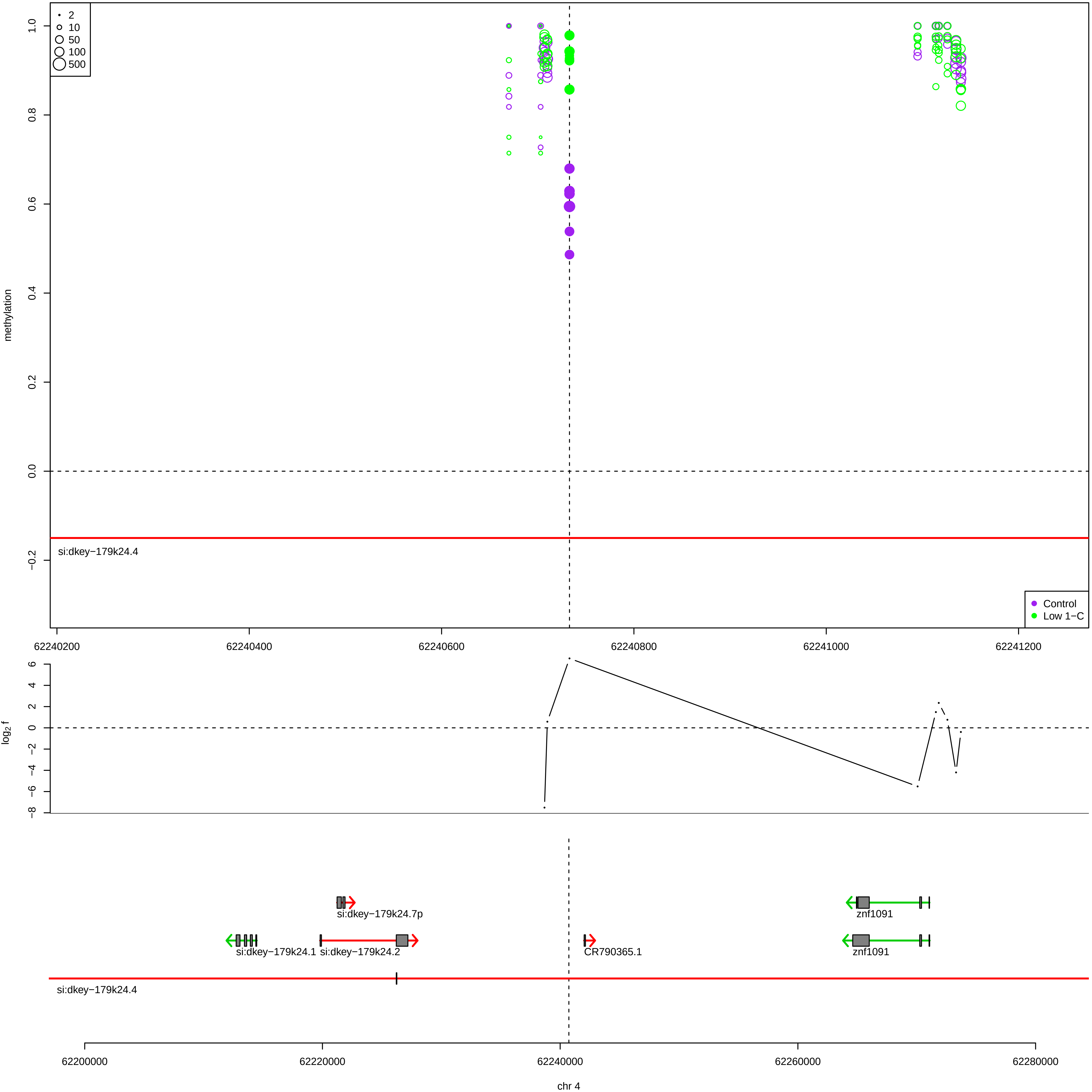

2
10
50
100
500
methylation
1.0
0.8
0.6
0.4
0.2
0.0
−0.2
si:dkey−179k24.4
Control
Low 1−C
62240200
62240400
62240600
62240800
62241000
62241200
6
4
2
0
−2
−4
−6
−8
log2 f
si:dkey−179k24.7p
znf1091
si:dkey−179k24.1
si:dkey−179k24.2
CR790365.1
znf1091
si:dkey−179k24.4
62200000
62220000
62240000
62260000
62280000
chr 4

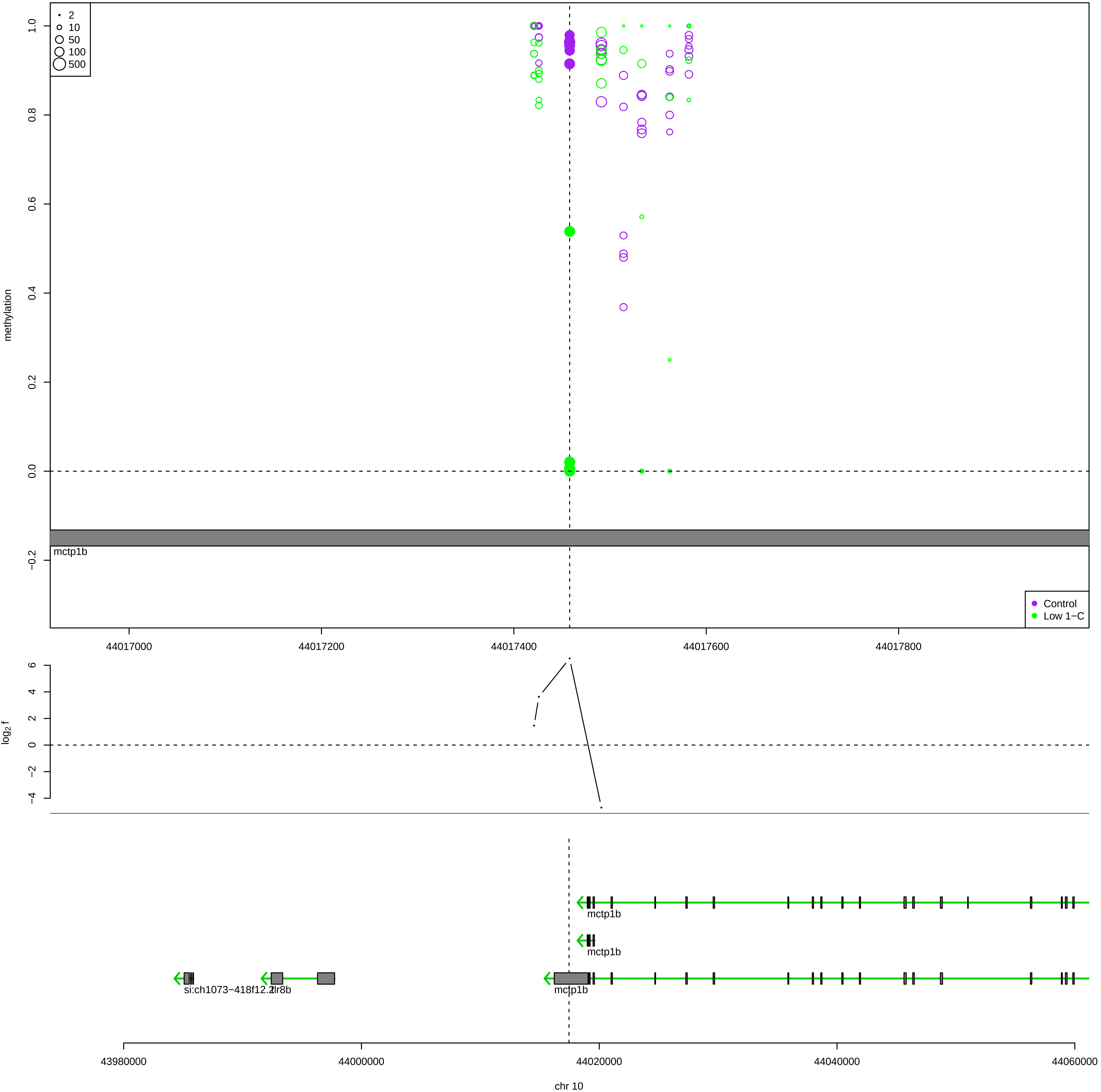

2
10
50
100
500
methylation
1.0
0.8
0.6
0.4
0.2
0.0
−0.2
mctp1b
Control
Low 1−C
44017000
44017200
44017400
44017600
44017800
log2 f
6
4
2
0
−2
−4
mctp1b
mctp1b
si:ch1073−418f12.2
tlr8b
mctp1b
43980000
44000000
44020000
44040000
44060000
chr 10

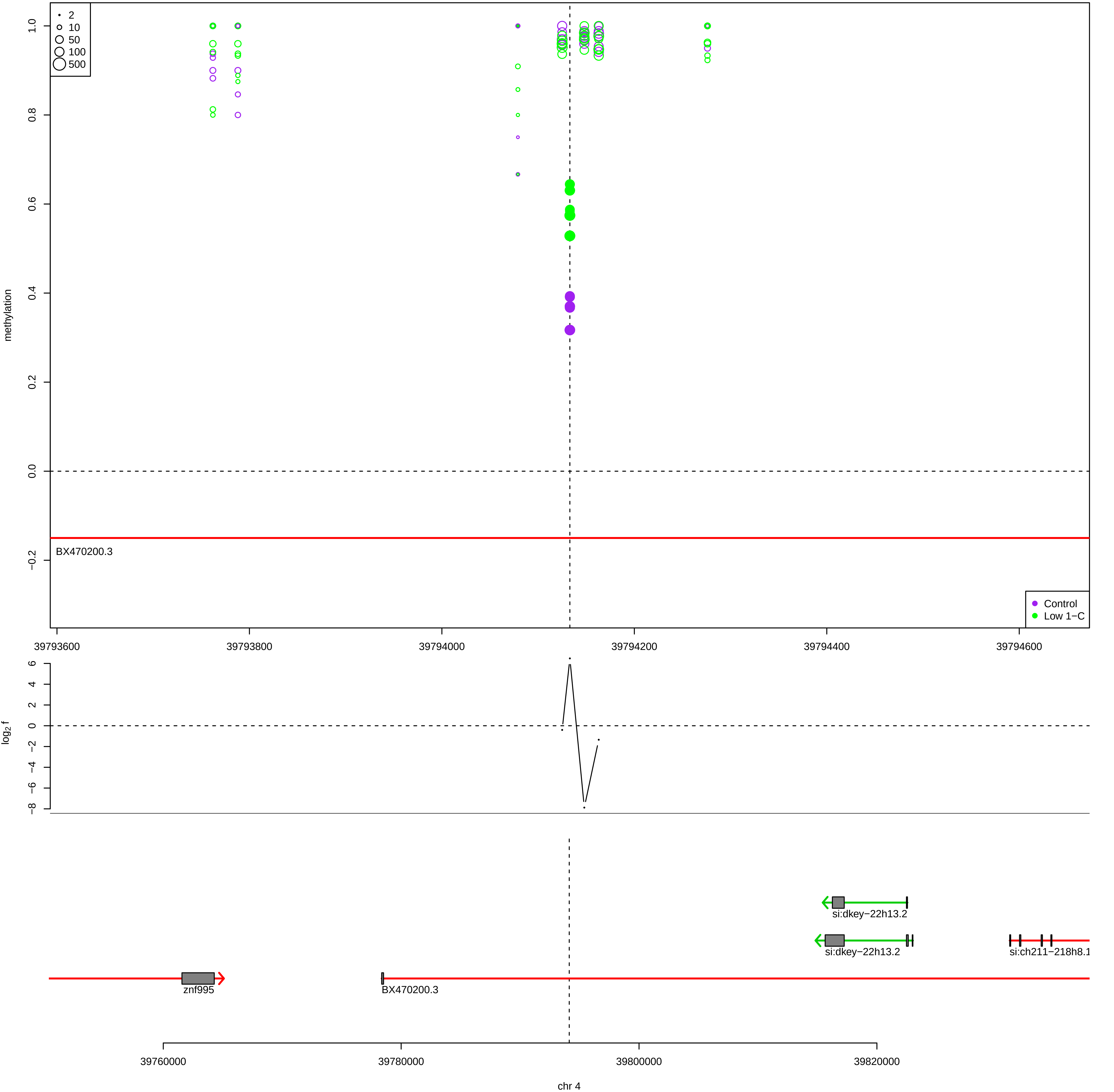

2
10
50
100
500
methylation
1.0
0.8
0.6
0.4
0.2
0.0
-0.2
BX470200.3
Control
Low 1−C
39793600
39793800
39794000
39794200
39794400
39794600
log2 f
6
4
2
0
−2
−4
−6
−8
si:dkey−22h13.2
si:dkey−22h13.2
si:ch211−218h8.1
znf995
BX470200.3
39760000
39780000
39800000
39820000
chr 4

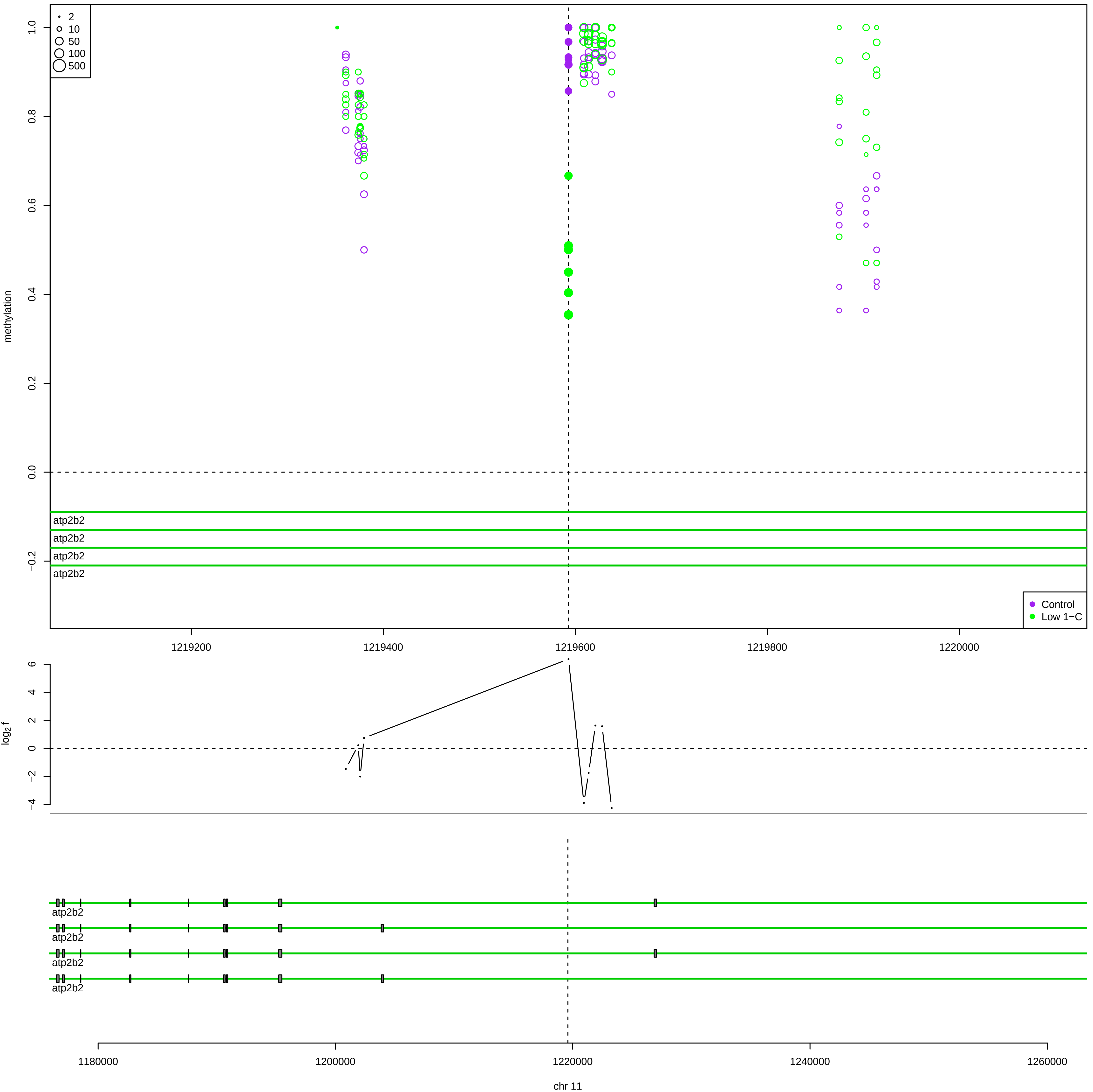

methylation
2
10
50
100
500
Control
Low 1−C
atp2b2
atp2b2
atp2b2
atp2b2
1219200
1219400
1219600
1219800
1220000
$\log_2 f$
atp2b2
atp2b2
atp2b2
atp2b2
1180000
1200000
1220000
1240000
1260000
chr 11

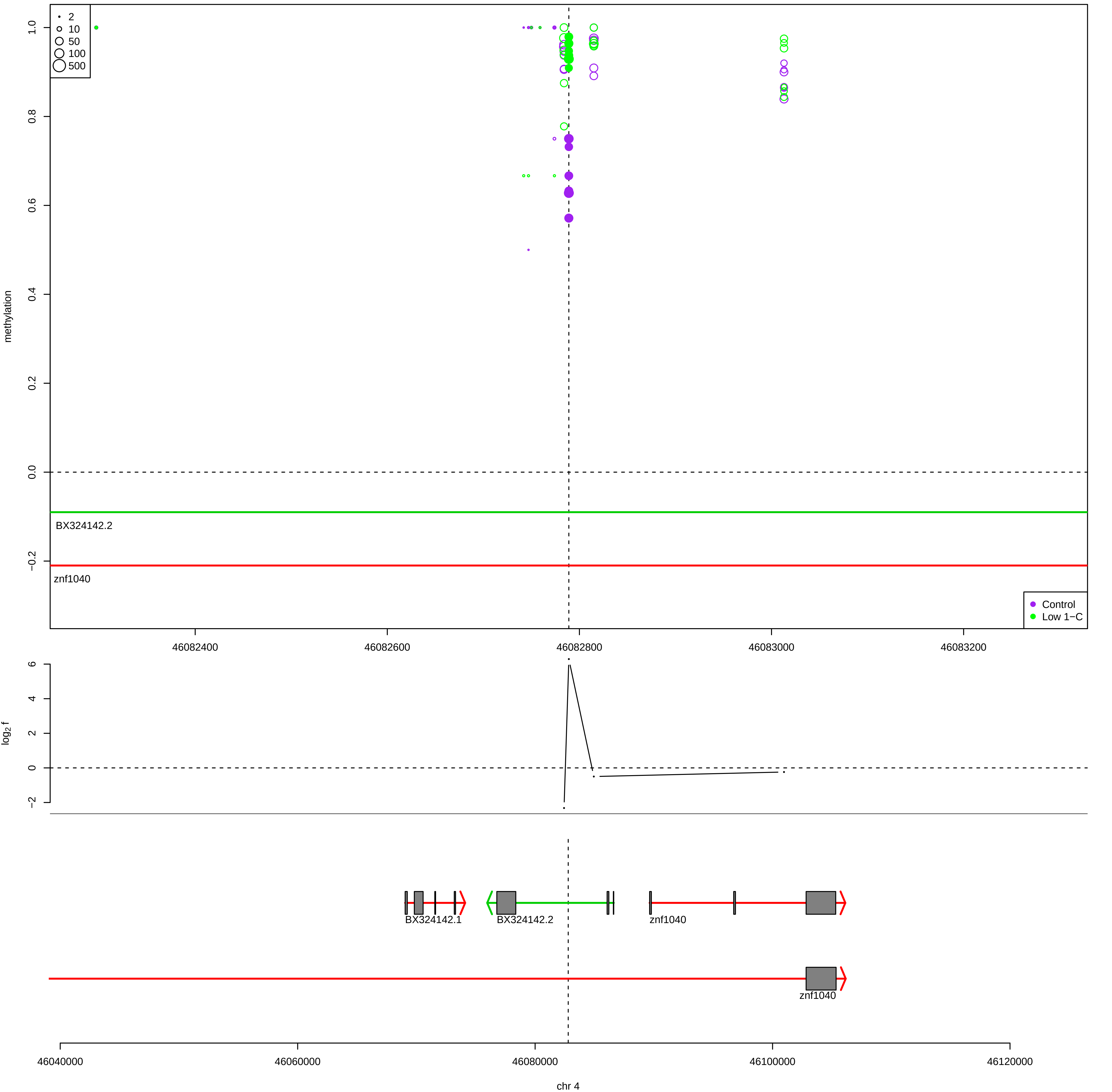

2
10
50
100
500
methylation
1.0
0.8
0.6
0.4
0.2
0.0
−0.2
BX324142.2
znf1040
Control
Low 1−C
46082400
46082600
46082800
46083000
46083200
log2 f
6
4
2
0
−2
BX324142.1
BX324142.2
znf1040
znf1040
46040000
46060000
46080000
46100000
46120000
chr 4

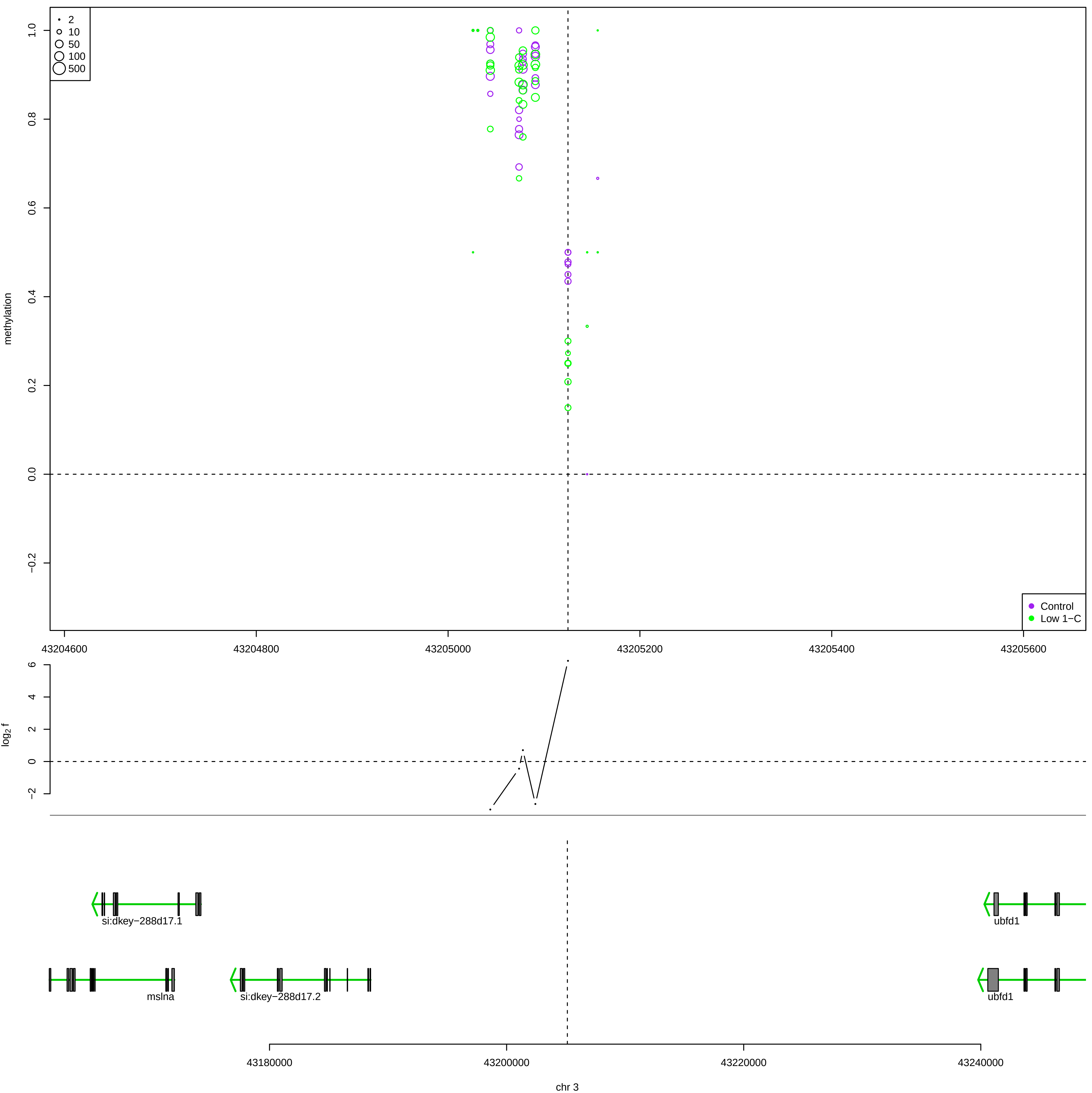

methylation
1.0
0.8
0.6
0.4
0.2
0.0
−0.2
2
10
50
100
500
Control
Low 1−C
43204600
43204800
43205000
43205200
43205400
43205600
log2 f
6
4
2
0
−2
si:dkey−288d17.1
ubfd1
mslna
si:dkey−288d17.2
ubfd1
43180000
43200000
43220000
43240000
chr 3

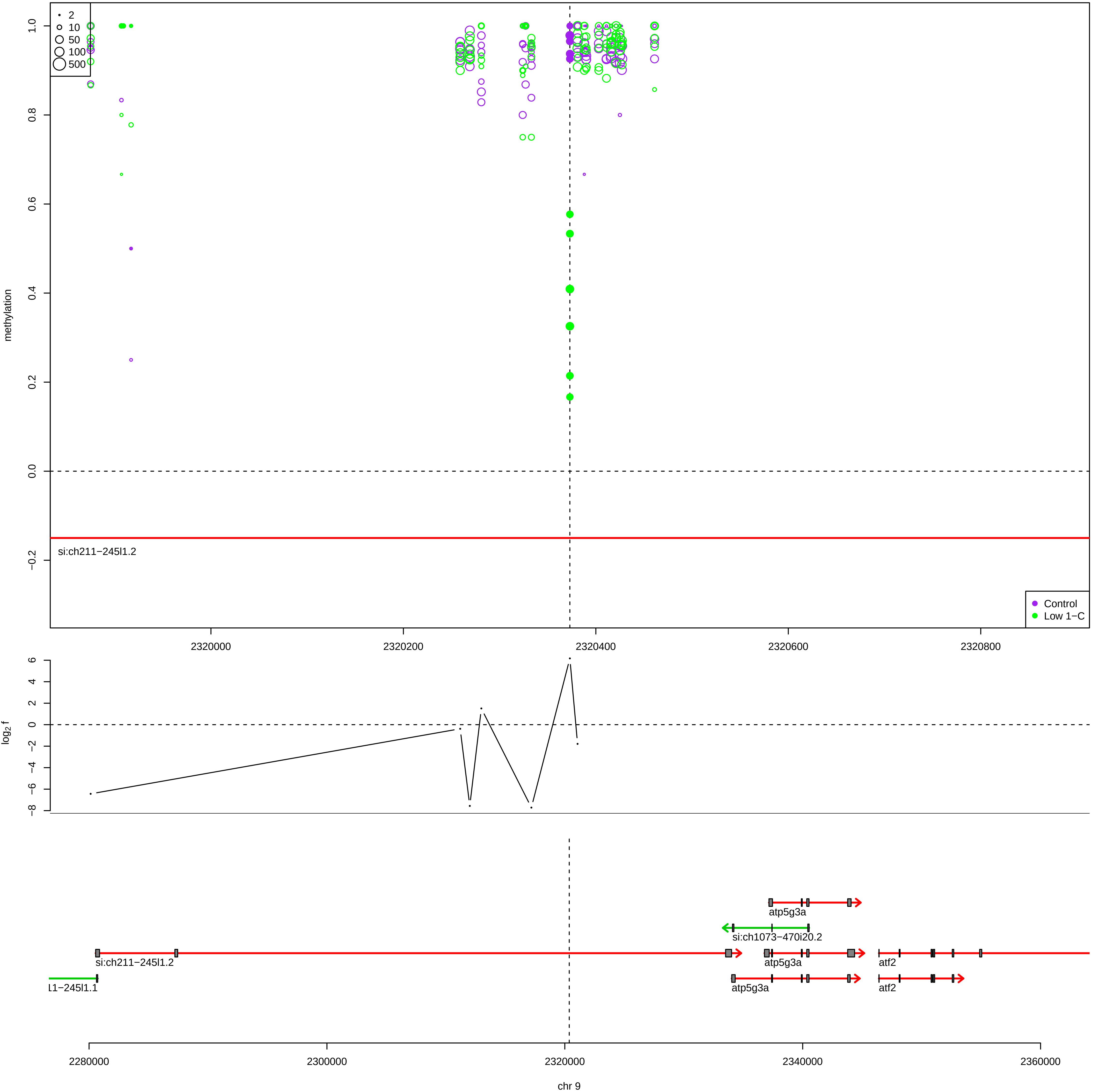

2
10
50
100
500
methylation
1.0
0.8
0.6
0.4
0.2
0.0
−0.2
si:ch211−245l1.2
Control
Low 1−C
2320000
2320200
2320400
2320600
2320800
log2 f
6
4
2
0
−2
−4
−6
−8
atp5g3a
si:ch1073−470i20.2
si:ch211−245l1.2
atp5g3a
atf2
l1−245l1.1
atp5g3a
atf2
2280000
2300000
2320000
2340000
2360000
chr 9

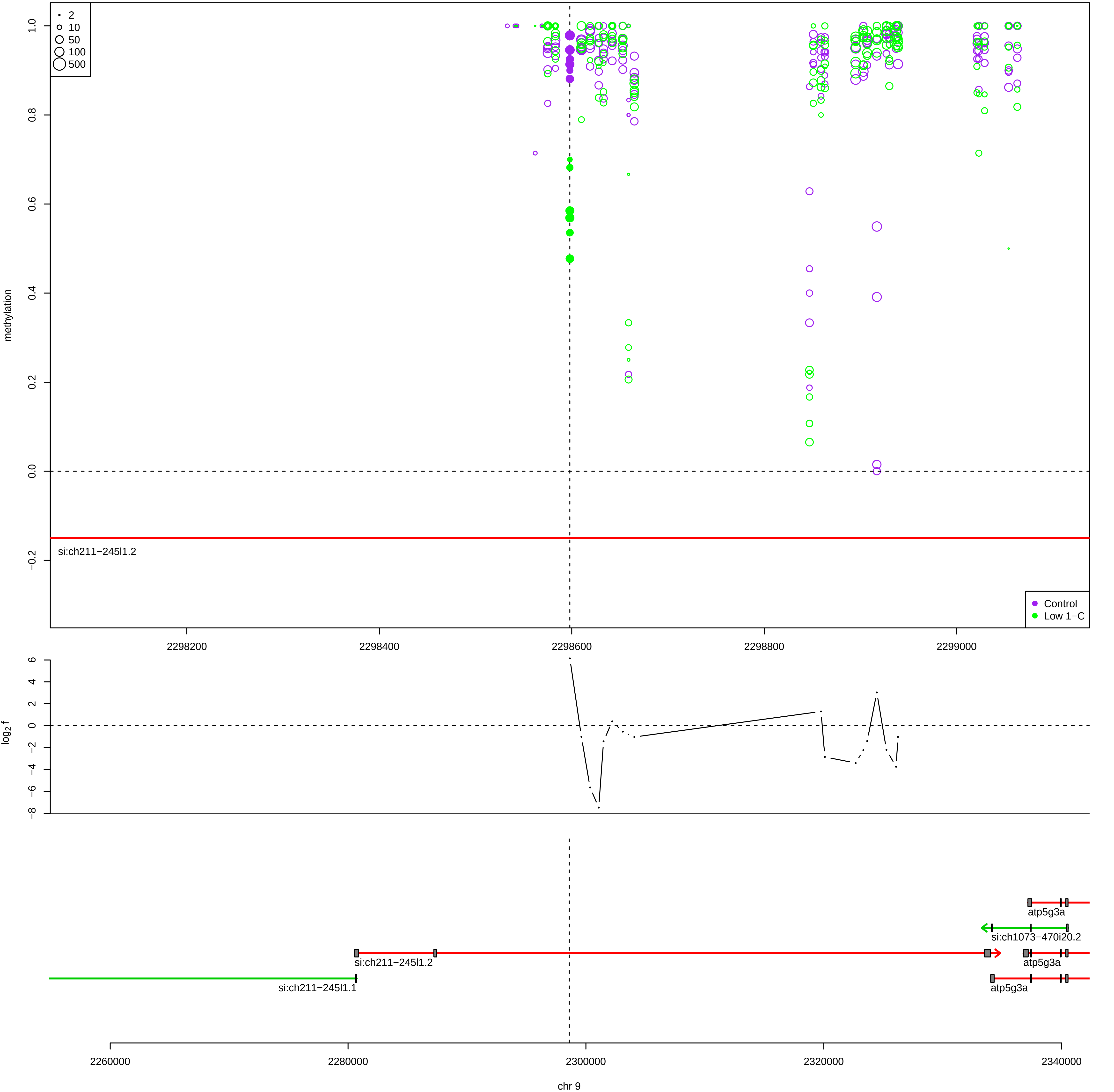

2
10
50
100
500
methylation
1.0
0.8
0.6
0.4
0.2
0.0
−0.2
si:ch211−245l1.2
Control
Low 1−C
2298200
2298400
2298600
2298800
2299000
log2 f
6
4
2
0
−2
−4
−6
−8
atp5g3a
si:ch1073−470i20.2
si:ch211−245l1.2
atp5g3a
si:ch211−245l1.1
atp5g3a
2260000
2280000
2300000
2320000
2340000
chr 9

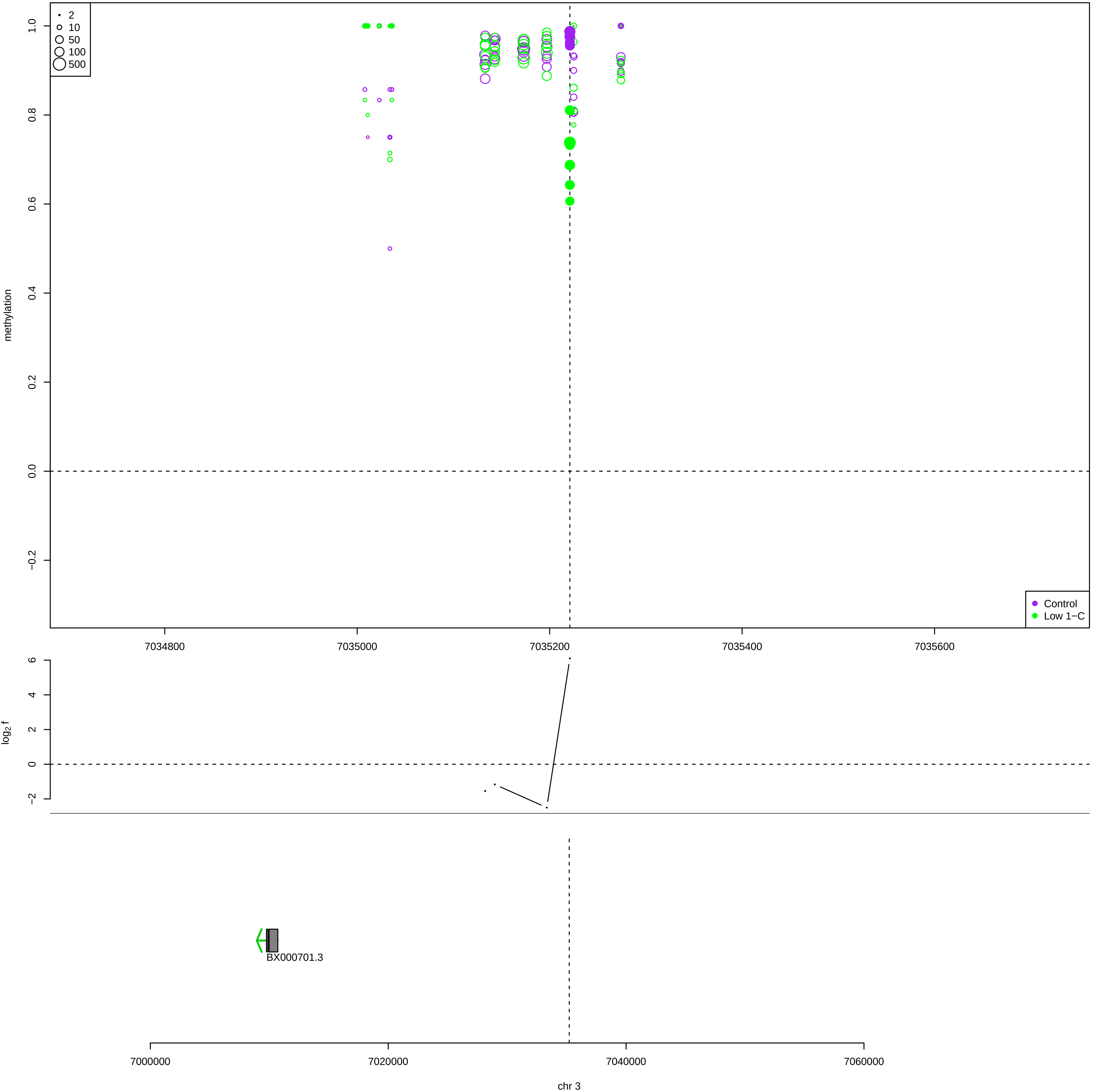

2
10
50
100
500
Control
Low 1−C
methylation
1.0
0.8
0.6
0.4
0.2
0.0
−0.2
7034800
7035000
7035200
7035400
7035600
$log_2 f$
6
4
2
0
−2
BX000701.3
7000000
7020000
7040000
7060000
chr 3

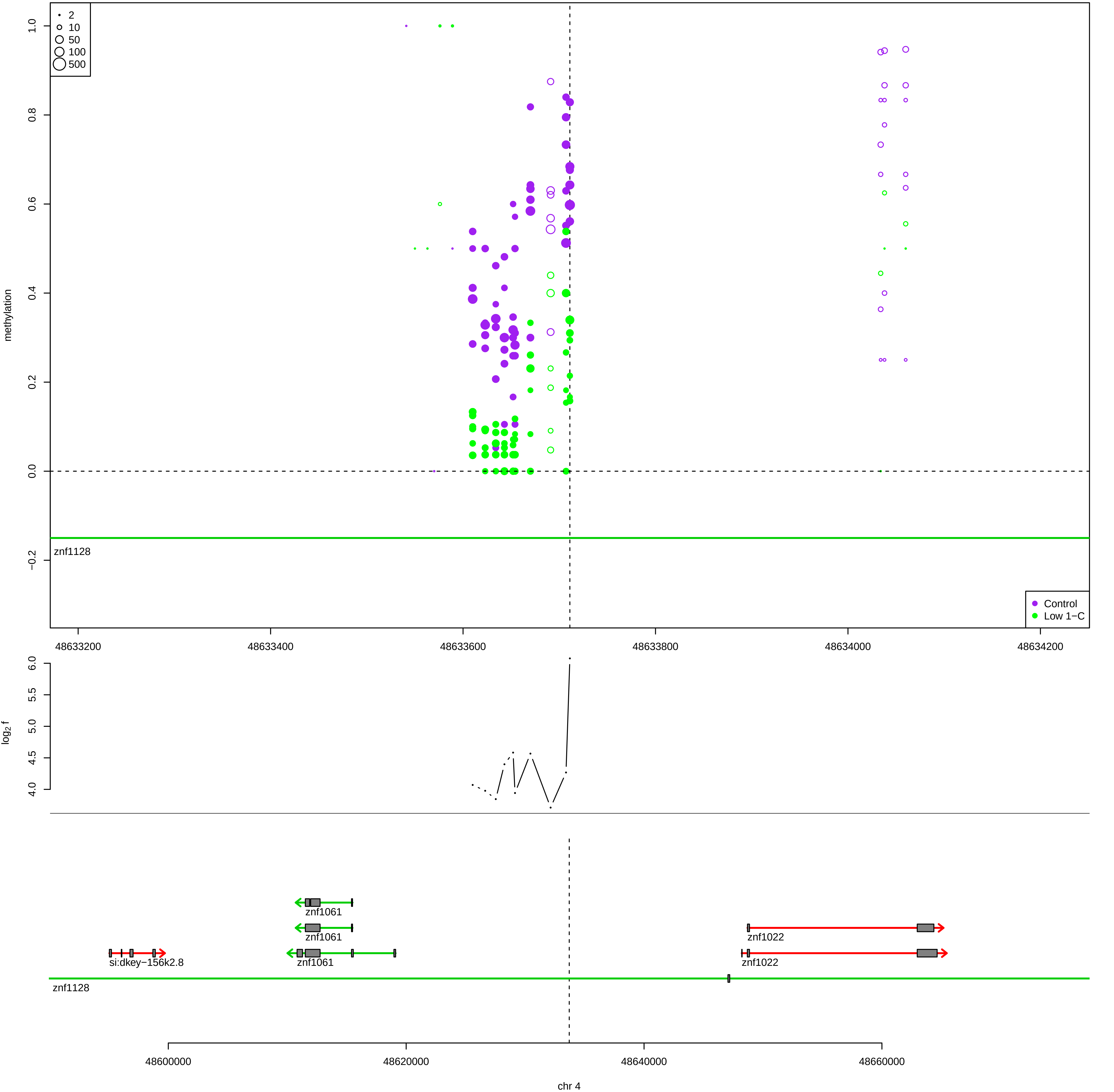

2
10
50
100
500
methylation
1.0
0.8
0.6
0.4
0.2
0.0
-0.2
znf1128
Control
Low 1−C
48633200
48633400
48633600
48633800
48634000
48634200
log2 f
6.0
5.5
5.0
4.5
4.0
znf1061
znf1061
znf1061
znf1022
znf1022
si:dkey−156k2.8
znf1128
48600000
48620000
48640000
48660000
chr 4

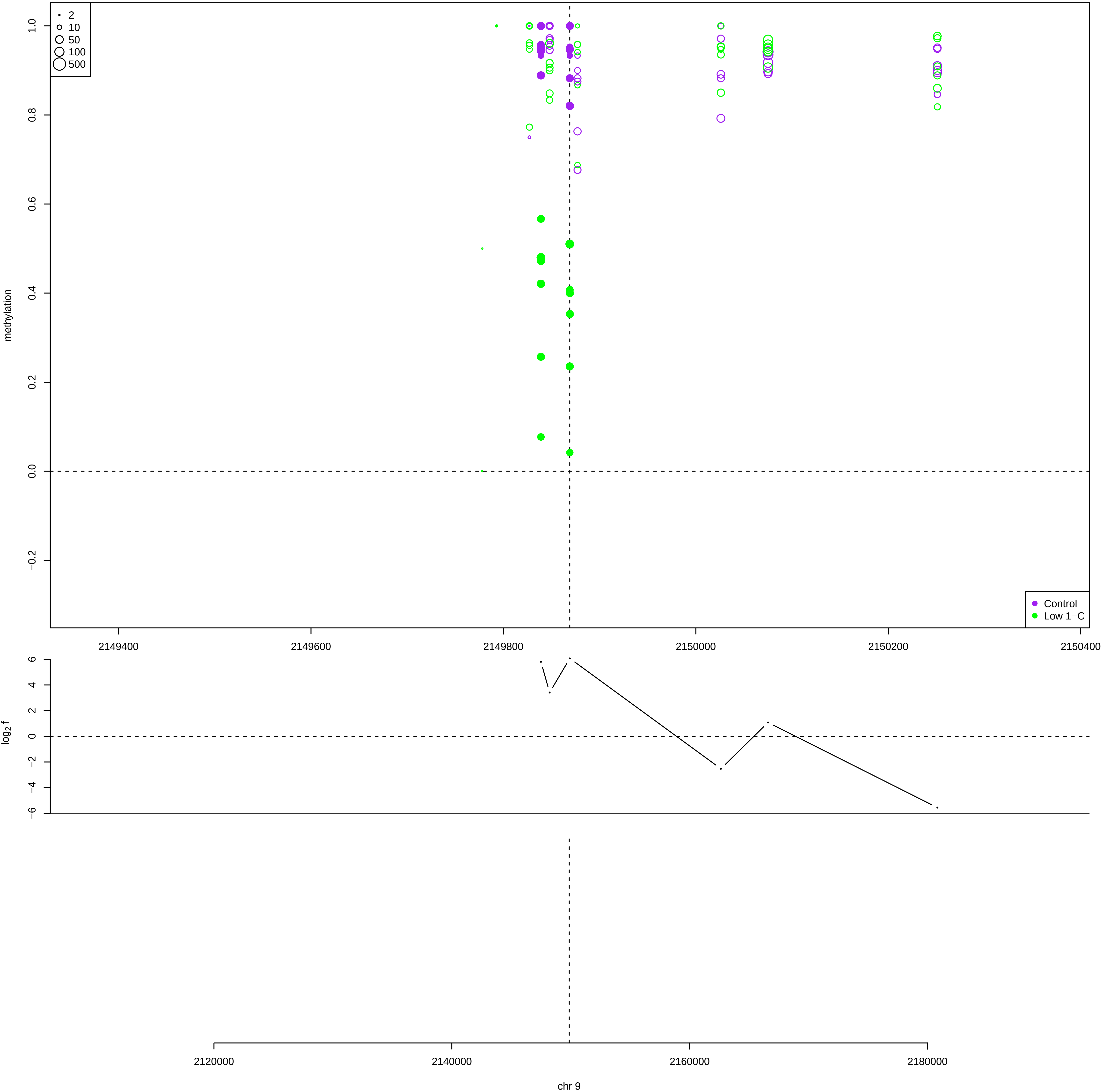

2
10
50
100
500
methylation
1.0
0.8
0.6
0.4
0.2
0.0
−0.2
Control
Low 1−C
2149400
2149600
2149800
2150000
2150200
2150400
log2 f
6
4
2
0
−2
−4
−6
2120000
2140000
2160000
2180000
chr 9

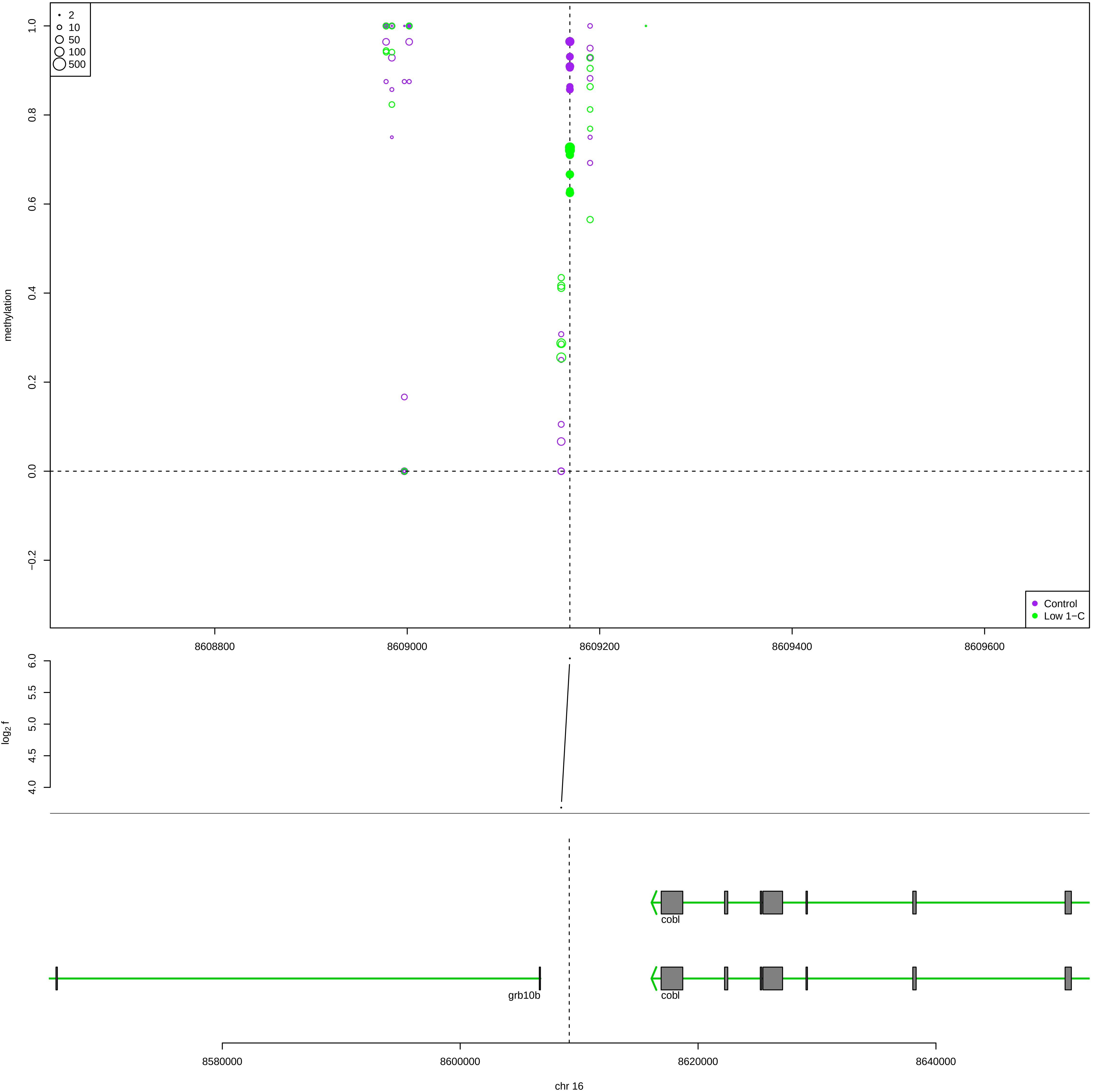

2
10
50
100
500
Control
Low 1−C
methylation
1.0
0.8
0.6
0.4
0.2
0.0
−0.2
8608800
8609000
8609200
8609400
8609600
log2 f
6.0
5.5
5.0
4.5
4.0
cobl
grb10b
cobl
8580000
8600000
8620000
8640000
chr 16

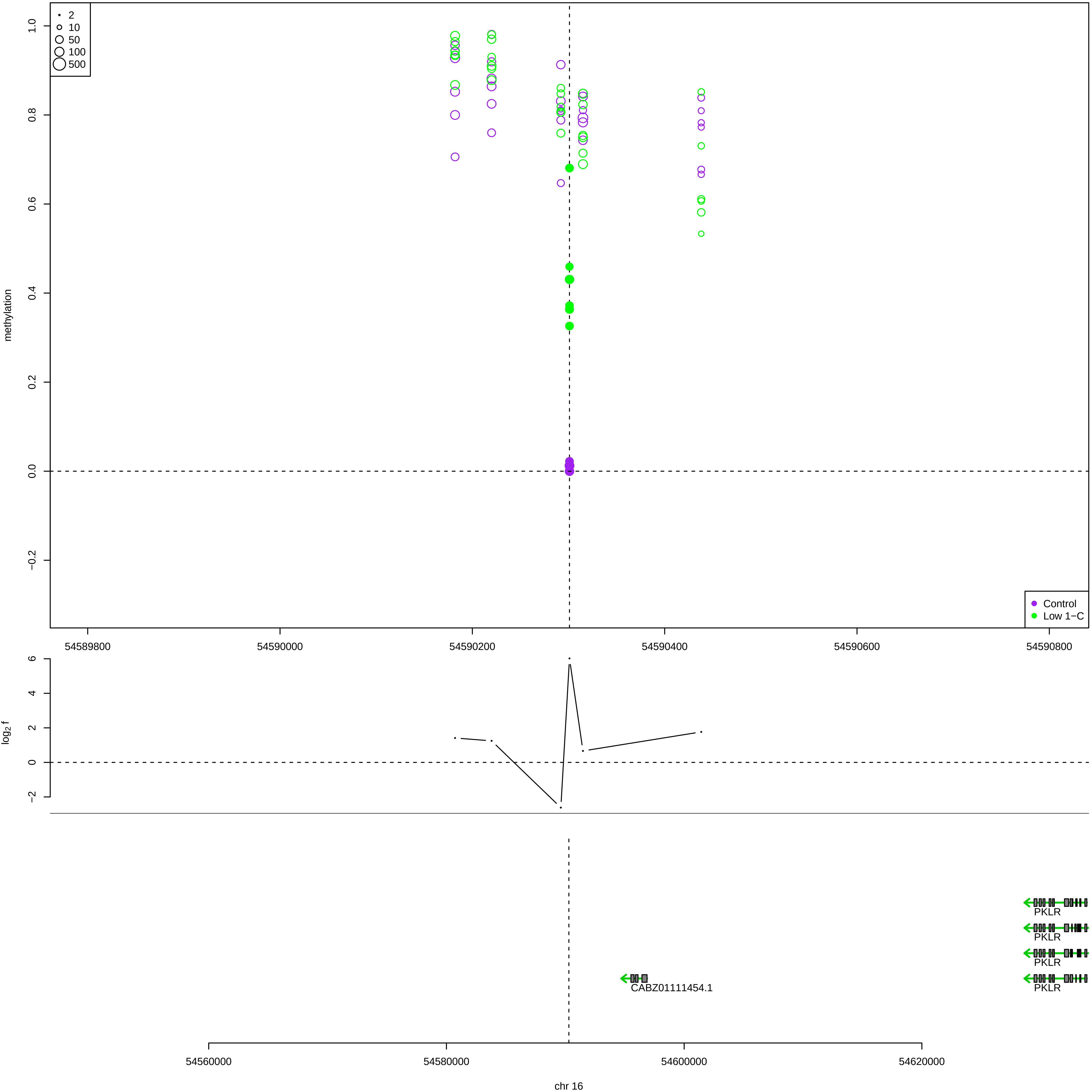

2
10
50
100
500
methylation
1.0
0.8
0.6
0.4
0.2
0.0
−0.2
Control
Low 1−C
54589800
54590000
54590200
54590400
54590600
54590800
log2 f
6
4
2
0
−2
PKLR
PKLR
PKLR
PKLR
CABZ01111454.1
54560000
54580000
54600000
54620000
chr 16

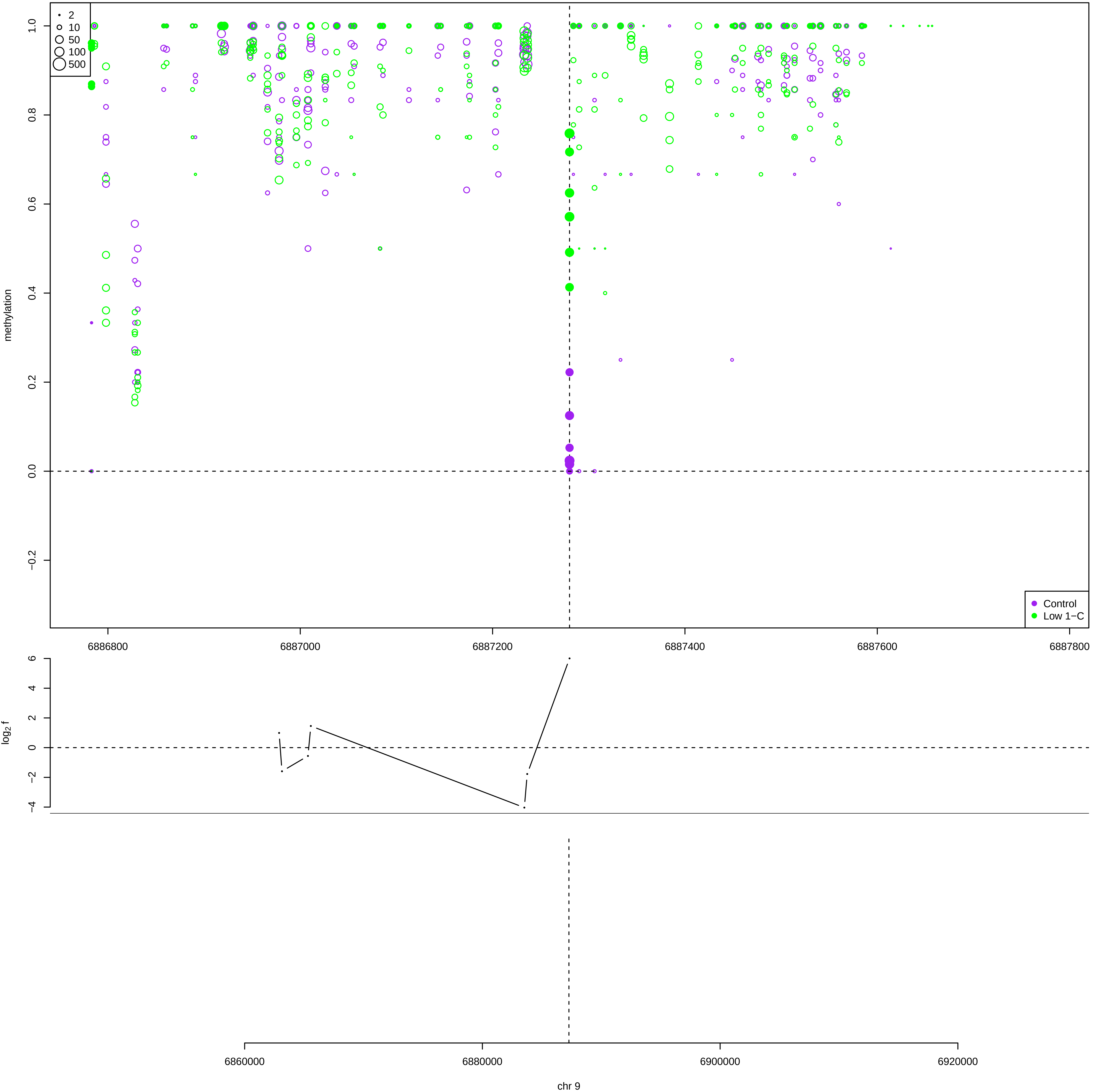

2
10
50
100
500
1.0
0.8
0.6
0.4
0.2
0.0
−0.2
methylation
Control
Low 1−C
6886800
6887000
6887200
6887400
6887600
6887800
6
4
2
0
−2
−4
log2 f
6860000
6880000
6900000
6920000
chr 9

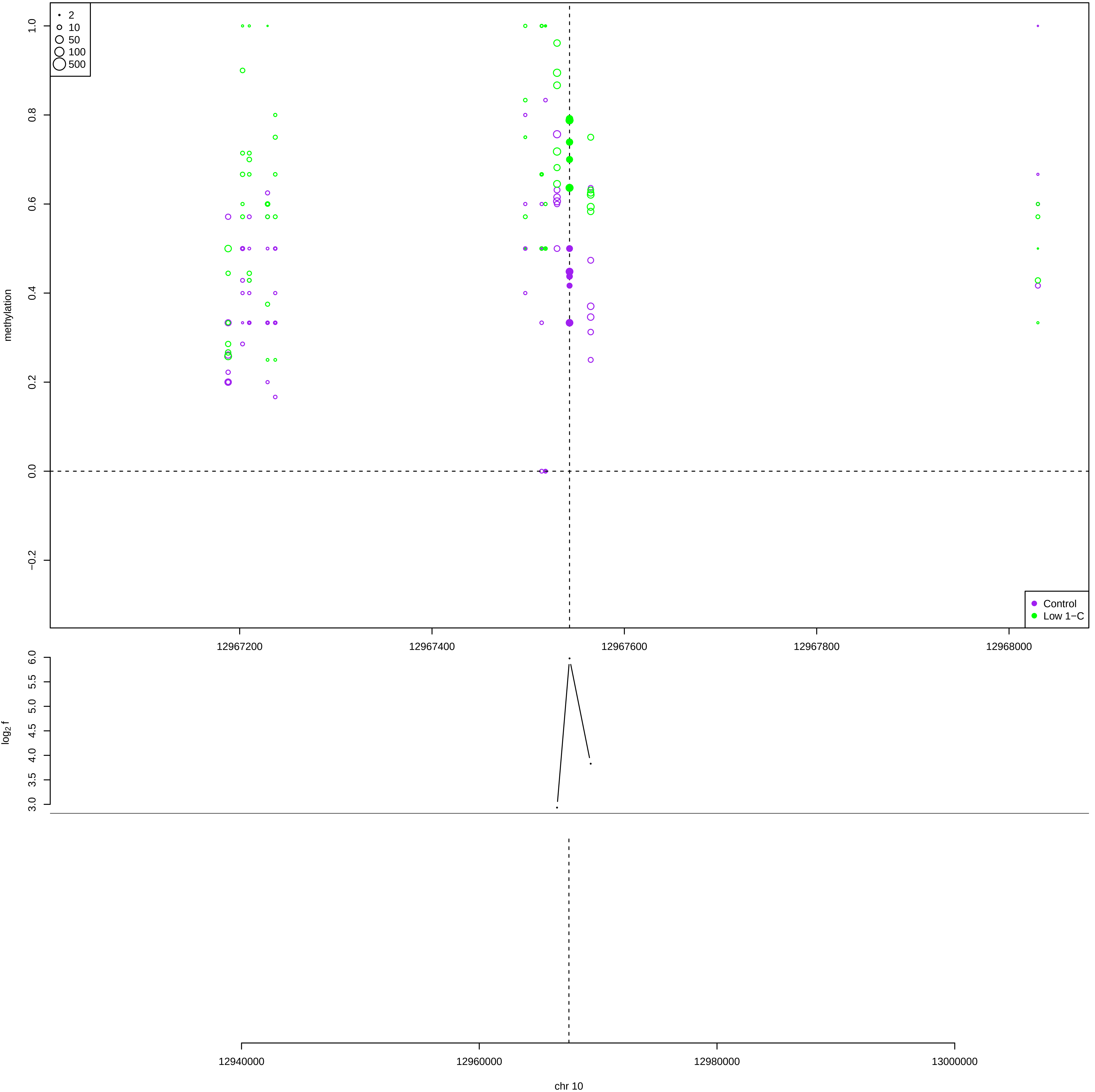

2
10
50
100
500
Control
Low 1−C
methylation
1.0
0.8
0.6
0.4
0.2
0.0
−0.2
12967200
12967400
12967600
12967800
12968000
log2 f
6.0
5.5
5.0
4.5
4.0
3.5
3.0
12940000
12960000
12980000
13000000
chr 10

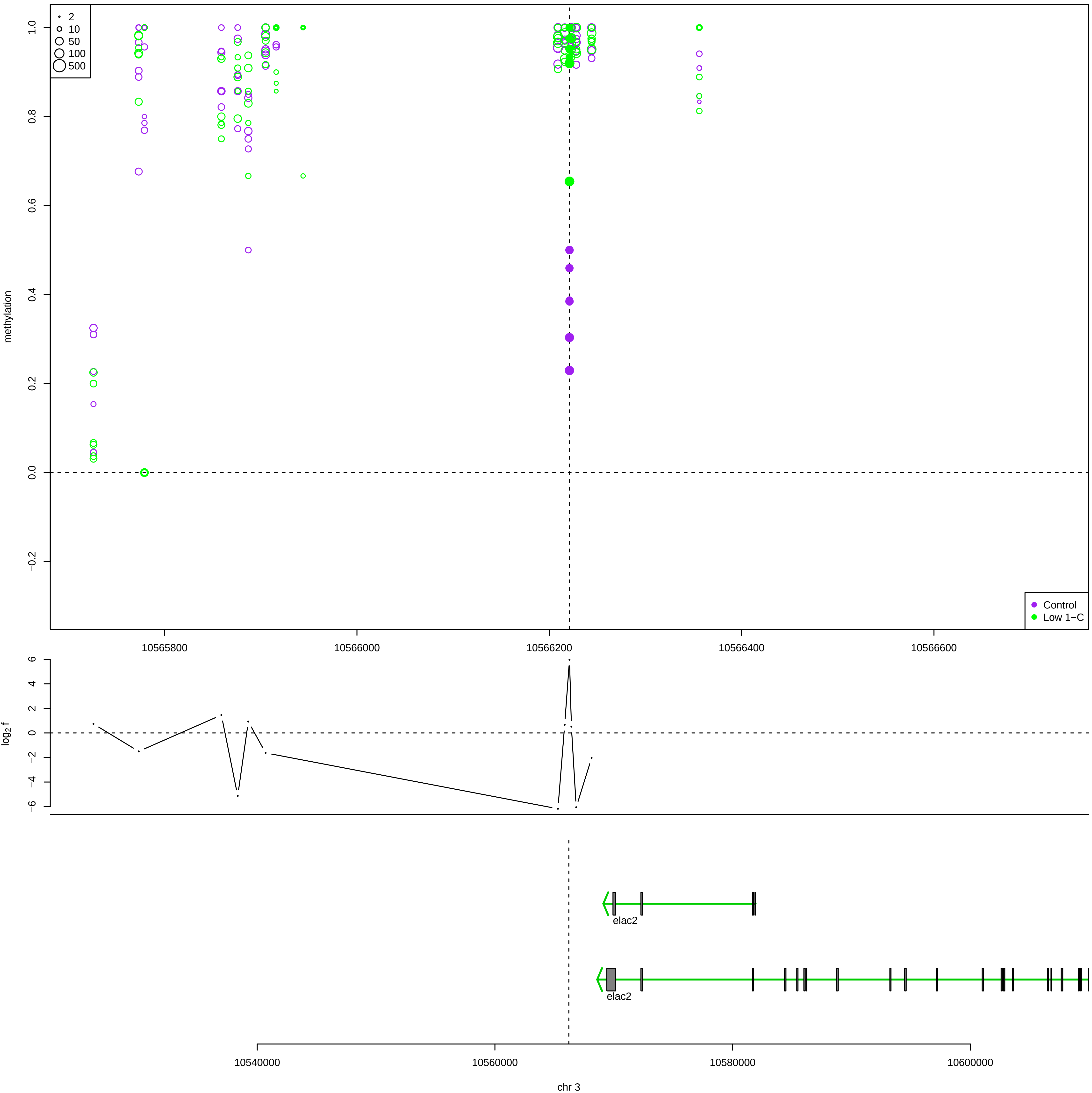

methylation
2
10
50
100
500
Control
Low 1−C
1.0
0.8
0.6
0.4
0.2
0.0
−0.2
10565800
10566000
10566200
10566400
10566600
log2 f
6
4
2
0
−2
−4
−6
elac2
elac2
10540000
10560000
10580000
10600000
chr 3

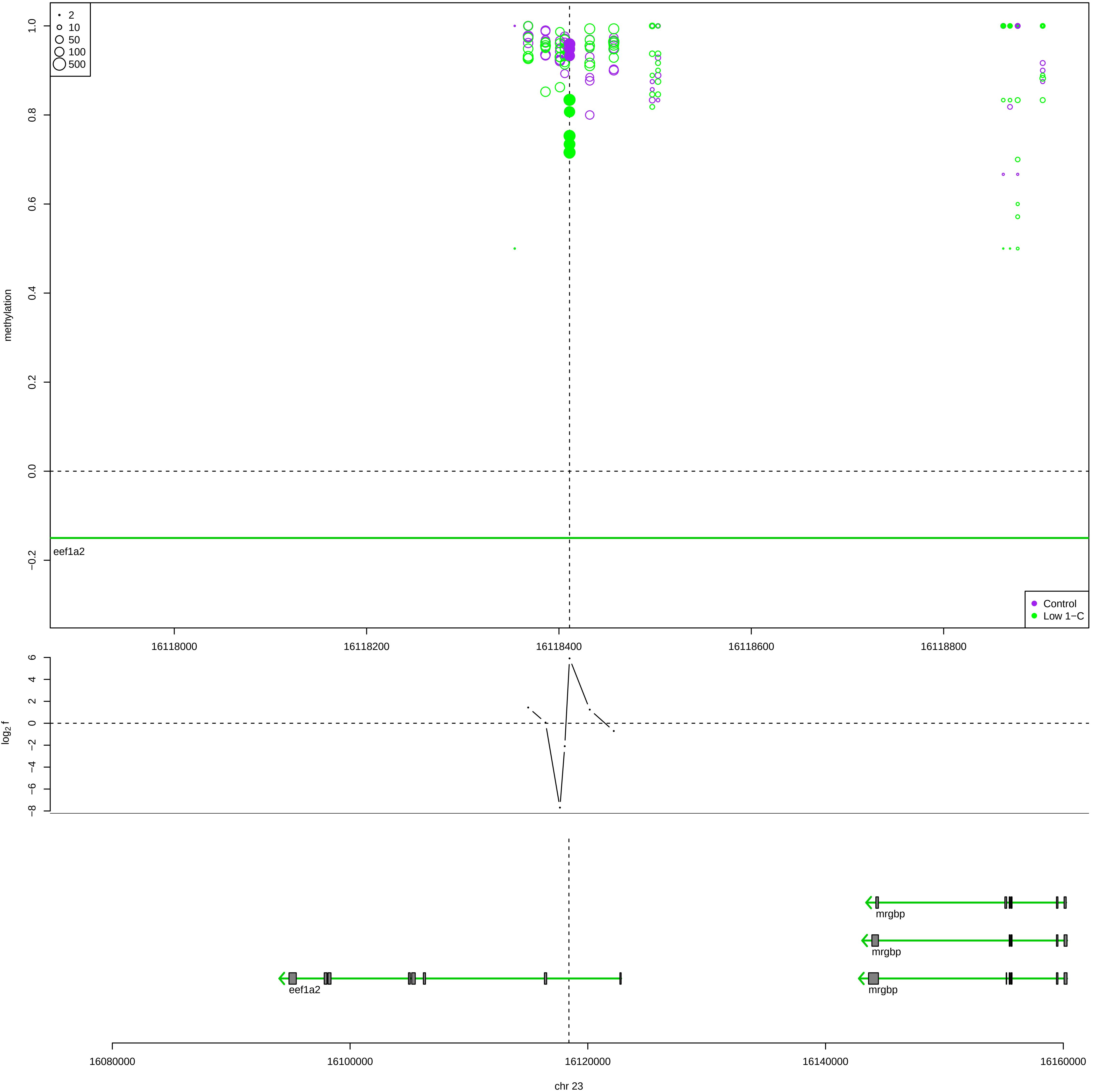

2
10
50
100
500
methylation
1.0
0.8
0.6
0.4
0.2
0.0
−0.2
eef1a2
Control
Low 1−C
16118000
16118200
16118400
16118600
16118800
$\log_2 f$
6
4
2
0
−2
−4
−6
−8
mrgbp
mrgbp
eef1a2
mrgbp
16080000
16100000
16120000
16140000
16160000
chr 23

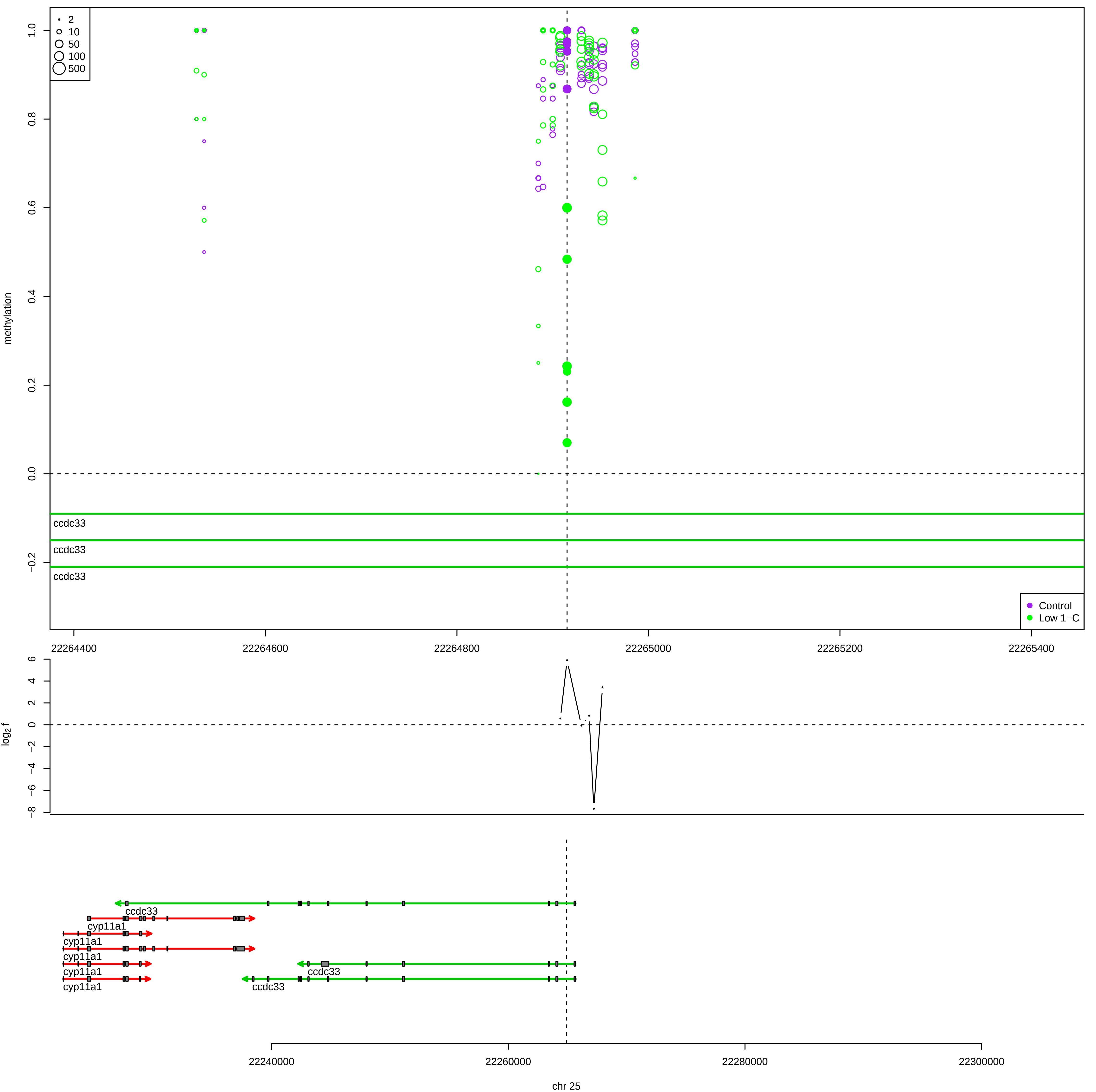

methylation
2
10
50
100
500
ccdc33
ccdc33
ccdc33
Control
Low 1−C
22264400
22264600
22264800
22265000
22265200
22265400
log2 f
ccdc33
cyp11a1
cyp11a1
cyp11a1
cdc33
cyp11a1
ccdc33
cyp11a1
22240000
22260000
22280000
22300000
chr 25

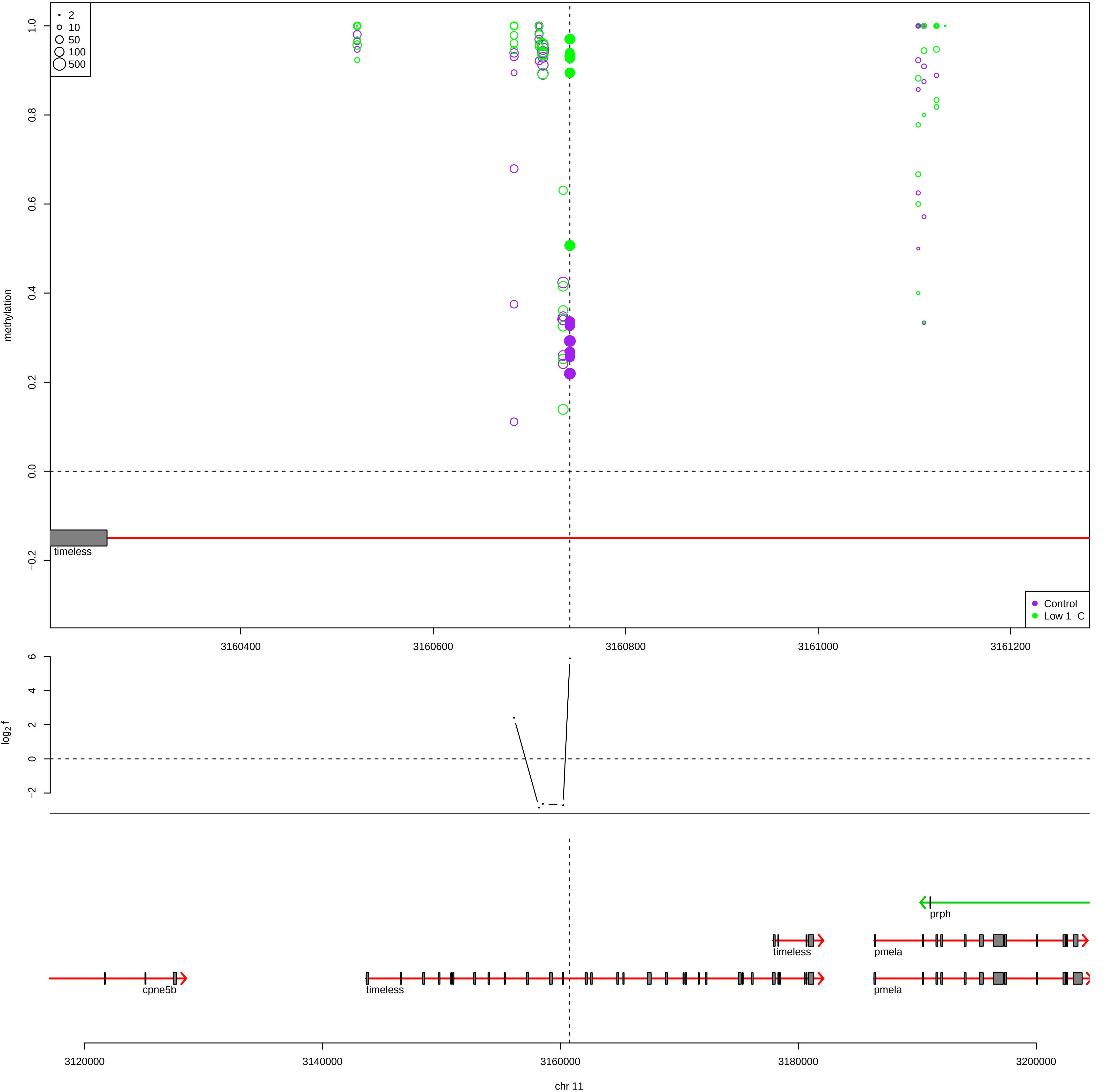

methylation
1.0
0.8
0.6
0.4
0.2
0.0
−0.2
2
10
50
100
500
timeless
Control
Low 1−C
3160400
3160600
3160800
3161000
3161200
log2 f
6
4
2
0
−2
prph
timeless
pmela
cpne5b
timeless
pmela
3120000
3140000
3160000
3180000
3200000
chr 11

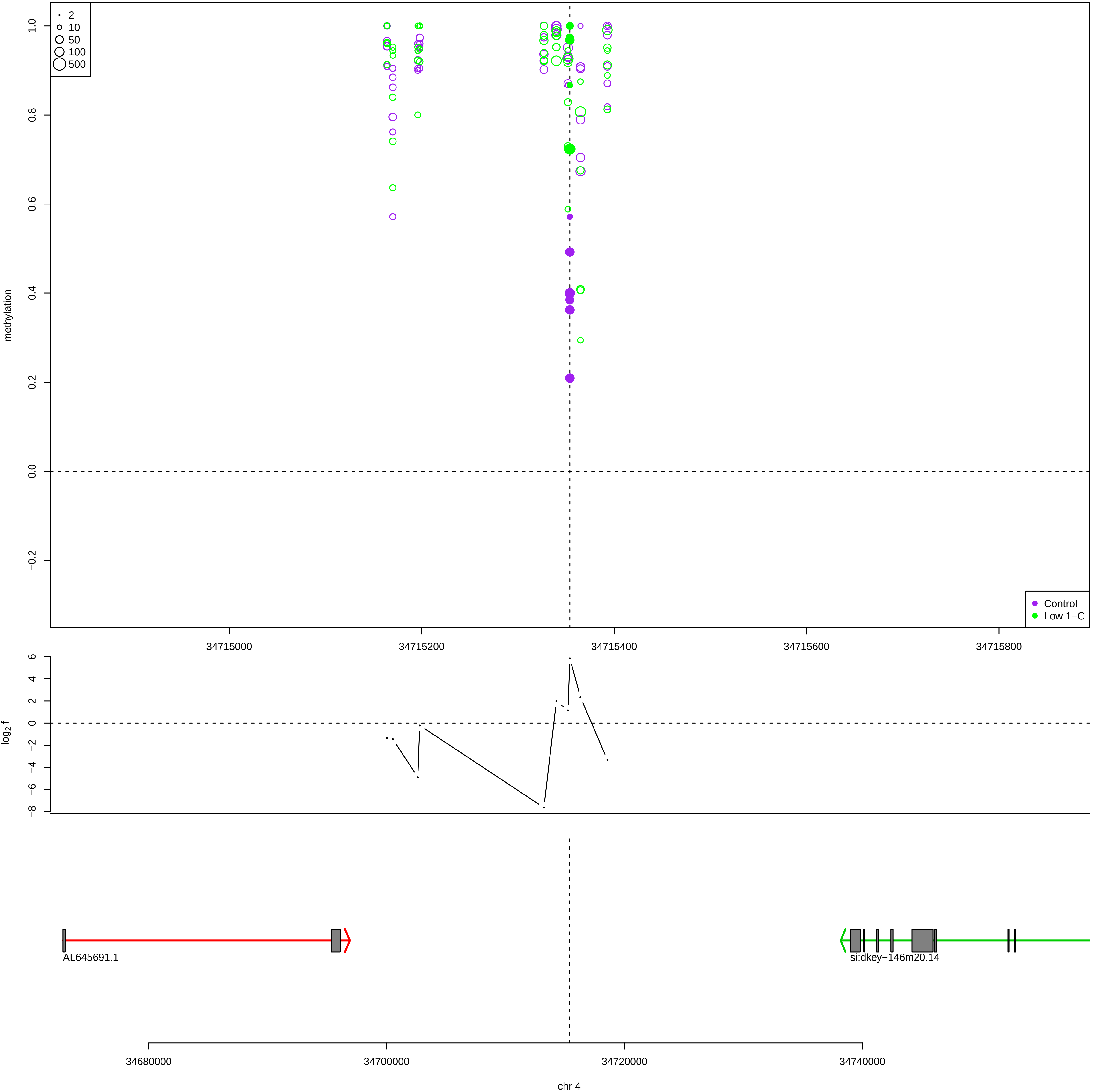

methylation
1.0
0.8
0.6
0.4
0.2
0.0
−0.2
2
10
50
100
500
Control
Low 1−C
34715000
34715200
34715400
34715600
34715800
log2 f
6
4
2
0
−2
−4
−6
−8
AL645691.1
si:dkey−146m20.14
34680000
34700000
34720000
34740000
chr 4

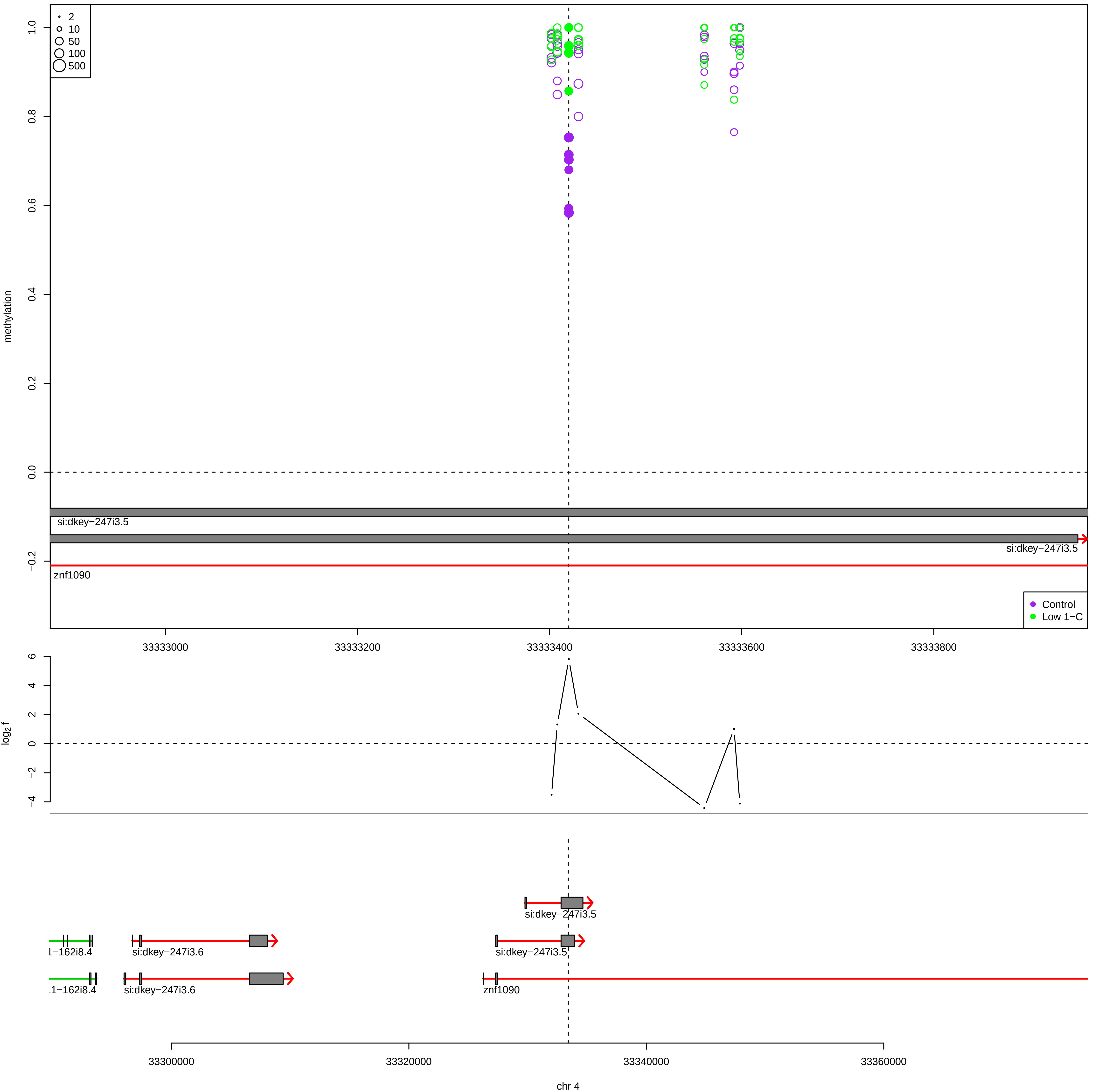

2
10
50
100
500
methylation
1.0
0.8
0.6
0.4
0.2
0.0
−0.2
si:dkey−247i3.5
si:dkey−247i3.5
znf1090
Control
Low 1−C
33333000
33333200
33333400
33333600
33333800
6
4
2
0
−2
−4
log2 f
1−162i8.4
si:dkey−247i3.6
si:dkey−247i3.5
si:dkey−247i3.5
.1−162i8.4
si:dkey−247i3.6
znf1090
33300000
33320000
33340000
33360000
chr 4

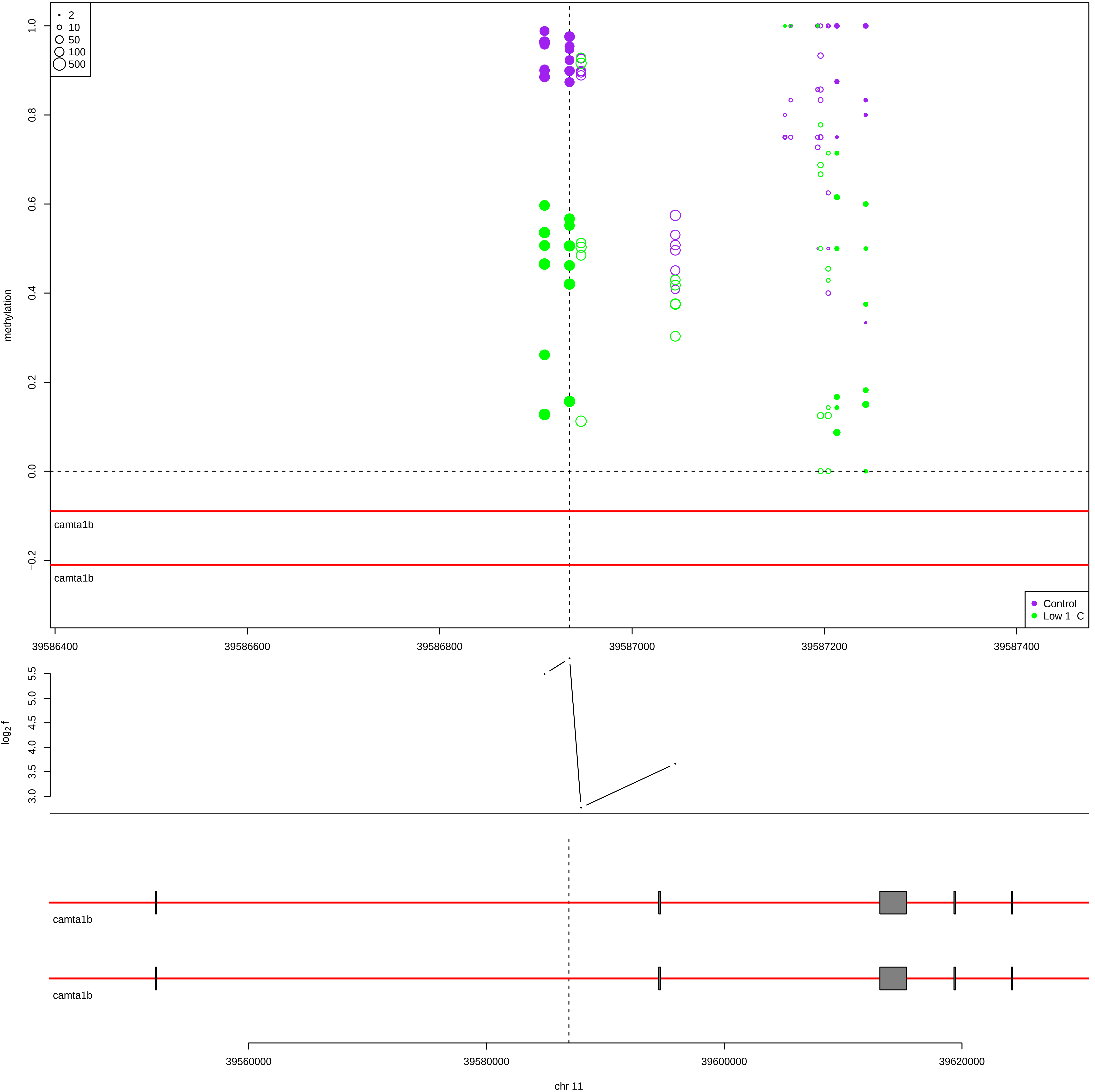

2
10
50
100
500
methylation
1.0
0.8
0.6
0.4
0.2
0.0
−0.2
camta1b
camta1b
Control
Low 1−C
39586400
39586600
39586800
39587000
39587200
39587400
log2 f
5.5
5.0
4.5
4.0
3.5
3.0
camta1b
camta1b
39560000
39580000
39600000
39620000
chr 11

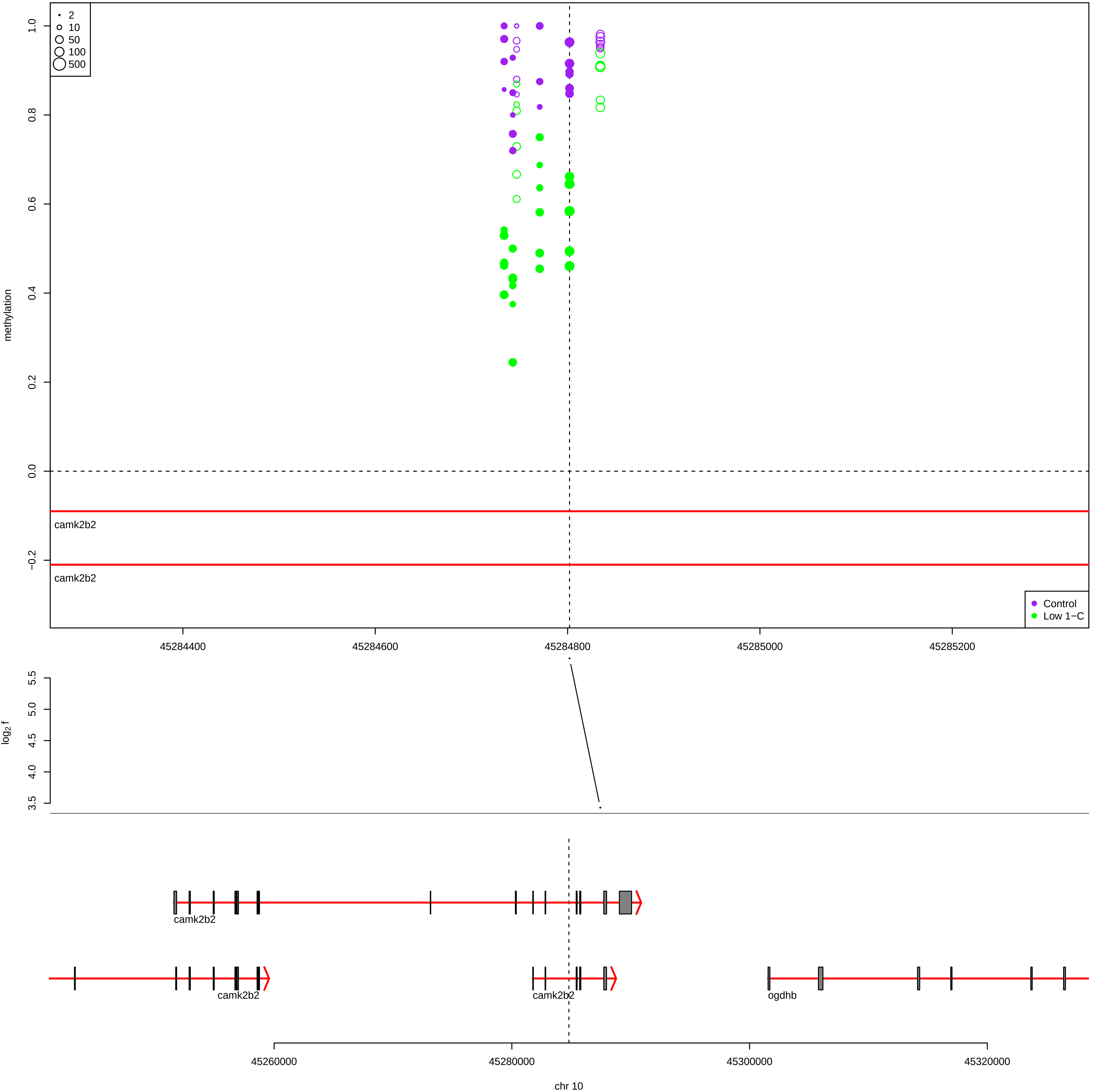

methylation
2
10
50
100
500
1.0
0.8
0.6
0.4
0.2
0.0
−0.2
camk2b2
camk2b2
Control
Low 1−C
45284400
45284600
45284800
45285000
45285200
log2 f
5.5
5.0
4.5
4.0
3.5
camk2b2
camk2b2
camk2b2
ogdhb
45260000
45280000
45300000
45320000
chr 10

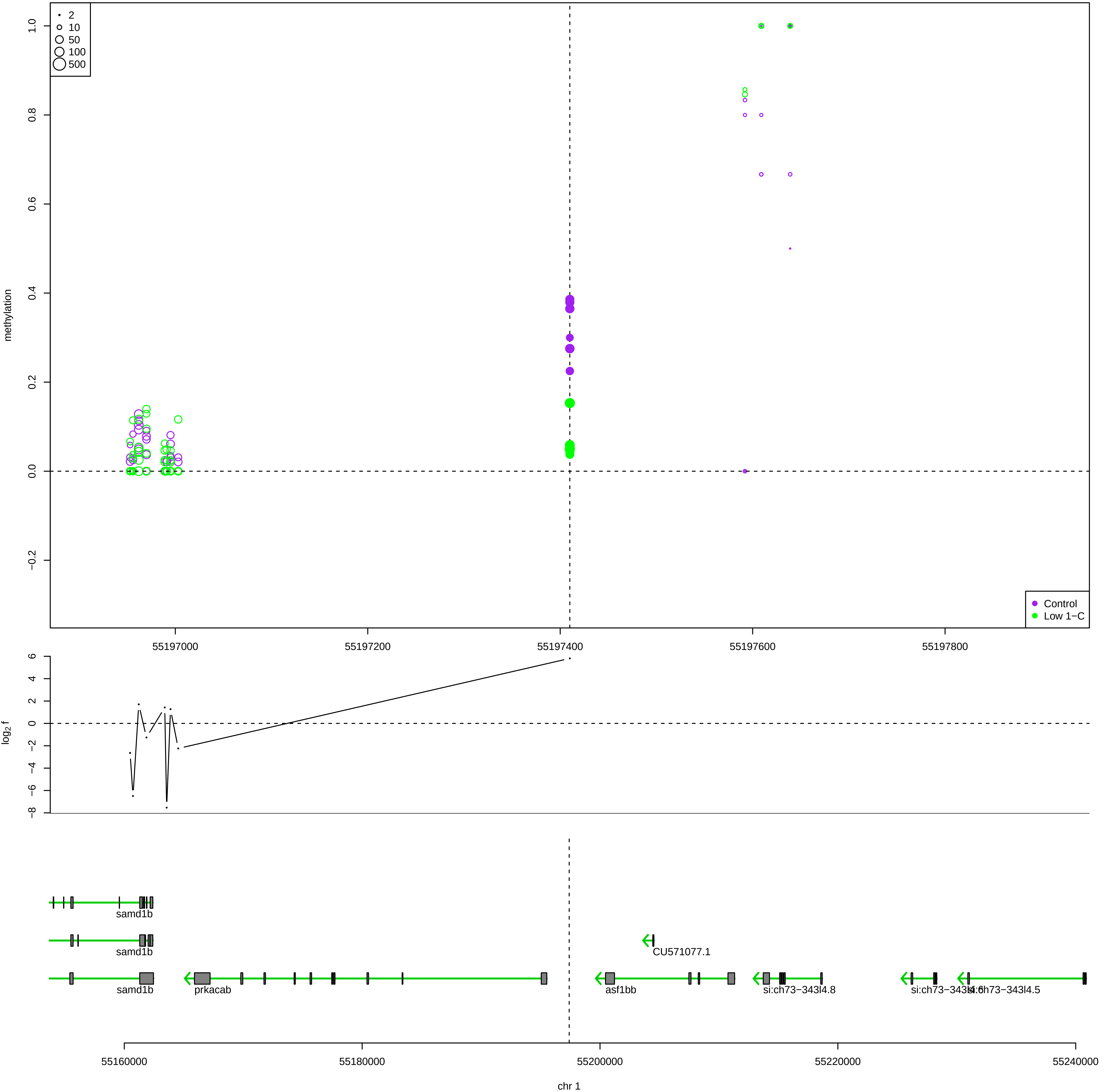

methylation
1.0
0.8
0.6
0.4
0.2
0.0
−0.2
2
10
50
100
500
Control
Low 1−C
55197000
55197200
55197400
55197600
55197800
log2 f
6
4
2
0
−2
−4
−6
−8
samd1b
samd1b
samd1b
prkacab
CU571077.1
asf1bb
si:ch73−343l4.8
si:ch73−343l4.6
si:ch73−343l4.5
55160000
55180000
55200000
55220000
55240000
chr 1

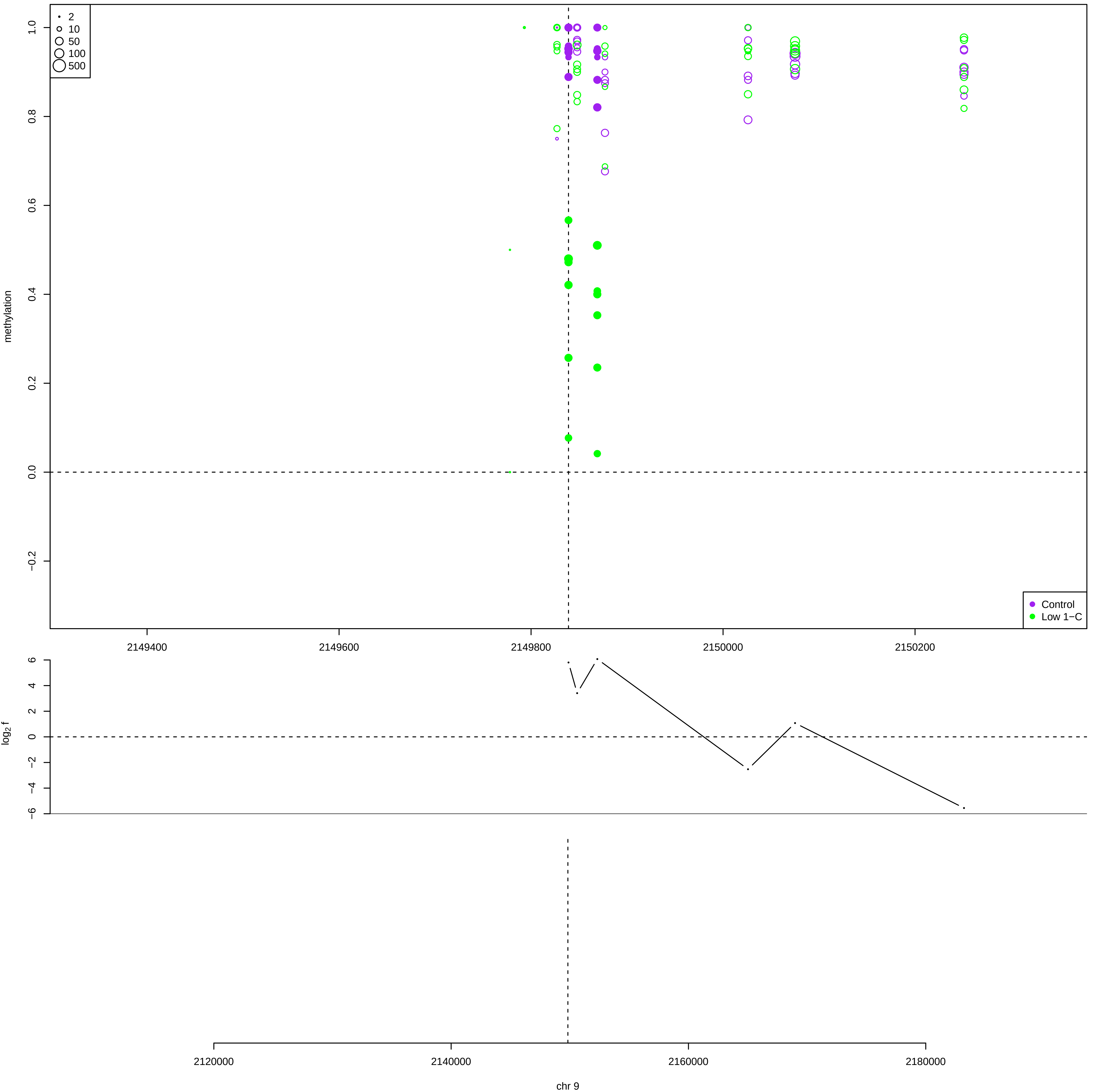

methylation
2
10
50
100
500
Control
Low 1−C
1.0
0.8
0.6
0.4
0.2
0.0
−0.2
2149400
2149600
2149800
2150000
2150200
log2 f
6
4
2
0
−2
−4
−6
2120000
2140000
2160000
2180000
chr 9

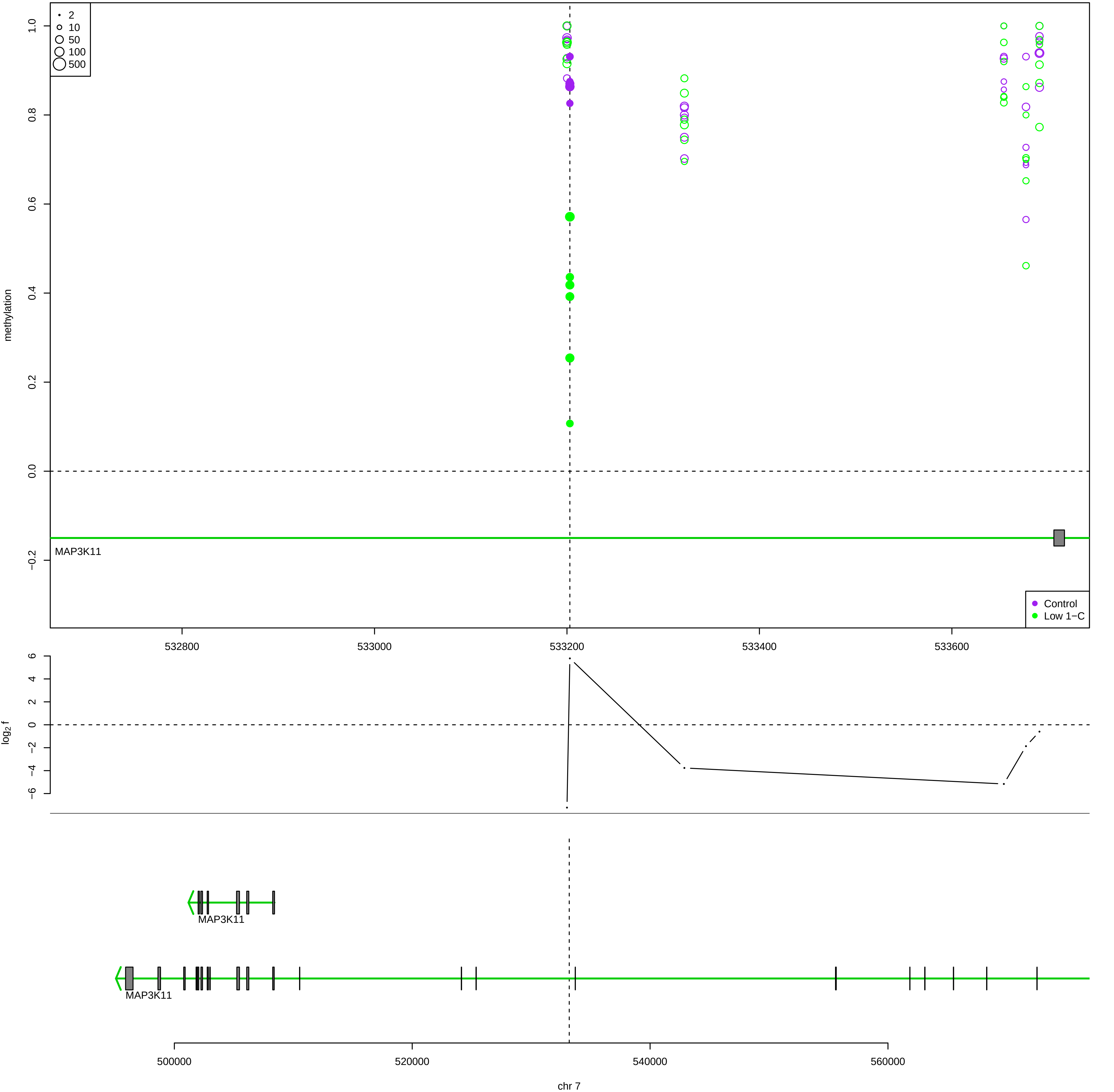

2
10
50
100
500
1.0
0.8
0.6
0.4
0.2
0.0
−0.2
methylation
MAP3K11
Control
Low 1−C
532800
533000
533200
533400
533600
6
4
2
0
−2
−4
−6
log_2 f
MAP3K11
MAP3K11
500000
520000
540000
560000
chr 7

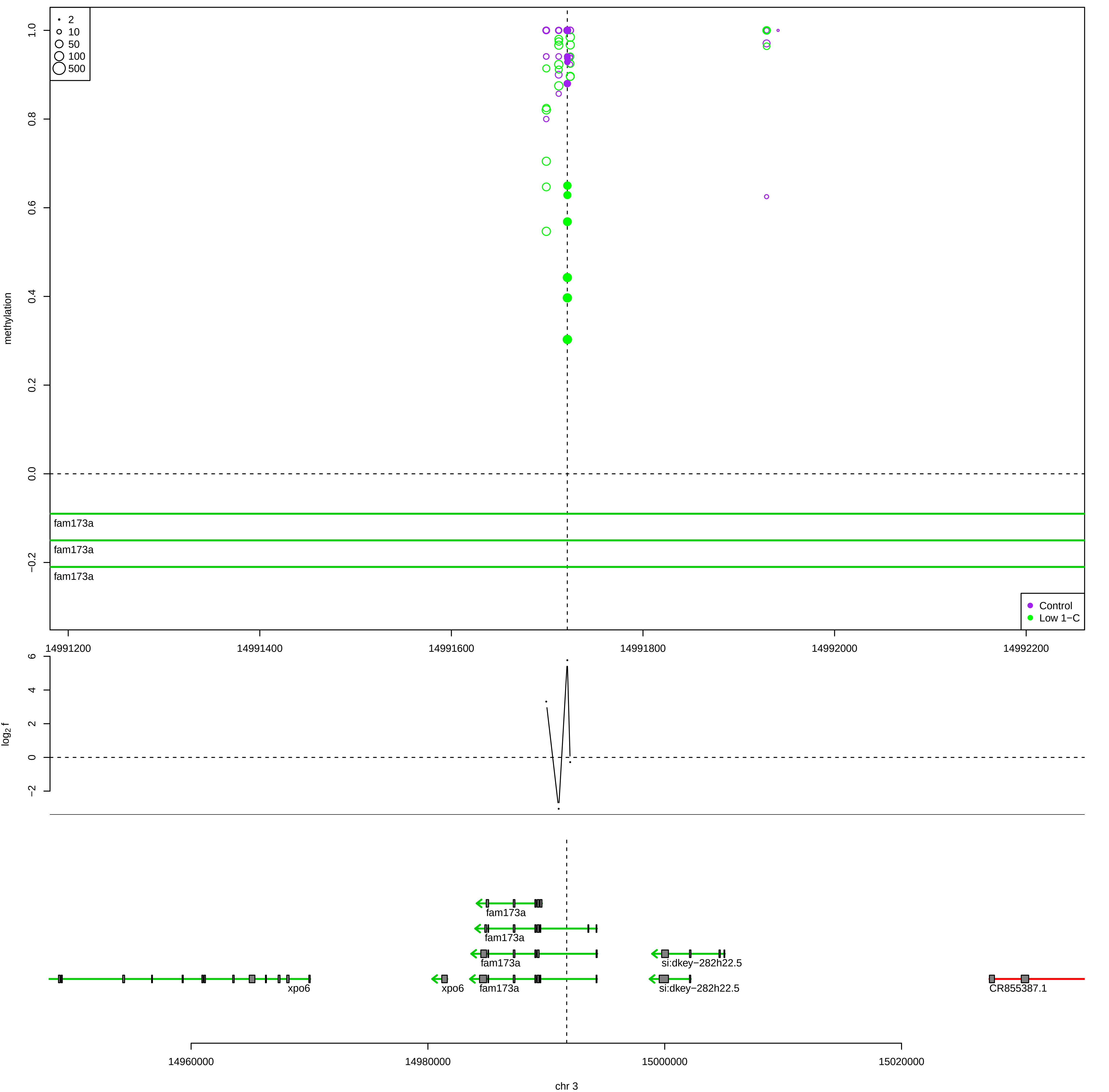

2
10
50
100
500
methylation
1.0
0.8
0.6
0.4
0.2
0.0
−0.2
fam173a
fam173a
fam173a
Control
Low 1−C
14991200
14991400
14991600
14991800
14992000
14992200
log2 f
6
4
2
0
−2
fam173a
fam173a
fam173a
si:dkey−282h22.5
xpo6
xpo6
fam173a
si:dkey−282h22.5
CR855387.1
14960000
14980000
15000000
15020000
chr 3

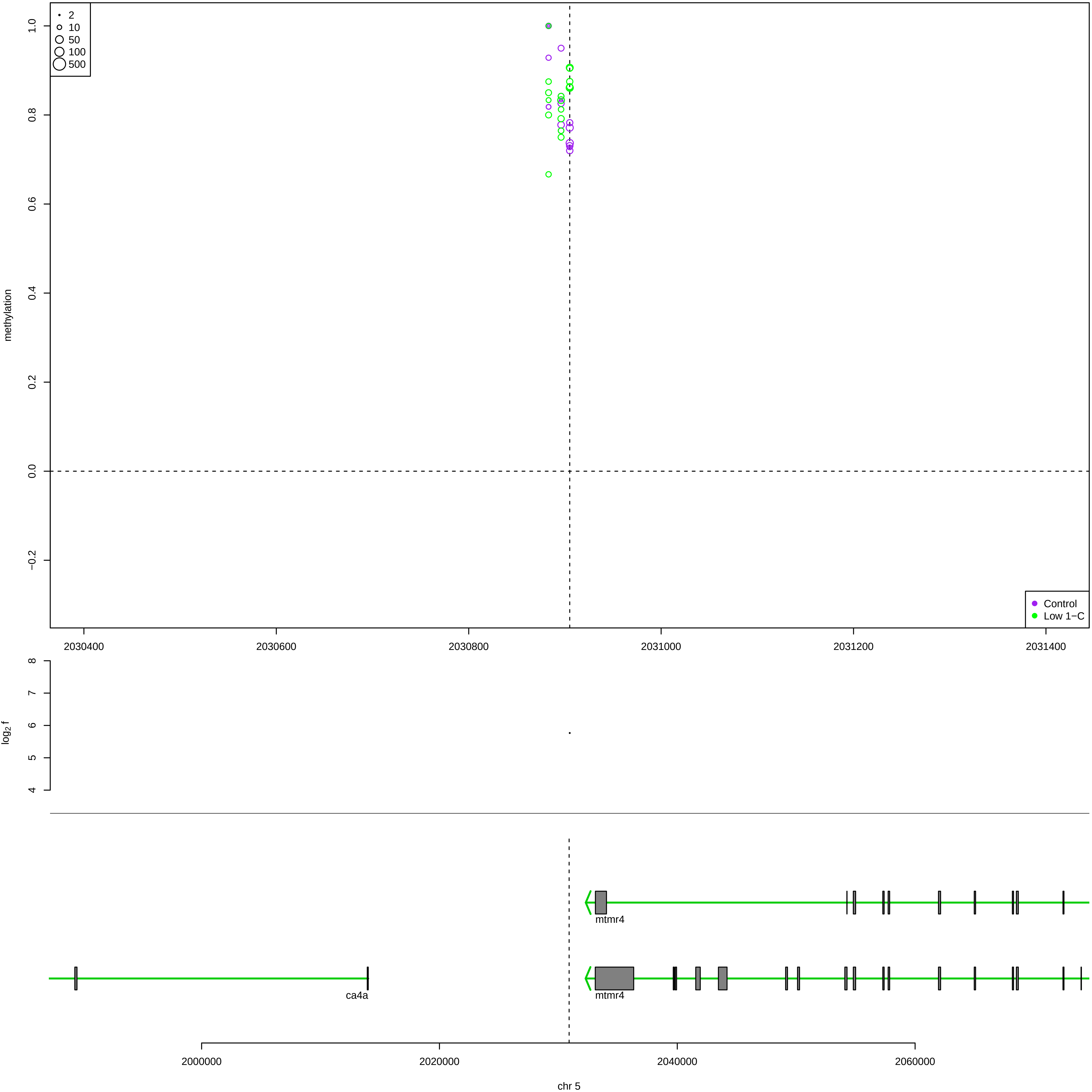

2
10
50
100
500
Control
Low 1−C
methylation
1.0
0.8
0.6
0.4
0.2
0.0
−0.2
2030400
2030600
2030800
2031000
2031200
2031400
log_2 f
8
7
6
5
4
mtmr4
mtmr4
ca4a
2000000
2020000
2040000
2060000
chr 5

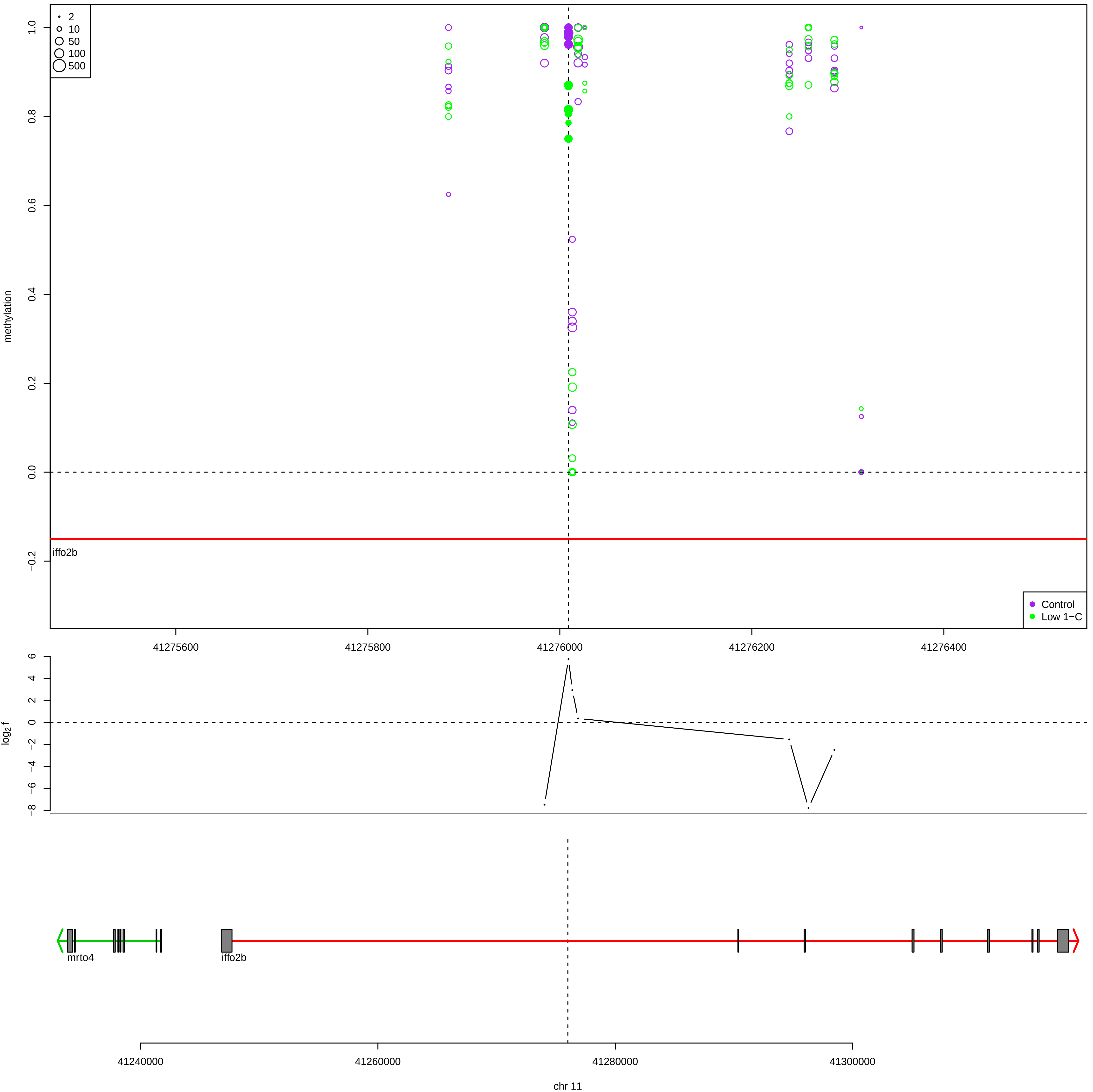

2
10
50
100
500
methylation
iffo2b
Control
Low 1−C
41275600
41275800
41276000
41276200
41276400
log2 f
mrto4
iffo2b
41240000
41260000
41280000
41300000
chr 11

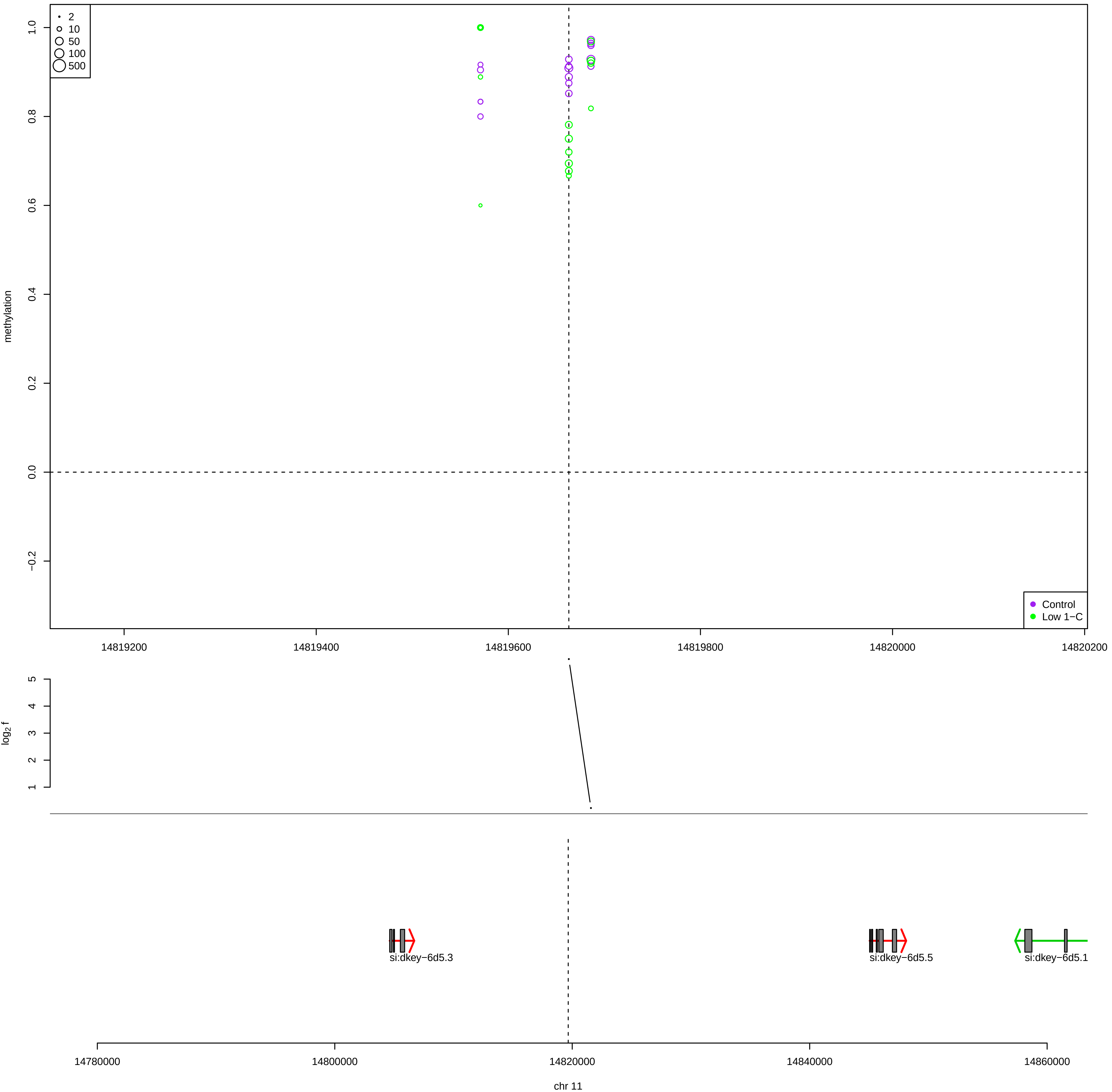

2
10
50
100
500
methylation
1.0
0.8
0.6
0.4
0.2
0.0
−0.2
Control
Low 1−C
14819200
14819400
14819600
14819800
14820000
14820200
log2 f
5
4
3
2
1
si:dkey−6d5.3
si:dkey−6d5.5
si:dkey−6d5.1
14780000
14800000
14820000
14840000
14860000
chr 11

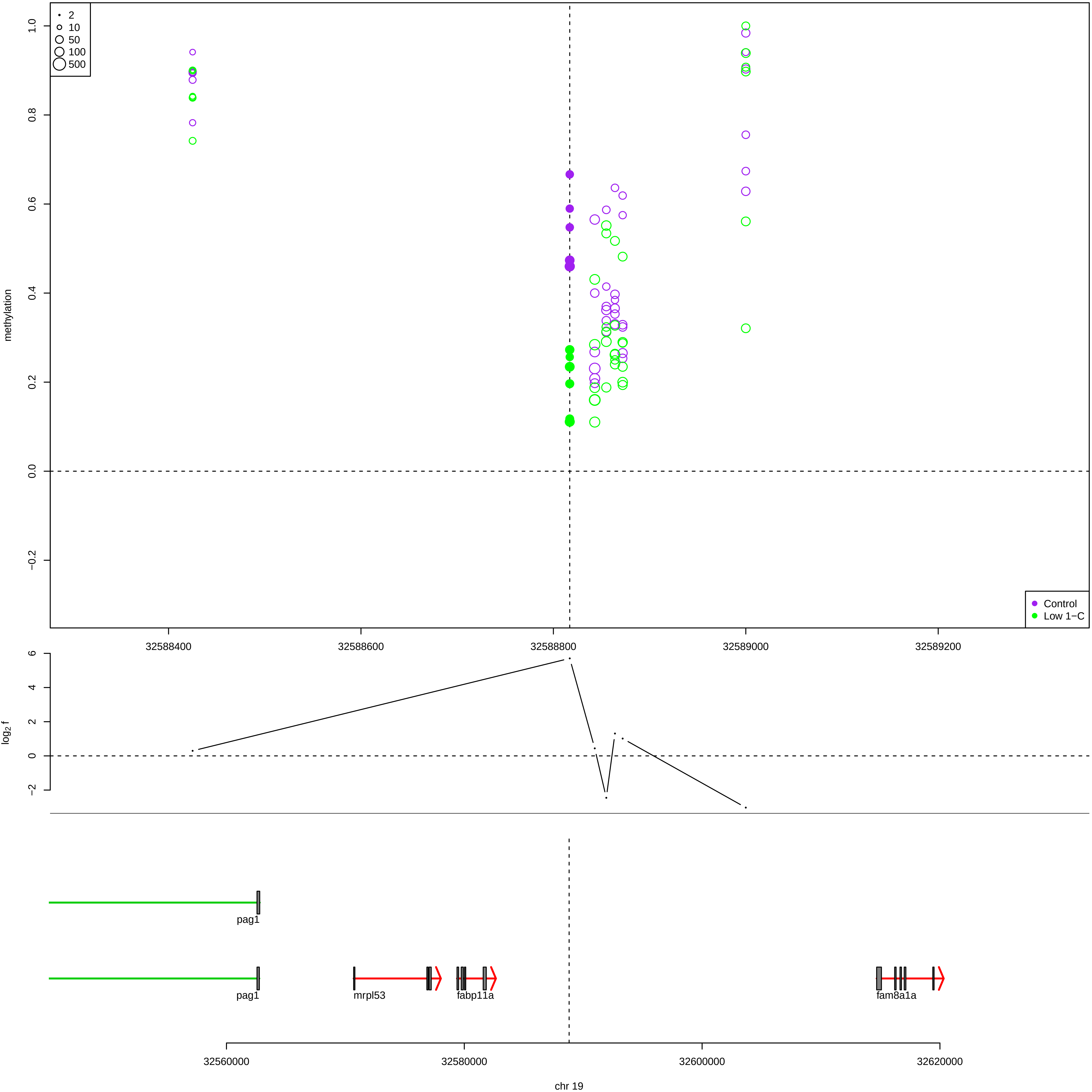

2
10
50
100
500
1.0
0.8
0.6
0.4
0.2
0.0
−0.2
methylation
Control
Low 1−C
32588400
32588600
32588800
32589000
32589200
6
4
2
0
−2
log2 f
pag1
pag1
mrpl53
fabp11a
fam8a1a
32560000
32580000
32600000
32620000
chr 19

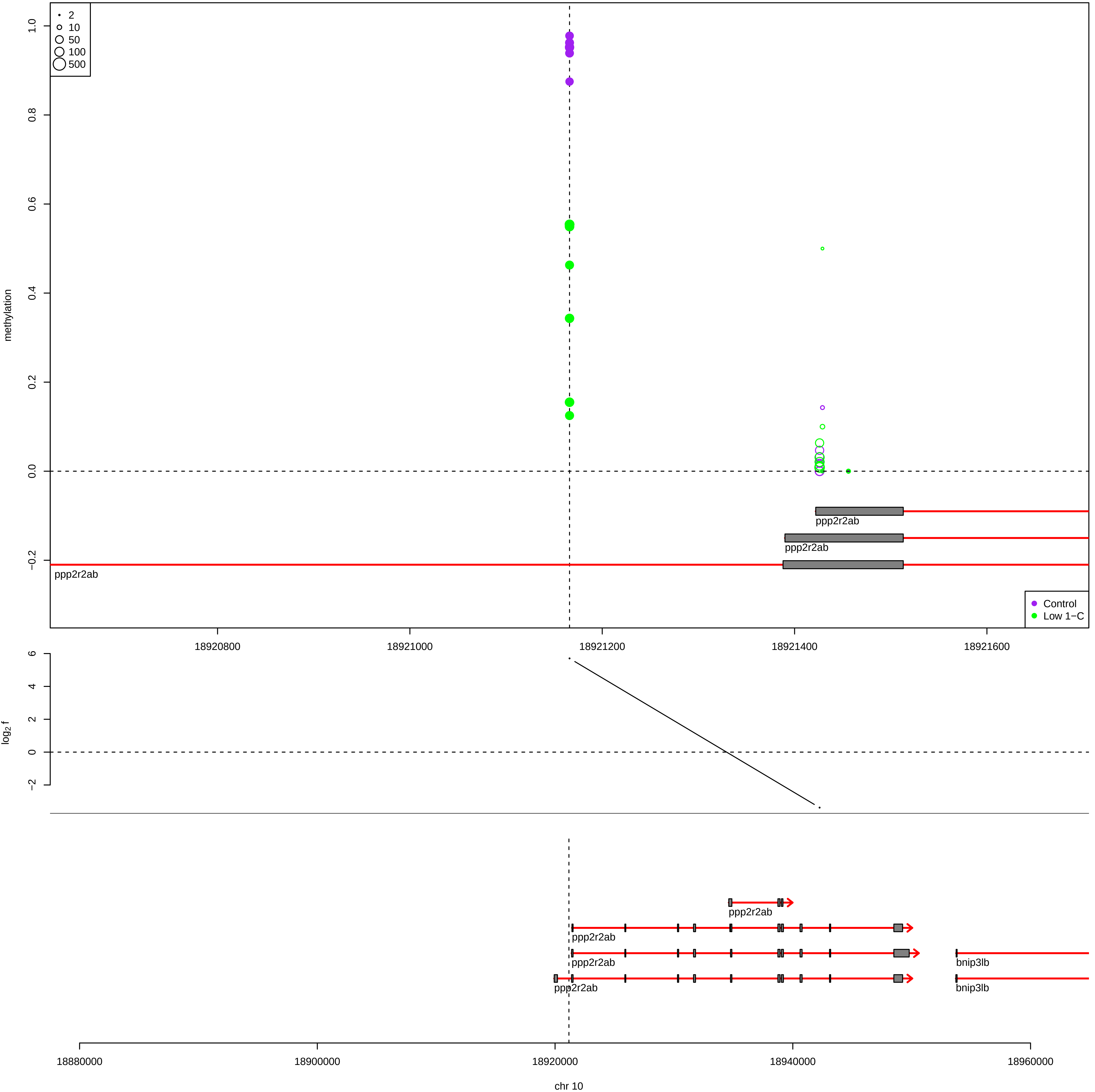

2
10
50
100
500
methylation
1.0
0.8
0.6
0.4
0.2
0.0
−0.2
ppp2r2ab
ppp2r2ab
ppp2r2ab
Control
Low 1−C
18920800
18921000
18921200
18921400
18921600
log2 f
6
4
2
0
−2
ppp2r2ab
ppp2r2ab
ppp2r2ab
ppp2r2ab
bnip3lb
bnip3lb
18880000
18900000
18920000
18940000
18960000
chr 10

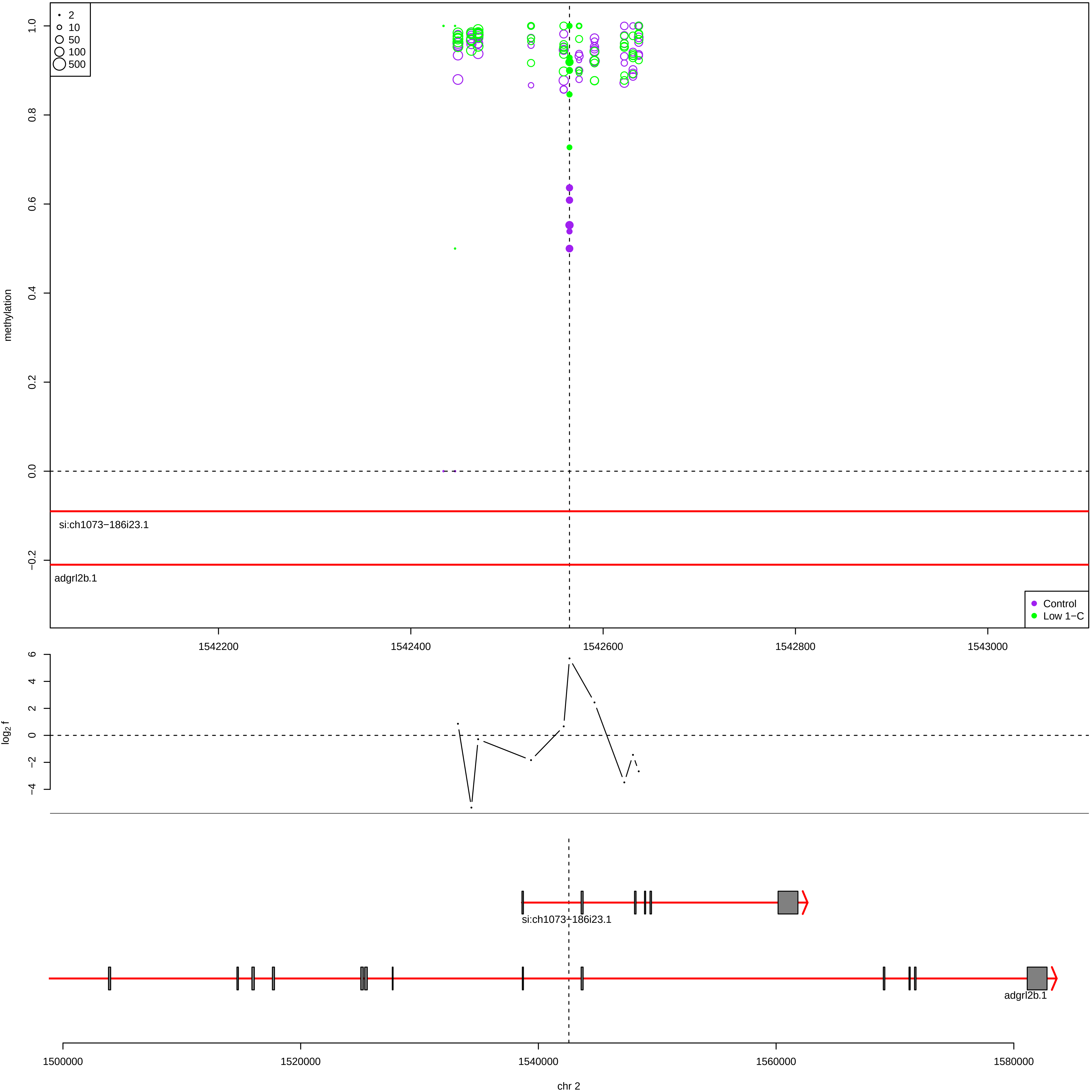

methylation
2
10
50
100
500
1.0
0.8
0.6
0.4
0.2
0.0
−0.2
si:ch1073−186i23.1
adgrl2b.1
Control
Low 1−C
1542200
1542400
1542600
1542800
1543000
log2 f
6
4
2
0
−2
−4
si:ch1073−186i23.1
adgrl2b.1
1500000
1520000
1540000
1560000
1580000
chr 2

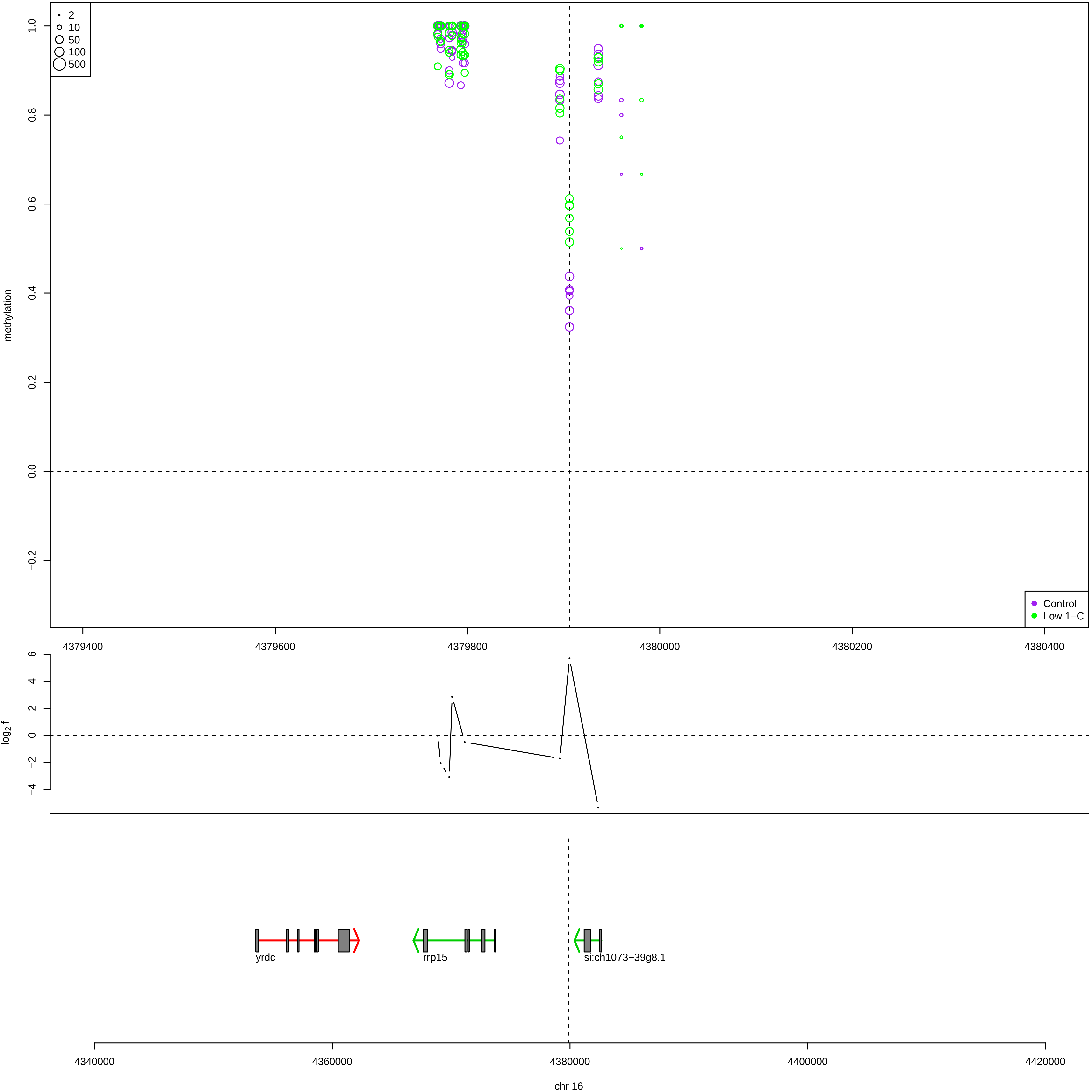

methylation
2
10
50
100
500
Control
Low 1−C
4379400
4379600
4379800
4380000
4380200
4380400
log2 f
yrdc
rrp15
si:ch1073−39g8.1
4340000
4360000
4380000
4400000
4420000
chr 16

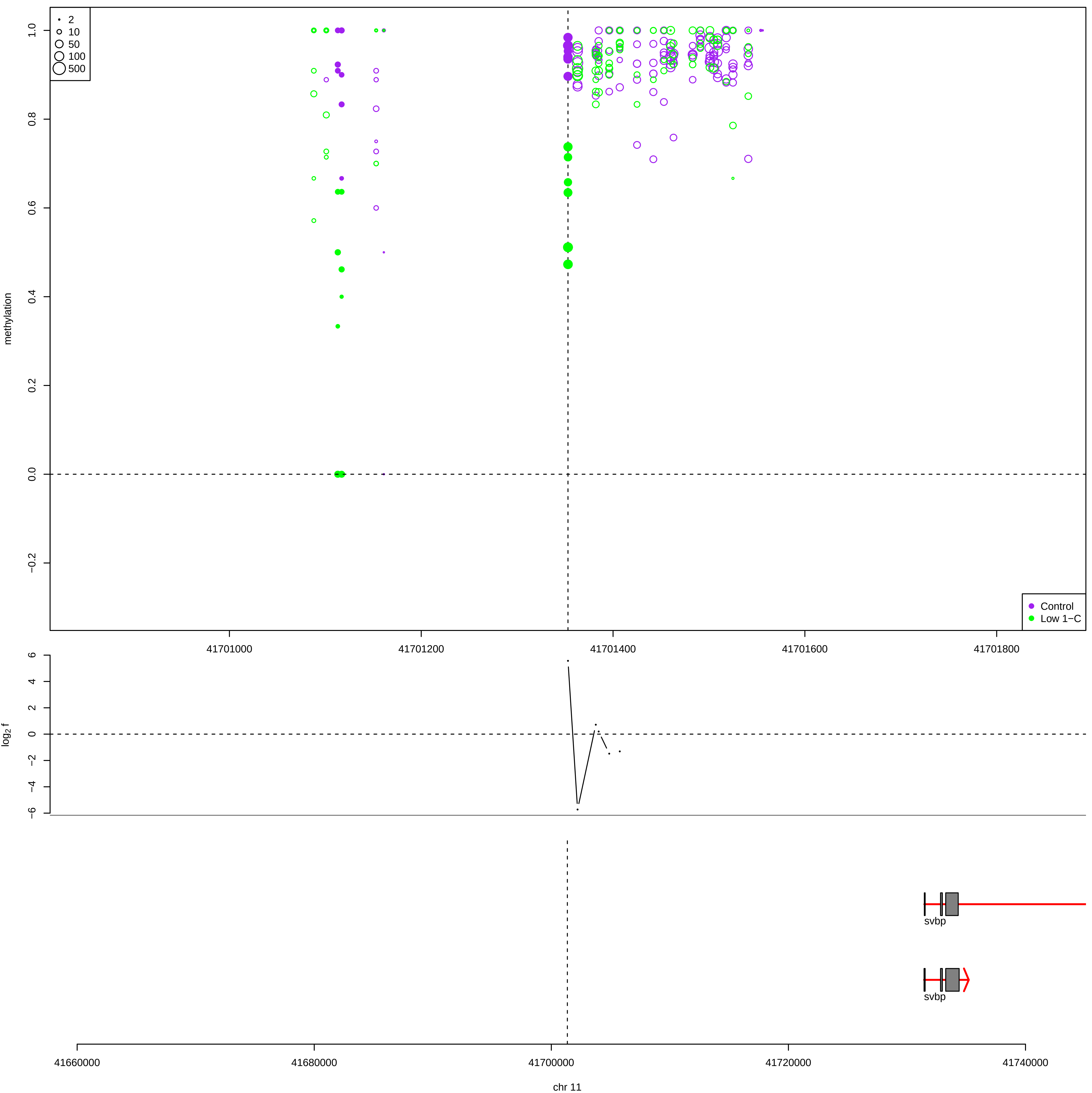

2
10
50
100
500
Control
Low 1−C
methylation
1.0
0.8
0.6
0.4
0.2
0.0
−0.2
41701000
41701200
41701400
41701600
41701800
log2 f
6
4
2
0
−2
−4
−6
svbp
svbp
41660000
41680000
41700000
41720000
41740000
chr 11

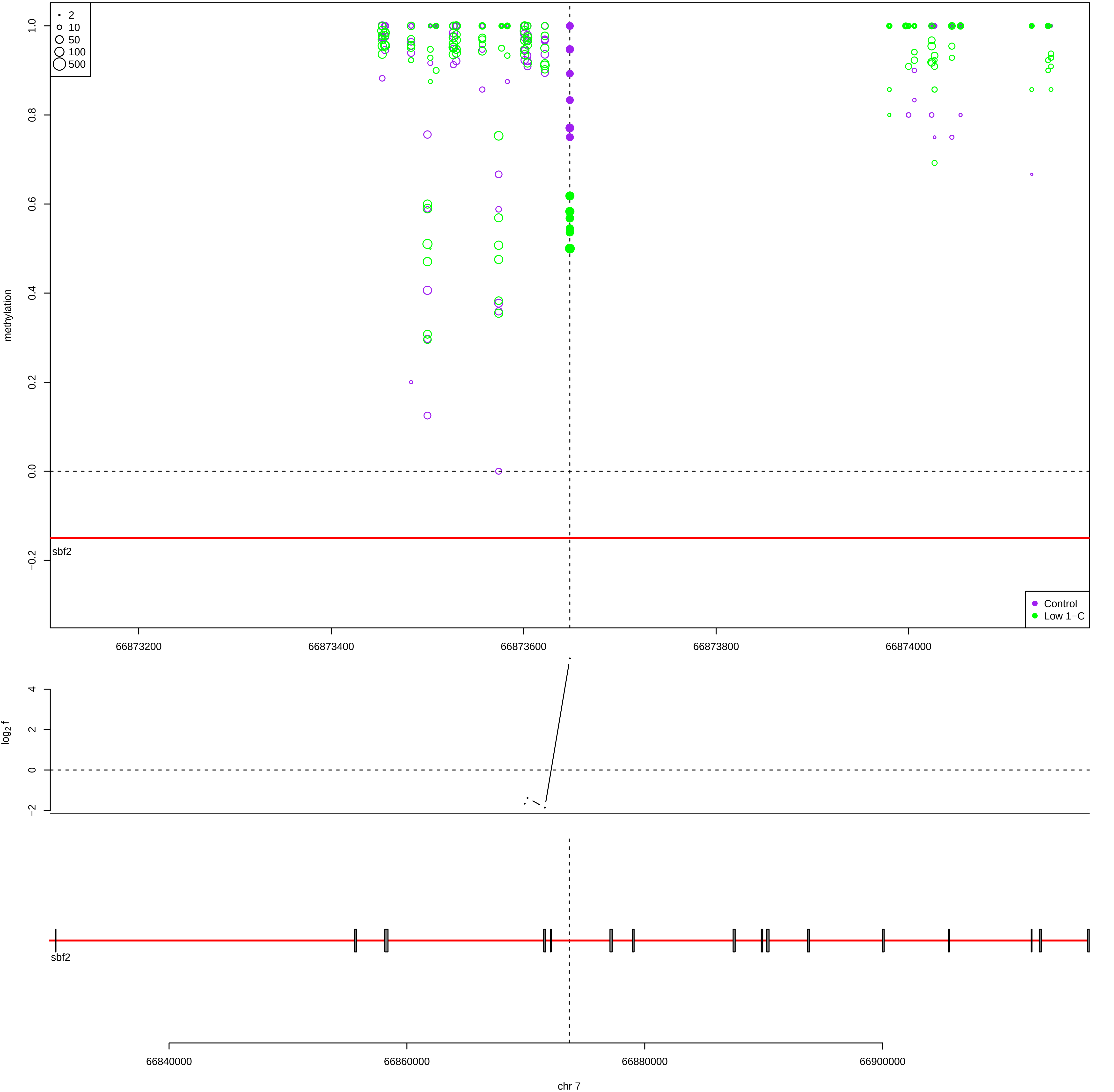

2
10
50
100
500
methylation
1.0
0.8
0.6
0.4
0.2
0.0
−0.2
sbf2
Control
Low 1−C
66873200
66873400
66873600
66873800
66874000
log2 f
4
2
0
−2
sbf2
66840000
66860000
66880000
66900000
chr 7

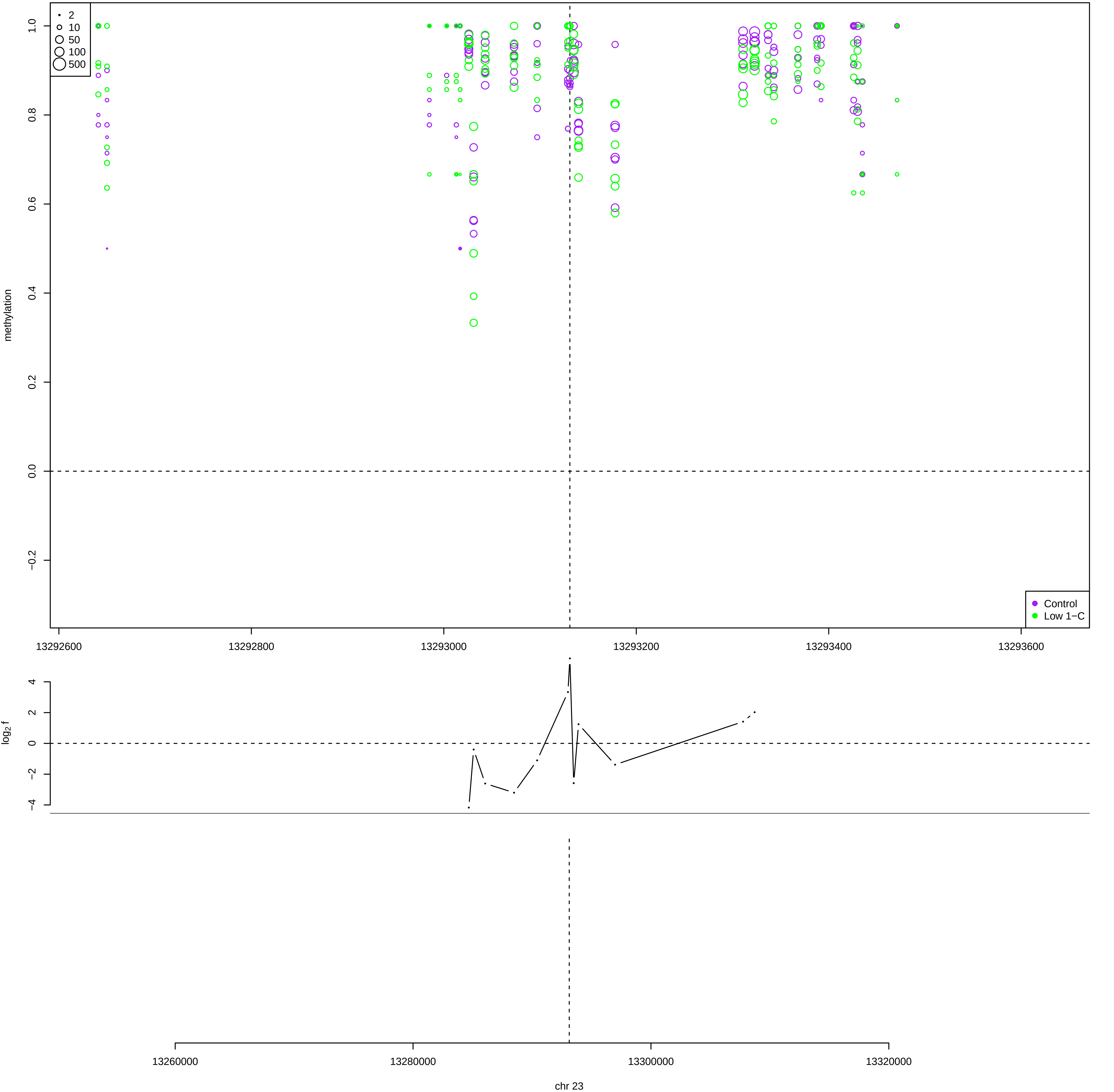

2
10
50
100
500
methylation
1.0
0.8
0.6
0.4
0.2
0.0
−0.2
Control
Low 1−C
13292600
13292800
13293000
13293200
13293400
13293600
log2 f
4
2
0
-2
-4
13260000
13280000
13300000
13320000
chr 23

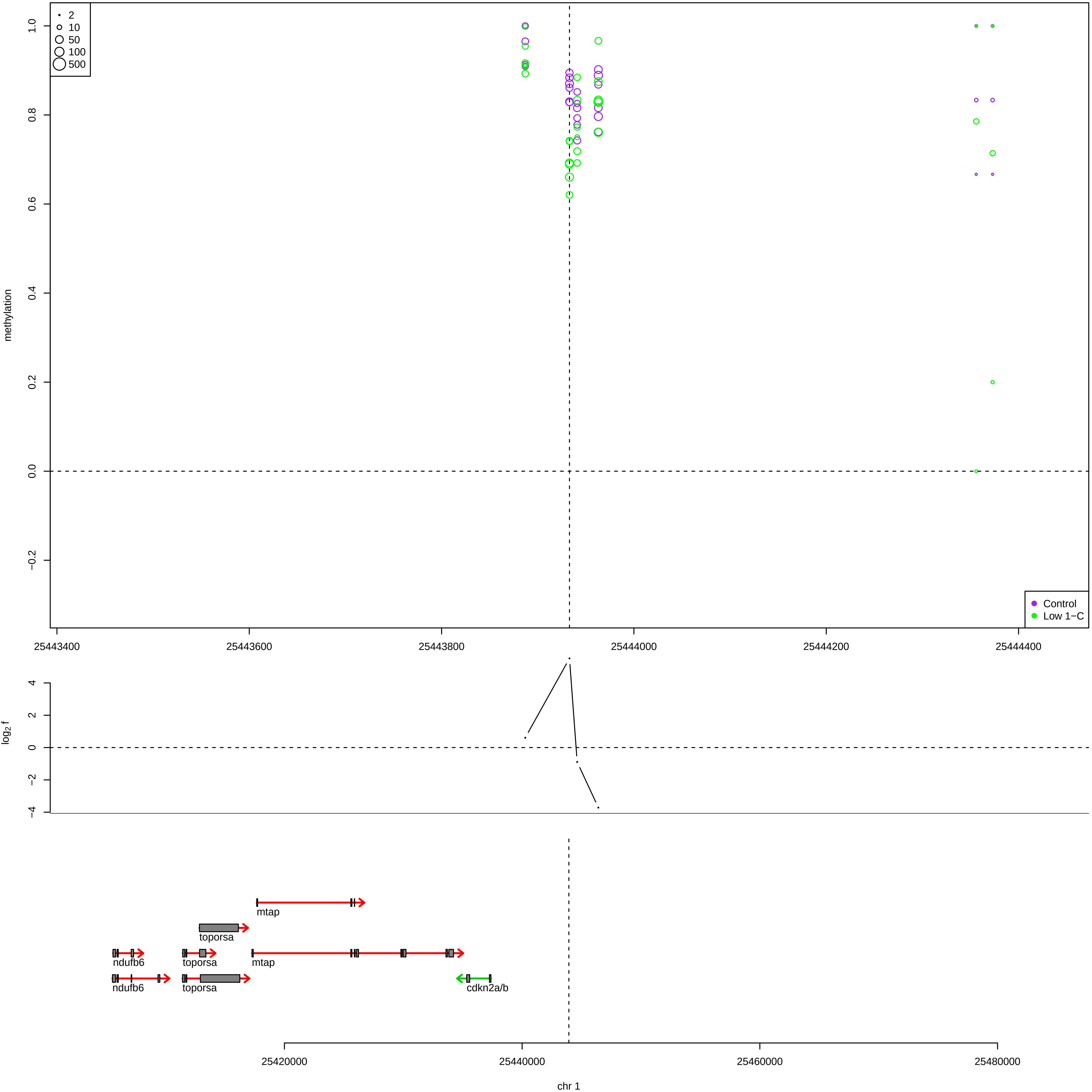

2
10
50
100
500
methylation
1.0
0.8
0.6
0.4
0.2
0.0
−0.2
Control
Low 1−C
25443400
25443600
25443800
25444000
25444200
25444400
log2 f
4
2
0
−2
−4
mtap
toporsa
ndufb6
toporsa
mtap
ndufb6
toporsa
cdkn2a/b
25420000
25440000
25460000
25480000
chr 1

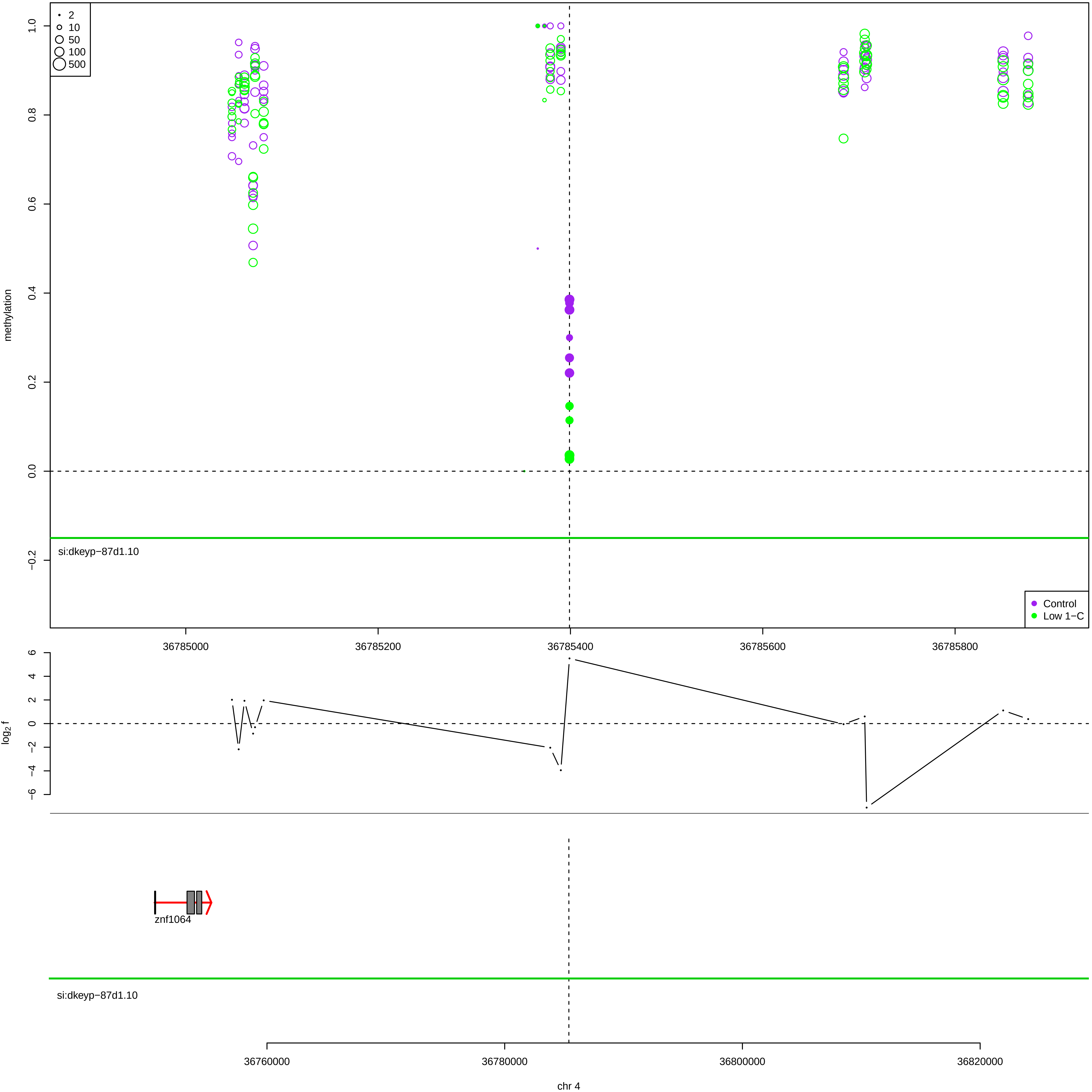

2
10
50
100
500
methylation
1.0
0.8
0.6
0.4
0.2
0.0
-0.2
si:dkeyp-87d1.10
Control
Low 1-C
36785000
36785200
36785400
36785600
36785800
$log_2 f$
6
4
2
0
-2
-4
-6
znf1064
si:dkeyp-87d1.10
36760000
36780000
36800000
36820000
chr 4

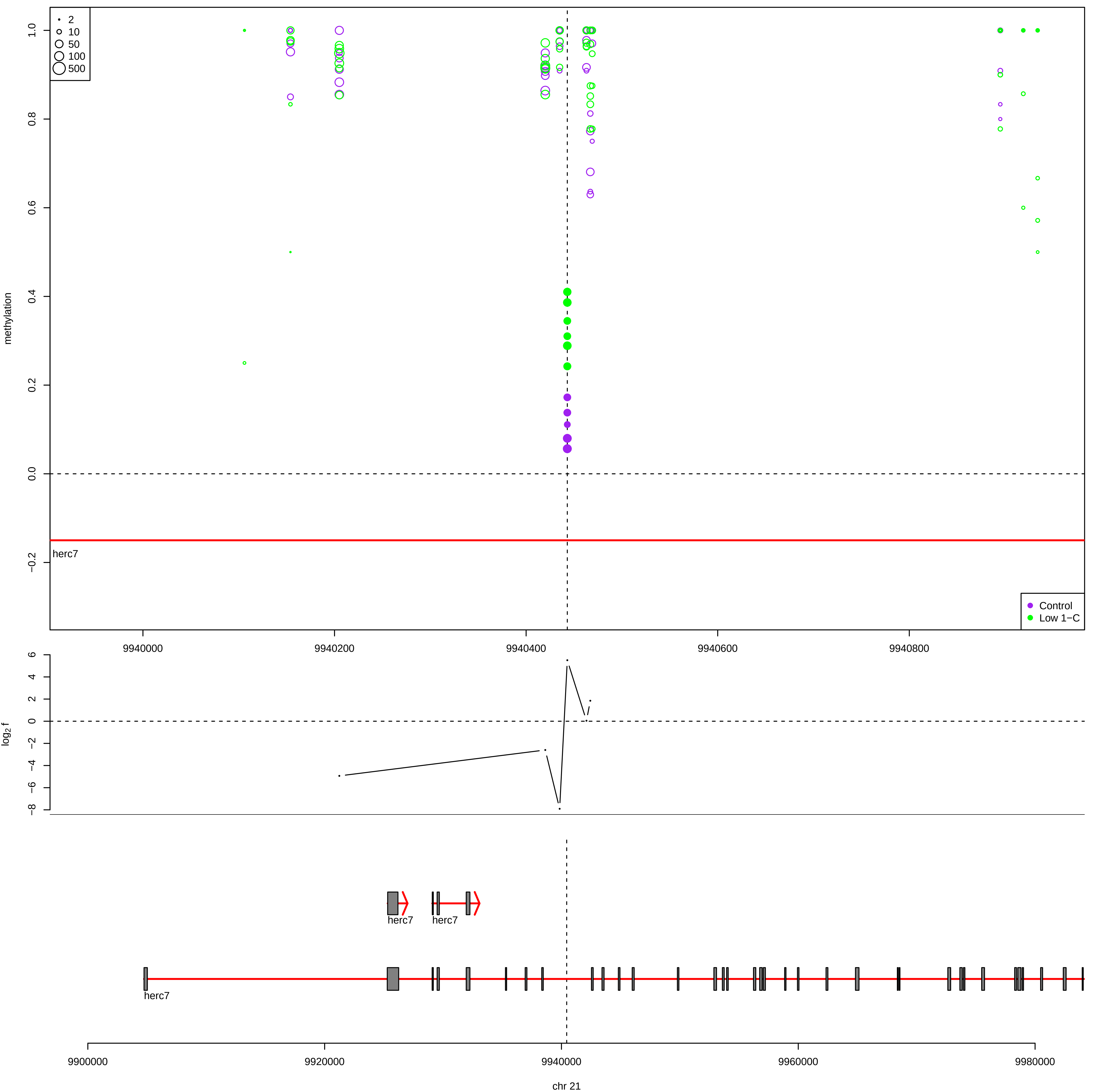

2
10
50
100
500
methylation
1.0
0.8
0.6
0.4
0.2
0.0
−0.2
herc7
Control
Low 1−C
9940000
9940200
9940400
9940600
9940800
6
4
2
0
−2
−4
−6
−8
log2 f
herc7
herc7
herc7
9900000
9920000
9940000
9960000
9980000
chr 21

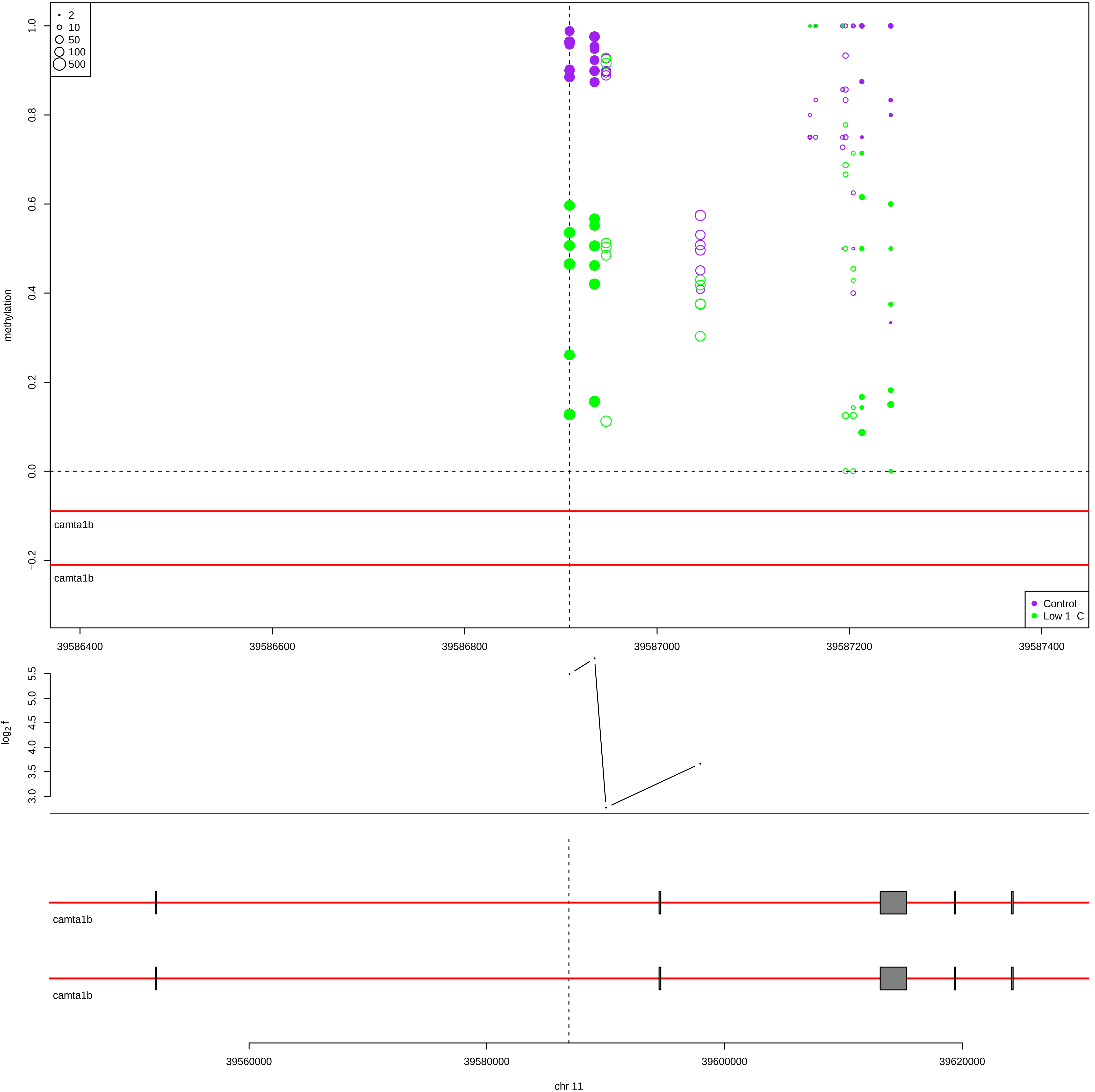

2
10
50
100
500
methylation
1.0
0.8
0.6
0.4
0.2
0.0
−0.2
camta1b
camta1b
Control
Low 1−C
39586400
39586600
39586800
39587000
39587200
39587400
log2 f
5.5
5.0
4.5
4.0
3.5
3.0
camta1b
camta1b
39560000
39580000
39600000
39620000
chr 11

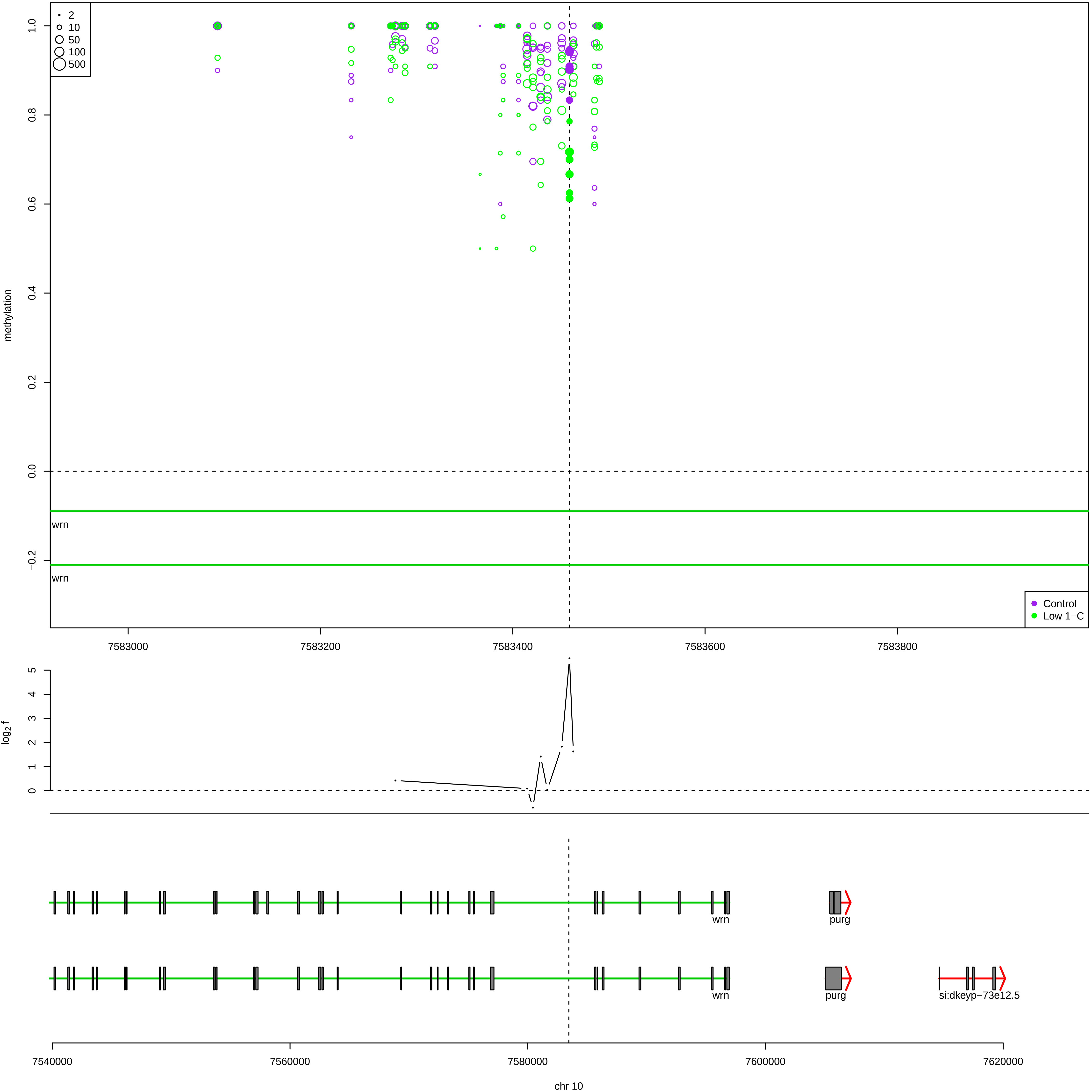

2
10
50
100
500
methylation
1.0
0.8
0.6
0.4
0.2
0.0
−0.2
wrn
wrn
Control
Low 1−C
7583000
7583200
7583400
7583600
7583800
log2 f
5
4
3
2
1
0
wrn
purg
wrn
purg
si:dkeyp−73e12.5
7540000
7560000
7580000
7600000
7620000
chr 10

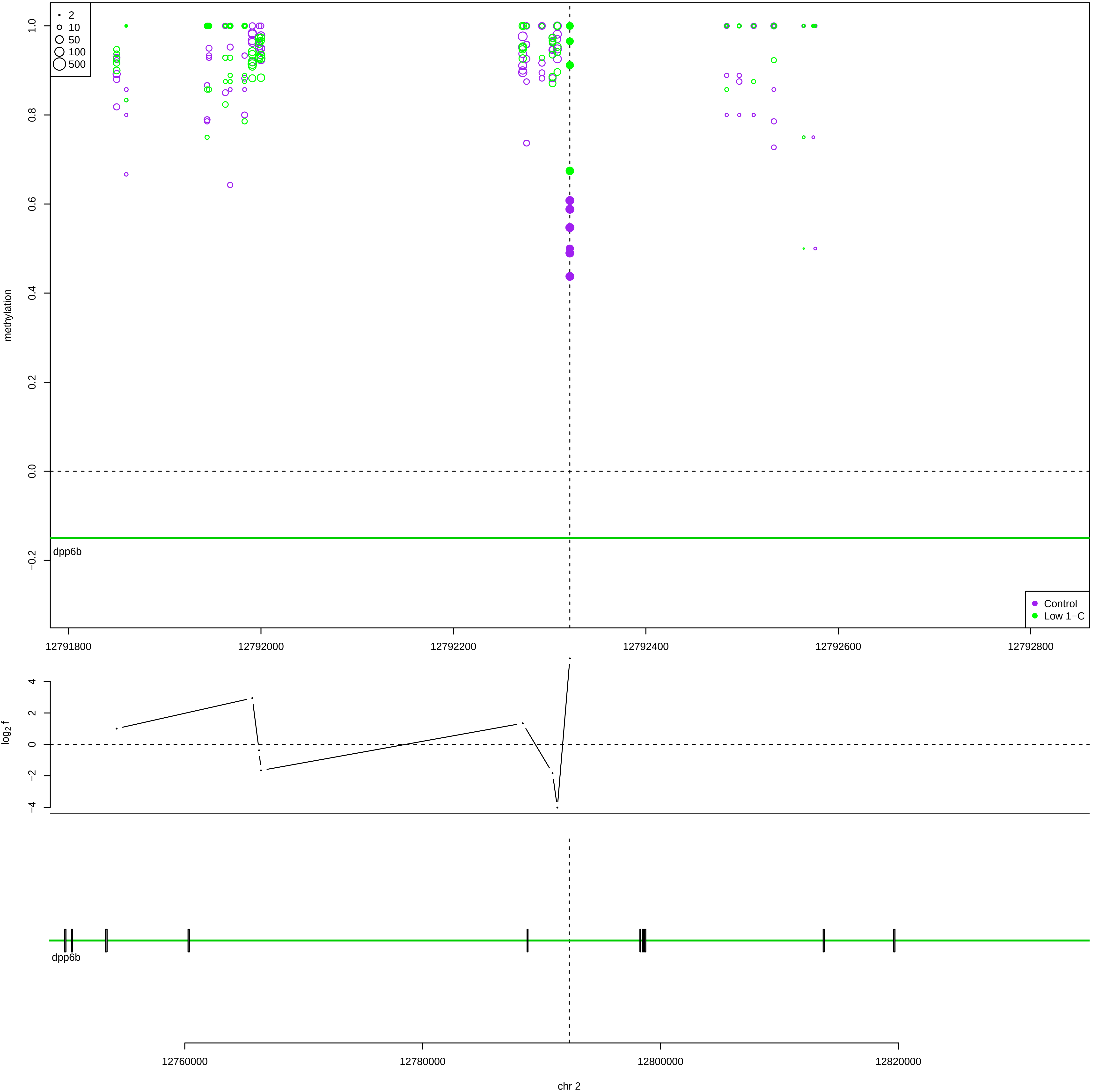

2
10
50
100
500
methylation
1.0
0.8
0.6
0.4
0.2
0.0
−0.2
dpp6b
Control
Low 1−C
12791800
12792000
12792200
12792400
12792600
12792800
4
2
0
−2
−4
$\log_2 f$
dpp6b
12760000
12780000
12800000
12820000
chr 2

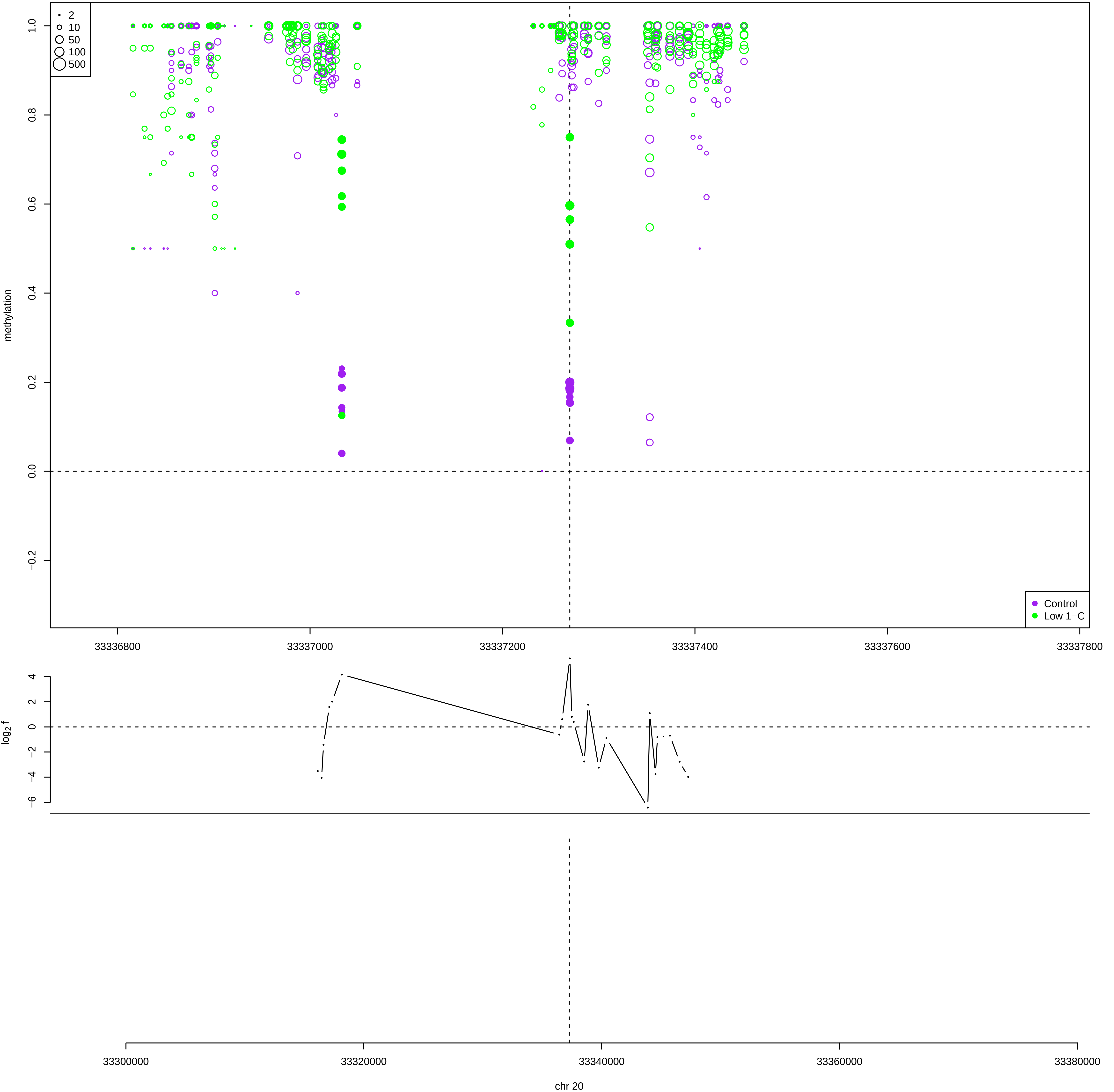

2
10
50
100
500
1.0
0.8
0.6
0.4
0.2
0.0
−0.2
methylation
Control
Low 1−C
33336800
33337000
33337200
33337400
33337600
33337800
4
2
0
−2
−4
−6
$\log_2 f$
33300000
33320000
33340000
33360000
33380000
chr 20

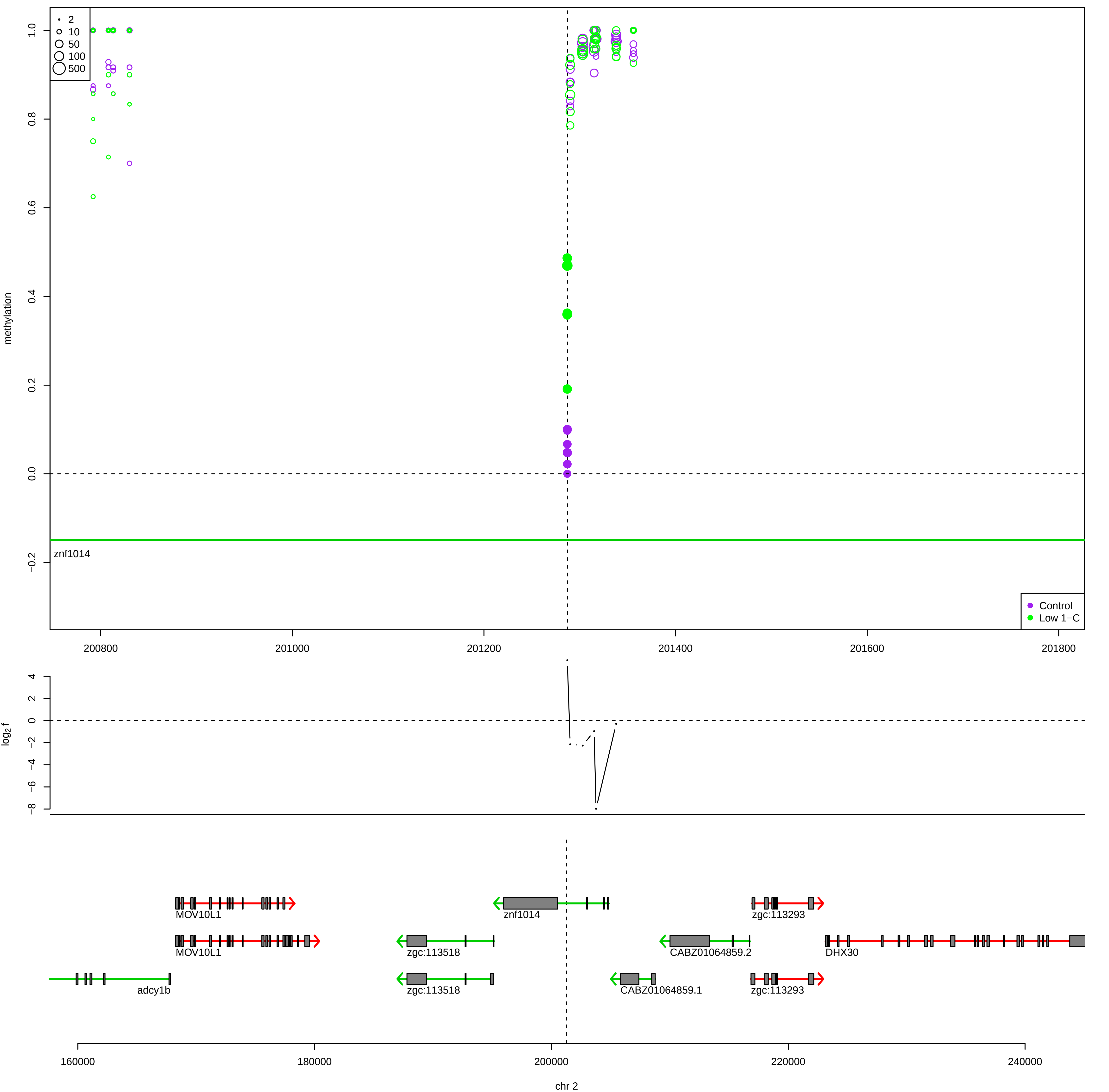

2
10
50
100
500
methylation
1.0
0.8
0.6
0.4
0.2
0.0
−0.2
znf1014
Control
Low 1−C
200800
201000
201200
201400
201600
201800
log2 f
4
2
0
−2
−4
−6
−8
MOV10L1
MOV10L1
adcy1b
zgc:113518
zgc:113518
znf1014
CABZ01064859.2
CABZ01064859.1
zgc:113293
zgc:113293
DHX30
160000
180000
200000
220000
240000
chr 2

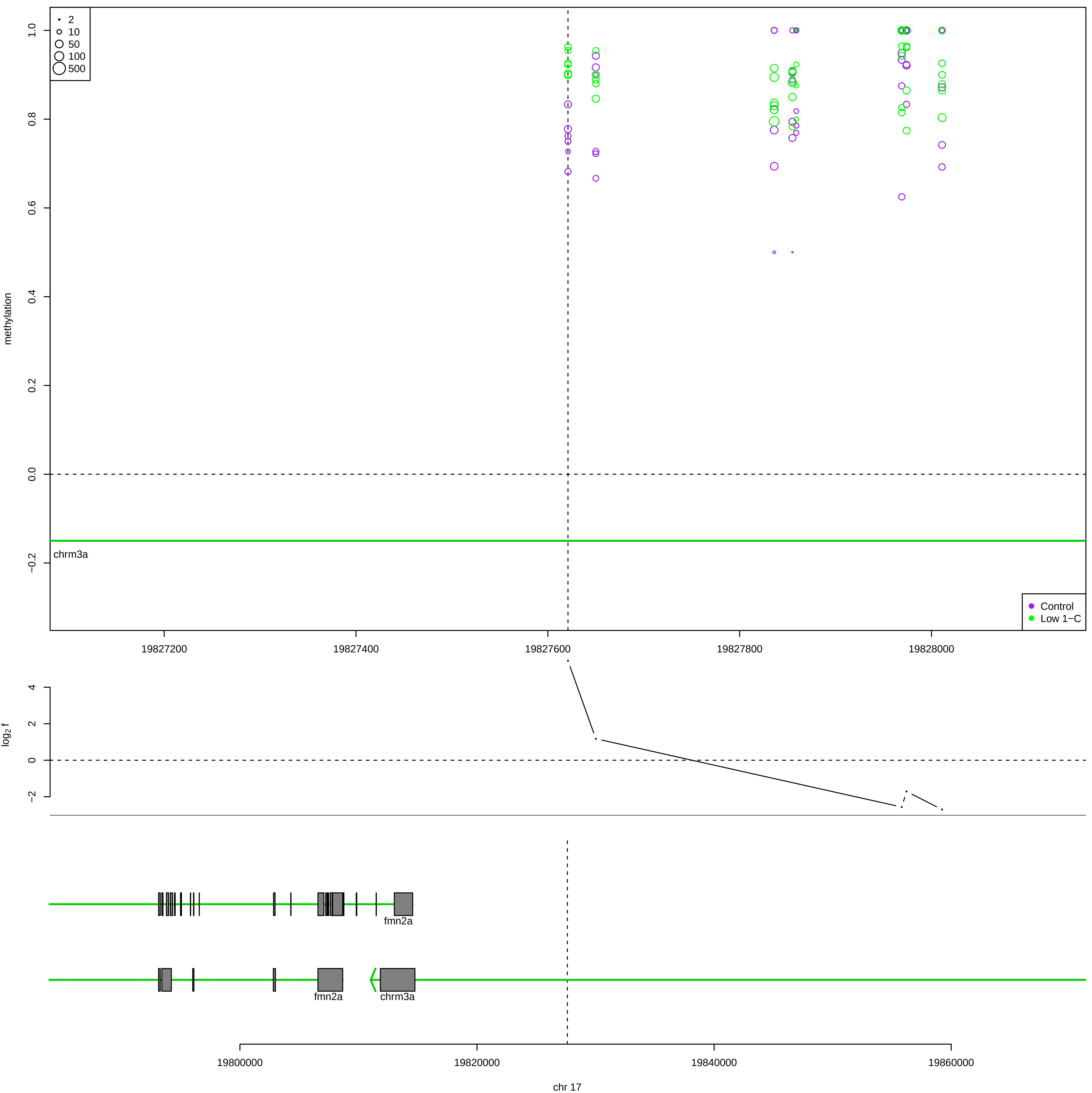

2
10
50
100
500
methylation
1.0
0.8
0.6
0.4
0.2
0.0
−0.2
chrm3a
Control
Low 1−C
19827200
19827400
19827600
19827800
19828000
log_2 f
4
2
0
−2
fmn2a
fmn2a
chrm3a
19800000
19820000
19840000
19860000
chr 17

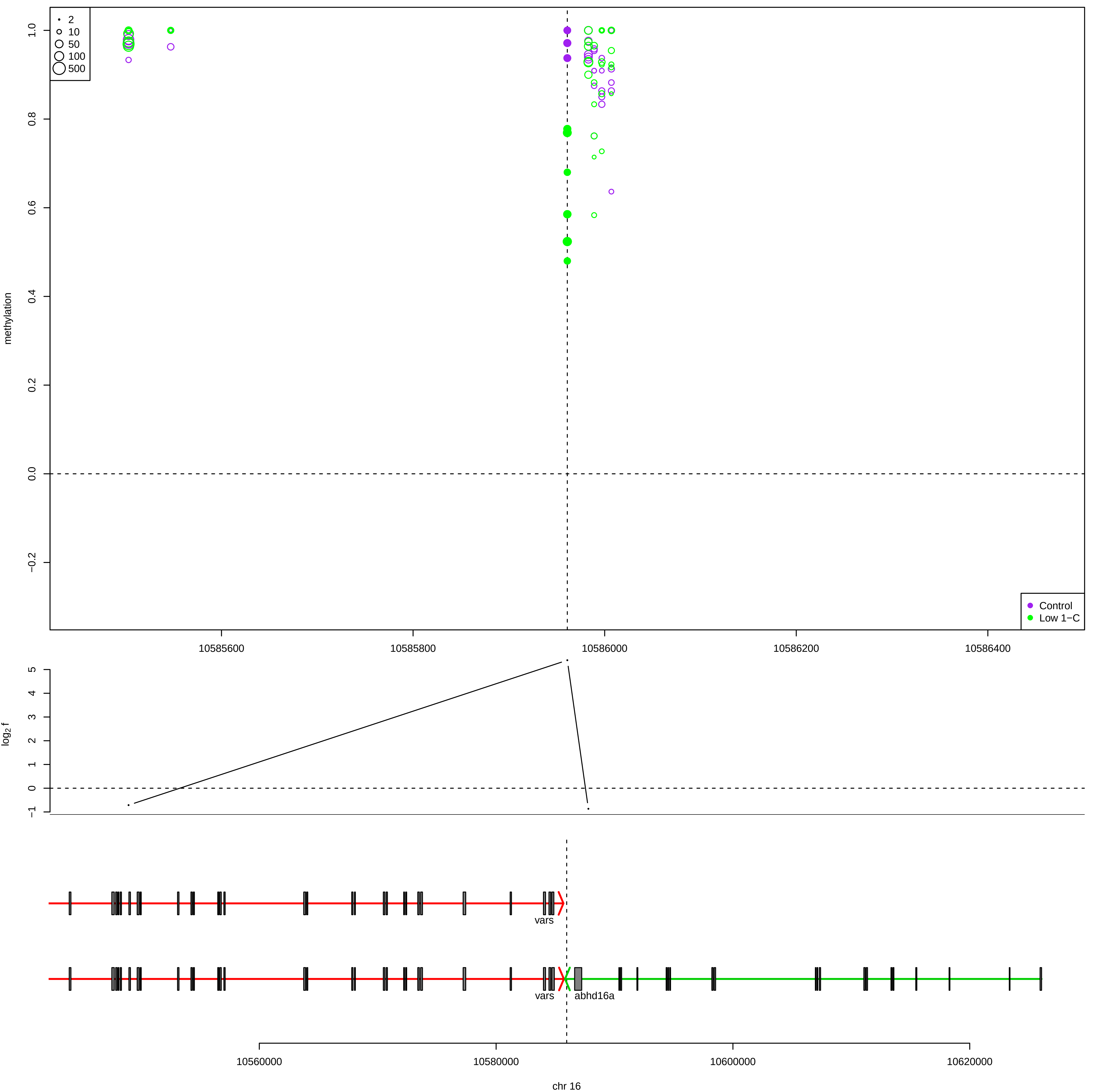

methylation
1.0
0.8
0.6
0.4
0.2
0.0
−0.2
2
10
50
100
500
Control
Low 1−C
10585600
10585800
10586000
10586200
10586400
$\log_2 f$
5
4
3
2
1
0
−1
vars
vars
abhd16a
10560000
10580000
10600000
10620000
chr 16

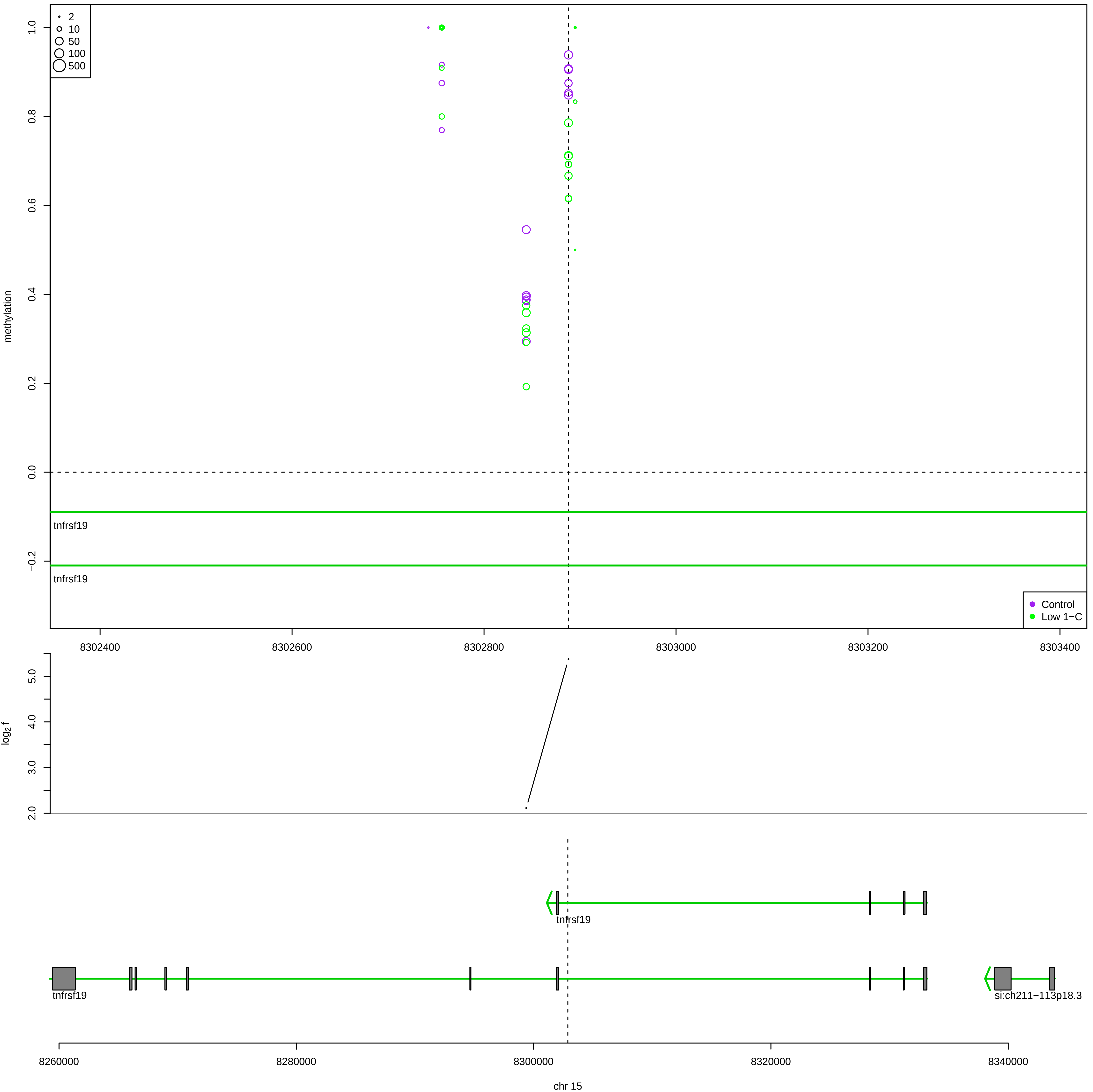

2
10
50
100
500
methylation
1.0
0.8
0.6
0.4
0.2
0.0
−0.2
tnfrsf19
tnfrsf19
Control
Low 1−C
8302400
8302600
8302800
8303000
8303200
8303400
$\log_2 f$
5.0
4.0
3.0
2.0
tnfrsf19
tnfrsf19
si:ch211−113p18.3
8260000
8280000
8300000
8320000
8340000
chr 15

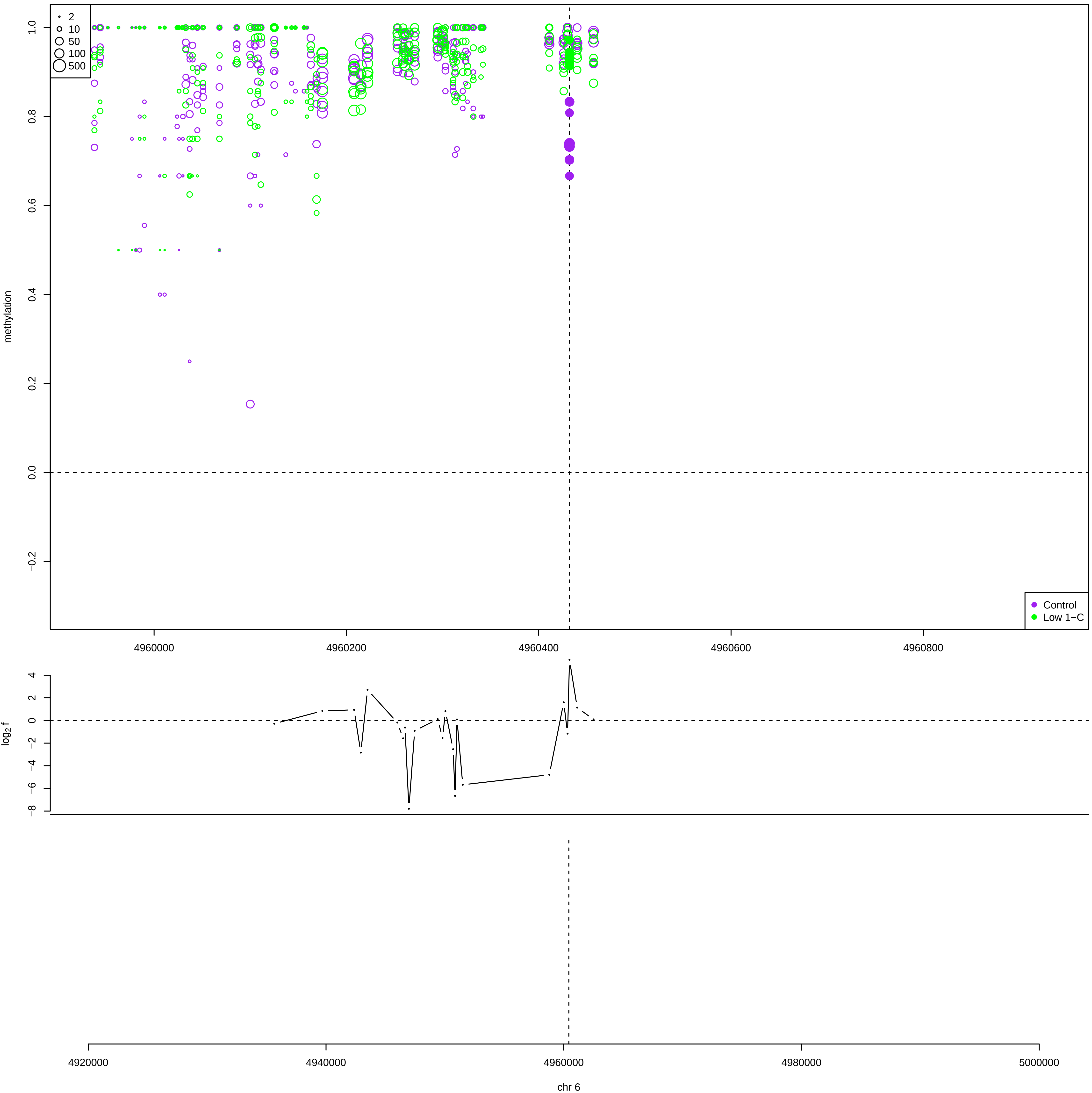

methylation
1.0
0.8
0.6
0.4
0.2
0.0
−0.2
2
10
50
100
500
Control
Low 1−C
4960000
4960200
4960400
4960600
4960800
log2 f
4
2
0
−2
−4
−6
−8
4920000
4940000
4960000
4980000
5000000
chr 6

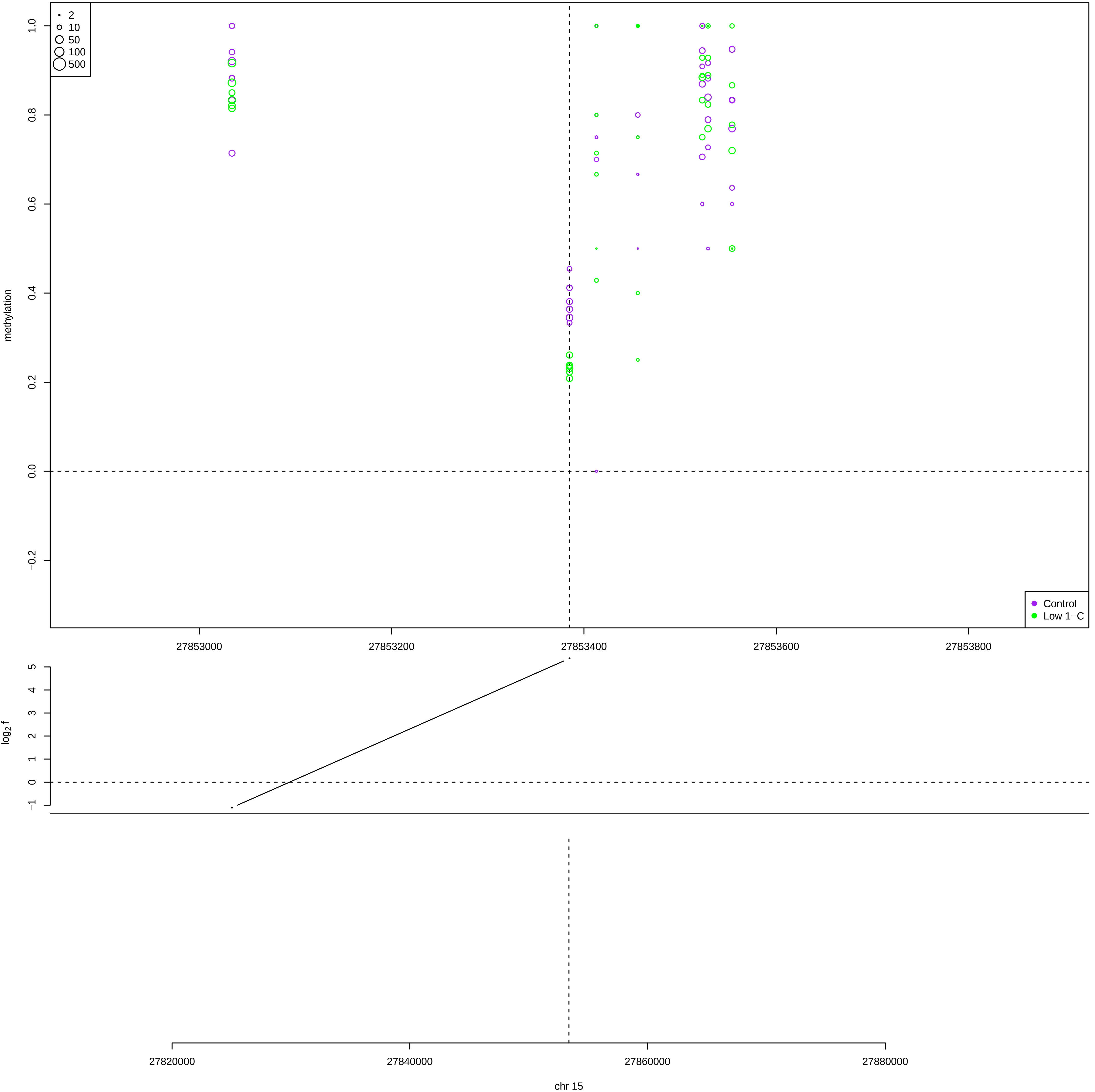

2
10
50
100
500
Control
Low 1−C
methylation
1.0
0.8
0.6
0.4
0.2
0.0
−0.2
27853000
27853200
27853400
27853600
27853800
log2 f
5
4
3
2
1
0
−1
27820000
27840000
27860000
27880000
chr 15

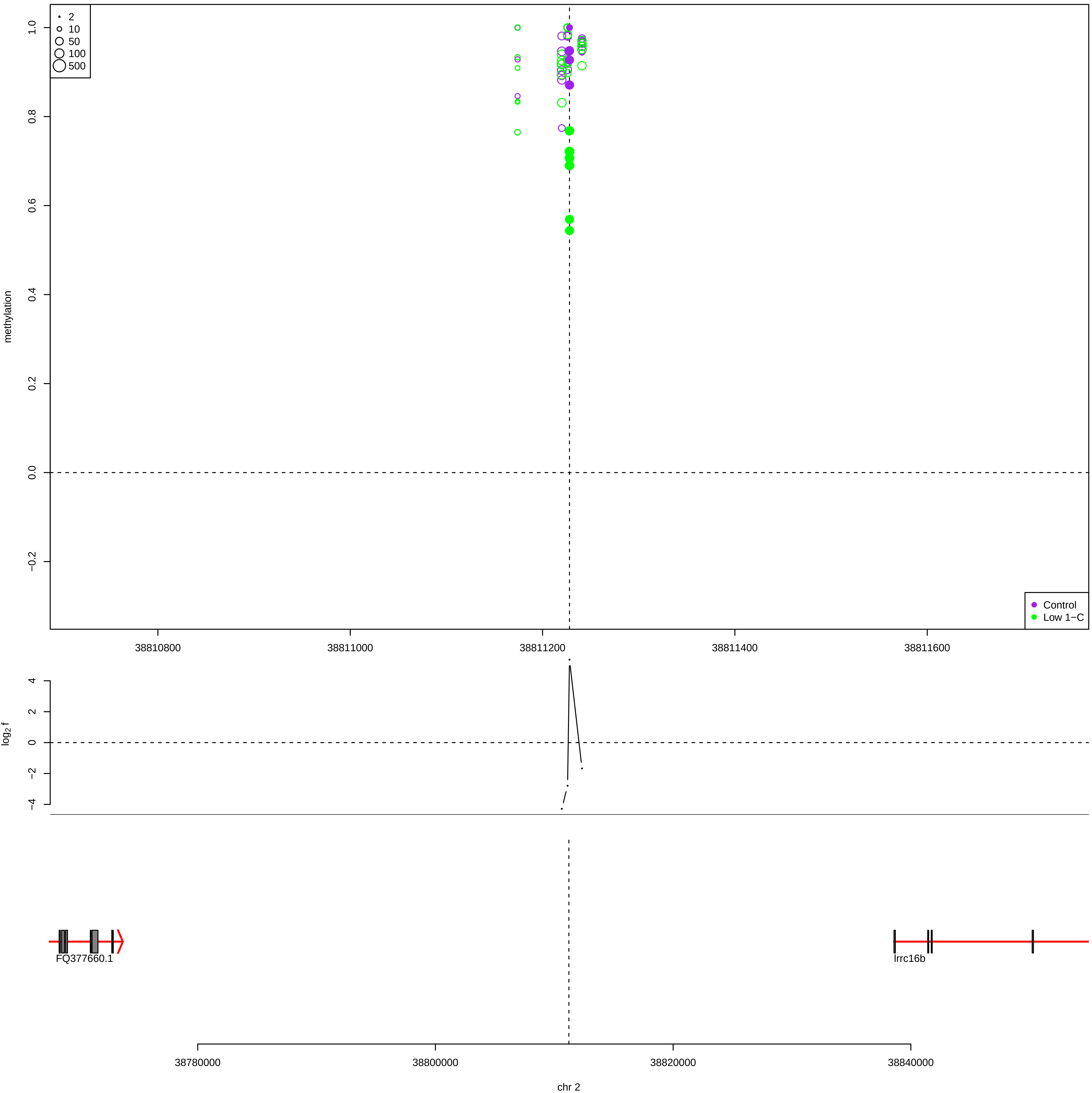

2
10
50
100
500
methylation
1.0
0.8
0.6
0.4
0.2
0.0
−0.2
Control
Low 1−C
38810800
38811000
38811200
38811400
38811600
log2 f
4
2
0
−2
−4
FQ377660.1
lrrc16b
38780000
38800000
38820000
38840000
chr 2

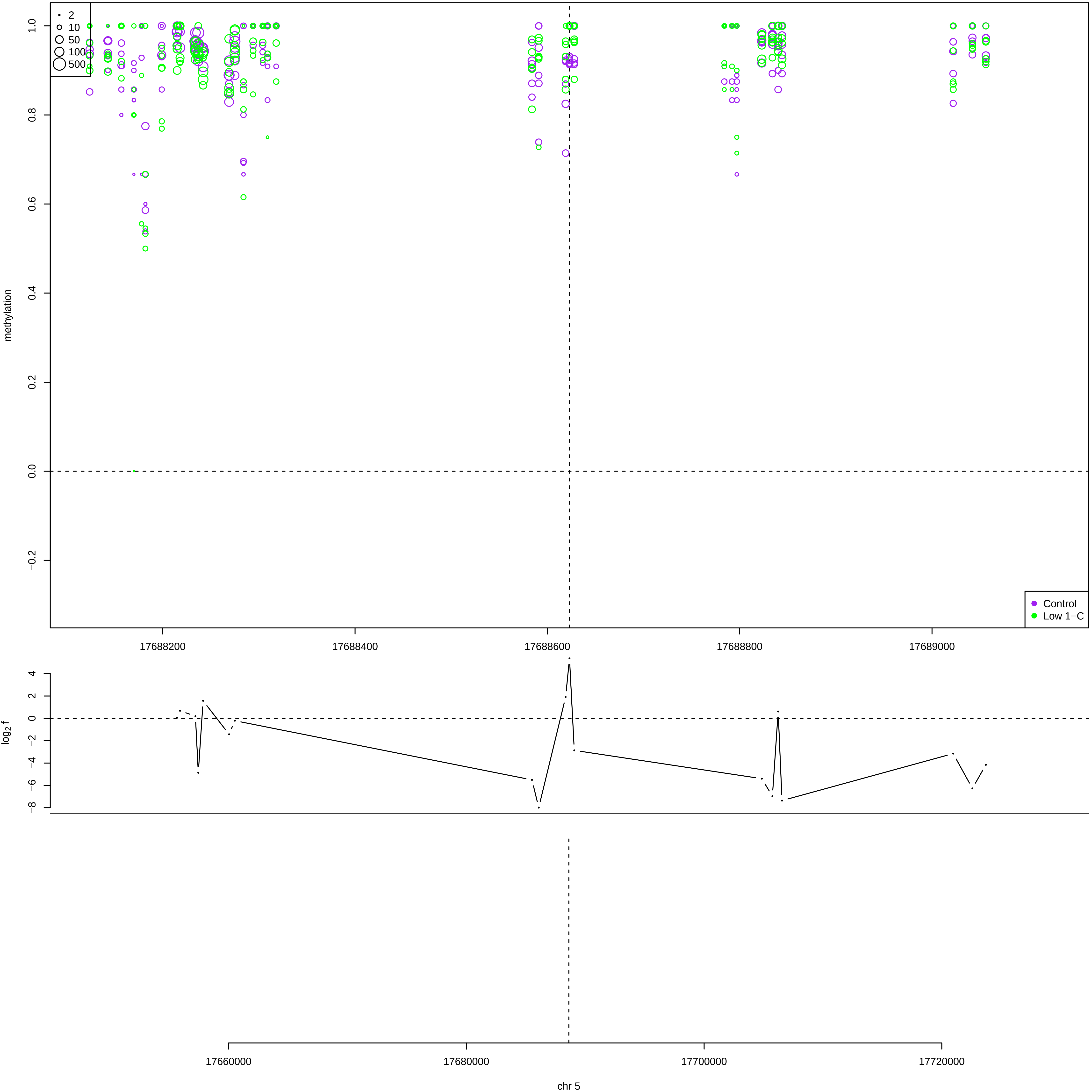

2
10
50
100
500
methylation
1.0
0.8
0.6
0.4
0.2
0.0
−0.2
Control
Low 1−C
17688200
17688400
17688600
17688800
17689000
4
2
0
−2
−4
−6
−8
$\log_2 f$
17660000
17680000
17700000
17720000
chr 5

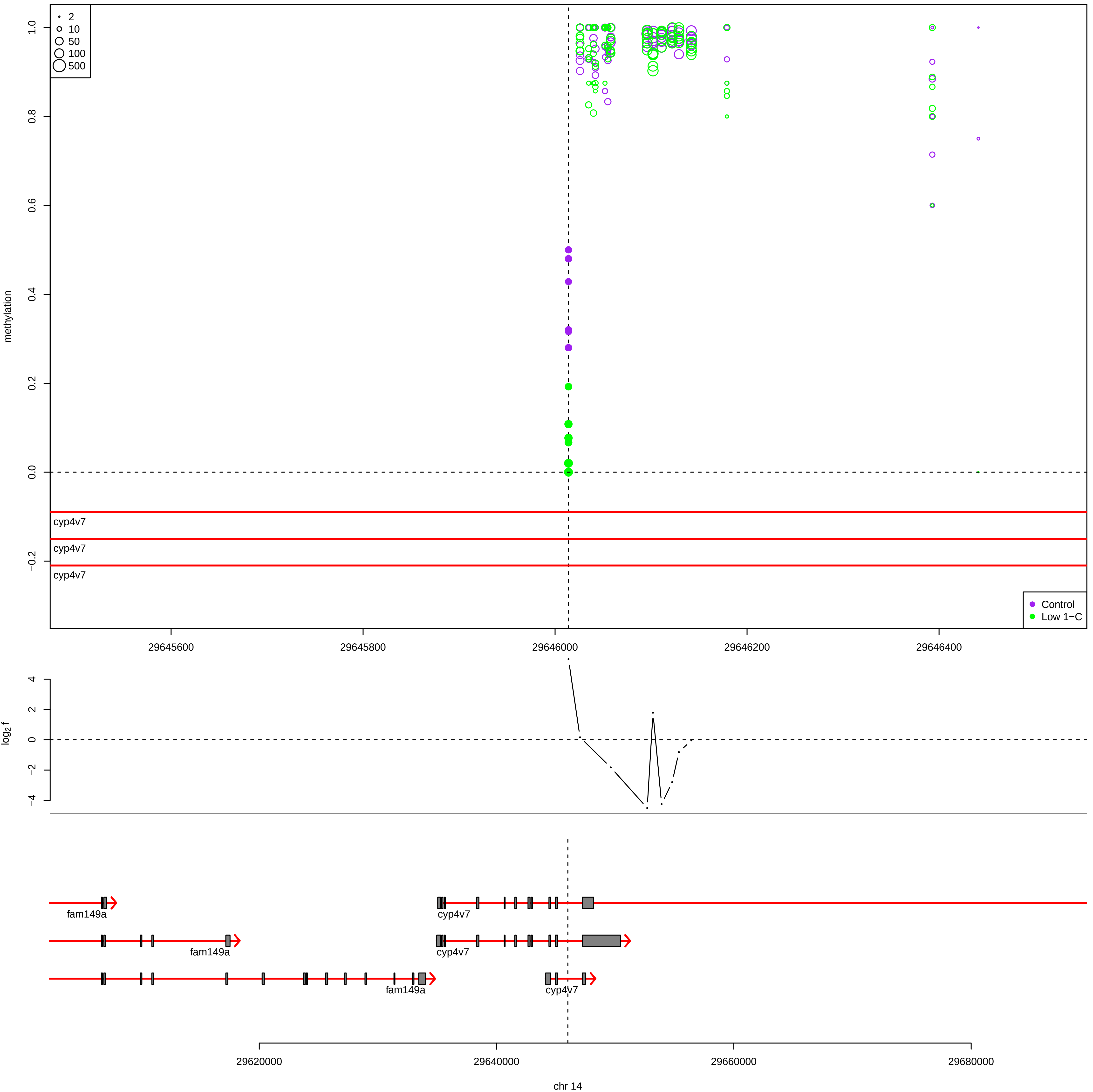
2
10
50
100
500
methylation
1.0
0.8
0.6
0.4
0.2
0.0
−0.2
cyp4v7
cyp4v7
cyp4v7
Control
Low 1−C
29645600
29645800
29646000
29646200
29646400
$\log_2$ f
4
2
0
−2
−4
fam149a
fam149a
fam149a
cyp4v7
cyp4v7
cyp4v7
29620000
29640000
29660000
29680000
chr 14

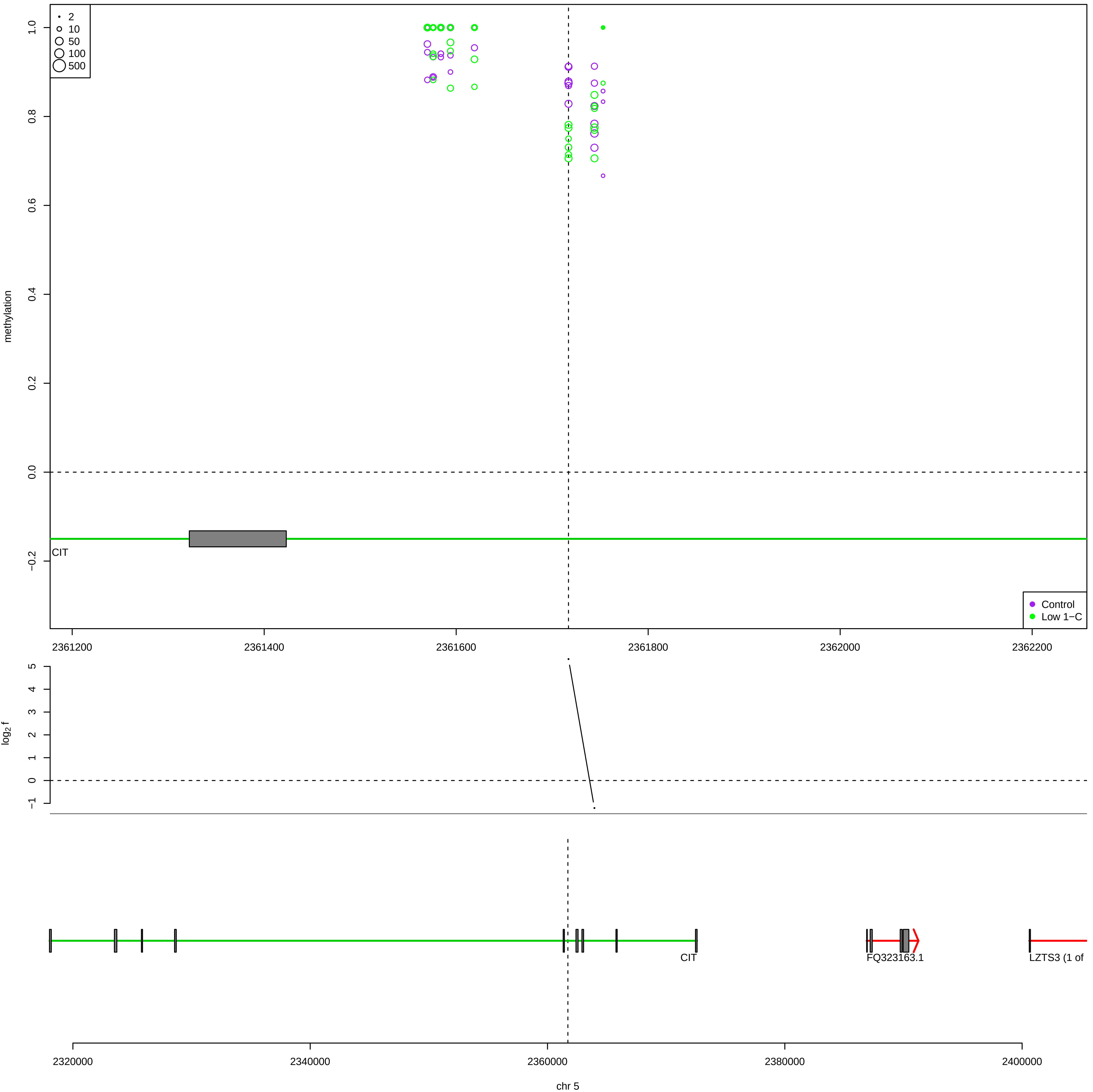

2
10
50
100
500
1.0
0.8
0.6
0.4
0.2
0.0
−0.2
methylation
CIT
Control
Low 1−C
2361200
2361400
2361600
2361800
2362000
2362200
5
4
3
2
1
0
−1
log2 f
CIT
FQ323163.1
LZTS3 (1 of
2320000
2340000
2360000
2380000
2400000
chr 5

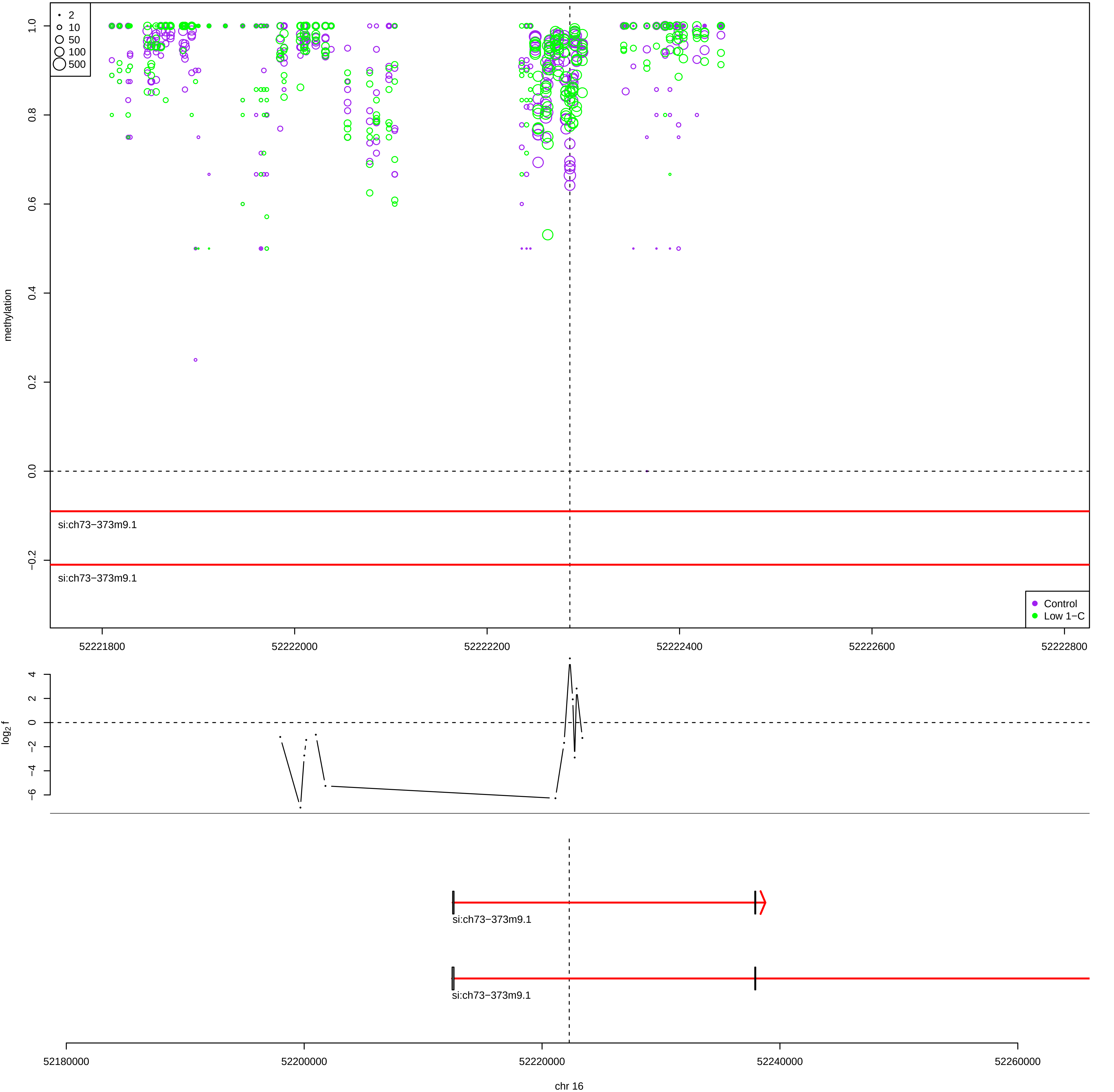

2
10
50
100
500
methylation
1.0
0.8
0.6
0.4
0.2
0.0
−0.2
si:ch73−373m9.1
si:ch73−373m9.1
Control
Low 1−C
52221800
52222000
52222200
52222400
52222600
52222800
log2 f
4
2
0
−2
−4
−6
si:ch73−373m9.1
si:ch73−373m9.1
52180000
52200000
52220000
52240000
52260000
chr 16

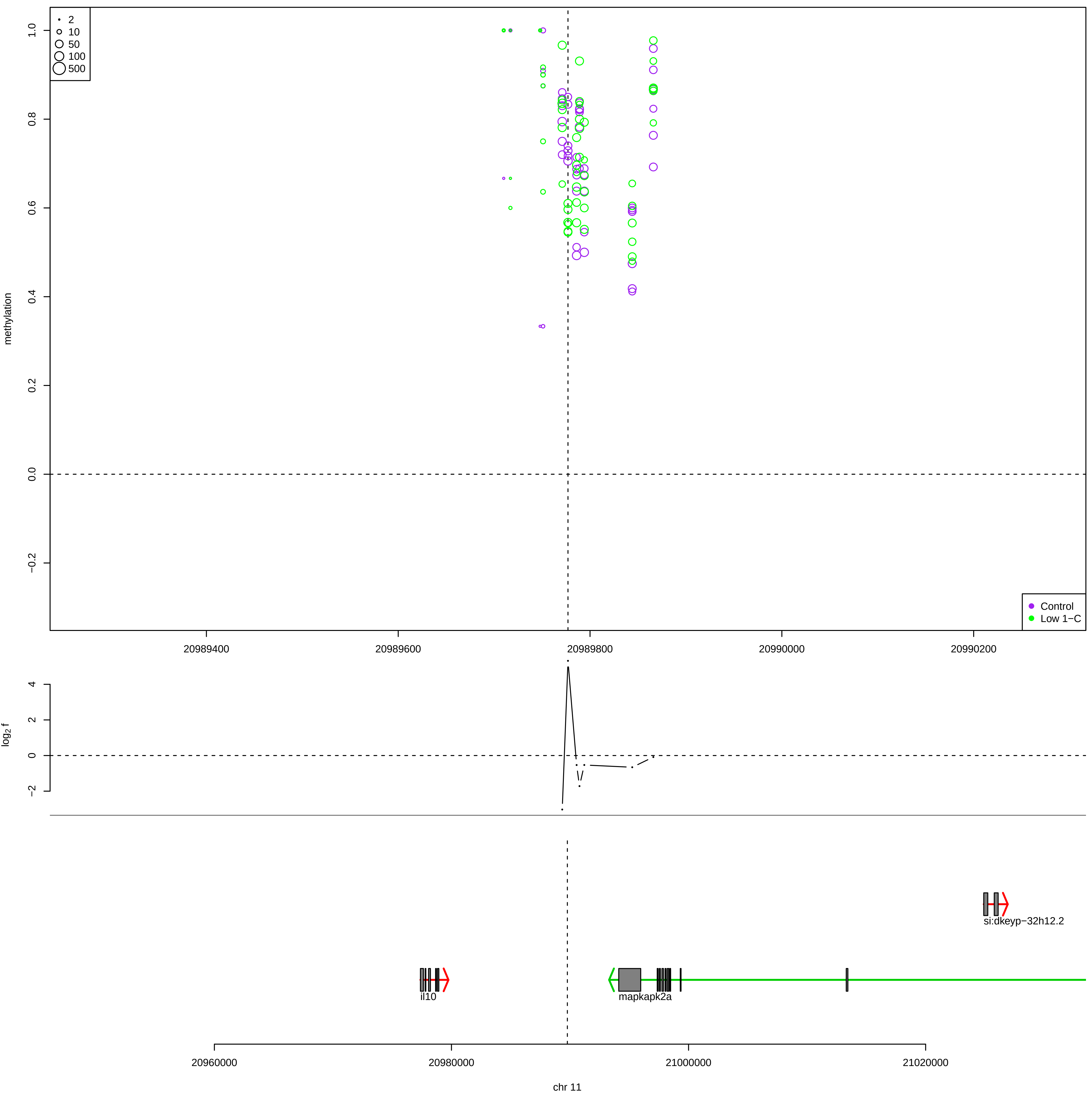

2
10
50
100
500
methylation
1.0
0.8
0.6
0.4
0.2
0.0
−0.2
Control
Low 1−C
20989400
20989600
20989800
20990000
20990200
log2 f
4
2
0
−2
si:dkeyp−32h12.2
il10
mapkapk2a
20960000
20980000
21000000
21020000
chr 11

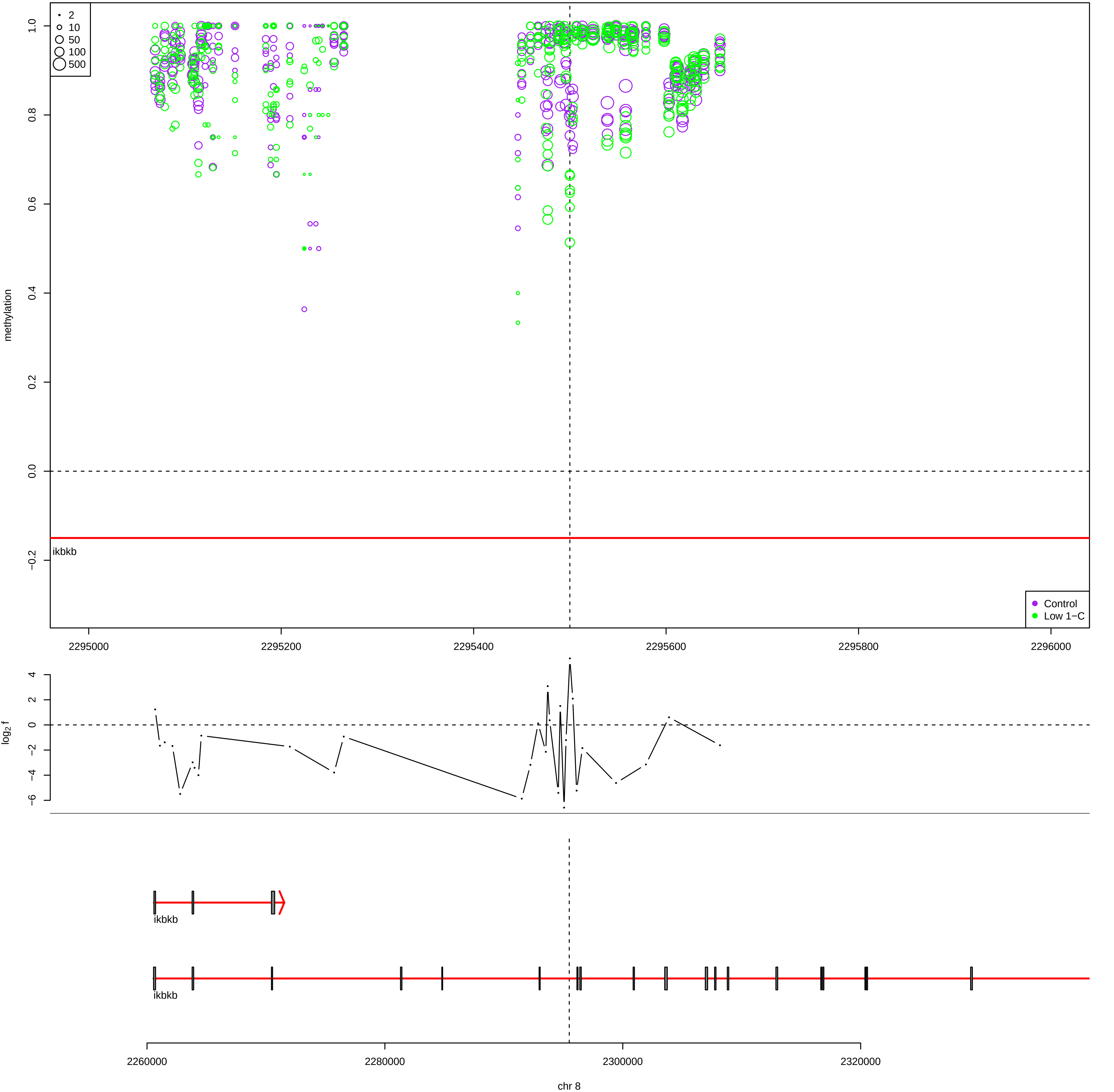

2
10
50
100
500
1.0
0.8
0.6
0.4
0.2
0.0
-0.2
methylation
ikbkb
Control
Low 1−C
2295000
2295200
2295400
2295600
2295800
2296000
4
2
0
-2
-4
-6
log_2 f
ikbkb
ikbkb
2260000
2280000
2300000
2320000
chr 8

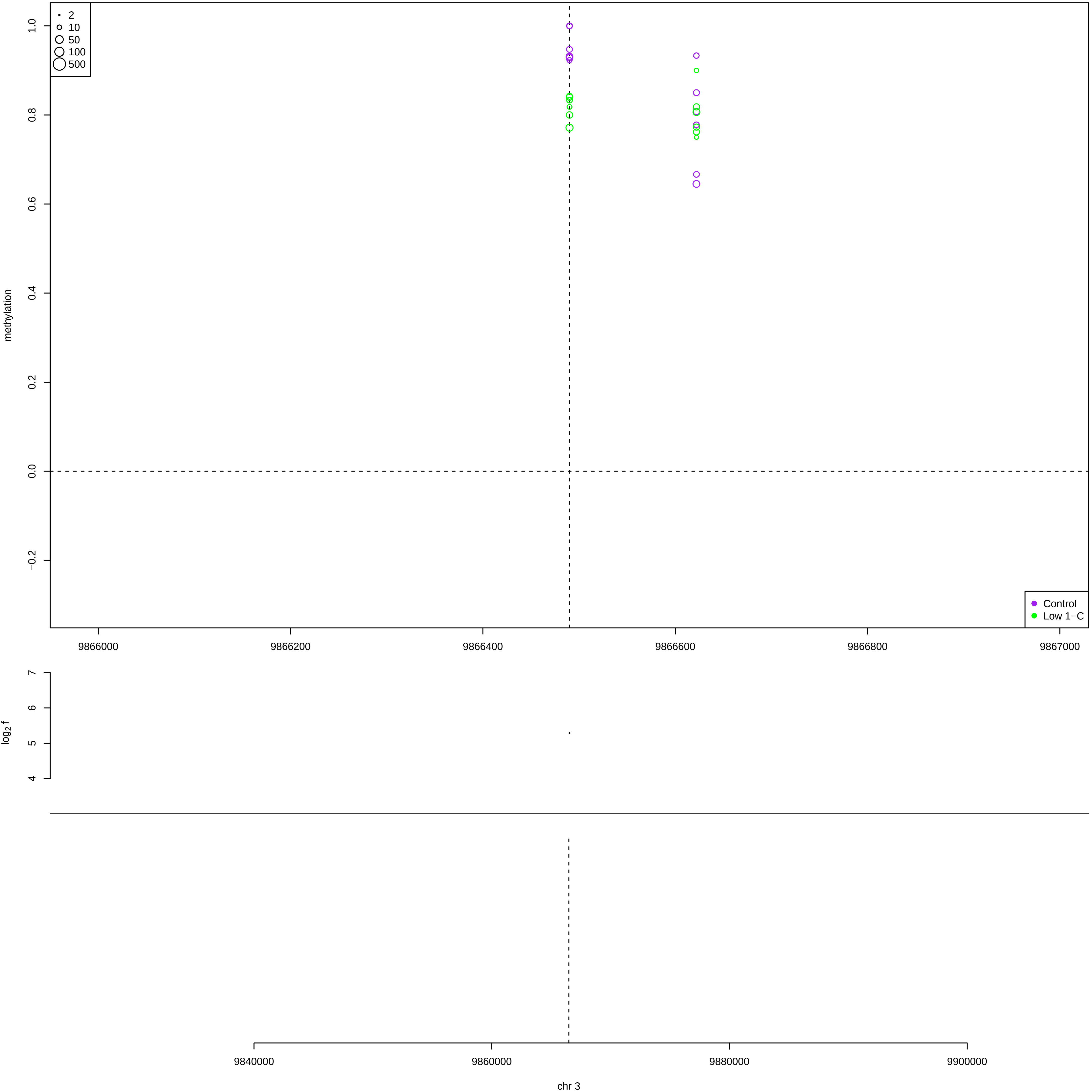

2
10
50
100
500
methylation
1.0
0.8
0.6
0.4
0.2
0.0
−0.2
Control
Low 1−C
9866000
9866200
9866400
9866600
9866800
9867000
$\log_2 f$
7
6
5
4
9840000
9860000
9880000
9900000
chr 3

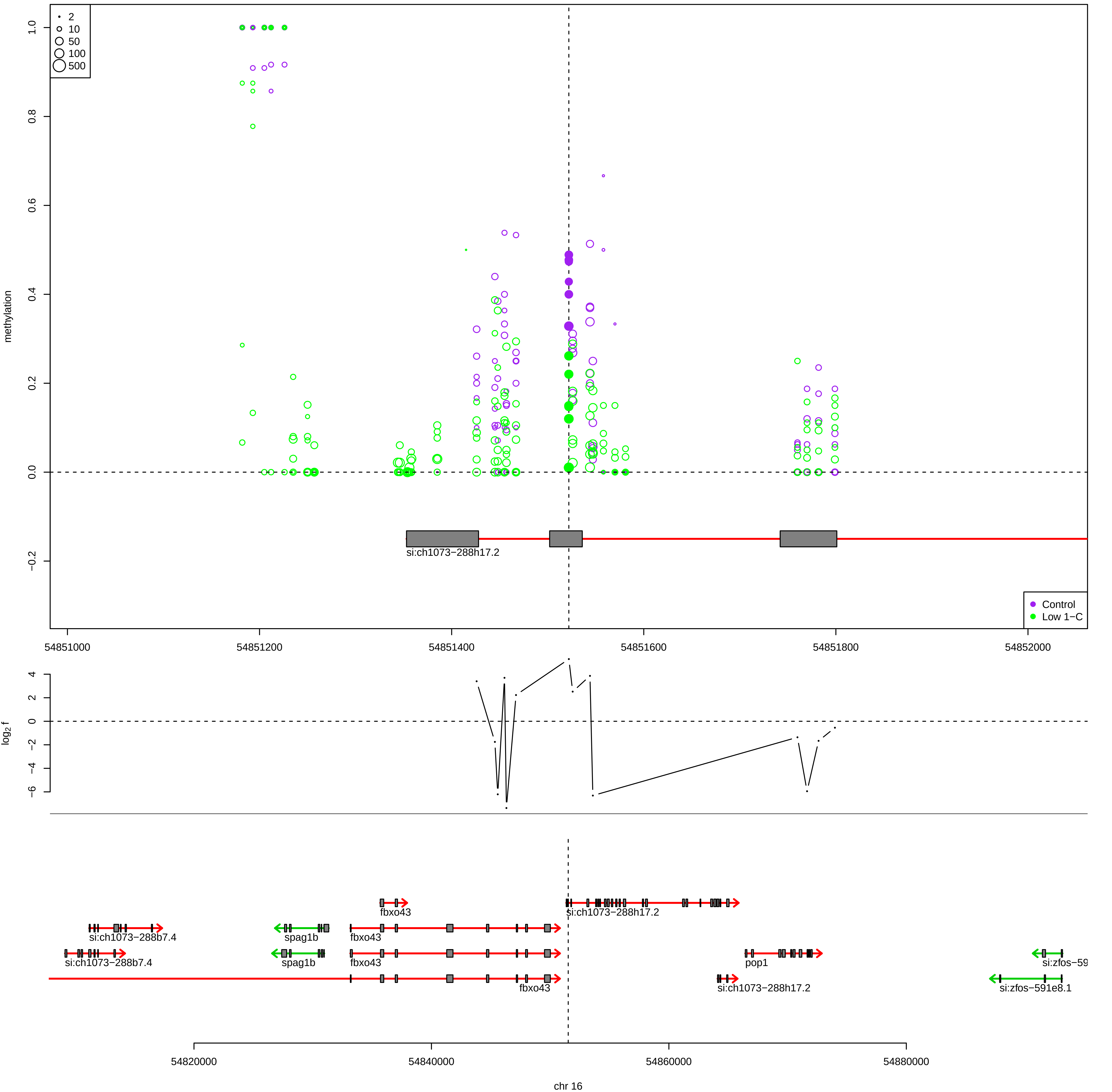

methylation
2
10
50
100
500
1.0
0.8
0.6
0.4
0.2
0.0
−0.2
si:ch1073−288h17.2
Control
Low 1−C
54851000
54851200
54851400
54851600
54851800
54852000
log2 f
4
2
0
−2
−4
−6
fbxo43
si:ch1073−288h17.2
si:ch1073−288b7.4
spag1b
fbxo43
si:ch1073−288b7.4
spag1b
fbxo43
pop1
fbxo43
si:ch1073−288h17.2
si:zfos−591e8.1
54820000
54840000
54860000
54880000
chr 16

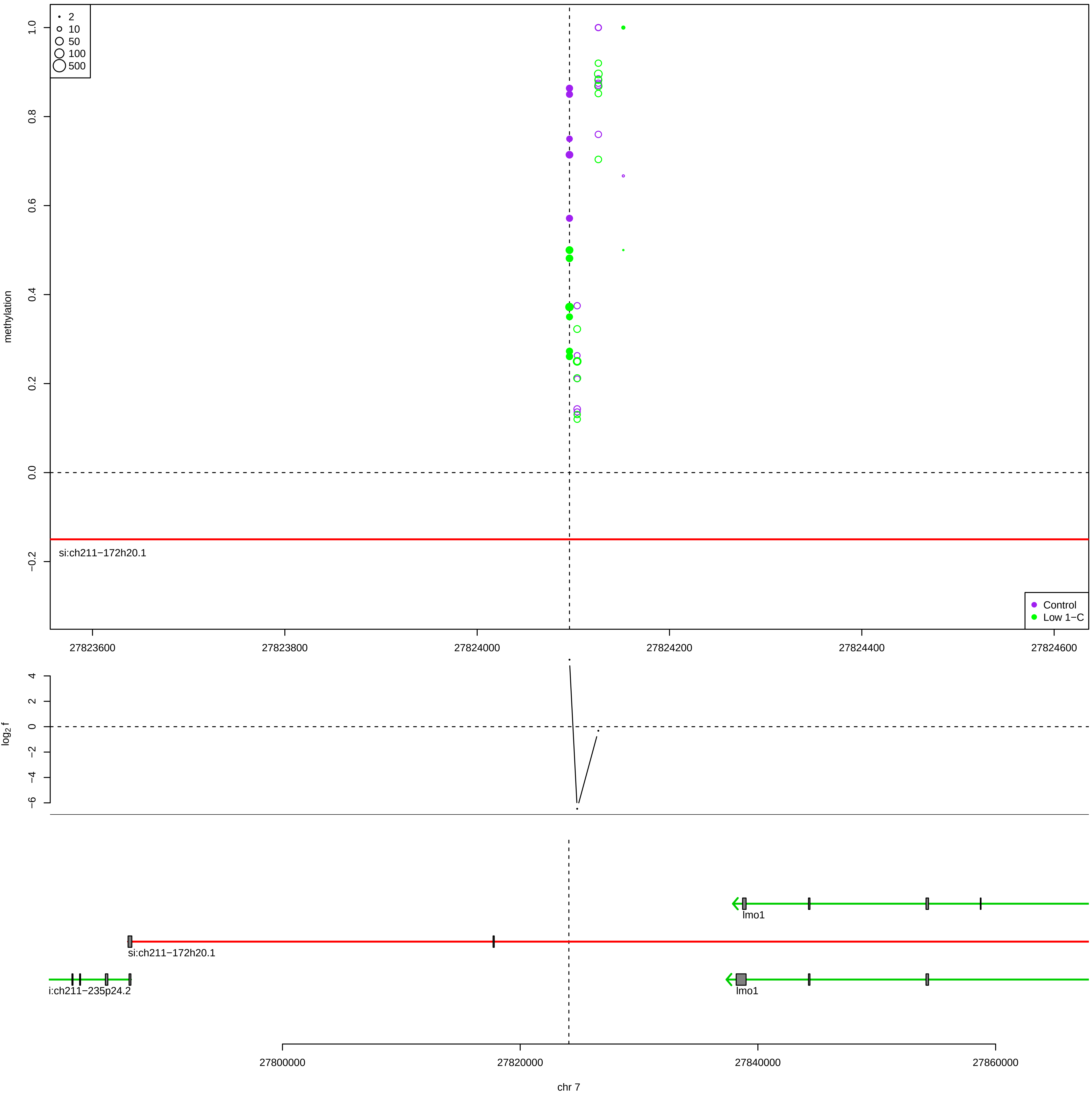

2
10
50
100
500
methylation
1.0
0.8
0.6
0.4
0.2
0.0
−0.2
si:ch211−172h20.1
Control
Low 1−C
27823600
27823800
27824000
27824200
27824400
27824600
log2 f
4
2
0
−2
−4
−6
lmo1
si:ch211−172h20.1
i:ch211−235p24.2
lmo1
27800000
27820000
27840000
27860000
chr 7

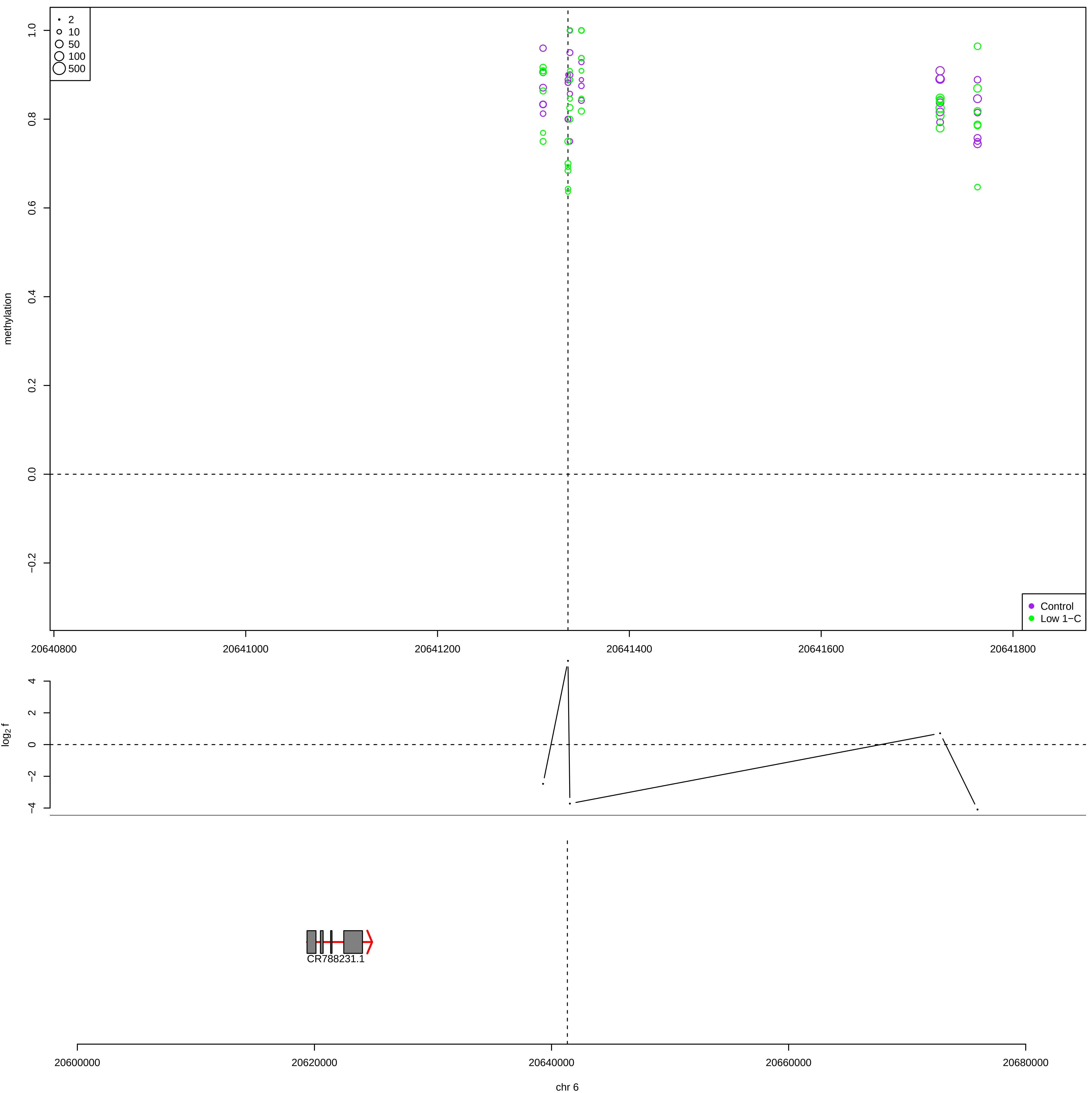

2
10
50
100
500
Control
Low 1−C
methylation
1.0
0.8
0.6
0.4
0.2
0.0
−0.2
20640800
20641000
20641200
20641400
20641600
20641800
log2 f
4
2
0
−2
−4
CR788231.1
20600000
20620000
20640000
20660000
20680000
chr 6

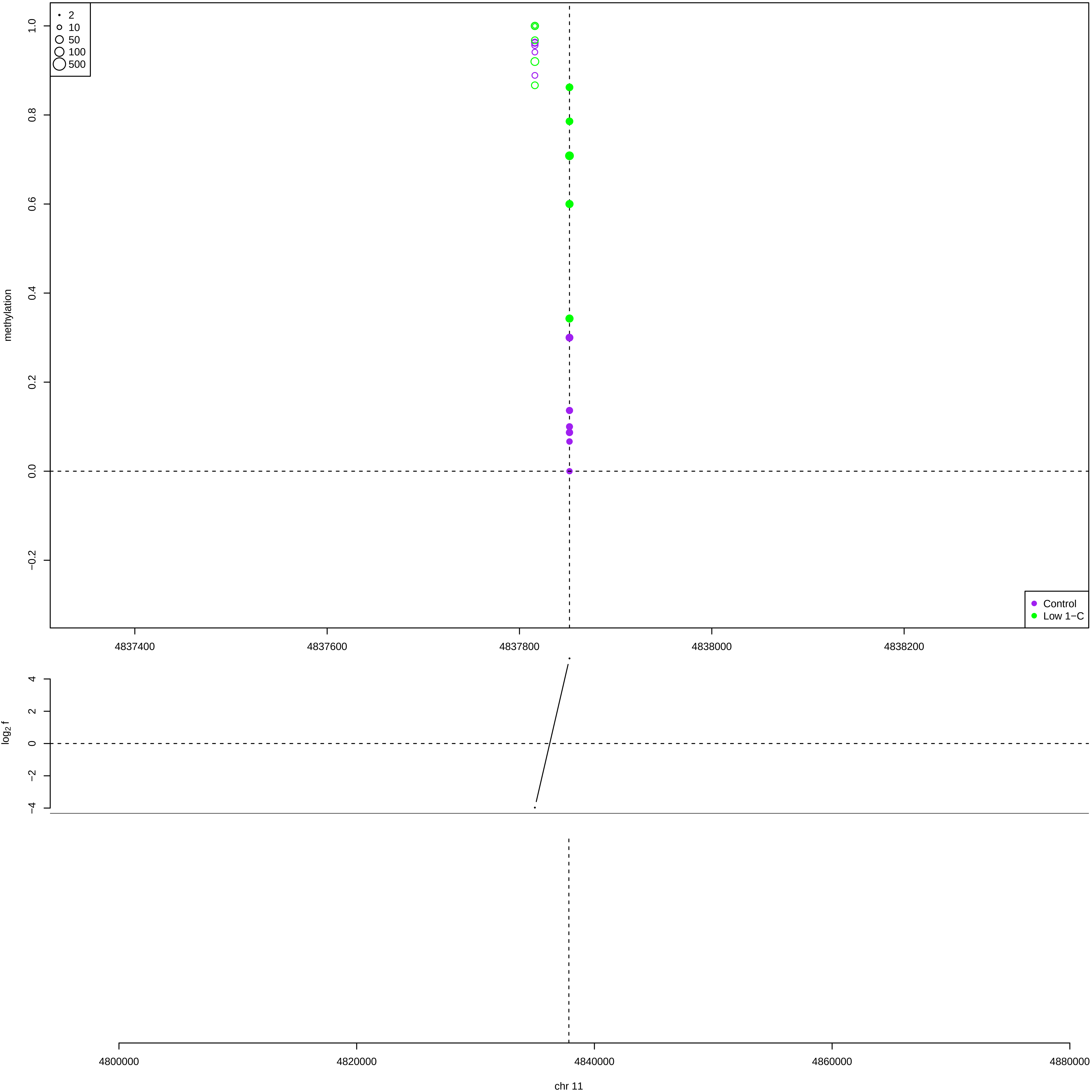

2
10
50
100
500
methylation
1.0
0.8
0.6
0.4
0.2
0.0
−0.2
Control
Low 1−C
4837400
4837600
4837800
4838000
4838200
log_2 f
4
2
0
−2
−4
4800000
4820000
4840000
4860000
4880000
chr 11

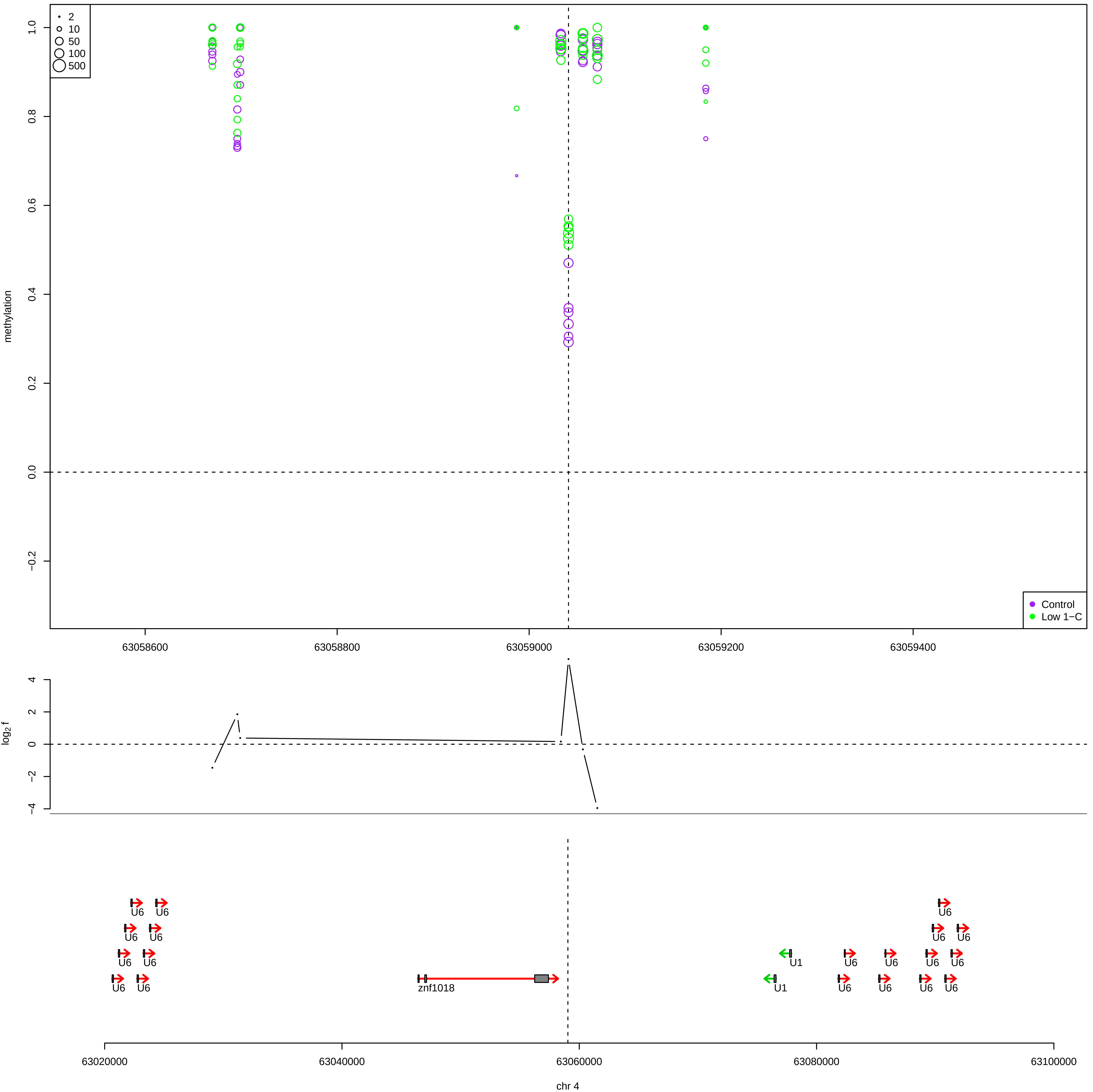

2
10
50
100
500
methylation
1.0
0.8
0.6
0.4
0.2
0.0
−0.2
Control
Low 1−C
63058600
63058800
63059000
63059200
63059400
log2 f
4
2
0
−2
−4
U6
U1
znf1018
63020000
63040000
63060000
63080000
63100000
chr 4

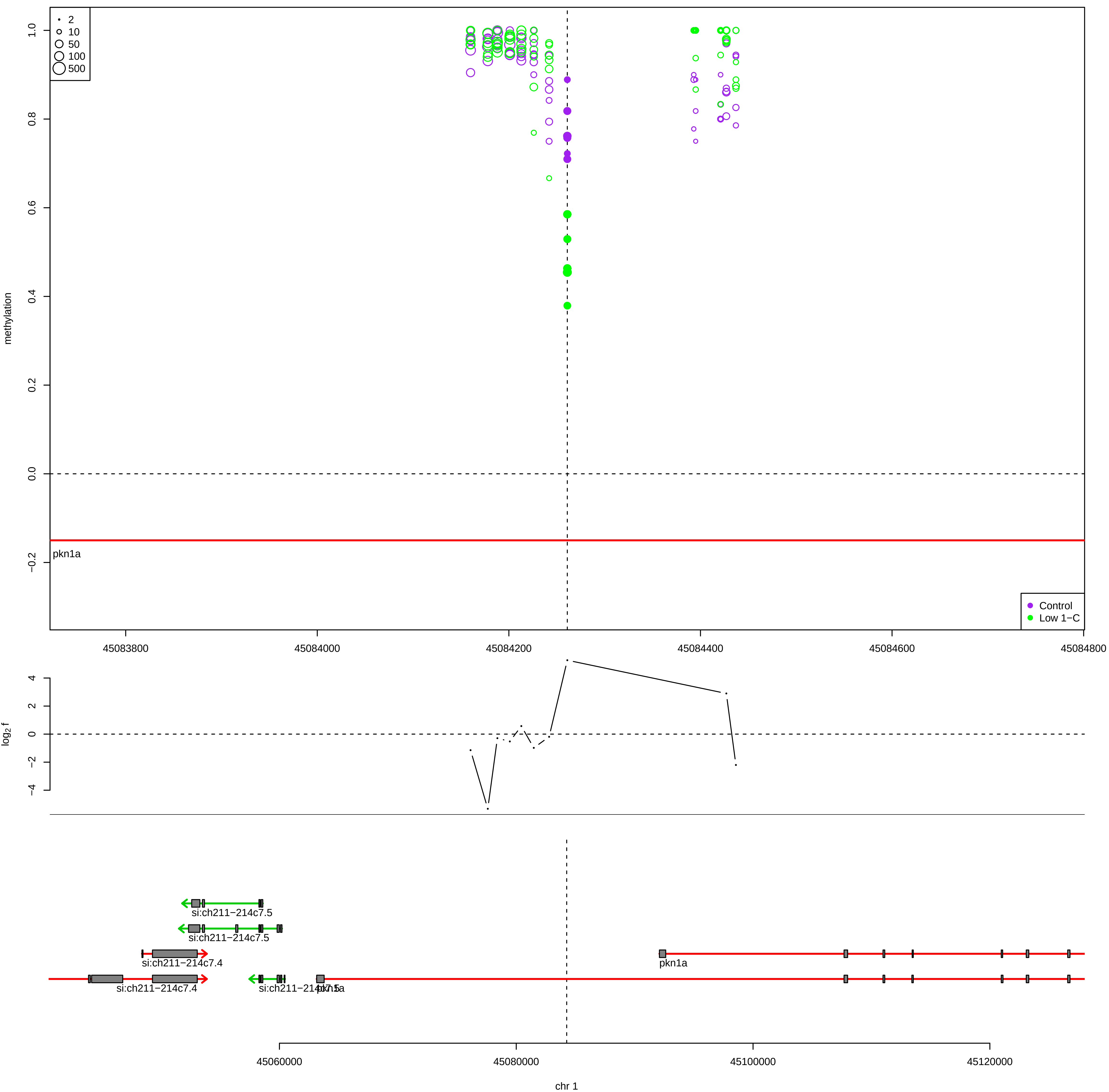

2
10
50
100
500
methylation
1.0
0.8
0.6
0.4
0.2
0.0
−0.2
pkn1a
Control
Low 1−C
45083800
45084000
45084200
45084400
45084600
45084800
log2 f
4
2
0
−2
−4
si:ch211−214c7.5
si:ch211−214c7.5
si:ch211−214c7.4
si:ch211−214c7.4
pkn1a
45060000
45080000
45100000
45120000
chr 1

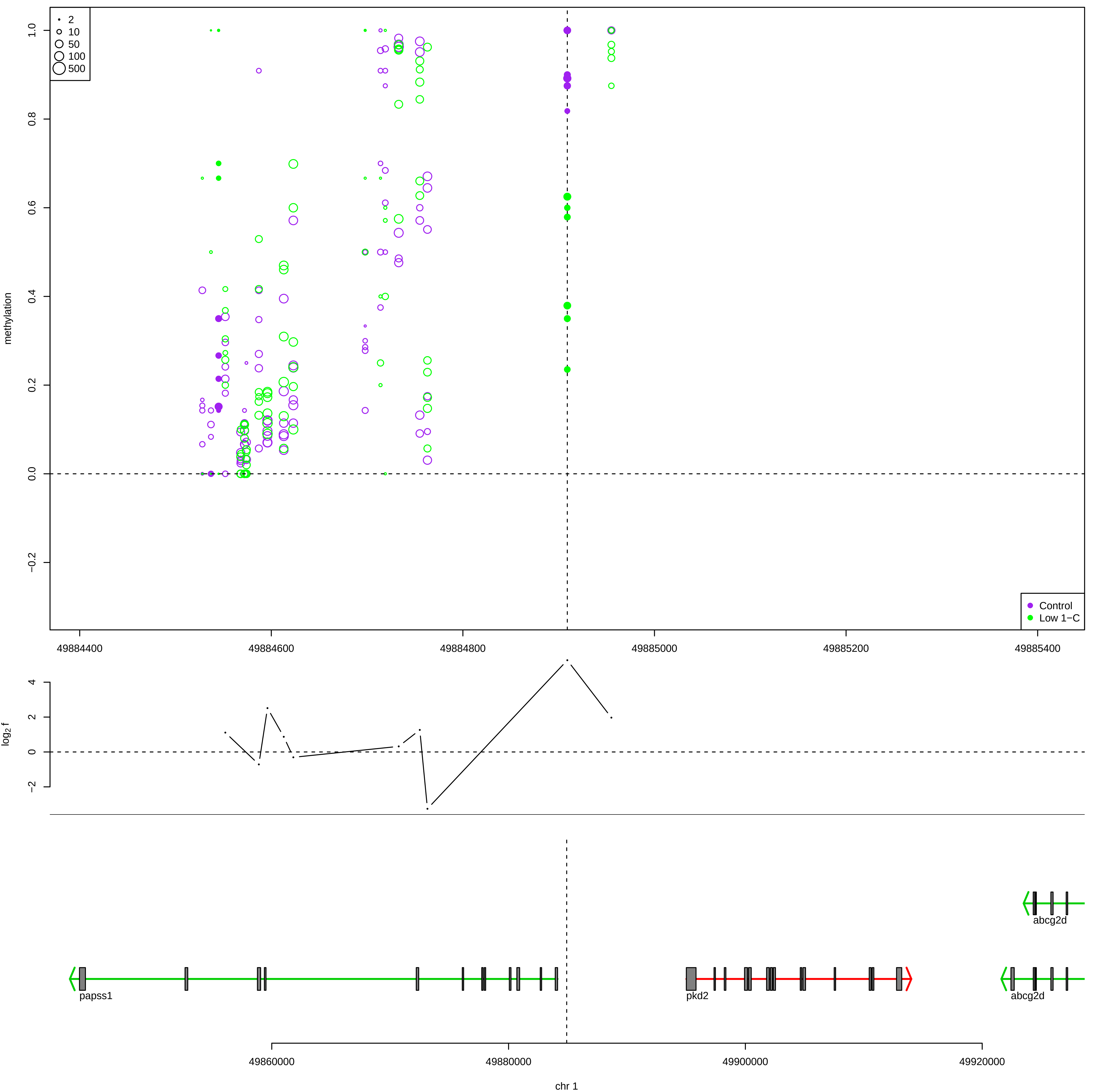

2
10
50
100
500
methylation
1.0
0.8
0.6
0.4
0.2
0.0
−0.2
Control
Low 1−C
49884400
49884600
49884800
49885000
49885200
49885400
log2 f
4
2
0
−2
abcg2d
papss1
pkd2
abcg2d
49860000
49880000
49900000
49920000
chr 1

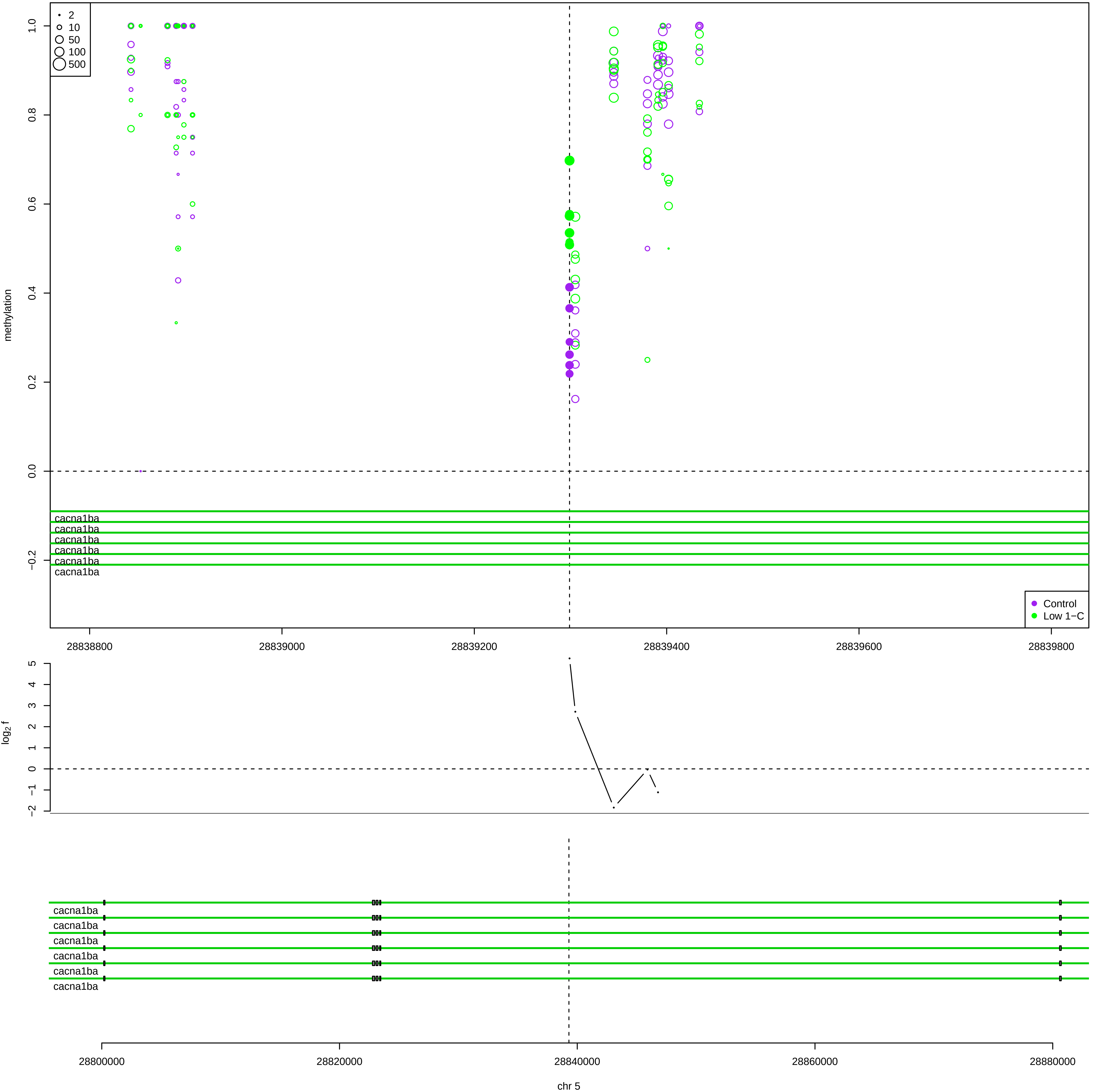

2
10
50
100
500
methylation
1.0
0.8
0.6
0.4
0.2
0.0
−0.2
cacna1ba
cacna1ba
cacna1ba
cacna1ba
cacna1ba
cacna1ba
Control
Low 1−C
28838800
28839000
28839200
28839400
28839600
28839800
log2 f
5
4
3
2
1
0
−1
−2
cacna1ba
cacna1ba
cacna1ba
cacna1ba
cacna1ba
cacna1ba
28800000
28820000
28840000
28860000
28880000
chr 5

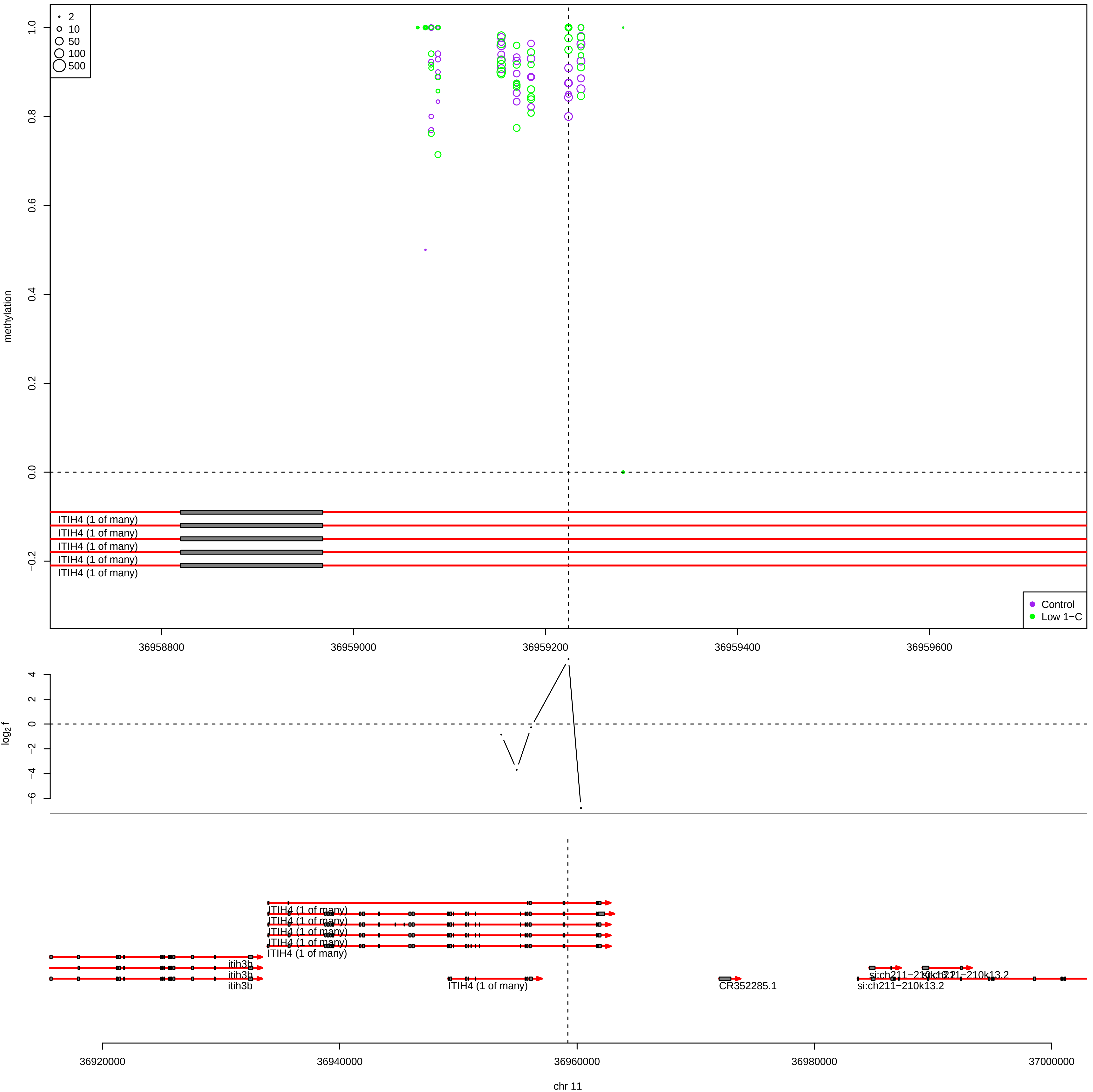

2
10
50
100
500
1.0
0.8
0.6
0.4
0.2
0.0
−0.2
methylation
ITIH4 (1 of many)
ITIH4 (1 of many)
ITIH4 (1 of many)
ITIH4 (1 of many)
ITIH4 (1 of many)
Control
Low 1−C
36958800
36959000
36959200
36959400
36959600
log2 f
4
2
0
−2
−4
−6
ITIH4 (1 of many)
ITIH4 (1 of many)
ITIH4 (1 of many)
ITIH4 (1 of many)
ITIH4 (1 of many)
itih3b
itih3b
itih3b
ITIH4 (1 of many)
CR352285.1
si:ch211−210k13.2
si:ch211−210k13.2
si:ch211−210k13.2
36920000
36940000
36960000
36980000
37000000
chr 11

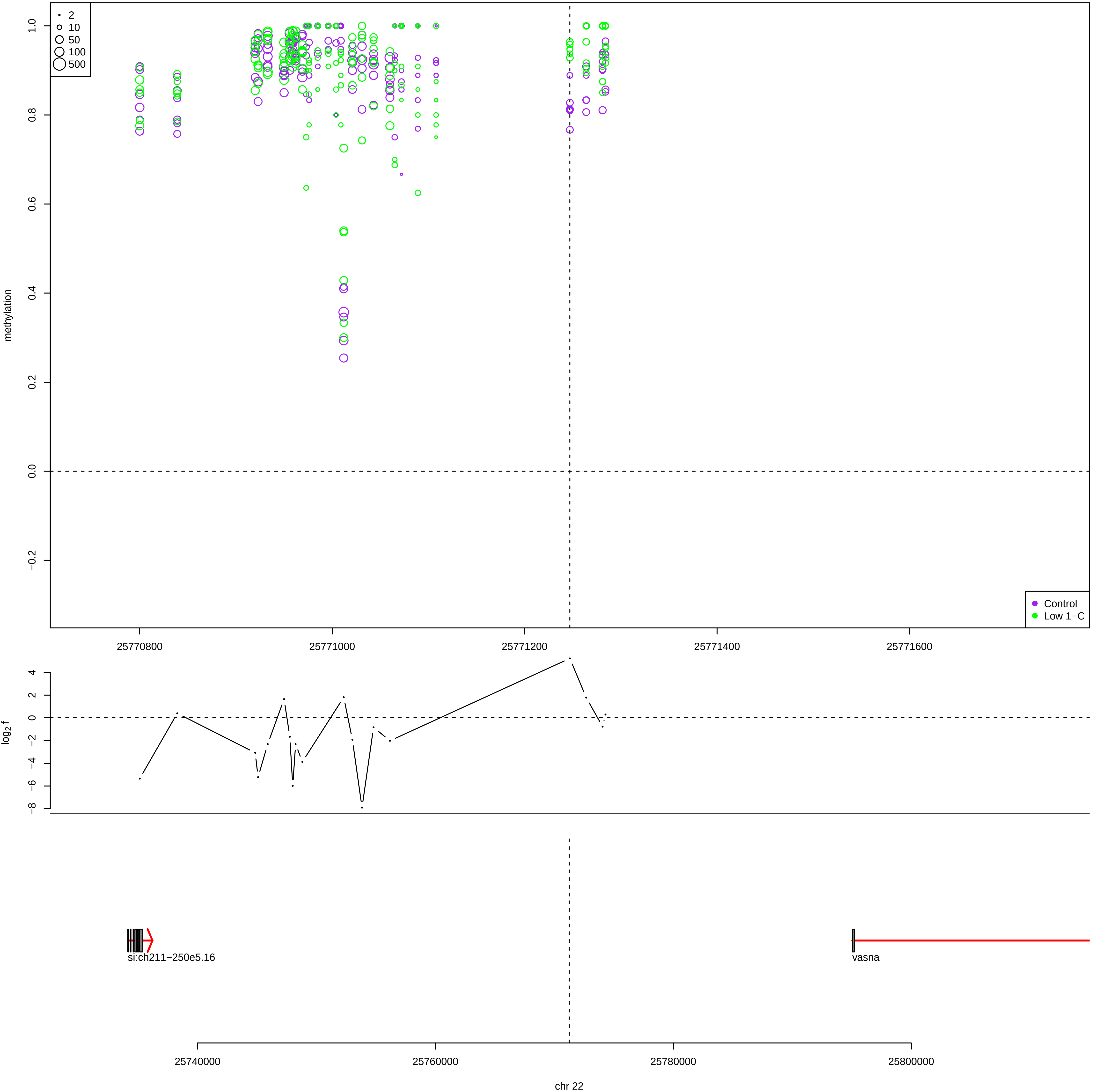

2
10
50
100
500
methylation
1.0
0.8
0.6
0.4
0.2
0.0
−0.2
Control
Low 1−C
25770800
25771000
25771200
25771400
25771600
log2 f
4
2
0
−2
−4
−6
−8
si:ch211−250e5.16
vasna
25740000
25760000
25780000
25800000
chr 22

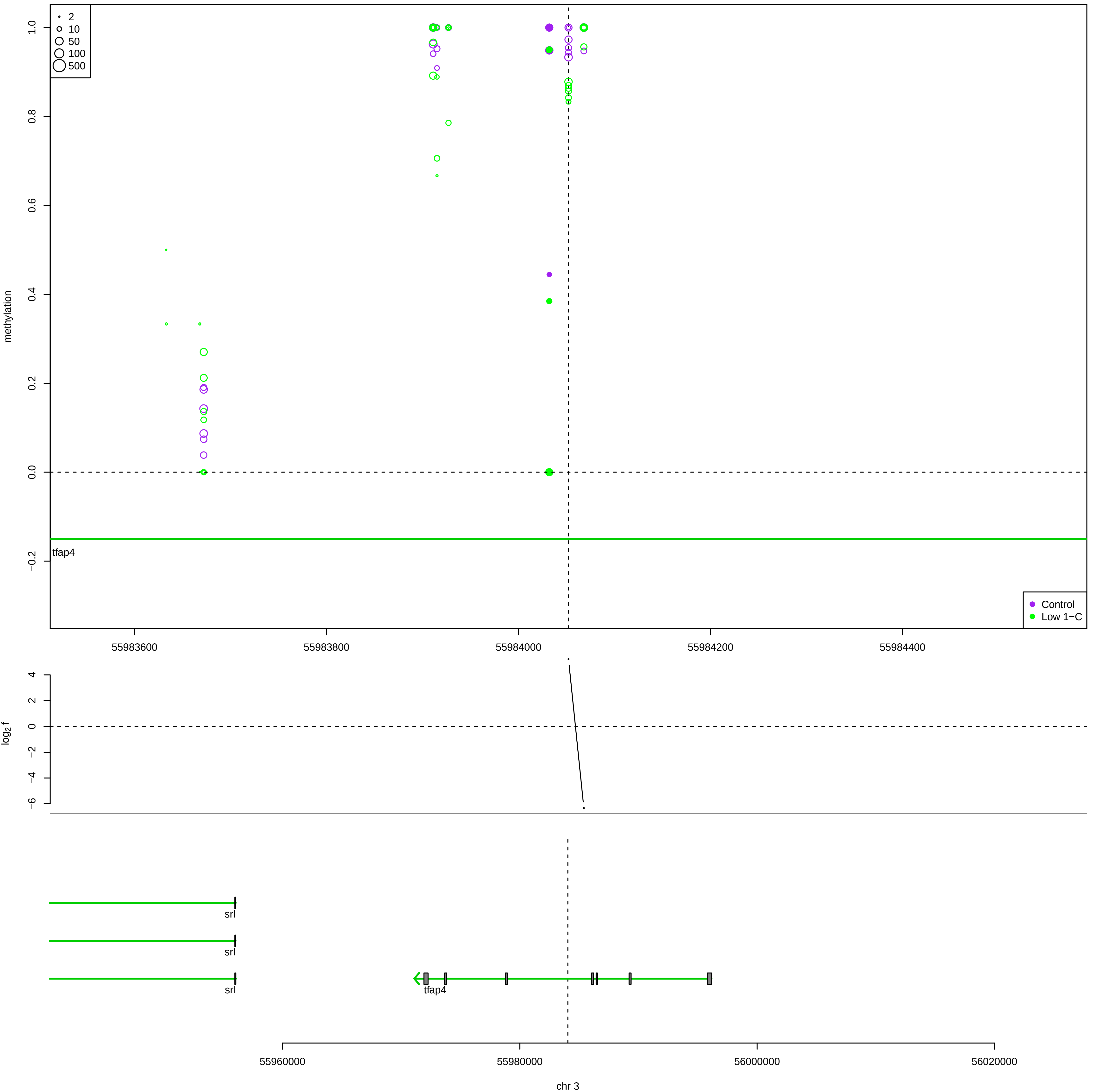

2
10
50
100
500
methylation
1.0
0.8
0.6
0.4
0.2
0.0
−0.2
tfap4
Control
Low 1−C
55983600
55983800
55984000
55984200
55984400
log2 f
4
2
0
−2
−4
−6
srl
srl
srl
tfap4
55960000
55980000
56000000
56020000
chr 3

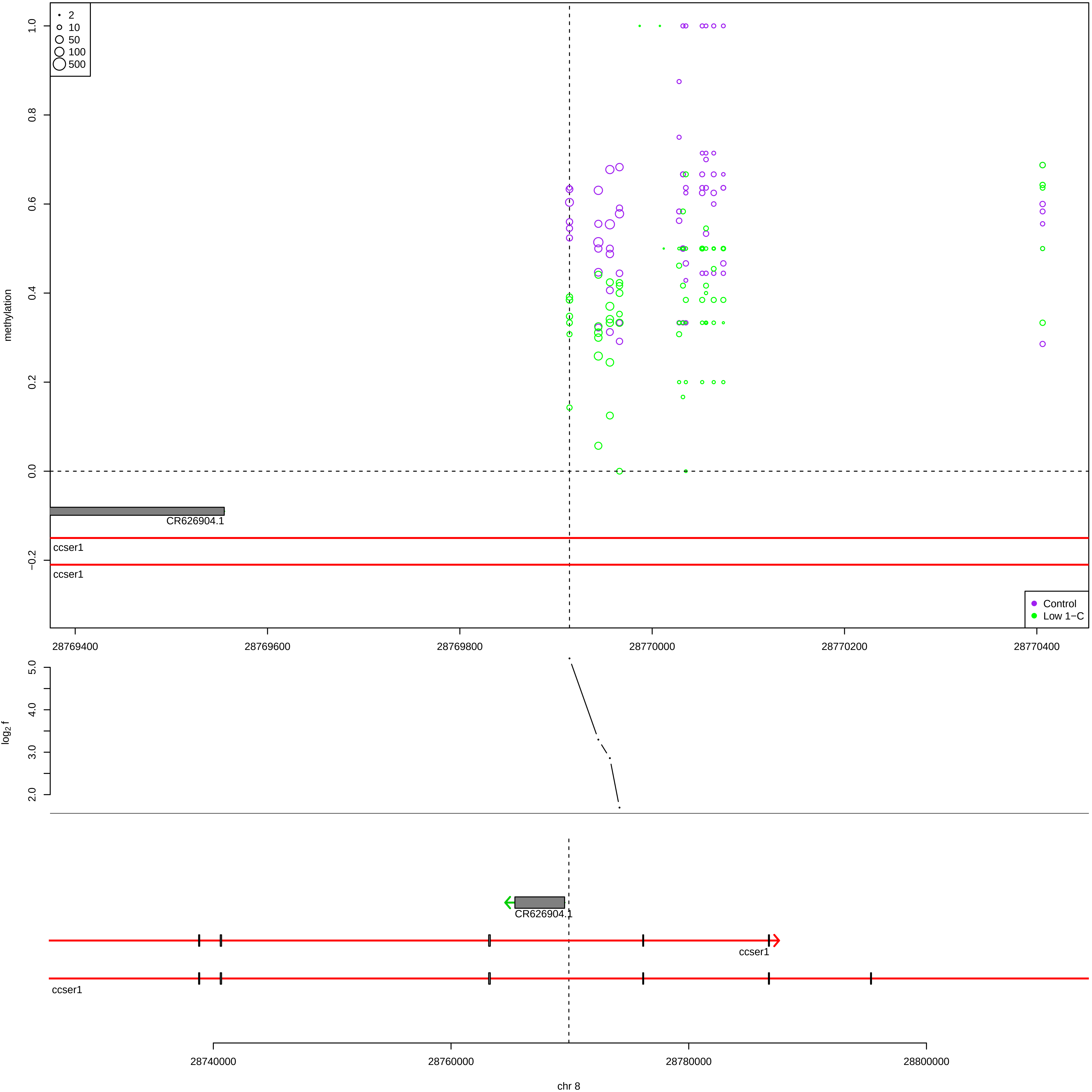

2
10
50
100
500
methylation
1.0
0.8
0.6
0.4
0.2
0.0
−0.2
CR626904.1
ccser1
ccser1
Control
Low 1−C
28769400
28769600
28769800
28770000
28770200
28770400
log2 f
5.0
4.0
3.0
2.0
CR626904.1
ccser1
ccser1
28740000
28760000
28780000
28800000
chr 8

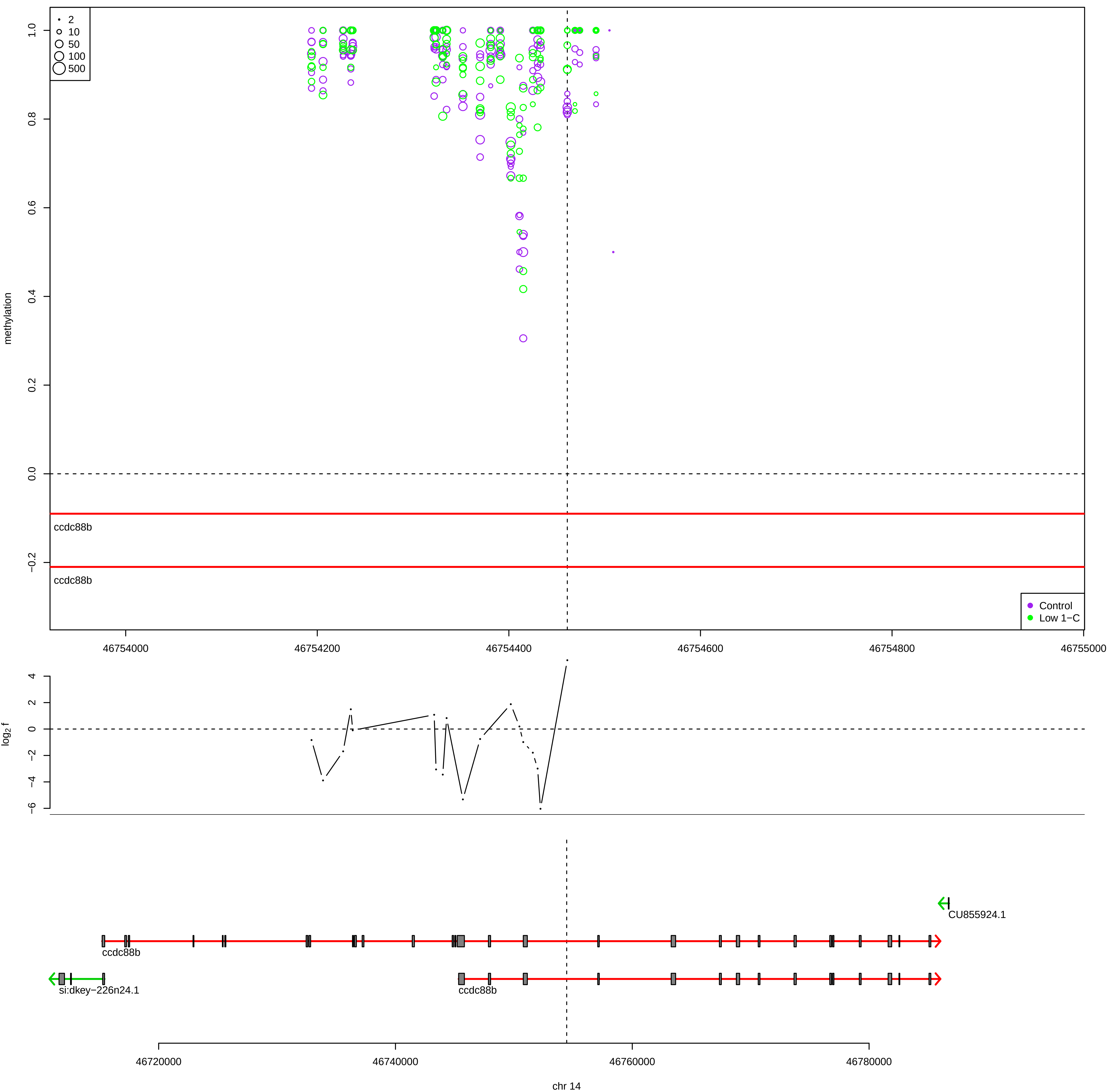

methylation
2
10
50
100
500
Control
Low 1−C
ccdc88b
ccdc88b
46754000
46754200
46754400
46754600
46754800
46755000
log2 f
CU855924.1
ccdc88b
si:dkey−226n24.1
ccdc88b
46720000
46740000
46760000
46780000
chr 14

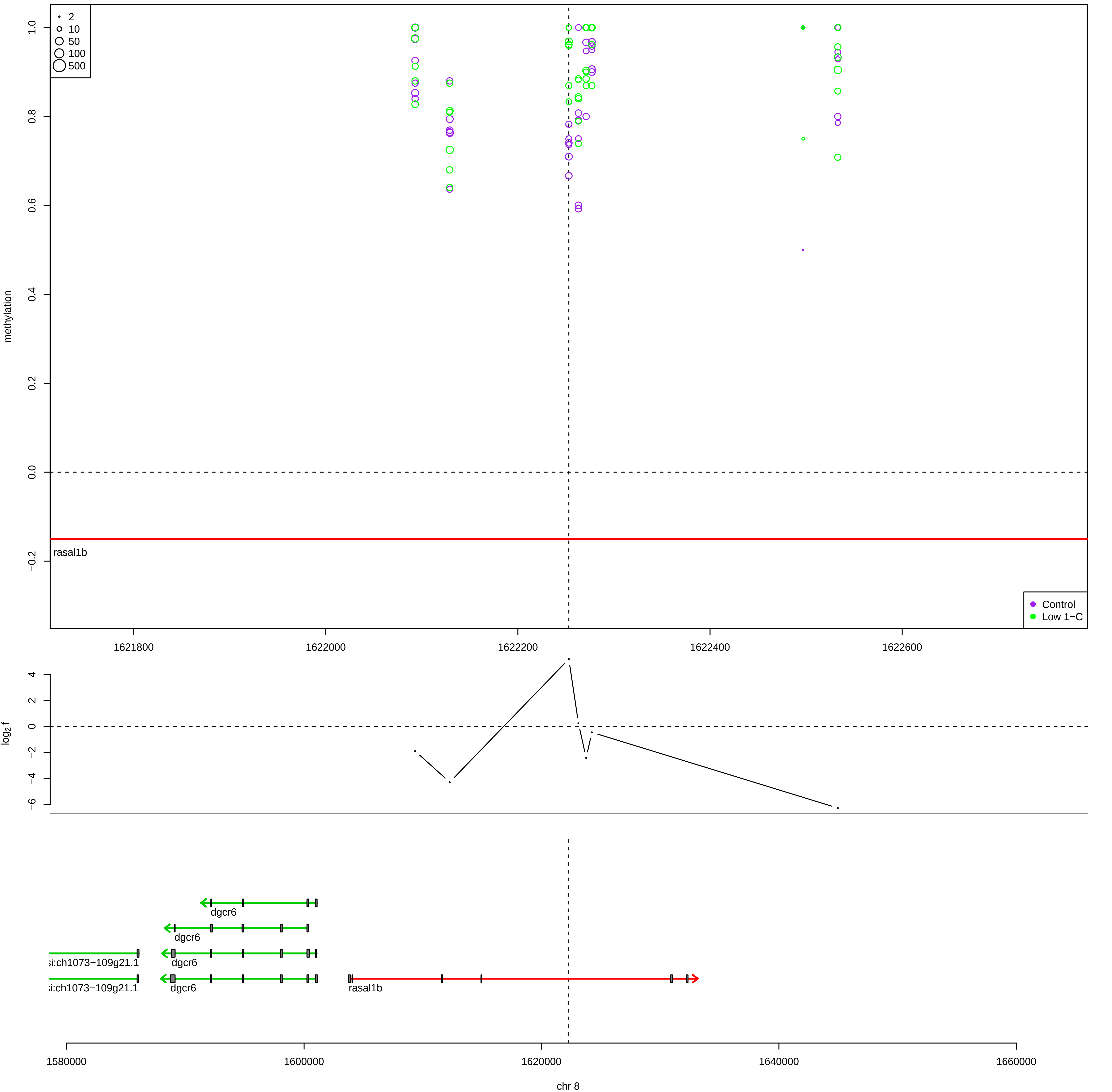

2
10
50
100
500
methylation
1.0
0.8
0.6
0.4
0.2
0.0
−0.2
rasal1b
Control
Low 1−C
1621800
1622000
1622200
1622400
1622600
log2 f
4
2
0
−2
−4
−6
dgcr6
dgcr6
si:ch1073−109g21.1
dgcr6
si:ch1073−109g21.1
dgcr6
rasal1b
1580000
1600000
1620000
1640000
1660000
chr 8

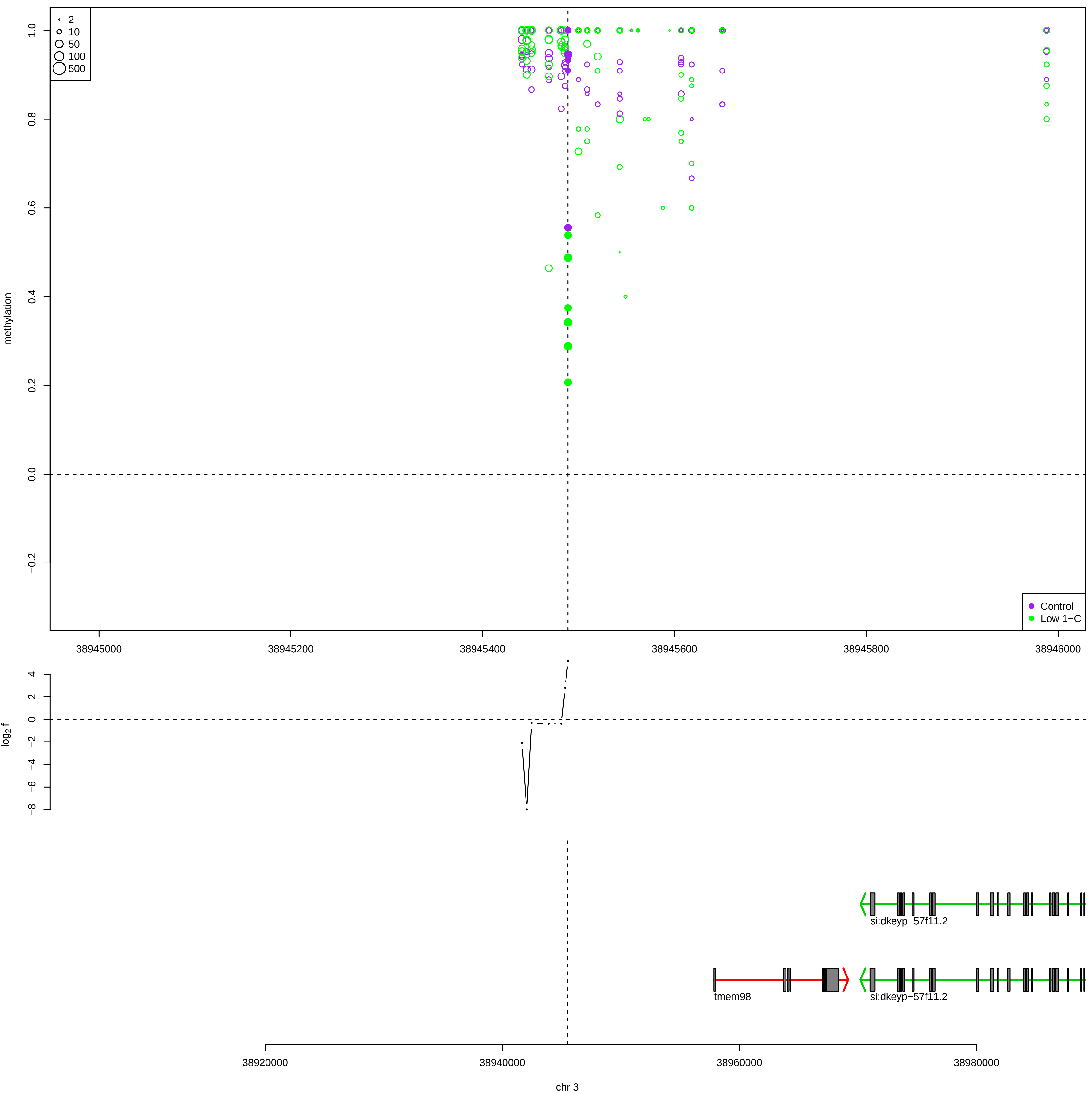

2
10
50
100
500
Control
Low 1−C
methylation
1.0
0.8
0.6
0.4
0.2
0.0
−0.2
38945000
38945200
38945400
38945600
38945800
38946000
log2 f
4
2
0
−2
−4
−6
−8
si:dkeyp−57f11.2
tmem98
si:dkeyp−57f11.2
38920000
38940000
38960000
38980000
chr 3

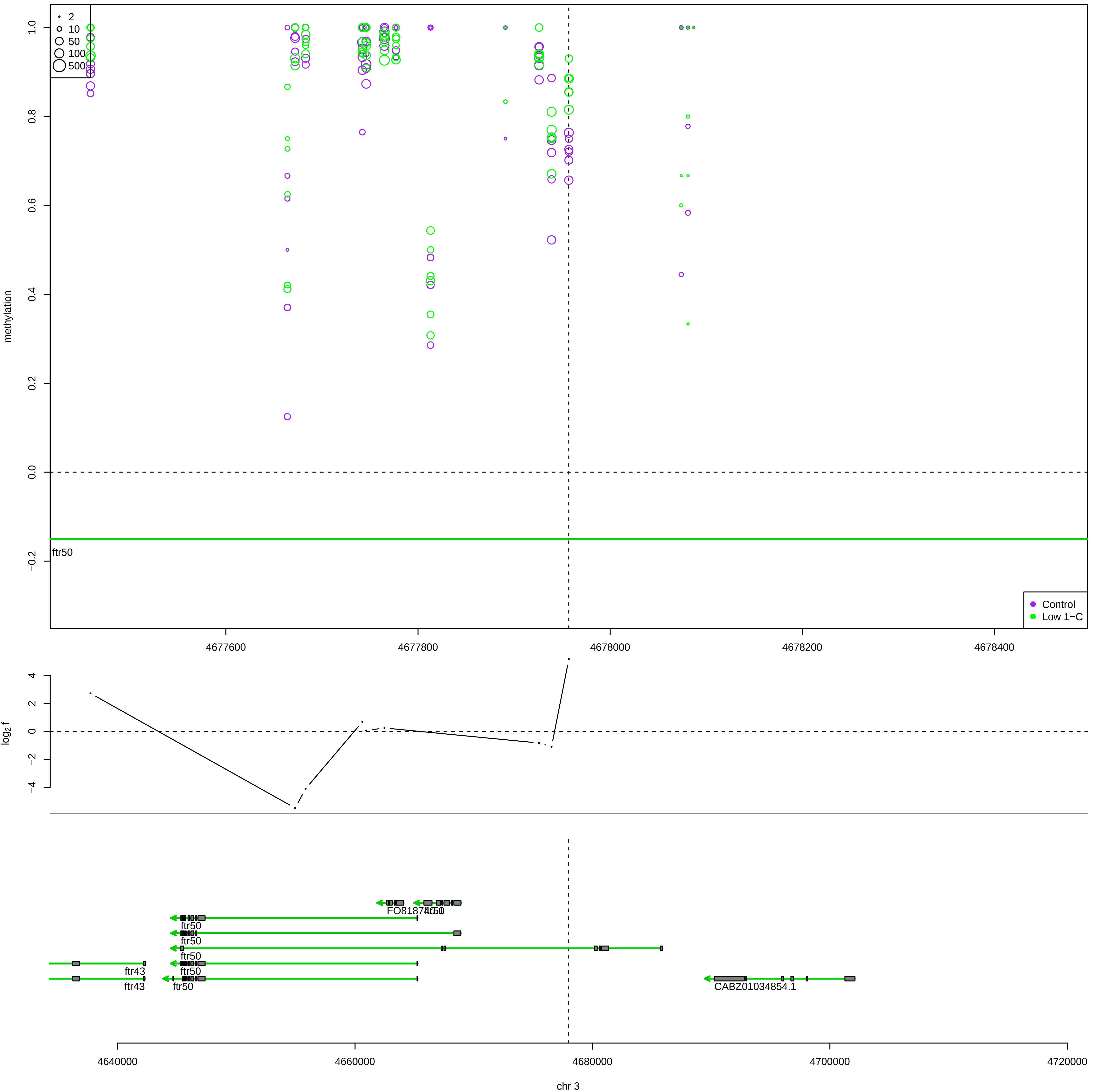

methylation
1.0
0.8
0.6
0.4
0.2
0.0
−0.2
2
10
50
100
500
ftr50
Control
Low 1−C
4677600
4677800
4678000
4678200
4678400
log2 f
4
2
0
−2
−4
FO818740.1
ftr50
ftr50
ftr50
ftr43
ftr50
ftr43
ftr50
CABZ01034854.1
4640000
4660000
4680000
4700000
4720000
chr 3

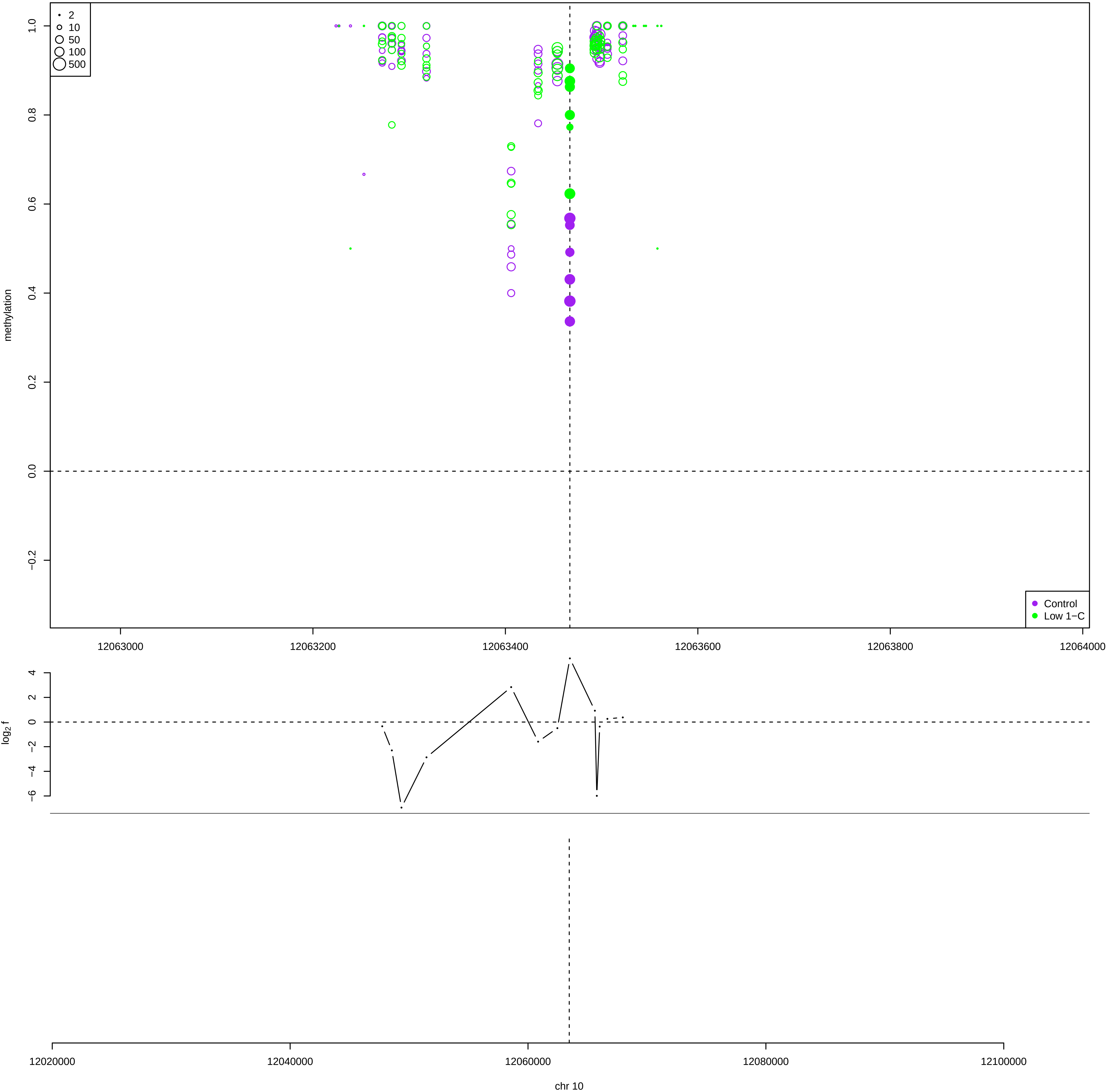

2
10
50
100
500
Control
Low 1−C
methylation
1.0
0.8
0.6
0.4
0.2
0.0
−0.2
12063000
12063200
12063400
12063600
12063800
12064000
$\log_2 f$
4
2
0
−2
−4
−6
12020000
12040000
12060000
12080000
12100000
chr 10

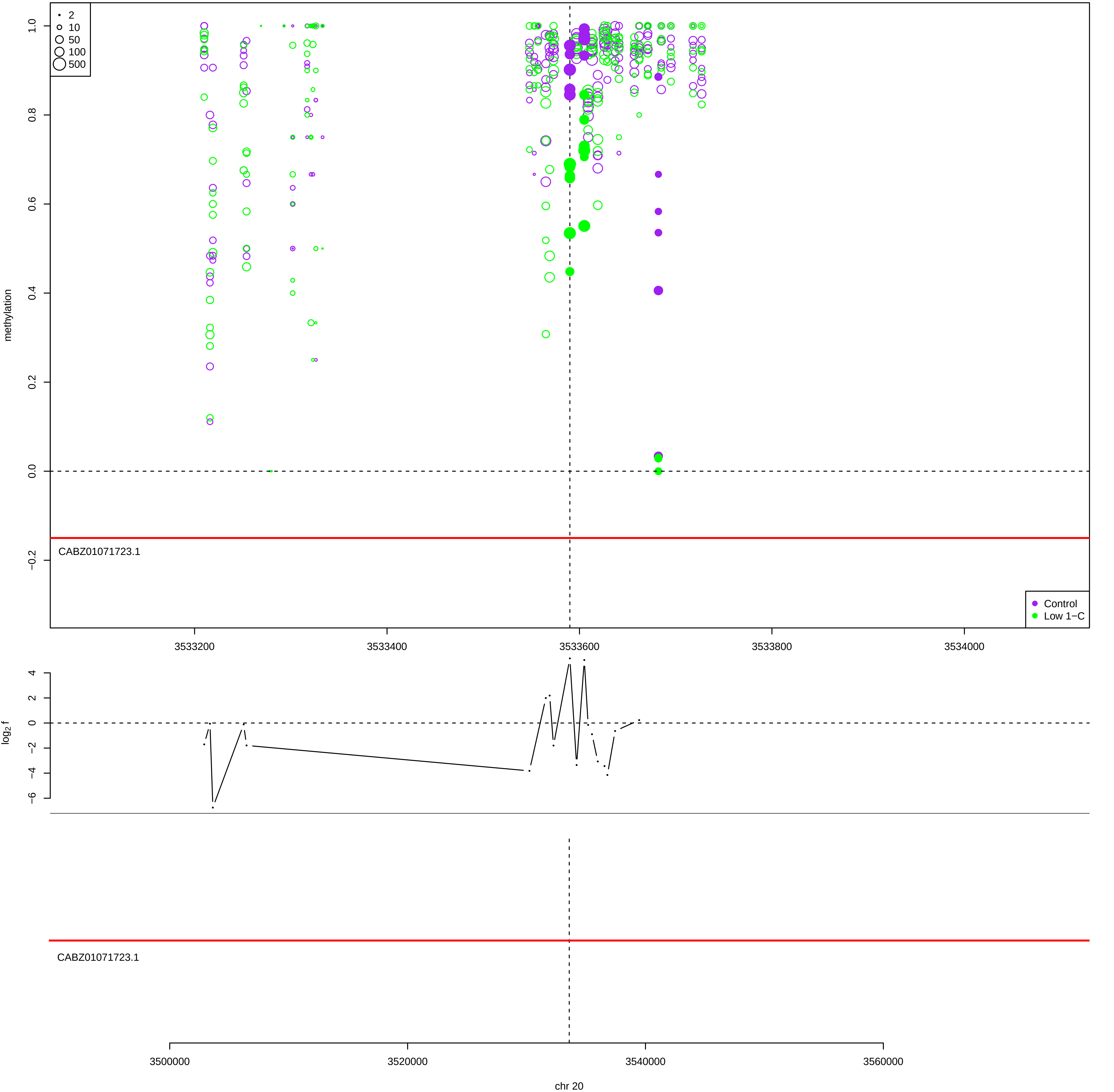

methylation
2
10
50
100
500
Control
Low 1−C
CABZ01071723.1
3533200
3533400
3533600
3533800
3534000
log2 f
CABZ01071723.1
3500000
3520000
3540000
3560000
chr 20

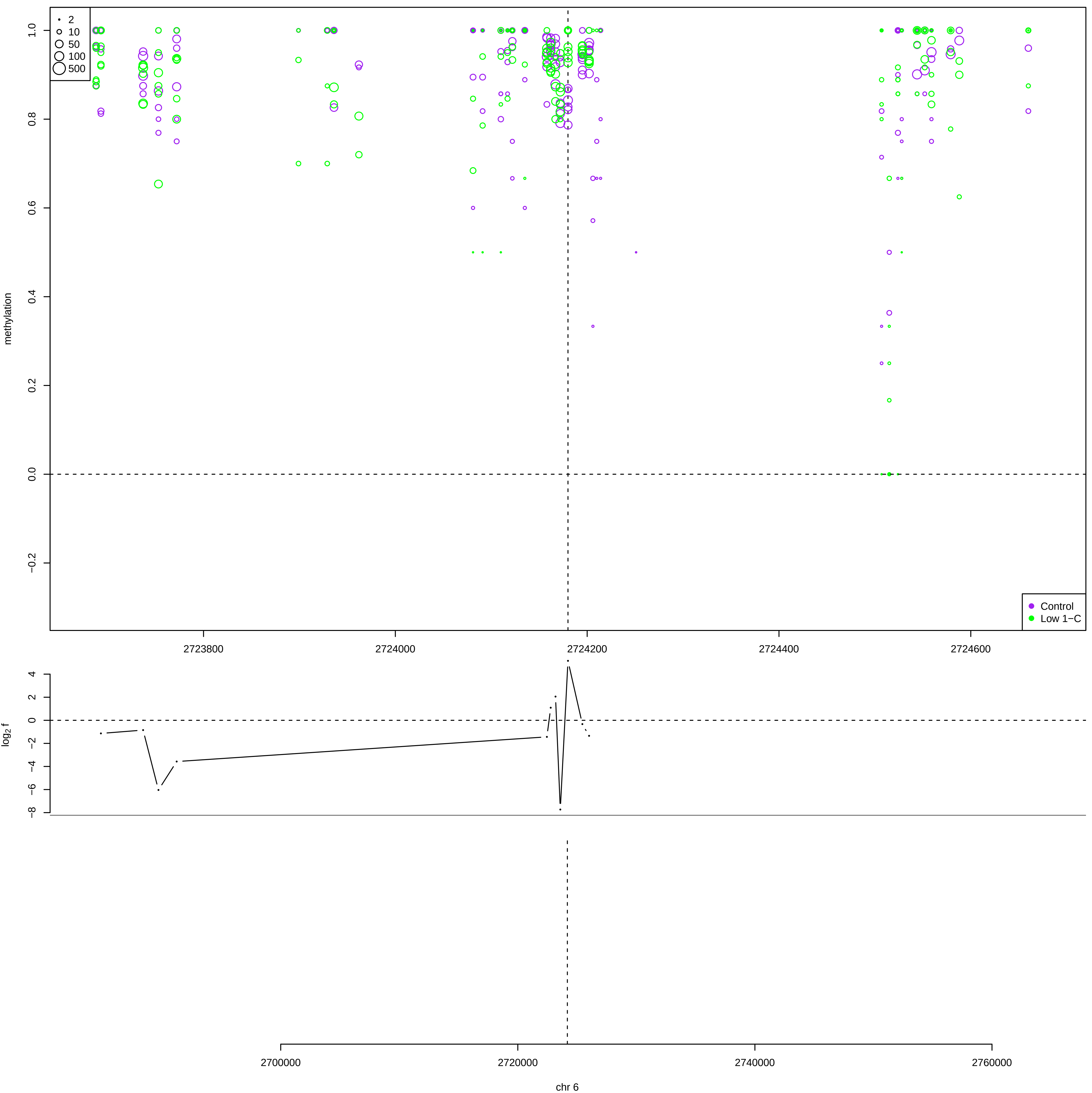

2
10
50
100
500
methylation
1.0
0.8
0.6
0.4
0.2
0.0
−0.2
Control
Low 1−C
2723800
2724000
2724200
2724400
2724600
log2 f
4
2
0
−2
−4
−6
−8
2700000
2720000
2740000
2760000
chr 6

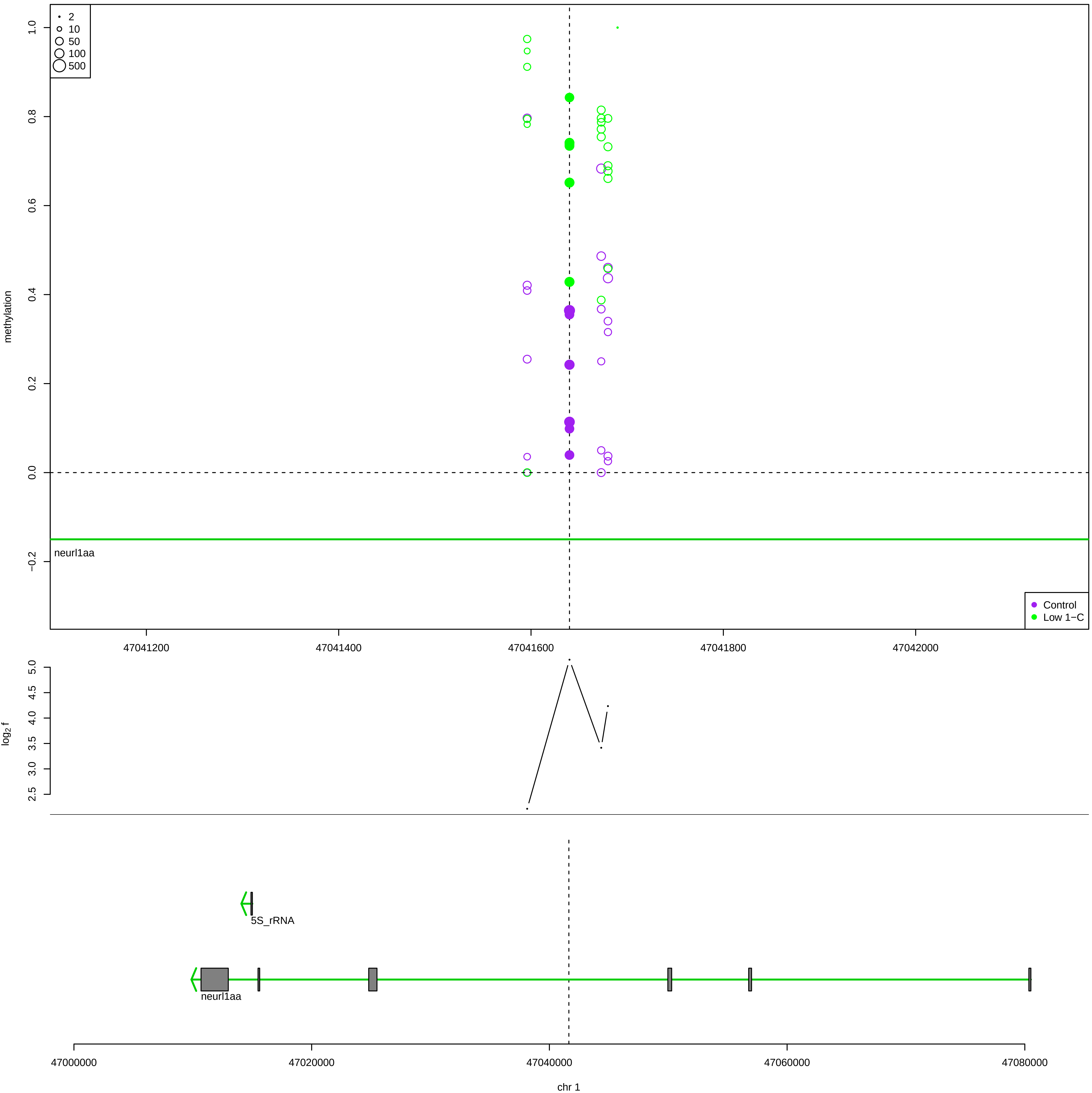

2
10
50
100
500
methylation
1.0
0.8
0.6
0.4
0.2
0.0
−0.2
neurl1aa
Control
Low 1−C
47041200
47041400
47041600
47041800
47042000
$\log_2 f$
5.0
4.5
4.0
3.5
3.0
2.5
5S_rRNA
neurl1aa
47000000
47020000
47040000
47060000
47080000
chr 1

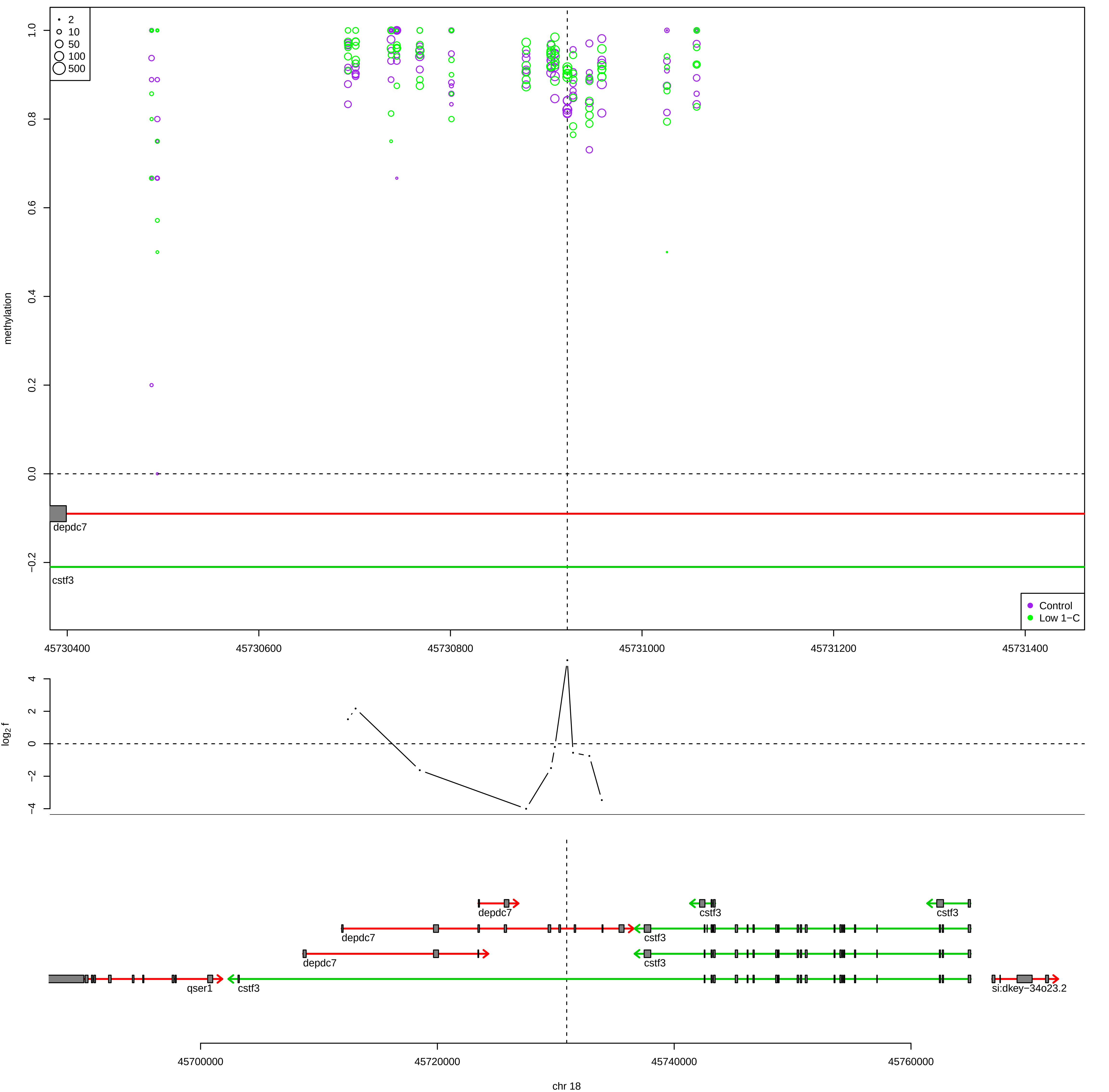

2
10
50
100
500
methylation
1.0
0.8
0.6
0.4
0.2
0.0
−0.2
depdc7
cstf3
Control
Low 1−C
45730400
45730600
45730800
45731000
45731200
45731400
log2 f
4
2
0
-2
-4
depdc7
cstf3
depdc7
cstf3
depdc7
cstf3
qser1
cstf3
si:dkey−34o23.2
45700000
45720000
45740000
45760000
chr 18

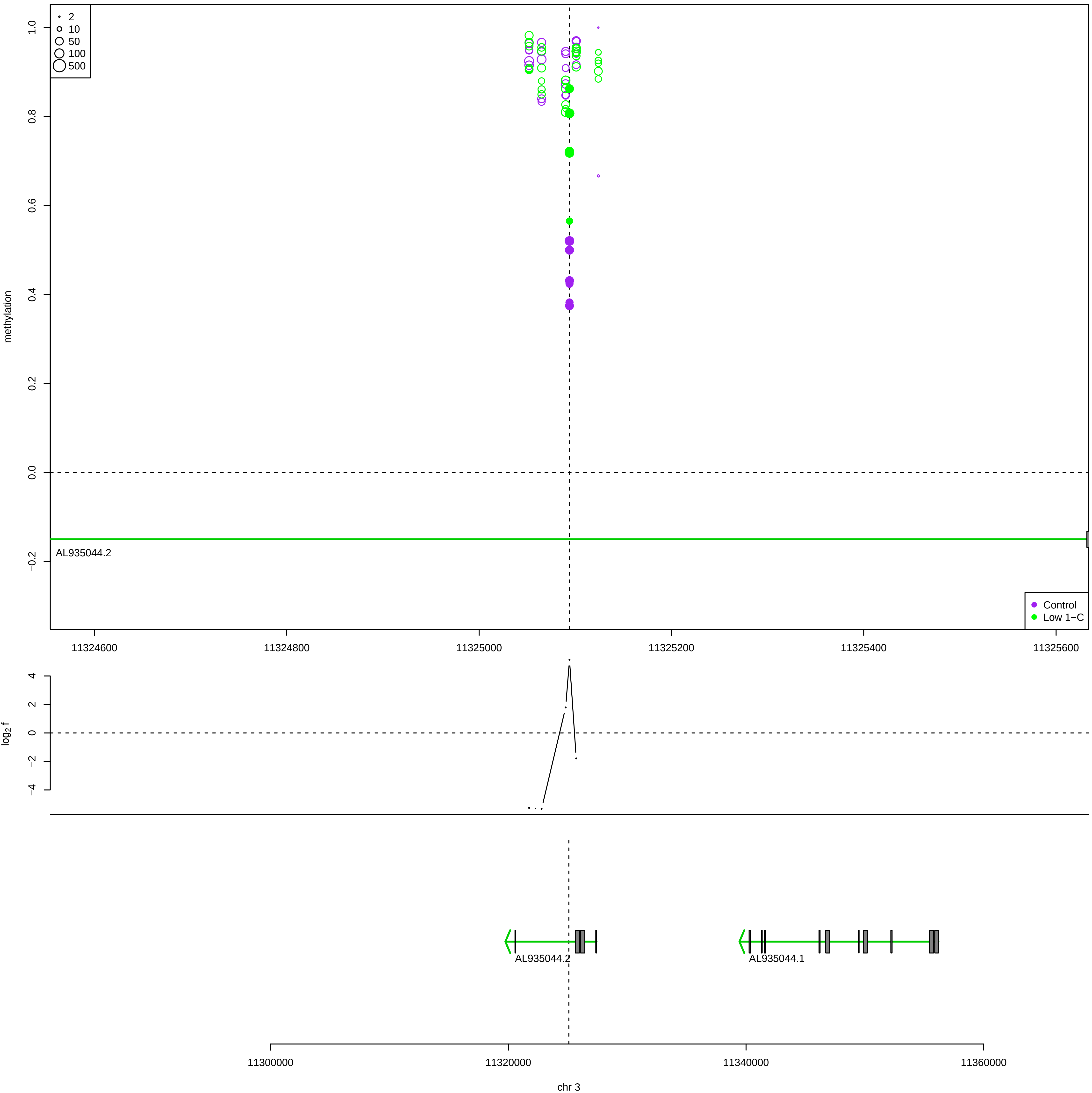

2
10
50
100
500
methylation
1.0
0.8
0.6
0.4
0.2
0.0
−0.2
AL935044.2
Control
Low 1−C
11324600
11324800
11325000
11325200
11325400
11325600
log2 f
4
2
0
−2
−4
AL935044.2
AL935044.1
11300000
11320000
11340000
11360000
chr 3

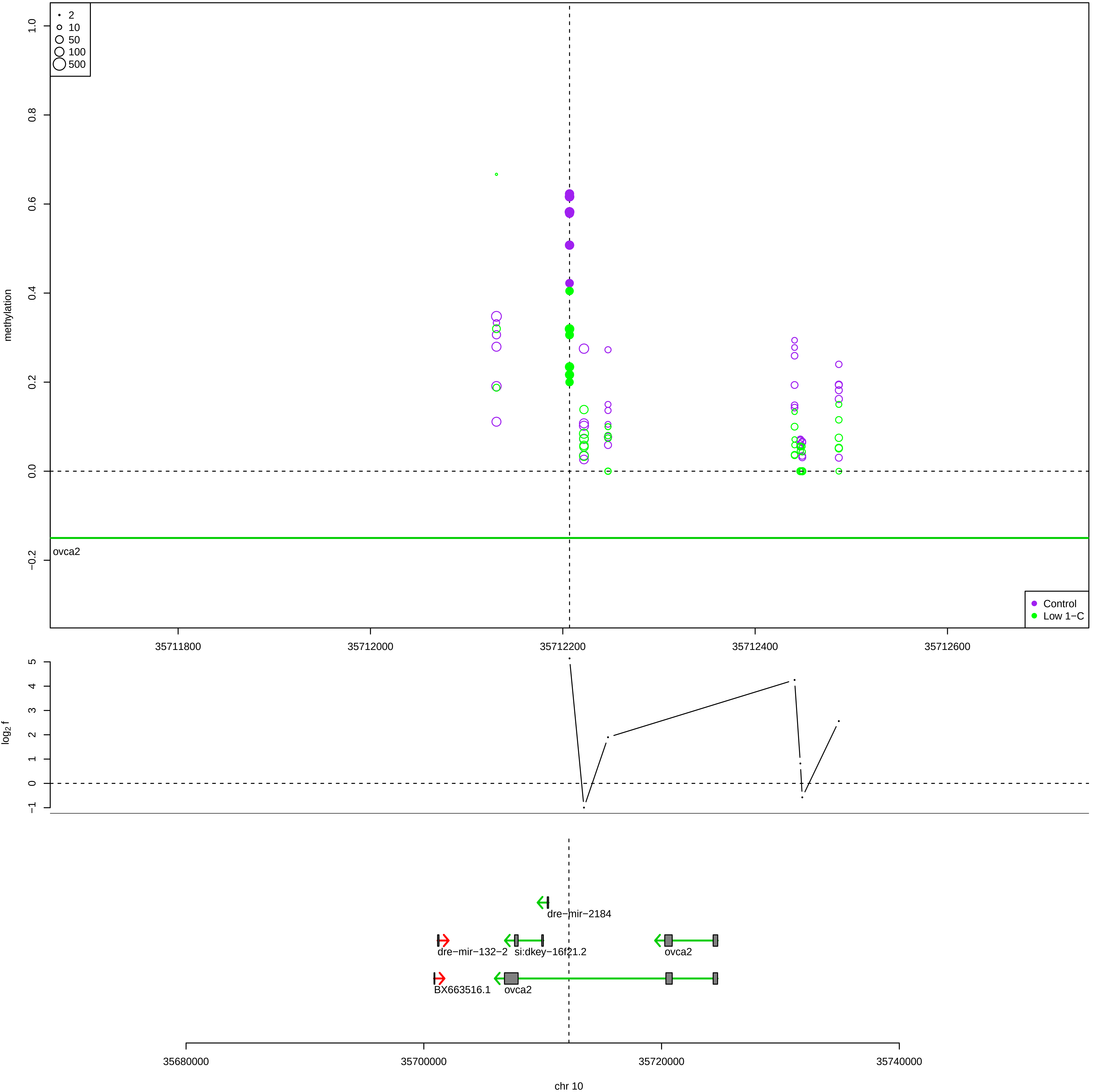

2
10
50
100
500
methylation
1.0
0.8
0.6
0.4
0.2
0.0
−0.2
ovca2
Control
Low 1−C
35711800
35712000
35712200
35712400
35712600
log2 f
5
4
3
2
1
0
−1
dre−mir−2184
dre−mir−132−2
si:dkey−16f21.2
ovca2
BX663516.1
ovca2
35680000
35700000
35720000
35740000
chr 10

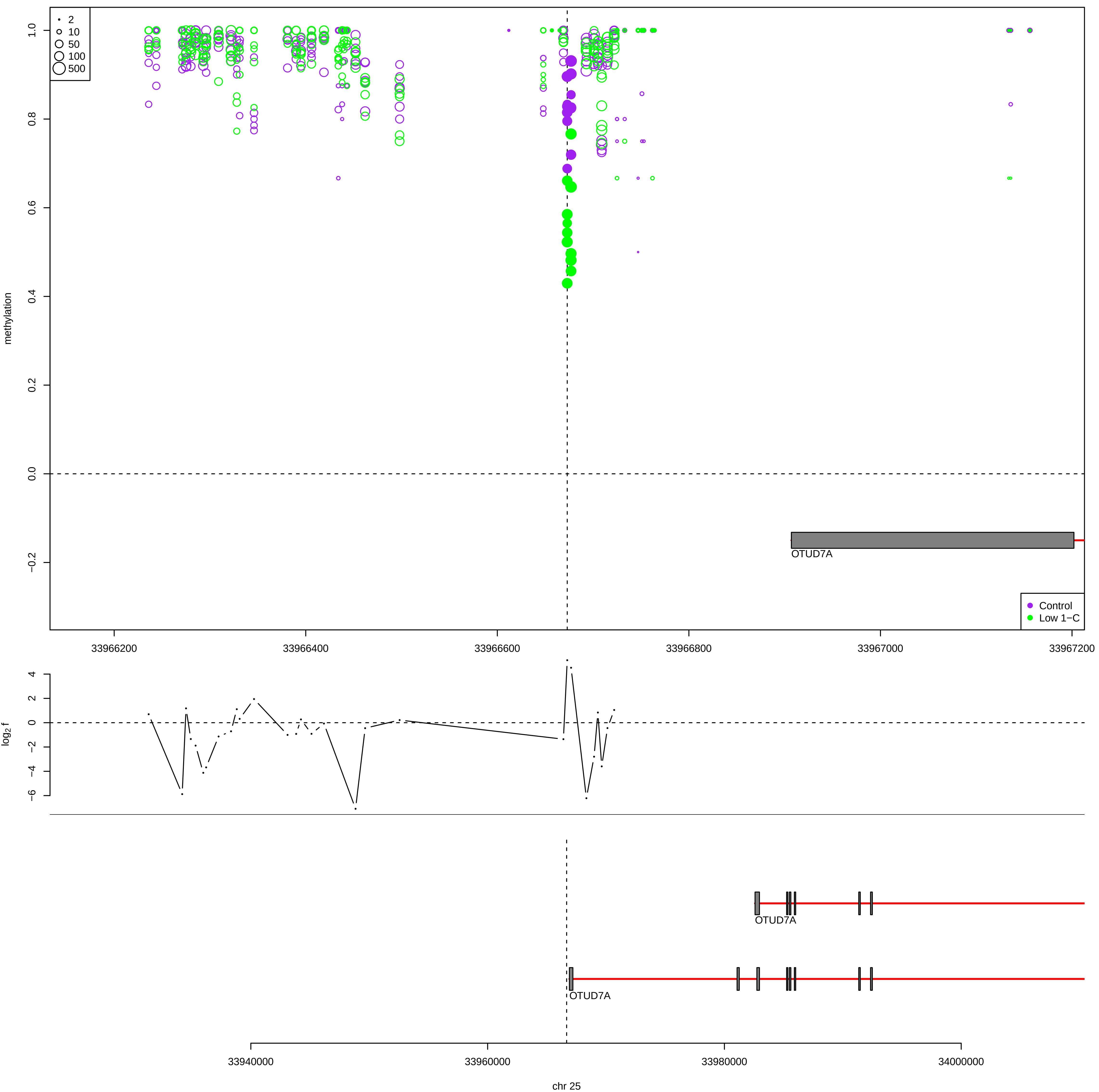

methylation
2
10
50
100
500
1.0
0.8
0.6
0.4
0.2
0.0
-0.2
OTUD7A
Control
Low 1−C
33966200
33966400
33966600
33966800
33967000
33967200
log2 f
4
2
0
−2
−4
−6
OTUD7A
OTUD7A
33940000
33960000
33980000
34000000
chr 25

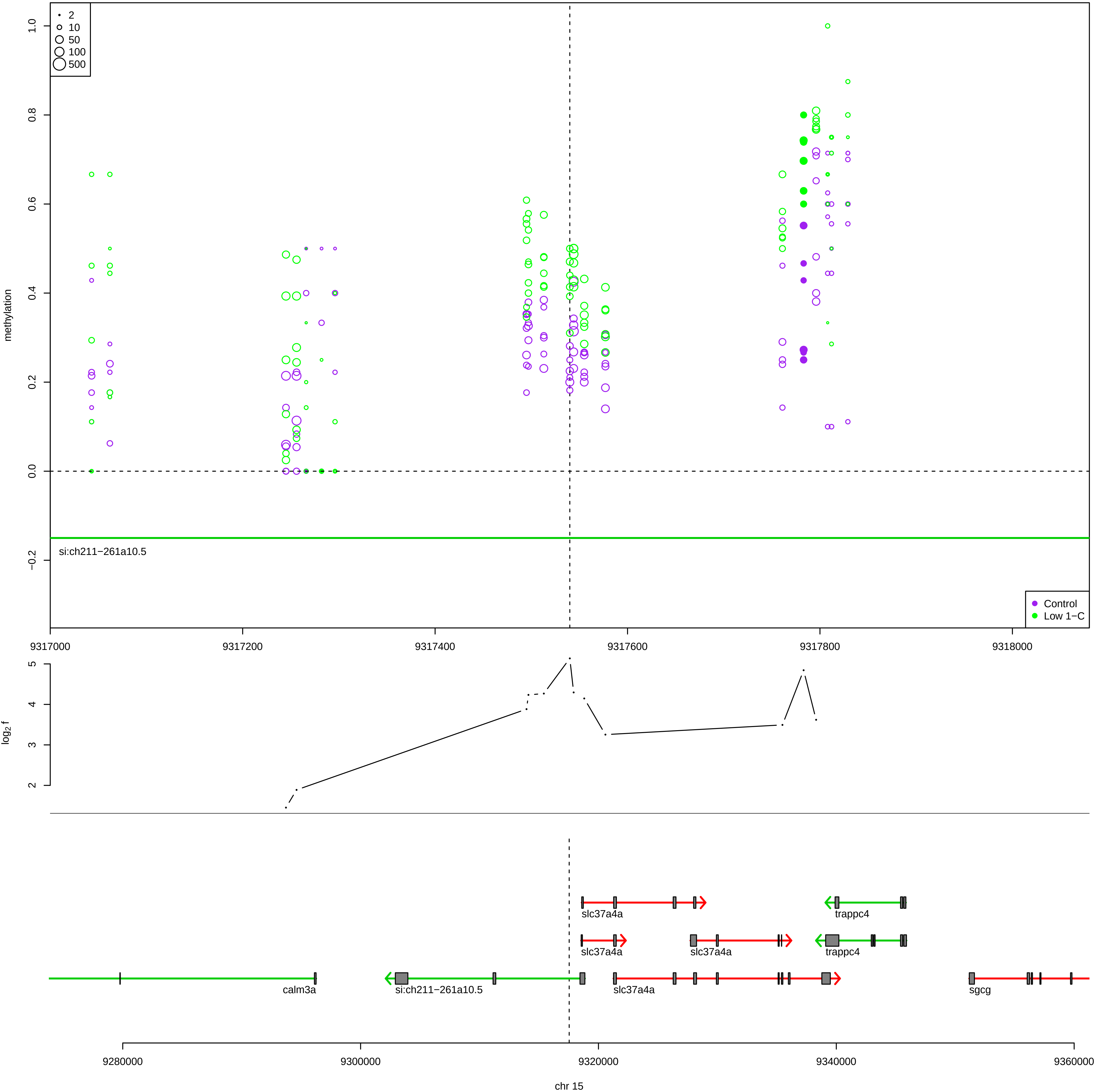

2
10
50
100
500
methylation
1.0
0.8
0.6
0.4
0.2
0.0
−0.2
si:ch211−261a10.5
Control
Low 1−C
9317000
9317200
9317400
9317600
9317800
9318000
log2 f
5
4
3
2
slc37a4a
trappc4
slc37a4a
slc37a4a
trappc4
calm3a
si:ch211−261a10.5
slc37a4a
sgcg
9280000
9300000
9320000
9340000
9360000
chr 15

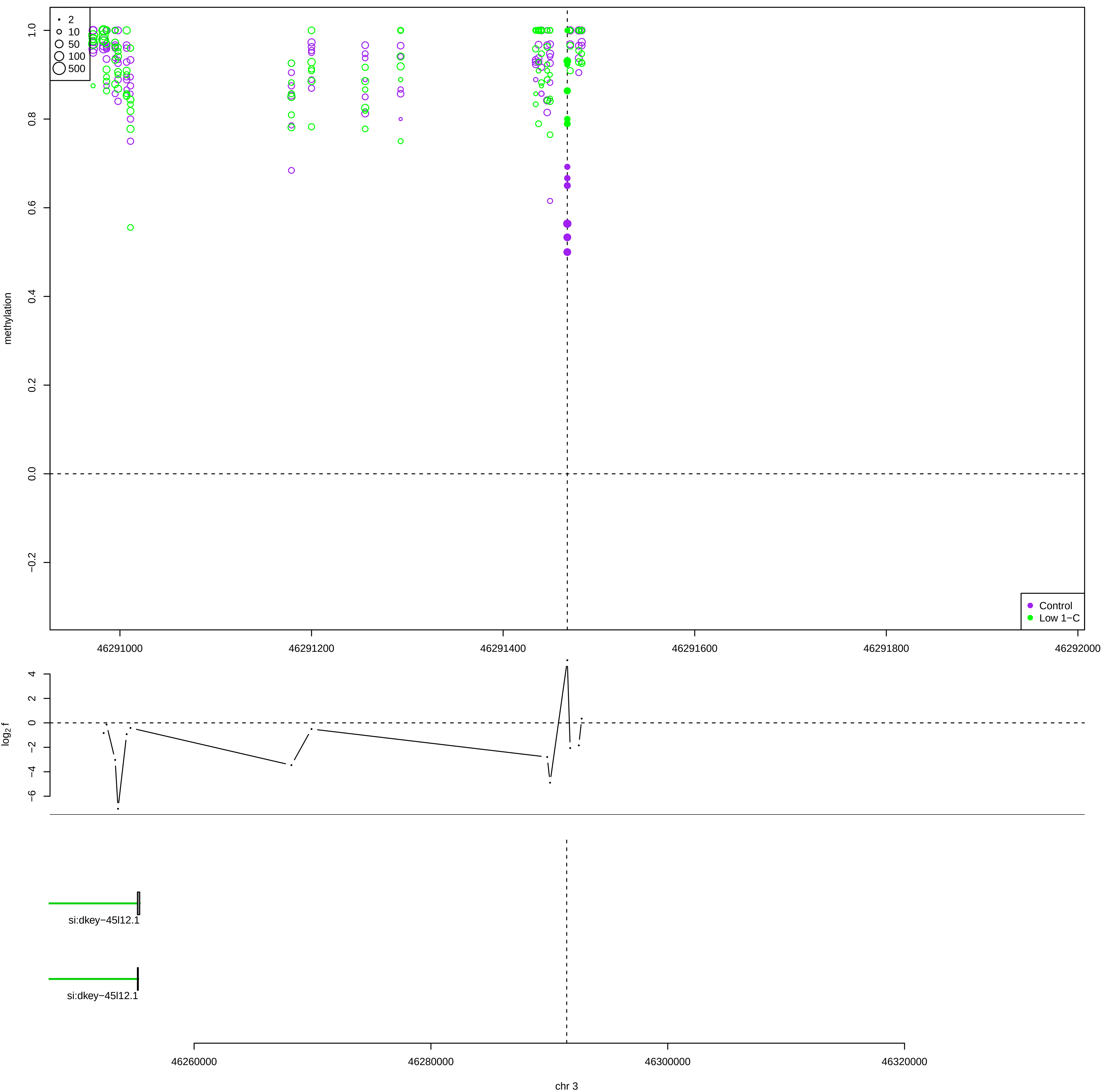

2
10
50
100
500
Control
Low 1−C
methylation
1.0
0.8
0.6
0.4
0.2
0.0
−0.2
46291000
46291200
46291400
46291600
46291800
46292000
log2 f
4
2
0
−2
−4
−6
si:dkey−45l12.1
si:dkey−45l12.1
46260000
46280000
46300000
46320000
chr 3

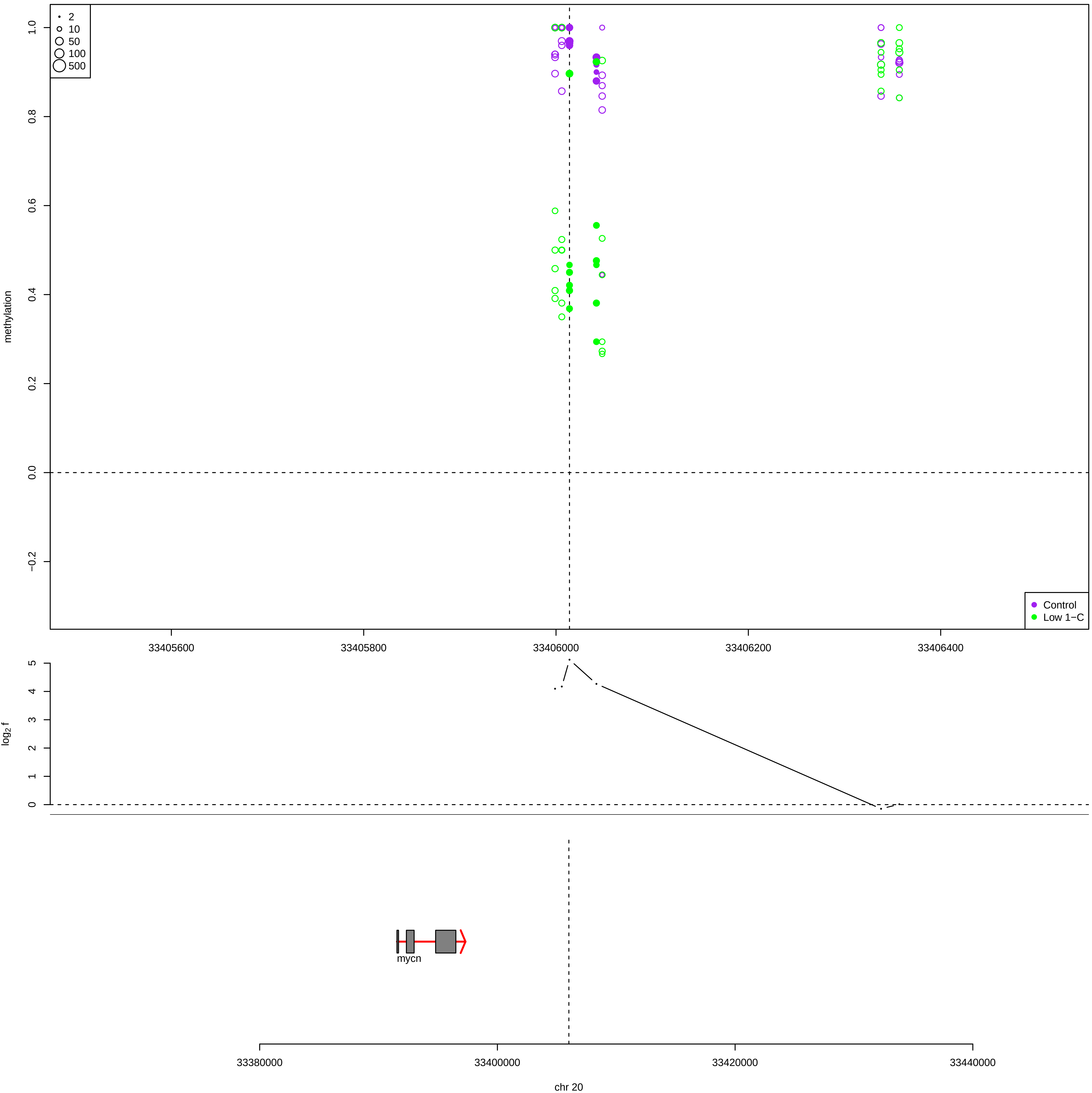

2
10
50
100
500
Control
Low 1−C
methylation
1.0
0.8
0.6
0.4
0.2
0.0
−0.2
33405600
33405800
33406000
33406200
33406400
log2 f
5
4
3
2
1
0
mycn
33380000
33400000
33420000
33440000
chr 20

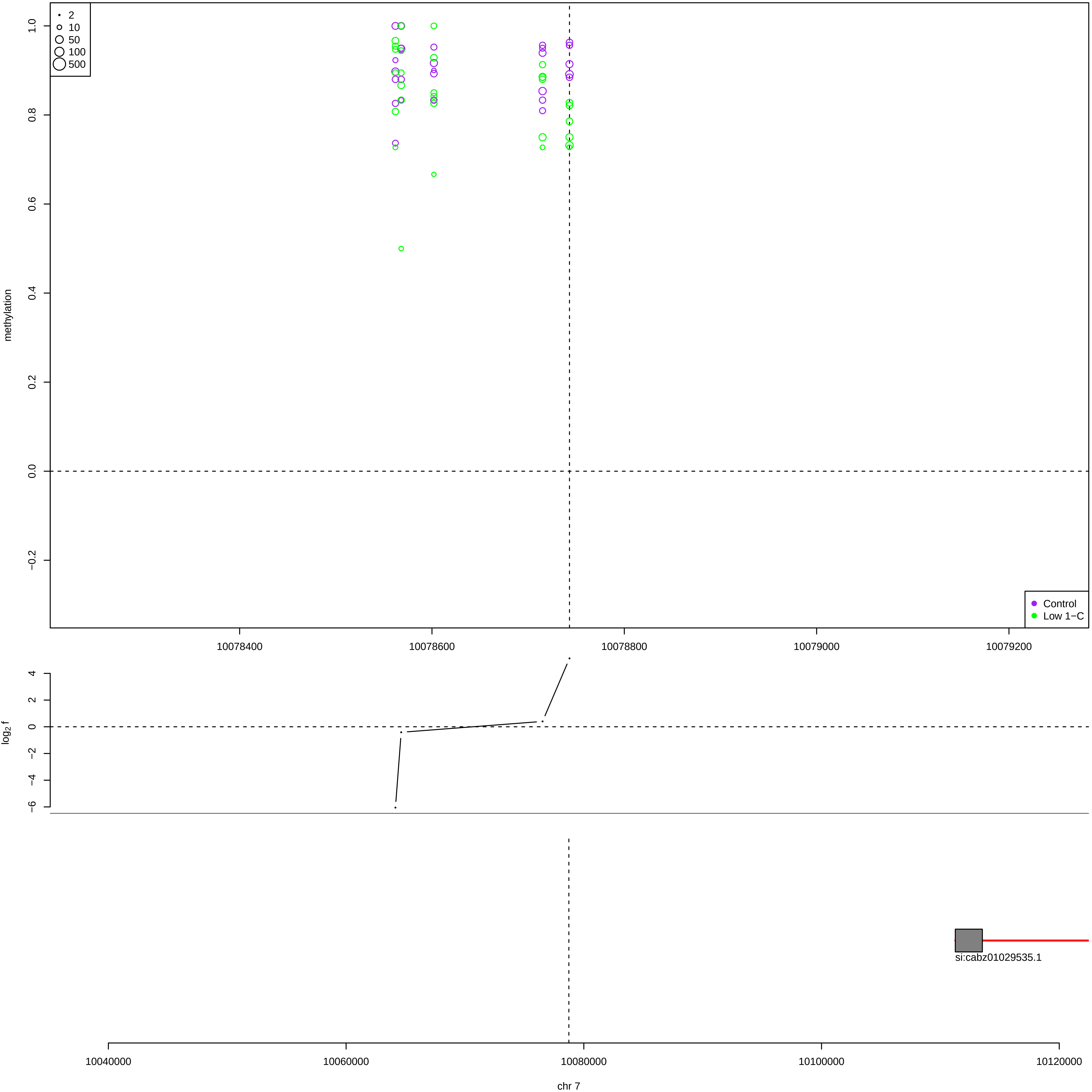

methylation
1.0
0.8
0.6
0.4
0.2
0.0
−0.2
2
10
50
100
500
Control
Low 1−C
10078400
10078600
10078800
10079000
10079200
log2 f
4
2
0
−2
−4
−6
si:cabz01029535.1
10040000
10060000
10080000
10100000
10120000
chr 7

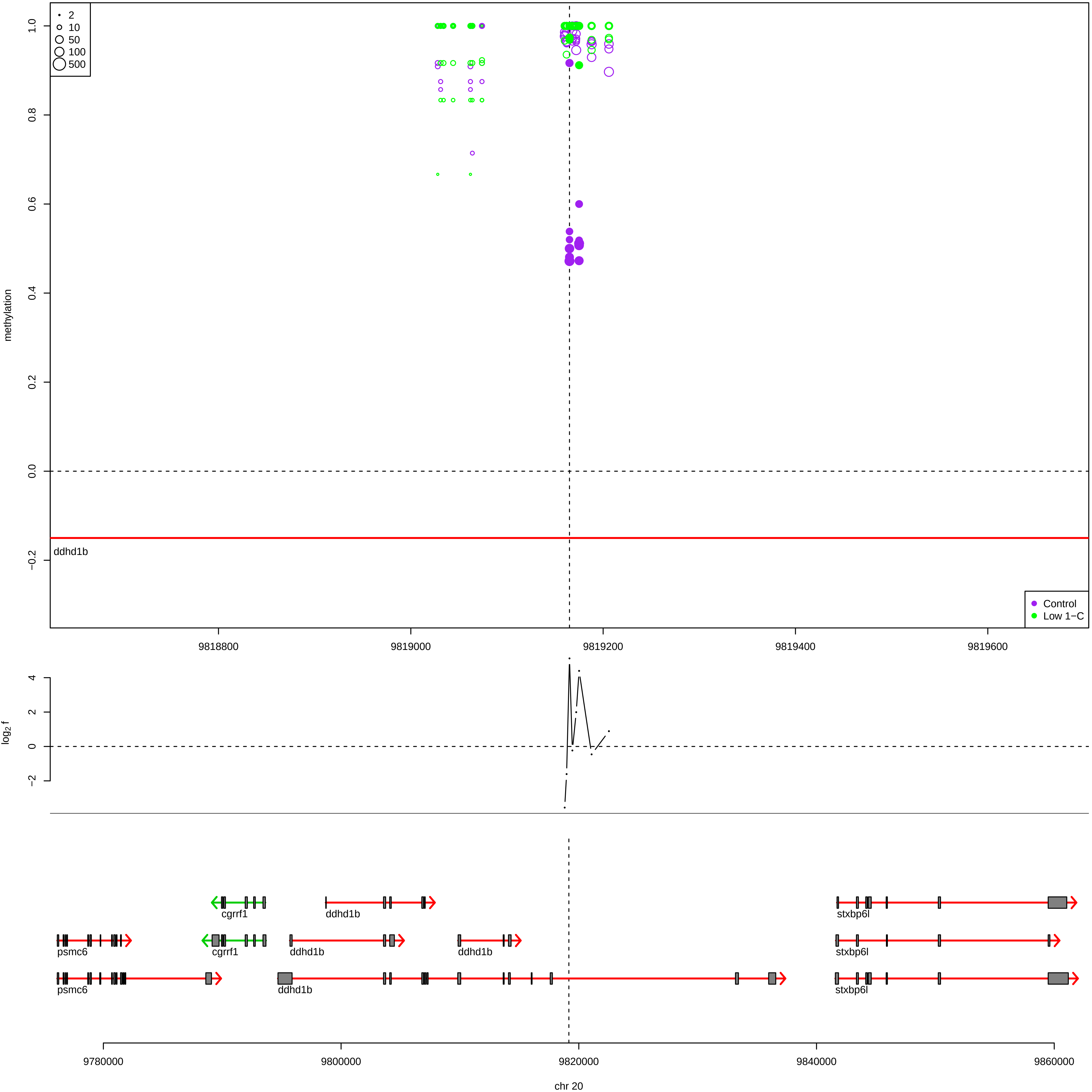

2
10
50
100
500
methylation
1.0
0.8
0.6
0.4
0.2
0.0
−0.2
ddhd1b
Control
Low 1−C
9818800
9819000
9819200
9819400
9819600
log2 f
4
2
0
−2
cgrrf1
ddhd1b
stxbp6l
psmc6
cgrrf1
ddhd1b
ddhd1b
stxbp6l
psmc6
ddhd1b
stxbp6l
9780000
9800000
9820000
9840000
9860000
chr 20

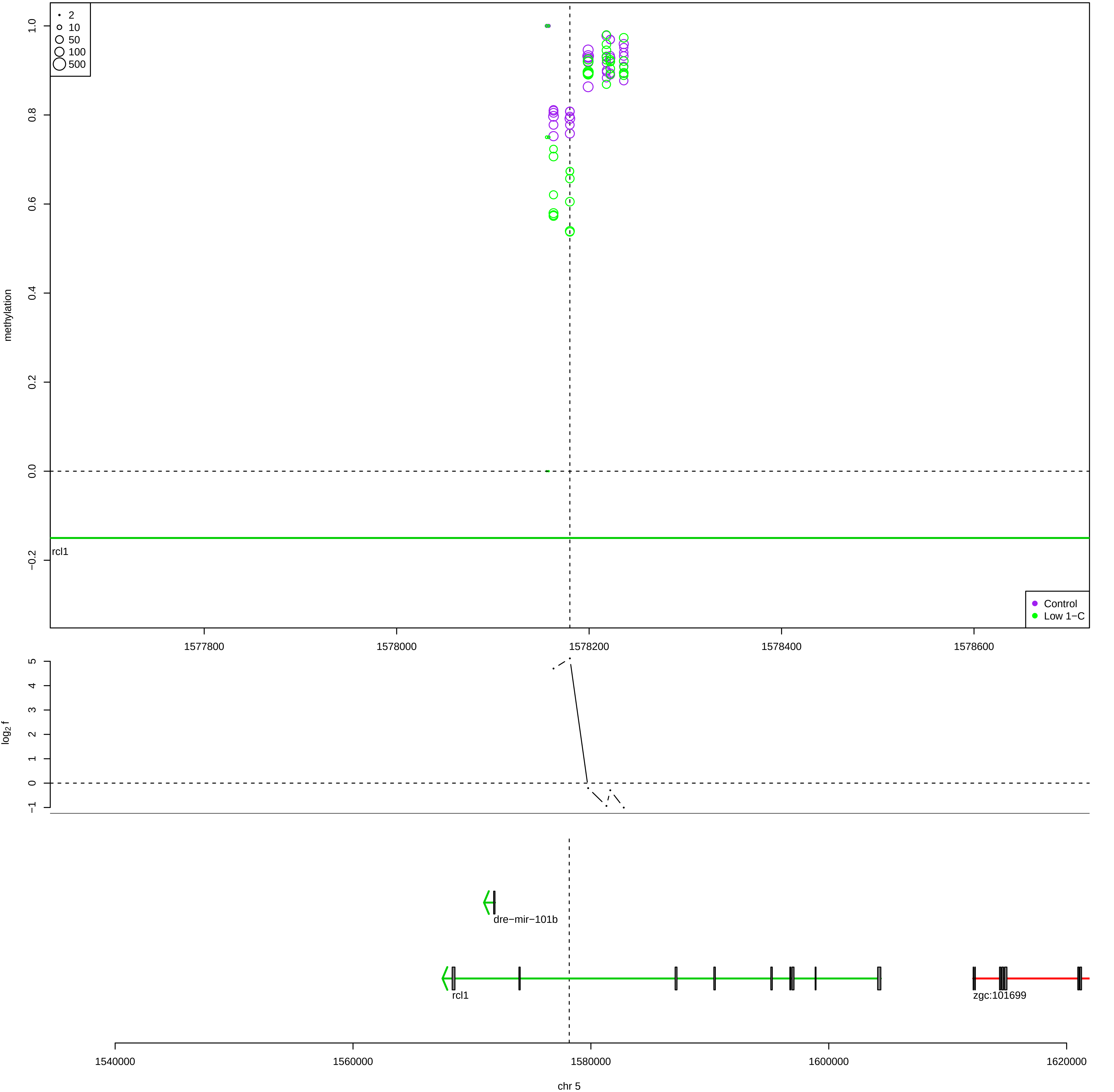

2
10
50
100
500
methylation
1.0
0.8
0.6
0.4
0.2
0.0
−0.2
rcl1
Control
Low 1−C
1577800
1578000
1578200
1578400
1578600
log2 f
5
4
3
2
1
0
−1
dre−mir−101b
rcl1
zgc:101699
1540000
1560000
1580000
1600000
1620000
chr 5

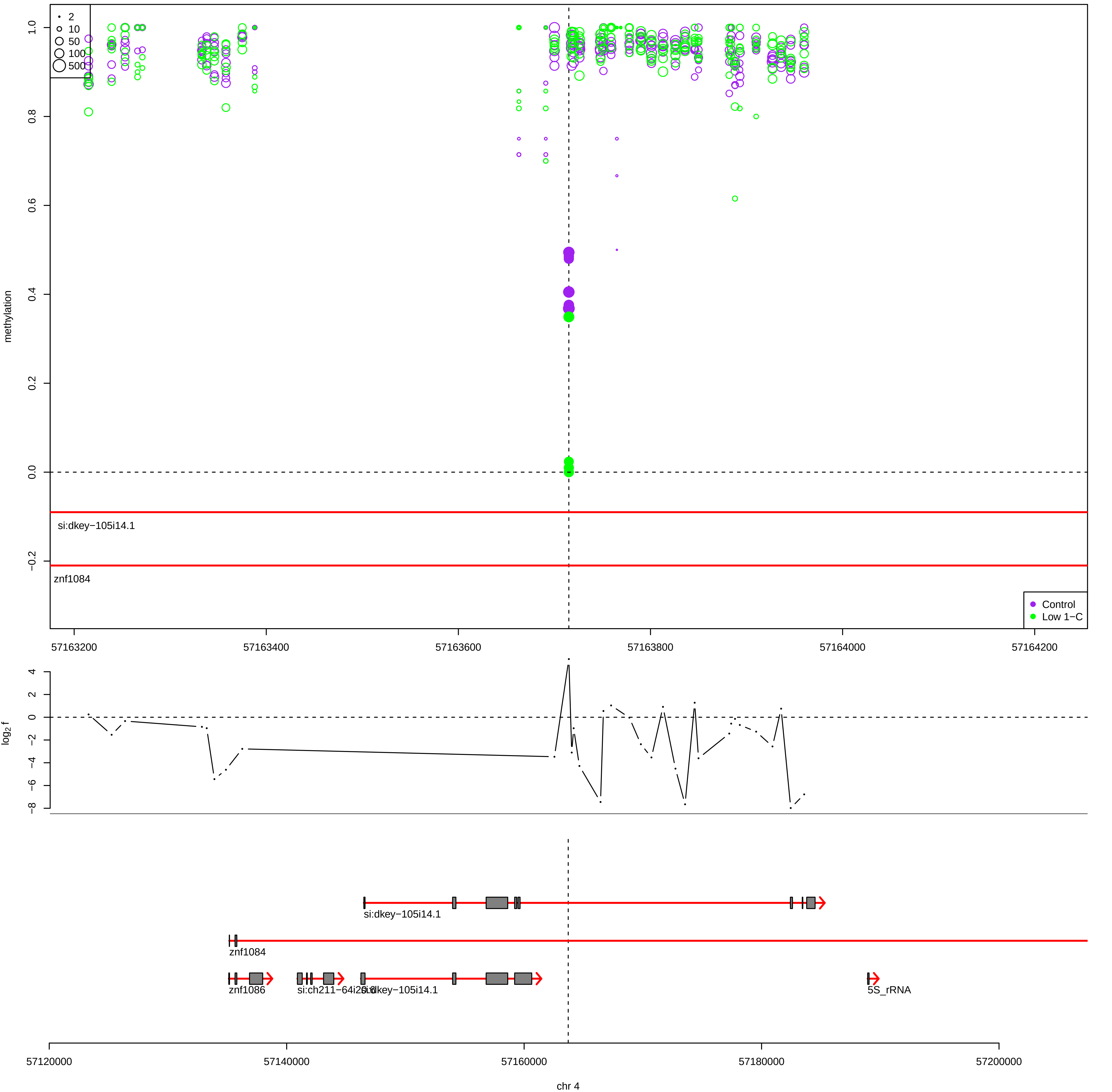

2
10
50
100
500
methylation
1.0
0.8
0.6
0.4
0.2
0.0
−0.2
si:dkey−105i14.1
znf1084
Control
Low 1−C
57163200
57163400
57163600
57163800
57164000
57164200
log2 f
4
2
0
−2
−4
−6
−8
si:dkey−105i14.1
znf1084
znf1086
si:ch211−64i20.6
si:dkey−105i14.1
5S_rRNA
57120000
57140000
57160000
57180000
57200000
chr 4

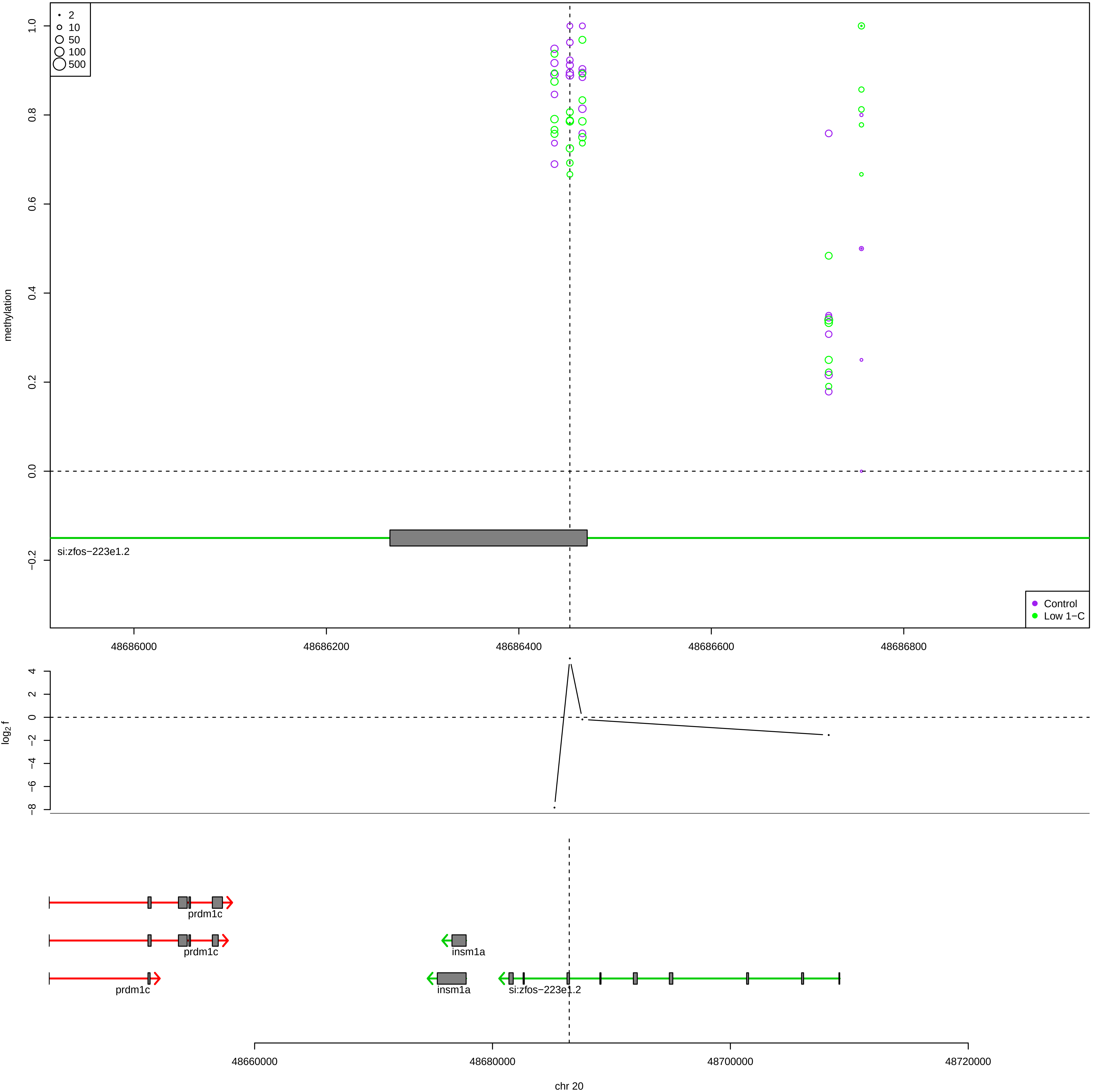

2
10
50
100
500
methylation
1.0
0.8
0.6
0.4
0.2
0.0
-0.2
si:zfos-223e1.2
Control
Low 1-C
48686000
48686200
48686400
48686600
48686800
$\log_2 f$
4
2
0
-2
-4
-6
-8
prdm1c
prdm1c
insm1a
prdm1c
insm1a
si:zfos-223e1.2
48660000
48680000
48700000
48720000
chr 20

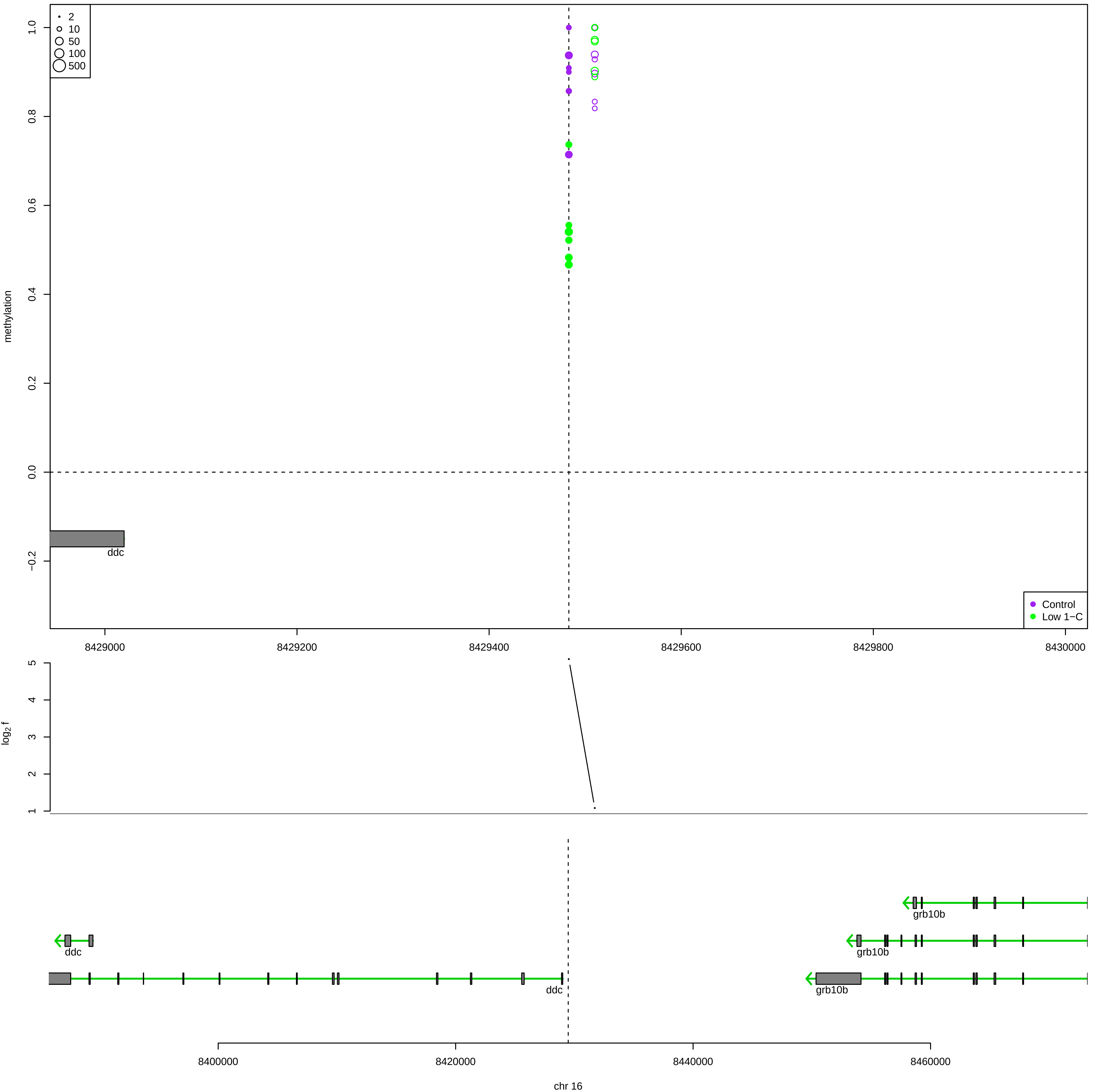

methylation
1.0
0.8
0.6
0.4
0.2
0.0
−0.2
2
10
50
100
500
ddc
Control
Low 1−C
8429000
8429200
8429400
8429600
8429800
8430000
log2 f
5
4
3
2
1
grb10b
grb10b
grb10b
ddc
ddc
8400000
8420000
8440000
8460000
chr 16

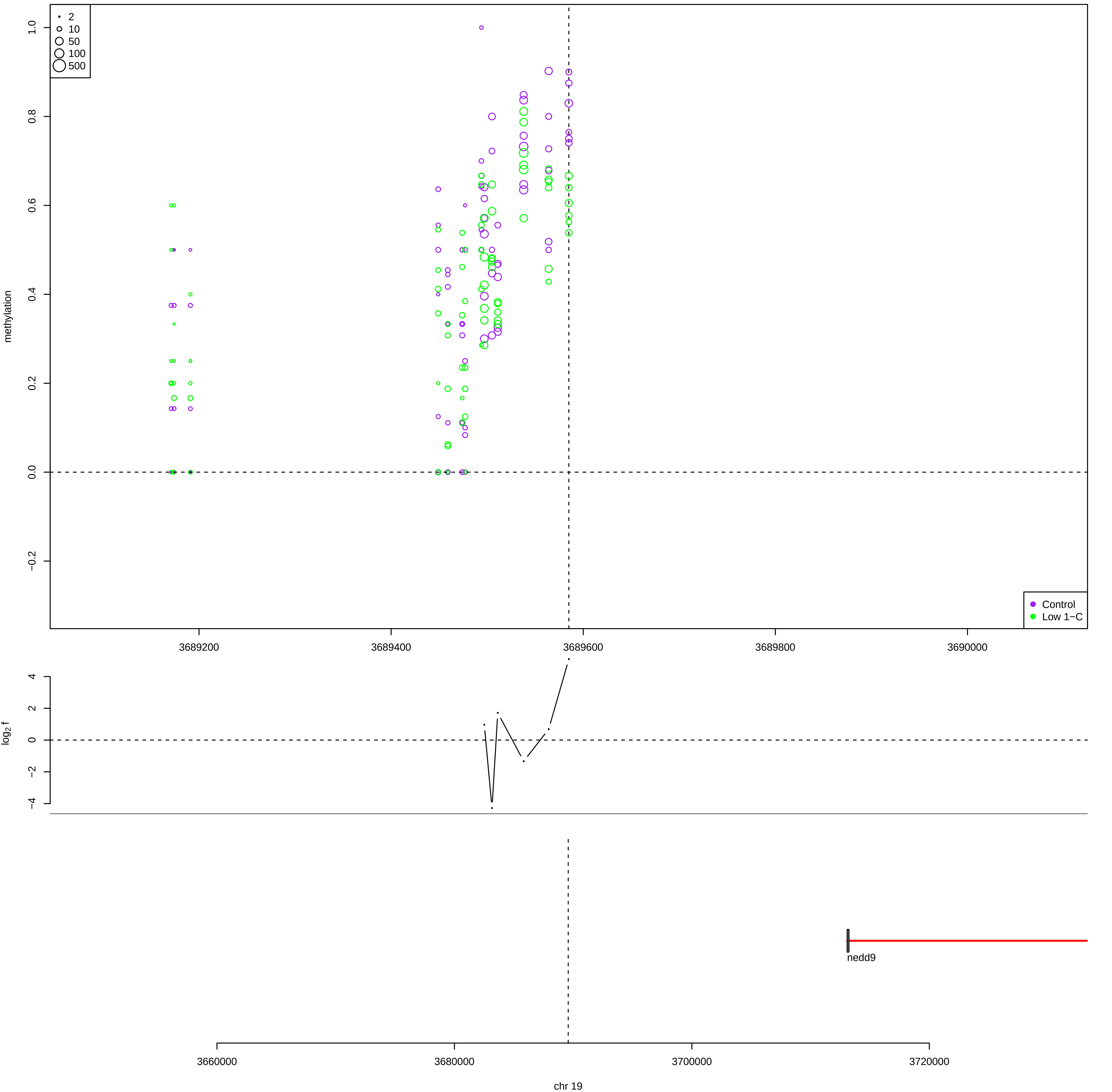

2
10
50
100
500
1.0
0.8
0.6
0.4
0.2
0.0
−0.2
methylation
Control
Low 1−C
3689200
3689400
3689600
3689800
3690000
4
2
0
−2
−4
$\log_2 f$
nedd9
3660000
3680000
3700000
3720000
chr 19

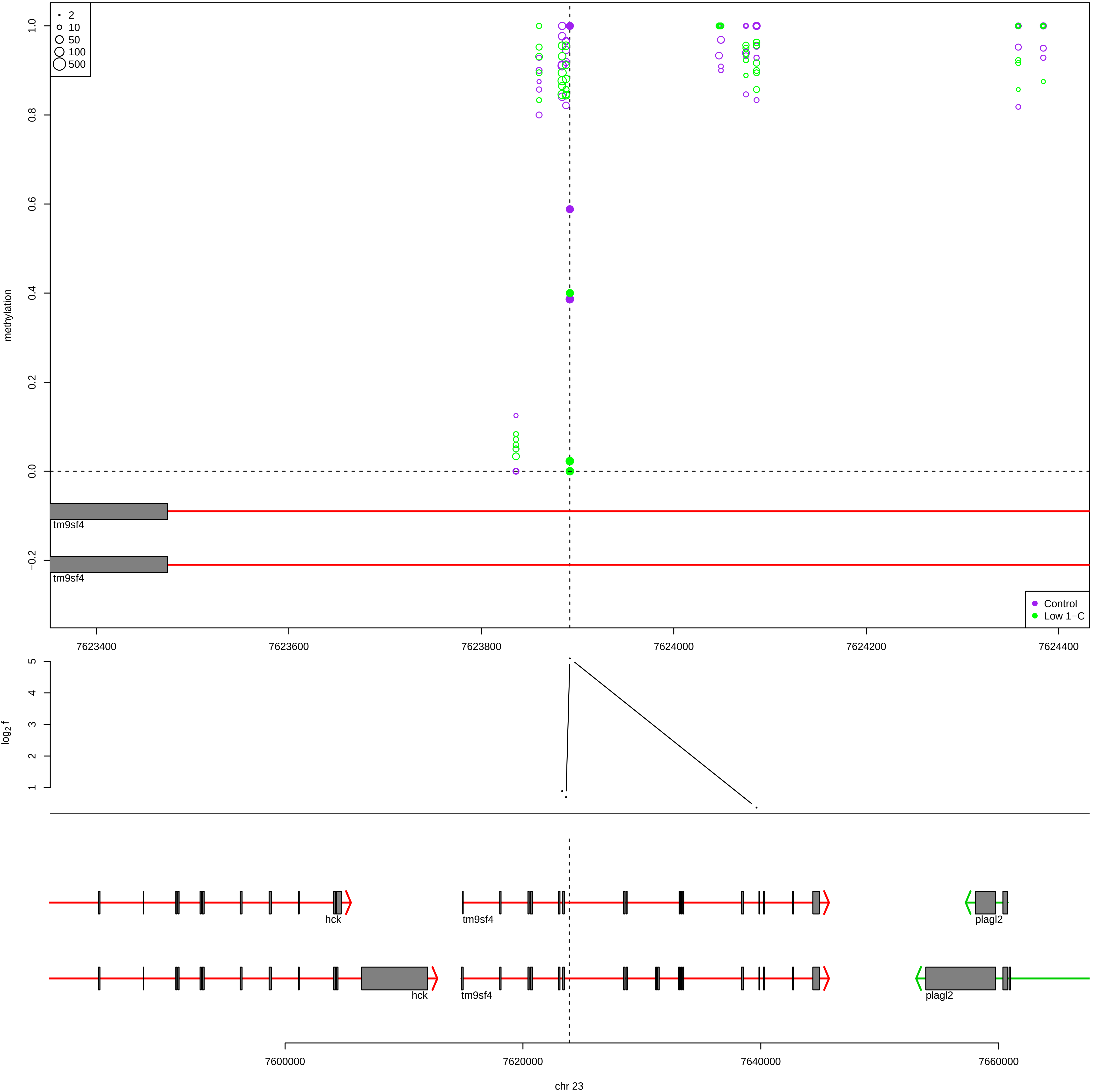

2
10
50
100
500
methylation
1.0
0.8
0.6
0.4
0.2
0.0
−0.2
tm9sf4
tm9sf4
Control
Low 1−C
7623400
7623600
7623800
7624000
7624200
7624400
log_2 f
5
4
3
2
1
hck
tm9sf4
plagl2
hck
tm9sf4
plagl2
7600000
7620000
7640000
7660000
chr 23
